# Supplementary material for: In Vitro Anti-Influenza Virus Activity of Non-Polar Primula veris subsp. veris Extract
Source: Pharmaceuticals (Basel). 2022 Dec 5;15(12):1513. doi: 10.3390/ph15121513 (PMC9787935; doi:10.3390/ph15121513)
Supplement: Supplementary file 1 [file pharmaceuticals-15-01513-s001.zip › pharmaceuticals-2035816-supplementary.pdf]

# In Vitro Anti-Influenza Virus Activity of Non-Polar *Primula veris* subsp. *veris* Extract

**Aristides G. Eliopoulos [1,2,*], Apostolis Angelis [3], Anastasia Liakakou [3] and Leandros A. Skaltsounis [3,*]**

[1] Department of Biology, School of Medicine, National and Kapodistrian University of Athens, 11527 Athens, Greece
[2] Center of Basic Research, Biomedical Research Foundation of the Academy of Athens, 11527 Athens, Greece
[3] Department of Pharmacy, Division of Pharmacognosy and Natural Products Chemistry, National and Kapodistrian University of Athens, 15771 Athens, Greece
* Correspondence: eliopag@med.uoa.gr (A.G.E.); skaltsounis@pharm.uoa.gr (L.A.S.)

**Contents:**

Fig. S1: GC-MS analysis of non-polar extract of *P. veris* subsp. *veris*.
Fig. S2: GC-MS analysis of esterified non polar extract of *P. veris* subsp. *veris*.
Fig. S3: TLC chromatograms of combined CPC fractions at UV-254nm and visible after spraying with sulfuric vanillin and heating.

Table S1: The main fatty compounds of non-polar extract of *P. veris* subsp. *veris* identified by GC-MS analysis.
Table S2: The main fatty compounds of esterified non-polar extract of *P. veris* subsp. veris identified by GC-MS analysis.
Table S3: Secondary metabolites identified in non-polar extract of *P. veris* subsp. *veris* by HPLC-HRMS analysis.
Table S4: Gene/Protein terms associated with bioactive chemicals found in *P. veris*.
Table S5: Gene/Protein terms associated with Influenza.
Table S6: Gene/Protein terms associated with the intersection of influenza and phytochemical classes found in *P. veris*.
Table S7: KEGG pathways enriched in the intersection gene/protein terms of Table S6.
Table S8: Reactome pathways enriched in the intersection gene/protein terms of Table S6.
Table S9: Transcription factors predicted to regulate genes/proteins related to the intersection of *P.veris* metabolites and influenza infection.

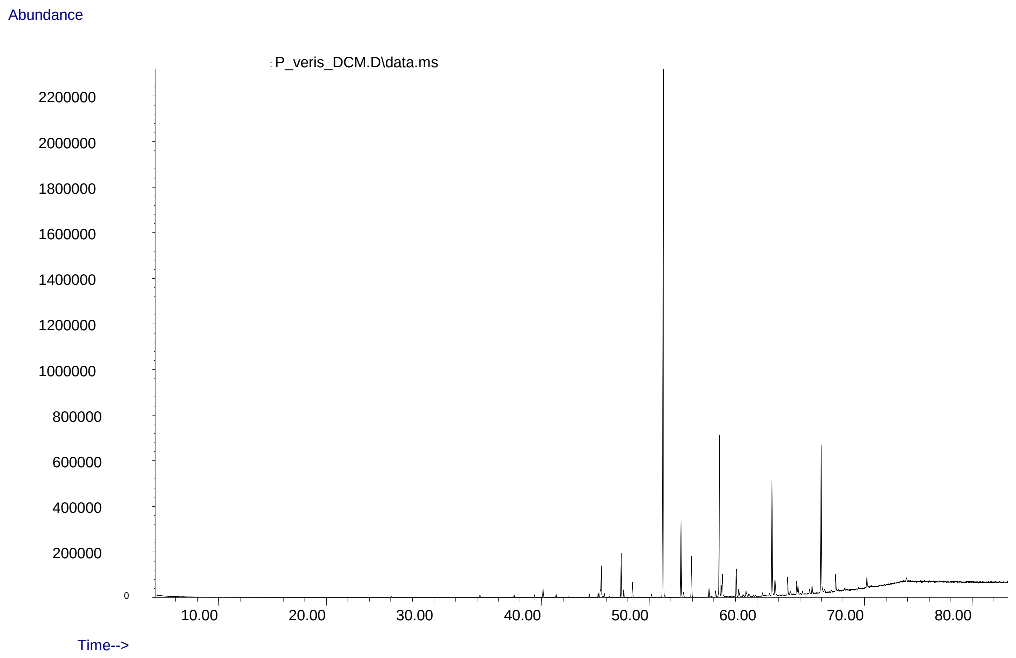


Fig. S1. GC-MS analysis of non-polar extract of *P. veris* subsp. *veris*

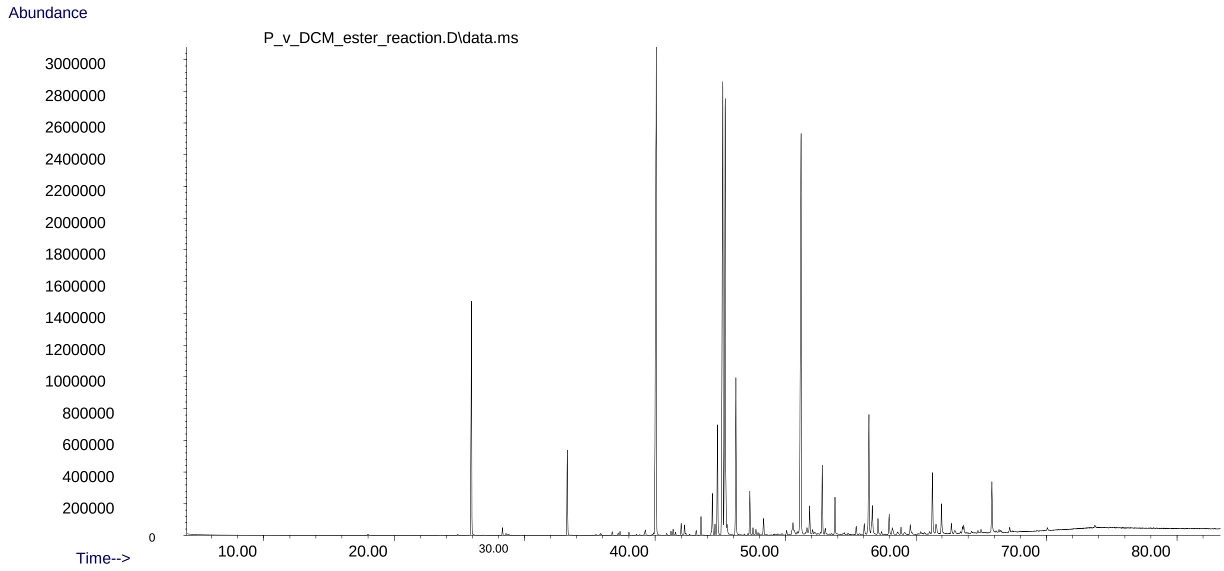


Fig. S2: GC-MS analysis of esterified non polar extract of *P. veris* subsp. *veris*

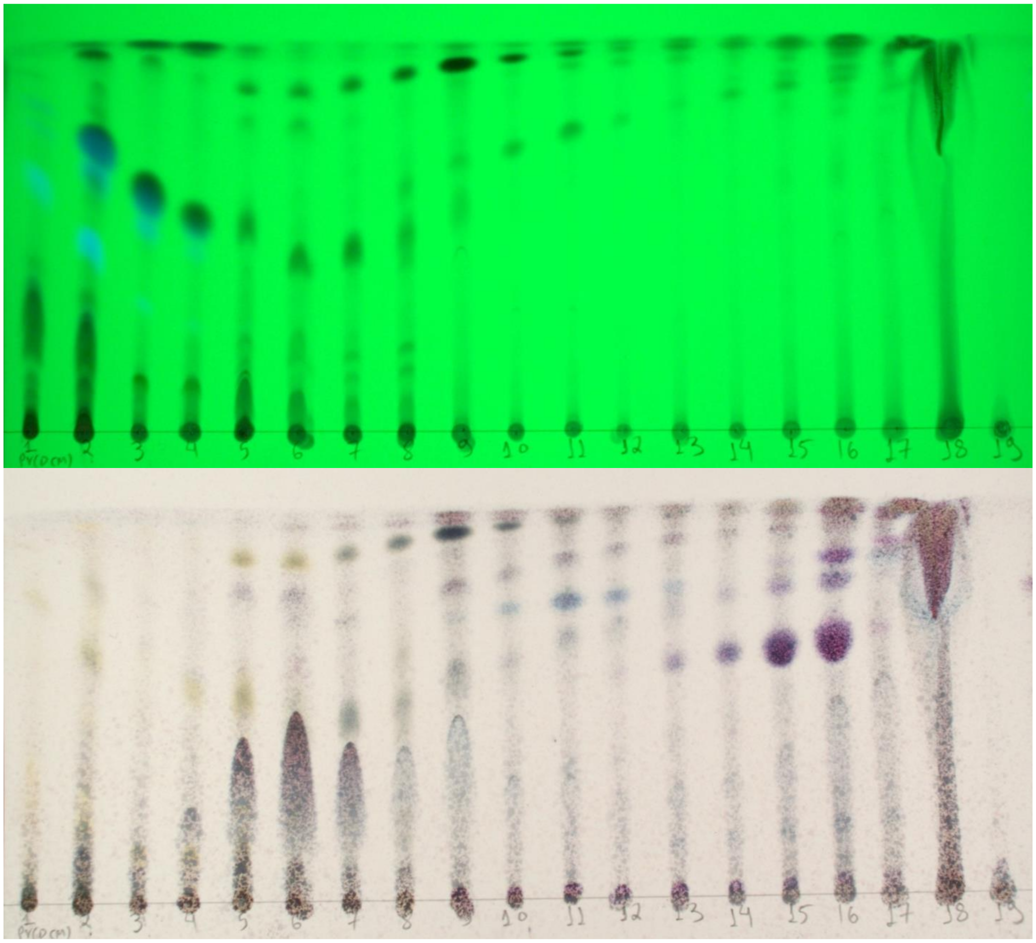

**Fig. S3**: TLC chromatograms of combined CPC fractions at 254nm (upper) and Vis (lower)

**Table S1**: The main fatty compounds of non-polar extract of *P. veris* subsp. *veris* identified by GC-MS analysis

| No | Rt | %of total | Name |
|---|---|---|---|
| 1 | 40.14 | 0.55 | Methyl hexadecanoate |
| 1 | 45.53 | 2.03 | Heneicosane |
| 2 | 47.38 | 2.75 | Nonadecane |
| 3 | 48.44 | 0.89 | Docosane |
| 4 | 51.31 | 41.67 | Tricosane |
| 5 | 52.94 | 4.85 | Tetracosane |
| 6 | 53.92 | 2.52 | Eicosane |
| 7 | 56.51 | 11.22 | Pentacosane |
| 8 | 56.79 | 2.23 | 2-nonadecanol |
| 9 | 58.07 | 1.71 | Hexacosane |
| 10 | 61.39 | 8.12 | Octadecane |
| 11 | 61.67 | 1.61 | 2-Pentacosanone |
| 12 | 65.69 | 10.98 | Pentacosane |

**Table S2**: The main fatty compounds of esterified non-polar extract of *P. veris* subsp. veris identified by GC-MS analysis

| No | Rt | % of total | Name |
|---|---|---|---|
| 1 | 25.93 | 6.16 | Methyl dodecanoate |
| 2 | 33.27 | 2.09 | Methyl tetradecanoate |
| 3 | 40.09 | 17.07 | Methyl hexadecanoate |
| 4 | 44.78 | 2.87 | (6Z,9Z,12Z,15Z)-Methyl octadeca-6,9,12,15-tetraenoate |
| 5 | 45.19 | 15.23 | Methyl linoleate |
| 6 | 45.37 | 15.04 | 9,12,15-Octadecatrienoic acid, methyl ester |
| 7 | 46.19 | 4.02 | Methyl octadecanoate |
| 8 | 47.26 | 1.05 | Docosane |
| 9 | 51.18 | 14.23 | Tricosane |
| 10 | 52.81 | 1.79 | Heneicosane |
|  | 53.79 | 0.98 | Tetracosane |
| 11 | 56.38 | 3.62 | Pentacosane |
| 12 | 56.65 | 1.21 | Tetratriacontyl trifluoroacetate |
| 13 | 57.08 | 0.49 | Docosanoic acid, methyl ester |

| | | | |
|---|---|---|---|
| 14 | 61.25 | 1.76 | Octacosane |
| 15 | 61.95 | 0.85 | Tetracosanoic acid, methyl ester |
| 16 | 65.81 | 1.57 | Nonadecane |

**Table S3**. Secondary metabolites identified in non-polar extract of *P. veris* subsp. *veris* by HPLC-HRMS analysis

| Nº | Rt (min) | Compound | | m/z | Suggested EC | RDB eq. | Delta (ppm) |
|---|---|---|---|---|---|---|---|
| **TERPENES** | | | | | | | |
| 1 | 6.23 | Loliolide | $[M+H]^+$ | 197.1168 | $C_{11}H_{17}O_3$ | 3.5 | 1.93 |
| 2 | 6.24 | Actinodiolide | $[M+H]^+$ | 179.1067 | $C_{11}H_{15}O_2$ | 4.5 | 0.36 |
| 3 | 9.15 | Dihydroactinodiolide | $[M+H]^+$ | 181.1222 | $C_{11}H_{17}O_2$ | 3.5 | -0.31 |
| 4 | 12.59 | 14,26-epoxyserratane-3,21-dione | $[M+H]^+$ | 455.3424 | $C_{30}H_{46}O_3$ | 7.5 | 1.05 |
| 5 | 17.84 | Stigmasterol | $[M+H]^+$ | 413.3779 | $C_{29}H_{49}O$ | 5.5 | 0.41 |
| **FLAVONE DERIVATIVES** | | | | | | | |
| 6 | 8.00 | Tetramethoxy-hydroxy-flavone (I) | $[M+H]^+$ | 359.1126 | $C_{19}H_{19}O_7$ | 10.5 | 0.14 |
| 7 | | Pentamethoxy-hydroxy-flavone (I) | $[M+H]^+$ | 389.1231 | $C_{20}H_{21}O_8$ | 10.5 | 0.12 |
| 8 | 8.12 | Trimethoxy-hydroxy-flavone | $[M+H]^+$ | 329.1020 | $C_{18}H_{17}O_6$ | 10.5 | 0.17 |
| 9 | | Tetramethoxy-hydroxy-flavone (II) | $[M+H]^+$ | 359.1126 | $C_{19}H_{19}O_7$ | 10.5 | 0.14 |
| 10 | | Pentamethoxy-hydroxy-flavone (II) | $[M+H]^+$ | 389.1231 | $C_{20}H_{21}O_8$ | 10.5 | 0.12 |
| 11 | 8.43 | Hydroxy-flavone | $[M+H]^+$ | 239.0702 | $C_{15}H_{11}O_3$ | 10.5 | -0.38 |
| 12 | | Methoxy-hydroxy-flavone | $[M+H]^+$ | 269.0809 | $C_{16}H_{13}O_4$ | 10.5 | 0.35 |
| 13 | | Dimethoxy-hydroxy-flavone (I) | $[M+H]^+$ | 299.0915 | $C_{17}H_{15}O_5$ | 10.5 | 0.2 |
| 14 | 8.63 | Dimethoxy-hydroxy-flavone (I) | $[M+H]^+$ | 299.0915 | $C_{17}H_{15}O_5$ | 10.5 | 0.2 |
| 15 | 9.08 | Trimethoxy-dihydroxy-flavone | $[M+H]^+$ | 345.0972 | $C_{17}H_{15}O_5$ | 10.5 | 0.84 |
| 16 | | Dimethoxy-dihydroxy-flavone | $[M+H]^+$ | 315.0865 | $C_{17}H_{15}O_6$ | 10.5 | 0.46 |
| 17 | | Pentamethoxy-flavone (I) | $[M+H]^+$ | 373.1284 | $C_{20}H_{21}O_7$ | 10.5 | 0.56 |
| 19 | | Pentamethoxy-dihydroxy-flavone | $[M+H]^+$ | 405.1183 | $C_{20}H_{21}O_9$ | 10.5 | 0.62 |

| | | | | | | | |
|---|---|---|---|---|---|---|---|
| 20 | | Hexamethoxy-dihydroxy-flavone (I) | $[M+H]^+$ | 435.1287 | $C_{21}H_{23}O_{10}$ | 10.5 | 0.20 |
| 21 | 9.24 | Pentamethoxy-flavone (II) | $[M+H]^+$ | 373.1284 | $C_{20}H_{21}O_7$ | 10.5 | 0.56 |
| 22 | | Hexamethoxy-flavone (I) | $[M+H]^+$ | 403.1389 | $C_{21}H_{23}O_8$ | 10.5 | 0.51 |
| 23 | | Hexamethoxy-dihydroxy-flavone (II) | $[M+H]^+$ | 435.1287 | $C_{21}H_{23}O_{10}$ | 10.5 | 0.20 |
| 24 | 9.42 | Dimethoxy-flavone | $[M+H]^+$ | 283.0966 | $C_{17}H_{15}O_4$ | 10.5 | 0.26 |
| 25 | | Tetramethoxy-flavone (I) | $[M+H]^+$ | 343.1177 | $C_{19}H_{19}O_6$ | 10.5 | 0.28 |
| 26 | | Pentamethoxy-flavone (III) | $[M+H]^+$ | 373.1284 | $C_{20}H_{21}O_7$ | 10.5 | 0.56 |
| 27 | | Hexamethoxy-flavone (II) | $[M+H]^+$ | 403.1389 | $C_{21}H_{23}O_8$ | 10.5 | 0.51 |
| 28 | 9.55 | Tetramethoxy-flavone (II) | $[M+H]^+$ | 343.1177 | $C_{19}H_{19}O_6$ | 10.5 | 0.28 |
| 29 | | Pentamethoxy-flavone (IV) | $[M+H]^+$ | 373.1284 | $C_{20}H_{21}O_7$ | 10.5 | 0.56 |
| 30 | | Hexamethoxy-flavone (III) | $[M+H]^+$ | 403.1389 | $C_{21}H_{23}O_8$ | 10.5 | 0.51 |
| 31 | 9.82 | Trimethoxy-flavone (I) | $[M+H]^+$ | 313.1068 | $C_{18}H_{17}O_5$ | 10.5 | -0.77 |
| 32 | 10.04 | Methoxy-dihydroxy-flavone | $[M+H]^+$ | 285.0757 | $C_{16}H_{33}O_5$ | 10.5 | -0.04 |
| 33 | | Trimethoxy-flavone (II) | $[M+H]^+$ | 313.1068 | $C_{18}H_{17}O_5$ | 10.5 | 0.51 |
| 34 | | Tetramethoxy-flavone (II) | $[M+H]^+$ | 343.1177 | $C_{19}H_{19}O_6$ | 10.5 | 0.28 |
| 35 | 10.21 | Flavone | $[M+H]^+$ | 223.0747 | $C_{15}H_{11}O_2$ | 10.5 | -2.76 |
| 36 | 10.45 | Methoxy-flavone | $[M+H]^+$ | 253.0859 | $C_{16}H_{13}O_3$ | 10.5 | 0.12 |
| **BIBENZYL DERIVATIVES** | | | | | | | |
| 37 | 10.50 | Bisbibenzyl derivative | $[M-H]^-$ | 441.1707 | $C_{28}H_{25}O_5$ | 16.5 | -0.06 |
| 38 | 10.76 | Riccardin C | $[M-H]^-$ | 423.1603 | $C_{28}H_{23}O_4$ | 17.5 | 0.37 |
| 39 | 11.22 | Bisbibenzyl derivative | $[M-H]^-$ | 425.1757 | $C_{28}H_{25}O_4$ | 16.5 | -0.34 |
| 40 | 11.39 | Bisbibenzyl derivative | $[M-H]^-$ | 455.1867 | $C_{29}H_{27}O_5$ | 16.5 | 0.75 |
| **TOCOPHEROLS AND TOCOENOLS** | | | | | | | |
| 41 | 11.92 | δ-Tocotrienol | $[M+H]^+$ | 397.3104 | $C_{27}H_{41}O_2$ | 7.5 | 0.89 |
| 42 | 12.67 | δ-Tocodienol | $[M+H]^+$ | 399.3261 | $C_{27}H_{43}O_2$ | 6.5 | 0.29 |
| 43 | 13.81 | α-Tocodienol | $[M+H]^+$ | 427.3571 | $C_{29}H_{47}O_2$ | 6.5 | 0.06 |
| 44 | 13.81 | (β or γ)-Tocodienol | $[M+H]^+$ | 413.3414 | $C_{28}H_{45}O_2$ | 6.5 | 0.02 |
| 45 | 14.35 | δ-Tocopherol | $[M+H]^+$ | 403.3546 | $C_{27}H_{47}O_2$ | 4.5 | -2.48 |
| 46 | 14.39 | α-Tocotrienol | $[M+H]^+$ | 425.3415 | $C_{29}H_{44}O_2$ | 7.5 | 0.27 |
| 47 | 14.86 | (β or γ)-Tocomonoenol | $[M+H]^+$ | 415.3568 | $C_{28}H_{47}O_2$ | 5.5 | -0.25 |
| 48 | 16.74 | (β or γ)-Tocopherol | $[M+H]^+$ | 417.3682 | $C_{28}H_{49}O_2$ | 4.5 | -4,47 |
| 49 | 16.84 | (β or γ)-Tocotrienol | $[M+H]^+$ | 411.3259 | $C_{28}H_{43}O_2$ | 7.5 | 0.35 |
| 50 | 16.84 | δ-Tocomonoenol | $[M+H]^+$ | 401.3421 | $C_{27}H_{45}O_2$ | 5.5 | -0.22 |
| 51 | 16.99 | α-Tocomonoenol | $[M+H]^+$ | 429.3703 | $C_{29}H_{49}O_2$ | 5.5 | -0.37 |
| 52 | 17.16 | (β or γ)-Tocotrienol | $[M+H]^+$ | 411.3261 | $C_{28}H_{43}O_2$ | 7.5 | 0.32 |

| | | | | | | | |
|---|---|---|---|---|---|---|---|
| 53 | 17.31 | (β or γ)-Tocopherol | $[M+H]^+$ | 417.3729 | $C_{28}H_{49}O_2$ | 4.5 | -0.18 |
| 54 | 17.37 | α-Tocopherol | $[M+H]^+$ | 431.3848 | $C_{29}H_{51}O_2$ | 4.5 | -3.53 |
| **PHENYLALKANE DERIVATIVES** | | | | | | | |
| 55 | 16.65 | 1-Phenyloctadecane-1,3-diyl-diacetate or 16-Methyl-phenyleptadecane-1,3-diyl-diacetate | $[M+H]^+$ | 447.3468 | $C_{28}H_{47}O_4$ | 5.5 | 0.06 |
| 56 | 17.21 | 1-Phenylhexadecane-1,3-diyl-diacetate or 14-Methyl-phenylpentadecane-1,3-diyl-diacetate | $[M+H]^+$ | 419.3159 | $C_{26}H_{43}O_4$ | 5.5 | 0.72 |
| 57 | 17.45 | 1-Phenyltetradecane-1,3-diyl-diacetate or 12-Methyl-phenyltriadecane-1,3-diyl-diacetate | $[M+H]^+$ | 391.2844 | $C_{24}H_{39}O_4$ | 5.5 | 0.19 |
| **OTHER COMPOUNDS** | | | | | | | |
| 58 | 7.38 | Vanillic acid | $[M-H]^-$ | 167.0351 | $C_8H_7O_4$ | 5.5 | 0.95 |

| TABLE S4: GENE/PROTEIN NAMES ASSOCIATED WITH BIOACTIVE PHYTOCHEMICALS | | |
|---|---|---|
| **NAME** | **DBID** | **SYNONYMS** |
| AKT1 | ENSP00000451828 | Akt, protein kinase B, Rac, Akt1, PKBalpha, v-akt murine thymoma viral oncogene homolog 1, PKB-alpha, protein kinase Balpha, AKT-1, protein kinase B-alpha, hAKT1, Akt 1, protein kinase B alpha, Pkb, proteinkinase B, protein kinase-B, Akt1 human, RA-C, RAC-alpha serine/threonine-protein kinase, v-akt murine-thymoma viral-oncogene-homolog 1, v-Akt murine thymoma viral oncogene homolog-1, AKT serine/threonine kinase 1, protein-kinase B, protein kinaseB, proteinkinaseB |
| MAPK3 | ENSP00000263025 | ERK 1, Erk1, MAPK3, extracellular signal-regulated kinase 1, p44 MAPK, extracellular signal-regulated kinase-1, p44mapk, p44erk1, ERK-1, extracellular signal regulated kinase-1, ERT2, Erk 1P, extracellular-signal regulated kinase 1, p44-MAPK, extracellular signal regulated kinase 1, ERK-1-P, mitogen-activated protein kinase 3, mitogen-activated protein kinase3, mitogen-activated protein kinase-3, extracellular signal-regulated-kinase 1 |
| TP53 | ENSP00000269305 | p53, TP53, tumor suppressor p53, Trp53, TP 53, tumor protein p53, tumor-suppressor p53, cellular tumor antigen p53, p53 human, TP53 p, p-53, p 53, U94788, phosphoprotein p53, p53-P, p53p, TP-53, T-p53, p53 p, tumorsuppressor p53, 2 BIM, tumor- suppressor p53, Hp53, p53- human, 2vuk, TP53-p, 2-bim, 1 PET, p53 - p |
| PTGS2 | ENSP00000356438 | COX-2, COX2, cyclooxygenase-2, PGHS-2, Cyclooxygenase 2, hCox-2, Ptgs2, prostaglandin endoperoxide synthase 2, Cox2p, prostaglandin-endoperoxide synthase 2, pHS2, cyclo-oxygenase 2, PGG/HS, prostaglandin-endoperoxide synthase-2, EC 1.14.99.1, cyclo-oxygenase-2, COX 2, PHS-2, cyclooxygenase -2, COX- 2, cyclooxygenase- 2, Prostaglandin G/H synthase 2, prostaglandin G/H synthase-2, cycloo-xygenase-2, cyclooxygenase2, prostaglandin H2 synthase 2, PTGS-2, Cyclo-oxygenase2, COX -2, prostaglandin-endoperoxide-synthase-2, prostaglandin-endoperoxide synthase 2 (prostaglandin G/H synthase and cyclooxygenase, Prostaglandin- endoperoxide synthase 2, prostaglandin-endoperoxide synthase 2 (prostaglandin G/H synthase and cyclo-oxygenase, prostaglandin endoperoxide synthase-2 |

| | | |
|---|---|---|
| IL6 | ENSP00000385675 | Il6, HGF, IL-6, interleukin 6, interleukin-6, IFN-beta2, IFNB2, BSF-2, IFN-beta 2, hybridoma growth factor, 1 Alu, interferon-beta-2, B cell stimulatory factor 2, interferon-beta 2, IFN beta 2, interferon beta 2, interferon beta2, B-cell stimulatory factor 2, hIL-6, H-IL-6, interleukin6, 1-IL-6, 1 IL-6, inter-leukin-6, hIL6, IL 6, Interleukin- 6, IL- 6, 2 IL-6, B cell stimulatory factor-2, BSF2, IL -6, Interleukin -6, interleukin - 6 |
| NFE2L2 | ENSP00000380252 | NRF-2, Nrf2, NF-E2-related factor 2, HEBP1, NFE2L2, nuclear factor erythroid 2-related factor-2, nuclear factor-erythroid 2-related factor 2, Nuclear factor,  erythroid-derived 2,  like 2, NF-E2 related factor-2, Nuclear factor erythroid-2 related factor 2, nuclear factor erythroid 2-related factor 2, NF-E2 related factor 2, NF-E2-related factor-2, NF-E2-related factor2, nuclear factor erythroid-2-related factor 2, Nuclear factor-erythroid 2 related factor 2, nuclear factor-erythroid 2-related factor-2, Nuclear factor erythroid-2-related factor-2, nuclear factor-erythroid-2-related factor 2, NFE2-related factor 2, NRF 2, nuclear factor,  erythroid derived 2,  like 2, nuclear factor-like 2, nuclear-factor-erythroid-2-related-factor-2, Nuclear factor erythroid-2 related factor-2, Nuclear factor,  erythroid 2-like 2, nuclear factor erythroid-2-related-factor-2, nuclear factor-erythroid-2-related factor-2, hNrf2, nuclear factor,  erythroid 2 like 2, nuclear factor erythroid 2 related factor 2, nuclear factor, erythroid 2 like-2, nuclear factor erythroid2-related factor 2, Nuclear factor erythroid 2-related factor2, nuclear factor-erythroid 2-related factor2, nuclear factor - erythroid - 2 - related factor 2, nuclear factor-erythroid 2 related factor-2, nuclear factor erythroid2-related factor2, Nuclear Factor,  Erythroid-2-Like-2, nuclear factor-erythroid 2 -related factor 2 |
| MMP9 | ENSP00000361405 | matrix metalloproteinase 9, MMP-9, gelatinase B, matrix metalloproteinase-9, 92-kDa type IV collagenase, MMP9, matrix-metalloproteinase-9, 92 kDa type IV collagenase, CLG4B, gelatinase-B, 92-kDa gelatinase, 92 kDa gelatinase, EC 3.4.24.35, MMP 9, matrix-metalloproteinase 9, matrixmetalloproteinase-9, matrix metallopeptidase 9, matrix metallopro-teinase-9, hMMP9, matrix metallopeptidase-9, 92kDa type IV collagenase, matrixmetallo-proteinase-9, matrix metalloproteinase-9, matrix metallo proteinase 9, Matrix metalloproteinase -9, matrix metalloproteinase- 9, matrix metalloproteinase9, Matrix metallopeptidase9, MMP- 9, 1L6J, MMP -9 |

| | | |
|---|---|---|
| EGFR | ENSP00000275493 | ErbB, epidermal growth factor receptor, EGFR, ErbB1, EGF-R, ErbB-1, epidermal growth factor-receptor, HER1, ErbB 1, ErbB2 receptor tyrosine kinases, Erb B, Erb B-1, epidermal-growth-factor receptor, epidermal-growth-factor-receptor, epidermal growth-factor receptor, EG FR, EGF R, erb-B, epidermalgrowth factor receptor, HER-1, men--a, proto-oncogene c-erbB1, erb-b1, proto-oncogene c-erb-B1, epi- dermal growth factor receptor, hErbB-1, men-a, MENA, epidermal growth factor receptor, epidermal growth factor receptor, epidermal growth factor receptor, epidermal growth-factor-receptor, EGFR-202, Men - A, epidermal-growth factor receptor, EGFR Human, HER 1, 2 ITP, proto-oncogene c-erbB-1 |
| TNF | ENSP00000398698 | TNF, TNF-alpha, TNFA, TNFalpha, TNF alpha, cachectin, tumor-necrosis factor superfamily, tumor-necrosis-factor, tumor necrosis factor superfamily, tumor necrosis-factor, Tumor-necrosis factor, 1 TNF, Tumor Necrosis Factor, TNF-a, TNF- alpha, TNF A, TNFSF2, tumor necrosis factor super-family, TNF superfamily, member 2, tumor necrosis factor-superfamily, Dif, TNF -A, tumor necrosis factor super family |
| CDKN1A | ENSP00000384849 | p21, Cip1, Waf1, p21CIP1/WAF1, Cdkn1a, p21CIP1, cyclin-dependent kinase inhibitor 1A, CDKN1, PIC1, p 21, Sdi1, Hp 2-1, Hp2-1, p21 Cip1, WAF-1, CIP-1, cyclin-dependent kinase inhibitor 1, PIC-1, p-21, p21Cip1/WAF-1, p2 1, Cyclin-dependent kinase-inhibitor 1, Cap20, Mda-6, Sdi-1, MDA6, p21CIP-1, CIP 1, p21-p, p21 Cip1/Waf1, Cyclin-dependent kinase inhibitor-1A, p21CIP-1/WAF-1, p21-Cip1, CDK-interacting protein 1, Cyclin-Dependent Kinase Inhibitor-1, cyclin dependent kinase inhibitor 1A, P2-1, cyclin dependent kinase inhibitor 1, HP21 |
| JUN | ENSP00000360266 | c-Jun, AP-1, Jun, HAP-1, transcription factor AP-1, activator protein 1, HAP1, p39, jun oncogene, Cjun, jun proto-oncogene, proto-oncogene c-Jun, JUN protooncogene, Ap1, transcription factor AP1, activator protein1, Activator protein-1, activator-protein 1, c- jun, v-JUN avian sarcoma virus 17 oncogene homolog, Activator-Protein-1, c Jun, Jun proto-oncogene, AP-1 transcription factor subunit, protooncogene c-jun |

| | | |
|---|---|---|
| ESR1 | ENSP00000405330 | estrogen receptor, estrogen receptor alpha, 2-p15, 2p15, ERalpha, ER-alpha, ER alpha, estrogen receptor-alpha, 4-q13, ESR1, estrogen-receptor, 5 due, estrogen receptor 1, oestrogen receptor alpha, ERa, oestrogen receptor-alpha, oestrogen-receptor-alpha, estradiol receptor, 4q 13, 5 TMS, NR3A1, Esr, 2----p15, ESR 1, ER-a, estrogen-receptor-alpha, 5 T MR, estrogen-receptor alpha, hesr1, estrogen receptor-1, estrogen receptor-a, 5-E15, estrogen receptor- 1, estrogen receptor -alpha, ESR-1, oestrogen receptoralpha, estrogen receptoralpha, estrogen receptor1, estrogen receptor- alpha, E2 receptor-alpha, estrogen receptor, estrogen receptor, ESR1P, 5 ACC, AL356311, estrogen receptora, ESR1-008, ESR1-203, 4-PPs, 4 mgC, 4mgC, 5TMS, 5-e19 |
| MAPK1 | ENSP00000215832 | p38, p42 MAPK, ERK2, p41mapk, mitogen-activated protein kinase 1, MAPK1, p41, extracellular signal-regulated kinase 2, p42mapk, MAPK 1, erk, mitogen activated protein kinase 1, MAPK2, extracellular signal-regulated kinase-2, ERK-2, 5 NHL, extracellular signal regulated kinase 2, p38-P, p-38, p42-MAPK, Extracellular signal regulated kinase-2, extracellular-signal-regulated kinase2, ERK-2-P, Erk 2, mitogen-activated protein kinase-1, extracellular-signal-regulated kinase 2, extracellular signal regulated-kinase-2, mitogen-activated-protein-kinase 1, MAPK1 p, extracellular signal-regulated kinase2, Mitogen-Activated Protein Kinase 2, MAPK-1, 3W 55, HP40, ER K, mitogen- activated protein kinase 1, mitogen activated protein kinase1 |
| GABPA | ENSP00000346886 | NRF-2, Nrf2, GABPA, transcription factor E4TF1-60, E4TF1-60, nuclear respiratory factor 2 alpha subunit, GA-binding protein transcription factor, alpha subunit, D13318, NRF 2, hNrf2, Nrf2-a, nrf2a |
| MAPK14 | ENSP00000229795 | p38, p38 alpha, p38alpha, p38 MAP kinase, 3 new, Mapk14, Mxi2, CSBP2, CSBP, SAPK2a, CSBP1, p38-MAP kinase, p38 MAP-kinase, p38-P, mitogen-activated protein kinase 14, p-38, 3 mGy, p38MAP kinase, p38 MAPKinase, p38MAP-kinase, 3 MPA, 3 hub, 3 FLs, Mxi-2 |

| | | |
|---|---|---|
| STAT3 | ENSP00000264657 | STAT3, signal transducer and activator of transcription 3, Stat-3, Acute-phase response factor, APRF, signal transducer and activator of transcription-3, acute phase response factor, STAT 3, STAT3 P, Stat 3P, signal-transducer-and-activator-of-transcription-3, signal transducer and activator of transcription3, STAT3-P, signal-transducer and activator of transcription 3, hSTAT3, signal transducer and activator of transcription 3, signal transducer and activator of transcription 3, signal transducer and activator of transcription 3 (acute-phase response factor |
| EGF | ENSP00000265171 | EGF, epidermal growth factor, epidermal-growth-factor, epidermal growth-factor, pro-epidermal growth factor, epidermal-growth factor, Epidermal growth factor, epidermalgrowth factor, Epidermal growthfactor |
| AR | ENSP00000363822 | HUMARA, AR, androgen receptor, dihydrotestosterone receptor, androgen-receptor, Tfm, 1E3-G, Dihydrotestosterone-receptor, A-Is, NR3C4, AR8, SMAX1, ar202, Androgenreceptor, AI s, HU-MARA |
| AHSA1 | ENSP00000216479 | p38, Aha1, p38-P, p-38, Ahsa1, Activator of 90kDa heat shock protein ATPase homolog 1 |
| CRK | ENSP00000300574 | p38, CrkII, Crk, Crk-II, protooncogene C-CRK, p38-P, p-38 |
| GRAP2 | ENSP00000339186 | p38, growth factor receptor-binding protein, GADS, grap-2, Grap2, Grb-2 related adaptor protein 2, p38-P, p-38, growth factor receptor binding protein |
| RNF19A | ENSP00000428968 | p38, Dorfin, double ring-finger protein, p38-P, double RING finger protein, p-38 |
| POLDIP2 | ENSP00000475924 | p38, HSPC017, PDIP38, p38-P, p-38, POLDIP2, POLD4, Polymerase delta-interacting protein 2, Polymerase Delta Interacting Protein 2 |

| | | |
|---|---|---|
| IGF1 | ENSP00000302665 | IGF-I, IGF-1, igf1, insulin-like growth factor I, insulin-like growth factor-I, insulin-like growth factor 1, MGF, somatomedin-C, insulin-like growth factor-1, IBP-1, IGFI, somatomedin C, IGF I, insulin like growth factor 1, insulin like growth factor-1, insulin-like-growth-factor I, insulinlike growth factor-I, IGF 1, insulin-like growth factor - 1, insulin-like growth-factor-I, 1GF-1, IGF- 1, mechano growth factor, insulin-like-growth factor-1, hIGF-1, Insulin like-growth factor I, mechano-growth factor, insulin-like growth factor1, insulinlike growth factor 1, Insulinlike growth factor-1, insulin like growth factor I, insulin-like-growth factor 1, Insulin-like-Growth Factor-I, IGF-1 A, Insulin like growth factor-I, insulin-like growth-factor I, insulin like growth factor -1, insulin like growth factor- I, hIGF1, Insulin like-growth factor-1, IGF - 1 |
| FOS | ENSP00000306245 | AP-1, Fos, c-fos, proto-oncogene c-fos, p55, c- fos, G0S7, Ap1, protooncogene c-fos, cFOS, proto-oncogene cfos, p 55, v-fos FBJ murine osteosarcoma viral oncogene homolog, FBJ murine osteosarcoma viral oncogene homolog, cellular oncogene fos, HP55, cF-OS, Fos proto-oncogene, AP-1 transcription factor subunit |
| AIMP2 | ENSP00000223029 | p38, AIMP2, aminoacyl tRNA synthetase complex interacting multifunctional protein 2, p38-P, p-38, JTV-1, aminoacyl tRNA synthetase complex-interacting multifunctional protein 2, JTV1, aminoacyl-tRNA synthetase complex-interacting multifunctional protein 2 |
| INS | ENSP00000380432 | insulin, INS, IDDM1, ILPR, 2 WS1, 5-EMs, 3 in c, 2 CEU, 1 hit, 1-His, 3W 12, IRDN, 1hi T, 1 Efe |
| MMP2 | ENSP00000219070 | MMP-2, MMP2, gelatinase A, 72-kDa type IV collagenase, matrix metalloproteinase-2, matrix metalloproteinase 2, TBE1, gelatinase-A, CLG4, 72 kDa gelatinase, 72-kDa gelatinase, matrix metalloproteinase -2, matrix-metalloproteinase-2, 72 kda type IV collagenase, 72-kDa type-IV collagenase, MMP 2, MMP-II, matrix metallopeptidase-2, matrix metallopeptidase 2, matrix metalloproteinase-2, MMP- 2, matrix metalloproteinase2, matrix metallopeptidase -2, matrix-metalloproteinase 2, matrix metalloproteinase- 2, matrix metallo peptidase 2, TBE-1, EC 3.4.24.24 |

| | | |
|---|---|---|
| CASP3 | ENSP00000311032 | SCA1, caspase-3, SCA 1, Sca-1, CASP3, caspase 3, 2 DKO, CPP32, apopain, YAMA, caspase -3, casp-3, caspase3, caspase- 3, cysteine protease CPP32, 1 nM E, CPP-32, Casp 3, caspase 3, 2-DKO, caspase 3, apoptosis-related cysteine protease |
| TTPA | ENSP00000260116 | alpha-tocopherol transfer protein, TPP1, ATTP, TTPA, alpha-tocopherol-transfer protein, alpha TTP, alphaTTP, alpha-TTP, alpha -tocopherol transfer protein, TPP-1, TTP1, alpha tocopherol transfer protein, TTP-1, tocopherol transfer protein, 5 mu g, Ataxia with vitamin e deficiency, tocopherol-transfer protein, a-tocopherol transfer protein |
| BDNF | ENSP00000414303 | BDNF, brain-derived neurotrophic factor, brain derived neurotrophic factor, neurotrophin, Brain-Derived-Neurotrophic-Factor, brain- derived neurotrophic factor, brain-derived neurotrophic-factor, brain-derived-neurotrophic factor, brainderived neurotrophic factor, Brain-derived neuro-trophic factor, BDNF human, brain-derived neurotrophic factor, Brai nderived Neurotrophic Factor |
| JUND | ENSP00000252818 | AP-1, JUND, activator protein 1, jun D, Ap1, jun-D, activator protein1, transcription factor JunD, Activator protein-1, activator-protein 1, jun d proto-oncogene, Activator-Protein-1, JunD Proto-Oncogene |
| NSG1 | ENSP00000388823 | p21, Hp2-1, D4S234, p 21, Hp 2-1, p-21, p2 1, p21-p, P2-1, NEEP21, HP21 |
| TCEAL1 | ENSP00000361708 | TCEAL1, p21, Hp2-1, SIIR, pp21, Hp 2-1, p 21, p-21, p2 1, p21-p, p-p21, P2-1, HP21 |

| | | |
|---|---|---|
| ERBB2 | ENSP00000269571 | HER2/neu, HER-2/neu, HER-2/ neu, erbB2, HER2, ErbB-2, p185erbB2, Her-2, human epidermal growth factor receptor 2, human epidermal growth factor receptor-2, Her 2, Erb-B2, v-erb-b2 avian erythroblastic leukemia viral oncogene homolog 2, Erb B-2, p185erbB-2, cd 340, HER2/ neu, HER2 / neu, proto-oncogene c-erbB-2, HER 2/neu, erb-B-2, HER-2 /neu, proto-oncogene c-erbB2, erb B2, HER-2 p, HER -2/ neu, hHER-2, Her - 2 / neu, human epidermal growth factor receptor2, p 185ERBB2, protooncogene c-erbB-2, neuro/glioblastoma derived oncogene homolog, human epidermal growth factor receptor 2, human epidermal growth-factor receptor-2, human epidermal growth factor receptor 2, tyrosine kinase-type cell surface receptor HER2, HER2-p, erb-b2 receptor tyrosine kinase 2, V-Erb-B2 avian erythroblastic leukemia viral oncogene homolog-2, Human epidermal growth factor receptor - 2, ErbB2 Receptor Tyrosine Kinase 2, p185-ERBB2, receptor tyrosine-protein kinase erbB-2, human epidermal growth factor-receptor 2, HumanEpidermal Growth Factor Receptor 2, hHER2, ErbB2 human, ErbB 2, H uman Epidermal Growth Factor Receptor 2, human-epidermal-growth-factor-receptor 2, 3RCD, CD340, human epidermal growth-factor receptor 2, ERBB-2 - p |
| HIF1A | ENSP00000437955 | hypoxia-inducible factor 1alpha, HIF-1alpha, HIF-1, Hif1a, HIF-1 alpha, Hif1 alpha, HIF1alpha, hypoxia inducible factor 1alpha, hypoxia-inducible factor-1alpha, hypoxia-inducible factor-1 alpha, MOP1, HIF1, hypoxiainducible factor-1alpha, hypoxia-inducible factor 1 alpha, hypoxia inducible factor-1alpha, hypoxia-inducible factor 1, alpha subunit, Hypoxia inducible factor 1 alpha, hypoxia inducible factor-1 alpha, HIF1-alpha, HIF-1a, hypoxia-inducible-factor-1 alpha, hypoxia-inducible-factor 1alpha, HIF- 1 alpha, hypoxia-inducible factor 1-alpha, HIF-1-alpha, Hypoxia inducible factor 1-alpha, hypoxia-inducible factor-1-alpha, HIF 1alpha, HIF 1-alpha, hypoxia inducible factor 1 alpha subunit, hypoxia-inducible factor -1alpha, Hypoxia inducible-factor1alpha, Hypoxia-inducible factor 1 alpha subunit, 1LM8, HIF 1 alpha, hypoxia inducible factor-1-alpha, hypoxia inducible factor1 alpha, hypoxia-inducible-factor 1-alpha, Hypoxia Inducible Factor -1 alpha, hypoxia-inducible factor1-alpha, hypoxia-inducible-factor-1alpha, HIF1-a, hypoxia-inducible factor-1 alpha subunit, Hypoxia-inducible factor -1 alpha |

| | | |
|---|---|---|
| HRAS | ENSP00000407586 | Ha-ras, K-ras, p21 Ras, N-Ras, NRAS, Kras2, H-Ras, KRAS, HRAS1, HRAS, Harvey rat sarcoma viral oncogene homolog, Kras-2, p21RAS, c-Ha-ras-1, p21-ras, c-Ha-ras1, c-Ha-ras 1, v-Ha-ras Harvey rat sarcoma viral oncogene homolog, c-Ha ras-1, c-Ha- ras1, 5p21, c-H-ras, 1 AGP, Hras-1, 1AGP, H-ras-1, 1 q21, K-ras-2, 2 q21, Ha ras, 1-AGP, 1q 21, H-ras 1, K ras, H-ras1, K- ras, k- ras -2, 2Rgc, H ras, HaRas, K-ras2, K -ras, H- ras, N- RAS, GTPase HRas, 1 q 21, 2 ClC, H-p21Ras, HRas proto-oncogene, GTPase, CTlo, H -ras, K-ras 2, c-bas/has, 1 PLL, cHa-Ras-1 |
| LEP | ENSP00000312652 | leptin, LEP, ObS, obese protein |
| JUNB | ENSP00000303315 | AP-1, JunB, jun B, Ap1, jun-B, transcription factor JunB, junB proto-oncogene, junB protooncogene, transcription factor Jun B, transcription factor jun-B |
| CXCL8 | ENSP00000306512 | IL-8, interleukin-8, interleukin 8, NAP-1, CXCL8, IL8, hNap1, TSG-1, Monocyte-derived neutrophil chemotactic factor, Neutrophil-activating peptide-1, NAP1, MDNCF, MONAP, T cell chemotactic factor, GCP-1, chemokine ligand 8, TSG1, CXCL-8, interleukin- 8, NAP 1, interleukin8, LUCT, IL 8, chemokine ligand-8, IL- 8, chemokine (C-X-C motif) ligand 8, C-X-C motif chemokine ligand 8, interleukin - 8, interleukin -8, hIL-8, C-X-C motif chemokine 8, 3-10C |
| CYP1A1 | ENSP00000369050 | cytochrome P-4501A1, AhRR, CYP1A1, hCyP1, cytochrome P-450C, P1-450, CYPIA1, Cyp1a-1, CYP1, cytochrome P450 1A1, hCYP1A1, cytochrome P450 family 1, EC 1.14.14.1, Cyp 1a1, cytochrome P-450 1A1, cytochrome P4501A1, CYP I A1, CYP-1A1, cytochrome-P450 1A1, cytochrome P450-1A1, Cyp 1a-1, Cytochrome P450, family 1, subfamily A, polypeptide 1, cytochrome P450c, cytochrome P 450 1A1, Cytochrome P 4501A1, CYP1 A1, cytochrome P450 family 1 subfamily A member 1, A-HH |
| CCND1 | ENSP00000227507 | Ccnd1, cyclin D1, PRAD1, bcl-1, D11S287E, cyclinD1, BCL1, BCL1 oncogene, U21B31, PRAD 1, PRAD-1, bcl -1, cyclin-D1, PRAD1 oncogene, BCL-1 oncogene, Bcl 1, CCN D1, cy-clin D1, Ccnd-1, Cyclin D-1, Cyclin D 1, CCND 1, CyclinD-1, cyclinD 1, B-CL1, PRAD-1 oncogene |
| FOSB | ENSP00000245919 | AP-1, activator protein 1, FosB, Ap1, activator protein1, Activator protein-1, activator-protein 1, fos B, fos-B, GOS-3, FBJ murine osteosarcoma viral oncogene homolog B, GOS3, AP- 1, oncogene Fosb, Activator-Protein-1, FosB Proto-oncogene, AP-1 Transcription Factor Subunit |

| | | |
|---|---|---|
| HGF | ENSP00000222390 | HGF, Hepatocyte growth factor, scatter factor, lung fibroblast-derived mitogen, Hepatocyte growth-factor, F-TCF, hepatocyte-growth-factor, hepatocyte-growth factor |
| HMOX1 | ENSP00000216117 | HO-1, Heme oxygenase-1, Hsp32, Hmox1, heme oxygenase 1, heme-oxygenase-1, HO1, HSP-32, heme oxygenase- 1, hHO-1, hemeoxygenase-1, HMOX-1, heme oxygenase1, heme-oxygenase 1, hemeoxygenase 1, HO- 1, heme oxygenase (decycling) 1 |
| CFTR | ENSP00000003084 | cystic fibrosis transmembrane conductance regulator, CFTR, MRP7, ABCC7, cAMP-dependent chloride channel, cystic fibrosis transmembrane-conductance regulator, cystic fibrosis trans-membrane conductance regulator, CTFR, cystic-fibrosis transmembrane-conductance regulator, cystic-fibrosis transmembrane conductance regulator, cystic fibrosis trans membrane conductance regulator, cAMP dependent chloride channel, cystic fibrosis transmembrane conductance regulator (ATP-binding cassette subfamily C, member 7, ATP binding cassette sub-family C, cystic fibrosis transmembrane conductance regulator (ATP-binding cassette sub-family C, member 7, ATP-binding cassette subfamily C, member 7, cystic fibrosis transmembrane conductance regulator, CFTR human, cystic fibrosis-transmembrane conductance regulator, ATP-binding cassette sub-family C, CFTR/MRP, cystic fibrosis transmembrane conductance-regulator, ABC-C7, ATP binding cassette subfamily C, cystic-fibrosis-transmembrane-conductance-regulator |
| MT-CO2 | ENSP00000354876 | COX II, Cox-2, COII, Cox2, CO II, cytochrome C oxidase subunit II, COXII, Cox2p, MT-CO2, cytochrome C oxidase II, hCOX-2, COX 2, MTCO2, COX-II, COX- 2, cytochrome c oxidase subunit 2, COX -2, cytochrome c oxidase polypeptide II, CoI I |
| SIRT1 | ENSP00000212015 | SIR2, sirtuin, Sir2alpha, Sir2p, SIRT1, sirtuins, hSIR2, sirtuin 1, sirtuin-1, SIRTUIN1, hSIRT1, Sir2-like protein 1, Sirt-1, SIRT 1, Sir 2, NAD-dependent protein deacetylase sirtuin-1, NAD-dependent protein deacetylase sirtuin 1 |

| | | |
|---|---|---|
| RAF1 | ENSP00000251849 | Raf-1, Raf, RAF1, c-Raf, CRAF, RAF family, Raf- 1, v-raf-1 murine leukemia viral oncogene homolog 1, RNase3, RNase 3, C- Raf, v-raf-1 murine leukemia viral oncogene homolog-1, RAF proto-oncogene serine/threonine-protein kinase, proto-oncogene C-Raf, Raf-1 proto-oncogene, serine/threonine kinase, C-Raf proto-oncogene, serine/threonine kinase, C RAF, RAF protooncogene serine/threonine-protein kinase |
| MTOR | ENSP00000354558 | RAFT1, mammalian Target of Rapamycin, mTOR, mTOR complex 1, mechanistic target of rapamycin, rapamycin and FKBP12 target 1, FRAP1, mTOR complex 2, 1 FAP, 2 FAP, FRAP2, mammalian-target-of-rapamycin, FKBP-rapamycin-associated protein, mammalian-target of rapamycin, mammalian target-of-rapamycin, mTOR complex1, mammalian target of rapamycin, FK506 binding protein 12-rapamycin associated protein 1, FKBP12-rapamycin complex-associated protein 1, mTor-complex-2, mTOR complex-1, mTOR complex-2, mechanistic target of rapamycin (serine/threonine kinase, mechanistic target of rapamycin kinase, serine/threonine protein kinase mTOR, m-TOR, 4 FAP, mTOR complex2, 2FAP, mammalian-target-of rapamycin, mechanistic target-of-rapamycin, serine/threonine-protein kinase mTOR, 3 FAP |
| FGF2 | ENSP00000264498 | fibroblast growth factor-2, basic fibroblast growth factor, FGF-2, bFGF, fibroblast growth factor 2, FGF2, HBGF-2, FGFB, fibroblast growth factor 2 (basic, fibroblast-growth factor 2, hFGF-2, b-FGF, hFGF2, basic fibroblast-growth factor, basic-fibroblast growth factor, 1 C vs, fibroblast growth factor2, basic fibroblast growth factor, FGF-b, 2 FGF, b FGF, FGF 2, fibroblast-growth-factor-2 |
| KRAS | ENSP00000256078 | Ki-ras, K-Ras, Kras2, KRAS, Kras 4A, c-Ki-ras2, c-Ki-ras, c-K-ras, KRAS4A, K ras, KRAS4B, K-ras-2, c-Ki- ras2, K-Ras4A, Kras-2, K-ras-1, c-Ki-ras-2, K- ras, K-Ras4B, k- ras -2, cKRAS, Ki- ras, K-ras2, Kirsten rat sarcoma viral oncogene homolog, NS3P, K -ras, v-Ki-ras2 Kirsten rat sarcoma viral oncogene homolog, c-Ki-ras 2, K-ras 4A, K-ras 4B, KRAS-1, KRAS-4A, GTPase K-ras, HNS3, HNS-3, GTPase KRas, KRAS proto-oncogene, GTPase, HCFC2, Kirsten-rat sarcoma viral oncogene homolog, KRAS 4B, KRAS-4B, K-ras 2, v-Ki-ras-2 Kirsten rat sarcoma viral oncogene homolog, Kiras |

| | | |
|---|---|---|
| SRC | ENSP00000362680 | Src, Src family tyrosine kinases, c-Src, SRC-1, pp60c-src, p60Src, proto-oncogene c-src, cSrc, SRC1, protooncogene c-src, Src-family tyrosine kinases, 2 Src, 1a1b, p60-SRC, 1A1--a, proto-oncogene tyrosine-protein kinase Src, v-src avian sarcoma viral oncogene homolog, 1 HCs, Src proto-oncogene, non-receptor tyrosine kinase, SRC proto-oncogene, nonreceptor tyrosine kinase |
| ESR2 | ENSP00000343925 | ERbeta, estrogen receptor beta, ESR2, ER beta, estrogen receptor 2, estrogen receptor-beta, oestrogen receptor beta, ESR beta, erb, ER-beta, oestrogen receptor-beta, ER-B, hesr2, ESR-beta, estrogen receptor b, ESR-b, Estrogen-receptor beta |
| APOE | ENSP00000252486 | Apolipoprotein E, apo E, APOE, APOE4, APOE-4, AD2, apolipoprotein-E, apo-E, apo E4, apolipo-protein E, apo E-4, APOE 4, 1 or -2, Apo-E4, apo E 4, apolipoproteinE, 1 or -3, h-apoE4, AF261279, Apo lipoprotein E, hAPOE4, APOE-005, APOE-001, AD-2, Ad 2, ApoE human |
| CCL2 | ENSP00000225831 | MCP-1, monocyte chemoattractant protein-1, CCL2, MCAF, monocyte chemotactic protein-1, monocyte chemoattractant protein 1, Scya2, Mcp1, small inducible cytokine A2, monocyte chemotactic protein 1, chemokine ligand 2, GDCF-2, Ccl-2, chemokine (C-C motif) ligand 2, monocyte chemotactic and activating factor, monocyte-chemotactic protein-1, Monocyte-chemo-attractant-protein-1, monocyte-chemo-attractant protein-1, hMCP-1, monocyte-chemoattractant protein-1, MCP 1, monocytechemoattractant protein 1, chemokine (C-C motif) ligand-2, 1 mL 0, cc l2, monocyte-chemoattractant-protein-1, MCP- 1, Monocyte-chemotactic-protein-1, chemokine (CC motif) ligand-2, C-C motif chemokine ligand 2, C-C motif chemokine 2, chemokine ligand-2, SMC-CF, monocyte chemotactic protein-1, monocyte chemo-attractant protein-1, monocyte chemoattractant protein -1, monocyte chemotactic protein -1, monocyte chemo-attractant protein 1, chemokine (CC motif) ligand 2, Monocyte Chemoattractant Protein1, CC-motif Chemokine Ligand 2, chemokine-ligand-2, CC motif chemokine ligand 2, monocyte chemoattractantprotein-1, chemokine (C-C- motif) ligand 2, Monocyte-chemoattractant protein 1 |

| | | |
|---|---|---|
| MAP2K1 | ENSP00000302486 | Mek1, MAP2K1, MKK1, mitogen-activated protein kinase kinase-1, MEK 1, MEK-1, mitogen-activated protein kinase kinase 1, PRKMK1, MAPK/ERK kinase 1, 3 MBL, MAPKK-1, mitogen activated protein kinase kinase-1, mitogen activated protein kinase kinase 1, MKK-1, dual specificity mitogen-activated protein kinase kinase 1, dual specificity mitogen activated protein kinase kinase 1, 3 or n, MAP2K1 p |
| PTEN | ENSP00000361021 | PTEN, TEP1, MMAC1, PTEN1, mutated in multiple advanced cancers 1, TEP-1, hTEP1, PTEN human, GLM-2, mutated in multiple advanced cancers-1, mHam, P-TEN |
| MMP1 | ENSP00000322788 | Matrix metalloproteinase-1, CLG, MMP1, fibroblast collagenase, MMP-1, matrix metalloproteinase 1, interstitial collagenase, matrix-metalloproteinase-1, hMMP1, EC 3.4.24.7, MMP 1, CLGN, matrix metallopeptidase 1, matrix metallo-proteinase-1, matrix metalloproteinase1, Matrix Metallopeptidase-1 |
| EGR1 | ENSP00000239938 | Egr-1, Egr1, Zif268, Krox24, NGFI-A, Krox-24, early growth response-1, tis8, early-growth response 1, G0S30, NGFIA, early growth response protein 1, Krox 24, transcription factor Zif268, early growth response 1, early growth response protein-1, early-growth response-1, early-growth-response 1, nerve growth factor-induced protein A, EGR 1, Early Growth Response- 1, zif-268 |
| PLAU | ENSP00000361850 | urokinase-type plasminogen activator, u-PA, uPA, PLAU, Atf, urokinase type plasminogen activator, urokinase- type plasminogen activator, urokinase type plasminogen-activator, urokinase-type plasminogen-activator, urokinase type-plasminogen activator, plasminogen activator,  urokinase, urokinasetype plasminogen activator, Plau-001, Plau-004, 4 Gly, up-a, Urokinase-type-plasminogen-activator |
| IL1B | ENSP00000263341 | IL-1, IL-1beta, interleukin-1beta, IL-1 beta, interleukin-1B, interleukin 1beta, Il1b, IL1-beta, interleukin-1 beta, IL1, interleukin 1B, IL1beta, interleukin 1 beta, Il-1b, interleukin-1-beta, IL 1 beta, interleukin- 1beta, interleukin 1-beta, interleukin 1- beta, IL1 beta, IL-1-B, IL1 B, IL-1-beta, IL1-B, IL- 1beta, Interleukin1beta, interleukin-1 b, inter-leukin-1 beta, interleukin- 1 beta, IL-1 b, IL 1beta, interleukin 1-B, IL 1, Interleukin 1,  beta, IL -1, IL- 1 beta, IL-1 -beta |

| | | |
|---|---|---|
| N | ENSP00000479618 | c-Myc, myc, protooncogene c-myc, proto-oncogene c-myc, CMYC, MYCC, 2 or 9, MYC-C, proto-oncogene c- myc, c- myc, myc human, c -MYC, mrtl, Myc C, v-myc avian myelocytomatosis viral oncogene homolog, myc proto-oncogene protein, c Myc, MYC proto-oncogene, bHLH transcription factor, CMY-C |
| BRCA1 | ENSP00000418960 | BRCA1, breast cancer 1, breast cancer-1, BRCAI, BRCA 1, breast cancer type 1 susceptibility protein, BRCA1 p, U14680, FANCS, BRCA-1, breast cancer 1, early onset, Breast-Cancer-1, hBRCA1, BRCA1 human, Breast CAncer1, 1T15, BRCA1, DNA repair-associated, BRCA1, DNA repair associated |
| MCL1 | ENSP00000358022 | Mcl-1, MCL-1L, bcl-2 family, MCL1, MCL-1S, Bcl2-family, Bcl2 family, E-AT, myeloid cell leukemia-1, myeloid cell leukemia sequence-1, myeloid cell leukemia sequence 1, Bcl-2-family, mcl-1/EAT, myeloid cell leukemia 1, myeloid-cell leukemia sequence 1, MCL1s, Mcl1L, HGNC: 6943, Mcl1-S, MCL1-L, 4-bp I, Bcl 2-family, 5 LoF, hMcl-1, 2MHS, induced myeloid leukemia cell differentiation protein Mcl-1, Mcl-1ES, Mcl -1, mcl1/EAT |
| CDH1 | ENSP00000261769 | CDH1, E-cadherin, Ecad, uvomorulin, epithelial cadherin, L-CAM, hCDH1, Arc1, E-cad, cadherin-1, cd 324, cadherin 1, Ecadherin, CDH-1, E cadherin, E- cadherin, CAM 120/80, epithelial-cadherin, Arc-1, cadherin 1, type 1, E-cadherin, cadherin1, CD324 |
| JAK2 | ENSP00000371067 | Jak2, Janus kinase-2, Janus kinase 2, Jak-2, Janus Kinase2, 5UT1, 5UT4, 5UT5, JAK 2, 5UT3, JAK2 p, Janus-kinase-2, 3KRR, JAK2p, Janus-kinase 2, januskinase-2 |
| BCL2L1 | ENSP00000302564 | BCL-XL, BCL-2 family, BclXL, BCL-XS, Bcl2l1, Bcl-x, Bcl2l, BCL2 family, Bclx, apoptosis regulator Bcl-X, Bcl-2-family, BCLX-L, BCLX-S, Bcl-x L, Bcl-xL/S, Bcl- xS, BCL2-family, Bcl2-like 1, bcl-x-L, Bcl2-L1, BclXs, Bcl 2-family, BCL-2-like 1, Bcl-2-like protein 1, 2 LPC, hBCL2L1, Bcl xl, BCL2 like 1 |

| | | |
|---|---|---|
| NQO1 | ENSP00000319788 | NQO1, DTD, diaphorase, NMOR1, DIA4, DT-diaphorase, QR1, NAD(P)H:quinone oxidoreductase 1, DIA 4, DT diaphorase, NAD(P)H:quinone oxidoreductase1, NAD(P)H: quinone oxidoreductase 1, hNQO1, NAD(P)H: quinone oxidoreductase1, NAD(P)H:quinone oxidoreductase-1, NAD(P)H:quinone oxido-reductase 1, quinone reductase 1, NQO-1, NAD(P)H:quinoneoxidoreductase 1, NAD(P)H: quinone oxidoreductase-1, NAD(P) H:quinone oxidoreductase 1, NAD(P)H dehydrogenase, quinone 1, NAD(P)H:Quinone Oxidoreductase 1, NAD(P)H dehydrogenase [quinone] 1, NAD (P) H: quinone oxidoreductase 1, NAD(P)H quinone dehydrogenase 1, NAD (P)H:quinone oxidoreductase 1, NAD(P)H Quinone Dehydrogenase-1, 1 kBq, NQ-O1, dt-d, azoreductase, NAD(P)H: quinoneoxidoreductase1 |
| RELA | ENSP00000384273 | p65, RelA, transcription factor p65, NFKB3, Rel A, Rel-A, v-rel avian reticuloendotheliosis viral oncogene homolog A, p-65, p65-P |
| CD44 | ENSP00000398632 | CD44, Heparan sulfate proteoglycan, CDw44, pgp1, Pgp-1, MC56, CD44 molecule, MIC4, Hermes antigen, heparan-sulfate proteoglycan, CD 44, heparansulfate proteoglycan, CD44 antigen, hyaluronate receptor, CD-44, heparan-sulfate-proteoglycan, HCELL, heparan sulfate-proteoglycan, CD44 molecule (Indian blood group, CD44R, ECMRIII |
| ICAM1 | ENSP00000264832 | ICAM-1, intercellular adhesion molecule-1, Hb B2, CD54, ICAM1, human rhinovirus receptor, intercellular adhesion molecule 1, BB2, HbB2, Hb-B2, CD 54, ICAM 1, ICAM- 1, 1 p53, inter-cellular adhesion molecule-1, intercellular-adhesion-molecule-1, Inter-Cellular Adhesion Molecule 1, 1-p53, intercellular adhesion molecule -1, intercellular-adhesion molecule 1, P3.58, intercellular adhesion molecule1, intercellular adhesionmolecule-1 |

| | | |
|---|---|---|
| APP | ENSP00000284981 | amyloid precursor protein, APP, amyloid-beta precursor protein, amyloid-precursor protein, protease nexin 2, PN-II, Protease nexin-2, PN-2, APP-I, 1 AML, AD1, amyloid beta precursor protein, 2 BP4, Aa----a, Aa A, CTF-gamma, Protease nexin-II, protease nexin II, amyloid precursor-protein, amyloid b precursor protein, 2 LOH, CTFgamma, PreA4, 5 HOX, amyloid beta (A4) precursor protein, amyloid-beta-precursor protein, amyloid beta-precursor protein, cVAP, amyloid-precursor-protein, HA4, 1IYT, 2BEG, PN2, amyloid pre-cursor protein, AD-1, amyloid-beta A4 protein, amyloid beta A4 protein |
| PPARG | ENSP00000287820 | PPAR gamma, PPARgamma, PPAR-gamma, PPARG, peroxisome proliferator-activated receptor gamma, peroxisome proliferator-activated receptor-gamma, peroxisome proliferator activated receptor gamma, peroxisome proliferator activated receptor-gamma, peroxisome-proliferator-activated receptor-gamma, 5-two, peroxisome-proliferator-activated receptor gamma, peroxisome proliferator- activated receptor gamma, PPARg amma, NR1C3, peroxisome-proliferator activated receptor-gamma, PPARG2, peroxisome-proliferator activated receptor gamma, Peroxisome proliferator-activated receptorgamma, peroxisome proliferator activated-receptor gamma, peroxisome proliferator activated receptorgamma, PPAR-g, PPAR- gamma, Pparg1, peroxisome proliferatoractivated receptor-gamma, Peroxisome proliferator-activated- receptor-gamma, peroxisome proliferator-activated receptor g, PPARG-2, peroxisome proliferator-activated receptor g, peroxisome proliferator-activated receptor-g, PPAR-g2, peroxisome-proliferator-activated-receptor-gamma, PPAR G, C-IMT 1, peroxisome proliferator activated receptor - gamma |
| CYP19A1 | ENSP00000379683 | aromatase, CYP19, CYP19A1, CYP XIX, P450arom, P-450Arom, CYP 19, cytochrome P-450Arom, estrogen synthase, cytochrome P450arom, ARO1, P450 arom, cytochrome P450, subfamily XIX, cytochrome P450 19A1, Aro 1, cytochrome P450, family 19, subfamily A, polypeptide 1, cytochrome p450 family 19, CYP-19, cytochrome P450 family 19 subfamily A member 1 |

| | | |
|---|---|---|
| HSPA4 | ENSP00000302961 | Hsp70, Hsp70RY, hsp70 RY, Apg-2, HS24/p52, HSP-70, HSph2, HSP 70, heat-shock 70 kDa proteins, heat shock 70-kDa protein 4, heat shock 70 kDa proteins, HSPA4, HapG2, Heat shock protein family A member 4, hHSP70, heat shock 70 kDa protein 4 |
| TNFRSF10B | ENSP00000276431 | death receptor 5, DR5, TRAIL-R2, TRICK2, TNFRSF10B, KILLER/DR5, death receptor-5, DR-5, KILLER/ DR5, kill ER, TRAILR2, TRAIL R2, hDR5, TNF-related apoptosis-inducing ligand receptor 2, TNF-receptor superfamily member 10b, TNF-related apoptosis-inducing ligand receptor-2, TNF receptor superfamily member 10b |
| EDN1 | ENSP00000368683 | endothelin-1, ET-1, EDN1, preproendothelin-1, PPET-1, endothelin 1, ET1, pre-proendothelin-1, hET-1, EDN-1, prepro endothelin-1, prepro-endothelin-1, ppET1, endothelin- 1, endothelin1, ET- 1, preproendothelin 1, endothelin - 1, EDN 1, E- T1 |
| NOS3 | ENSP00000297494 | eNOS, NOS 3, NOS3, endothelial nitric oxide synthase, endothelial NOS, Endothelial nitric-oxide synthase, NOS-3, nitric oxide synthase 3, constitutive NOS, ecNOS, nitric oxide synthase-3, AF519768, endothelial nitric oxide-synthase, e-NOS, nitric-oxide synthase 3, ec-NOS, hNOS3, 3 NOS, endothelial-nitric-oxide synthase, Endothelial nitric oxide synthase, endothelial-NOS, NOS type III, endothelial-nitric oxide synthase |
| GORASP1 | ENSP00000313869 | p65, GRASP65, GOLPH5, GORASP1, Golgi peripheral membrane protein p65, GRASP-65, p-65, p65-P |
| WNK1 | ENSP00000341292 | p65, WNK1, HSN2, serine/threonine protein kinase WNK1, kinase-deficient protein, PRKWNK1, Kdp, p-65, WNK-1, p65-P, WNK lysine deficient protein kinase 1, pSK |
| SYT1 | ENSP00000261205 | SYT, p65, synaptotagmins, synaptotagmin I, Synaptotagmin-I, synaptotagmin-1, Syt1, synaptotagmin 1, Syt 1, p-65, p65-P, Synaptotagmin1, Syt-1 |
| APOB | ENSP00000233242 | apo B, apolipoprotein B, ApoB, apo-B, apoB-100, apolipoprotein B100, apolipoprotein B-100, apoB-48, apoB48, apo B48, apo B-100, apolipoprotein-B, apo-B-48, Apo-B-100, apo-B100, apo B-48, apo-B48, apoB100, apo B100, apolipo-protein B100, apolipoprotein B-100, ApolipoproteinB 100, apoB 100, apolipoprotein B 100, apo B 100, apolipoprotein-B 100, apolipoprotein-B-100, apo-lipoprotein B-100 |
| PSMC6 | ENSP00000401802 | p44, p42, PSMC6, PSMC6P, p 44, proteasome subunit p42, p-44 |

| | | |
|---|---|---|
| CAV1 | ENSP00000339191 | caveolin-1, Cav-1, VIP21, CaV1, CGL3, caveolin 1, caveolin1, Pph3, AF125348, caveolin- 1, cav- 1, caveolin -1 |
| PLAUR | ENSP00000339328 | uPAR, PLAUR, u-PAR, urokinase-type plasminogen activator receptor, CD87, uPA-R, MO3, Urokinase type plasminogen activator receptor, urokinase plasminogen activator surface receptor, u-PA-R, urokinase-type plasminogen activator receptor, plasminogen activator, urokinase receptor |
| CCN2 | ENSP00000356954 | CTGF, CCN2, connective tissue growth factor, IGFBP-8, CCN-2, connective-tissue growth factor, connective-tissue growth-factor, C onnective tissue growth factor, C TGF, CCN family member 2 |
| N | ENSP00000277541 | Notch1, Notch, Notch-1, Notch 1, TAN-1, Tan1, HHN1, HN-1, Notch -1, HN1, 3 NBN, Notch homolog 1, translocation-associated, neurogenic locus notch homolog protein 1 |
| ABCB1 | ENSP00000478255 | MDR1, P-gp, ATP-binding cassette sub-family B member 1, ATP-binding cassette subfamily B member 1, ABCB1, MDR-1, multidrug resistance protein 1, P-glycoprotein 1, PGY1, gp170, MDR1 P, multi-drug resistance protein-1, pgp, mdr 1, multidrug-resistance protein 1, MDR1-P, multidrug resistance protein-1, MDR-1 P, MDR- 1, MDR1p, multi drug resistance protein 1, ATP-binding cassette, subfamily B, member 1, ATP-binding cassette, sub-family B, member 1, ATP-binding cassette sub-family B member -1, ATP-binding cassette, sub-family B (MDR/TAP), member 1, MDR1 human, multi-drug resistance protein 1, ATP binding cassette sub-family B member 1, ClCs, CD243, ATP-binding cassette, subfamily B (MDR/TAP), member 1, ATP binding cassette subfamily B member 1, ATP-binding cassette subfamily B, P-glycoprotein-1, PGY-1, ABCB1 p, GP-170, hMDR1, hABCB1, ATP-Binding Cassette sub-family B, ATP-bindingcassette sub-family B member 1, Ab-cb-1, ABC-20, multidrug-resistance-protein-1 |
| CREB1 | ENSP00000387699 | CREB, Creb-1, CREB1, cyclic AMP responsive element-binding protein-1, cyclic AMP-responsive element binding protein-1, cAMP-responsive element-binding protein 1, cyclic AMP-responsive element binding protein 1, cAMP-responsive element binding protein 1, cAMP responsive element binding protein 1, cAMP responsive element-binding protein1, cAMP responsive element binding protein-1, cyclic AMP responsive element binding protein 1, cAMP responsive element-binding protein 1, cyclic-AMP responsive element binding protein 1 |

| | | |
|---|---|---|
| TLR4 | ENSP00000363089 | TLR4, toll-like receptor-4, TLR-4, Toll-like receptor 4, hTLR4, hToll, TLR 4, TOLL, Toll Like Receptor-4, toll like receptor 4, Toll-like-receptor 4, toll like receptors, Tolllike receptor 4, Tlr, Toll-like receptor4, toll- like receptors, TLR- 4, toll- like receptor4, Toll like receptor4, Toll-Like-Receptors, Toll-Like-Receptor-4, Toll- like receptor 4 |
| AKR1B1 | ENSP00000285930 | AR, adr, ALR2, aldose reductase, AKR1B1, EC 1.1.1.21, aldehyde reductase, aldo-keto reductases, aldoketoreductases, 2 in E, 2 PDL, aldose-reductase, 3 GHR, aldo-keto reductase family 1, member B1, aldo-keto reductase family 1 member B1, 4 PRR, 2 ACs, 3 GHS, 2 PDX, aldoketo reductases, 4 IGs, 2 PDN, 2 PDB, ALDR1 |
| GPER1 | ENSP00000380281 | GPR30, FEG-1, MER, GPCR-Br, GPCR -Br, CMKRL2, LyGPR, lymphocyte derived G-protein-coupled receptor, Membrane estrogen receptor, LERGU, G-protein coupled receptor 30, LERGU-2, G protein-coupled receptor 30, G-protein-coupled receptor 30, G protein-coupled estrogen receptor, GPER, G-protein-coupled receptor-30, G protein-coupled estrogen receptor 1, G-protein-coupled estrogen receptor, GPER-1, GPER1, GPR 30, G-protein-coupled estrogen receptor 1, G-protein coupled estrogen receptor, G protein coupled estrogen receptor, G protein-coupled receptor-30, G-protein-coupled estrogen receptor-1, G protein-coupled estrogen receptor-1, G protein coupled receptor 30, GPER 1, G-protein coupled estrogen receptor 1 |
| IL10 | ENSP00000412237 | TGIF, IL-10, interleukin-10, IL10, Interleukin 10, hIL-10, interleukin10, cytokine synthesis inhibitory factor, CSIF, hIL10, IL 10, interleukin- 10, IL- 10, IL-10 A, h-IL10, Interleukin -10, interleukin--10, IL10 A, IL10A, h-IL-10 |
| CXCR4 | ENSP00000386884 | CXCR-4, CXCR4, hFB22, LESTR, Fusin, chemokine receptor 4, LCR1, leukocyte-derived seven-transmembrane domain receptor, HM89, D2S201E, LAP3, LCR 1, CD184, C-X-C chemokine receptor type-4, NPY-R, CXCR 4, L-CR1, C-X-C chemokine receptor type 4, chemokine (C-X-C motif) receptor 4, C-X-C motif chemokine receptor 4, LAP 3, chemokine receptor-4, chemokine (CXC motif) receptor 4, CXC chemokine receptor type 4, CXC motif chemokine receptor 4, CHEMOKINE, C-X-C-motif chemokine receptor 4, hCXCR4, C-X-C motif chemokine receptors, C-X-C motif chemokine receptor-4 |

| | | |
|---|---|---|
| NTRK2 | ENSP00000277120 | TrkB, NTRK2, neurotrophic tyrosine kinase receptor type 2, TRK-B, TRK-B tyrosine kinase, TrkB tyrosine kinase, tropomyosin-related kinase B, gp145trkB, Trk B, tropomyosin related kinase B, neurotrophic tyrosine kinase, receptor, type 2, neurotrophic receptor tyrosine kinase 2, NTRK-2, neurotrophic tyrosine kinase receptor type2, tropomyosin-related kinase-B |
| NOS2 | ENSP00000327251 | iNOS, NOS2, NOS2A, inducible NO synthase, hep-NOS, nitric oxide synthase-2, nitric oxide synthase 2, nitric oxide synthase 2A, hepatocyte NOS, NOS-2, nitric oxide-synthase-2, inducible NO-synthase, 4 NOS, nitric oxide synthase2, EC 1.14.13.39, nitric oxide synthase-2, i-NOS, hNOS2, Nitric-Oxide Synthase 2, NOS type II, NOS 2 |
| PARP1 | ENSP00000355759 | PARP, Adprt, poly(ADP-Ribose) polymerases, PARP-1, ADP-ribosyltransferase, PARP1, Poly(ADP-ribose) polymerase-1, poly(ADP-ribose) polymerase 1, poly (ADP-ribose) polymerase-1, poly (ADP ribose) polymerase-1, ADP-ribosyl transferase, EC 2.4.2.30, poly(ADP-ribose)polymerase-1, poly (ADP-ribose) polymerase 1, poly(ADP-ribose)polymerase 1, poly (ADP-ribose) polymerases, ADP ribosyltransferase, ADPRT1, poly (ADP ribose) polymerases, hPARP-1, poly-(ADP-ribose) polymerase 1, ADP ribosyl transferase, poly (ADP ribose) polymerase 1, poly [ADP-ribose] polymerase 1, PARP 1, poly(ADP ribose) polymerase-1, ARTD1, ARTD-1, poly(ADP-ribose)polymerase1, Poly (ADP-ribose) polymerase1, poly-[ADP-ribose] polymerase-1, Poly-(ADP-ribose)-polymerase1, poly[ADP-ribose] polymerase-1, ZINC fingers, Poly (ADPRibose) Polymerase-1, Poly-(ADP-Ribose) Polymerase-1, Poly[ADP-ribose] polymerase 1, poly(ADP-ribose)-polymerase 1, Poly(ADP-ribose)polymerases, poly-(ADP-ribose)-polymerase 1, Poly (ADP-ribose) polymerase- 1, poly(ADP ribose) polymerase 1 |
| CD40LG | ENSP00000359663 | TNF, CD40 ligand, CD40L, tumor-necrosis factor, TNF-related activation protein, HIGM1, gp39, CD154, CD40LG, CD40-ligand, hCD40L, tumor necrosis-factor, tumor-necrosis-factor, tumor necrosis factor superfamily, CD40-L, tumor-necrosis factor superfamily, TNFSF5, Tumor Necrosis Factor, CD 40L, T cell antigen gp39, CD-40 ligand, CD 154, CD 40 ligand, tumor necrosis factor super-family, tumor necrosis factor-superfamily, CD 40 L, tumor necrosis factor super family, CD40 L |

| | | |
|---|---|---|
| IL4 | ENSP00000231449 | IL-4, interleukin-4, interleukin 4, IL 4, IL4, B-cell stimulatory factor 1, hIL-4, BSF-1, 2 in T, hIL4, interleukin4, IL-4p, IL -4, H IL4, IL- 4 |
| CRP | ENSP00000255030 | C-reactive protein, Ptx-1, PTX1, CRP, C reactive protein, short pentraxins, hPTX1, C-reactive-protein, C-RP, C-reactive protein, pentraxin related, C- reactive protein, C - reactive protein, C--reactive protein, Creactive protein, CR P |
| PPARA | ENSP00000385523 | PPARalpha, peroxisome proliferator-activated receptor alpha, PPAR alpha, PPAR-alpha, PPAR, peroxisome-proliferator-activated receptor alpha, peroxisome proliferator activated receptor alpha, Ppara, hPPAR, peroxisome proliferator-activated receptor-alpha, peroxisome-proliferator-activated receptor-alpha, Nr1c1, peroxisome proliferator activated receptor-alpha, Peroxisome proliferator--activated receptor alpha, PPAR- alpha, peroxisome-proliferator-activated receptor-a, peroxisome-proliferator-activated-receptor-alpha, peroxisome proliferative activated receptor,  alpha, 4 BCR, PPAR-a, PPAR A, NR1 C1 |
| GCG | ENSP00000387662 | GLP-1, glucagon-like peptide 1, GLP-2, glp1, glucagon, Glucagon-like peptide-1, preproglucagon, glucagon-like peptide 2, Glucagon-like peptide-2, Gcg, Glucagon-like-peptide-1, Glucagonlike peptide-1, hGLP-1, Glucagon like peptide 1, glucagon-like peptide-1, hGLP-2, glucagon like peptide-2, glucagon-like-peptide 1, glucagon like peptide-1, GLP2, glucagonlike peptide 1, GRPP, glucagon- like peptide- 1, GLP- 1, Glucagon-likepeptide1, Glucagon like-peptide-1, glucagon like peptide -1, glucagon-like peptide- 1, glucagon-like peptide -1, GLP 1, pre-proglucagon, Glucagon like peptide 2, GLP -1, prepro-glucagon, glucagon-likepeptide-2 |

| | | |
|---|---|---|
| GSTM1 | ENSP00000311469 | GSTM1, GST-1, GST1, GSTM1-1, mu1, glutathione S-transferase M1, glutathione-S-transferase M1, GTH4, GST M1, EC 2.5.1.18, GSTM 1, glutathione S-transferase mu 1, glutathione S-transferase mu-1, GSTM-1, glutathione-S-transferase Mu1, glutathione S-transferase M 1, Glutathione S-transferase mu1, GST-M1, glutathione-S-transferase mu 1, glutathione S-transferase -M1, glutathione-S transferase mu 1, glutathione- S-transferase M1, glutathione S-transferase-M1, glutathione S transferase M1, glutathione Stransferase M1, hGSTM1, Glutathione-S-transferaseM1, glutathione S- transferase M1, Mu-1, Glutathione -S -transferase M1, 1xWk, Mu 1, Glutathione S transferase mu 1, Glutathione-S Transferase M1 |
| MPO | ENSP00000225275 | myeloperoxidase, MPO, myelo-peroxidase, Myelo per oxidase |
| ZHX2 | ENSP00000314709 | Raf, ZHX2, Afr1, Zinc fingers and homeoboxes 2, ZHX-2, zinc fingers and homeoboxes protein 2, zinc-fingers and homeoboxes 2 |
| SPP1 | ENSP00000378517 | Osteopontin, SPP1, Opn, urinary stone protein, Eta-1, SPP-1, BspI, secreted phosphoprotein 1, sibling family, eta1, bone sialoprotein-1, Secreted phosphoprotein-1, uropontin, bone sialoprotein I, hSPP1, early T-lymphocyte activation-1, bone sialoprotein 1, early T-lymphocyte activation 1 |
| MTTP | ENSP00000427679 | microsomal triglyceride transfer protein, ABL, microsomal triglyceride-transfer protein, Mttp, microsomal triglyceride transfer protein large subunit, microsomal triglyceride-transfer-protein, MT-TP, mtp, Mt-P |
| AREG | ENSP00000379097 | AR, Amphiregulin, schwannoma-derived growth factor, AREG, SDGF |
| MMP3 | ENSP00000299855 | Str1, MMP3, stromelysin-1, MMP-3, STMY1, matrix metalloproteinase-3, SL1, STMY, SL-1, stromelysin 1, matrix metalloproteinase 3, matrix metalloprotein-ase-3, Sl 1, matrix metallo-proteinase-3, matrix-metalloproteinase-3, Matrix metalloproteinase3, stromelysin- 1, matrixmetalloproteinase-3, matrix metallopeptidase 3, matrix metallopeptidase-3, MMP 3, MMP- 3 |

| | | |
|---|---|---|
| HMGB1 | ENSP00000345347 | HMG-1, HMGB1, High-mobility group box 1, high-mobility-group box 1, high-mobility group box-1, HMG1, high mobility group protein 1, high mobility group box 1, amphoterin, SBP-1, HMGB 1, HMGB-1, high mobility group protein-1, High-mobility group protein 1, high mobility group protein B1, high mobility group box-1, high mobility group box1, high-mobility group box1, high-mobility group-box 1, high mobility group-box 1, High-mobility group protein B1, high-mobility-group-box-1, High-Mobility-Group-Protein B1, high mobility group box -1, high-mobility group box 1, SBP1, high-mobility group-box-1, HMGB -1, High Mobility Group Protein-B1 |
| CYCS | ENSP00000307786 | cytochrome c, CYCS, cyc, cytochrome c, somatic, cytochrome-c, cytochromec |
| ABCG2 | ENSP00000237612 | ABCG2, breast cancer resistance protein, BCRP, ABCP, MXR1, mitoxantrone resistance-associated protein, Bcrp1, urate exporter, MXR, ATP-binding cassette, subfamily G, ABC-P, ABCG-2, breast cancer-resistance protein, MXR-1, ATP-binding cassette, subfamily G, member 2, ATP-binding cassette, subfamily G (WHITE), member 2, BCRP-1, hABCG2, ATP-binding cassette sub-family G member 2, ATP-binding cassette subfamily G member 2, CD338, ATP binding cassette subfamily G member 2, ATP-binding cassette subfamily G, ATP-binding cassette, sub-family G (WHITE), member 2, ABCG 2, ATP-binding cassette sub-family G, ATP binding cassette sub-family G member 2, breast-cancer resistance protein, ATP binding cassette subfamily G member 2 (Junior blood group |
| COMT | ENSP00000354511 | COMT, Catechol-O-methyltransferase, catechol O-methyltransferase, catechol-O-methyl transferase, catechol-O-methyl-transferase, EC 2.1.1.6, catechol O-methyl transferase, catechol-O-methyl- transferase, catechol- O-methyltransferase, Catechol- O -methyltransferase, catechol-Omethyltransferase, Catechol-O methyltransferase |

| | | |
|---|---|---|
| CXCL12 | ENSP00000379140 | sDF1, stromal cell-derived factor 1, SDF-1, CXCL12, SCYB12, sdf-1a, stromal cell-derived factor-1, sdf1a, stromal cell derived factor 1, stromal-cell-derived factor 1, stromal cell derived factor-1, chemokine ligand 12, SDF -1, SDF1B, chemokine (C-X-C motif) ligand 12, Stromal-cell derived factor-1, stromal-cell-derived factor-1, CXCL1-2, chemokine ligand-12, CXCL-12, Stromal-cell derived factor 1, CXCL 12, stromal cell-derived factor 1, C-X-C motif chemokine ligand 12, C-X-C motif chemokine 12, CXC motif chemokine 12, SDF1 A, Stromal cell-derived factor1, C-X-C motif chemokine-12, CXC-motif-chemokine 12, stromal cell -derived factor-1, intercrine reduced in hepatomas, pre-B-cell growth-stimulating factor |
| MAPK8 | ENSP00000378974 | Jnk1, Jun N-terminal kinase, MAPK8, SAPK1, jun-N-terminal kinase, JNK-1, c-Jun N-terminal kinase 1, c-Jun-N-terminal kinase 1, c-Jun N-terminal kinase-1, jun N terminal kinase, JNK 1, mitogen-activated protein kinase 8, c-Jun N-terminal Kinase1, JNk, MAPK 8, c-Jun-N-terminal kinase-1, Jun-N terminal kinase |
| DPP4 | ENSP00000353731 | dipeptidyl peptidase IV, DPP-IV, DPPIV, CD26, DPP4, CD 26, DPP IV, ADCP-2, ADAbp, ADCP2, dipeptidylpeptidase IV, dipeptidyl-peptidase IV, EC 3.4.14.5, 2 on C, dipeptidyl peptidase 4, T-cell activation antigen CD26, hCD26, Dipeptidyl peptidase-4, 2 OAG, dipeptidylpeptidase 4, Dipeptidyl peptidase-IV, dipeptidylpeptidase-4, DPP-4, dipeptidylpeptidase-IV, dipeptidyl-peptidase 4, Tp103, hDPP4, dipeptidyl-peptidase-4, DPP 4, 2 OLE, dipeptidyl peptidase4, Dipeptidyl-peptidase-IV, dipeptidyl peptidase- 4, adenosine deaminase complexing protein 2, 3 CCC |
| NQO2 | ENSP00000369822 | NQO2, hNQO2, NRH:quinone oxidoreductase 2, NRH:quinone oxidoreductase2, quinone reductase 2, NRH: quinone oxidoreductase2, 3 NHL, QR2, NRH: quinone oxidoreductase 2, Quinone Reductase-2, NRH:quinoneoxidoreductase-2 |
| CYP1B1 | ENSP00000478561 | CYP1B1, cytochrome P450 1B1, GLC3A, cytochrome P4501B1, cytochrome P450 family 1, EC 1.14.14.1, cytochrome P450-1B1, cytochrome P-450 1B1, cytochrome P 450 1B1, CYP 1B1, CP1B, U56438, P4501B1, Cytochrome P450, subfamily I, CYPIB1, hCYP1B1, cytochrome P450, family 1, subfamily B, polypeptide 1, P450 1B1, CYP1B1 p, cytochrome P450 family 1 subfamily B member 1 |

| | | |
|---|---|---|
| BACE1 | ENSP00000318585 | Asp 2, memapsin 2, BACE, beta-site APP-cleaving enzyme, BACE1, beta-site APP cleaving enzyme, beta-site APP cleaving enzyme 1, BACE-1, 3 in 3, Asp2, beta-site APP-cleaving enzyme 1, 3 in 4, 2 of 0, beta-site amyloid precursor protein-cleaving enzyme 1, beta-secretase-1, 3 in D, Asp-2, memapsin2, hBACE1, beta-site amyloid precursor protein-cleaving enzyme-1, Beta-site amyloid precursor protein cleaving enzyme 1, beta-secretase 1, beta-site-APP-cleaving enzyme-1, BACE 1, Memapsin-2, beta-site APP cleaving enzyme-1, EC 3.4.23.46, beta-site amyloid precursor protein cleaving enzyme-1, 4 dpf, 2 - q11, beta site APP cleaving enzyme 1, beta site amyloid precursor protein cleaving enzyme 1, 3 CIC, 2QP8, 2FDP, 4B70, 2WEZ, 3R1G, Beta Site Amyloid Precursor Protein Cleaving Enzyme-1, Beta-site amyloid-precursor-protein cleaving enzyme 1, 1FKN, Beta secretase 1, 1W51, Beta site APP Cleaving Enzyme, Beta-site amyloid precursor protein cleaving enzyme1 |
| ABL1 | ENSP00000361423 | c-Abl, v-Abl, Abl, BCR/ABL, p150, ABL1, protooncogene c-abl, c- abl, proto-oncogene c-abl, BCR/ ABL, Abl family tyrosine kinases, Abl-1, Abelson murine leukemia viral oncogene homolog 1, 1OPL, v-Abl Abelson murine leukemia viral oncogene homolog 1, 2-abl, c-abl oncogene 1, receptor tyrosine kinase, c-abl oncogene 1, non-receptor tyrosine kinase, Abelson tyrosine-protein kinase 1, c-ABL1, C-abl oncogene 1, nonreceptor tyrosine kinase, cAbl, ABL proto-oncogene 1, nonreceptor tyrosine kinase, ABL 1, tyrosine protein kinase ABL1, 1BBz, ABL proto-oncogene 1, non-receptor tyrosine kinase, tyrosine-protein kinase ABL1, Abelson murine leukemia viral oncogene homolog1, BCR / ABL |
| BRCA2 | ENSP00000369497 | BRCA2, FANCD, BRCC2, BRCA-2, U43746, breast cancer 2, early onset, XRCC11, BRCA 2, BRCA2 p, GLM-3, breast cancer type 2 susceptibility protein, Breast cancer 2, BRCA2-P |

| | | |
|---|---|---|
| TNFSF11 | ENSP00000381775 | ODF, OPGL, TRANCE, RANKL, CD254, Tnfsf11, osteoclast differentiation factor, receptor activator of nuclear factor-kappaB ligand, osteoprotegerin-ligand, tumor necrosis factor superfamily, Receptor activator of nuclear factor-kappa B ligand, osteoprotegerin ligand, TNF-related activation-induced cytokine, RANK-L, receptor activator of nuclear factor kappaB ligand, tumor-necrosis factor superfamily, OPG-L, sODF, AB037599, receptor-activator of nuclear factor-kappaB ligand, receptor activator of nuclear factor kappa B ligand, receptor activator of nuclear factor kappa-B ligand, tumor necrosis factor super-family, tumor necrosis factor-superfamily, receptor-activator of nuclear-factor kappa B ligand, receptor activator of nuclear factor-kappa-B ligand, Receptor Activator of Nuclear Factor Kappa- B Ligand, tumor necrosis factor super family, Receptor-Activator of Nuclear Factor-Kappa B Ligand, RANK L, receptor activator of nuclear factor kappa-B- ligand |
| SOD1 | ENSP00000270142 | SOD1, SOD-1, Superoxide dismutase 1, superoxide dismutase-1, EC 1.15.1.1, hSOD1, IPO-A, Superoxide dismutase 1, soluble, SOD 1, h-SOD1, hSOD-1, Als1p, superoxide dismutase1, 1 U x L, superoxide dismutase [Cu-Zn, superoxide dismutase -1, SOD1 p, super-oxide dismutase 1, superoxide-dismutase-1 |
| IL18 | ENSP00000280357 | IL-18, IL18, IGIF, interleukin-18, Interleukin 18, interferon-gamma-inducing factor, interferon-gamma-inducing-factor, interferon gamma inducing factor, interferon gamma-inducing factor, hIL-18, IL 18, IL-1F4, hIL18 |
| NLRP3 | ENSP00000337383 | AVP, cryopyrin, CIAS1, NALP3, Nlrp3, PYPAF1, NLR family, pyrin domain containing-3, NLR family, pyrin domain containing 3, NLR family pyrin domain containing 3, NACHT, LRR and PYD domains-containing protein 3, NLRP3 p, CIAS-1, NALP-3, PYPAF-1, Fcas, nucleotide-binding oligomerization domain, leucine-rich repeat and pyrin domain containing 3, NLR family pyrin domain-containing 3, NLR family, pyrin domain-containing 3, NLR family pyrin domain containing-3, NLRP-3, nucleotide-binding oligomerization domain-, leucine-rich repeat- and pyrin domain-containing 3, NLR Family-Pyrin Domain-Containing 3, NLRP 3, nucleotide-binding oligomerization domain-, leucine-rich repeat and pyrin domain-containing 3 |

| | | |
|---|---|---|
| YBX1 | ENSP00000361626 | YB-1, dbpB, CBFA, YB1, Ybx1, U11/U12 di-snRNP, Y box-binding protein-1, CBF-A, Y-box binding protein-1, DNA-binding protein B, Y-box binding proteins, Y-box-binding protein-1, Y box binding protein 1, Y-box binding protein 1, Y-box-binding protein 1, Y box-binding protein 1, Hyb1, Y-box transcription factor, Y-box binding protein-1, Y box binding protein-1, BP-8 |
| GDNF | ENSP00000409007 | GDNF, glial cell line-derived neurotrophic factor, glial-cell-line-derived neurotrophic factor, GDNF family ligands, ATF1, ATF2, ATF-2, glial-derived neurotrophic factor, ATF-1, glial cell-line-derived neurotrophic factor, glial cell line derived neurotrophic factor, ATF 1, glial cell-derived neurotrophic factor, Glial cell-line derived neurotrophic factor, Atf, glial cell derived neurotrophic factor, glial cell line--derived neurotrophic factor, 3FUB, Glial derived neurotrophic factor, Glial-cell line-Derived Neurotrophic Factor, glial-cell-line derived neurotrophic factor, Glialcell line-derived neurotrophic factor, Glial-cell line derived neurotrophic factor, glial-cell derived neurotrophic factor, glial cell line -derived neurotrophic factor |
| CYP2E1 | ENSP00000440689 | CYP2E1, CYP2E, cytochrome P450-2E1, Cyp2e-1, cytochrome P450 2E1, P450j, cytochrome P-4502E1, Cytochrome P4502E1, CYP 2E1, cytochrome P-450 2E1, hCYP 2E1, CYPIIE1, cytochrome P 4502E1, cytochrome P450, family 2, subfamily E, polypeptide 1, cytochrome P450 family 2 subfamily E member 1, cytochrome P-450j, cytochrome P45 02E1 |
| BRAF | ENSP00000288602 | p94, B-Raf, BRAF, RAF family, serine/threonine-protein kinase BRAF, B-raf-1, v-raf murine sarcoma viral oncogene homolog B1, proto-oncogene BRAF, v-raf murine sarcoma viral oncogene homolog B, proto-oncogene B-raf, B RA F, BRAF1, BRAF-1, serine/threonine-protein kinase B-raf, BRAF human, protooncogene BRAF, B-Raf proto-oncogene, serine/threonine kinase, vRAF murine sarcoma viral oncogene homolog B1, serine/threonine protein kinase B-raf |
| PRL | ENSP00000302150 | PRL, prolactin, pro-lactin, PR l, Prl human, growth hormone family |

| | | |
|---|---|---|
| SP1 | ENSP00000329357 | Sp1, transcription factor Sp-1, transcription factor Sp1, Sp-1, specificity protein 1, Sp 1, Sp1 transcription factor, specificity protein-1, SP1-transcription-factor, transcription factor Sp 1, SP-1 transcription factor, SP 1 transcription factor, HSP1, Sp transcription factors, Sp-transcription factors, transcription-factor Sp1 |
| ISYNA1 | ENSP00000337746 | iNOS, myo-inositol 1-phosphate synthase, ISYNA1, IPS-1, EC 5.5.1.4, INO1, IPS1, i-NOS, Inositol-3-phosphate synthase 1 |
| ADIPOQ | ENSP00000389814 | apM1, GBP28, apM-1, adiponectin, Acrp30, Adipoq, 30-kDa adipocyte complement-related protein, gelatin-binding protein, AdiPose Most abundant Gene transcript 1, ADPN, adipose most abundant gene transcript-1, Acdc, HGNC:13633, adiponectin, C1Q and collagen domain containing, Acrp 30 |
| SERPINE1 | ENSP00000223095 | PAI-1, Plasminogen activator inhibitor-1, SERPINE1, PAI1, plasminogen activator inhibitor 1, pAI, plasminogen-activator-inhibitor-1, plasminogen activator inhibitor, type I, plasminogen-activator inhibitor 1, PLANH1, Endothelial plasminogen activator inhibitor, plasminogen-activator inhibitor-1, PA-I, plasminogen activator inhibitor -1, serpin E1, serpine 1, PA I-1, PA I, SERPINE-1, hPAI-1, PAI 1, plasminogen activator-inhibitor 1, serpin family E member 1, plasminogen activator-inhibitor-1, serpin peptidase inhibitor, clade E, member 1, PAI -1, plasminogen activator inhibitor - 1, PAI- 1 |
| GDF15 | ENSP00000252809 | MIC-1, macrophage inhibitory cytokine-1, Growth differentiation factor 15, GDF15, GDF-15, PLAB, Growth/differentiation factor 15, Growth differentiation factor-15, macrophage inhibitory cytokine 1, placental bone morphogenetic protein, MIC1, placental TGF-beta, Growth/differentiation factor-15, NAG-1, macrophage-inhibitory cytokine-1, Nonsteroidal anti-inflammatory drug-activated gene-1, prostate differentiation factor, nonsteroidal antiinflammatory drug-activated gene-1, growth-differentiation factor-15, non-steroidal anti-inflammatory drug-activated gene-1, growth-differentiation factor 15, nonsteroidal anti-inflammatory drug-activated gene 1, non-steroidal anti-inflammatory drug activated gene-1, NSAID-activated gene 1 protein, PTGFB, GDF 15, Growth differentiation factor- 15 |

| | | |
|---|---|---|
| TIMP1 | ENSP00000218388 | Epo, TIMP1, TIMP, TIMP-1, fibroblast collagenase inhibitor, erythroid-potentiating activity, tissue inhibitor of metalloproteinases 1, tissue inhibitor of metalloproteinase-1, Tissue inhibitor of metalloproteinases-1, TIMP- 1, hTIMP-1, TIMP 1, tissue inhibitor of metalloproteinase 1, tissue-inhibitor of metalloproteinase-1, metalloproteinase inhibitor 1, metalloproteinase inhibitor-1, TIMP metallopeptidase inhibitor 1, TIMP metallopeptidase inhibitor-1, hci, tissue inhibitor of metalloproteinases1, TIMP -1, Erythroid potentiating activity |
| FN1 | ENSP00000346839 | fibronectin, FN1, Fn-1, cold insoluble globulin, EDB, fibronectin 1, 2-EC3, MSF, cold-insoluble globulin, ED-B, cIg, C-Ig, fibronectin-1, migration stimulating factor, fibronectin1, Migration-stimulating factor |
| NF1 | ENSP00000351015 | HNF1, NF1, neurofibromin, NF-1, 1 NF1, Hnf-1, NF 1, Neurofibromin 1, VRNF, neuro-fibromin, 3 PEG, WSs, neurofibromin-1, NF1 human, Neurofibromin1 |
| NR1I2 | ENSP00000336528 | SXR, PAR1, PXR, PAR-1, hPAR-2, pregnane X receptor, PAR2, steroid and xenobiotic receptor, orphan nuclear receptor PXR, NR1I2, PAR 2, PR r, PAR-2, Par 1, pregnane-X receptor, pregnane-X-receptor, hPar1, hPar2, nuclear receptor subfamily 1 group I member 2, pregnane X-receptor, NR 1I2 |
| ABCA1 | ENSP00000363868 | ATP-binding cassette transporter 1, ABC1, ATP-binding cassette transporter-1, ABCA1, hABC1, ATP binding cassette transporter 1, cholesterol efflux regulatory protein, CERP, ATP-binding cassette, subfamily A, member 1, AbC-1, cholesterol-efflux regulatory protein, hABC-1, ATP-binding-cassette transporter 1, hABCA1, ABCA-1, ATP-binding cassette, sub-family A, member 1, ATP-binding cassette sub-family A member 1, ABC-A1, ABCA1p, ABCA1 p, ATP-binding cassette subfamily A member 1, ATP binding cassette subfamily A, ATP binding cassette subfamily A member 1, ATP-binding cassette subfamily A, ATP-binding cassette transporter1, ABCA 1, ATP-binding cassette sub-family a member1 |

| | | |
|---|---|---|
| SLC2A1 | ENSP00000416293 | GLUT1, GLUT, Glut-1, SLC2A1, DYT9, HepG2 glucose-transporter, HepG2 glucose transporter, DYT17, DYT18, Hep G2 glucose transporter, hGluT-1, GLUT 1, cse, solute carrier family 2 (facilitated glucose transporter), member 1, glut1DS, GLUT1-P, GLUT- 1, solute carrier family 2, member 1, CS-E, Hep-G2 glucose transporter, Solute carrier family 2 member 1, GLUT-1DS, Solute carrier family 2, facilitated glucose transporter member 1 |
| MMP13 | ENSP00000260302 | collagenase-3, MMP13, CLG3, collagenase 3, MMP-13, matrix metalloproteinase-13, matrix metalloproteinase 13, hMMP-13, hMMP13, matrix metallopeptidase 13, matrixmetalloproteinase-13, Matrix metalloproteinase13, Matrix Metallopeptidase-13, MMP-1-3, 1XUC, 3WV1, matrix-metalloproteinase 13 |
| ZNRD2 | ENSP00000312318 | p27, p2-7, p2 7, autoantigen P27 |
| HP | ENSP00000348170 | haptoglobin, hPR, haptoglobin-related protein, zonulin, Haptoglobin related protein, HP r, B_P |
| NGF | ENSP00000358525 | NGF, nerve growth factor, NGFB, beta-NGF, nerve-growth-factor, betaNGF, beta-nerve growth factor, nerve-growth factor, nerve growth-factor, Beta nerve growth factor, NGF human, nerve growth fac- tor, beta-nerve-growth-factor |
| DCTN6 | ENSP00000221114 | p27, p2-7, p2 7, dynactin 6, DCTN6, Dynactin Subunit 6 |
| SNRPE | ENSP00000400591 | BRAF, B-Raf, small nuclear ribonucleoprotein E, SNRPE, SmE, Sm-E, snRNP E, B RA F, small nuclear ribonucleoprotein polypeptide E |
| TMED7 | ENSP00000405926 | p27, p2-7, p2 7, TMED7 |
| PSMD9 | ENSP00000440485 | p27, p2-7, PSMD9, p2 7, Rpn4, proteasome 26S subunit, non-ATPase 9 |
| IFI27 | ENSP00000483430 | p27, ISG12, IFI27, p2-7, p2 7, Interferon alpha-inducible protein 27, interferon-alpha-inducible protein 27, ISG12A, Interferon-alpha-inducible-protein 27, interferon-alpha inducible protein 27 |
| ETS1 | ENSP00000376436 | Ets1, Ets-1, p54, EWSR2, cETS1, c-ets-1, ETS 1, ETS-protein, c-ets 1, ETS protein, c-Ets1, v-ets avian erythroblastosis virus E26 oncogene homolog 1, ETS proto-oncogene 1, transcription factor, avian erythroblastosis virus E26 oncogene homolog 1, v-ets avian erythroblastosis virus E26 oncogene homolog1 |
| RNASE3 | ENSP00000302324 | Raf-1, Raf1, eosinophil cationic protein, 2 kb 5, RNS3, ribonuclease 3, RNase 3, Raf- 1, RNASE3, eCp, Eosinophil-cationic protein |

| | | |
|---|---|---|
| MET | ENSP00000317272 | c-Met, MET proto-oncogene, HGFR, met protooncogene, hepatocyte growth factor receptor, meT, MET proto-oncogene, receptor tyrosine kinase, proto-oncogene c-met, hepatocyte growth-factor receptor, scatter factor receptor, HGF/SF receptor, protooncogene c-Met, cMET, HGF-R, c- MET, tyrosine-protein kinase Met, tyrosine-protein kinase-met, 4 DEG |
| SHBG | ENSP00000369816 | SHBG, sex hormone binding globulin, androgen-binding protein, TeBG, Sex hormone-binding globulin, sex steroid-binding protein, sex-hormone binding globulin, androgen binding protein, sex hormone- binding globulin, sex steroid binding protein, sex-hormone-binding globulin, Sex Hormonebinding Globulin |
| ATM | ENSP00000278616 | AT1, ATM, TEL1, ataxia-telangiectasia-mutated, ATDC, ataxia telangiectasia-mutated, AT-1, ataxia telangiectasia mutated, Hat1, ataxia-telangiectasia mutated, TEL1p, tel 1p, ATM serine/threonine kinase, AT 1, AT-C |
| CASP8 | ENSP00000351273 | caspase-8, 3H11, Caspase 8, CASP8, apoptotic cysteine protease, FLICE, Mch5, 4 PS1, death effector domain containing, FADD-homologous ICE/CED-3-like protease, CAP4, CASP-8, Mch-5, caspase- 8, Casp 8, Caspase8, CAS P8, FADD-like ICE, caspase-8, apoptosis-related cysteine peptidase |
| FASLG | ENSP00000356694 | FasL, CD95 ligand, CD95L, TNFSF6, Fas-ligand, tumor-necrosis factor superfamily, CD95-ligand, tumor necrosis factor superfamily, FASLG, CD178, Fas-L, FAS ligand, Fas L, CD95-L, fasligand, Fas -ligand, tumor necrosis factor super-family, tumor necrosis factor-superfamily, Fas ligand (TNF superfamily, member 6, tumor necrosis factor super family, CD95Ligand, APT1LG1 |
| NPC1L1 | ENSP00000289547 | NPC1L1, Niemann-Pick C1-Like protein 1, NPC1-like intracellular cholesterol transporter 1, hNPC1L1 |
| EIF2AK3 | ENSP00000307235 | pancreatic eIF-2alpha kinase, PERK, WRS, EIF2AK3, eukaryotic translation initiation factor 2-alpha kinase 3, p-ERK, eukaryotic translation initiation factor-2alpha kinase 3, pancreatic eIF2alpha kinase, PRKR-like endoplasmic reticulum kinase, eukaryotic translation initiation factor 2alpha kinase 3, eukaryotic translation initiation factor 2 alpha kinase 3 |
| GAST | ENSP00000331358 | gastrin, GAS, pre-pro gastrin, preprogastrin, prepro-gastrin, pre-pro-gastrin, Pre-progastrin |

| | | |
|---|---|---|
| SMAD3 | ENSP00000332973 | Smad3, Smad family, mothers against decapentaplegic homolog-3, Smad-3, SMAD, mothers against DPP homolog 3, Madh3, Smad 3, 1U7F, SMAD family member 3, Mothers against decapentaplegic homolog 3, mothers against decapentaplegic homolog3 |
| COL11A2 | ENSP00000363840 | PARP, COL11A2, Col11a-2, HKE5, collagen, type XI, alpha 2, collagen alpha-2(XI) chain |
| ATF2 | ENSP00000264110 | ATF-2, ATF2, Activating transcription factor 2, TREB7, CRE-BP1, CREB-2, CREB2, cAMP response element-binding protein CRE-BP1, activating transcription factor-2, CREBP1, activating transcription factor2 |
| NPM1 | ENSP00000296930 | NPM, nucleophosmin, Npm1, nucleophosmin 1, numatrin, nucleolar protein NO38, nucleolar phosphoprotein B23, nucleophosmin-1, nucleophosmin1, nucleophosmin (nucleolar phosphoprotein B23, numatrin, NP-M1, NPM-1 |
| PCSK9 | ENSP00000303208 | FH3, HFH-3, NARC-1, proprotein convertase subtilisin/kexin type 9, Pcsk9, neural apoptosis-regulated convertase 1, PC9, Hfh3, neural apoptosis regulated convertase 1, HCHOLA3, proprotein convertase subtilisin/kexin-type 9, PC-9, PCSK9 p, Neural Apoptosis-Regulated Convertase1, NARC1, proprotein convertase subtilisin/kexin type-9, Pc 9, PCSK-9, FH-3, hPCSK9, pro-protein convertase subtilisin/kexin type 9, Proprotein convertase-subtilisin/kexin type 9, 3 BPs, proprotein convertase subtilisin/kexin type9, Proprotein convertase subtilisin / Kexin type 9, PCSK 9, proprotein-convertase-subtilisin/kexin type 9 |
| HSP90AA1 | ENSP00000335153 | hsp90, LAP2, Hsp90a, hsp89, HSP90AA1, HSP-90, HSP89A, HSP 90, HSPCA, Hsp86, heat shock 90 kDa protein 1, alpha, hsp90N, 2 CCT, Hsp90 N, HSP90AA 1, heat shock protein HSP 90-alpha, HSPC1, LAP-2, 2c-CS, heat shock protein 90 alpha family class A member 1, 4 CWP, hHSP90 |
| HSPB1 | ENSP00000248553 | small heat-shock proteins, hsp27, small heat shock proteins, HSPB1, HSP25, HSP-27, heat shock 27 kD protein 1, HSP 27, heat shock 27kDa protein, P04792, sHSP, heat shock 27-kDa protein, HSP-28, heat shock 27-kDa protein 1, heat shock 27 kDa protein 1, hHsp27, heat-shock 27-kDa protein 1, heat shock protein beta-1, Hsp-25, heat-shock protein beta-1, heat shock protein beta 1, HSP28, heat shock protein family B (small) member 1, Heat shock protein family B member 1, srp 27 |

| | | |
|---|---|---|
| FXN | ENSP00000366482 | FRDA, frataxin, FXN, EC 1.16.3.1, CyaY, Friedreich ataxia protein, frataxin, mitochondrial |
| SREBF1 | ENSP00000348069 | SREBP-1a, SREBP1, SREBP-1, SREBP1c, SREBF1, SREBP-1c, sterol regulatory element-binding protein-1, Sterol regulatory element-binding protein 1, sterol regulatory element binding protein-1, Sterol Regulatory Element Binding Transcription Factor 1, SREBP1a, sterol regulatory element binding protein 1, sterol-regulatory-element-binding-protein-1, SREBF-1, sterol-regulatory element binding protein-1, SREBP-1a p, SREBP 1, SREBP1-c, sterol-regulatory-element-binding protein-1, sterol-regulatory-element-binding protein 1, sterol regulatory element-binding transcription factor 1, sterol regulatory element-binding protein1, sterol-regulatory element-binding transcription factor-1, sterol-regulatory element binding protein 1, sterol regulatory element-binding transcription factor-1, Sterol regulatory element binding protein1, sterol-regulatory element binding transcription factor-1, sterol regulatory element binding transcription factor1 |
| VIP | ENSP00000356213 | VIP, vasoactive intestinal peptide, PHM-27, prepro-VIP, Vasoactive-intestinal peptide, Vasoactive-intestinal-peptide, prepro VIP |
| CD40 | ENSP00000361359 | p50, CD40, Bp50, B-cell surface antigen CD40, CD40 molecule, hCD40, CDw40, P 5 0, CD40L receptor, tumor necrosis factor receptor superfamily member 5, TNFRSF5, CD-40, CD 40, B p50, HP 50 |
| RPS6KA3 | ENSP00000368884 | RPS6KA3, RSK2, RSK, Ribosomal Protein S6 Kinase, 90 kDa, Polypeptide 3, Cls, MRX19, ISPK-1, Rsk-2, Ribosomal S6 kinase 2, insulin-stimulated protein kinase 1, HU-3, hRSK2, ribosomal S6 kinase-2, Hu3, U08316, p90RSK2, ribosomal protein S6 kinase A3, ribosomal protein S6 kinase alpha-3 |
| SNAI1 | ENSP00000244050 | Snail, SnaH, SNAI1, SNAIL1, SNAI1P, Sna, snail-1, snai-1, zinc finger protein SNAI1, Snail family zinc finger 1, Snail 1, Snail family transcriptional repressor 1, Snail homolog 1 |

| | | |
|---|---|---|
| MDM2 | ENSP00000258149 | MDM2, HdmX, Hdm2, MDM-2, oncoprotein MDM2, hMDM2, MDM2 proto-oncogene, oncoprotein mdm-2, 2 MPS, p53-binding protein MDM2, MD M2, HDM-2, double-minute 2 protein, double minute-2 protein, double minute 2 protein, Mdm2 p53 binding protein homolog, MDM2 proto-oncogene, E3 ubiquitin protein ligase, E3 ubiquitin protein ligase MDM2, E3 ubiquitin-protein ligase Mdm2, MDM2-proto-oncogene, MDM 2, mdm-2 proto-oncogene |
| THBS1 | ENSP00000260356 | thrombospondin-1, TSP1, TSP-1, T-SP1, THBS1, thrombospondin 1, Thbs, HTSP1, THBS-1, hTSP-1, THBS 1, Thrombospondin1, Thrombo-Spondin 1 |
| TLR2 | ENSP00000260010 | TLR2, Toll-like receptor 2, TLR-2, toll-like receptor-2, TIL4, hTLR2, TLR 2, T IL-4, Tlr, Toll-like-receptor 2, T IL4, toll like receptors, toll-like receptor -2, Toll-like receptor2, toll like receptor 2, T IL-4, Toll Like Receptor-2, toll- like receptors, TLR- 2, Toll like receptor- 2, Toll-like-receptor-2, Toll-Like-Receptors, Tolllike receptor 2 |
| KCNH4 | ENSP00000264661 | Elk1, BEC2, Elk-1, hElk-1, KCNH4 |
| CDK20 | ENSP00000322343 | c-Crk, p42, cell cycle-related kinase, CCRK, cell-cycle-related kinase, CDK20, Cyclin-dependent kinase 20 |
| KCNH8 | ENSP00000328813 | Elk-1, ElK1, KCNH8, Elk3, hElk-1, Elk-3 |
| CASP9 | ENSP00000330237 | caspase-9, Casp9, caspase 9, Apaf-3, casp-9, caspase9, caspase- 9, caspase -9 |
| NUP43 | ENSP00000342262 | p42, Nup43 |
| CDKL1 | ENSP00000379176 | p42, KKIALRE, Cdkl1, Cyclin-dependent kinase-like1, Cyclin-dependent kinase-like 1, CDKL-1, cyclin dependent kinase like 1 |
| GPX1 | ENSP00000407375 | glutathione peroxidase 1, Selenoprotein S, cellular glutathione peroxidase, Gpx1, glutathione peroxidase-1, GPX-1, GSHPx-1, hGPX1, glutathione peroxidase1, selenoprotein-S, GP x 1, Gpx 1, glutathione peroxidase 1, Glutathione Peroxidase -1, GPX -1, GSHPX1 |
| PRKCA | ENSP00000408695 | PKC alpha, PKC-alpha, PRKCA, PKCalpha, PRKACA, protein kinase C-alpha, protein kinase Calpha, protein kinase C alpha, Pkca, protein kinase C, alpha, PKC-a |
| ALB | ENSP00000295897 | serum albumin, Alb, albumin, Hsa, Pro2619, 1-HK2 |
| HSPB2 | ENSP00000302476 | hsp27, small heat shock proteins, HspB2, MKBP, small heat-shock proteins, HSP-27, HSP 27, sHSP, hHsp27 |
| HSPB3 | ENSP00000303394 | small heat-shock proteins, hsp27, small heat shock proteins, HspB3, HspL27, HSP-27, protein-3, Protein 3, HSP 27, sHSP, hHsp27, protein3, Heat shock protein beta 3 |

| | | |
|---|---|---|
| GPX4 | ENSP00000346103 | snGPx, GPx4, glutathione peroxidase 4, phGPx, selenoprotein S, gpx-4, glutathione peroxidase-4, selenoprotein-S, 2 OBI, phospholipid hydroperoxidase, Gpx 4, MCSP |
| CDK4 | ENSP00000257904 | CDK4, Cdk-4, cyclin-dependent kinase 4, cyclin-dependent kinase-4, CDK 4, cyclin dependent kinase 4, cyclin-dependent kinase4, cyclin-dependent-kinase 4, cell division protein kinase 4, cyclin-dependent-kinase-4 |
| RPS6KB1 | ENSP00000225577 | S6K, S6K1, p70s6k, p70 alpha, p70alpha, RPS6KB1, PS6K, p70 S6K, p70-S6K, p-S6K, ribosomal protein S6 kinase, 70 kDa, polypeptide 1, S6K-1, ribosomal protein S6 kinase, 70kDa, polypeptide 1, ribosomal protein S6 kinase beta 1, ribosomal protein S6 kinase beta-1, ribosomal protein S6 kinase B1, p-70S6K |
| ARL6IP5 | ENSP00000273258 | GTRAP3-18, Hp 2-2, JWA, HP2-2, AF070523, PRAF3, Arl6-IP5, Hp22, ARL6IP5 |
| PHB2 | ENSP00000441875 | PHB2, Hp 2-2, repressor of estrogen receptor activity, prohibitin 2, HP2-2, hBAP, Bcap-37, Bcap37, Phb2p, bap, Hp22, Prohibitin-2 |
| ANXA5 | ENSP00000296511 | annexin V, annexin A5, PP4, ANX5, Annexin-V, ENX2, endonexin II, placental anticoagulant protein I, annexins, ANXA5, Annexin 5, vascular anticoagulant-alpha, lipocortin V, anchorin CII, annexinV, annexin-A5, 1-AVH, RPRGL3, AnnexinA5, calphobindin I |

| | | |
|---|---|---|
| MMP14 | ENSP00000308208 | MT1-MMP, MT-MMP, MMP-14, membrane-type 1 matrix-metalloproteinase, Membrane-type 1 matrix metalloproteinase, MMP14, membrane type-1 matrix metalloproteinase, MT-MMP-1, matrix metallopeptidase 14, membrane type matrix metalloproteinase-1, membrane-type 1-matrix metalloproteinase, membrane type 1-matrix metalloproteinase, membrane-type-1-matrix metalloproteinase, membrane-type-1 matrix metalloproteinase, membrane type 1 matrix metalloproteinase, membrane-type matrix metalloproteinase-1, MT1 MMP, membrane type-matrix metalloproteinase-1, matrix metalloproteinase 14, Membrane type 1 metalloprotease, matrix metalloproteinase-14, MT1 -MMP, MT-1 MMP, MT1MMP, MT1-MMP, membrane type-1 metalloprotease, membrane-type matrix metalloproteinase 1, membrane type matrix metalloproteinase 1, MMP 14, membrane type1 matrix metalloproteinase, matrix metallo-proteinase 14, MMP14 p, membrane type 1-metalloprotease, Membrane Type 1- Matrix Metalloproteinase, MTMMP |
| CHP1 | ENSP00000335632 | HP2-2, p24, CBLP, CHP1, Hp 2-2, calcineurin homologous protein, calcineurin-like EF hand protein 1, calcineurin B-like protein, calcineurin b homologous protein 1, P24P, Hp22, P2 4, CHP-1, calcineurin-like EF-hand protein 1, HP24 |
| DYNC1H1 | ENSP00000348965 | cytoplasmic dynein heavy chain 1, DYNC1H1, DHC1a, HP2-2, Hp 2-2, DNCHC1, DNECL, cytoplasmic dynein 1 heavy chain 1, Hp22, dynein,  cytoplasmic 1,  heavy chain 1, Dynein Cytoplasmic 1 Heavy Chain 1, CMT2O |
| DNMT1 | ENSP00000352516 | DNMT1, DNA methyltransferase 1, DNA-methyltransferase 1, DNMT, DNA methyltransferase-1, DNA (cytosine-5-)-methyltransferase 1, hDNMT1, DNA (cytosine-5)-methyltransferase 1, EC 2.1.1.37, AIm, dnmt-1, DNMT 1, DNA-methyltransferase-1, DNA methyltransferase1, DNA methyl transferase 1, DNA methyl-transferase 1, DNA methyl-transferase-1, DNA-methyltransferase1, DNA (cytosine-5) methyltransferase 1, DNA-Methyl-Transferase 1 |

| | | |
|---|---|---|
| BCL2L11 | ENSP00000376943 | Bim, BimS, Bcl2l11, BimEL, Bcl-2-interacting mediator of cell death, BCL-2 interacting mediator of cell death, BimL, Bim-EL, BCL2-like 11, Bcl-2-like protein 11, BCL2-like11, Bcl-2 like 11, Bcl2-interacting mediator of cell death, bcl-2-like 11, Bcl-2 L11, bam, BCL2-L11, Bcl-2 like protein 11, BCL2 interacting mediator of cell death, Bcl2 l11 |
| FOXO3 | ENSP00000385824 | FKHRL1, FKHR-L1, FOXO3a, FoxO3, AF6q21, forkhead box O3, FOXO2, FKHRL-1, Foxo3P, Forkhead Box Protein O3, hFOXO3, forkhead-box O3, forkhead box O 3, FOXO-3a |
| SOD2 | ENSP00000446252 | EC 1.15.1.1, MnSOD, SOD2, superoxide dismutase 2, superoxide dismutase-2, SOD-2, 2 ADP, IPO-B, Mn-SOD, 2 gDs, Superoxide dismutase2, super oxide dismutase-2, superoxide dismutase 2, mitochondrial, hSOD2, Mn SOD |
| PGF | ENSP00000451040 | PIG-F, PlGF, placental growth factor, placenta growth factor, PIGF, VEGF family, PlGF-2, P-GF, VEGF-family, p-IGF, Pgf, PLGF2 |
| TNFSF10 | ENSP00000241261 | Apo2L, tumor-necrosis factor superfamily, Tnfsf10, Apo-2L, TL2, TNF-related apoptosis-inducing ligand, Apo2 ligand, tumor necrosis factor superfamily, Apo-2 ligand, TNF-related apoptosis inducing ligand, Apo2-L, TNF-related apoptosis -inducing Ligand, TNF-related apoptosis-inducing-ligand, TNF Related Apoptosis Inducing Ligand, tumor necrosis factor super-family, tumor necrosis factor super family, tumor necrosis factor-superfamily, tumor necrosis factor superfamily,  member 10, Apo 2 ligand, CD253, TNF-related apoptosis inducing-ligand, TNF-related apoptosisinducing ligand, TNF related apoptosis inducing-ligand, TL-2, tumor necrosis factor ligand superfamily member 10, TNF related apoptosis-inducing ligand, Apo-2-ligand, TNF-related-apoptosis-inducing-ligand |
| CCR7 | ENSP00000246657 | Evi-1, CCR7, EVI1, chemokine receptor 7, EBI 1, EBI1, EBI-1, CCR-7, Cmkbr7, MIP-3 beta receptor, C-C chemokine receptor type 7, C-C motif chemokine receptor 7, Chemokine (C-C motif) receptor 7, CD197, Chemokine receptor-7, EVI 1, C-C motif chemokine receptors, CC chemokine receptor type 7 |

| | | |
|---|---|---|
| VCAM1 | ENSP00000294728 | L1CAM, vascular cell adhesion molecule-1, VCAM1, VCAM-1, vascular cell adhesion molecule 1, L1-CAM, L1 CAM, CD106, CD 106, vascular cell-adhesion molecule 1, vascular cell-adhesion molecule-1, hL1CAM, vascular cell adhesion protein-1, Vascular cell adhesion protein 1, VCAM- 1, V-CAM-1, V-CAM1, VCAM 1 |
| MAPK7 | ENSP00000311005 | PRKM7, Erk5, BMK1, extracellular signal-regulated kinase 5, extracellular-signal regulated kinase 5, mitogen activated protein kinase 7, MAPK7, extracellular signal-regulated kinase-5, ERK-5, extracellular signal regulated kinase 5, extracellular signal regulated kinase-5, Extracellular-signal-regulated kinase 5, extracellular-signal-regulated kinase-5, Mitogen-activated protein kinase 7, ERK 5, Extra-cellular signal regulated kinase-5 |
| FGF13 | ENSP00000322390 | Fibroblast growth factor homologous factor 2, FGF2, FGF-2, Fgf13, FHF2, Fibroblast Growth Factor 13, hFGF2, FGF-13, FGF 2 |
| MAPT | ENSP00000340820 | Tau, MAPT, microtubule-associated protein tau, microtubule associated protein tau, MTBT1, Paired helical filament tau, paired helical filament-tau, MTBT2, microtubule-associated-protein-tau, Microtubule-associated protein-tau, microtubuleassociated protein tau, P10636 |
| XIAP | ENSP00000360242 | X-linked inhibitor of apoptosis protein, XIAP, X-linked inhibitor of apoptosis, hILP, BIRC4, X-linked inhibitor-of-apoptosis protein, X-linked inhibitor-of-apoptosis, X-IAP, MiHA, Baculoviral IAP repeat-containing-4, baculoviral IAP repeat-containing protein-4, baculoviral IAP repeat-containing protein 4, X linked inhibitor-of-apoptosis protein, E3 ubiquitin-protein ligase XIAP |
| STAT1 | ENSP00000354394 | STAT-1, STAT1, ISGF3, ISGF-3, signal transducer and activator of transcription-1, signal transducer and activator of transcription 1, ISG F3, Stat 1, STAT1-P, signal transducer and activator of transcription1, STAT91 |
| BECN1 | ENSP00000355231 | beclin 1, Beclin1, Beclin-1, Becn1, vps30, Atg6, Atg-6, beclin 1, autophagy related, becn-1, Beclin-1, Autophagy-related |

| | | |
|---|---|---|
| BMP2 | ENSP00000368104 | BMP2, bone morphogenetic proteins, BMP-2, BMP2A, BMP-2A, bone morphogenetic protein-2, bone morphogenetic protein 2A, bone morphogenetic protein 2, BMP 2, bone-morphogenetic protein 2A, 3 BMP, bone morphogenetic proteins, bone morphogenetic protein 2, HGNC:1069, bonemorphogenetic protein-2, Bmp |
| PRKCD | ENSP00000378217 | Protein kinase Cdelta, PKC-delta, PKCdelta, protein kinase C delta, PKC delta, PRKCD, Pkcd, protein kinase C-delta, protein kinase C delta type, PKC-D |
| PDZD2 | ENSP00000402033 | Pin1, PAPIN, PDZD2, PDZ domain containing 2, hPin1, PDZ-domain-containing 2, Pin-1, PIN 1, PDZK3, PDZ domain containing protein 3, PDZ domain-containing protein 2, PDZ domain-containing protein 3, PDZ domain containing 3 |
| RPS6KA1 | ENSP00000435412 | Rsk, p90rsk, HU-1, RPS6KA1, Hu1, RSK1, p90 rsk, p90-RSK, RSK-1, Ribosomal S6 kinase 1, ribosomal protein S6 kinase A1, ribosomal S6 kinase1, 2 Wnt, 2-Wnt, 90 kDa ribosomal protein S6 kinase 1 |
| SEC14L2 | ENSP00000478755 | TAP1, SEC14L2, alpha-tocopherol associated protein, hTAP1, Spf, alpha-tocopherol-associated protein, Supernatant protein factor, TAP-1, TAP 1, alpha tocopherol-associated protein |
| GLI1 | ENSP00000228682 | Gli, GLI-1, Gli1, oncogene GLI, glioma-associated oncogene family zinc finger-1, glioma-associated-oncogene, glioma-associated oncogene homolog 1, Glioma-associated oncogene, glioma-associated oncogene homolog1, Gli 1, GLI family zinc finger 1, Glioma-associated oncogene homolog-1, glioma-associated oncogene family zinc finger 1, Zinc Finger protein GLI1 |
| PIN1 | ENSP00000247970 | Pin1, UBL5, hPin1, peptidyl-prolyl cis-trans isomerase Pin1, Pin-1, PIN 1, 2 XPA, Peptidyl-prolyl cis-trans isomerase NIMA-interacting 1, Peptidylprolyl cis/trans isomerase, NIMA-interacting 1 |
| KCNH2 | ENSP00000262186 | HERG, Kv11.1, ERG1, KCNH2, erg, hERG1, erg 1, H-erg, SQT1, U04270, ERG-1, KCNH2-p, ER-G1, potassium voltage-gated channel subfamily H member 2, KCNH2 p, Kv 11.1 |

| | | |
|---|---|---|
| NFKBIA | ENSP00000216797 | IkappaBalpha, ikba, I kappa B alpha, IkappaB-alpha, mad3, NFKBIA, hMAD-3, IkappaB alpha, MAD-3, I kappa B-alpha, I kappaB alpha, NFKBI, I-kappaBalpha, I kappa B- alpha, I-kappa B alpha, Ikappa Balpha, Ikappa-Balpha, I-kappa B-alpha, NFkappaB inhibitor alpha, I-kappaB alpha, I-kBa, NF-kappaB inhibitor alpha, I-kappaB-alpha, nuclear factor of kappa light polypeptide gene enhancer in B-cells inhibitor, alpha, NF-kB Inhibitor alpha, NF-kBIA, I-kappa-B-alpha, NF-kappa-B inhibitor alpha, NFKB inhibitor alpha |
| AXL | ENSP00000301178 | Axl, Axl receptor tyrosine kinase, ARK, axl oncogene |
| IL13 | ENSP00000304915 | IL-13, IL13, p600, interleukin 13, interleukin-13, hIL-13, Bhr1, hIL13, Interleukin13, IL13 p, IL 13, 5L6Y, IL- 13 |
| CYP3A4 | ENSP00000337915 | CYP3A4, cytochrome P450 3A4, CYP3A, HLp, P450PCN1, cholesterol 25-hydroxylase, cytochrome P450HLp, Cytochrome P-450 3A4, cholesterol-25-hydroxylase, nifedipine oxidase, CYP3A3, NF25, cytochrome P-450 PCN1, cytochrome P4503A4, cytochrome P450pcn1, CYP 3A4, CP33, hCYP3A4, Cholesterol 25 hydroxylase, NF2 5, cytochrome P450, subfamily IIIA, polypeptide 4, AF209389, cytochrome P450-3A4, CYP 3A, CYP-3A4, cytochrome P450 family 3, CYP3 A4, cytochrome P450 family 3 subfamily A member 4 |
| POMC | ENSP00000384092 | POMC, beta-endorphin, ACTH, proopiomelanocortin, alpha-melanocyte-stimulating hormone, adrenocorticotropic hormone, pro-opiomelanocortin, CLIP, adrenocorticotropin, pro-opio-melanocortin, alpha-melanocyte stimulating hormone, beta-melanocyte stimulating hormone, beta-melanocyte-stimulating hormone, beta-lipotropin, adrenocortico tropic hormone, msh, alpha-melanocyte--stimulating hormone, alpha melanocyte stimulating hormone, Alpha melanocyte-stimulating hormone, Beta endorphin, npp, NP-P, adreno corticotropic hormone, pOC |
| GDF2 | ENSP00000463051 | BMP-9, BMP9, Bone morphogenetic protein 9, Bone morphogenetic protein-9, GDF2, growth differentiation factor 2, Bone morphogenetic protein9 |
| HSPA1A | ENSP00000364802 | hsp72, HSP 72, HSP70-2, HSP70-1, HSPA1B, heat shock 70 kDa protein 1A, HSPA1A, Hsp-72, HSP70.2, heat shock 70 kDa protein 1, 5 BPM, heat shock 70 kDa proteins, HSPA1, HSP70-1B, HSP 70-1, h-Hsp72, Hsp 70-2, Hsp-70 1B, 3 LoF, Heat shock protein family A member 1A, hsx70 |

| | | |
|---|---|---|
| ALK | ENSP00000373700 | ALK, anaplastic lymphoma kinase, ALK receptor tyrosine kinase, anaplastic-lymphoma-kinase, ALK receptor tyrosine-kinase, CD246, anaplastic lymphoma receptor tyrosine kinase, ALK tyrosine kinase receptor, anaplastic-lymphoma kinase, anaplastic lymphomakinase, 3 LCS, anaplasticlymphomakinase, anaplasticlymphoma kinase |
| CLU | ENSP00000315130 | clusterin, apolipoprotein J, TRPM2, APOJ, SGP-2, CLU, SP-40, testosterone-repressed prostate message 2, TRPM-2, sulfated glycoprotein-2, Sulfated glycoprotein 2, apo J, complement lysis inhibitor, complement-lysis inhibitor, Testosterone-repressed prostate message-2, NA1/NA2, APO-J, SGP2, CLU1, CLU2, TRPM 2, CL-I, CLU 1, CLU 2, Apolipoprotein-J |
| SLC2A4 | ENSP00000320935 | GLUT4, GLUT-4, SLC2A4, GLUT 4, hGLUT-4, GLUT4-P, solute carrier family 2 member 4, Solute carrier family-2-member-4, solute carrier family 2 (facilitated glucose transporter), member 4, solute carrier family 2, facilitated glucose transporter member 4, hGLUT4 |
| CYP17A1 | ENSP00000358903 | P450c17, CYP17, cytochrome P450c17, CYP17A1, P450 c17, steroid 17alpha-hydroxylase/17, 20 lyase, steroid 17 alpha-monooxygenase, CYP 17, P-450C17, steroid 17 alpha-hydroxylase/17, 20 lyase, Cytochrome P450c 17, cytochrome P-450c17, hCYP17, CYP17 A1, cytochrome P450 17A1, steroid 17alpha-hydroxylase/17, 20-lyase, P450c 17, cytochrome P450, family 17, subfamily a, polypeptide 1, CYP17-A1, Cytochrome P450-C17, CYP-17, cytochrome P450 family 17 subfamily A member 1, CYP 17A1, cytochrome P450 family 17 |
| WNT5A | ENSP00000264634 | Wnt family, Wnt5a, Wnt-5a, Wnt5a protein, Wnt 5A, Wnt-5a protein, Wnt-family, wingless-type MMTV integration site family, member 5A, Wnt5-a, protein WNT5A, Wnt family member 5a, hWnt5a |
| MIR215P | hsa-miR-21-5p | mir-21, hsa-mir-21, MIR21, miR 21, miR-21-5p, hsa-miR-21-5p, miR-21, MiR 21-5p, hsa-miR21 |
| MIR34A5P | hsa-miR-34a-5p | miR-34a, miR34a, hsa-miR-34a, miR-34a-5p, miR34A-5p, hsa-miR34a, MiR- 34a, hsa-miR-34a-5p, mi-R34a |

| | | |
|---|---|---|
| NTRK1 | ENSP00000431418 | TRKA, tyrosine kinase receptor, NTRK1, Trk, TRK-A, gp140trk, mtC, high-affinity nerve growth factor receptor, trk1, TRK A, neurotrophic tyrosine kinase receptor type 1, tyrosine kinase-receptor, p140trka, tyrosine-kinase receptor, tyrosinekinase receptor, tyrosine kinase receptor A, p140 TrkA, tyrosine-kinase-receptor, high affinity nerve growth factor receptor, tropomyosin-related kinase A, neurotrophic tyrosine kinase, receptor, type 1, p-140 TrkA, tropomyosin related kinase A, 4 PMs, neurotrophic tyrosine kinase receptor type 1, neurotrophic receptor tyrosine kinase 1, 1-shC, tropomyosin-related kinase-A, Trk- A, p140trk-A |
| PGPEP1 | ENSP00000269919 | Pyroglutamyl peptidase I, pcP, Pyroglutamyl-peptidase I, Pg I, P-gp, PGI, EC 3.4.19.3, AJ278828, pgp, PGPEP1, PC p, Pg-I, pyroglutamyl-aminopeptidase |
| GJA1 | ENSP00000282561 | Cx43, connexin43, GJA1, GJA 1, Connexin 43, Gja-1, cx 43, connexin-43, gap junction protein alpha-1, gap junction protein alpha 1, GJA1P, Cx-43, hCx43, Gap junction protein-alpha 1, C x 43, Gap Junction Protein Alpha1, PPKCA, Cx43 p, EKV-P, gap junction protein, alpha 1, 43 kDa, cMDR, Gjal |
| F2RL1 | ENSP00000296677 | PAR-2, proteinase-activated receptor-2, PAR2, proteinase activated receptor-2, proteinase-activated receptor 2, F2rl1, proteinase activated receptor 2, PAR 2, hPar2, coagulation factor II receptor-like 1, Proteinase activated-receptor 2, proteinaseactivated receptor-2 |
| PSEN1 | ENSP00000326366 | presenilin-1, PSEN1, PS1, PS-1, presenilin 1, HPS1, HPS-1, Ps 1, AD3, PS -1, presenilin1, PSEN-1, pre-senilin 1, Ad3p, PSEN1 p, Presenilin -1, S18-2, AD-3, Hp-s1, PSEN 1, PSNL1 |
| PGP | ENSP00000330918 | phosphoglycolate phosphatase, P-gp, EC 3.1.3.18, pgp, Glycerol-3-phosphate phosphatase, G3PP |
| CTNNB1 | ENSP00000344456 | CTNNB1, beta-catenin, 1p22, beta catenin, beta -catenin, 1----p22, betacatenin, beta-catenins, beta- catenin, CTNNB 1, CTNNB-1, catenin beta1, 1 p22, CTNNB, catenin beta 1, CTNNB1 p, catenin (cadherin-associated protein), beta 1, 88kDa, catenin (cadherin-associated protein), beta 1, 88 kDa, catenin beta-1, Catenin-beta-1 |
| PON1 | ENSP00000222381 | PON1, PON, paraoxonases, paraoxonase 1, Paraoxonase-1, esterase-A, esterase A, EC 3.1.1.2, arylesterase 1, Paraoxonase1, PON 1, PON-1, EsA, hPON1, EC 3.1.8.1, serum paraoxonase/arylesterase 1, PON1 p |

| | | |
|---|---|---|
| SCGB2A2 | ENSP00000227918 | HMGB1, mammaglobin-1, HMGB 1, MGB1, HMGB-1, SCGB2A2, mammaglobin-A, secretoglobin family 2A member 2, Mammaglobin A, mammaglobin 1 |
| HBEGF | ENSP00000230990 | HB-EGF, heparin-binding epidermal growth factor, Hbegf, diphtheria toxin receptor, heparin-binding EGF-like growth factor, D-T-S, D-T-R, DTR, Hegfl, Heparin-binding epidermal-growth-factor, heparin binding epidermal growth factor, heparin binding EGF-like growth factor, heparin binding EGF like growth factor, heparin binding-EGF-like growth factor, heparin binding-epidermal growth factor, heparin- binding epidermal growth factor, heparin-binding EGF like growth factor |
| DUSP1 | ENSP00000239223 | MKP-1, VH1, Dusp1, VH 1, CL100, VH-1, dual-specificity phosphatase-1, MKP1, dual specificity phosphatase 1, mitogen-activated protein kinase phosphatase-1, mitogen-activated protein kinase phosphatase 1, hVH-1, V-H1, mitogen activated protein kinase phosphatase-1, Dual-specificity phosphatase 1, HVH1, PTPN10, Dual specificity protein phosphatase 1, Dual-specificity protein phosphatase 1, DUSP-1, dual specificity phosphatase-1, dual-specificity protein phosphatase1, dual-specificity protein phosphatase-1 |
| CCL21 | ENSP00000259607 | Exodus-2, CCL21, SCYA21, secondary lymphoid tissue chemokine, 6Ckine, secondary lymphoid-tissue chemokine, TCA4, CCL-21, Exodus2, slc, CCL 21, chemokine ligand 21, chemokine (C-C motif) ligand 21, C-C motif chemokine ligand 21, hCCL21 |
| GDE1 | ENSP00000261386 | MIR16, miR-16, Membrane Interacting protein of RGS16, miR- 16, miR 16 |
| FOXO1 | ENSP00000368880 | FKHR, Foxo1, forkhead box O1, FOXO1A, 3 COA, Foxo-1, forkhead in rhabdomyosarcoma, forkhead homolog in rhabdomyosarcoma, forkhead box protein O1, Forkhead boxO1, forkhead box protein O 1, FOXO 1, Forkhead box O 1, fork head box O 1, Forkhead box-O1, 3coa, fork head box protein O1 |

| | | |
|---|---|---|
| ABCC1 | ENSP00000382342 | MRP1, ABCC1, multidrug resistance-associated protein 1, multidrug resistance-associated protein-1, Mrp, ABCC, leukotriene C(4) transporter, hMRP1, ABC-C, MRP-1, multidrug resistance associated protein 1, multidrug resistance associated protein-1, MRP1 p, ATP-binding cassette, subfamily C, member 1, ATP binding cassette sub-family C, A-B-C-C, MR p, ATP-binding cassette sub-family C member 1, Gsx, ATP-binding cassette, sub-family C, member 1, MRP 1, multidrug-resistance-associated protein 1, multidrug-resistance-associated protein-1, ATP-binding cassette sub-family C, multidrug resistance associated protein1, ATP binding cassette subfamily C member 1, ATP binding cassette subfamily C, 2CBZ, ATP-binding cassette subfamily C member 1, hABCC1 |
| PRAP1 | ENSP00000416126 | uPA, u-PA, Proline-rich acidic protein 1, PRAP1, up-a |
| CDKN2A | ENSP00000418915 | ARF, CDKN2A, p16, INK4a, P16INK4A, p14, p19ARF, p14ARF, INK4, CDKN2, CDK4I, mts1, p16INK4, Ink4-a, MTS-1, cyclin-dependent kinase inhibitor 2a, multiple tumor suppressor 1, cyclin dependent kinase inhibitor 2A, CMM2, p16 INK4A, cyclin-dependent kinase inhibitor-2A, p16 INK4, p-16, p1 6, INK 4A, p14 ARF, TP16, p16-INK4A, p14-ARF, p 16, p19 ARF, p16-INK4, cyclin-dependent kinase 4 inhibitor A, HP-14, H-MTS1, CDKN2A p, MTs 1, p 14, HP14, p19-ARF, TP-16, multiple tumor suppressor-1, P 16ink4, CDKN 2, hp19ARF |
| MIR165P | hsa-miR-16-5p | MIR16, miR-16, hsa-miR-16, miR- 16, miR-16-5p, hsa-miR-16-5p, miR-16- 5p, miR 16, hsamiR-16-5p, miR- 16-5p |
| HESX1 | ENSP00000295934 | Hesx1, Rpx, ANF, HESX-1, HA-NF |
| ACHE | ENSP00000303211 | acetylcholinesterase, EC 3.1.1.7, acetyl-cholinesterase, acetyl cholinesterase, 1B41, N-AChE, EC3.1.1.7, 4M0E, acetylcholine sterase |
| BAX | ENSP00000293288 | Bcl2-associated X protein, Bcl-2-family, bcl-2 family, Bcl-2-associated X protein, Bcl2 family, Bcl-2 associated X protein, BCL2-family, Apoptosis regulator BAX, Bcl2 associated X protein, Bcl 2-family, Bcl2-like protein 4, Bcl-2 associated X, apoptosis regulator, Bcl-2-associated X, apoptosis regulator, BCL2 associated X, apoptosis regulator, Bcl-2-like protein 4, Bcl 2 associated x protein, Bcl-2-associated-X protein, BCL2-associated X, apoptosis regulator, Bcl-2-associated X-protein |

| | | |
|---|---|---|
| CCL11 | ENSP00000302234 | eotaxin, CCL11, Eotaxin1, eotaxin-1, SCYA11, Eotaxin 1, eosinophil chemotactic protein, chemokine (C-C motif) ligand 11, CCL 11, 2 MPM, CCL-11, C-C motif chemokine 11, C-C motif chemokine ligand 11, C-C motif chemokine11, chemokine ligand 11 |
| ATF3 | ENSP00000344352 | Activating transcription factor 3, ATF3, ATF-3, activating transcription factor-3, ATF3-008 |
| GLP1R | ENSP00000362353 | glp1, GLP-1R, GLP-1, Glp1r, Glucagon-like peptide-1 receptor, glucagon receptor family, glucagon-like peptide 1 receptor, Glucagonlike peptide-1 receptor, glucagon like peptide-1 receptor, hGLP-1R, GLP-1 R, GLP1-R, glucagon like peptide 1 receptor, glucagon -like peptide-1 receptor, Glucagon like-peptide 1 receptor, glucagon-like-peptide-1 receptor, glucagonlike peptide 1 receptor, GLP 1, GLP -1, GLP -1R, glucagon-like peptide 1receptor, glucagon-like-peptide 1 receptor, GLP-1-R |
| NPPA | ENSP00000365663 | Nppa, ANP, ANF, cDD, natriuretic peptide precursor A, natriuretic peptide A, Natriuretic Peptides-A, natriuretic peptides A |
| KLHL1 | ENSP00000367075 | KLHL1, MRP-2, Mrp2, Kelch-like 1, Kelch-like protein 1, MAYVEN-related protein 2, Mrp 2 |
| SYCE1L | ENSP00000367911 | Mrp2, MRP-2, Mrp 2 |
| PRNP | ENSP00000368752 | prion protein, PRNP, PrP, PRIP, PrP27-30, prion-protein, PrPC, PrP 27-30, PrP C, gss, pr p, 3 HER, AScr, Prn-p, major prion protein, 3 HES, PrP27 - 30, 1E1P, 1E1U, 2K1D |
| CASP1 | ENSP00000433138 | caspase-1, Caspase 1, casp1, interleukin-1 beta converting enzyme, IL1BC, interleukin 1 beta-converting enzyme, p45, interleukin-1 beta-converting enzyme, Interleukin-1beta Converting Enzyme, IL-1B C, interleukin 1beta converting enzyme, interleukin-1beta-converting enzyme, interleukin- 1beta-converting enzyme, caspase-1, interleukin 1beta-converting enzyme, interleukin-1-beta converting enzyme, interleukin-1-beta-converting enzyme, caspase1, IL1B C, CASP-1, interleukin-1betaconverting enzyme, interleukin 1 beta converting enzyme |
| CLDN2 | ENSP00000441283 | Claudin-2, Cldn2, claudin 2, CLDN-2, HSP82, CLDN 2, claudin2 |
| TTR | ENSP00000237014 | TTR, Transthyretin, Palb, prealbumin, TbpA, ATTR, CTS1, 4 act, 3-Neo, 2B15, T TR, CTS-1, 4 TNF, 3neo, 3cN0, pre-Albumin, 1 Tyr, AT-TR, 3 Neo, P-Alb, CT-S |

| | | |
|---|---|---|
| ADAM10 | ENSP00000260408 | ADAM 10, ADAM10, RAK, MADM, KUZ, ADAM-10, AD10, Ad18, disintegrin and metalloproteinase domain-containing protein 10, ADAM metallopeptidase domain 10 |
| TIMP2 | ENSP00000262768 | TIMP-2, Tissue inhibitor of metalloproteinases-2, TIMP2, tissue inhibitor of metalloproteinase 2, Tissue inhibitor of metalloproteinases 2, CSC-21K, hTIMP-2, tissue inhibitor of metalloproteinase-2, TIMP 2, tissue inhibitor of metalloproteinase2, tissue-Inhibitor of Metalloproteinase-2, TIMP-2, Tissue inhibitor of metallopeptidases, TIMP metallopeptidase inhibitor 2, metalloproteinase inhibitor-2, metalloproteinase inhibitor 2, TIMP metallopeptidase inhibitor-2 |
| CD274 | ENSP00000370989 | B7-H1, PD-L1, PDL1, CD274, B7H, Programmed cell death 1 ligand 1, B7-homolog 1, B7 homolog 1, PDL-1, B7H1, Programmed cell Death-1 Ligand 1, B7-H, programmed cell death-1 ligand-1, hPD-L1, PD--L1, programmed cell death 1 ligand-1, 3FN3, programmed cell death 1-Ligand 1, CD274 molecule, PD -L1, CD 274, PD L1, programmed cell-death 1 ligand 1, CD-274, PDCD1LG1 |
| PROM1 | ENSP00000426809 | AC133, CD133, Prominin-1, PROM1, Stargardt disease-4, STGD4, Prominin 1, MCDR2, prominin-like protein 1, PROML1, Prom-1, hCD133, Prominin1, CD 133, CD-133, PROM1 p |
| DNM1L | ENSP00000449089 | DLP1, DRP-1, dynamin-like protein, DRP1, DNM1L, Vps1p, dymple, HdynIV, Vps1, dynamin-related protein 1, DVLP, Dnm1p/Vps1p-like protein, hDLP1, dynamin-related protein-1, dynamin-like protein IV, dynamin 1-like, vps 1, dynamin related protein 1, dynamin-1-like protein, Dynamin-related protein1, dynamin 1 like protein, dynamin related protein-1, DRP 1, dynamin 1 like, Dynamin-Related-Protein 1, dynamin-1 like, dynamin 1-like protein |

| | | |
|---|---|---|
| ACE | ENSP00000290866 | angiotensin-converting enzyme, ACE, Angiotensin I-converting enzyme, DCP1, ACE1, HACE1, Angiotensin converting enzyme, angiotensin-converting-enzyme, angiotensin I converting enzyme, Angiotensin-I converting enzyme, angiotensin I--converting enzyme, peptidyl-dipeptidase A, EC 3.4.15.1, kininase II, hDcp1, peptidyl dipeptidase A, angiotensin-I-converting enzyme, CD143, angiotensin- converting enzyme, Angiotensinconverting enzyme, DCP-1, ich, ACE-1, angiotensin I-converting-enzyme, CD 143, angiotensin I- converting enzyme, angiotensin I converting enzyme 1, Angiotensin converting-enzyme, angiotensinconverting- enzyme, Angiotensin -converting enzyme, 2XY9, 3NXQ, Angiotensin-convertingenzyme, 4-APH, dipeptidyl-carboxypeptidase I, dipeptidyl carboxypeptidase-I |
| MGMT | ENSP00000302111 | MGMT, O6-methylguanine-DNA methyltransferase, EC 2.1.1.63, O6-methylguanine-DNA-methyltransferase, O-6-methylguanine-DNA methyltransferase, O6-methylguanine DNA methyltransferase, O6-methyl-guanine-DNA methyltransferase, O-6-methylguanine DNA methyltransferase, O6-methylguanine DNA-methyltransferase, O 6 -methylguanine-DNA methyltransferase, O6 methylguanine DNA methyl-transferase, O6 -methylguanine-DNA methyltransferase, O6 methylguanine DNA methyltransferase, O6-methylguanine-DNA methyl-transferase, O6-methyl guanine DNA methyl transferase, O6 methylguanine-DNA methyltransferase, O-6-methylguanine-DNA-methyltransferase, O6-methylguanine-DNA methyl transferase, O6-Methyl Guanine DNA Methyltransferase, O-6-methylguanine-DNA methyltransferase, O6-methylguanine-DNA-methyl transferase, O-6-methylguanine DNA-methyltransferase, O-6 methylguanine- DNA methyltransferase, O6-methylguanine-DNAmethyltransferase, O 6-methylguanine DNA methyltransferase, O6 methylguanine-DNA-methyltransferase, O6-methyl-guanine-DNA-methyltransferase, O6-methylguanine- DNA methyltransferase, O6 -methylguanine DNA methyltransferase, O6 -methylguanine DNA-methyltransferase, O 6-methylguanine-DNA methyltransferase, O-6-methyl guanine DNA methyltransferase |
| FEN1 | ENSP00000305480 | flap endonuclease-1, FEN-1, FEN1, RAD2, FEN 1, Mf1, Mf-1, maturation factor-1, Flap endonuclease 1, DNase IV, flap structure-specific endonuclease 1 |

| | | |
|---|---|---|
| EZH2 | ENSP00000320147 | Ezh2, Enhancer of Zeste homolog 2, Polycomb repressive complex 2, EZH1, Enx1, histone-lysine N-methyltransferase EZH2, EZH 2, Polycomb-Repressive Complex 2, polycomb repressive complex-2, enhancer of Zeste homolog-2, enhancer-of-zeste homolog 2, enhancer of zeste homolog2, enhancer of zeste 2 polycomb repressive complex 2 subunit, enhancer of zeste-homolog 2, KMT6A, KMT6, Polycomb Repressive Complex2, polycomb-repressive complex2, polycombrepressive complex 2, EZH2 p |
| GTF2H2 | ENSP00000328901 | TFIIH, p44, BTF2p44, BTF2, GTF2H2, 5 of 4, p 44, p-44 |
| SGK1 | ENSP00000356832 | SGK, SGK1, sgk-1, serum/glucocorticoid regulated kinase, serum/glucocorticoid-regulated kinase 1, h-SGK1, hSGK1, serum/glucocorticoid regulated kinase-1, serum/glucocorticoid-regulated kinase, serum/glucocorticoid regulated kinase 1, serum/glucocorticoid-regulated kinase-1 |
| IFI44 | ENSP00000359783 | p44, p 44, IFI44, Interferon-induced protein 44, p-44 |
| OSM | ENSP00000215781 | oncostatin M, osm, oncostatin-M, oncostatinM, OS-M |
| IFNG | ENSP00000229135 | interferon-gamma, IFNG, interferon gamma, interferongamma, immune interferon, Ifi, Ifg, IFN-g, interferon- gamma, interferon-g, inter-feron-gamma, IFN- g |
| ODC1 | ENSP00000234111 | ornithine decarboxylase, ODC1, ODC, EC 4.1.1.17, ornithine decarboxylase 1, ornithine decarboxylase-1, Odc1p |
| WDR77 | ENSP00000235090 | p44, MEP50, WD repeat domain 77, WD45, WDR77, methylosome protein 50, p44/Mep50, androgen receptor cofactor p44, p 44, WDR7-7, p-44 |
| APOA1 | ENSP00000236850 | apo AI, apoA-I, apo(a, apoAI, apolipoprotein AI, apolipoprotein A1, apo A-I, apolipoprotein A-I, APOA1, Apo-A1, apo-A-I, apo A-1, apolipoprotein A-1, APO A1, apo-AI, 1AV1, apolipoprotein A I, apoA-1, 2 MSC, ApolipoproteinA1, apolipoprotein-A1, Apo lipoprotein A-I, Apo lipoprotein A1, apolipoprotein-AI, apoA I, ApolipoproteinA-I, Apo (a |
| GRP | ENSP00000256857 | gastrin-releasing peptide, GRP, bombesin, prepro-GRP, GR-P, pro-GRP, neuromedin C, Gastrin releasing peptide, ProGRP, preproGRP, pre-pro-GRP, Pre-proGRP, GR P, Gastrin-releasing-peptide, prepro GRP |

| | | |
|---|---|---|
| TRPA1 | ENSP00000262209 | TRPA1, ANKTM1, hTRPA1, transient receptor potential cation channel, subfamily A, member 1, TRPA-1, transient receptor potential cation channel subfamily A member 1, TRP-A1 |
| CD4 | ENSP00000011653 | CD4, CD4 molecule, CD4 antigen, hCD4, CD4 receptor, CD 4, 1q6-8, CD-4, CD4-receptor, T cell surface glycoprotein CD4, CD 4 molecule, CD4- human, 5 Thr, 3 CD4, T-cell surface glycoprotein CD4 |
| SIRT2 | ENSP00000249396 | sirtuins, sirtuin, SIR2, SIR2Lp, hSIR2, SIRT2, Sir2p, SIR2L, Sirtuin 2, sirtuin-2, SIRT-2, Sirt2-p, Sirtuin2, SIRT 2, 5 mar |
| CDH2 | ENSP00000269141 | N-cadherin, CDH2, Cadherin 2, NCAD, N-Cad, neural cadherin, N cadherin, neural-cadherin, cadherin 2, type 1, N-cadherin (neuronal, N- cadherin, CDH-2, cadherin-2 |
| KLF4 | ENSP00000363804 | Kruppel-like factor 4, KLF4, GKLF, Kruppel-like factors, EZF, KLF-4, hKlf4, Kruppel-like factor4, Kruppel-like factor-4, Kruppel like factor 4, Krueppel-like factor 4 |
| SQSTM1 | ENSP00000374455 | p62, PDB3, SQSTM1, sequestosome 1, Zip3, p60, Zip3p, Sequestosome-1, hZip3, p-62, sequestosome1, ubiquitin-binding protein p62, SQSTM1 p, ubiquitin binding protein p62, SQSTM-1, SQSTM 1, phosphotyrosine-independent ligand for the Lck SH2 domain of 62 kDa |
| FGFR2 | ENSP00000410294 | FGFR2, fibroblast growth factor receptor 2, bek, CFD1, TK14, FGFR-2, K-SAM, fibroblast growth factor receptor-2, KGFR, fibroblast growth-factor receptor 2, Cfd1p, keratinocyte growth-factor receptor, keratinocyte growth factor receptor, TK25, KGF-R, fibroblast growth factor receptor2, FGFR 2, 2 PWL, FGFR2 p, Ect1, B BDs, FGF-R2 |
| ADCYAP1 | ENSP00000462647 | PACAP, ADCYAP1, pituitary adenylate cyclase activating polypeptide, pituitary adenylate cyclase-activating polypeptide, adenylate cyclase activating polypeptide 1, adenylate cyclase-activating polypeptide 1, adenylate cyclase-activating polypeptide 1 (pituitary, pituitary adenylate-cyclase activating polypeptide, pituitary adenylate cyclase- activating polypeptide |
| MUC5AC | ENSP00000485659 | Muc5AC, tracheo-bronchial mucin, tracheobronchial mucin, MUC5, mucin 5AC, Muc-5ac, MUC 5AC, LeB, mucin-5AC, mucin5AC, MUC5 AC, mucin 5AC, oligomeric mucus/gel-forming, MUC 5 |

| | | |
|---|---|---|
| CXCL10 | ENSP00000305651 | INP10, IP-10, CXCL10, Mob-1, IP10, chemokine ligand 10, Mob1, SCYB10, crg-2, CXCL-10, chemokine ligand-10, chemokine (C-X-C motif) ligand 10, Crg2, chemokine-ligand-10, Hc7, CXCL 10, C-X-C motif chemokine ligand 10, chemokine ligand 10, C-X-C motif chemokine 10, CXC motif chemokine 10, CXCL10p, CXC-L10, CXC motif chemokine ligand 10 |
| ANGPT2 | ENSP00000314897 | angiopoietin-2, Ang2, Angpt2, Ang-2, angiopoietin 2, ANGPT-2, Ang 2, ANGPT 2, Angiopoietin2, AGPT2 |
| ALOX5 | ENSP00000363512 | 5LO, ALOX5, 5-LO, arachidonate 5-lipoxygenase, EC 1.13.11.34, 5-LOX, 5LOX, Arachidonate 5 lipoxygenase, ALOX-5, EC1.13.11.34, Arachidonate-5-lipoxygenase, 5- lo |
| TXNIP | ENSP00000462521 | VDUP1, TXNIP, Thioredoxin binding protein-2, thioredoxin interacting protein, Thioredoxin-interacting protein, vitamin D3 up-regulated protein 1, Vitamin D3 upregulated protein 1, Thioredoxin-binding protein-2, Vitamin D3 up-regulated protein-1, thioredoxin binding protein 2, thioredoxin-binding protein 2, VDUP-1, THIF, Thioredoxin-interacting-protein |
| CDKN1B | ENSP00000228872 | Kip1, p27-Kip1, CDKN1B, p27Kip1, p27 KIP1, cyclin-dependent kinase inhibitor p27, MEN4, CDKN4, cyclin dependent kinase inhibitor p27, KIP 1, p27Kip-1, KIP-1, cyclin-dependent kinase inhibitor 1B, cyclin-dependent kinase inhibitor-1B, cyclin dependent kinase inhibitor 1B |
| SCARB1 | ENSP00000261693 | SR-BI, SRBI, CLA1, SR-B1, CLA-1, CD36 and LIMPII analogous-1, SCARB1, CD36L1, scavenger receptor class B,  member 1, scavenger receptor class B member 1, SRB1, hSR-B1, scavenger receptor class-B member 1, SRB-1, SRB-I, scavenger receptor class B member1 |
| SMAD2 | ENSP00000262160 | Smad2, Madr2, MADH2, Smad family, hsMAD2, SMAD-2, Smad 2, hMad2, JV18-1, Smad2P, JV-18, Smad2-P, mothers against decapentaplegic homolog 2, SMAD family member 2, Mad related protein 2, JV18 |
| SLC9A1 | ENSP00000263980 | NHE1, Slc9a1, NHE-1, solute carrier family 9, Na(+)/H(+) exchanger 1, Na(+)/H(+) exchanger-1, Na(+) /H(+) exchanger 1, Na(+) /H(+) exchanger-1, Sodium/hydrogen exchanger 1 |

| | | |
|---|---|---|
| BCHE | ENSP00000264381 | hE1, butyrylcholinesterase, BCHE, cholinesterase, cholinesterase 2, CHE1, pseudocholinesterase, CHE2, Che-1, EC 3.1.1.8, butyryl-cholinesterase, acylcholine-acyl-hydrolase, cholinesterase-2, acylcholine acylhydrolase, pseudo-cholinesterase, butyryl cholinesterase, AAH08396.1, butyrylcholine esterase, 1P0I, butyrylcholineesterase, 4BDS, acylcholine-acylhydrolase |
| MYCN | ENSP00000281043 | N-myc, MYCN, Nmyc, MODED, MYC-N, MYC not, Myc N, MYCN human, V-Myc Avian Myelocytomatosis Viral Oncogene Neuroblastoma-Derived Homolog, v-myc avian myelocytomatosis viral oncogene neuroblastoma derived homolog, N-myc proto-oncogene protein, N MYC |
| PLK1 | ENSP00000300093 | Plk1, polo-like kinase 1, Plk, 3----p36, Plk-1, STPK13, Polo-like kinase, 2 YAC, 3----p35, Polo-like kinase-1, PLK 1, polo-like kinase1, polo-like kinase 1, polo like kinase 1, Polo-like-kinase 1, Polo like kinase, Polo like kinase-1, 3p35, serine/threonine-protein kinase PLK1, Polo-like-kinase-1 |
| PSG1 | ENSP00000308970 | SP1, Sp-1, PSG4, PSGGA, PSBG1, Sp 1, PSG1, pregnancy-specific glycoproteins, DHFRP2, PSG95, PSG-1, pregnancy specific beta-1-glycoprotein 1, HSP1, PSG9, Pregnancy Specific Glycoproteins |
| CFLAR | ENSP00000312455 | FLIP, 3H11, I-FLICE, MRIT, CLARP, c-FLIP, FLAME-1, CFLAR, cFLIP, cFLIPL, cellular FLICE-like inhibitory protein, caspase-like apoptosis-regulatory protein, death effector domain containing, usurpin, c-FLIP-L, cFLIP-L, cFLIPS, c-FLIPS, c-FLIP L, c-FLIP-s, c-FLIPL, c- FLIP, c-FLIP R, c-FLIPR, cellular FLICE-like-inhibitory protein, caspase homolog, CASP8 and FADD-like apoptosis regulator, C Flip |
| F2R | ENSP00000321326 | PAR1, thrombin receptor, HTR, PAR-1, protease-activated receptor-1, F2r, protease activated receptor 1, proteinase-activated receptor-1, protease-activated receptor 1, protease-activated-receptor 1, Cf2r, thrombin-receptor, Par 1, protease activated receptor-1, Proteinase-activated receptor 1, hPar1, proteinase activated receptor-1, coagulation factor II receptor, protease-activated-receptor-1, coagulation factor II (thrombin) receptor, coagulation factor II thrombin receptor, 1-NRP, h-TR |
| DAND5 | ENSP00000323155 | Sp1, Sp-1, sp 1, Cerl-2, CER2, HSP1, DAND5, Cerl2, DAND5 p, Grem3 |
| JAK1 | ENSP00000343204 | JAK1, Janus kinase 1, Jak-1, JAK1A, JAK 1, Janus kinase1, Janus-Kinase-1, Janus kinase-1 |

| | | |
|---|---|---|
| MIR2055P | hsa-miR-205-5p | miR-205, MIR205, hsa-miR205, hsa-miR-205, miR-205-5p |
| IRS2 | ENSP00000365016 | Irs-2, insulin receptor substrate-2, IRS2, insulin receptor substrate 2, insulin-receptor substrate 2, insulin receptor substrate2 |
| GPC3 | ENSP00000377836 | GPC3, glypicans, glypican-3, SGBS1, OCI-5, Gpc-3, glypican 3, MXR7, GPC 3, Glypican3, GTR2-2 |
| EIF4G1 | ENSP00000416255 | p220, eIF4G, eIF4GI, PARK18, EIF4G1, eukaryotic translation initiation factor 4 gamma 1, eIF4G I, eIF4G1 p, eIF-4G, 4-aza, eIF4G-1, Eukaryotic translation initiation factor 4G-1, eukaryotic translation initiation factor 4G1, eukaryotic translation initiation factor 4-gamma 1 |
| DDIT3 | ENSP00000448665 | CCAAT/enhancer-binding protein homologous protein, CHOP-10, C/EBP homologous protein, DDIT3, gadd153, GADD 153, C/EBPzeta, C/EBP-homologous protein, CHOP10, DNA-damage-inducible transcript 3, CCAAT/enhancer binding protein homologous protein, CCAAT/enhancer binding-protein homologous protein, GADD-153, C/EBP homologous protein 10, CH OP, CCAAT/enhancer-binding protein-homologous protein, DNA Damage-Inducible Transcript 3, CCAAT/-enhancer-binding protein homologous protein, C/EBP homologous protein-10, CEBPZ, DNA damage inducible transcript 3, C/EBPhomologous protein, DNA damage-inducible transcript 3 protein, C/-EBP homologous protein |
| KEAP1 | ENSP00000171111 | Keap1, Kelch-like ECH-associated protein 1, 4 IFN, 4 in 4, Kelch-like ECH-associated protein-1, INrf2, Kelch like-ECH-associated protein 1, keap 1, Keap-1, Kelch-like ECH Associated Protein 1, Kelch-like ECH-associated protein1, kelch like ECH-associated protein 1, kelch like ECH associated protein 1, Kelch like ECH-associated protein1 |
| IL2 | ENSP00000226730 | interleukin-2, IL-2, Il2, interleukin 2, IL 2, TCGF, T-cell growth factor, T cell growth factor, hIL-2, Lymphokine, IL- 2, aldesleukin, interleukin2, hIL2, IL - 2, 3 in K |
| IGFBP5 | ENSP00000233813 | IGFBP-5, Igfbp5, insulin-like growth factor-binding protein 5, insulin-like growth factor-binding protein-5, insulin-like growth factor binding protein-5, insulin-like growth factor binding protein 5, IGFBP 5, insulin like growth factor binding protein 5 |

| | | |
|---|---|---|
| FLT3 | ENSP00000241453 | Flt3, Flk-2, Flt-3, stem cell tyrosine kinase-1, FLK2, STK-1, fms-related tyrosine kinase 3, FMS-like tyrosine kinase-3, fms-like tyrosine kinase 3, STK1, fetal liver kinase 2, stem cell tyrosine kinase 1, CD135, FMSlike tyrosine kinase 3, hFlt3, FMS-Like-Tyrosine kinase-3, fms-like tyro-sine kinase 3, fms-related tyrosine kinase-3, Fms-like  tyrosine kinase 3, S-TK1, FMS-like-tyrosine-kinase-3, FMS related tyrosine kinase 3, FMS like tyrosine kinase 3, FLT3 p, FMS-like tyrosine kinase3, FMS-like tyrosine-kinase-3, FMS-like tyrosine-kinase 3, fetal liver kinase-2, Fms related tyrosine kinase-3, Fms-related-tyrosine kinase 3, flt 3 |
| PPIG | ENSP00000260970 | CYP, CARS-Cyp, CASP10, SRcyp, Clk associating RS-cyclophilin, CASP-10, Casp 10, peptidyl-prolyl cis-trans isomerase G, PPIG |
| NCOR1 | ENSP00000268712 | N-CoR, N-CoR1, NCoR, TRAC-1, NCoR1, nuclear receptor corepressor 1, nuclear receptor co-repressor 1, 4 MDD, NCoR-1 |
| HK2 | ENSP00000290573 | HKII, hK2, hexokinase 2, EC 2.7.1.1, hexokinase-2, Hexokinase type II, HK II, HK-II, AC104135, HXK2, Hk-2, hexokinase2 |
| CCR5 | ENSP00000292303 | CCR5, ChemR13, CC CKR5, CKR-5, CKR5, chemokine receptor 5, chemokine receptor-5, CC-CKR-5, CC-CKR5, CMKBR5, ccr-5, chemokine-receptor 5, CCR 5, hCCR5, CD195, CCR5 human, C-C motif chemokine receptor 5, CC-motif chemokine receptor 5, C-C chemokine receptor type 5, 4 MBs, CHEMOKINE, CC chemokine receptor type 5, C-C chemokine receptor type-5, CC motif chemokine receptor 5, C-C motif chemokine receptor5, C-C motif chemokine receptors, CC-chemokine receptor type 5, HIV-1 fusion coreceptor |
| STAT5B | ENSP00000293328 | STAT5B, STAT5, signal transducer and activator of transcription 5b, hStat5, STAT 5, STAT-5, STAT 5b, STAT-5b, STAT5-P |
| FASN | ENSP00000304592 | Fasn, EC 2.3.1.85, 3 HHD |

| | | |
|---|---|---|
| UGT1A1 | ENSP00000304845 | UGT1A1, UGT1A, UGT1A8, UGT1, HUG-Br1, UDP-glucuronosyltransferase 1A, HUGT1, Gnt-1, UDPGT, UDP-glucuronosyltransferase 1A1, UGT-1, UGT-1A, GNT1, UGT-1 A, UGT1-A1, hUGT1A1, UDP-glucuronosyl transferase 1A1, UGT1 A1, UGT 1A1, UGT-1A1, UDP glucuronosyltransferase-1A, UDP-glucuronosyltransferase1A1, UDP glucuronosyltransferase 1A1, UDP-glucuronosyl-transferase 1A1, UDP-glucuronosyltransferase 1A, UDP-glucuronosyl transferase 1A, UGT 1A, UDP glucuronosyltransferase1A1, UDP-glucuronosyltransferase 1 A1, UDP glucuronosyltransferase 1A, UDP-glucuronosyltransferase 1A8, UDP glucuronosyltransferase family 1 member A1, UDP-glucuronosyltransferase-1A1, UGT1A 1, HUG Br1, hugBr1 |
| IRS1 | ENSP00000304895 | IRS-1, Insulin receptor substrate-1, hIRS-1, IRS1, insulin receptor substrate 1, irs 1, Insulin-receptor substrate 1, insulin-receptor substrate-1, insulin receptor substrate -1 |
| UCP2 | ENSP00000312029 | UCP2, uncoupling protein-2, uncoupling protein 2, UCP-2, Mitochondrial uncoupling protein-2, mitochondrial uncoupling protein 2, hUCP2, Uncoupling Protein2 |
| STAT5A | ENSP00000341208 | Stat5a, STAT5, MGF, signal transducer and activator of transcription 5a, STAT-5, STAT 5, hStat5, STAT 5 A, STAT 5A, STAT5-P, signal transducer and activator of transcription-5a, Signal Transducer and Activator of Transcription 5 A |
| AIM2 | ENSP00000357112 | AIM2, absent in melanoma 2, AIM-2, absent-in-melanoma 2, Absent in melanoma-2 |
| HDGF | ENSP00000357189 | HDGF, heparin-binding growth factor, hepatoma-derived growth factor, heparin binding growth factor, HBGF, heparin-binding growth factor family, heparin-binding growth factor |
| LCN2 | ENSP00000362108 | p25, lipocalins, 24p3, NGAL, lipocalin 2, Lcn2, lipocalin-2, neutrophil gelatinase-associated lipocalin, HNL, oncogene-24p3, siderocalin, lipocalin2, neutrophil gelatinase associated lipocalin, oncogene 24p3, LCN 2, Lcn-2, N-GAL, p-25, hLCN2, Neutrophil Gelatinase Associated-Lipocalin, neutrophil gelatinase- associated lipocalin, 1 ng L, Neutrophil-gelatinase-associated-lipocalin, HN-L, neutrophil-gelatinase associated lipocalin |

| | | |
|---|---|---|
| UGT1A8 | ENSP00000362549 | HUGT1, UGT1A1, UDP-glucuronosyltransferase 1A1, ugt1, UGT1A, UGT-1A, UGT1A8, UDP-glucuronosyltransferase 1A, Gnt-1, GNT1, UDPGT, UGT-1, UGT-1 A, UGT1-A1, hUGT1A1, UDP-glucuronosyl transferase 1A1, UGT1 A1, UGT 1A1, UGT-1A1, UDP glucuronosyltransferase-1A, UDP glucuronosyltransferase 1 family, polypeptide A1, UDP-glucuronosyltransferase 1 family, polypeptide A1, UDP-glucuronosyltransferase1A1, UDP glucuronosyltransferase 1A1, UDP-glucuronosyl-transferase 1A1, UDP-glucuronosyltransferase 1A, UDP-glucuronosyl transferase 1A, UGT 1A, UDP glucuronosyltransferase1A1, UDP-glucuronosyltransferase 1 A1, UDP glucuronosyltransferase 1A, UDP-glucuronosyltransferase 1A8, UDP glucuronosyltransferase family 1 member A1, UDP-glucuronosyltransferase-1A1, UGT1A 1 |
| NR5A1 | ENSP00000362690 | SF-1, steroidogenic factor-1, steroidogenic factor 1, Hsf1, Sf1, NR5A1, Ad4BP, Ftz-F1, HSF-1, FTZF1, FTZ1, HSF 1, nuclear receptor subfamily 5, group A, member 1, Nuclear receptor subfamily 5 group A member 1, NR5A1 p, adrenal 4 binding protein |
| PPP1R13B | ENSP00000202556 | p85, ASPP1, PPP1R13B, Apoptosis Stimulating of P53 Protein 1 |
| GSR | ENSP00000221130 | GSR, glutathione reductase, gluta thione reductase, glutathione S-reductase, glutathione-disulfide reductase, GluR, glutathione-reductase |
| CYP4F2 | ENSP00000221700 | CYP4F2, cytochrome P-450 family 4, cytochrome P450, family 4, subfamily F, polypeptide 2, cytochrome P450 4F2, Cytochrome P450 family 4 subfamily F member 2 |
| CD38 | ENSP00000226279 | CD38, CD38 molecule, EC 3.2.2.6, CD 38, CD38 antigen, 3 ROP, CD38 human |
| KCNA5 | ENSP00000252321 | hCK1, hK2, KCNA5, Kv1.5, hPCN1, potassium voltage-gated channel, shaker-related subfamily, member 5, potassium voltage-gated channel, shakerrelated subfamily, member 5, KCNA5 p, potassium voltage-gated channel subfamily A member 5, Hk-2 |
| MMP7 | ENSP00000260227 | matrilysin, MMP-7, pump-1, MMP7, Matrix metalloproteinase 7, matrin, Matrix metalloproteinase-7, 1 MMP, 1-MMP, 1 MMR, Matrix metalloproteinase7, matrix metallopeptidase 7, 1MMP, matrix-metalloproteinase-7, matrix metallopeptidase-7 |

| | | |
|---|---|---|
| NOX4 | ENSP00000263317 | renal NAD(P)H oxidase, NOX4, KOX, Renox, KOX 1, KOX1, Nox-4, Nox 4, NADPH oxidase 4, KO X, NADPH oxidase-4, NADPH oxidase4 |
| CDK2 | ENSP00000266970 | cyclin-dependent kinase 2, CDK2, CDKN2, cyclin-dependent kinase2, 2DS1, cyclin-dependent kinase-2, 2R3R, CDK-2, cyclin dependent kinase 2, 4 CFU, cyclin dependent kinase-2, 4 BCP, 3 EOC, 2 bpm, CDK 2, 3 IgG, 1 HCL |
| ERBB3 | ENSP00000267101 | HER3, ErbB3, 3----p 11, erbB-3, 3 p11, 3----p11, c-erbB-3, HER-3, human epidermal growth factor receptor 3, c-erbB3, erb-B-3, h-erb-B-3, MDA-BF-1, p180-ErbB3, Her- 3, c-erb-B3, p45-sErbB3, ERB-B3, human epidermal growth factor receptor-3, receptor tyrosine-protein kinase ErbB3, Tyrosine kinase-type cell surface receptor HER3, Erb-B2 receptor tyrosine kinase 3, Receptor Tyrosine-Protein Kinase ErbB-3, c-erb B3 |
| TJP1 | ENSP00000281537 | tight junction protein ZO-1, ZO-1, zona occludens-1, zona occludens protein 1, Tjp1, zona occludens protein-1, ZO1, tight junction protein 1, zona occludens 1, zonula occludens protein-1, zonula occludens protein 1, tight junction protein-1, Zo- 1, tight-junction protein ZO-1, zona-occludens-1, tjp-1 |
| ID1 | ENSP00000365280 | Id1, Id-1, HID1, inhibitor of differentiation 1, DNA-binding protein inhibitor ID1, inhibitor of DNA binding 1, inhibitor of differentiation-1, Inhibitor of DNA binding-1, Inhibitor of DNA-binding-1, DNA-binding protein inhibitor ID-1, inhibitor of DNA-binding 1 |
| SF1 | ENSP00000366604 | SF-1, SF1, HSF1, ZNF162, ZFM1, HSF-1, D11S636, BBP, mBBP, Splicing factor 1, HSF 1, splicing factor-1, BB p |
| NOX5 | ENSP00000373518 | NOX5, NADPH oxidase 5, NADPH oxidase5, NOX-5 |
| CDK1 | ENSP00000378699 | Cdc2, Cdk1, p34CDC2, cyclin-dependent kinase 1, cdc-2, cyclin dependent kinase 1, cyclin-dependent kinase-1, Cdkn1, cdc 2, CDK-1, cyclin-dependent kinase1, p34 cdc2 |
| CXCL1 | ENSP00000379110 | CXCL1, Gro-1, melanoma growth-stimulatory activity, Melanoma growth stimulatory activity, MGSA, gro, GRO1, GRO-a, CXCL-1, GRO 1 oncogene, C-X-C motif chemokine 1, chemokine (C-X-C motif) ligand 1 (melanoma growth stimulating activity,  alpha, fibroblast secretory protein, CHEMOKINE, chemokine (C-X-C motif) ligand 1 (melanoma growth-stimulating activity,  alpha, GROa, F-SP, C-X-C motif chemokine ligand-1, C-X-C motif chemokine ligand 1 |

| | | |
|---|---|---|
| ILK | ENSP00000379975 | p59, ILK1, ILK, integrin-linked kinase, integrin linked kinase, ILK-2, ILK-1, Integrin-linked protein kinase, p59ILK, HP59, ILK2 |
| ELAVL1 | ENSP00000385269 | HuR, elav, ELAVL1, MELG, Hu antigen R, ELAV1, Hu-r, Hu-antigen R, ELAV-like protein 1, ELAVL-1, ELAV-like RNA binding protein 1, Hu Antigen-R, ELAV-like RNA-binding protein 1, ELAV-1, ELAV-L1, ELAV like RNA binding protein 1, ELAV like RNA-binding protein 1 |
| FGFR1 | ENSP00000393312 | FLG, Fgfr1, fibroblast growth factor receptor 1, H-2, FGFR-1, KAL2, fibroblast growth factor receptor-1, hh3, FGF R1, FGFR 1, FGF-R1, FGF-R-1, H2-P, KAL-2, HH2, 1 C vs, fibroblast growth-factor receptor 1, bFGF-r, HH4, FGFR1 p, FLT2, proto-oncogene c-fgr |
| SLC52A1 | ENSP00000399979 | PAR2, RFT1, hRFT1, SLC52A1, RFVT1, GPR172B, G protein-coupled receptor 172B, RFT1 p, riboflavin transporter 1, PAR 2, RFT-1, PAR-2, hPar2, hRFVT -1, RFVT-1, hRFVT-1 |
| AGTR1 | ENSP00000419422 | AT1, AT1R, type 1 angiotensin II receptor, Agtr1b, AT1bR, angiotensin II type 1 receptor, Hat1, angiotensin II receptor type 1, Agtr1a, AT1aR, AT1B, AGTR1, angiotensin II receptor,  type 1, angiotensin II type-1 receptor, AT-1, type-1 angiotensin II receptor, angiotensin II type 1-receptor, AT1-R, Angiotensin-II type 1 receptor, AT 1, angiotensin-II type-1 receptor, AT1 R, angiotensin-II receptor type 1, angiotensin-II-type-1 receptor, angiotensin II-type 1 receptor, AT 1R, AGTR1 A, AF245699, AT1a-R, angiotensin II type1 receptor, AT2R1, angiotensin II receptor type-1, hAT1R, AT 1 R, AGT R1, 4YAY, AT-1R, AGTR 1, type 1 angiotensin-II receptor, h AT1 |
| ADAM9 | ENSP00000419446 | MDC9, MDC-9, ADAM9, meltrin gamma, ADAM metallopeptidase domain 9, metalloprotease/disintegrin/cysteine-rich protein 9, meltrin-gamma, ADAM 9, ADAM-9, disintegrin and metalloproteinase domain-containing protein 9 |
| MUC5B | ENSP00000436812 | HMG-1, MUC5B, MG1, HMG1, MUC5, MG-1, mucin 5B, MUC9, MUC-5B |
| HPGD | ENSP00000296522 | PGDH, hPGD, 15-hydroxyprostaglandin dehydrogenase, 15-PGDH, 15-hydroxy-prostaglandin dehydrogenase, 15PGDH, 15-Hydroxyprostaglandin-dehydrogenase, hydroxyprostaglandin dehydrogenase 15-(NAD, PHOAR1, 15-Hydroxy-Prostaglandin-Dehydrogenase, 15-hydroxy prostaglandin dehydrogenase |

| | | |
|---|---|---|
| STAT6 | ENSP00000300134 | STAT6, IL-4 STAT, STAT-6, Stat6b, Stat6c, signal transducer and activator of transcription-6, signal transducer and activator of transcription 6, STAT 6, IL4-Stat, signal transducer and activator of transcription6, STAT6 C |
| SPHK1 | ENSP00000313681 | SPHK1, hSPHK1, SPHK, sphingosine kinase 1, sphingosine kinase-1, SphK-1, sphingosine-kinase-1, Sphingosine-kinase 1, SPHK 1, sphingosine kinase1 |
| KLK3 | ENSP00000314151 | KLK3, prostate specific antigen, kallikreins, prostate-specific antigen, HK3, p30 antigen, prostate-specific-antigen, KLK 3, aPS, hKLK3, kallikrein 3, Psa, gamma-seminoprotein, kallikrein-related peptidase 3, kallikrein-related peptidase-3, 2PSA, 2 PSA, kallikrein related peptidase 3, prostate - specific antigen, prostate- specific antigen, Prostate Specific-Antigen |
| EIF4EBP1 | ENSP00000340691 | 4E-BP1, BP1, BP-1, PHAS-I, Eif4ebp1, 4EBP1, eukaryotic translation initiation factor 4E binding protein 1, BP 1, 4EBP-1, 4E-BP-1, eukaryotic translation initiation factor 4E binding protein-1, EIF4E-BP1, eukaryotic translation initiation factor 4E-binding protein 1, B P1, 4E- BP1, 4-EBP1, eukaryotic translation initiation factor 4E binding protein1 |
| FOXM1 | ENSP00000342307 | HNF3, Foxm1b, FoxM1, Ins-1, HNF-3, HFH-11, MPM2-reactive phosphoprotein 2, MPP2, FKHL16, M phase phosphoprotein 2, Forkhead box M1, MPP-2, TGT3, INS1, Forkhead box protein M1, Forkhead boxM1, Fork head box M1, Forkheadbox protein M1 |
| MAP3K5 | ENSP00000351908 | ASK1, apoptosis signal-regulating kinase-1, apoptosis signal-regulating kinase 1, MAPKKK5, MAP3K5, apoptosis-signal regulating kinase-1, ASK-1, MEKK5, Apoptosis signal-regulating kinase1, Apoptosis-signal-regulating-kinase-1, apoptosis signal regulating kinase 1, mitogen-activated protein kinase kinase kinase-5, Apoptosis signal regulating kinase-1, apoptosis-signal-regulating kinase 1 |
| TFRC | ENSP00000353224 | Trfr, TfR, transferrin receptor, p90, TfR1, CD71, Tfrc, hT9, transferrin-receptor, Tf-R, cd 71, TfR-1, hTfR1, HT-9, transferrin receptor protein 1 |
| UTRN | ENSP00000356515 | Utrophin, DRP-1, Drp1, UTRN, d RP, Drp |

| | | |
|---|---|---|
| DNTT | ENSP00000360216 | terminal deoxynucleotidyltransferase, TdT, terminal deoxynucleotidyl transferase, EC 2.7.7.31, DNTT, terminal-deoxynucleotidyl-transferase, terminal deoxynucleotidyl-transferase, terminal-deoxynucleotidyl transferase, terminal deoxy-nucleotidyl transferase, terminaldeoxynucleotidyl transferase, terminal deoxynucleotidyl trans-ferase |
| TFF1 | ENSP00000291527 | PS2, TFF1, HPS2, BCEI, PS-2, pNR-2, HPS-2, trefoil factor 1, PS -2, BCE I, Ps 2, protein pS2, TFF-1, Trefoil factor-1, BCE-I, TFF 1 |
| CIP2A | ENSP00000295746 | p90, CIP2A, Cancerous Inhibitor of PP2A, KIAA1524, p90 autoantigen, CIP2A p |
| PSMD1 | ENSP00000309474 | S1P, RPN2, Psmd1, p112, S-1-P, hRpn2 |
| CD34 | ENSP00000310036 | CD34, CD34 antigen, hCD34, CD 34, hematopoietic progenitor cell antigen CD34, CD-34, CD34 molecule, CD 34 antigen, CD34- human, CD3-4 |
| KLK2 | ENSP00000313581 | KLK2, HK2, glandular kallikrein-1, kallikrein 2, kallikreins, hGK-1, hKLK2, kallikrein-2, glandular kallikrein 1, KLK-2, kallikrein-related peptidase 2, KLK 2, Hk-2, hGK1 |
| TAC1 | ENSP00000321106 | substance P, NK2, neurokinin-1, substance K, Neurokinin 1, Tac2, tac1, nk-2, neurokinin A, preprotachykinin, substance-P, NKA, neurokinin-2, 4 h OM, protachykinin-1, neurokinin 2, neurokinin1, HNK2, neuropeptide K, neuropeptide gamma, N-Pk, Protachykinin, tachykinin precursor 1, pPT, tachykinin, precursor 1, Tac 1, Pp T, protachykinin 1, TAC-2 |
| HSPA5 | ENSP00000324173 | GRP78, Hspa5, 78 kDa glucose-regulated protein, MIF2, 78-kDa glucose-regulated protein, GRP-78, Bip, immunoglobulin heavy-chain binding protein, MIF-2, 78-kDa glucose regulated protein, heat shock 70-kDa protein 5, 78kDa glucose-regulated protein, immunoglobulin heavy chain binding protein, Heat shock 70 kDa protein 5, immunoglobulin heavy chain-binding protein, 3 LDL, 78 kDa glucose regulated protein, GRP 78, immunoglobulin-heavy-chain-binding protein, heat shock 70 kDa proteins, glucose regulated protein, 78kDa, glucose regulated protein, 78 kDa, heatshock 70-kDa protein 5, 78kDa glucose regulated protein, immunoglobulin heavy-chain-binding protein, heat shock protein family A member 5, Heat Shock Protein Family A (Hsp70) Member 5, glucose-regulated protein, 78kDa |

| | | |
|---|---|---|
| PMAIP1 | ENSP00000326119 | Noxa, PMAIP1, protein NOXA, phorbol-12-myristate-13-acetate-induced protein 1, PMAIP-1, phorbol 12-myristate 13-acetate induced protein 1, aPR |
| LEPR | ENSP00000330393 | LEPR, ObR, leptin receptor, OB-R, leptin-receptor, OB receptor, Ob-receptor, HuB219, Lep-R |
| SELE | ENSP00000331736 | E-selectin, ELAM-1, endothelial-leukocyte adhesion molecule 1, ELAM, endothelial leukocyte adhesion molecule-1, endothelial leukocyte adhesion molecule 1, SELE, endothelial-leukocyte-adhesion-molecule-1, CD62E, endothelial-leukocyte adhesion molecule-1, selectin E, endothelial adhesion molecule-1, endothelial leukocyte-adhesion molecule-1, E-sel, E- selectin, E selectin, selectine, CD62 E, ESEL, endothelial adhesion molecule 1, SEL-E |
| MAPK11 | ENSP00000333685 | p38beta, p38-2, p38 beta 2, p38beta2, SAPK2, MAPK11, SAPK2b, p38-beta |
| SMAD4 | ENSP00000341551 | SMAD4, MADH4, Smad family, JIP, DPC4, SMAD-4, DPC-4, Smad 4, hSmad4, mothers against decapentaplegic homolog 4, 1U7F, SMAD family member 4, SMAD family member 4, SMAD4 p |
| MBTPS1 | ENSP00000344223 | Site-1 protease, S1P, Mbtps1, site-1-protease, SKI-1, membrane-bound transcription factor peptidase, site 1, S-1-P, site 1 protease, subtilisin-kexin isozyme-1, subtilisin/kexin-isozyme-1, Subtilisin Kexin Isozyme-1, subtilisin/kexin-isozyme 1 |
| AP2A1 | ENSP00000351926 | AP-2 alpha, alpha-Adaptin A, AP-2alpha, AP2 alpha, AP2alpha, AP2A1, AP2-alpha, alpha 1 adaptin |
| P2RX4 | ENSP00000353032 | P2X4, P2rx4, ATP receptor, purinergic receptor, P2X4R, purinergic receptor P2X4, Purinergic receptor P2X 4, P2X purinoceptor 4 |
| DUSP12 | ENSP00000356920 | YVH1, serine/threonine-specific protein phosphatase, DUSP1, dual-specificity phosphatase 12, DUSP12, DUSP-1, Dual Specificity Phosphatase 12 |
| RUNX2 | ENSP00000360493 | CBFA1, Runx2, Ccd1, Runt-related transcription factor 2, Ccd, OSF2, AML3, Runx-2, Cbfa-1, runt-related transcription factor-2, AML-3, OSF-2, RUNX2 p, runt related transcription factor 2, Runx 2, runt-related transcription factor2 |

| | | |
|---|---|---|
| HDAC1 | ENSP00000362649 | Rpd3, histone deacetylase-1, HDAC1, NuRD complex, Sin3 histone deacetylase complex, HD1, HDAC-1, Rpd3p, histone deacetylase 1, RPD3L1, HDAC1p, hRPD3, hHDAC1, HDAC 1, HD-1, EMSY Complex, histone deacetylase1, histone-deacetylase 1 |
| GRB2 | ENSP00000376345 | GRB2, Grb-2, protein ASH, growth factor receptor-bound protein 2, growth factor receptor bound protein 2, growth factor receptor bound protein-2, Grb3-3, 2 VWF, adapter protein Grb2, 3 in 7, Growth factor receptor-bound protein-2, 3 in 8 |
| HOOK2 | ENSP00000380785 | HK2, HOOK2, hook 2, Hk-2 |
| KIF2A | ENSP00000385000 | hK2, KIF2A, KIF2, Kinesin-2, kinesin 2, KNS2, kinesin heavy chain member 2, Kinesin family member 2a, Hk-2 |
| TBC1D9 | ENSP00000411197 | MDR-1, MDR1, MDR1 P, mdr 1, MDR1-P, TBC1D9, MDR-1 P, MDR-1, MDR1p, hMDR1 |
| CASR | ENSP00000420194 | CASR, calcium-sensing receptor, Ca-SR, calcium sensing receptor, extracellular calcium-sensing receptor, Gprc2a, PCAR1, extracellular calcium sensing receptor, U20759, hCaSR, calcium- sensing receptor, Ca SR, calcium -sensing receptor |
| PECAM1 | ENSP00000457421 | PECAM-1, CD31, platelet endothelial cell adhesion molecule, GPIIa, PECAM1, CD-31, platelet-endothelial cell adhesion molecule, platelet/endothelial cell adhesion molecule-1, PE-CAM-1, platelet/endothelial cell adhesion molecule 1, CD 31, CD31 antigen, platelet/ endothelial cell adhesion molecule 1, platelet/ endothelial cell adhesion molecule-1 |
| TRPV1 | ENSP00000459962 | VR1, vanilloid receptor-1, TRPV1, HVR1, vanilloid receptor 1, CARKL, capsaicin receptor, transient receptor potential cation channel, subfamily V, member 1, vanilloid receptor subtype 1, capsaicin-receptor, Vanilloid receptor subtype-1, transient receptor potential cation channel subfamily V member 1, HVR 1, HVR-1, VR-1, hTRPV1, TRPV-1, SHPK, TRPV 1, SHK, transient receptor potential cation channel subfamily V member1 |

| | | |
|---|---|---|
| TRPV1 | ENSP00000461007 | vanilloid receptor subtype 1, vanilloid receptor-1, capsaicin receptor, VR1, TRPV1, HVR1, Vanilloid receptor subtype-1, Vanilloid Receptor 1, transient receptor potential cation channel, subfamily V, member 1, capsaicin-receptor, transient receptor potential cation channel subfamily V member 1, HVR 1, HVR-1, VR-1, hTRPV1, TRPV-1, TRPV 1, transient receptor potential cation channel subfamily V member1 |
| VEGFC | ENSP00000480043 | Vegf-C, Vegfc, Flt4 ligand, VEGF family, vascular endothelial growth factor-C, Vascular endothelial growth factor C, Vascular endothelial growth factor-related protein, VEGF-family, VEGF C, vascular en-dothelial growth factor C, VEGF -C |
| CETP | ENSP00000200676 | Cholesteryl ester transfer protein, CETP, cholesteryl-ester transfer protein, cholesteryl-ester-transfer protein, cholesterylester transfer protein, Cholesteryl ester-transfer protein, cholesteryl ester transfer protein, plasma, cholesteryl-ester-transfer-protein, Ce-tP |
| XRCC1 | ENSP00000262887 | XRCC1, Xrcc-1, X-ray repair cross-complementing protein 1, X-ray repair cross-complementing 1, X-Ray Repair Cross Complementing 1, XRCC 1, X-ray repair cross-complementing-1, x-ray repair complementing defective repair in Chinese hamster cells 1, X-ray repair cross complementing protein 1, X-ray repair crosscomplementing1, DNA repair protein XRCC1 |
| IL1A | ENSP00000263339 | IL-1, interleukin-1alpha, IL-1alpha, IL1A, IL-1 alpha, IL1, interleukin-1 alpha, interleukin 1 alpha, IL-1a, interleukin 1alpha, IL1alpha, hematopoietin 1, IL-1-alpha, interleukin 1A, hIL-1, interleukin-1A, Interleukin-1--a, interleukin 1-alpha, IL1 alpha, interleukin -1alpha, IL1-A, IL 1, I-L1alpha, IL -1, interleukin-1 A, hIL1, h IL-1, interleukin-1-alpha, IL1-alpha |
| NOTCH3 | ENSP00000263388 | Notch3, NOTCH 3, Notch-3, Notch homolog 3, neurogenic locus notch homolog protein 3 |
| AIP | ENSP00000279146 | aryl hydrocarbon receptor-interacting protein, aryl hydrocarbon receptor interacting protein, XAP2, ARA9, 4 apo, Ah receptor-interacting protein, Aip, aryl-hydrocarbon receptor interacting protein, aryl-hydrocarbon-receptor interacting protein, HGNC:358, aryl-hydrocarbon receptor-interacting protein, AI P |
| MIR1433P | hsa-miR-143-3p | miR-143, Mir143, hsa-miR-143, miR-143-3p, hsa-miR-143-3p, miR-143 -3p, miR- 143-3p |

| | | |
|---|---|---|
| MIR146A5P | hsa-miR-146a-5p | miR-146, miR-146a, hsa-mir-146a, miR146a, hsa-miR-146, hsa-mir146a, mir-146a-5p, miR146, Hsa-miR-146a-5p, miR- 146a, miR-146a- 5p, miR -146a, hsamiR-146a-5p, miR--146a, miR-146-a |
| TNFSF13B | ENSP00000365048 | TALL-1, BAFF, BLyS, TNFSF20, zTNF4, B lymphocyte stimulator, B cell-activating factor, B-cell activating factor, B-cell-activating factor, Tnfsf13b, tumor necrosis factor superfamily, B cell activating factor, B-lymphocyte stimulator, tumor-necrosis factor superfamily, tumor necrosis factor super-family, CD257, DTL, tumor necrosis factor-superfamily, B-cell-activating-factor, TNF superfamily member 13b, BAF, tumor necrosis factor super family |
| IGFBP3 | ENSP00000370473 | IGFBP-3, insulin-like growth factor-binding protein-3, IGF-binding protein-3, IGFBP3, insulin-like growth factor binding protein-3, IGF binding protein-3, insulinlike growth factor-binding protein-3, IGF binding protein 3, insulin-like growth factor-binding protein 3, BP-53, IGF-BP3, IGFBP3 -202, insulin-like growth factor binding protein 3, IGF-binding protein 3, Bp 53, Bp53, hIGFBP-3, IGFBP-3 -202, insulinlike growth factor binding protein-3, IGFBP 3, IGFBP 3 -202, insulin-like growth-factor-binding protein-3, insulin like growth factor-binding protein 3, IGFbinding protein 3, insulinlike growth factor binding protein 3, IGFBP-3-202, IGF BP-3, Insulin Like Growth Factor Binding Protein-3, IBP3, IGFBP - 3, insulin like growth factor binding protein 3, insulin-like-growth-factor-binding-protein-3 |
| CD9 | ENSP00000371958 | p24, CD9, MRP1, Hb A2, HBA2, MRP-1, BA-2, CD9 antigen, motility-related protein, Motility-Related Protein 1, CD9 molecule, motility-related protein-1, CD-9, motility related protein-1, P24P, MRP 1, P2 4, TSPAN29, MIC3, HP24, Hb-A2, Motility related protein 1 |
| LTB4R2 | ENSP00000445772 | BLTR2, BLT2, Nop9, JULF2, Leukotriene B4 receptor BLT2, leukotriene B4 receptor-2, leukotriene B4 receptor 2, LTB4R2 |
| IKBKE | ENSP00000464030 | IKK-i, inducible IkappaB kinase, IKKi, IKBKE, inhibitor of kappa light polypeptide gene enhancer in B-cells,  kinase epsilon, Inhibitor of kappa light polypeptide gene enhancer in B cells,  kinase epsilon, inhibitor of nuclear factor kappa-B kinase subunit epsilon, Inhibitor of Nuclear Factor Kappa B Kinase Subunit Epsilon |

| | | |
|---|---|---|
| KRT16 | ENSP00000301653 | PC-1, HPC1, KRT16, Pc 1, keratin 16, CK16, Pc1, KRT16A, HPC-1, CK 16, cytokeratin 16, cytokeratin-16, keratin-16, keratin16 |
| PGR | ENSP00000325120 | progesterone receptor, PGR, Progesterone-receptor, p-GR |
| BGN | ENSP00000327336 | biglycan, BGN, PGI, Pg I, PG-I, DS-PG-1, PG-S1, PGS1, biglycan proteoglycan |
| SNCA | ENSP00000338345 | alpha-synuclein, PD-1, SNCA, PARK1, alpha synuclein, PD1, hPD-1, alpha -synuclein, 3----q26, PARK4, non-A beta component of AD amyloid, PARK 1, non-Abeta component of AD amyloid, PD 1, alpha- synuclein, 1XQ8, synuclein-a, synuclein alpha, synuclein--a, Alpha - synuclein, hPD1, Non A4 Component of Amyloid Precursor, PD- 1, alphasynuclein |
| C1QTNF6 | ENSP00000338812 | C1QTNF6, CTRP6 |
| PTK2 | ENSP00000341189 | FAK, Fadk, pp125FAK, p125fak, FRNK, focal adhesion kinase-related nonkinase, protein tyrosine kinase 2, FAK1, PTK2 protein tyrosine kinase 2, Focal Adhesion Kinase 1, Focal Adhesion Kinase-1, 4 EBV |
| BTG3 | ENSP00000344609 | BTG3, APRO4, ANA/BTG3 |
| MDM4 | ENSP00000356150 | MDMX, MRP1, HdmX, MDM4, protein MDM4, hMRP1, MRP-1, MRP1 p, protein Mdmx, Mdm4 p53 binding protein homolog, MRP 1, MDM-4, double minute 4 protein |
| RBP4 | ENSP00000360522 | retinol binding protein-4, plasma retinol-binding protein, retinol binding protein 4, RBP4, Rbp-4, Retinol-binding protein 4, plasma retinol binding protein, lipocalins, Retinol-binding protein-4, plasma retinol binding-protein, Retinol-binding protein4, retinol binding protein -4, retinol-binding-protein-4, RBP 4, RBP -4, retinol binding-protein-4 |
| NCOA3 | ENSP00000361066 | RAC3, pCIP, AIB1, SRC-3, TRAM-1, ACTR, AIB-1, SRC3, p/CIP, NCOA3, thyroid hormone receptor activator molecule 1, NCoA-3, RAC 3, RAC-3, nuclear receptor coactivator 3, TRAM1, nuclear receptor coactivator-3, nuclear receptor co-activator-3, receptor-associated coactivator 3, CBP-interacting protein |

| | | |
|---|---|---|
| NGFR | ENSP00000172229 | p75NTR, p75(NTR, nerve growth factor receptor, NGFR, CD271, low affinity neurotrophin receptor p75NTR, low-affinity nerve growth factor receptor, nerve-growth-factor receptor, Low-affinity nerve-growth factor receptor, NGF-R, low-affinity neurotrophin receptor p75NTR, p75 NTR, p75 (NTR, tumor necrosis factor receptor superfamily member 16, NGF R, CD-271, p75 ICD, low affinity nerve growth factor receptor |
| APEX1 | ENSP00000216714 | HAP-1, APE1, Ref-1, M99703, HAP1, APX, APEX nuclease, APE-1, h-APE-1, ref1, redox factor-1, redox factor 1, Ref 1, APEX1, APEX nuclease 1, APEX nuclease (multifunctional DNA repair enzyme) 1, apurinic-apyrimidinic endonuclease 1, apurinic apyrimidinic endonuclease 1, APE 1, apurinic/apyrimidinic endodeoxyribonuclease 1, 1dE9, hAPE1 |
| SETX | ENSP00000224140 | Sen1p, senataxin, AOA2, SETX, SCAR1, KIAA0625, AOA 2, AOA-2, Sen1 |
| CMA1 | ENSP00000250378 | chymase, MCT1, MCT-1, CMA1, chymase-1, chymase 1, mast cell protease I |
| CCNB1 | ENSP00000256442 | cyclin B1, cyclinB1, CCNB1, Ccnb, cyclin-B1, G2/mitotic-specific cyclin B1, G2/mitotic-specific cyclin-B1, G2/mitotic-specific cyclinB1, Ccnb-1 |
| SMAD7 | ENSP00000262158 | Smad7, Smad family, MADH7, Smad 7, mothers against decapentaplegic homolog 7, Smad-7, mothers against decapentaplegic homolog7, smad family member 7 |
| MSH3 | ENSP00000265081 | Msh3, dup, MRP1, hMSH3, MutS homolog 3, MutS homologs, mismatch repair protein 1, hMRP1, MRP-1, Msh3p, MRP1 p, MSH-3, MRP 1, FAP-4 |
| SLC5A1 | ENSP00000266088 | hSGLT1, SGLT1, SLC5A1, SGLT-1, sodium/glucose cotransporter 1, sodium/glucose cotransporter-1, nagt, sodium/glucose co-transporter-1, SGLT 1 |
| IQGAP1 | ENSP00000268182 | IQGAP1, Sar1, p195, IQ motif-containing GTPase-activating protein 1, Sar1p, IQ motif-containingGTPase-activatingprotein1, IQ motif containing GTPase activating protein 1, IQGAP-1, Ras GTPase-activating-like protein IQGAP1, IQ motif-containing GTPase activating protein 1, IQ motif containing GTPase-activating protein 1, IQ-motif containing GTPase activating protein 1 |
| CCNA2 | ENSP00000274026 | cyclin A2, Cyclin A, CCN1, cyclinA, cyclin-A, Ccna2, Ccn1p, cyclinA2, 4 CFU, CCNA, cyclin-A2, 4 BCP, Ccn 1, 3 EOC, 2 bpm |

| | | |
|---|---|---|
| SLC12A9 | ENSP00000275730 | Cip1, SLC12A9, cation-chloride cotransporter interacting protein, CIP-1 |
| KIT | ENSP00000288135 | c-kit, KIT, SCF-R, CD117, proto-oncogene c-kit, protooncogene c-kit, cKit, p145c-kit, SCFR, CD-117, CD 117, c- kit, tyrosine protein kinase Kit, v-kit Hardy-Zuckerman 4 feline sarcoma viral oncogene homolog, tyrosine-protein kinase Kit, KIT proto-oncogene receptor tyrosine kinase, mast/stem cell growth factor receptor KIT |
| RIPK3 | ENSP00000216274 | RIP3, RIPK3, receptor-interacting protein 3, receptor-interacting serine-threonine kinase 3, receptor interacting protein 3, RIP 3, receptor-interacting serine/threonine-protein kinase 3, receptor interacting serine/threonine kinase 3, RIPK-3, Receptor-interacting serine/threonine protein kinase-3, Receptor-interacting serine/threonine kinase 3, receptor interacting serine/threonine-protein kinase-3, receptor-interacting serine/threonine protein kinase 3, receptor-interacting protein-3, receptor-interacting serine/threonine-protein kinase-3 |
| TNFRSF10A | ENSP00000221132 | DR4, TNFRSF10A, TRAIL-R1, death receptor 4, APO2, death receptor-4, DR-4, TNF-related apoptosis inducing ligand receptor-1, DR 4, TRAILR1, tumor necrosis factor receptor superfamily, member 10a, Apo 2, TRAILR-1, TRAIL R1, tumor necrosis factor receptor superfamily member 10A, TNF receptor superfamily member 10a, TNF-Related Apoptosis-Inducing Ligand Receptor 1 |
| EPO | ENSP00000252723 | erythropoietin, Epoetin, Epo, Epo human |
| ENPP2 | ENSP00000259486 | autotaxin, PD-I alpha, ATX, Pdnp2, NPP2, PD-Ialpha, NPP-2, E-NPP2, lysoPLD, ENPP2, ecto-nucleotide pyrophosphatase/phosphodiesterase 2, extracellular lysophospholipase D, lyso PLD, Ectonucleotide Pyrophosphatase/Phosphodiesterase 2, lyso-PLD, ENPP-2, ectonucleotide pyrophosphatase/phosphodiesterase-2 |
| SULF1 | ENSP00000260128 | HSulf-1, SULF1, hSulf1, sulfatase 1, Sulf-1, sulfatase-1, sulfatase1 |
| DAPK2 | ENSP00000261891 | DRP-1, Drp1, DAPK2, death-associated protein kinase 2, death-associated protein kinases, 2A2A |
| TYR | ENSP00000263321 | tyrosinase, TYR, EC 1.14.18.1, CMM8, TYRL, oculocutaneous albinism I-A, EC 1.14.-18.1 |
| CERS2 | ENSP00000271688 | LASS2, CerS2, ceramide synthase 2, tmsg-1, LAG1 homolog, ceramide synthase 2, TMSG1, L3P, CerS-2 |

| | | |
|---|---|---|
| SKP2 | ENSP00000274255 | SKP2, S-phase kinase-associated protein 2, p45, F-box protein SKP2, F box protein SKP2, p45Skp2, Fbl1, S-phase kinase associated protein 2, S-phase kinase associated protein-2, S-phase kinase-associated protein-2, Skp-2, S phase kinase-associated protein 2, S phase kinase-associated protein2, SKP 2, S phase kinase associated protein 2, FBXL1, S-phase kinase-associated protein2 |
| IFNA1 | ENSP00000276927 | IFN-alpha, IFNalpha, IFN alpha, Ifna13, IFNA1, interferon alpha 1, interferon-alpha 1, Ifn, IFN -alpha, interferon alpha 1b, IFN- alpha, interferon-alpha1, interferon-alpha1b, IFN-alpha1b |
| PAK1 | ENSP00000278568 | PAK1, p21-Activated Kinase 1, p65PAK, PAK-1, alpha-PAK, PAK 1, p21-activated kinase-1, hPAK1, Pak1p, p-21-activated kinase 1, P21 protein (Cdc42/Rac)-activated kinase 1, P-21 activated kinase 1, p21 protein-activated kinase 1, p-21 activated kinase-1, P21 activated kinase-1, P21 activated kinase 1, P21-activated kinase1, 6b-16 |
| DUSP6 | ENSP00000279488 | DUSP6, MKP-3, Pyst1, MKP3, dual specificity phosphatase 6, dual-specificity phosphatase 6, mitogen-activated protein kinase phosphatase-3, mitogen-activated protein kinase phosphatase 3, dual specificity protein phosphatase 6, dual specificity phosphatase-6, dual-specificity protein phosphatase 6 |
| DENR | ENSP00000280557 | DRP-1, Drp1, density-regulated protein, drp, DENR, density regulated protein, protein DRP1, d RP, density-regulated re-initiation and release factor |
| ANK3 | ENSP00000280772 | ankyrin-G, ANK3, ankyrinG, ankyrin G, ankyrin3, Ankyrin 3, ankyrin 3, node of Ranvier, ankyrin-3 |
| PTH | ENSP00000282091 | PTH, parathyroid hormone, parathormone, PreproPTH, prepro-PTH, parathyroid hormone 1, preproparathyroid hormone, hPTH1, pTH-1, Pre-proparathyroid hormone, parathyroid-hormone, PTH1, PTH 1, pre-pro-PTH, P-TH, hPTH 1, parathyrin |
| MRAS | ENSP00000289104 | M-Ras, MRAS, R-ras3, muscle RAS oncogene homolog, RRAS3 |
| TRPM2 | ENSP00000381023 | TrpC7, LTRPC2, TRPM2, TRPM-2, long transient receptor potential channel 2, Transient receptor potential melastatin 2, transient receptor potential cation channel, subfamily M, member 2, TRPM 2, transient receptor potential melastatin-2, NUDT9-H, Transient receptor potential channel 7, transient receptor potential cation channel subfamily M member 2 |

| | | |
|---|---|---|
| MARK2 | ENSP00000385751 | PAR-1, PAR1, Par-1b, protein serine/threonine kinase, EMK1, ELKL Motif Kinase, serine/ threonine kinase, serine/threonine-kinase, protein-serine/threonine kinase, ELKL motif kinase 1, MARK2, Mark-2, Par 1, Serine/Threonine kinase, serine / threonine kinase, PAR1b, Microtubule affinity-regulating kinase 2, HEMK1, serine/threonine protein kinase MARK2 |
| SETD2 | ENSP00000386759 | HIF-1, HIP-1, HIP1, p231HBP, Huntingtin-interacting protein 1, SET2, SETD2, HYPB, Huntingtin interacting protein 1, HBP231, HSPC069, huntingtin-interacting protein-1, HIF1, SET domain containing 2, histone-lysine N-methyltransferase SETD2, huntingtin interacting protein B, SET domain-containing protein 2, Huntingtin interacting protein-1, SET-2, SET domain containing protein 2, KMT3A, 4H12, Set domain-containing 2, L-LS, SET-domain-containing 2 |
| NTF3 | ENSP00000397297 | NT-3, neurotrophin-3, Neurotrophin 3, NTF3, HDNF, NGF-2, NT3, nerve growth factor-2, nt -3, nerve growth factor 2, NTF-3, hNT-3, hNT3, neurotrophic-factor, NEUROTROPHIC FACTOR, NT 3 |
| CCN1 | ENSP00000398736 | cyr61, CCN1, GIG-1, cysteine-rich angiogenic inducer 61, Ccn1p, IGFBP-10, Cyr 61, protein CYR61, cysteine-rich, angiogenic inducer, 61, CYR-61, cysteine-rich, angiogenic-inducer, 61, Ccn 1, CCN family member 1, C ysteine-rich angiogenic inducer 61, Cysteine Rich Angiogenic Inducer 61, cysteine-rich angiogenic inducer-61 |
| SHC1 | ENSP00000401303 | Shc, p66, Shc1, SHCA, SHC-1, SHC-transforming protein 1, 1-shC, sh-C, SHC Adaptor Protein 1 |
| SLCO6A1 | ENSP00000421339 | GST, gonad-specific transporter, G St, OATP6A1, SLCO6A1, Organic Anion Transporting Polypeptide 6A1, CT48, HCT48 |
| GSTK1 | ENSP00000431049 | GST, hGSTK1, G St, GSTK1, GSTK1-1, Glutathione S-transferase kappa 1 |
| APOA5 | ENSP00000445002 | APOAV, APOA5, apo-AV, apolipoprotein A5, apolipoprotein A-V, apolipoprotein AV, apoA-V, APO A-V, Apo A5, hApoA5, APOA5 p, apolipoprotein-A5, Apo AV |

| | | |
|---|---|---|
| FPR2 | ENSP00000468897 | formyl peptide receptor-like 1, FPRL1, Fpr2, Rfp, ALXR, FPRL-1, formyl peptide receptor like-1, formylpeptide receptor-like-1, FPRH1, FPRH2, formyl peptide receptors, lipoxin A4 receptor, HM63, formyl-peptide receptor-like 1, Formyl peptide-receptor like-1, formyl peptide receptor-2, formylpeptide receptors, formyl-peptide-receptor like-1, formyl peptide receptor 2, formyl-peptide-receptor-like 1, formyl peptide receptor2, formyl-peptide receptor 2, Formyl-peptide Receptors, Formyl-peptide receptor-2, R-FP, N-formyl peptide receptor 2 |
| GRIN2B | ENSP00000477455 | NR2B, GRIN2B, GluN2B, NR3, NMDAR2B, hNMDAR2B, glutamate receptor, ionotropic, N-methyl-D-aspartate 2B, hNR2B, glutamate receptor, ionotropic, N-methyl- d-aspartate 2B, N-methyl D-aspartate receptor subtype 2B, N-methyl-D-aspartate receptor subtype 2B, NMDAR 2B, glutamate ionotropic receptor NMDA-type subunit 2B |
| TMEM37 | ENSP00000303148 | PR-1, hpr-1, Pr1, Hpr1, Tmem37, HP-R1 |
| UGT1A6 | ENSP00000303174 | UGT1A1, HUGT1, Gnt-1, UGT1, UDPGT, HlugP1, ugt1a6, GNT1, UGT-1, HLUG P1, UGT1-A1, hUGT1A1, UGT1 A1, UGT 1A1, UDP-glucuronosyltransferase 1A6, UGT-1A1, UDP glucuronosyltransferase 1A6, UGT1A 1 |
| CRMP1 | ENSP00000321606 | DRP-1, CRMP1, DRP1, CRMP-1, collapsin response mediator protein 1, Collapsin Response Mediator Protein-1, CRMP 1, collapsin response mediator Protein1, collapsin-response mediator protein 1, DPYSL1 |
| CYP2B6 | ENSP00000324648 | CYP2B6, P-450, CYP2B, IIB1, P-450p, hIIB1, cytochrome P4502B6, P450p, Cytochrome P450 2B6, 3 IBD, cytochrome P450 family 2 subfamily B member 6, 4R RT, CYP2B7 |
| NME1 | ENSP00000337060 | nm23-H1, NM23, granzyme A-activated DNase, awd, NDPK-A, NME1, nm-23-H1, nucleoside diphosphate kinase A, NM23H1, NDK-A, Nm-23, A-WD, NDPK A, NDKA, NM 23, nucleoside diphosphate kinase--a, nm-23H1, nucleoside-diphosphate kinase A, NDPKA, nucleoside diphosphate kinase-A, Nme-1, NME/NM23 nucleoside diphosphate kinase 1, gaad, NDP-K A, Nm23H-1, nm23 H-1, nm 23-H1, nm23 H1, NM-23 H1, nm23-H 1 |
| FGFR3 | ENSP00000339824 | fibroblast growth factor receptor 3, FGFR3, fibroblast growth factor-receptor 3, FGFR-3, FGFR 3, fibroblast growth-factor receptor 3, fibroblast growth factor receptor-3, fibro-blast growth factor receptor 3, FGF-R3, FGFR3 p, Fibroblast Growth Factor Receptor3, FGF-R-3, hFGFR3, Cek2 |

| | | |
|---|---|---|
| OCLN | ENSP00000347379 | occludin, OCLN |
| FAS | ENSP00000347979 | CD95, TNFRSF6, APO-1, APT1, tumor necrosis factor receptor superfamily, member 6, APO1, CD-95, Fas-1, APO-1 antigen, CD 95, Apo- 1, tumor necrosis factor receptor super family member 6, FAS1, TN-FRSF6, tumor necrosis factor receptor superfamily member 6, TNFRSF-6, FAS -1, TNF receptor superfamily member 6, Fas cell surface death receptor, Fas 1, APO-1-antigen, CD9 5 |
| DIRAS3 | ENSP00000360020 | ARHI, DIRAS3, NOEY2, AR HI, ras homolog gene family, member I, Distinct subgroup of the Ras family member 3, DIRAS family GTPase 3 |
| GADD45A | ENSP00000360025 | gadd45, GADD45a, GADD 45, growth arrest and DNA-damage-inducible, alpha, Growth arrest and DNA damage inducible, alpha, GADD-45, DDIT1, growth arrest and DNA-damage-inducible alpha, growth arrest and DNA damage inducible alpha |
| EDN2 | ENSP00000361668 | ET-2, endothelin 2, ET2, endothelin-2, EDN2 |
| DKK1 | ENSP00000363081 | hDkk-1, DKK1, dkk-1, dickkopf homolog 1, Dickkopf-related protein 1, dickkopf-homolog-1, Dickkopf-homolog 1, dickkopf 1 homolog, dickkopf WNT signaling pathway inhibitor-1, dickkopf WNT signaling pathway inhibitor 1, dickkopf related protein 1, Dickkopf-related-protein 1, dickkopf-related protein-1, hDKK1, Dick Kopf-related protein1 |
| MALAT1 | MALAT1 | MALAT-1, MALAT1, NEAT2 |
| RNU1-75P | U1 | HSD1, Hu1, HSD6, HSD7, Hu-1, HSD2, RNU1, HSD-1, HSD-2, HSD 1, HSD5, RNU12, HU11, HSD 2, HSD4 |
| IL31 | ENSP00000366234 | IL-31, Interleukin 31, interleukin-31, IL31 |
| CYBB | ENSP00000367851 | gp91-phox, CYBB, gp91phox, gp91 phox, NOX2, gp91-1, gp-91phox, gp 91phox, gp91- phox, NOX-2, NOX 2, NADPH oxidase 2, NADPH oxidase-2, cytochrome b-245 heavy-chain, NADPH oxidase2, NADPH-oxidase 2, Cytochrome b-245 heavy chain, Cytochrome B-245, Beta Polypeptide, cytochrome b-245 beta chain |
| KL | ENSP00000369442 | klotho, EC 3.2.1.31 |
| CDX2 | ENSP00000370408 | Cdx2, Cdx-2, CDX3, CDX-3, homeobox protein CDX2, caudal type homeobox transcription factor 2, caudal type homeo box transcription factor 2, caudal-type homeobox transcription factor 2, CDX2P, Caudal-type homeobox 2, caudal type homeobox 2, CDX2 human, homeobox protein CDX-2, caudal-type homeobox protein 2, Caudal-type homeobox-2 |

| | | |
|---|---|---|
| SIRT3 | ENSP00000372191 | hSIRT3, sirtuins, SIRT3, sirtuin, 3 Glu, Sirtuin 3, Sirtuin-3, SIRT 3, Sirtuin3, SIRT-3, SIRT3-p |
| IL24 | ENSP00000375795 | mda-7, Interleukin 24, IL-24, ST16, IL24, interleukin-24, MOB-5, c49a, mda7, Melanoma differentiation-associated gene-7 protein, IL- 24, hIL-24, hIL24, mda- 7, Il10b, interleukin- 24, IL-10-B, Hmda-7, IL-10B |
| SNAI2 | ENSP00000380034 | snail2, SNAI2, Slug, Slugh, snail homolog 2, zinc finger protein SNAI2, Snail 2, snail-2, snail family zinc finger 2, Snail Family Transcriptional Repressor 2, SNAI 2 |
| DYRK1A | ENSP00000381932 | DYRK1A, DYRK, dual-specificity tyrosine-(Y)-phosphorylation regulated kinase 1a, DYRK1, mnb, dual specificity tyrosine phosphorylation-regulated kinase 1A, dual-specificity tyrosine-phosphorylation-regulated kinase 1A, dual-specificity Yak1-related kinase, DYRK-1A, Dual-specificity tyrosine(Y)-phosphorylation regulated kinase 1A, dual-specificity tyrosine phosphorylation-regulated kinase 1A, dual-specificity tyrosine(Y)-phosphorylation-regulated kinase 1A, dual-specificity tyrosine-(Y) phosphorylation-regulated kinase 1A, dual-specificity tyrosine-(Y)-phosphorylation-regulated kinase 1A, dual-specificity tyrosine (Y)-phosphorylation regulated kinase 1A, dual-specificity tyrosine (Y) phosphorylation-regulated kinase 1A, Dual specificity tyrosine phosphorylation regulated kinase-1A, dual specificity tyrosine-phosphorylation-regulated kinase 1A, dual specificity tyrosine (Y) phosphorylation regulated kinase 1A, Dual-specificity tyrosine-(Y)-phosphorylation-regulated kinase 1 A, Dual-Specificity Tyrosine-Phosphorylation Regulated Kinase 1A, dual-specificity tyrosine phosphorylation-regulated kinase-1A, dual specificity tyrosine phosphorylation regulated kinase 1A, Dual specificity tyrosine phosphorylation regulated kinase1A, dual-specificity tyrosine phosphorylation regulated kinase 1A, dual specificity tyrosine phosphorylation-regulated kinase 1 A |
| MAP3K1 | ENSP00000382423 | MEKK-1, MEKK1, MAP3K1, MEKK, MAPK/ERK kinase kinase 1, mitogen-activated protein kinase kinase kinase 1, MEKK-1-p |
| APLN | ENSP00000391800 | apelin, Apln |
| DCTN4 | ENSP00000414906 | p62, dynactin subunit p62, dynactin 4, DCTN4, dynactin-4, p-62, dynactin subunit 4 |
| CDK5 | ENSP00000419782 | Cdk5, Cyclin-dependent kinase 5, Cdk-5, cdk 5, Cyclin-dependent kinase-5, cyclin dependent kinase-5, Cyclin dependent kinase 5, cyclin-dependent kinase5, Cyclin- dependent kinase 5 |

| | | |
|---|---|---|
| TIPARP | ENSP00000420612 | RM-1, Tiparp, PARP-1, poly(ADP-ribose) polymerases, PARP1, EC 2.4.2.30, DKFZp434J214, poly (ADP-ribose) polymerases, Rm1, poly (ADP ribose) polymerases, PARP 1, TCDD-inducible poly(ADP-ribose) polymerase, DDF1, PARP-7, TCDD-inducible poly(ADP-ribose)polymerase, PARP7, ARTD14 |
| RPS6KA2 | ENSP00000427015 | Rsk3, RSK, HU-2, Hu2, RPS6KA2, p90 Rsk-3, p90RSK2, ribosomal S6 kinase 3 |
| PEAK1 | ENSP00000452796 | PEAK1, SgK269, KIAA2002, Pseudopodium-enriched atypical kinase 1, Sugen kinase 269, Pseudopodium enriched atypical kinase 1 |
| WWOX | ENSP00000457230 | WWOX, FRA16D, WOX1, WW domain-containing oxidoreductase, WW domain containing oxidoreductase, fragile site FRA16D oxidoreductase, WW-domain containing oxidoreductase, WW-domain-containing oxidoreductase, WWdomain-containing oxidoreductase, SCAR12, EIEE28 |
| NUP62 | ENSP00000471191 | p62, NUP62, p-62, nucleoporin 62 kDa, nucleoporin Nup62, nuclear pore glycoprotein p62, nucleoporin 62 |
| TARDBP | ENSP00000478249 | TDP-43, TARDBP, TDP43, TDP 43, hTDP-43, TDP-43 p, TDP- 43, hTDP43 |
| YAP1 | ENSP00000478927 | YAP65, YAP, YAP1, yes-associated protein 1, Yki, YAP2, yes-associated protein-1, transcriptional coactivator Yap1, YAP-1, hYAP1, yes associated protein 1, yes-associated-protein-1, Yes-associated protein1 |
| TSC2 | ENSP00000219476 | TSC2, Tuberin, TSC-2, TSC complex, TS- c2, TSC2 p, TSC 2, TSC2p, TSC complex subunit 2, lam |
| CCL13 | ENSP00000225844 | MCP-4, Scyl1, chemokine ligand 13, NCC-1, monocyte chemotactic protein-4, monocyte-chemotactic protein-4, monocyte chemoattractant protein-4, CCL13, CCL-13, monocyte chemoattractant protein 4, MCP4, CCL1-3, chemokine Ligand-13 |
| ULBP1 | ENSP00000229708 | ULBP1, RAET1I, ULBP-1, UL16-binding protein-1, UL16-binding protein 1, ULBP 1, UL16 binding protein 1 |
| EIF2AK2 | ENSP00000233057 | PKR, p68 kinase, EIF2AK2, PRKR, PK-R, EIF2AK1, interferon-inducible RNA-dependent protein kinase, Protein Kinase RNA-activated, protein kinase-RNA activated, interferon-induced, double-stranded RNA-activated protein kinase, EIF2AK1 p |

| | | |
|---|---|---|
| TARDBP | ENSP00000240185 | TDP-43, TAR-DNA-binding protein, TARDBP, TAR DNA-binding protein, TAR DNA binding protein, TAR DNA-binding protein 43, TDP43, TAR DNA binding protein-43, TAR-DNA binding protein, TAR DNA binding protein 43, TAR DNA-binding protein-43, TAR-DNA-binding protein 43, TAR-DNA-binding protein-43, TAR-DNA binding protein 43, TDP- 43, TAR-DNA binding protein-43, TAR DNA-bindingprotein 43, TDP 43, TARDNA-binding protein 43, hTDP-43, TDP, TDP-43 p, hTDP43, TAR DNA -binding protein |
| IL17C | ENSP00000244241 | IL-21, IL-17C, IL21, C-X2, Interleukin 17C, Interleukin-17C, CX2, IL-21, IL17C, IL 21, IL2 1 |
| SLC22A5 | ENSP00000245407 | OCTN2, SLC22A5, Solute carrier family 22 member 5, solute carrier family 22, member 5, hOCTN2, organic cation/carnitine transporter 2, organic cation/carnitine transporter-2, scd, Cdsp |
| FGL2 | ENSP00000248598 | Fgl2, pT49, Fibroleukin, fibrinogen-like protein 2, HFGL2, hpT49, fibrinogen-like 2, fibrinogen like protein 2, Fibrinogen-like protein-2, FGL 2 |
| SESN2 | ENSP00000253063 | Hi95, Hypoxia-induced gene, Sestrin2, SESN2, sestrin 2, Ses2, hypoxia induced gene, sestrin-2 |
| RFX1 | ENSP00000254325 | RFX, RFX1, RF-X, enhancer factor C, transcription factor RFX1, RFX-1, Regulatory factor X1, Regulatory factor X-1, regulatory factor X 1 |
| BRD4 | ENSP00000263377 | CaP, BRD4, bromodomain-containing 4, 5 CRM, chromosome-associated protein, bromodomain-containing protein 4, 4 mEq, 4 MEN, bromodomain containing 4, bromodomain containing protein 4, bromodomain-containing protein-4, bromodoma in-containing protein 4, bromodomaincontaining protein 4, BRD 4, M-CAP, chromosome associated protein |
| GTF2H1 | ENSP00000265963 | p62, TFIIH, BTF2, GTF2H1, p-62, Tfb1, general transcription factor IIH, polypeptide 1 |
| FGF7 | ENSP00000267843 | FGF7, KGF, keratinocyte growth factor, FGF-7, fibroblast growth factor 7, fibroblast growth factor-7, HBGF-7, heparin-binding growth factor-7, FGF 7 |
| UCN | ENSP00000296099 | UCN, urocortin, UroC |
| MMP19 | ENSP00000313437 | MMP19, MMP-19, MMP-18, matrix metalloproteinase 19, Matrix metalloproteinase-19, RasI, matrix metalloproteinase RASI, RASI-1 |

| | | |
|---|---|---|
| KHDRBS1 | ENSP00000313829 | Sam68, p68, p62, KHDRBS1, GAP-associated tyrosine phosphoprotein p62, p-62, HP68, KH domain containing, RNA binding, signal transduction associated 1, KH domain-containing, RNA-binding, signal transduction-associated 1 |
| MX2 | ENSP00000333657 | Hmx2, MX2, MxB, MX dynamin-like GTPase 2, Myxovirus Resistance Protein 2 |
| PKLR | ENSP00000339933 | PKR, PKLR, PKL, PK-R, PK-LR, RPK, EC 2.7.1.40, PK L, PK-L, PK R, PKRL, HPK1, R-PK, Pyruvate kinase L/R |
| SPAG8 | ENSP00000340982 | HSD1, SMP1, hSMP-1, HSD-1, HSD 1, SPAG8 |
| PTGES | ENSP00000342385 | mPGES, mPGES1, MGST1-L1, PIG12, PGES, prostaglandin E synthase, Microsomal prostaglandin E synthase-1, mPGES-1, microsomal prostaglandin E synthase 1, 4 bpm, microsomal glutathione S-transferase 1-like 1, PTGES, microsomal prostaglandin-E synthase-1, prostaglandin-E synthase |
| CD5 | ENSP00000342681 | CD5, HT1, Leu-1, Leu1, HT-1, CD5 molecule, cd 5, Leu1p, CD5 antigen, HT 1, Leu 1, T1P, CD5-antigen |
| FYN | ENSP00000346671 | fyn, SLK, Syn, p59fyn, Src family tyrosine kinases, src-like kinase, Src-family tyrosine kinases, FYN oncogene related to SRC, FGR, YES, tyrosine protein kinase Fyn |
| JPT1 | ENSP00000348316 | HN-1, HN1, hematological and neurological expressed 1, Arm2, Jupiter microtubule-associated homolog 1, JPT1 |
| AKR1B10 | ENSP00000352584 | Arl1, ARL-1, aldoketoreductases, aldo-keto reductases, hARL1, small intestine reductase, Arl1p, AKR1B10, aldose reductase-like-1, Aldo-keto reductase family 1, member B10, Aldo-keto reductase family 1 member B10, aldoketo reductases, H Is, Aldo-ketoreductase family 1 member B10 |
| BSCL2 | ENSP00000354032 | Gng3lg, BSCL2, Seipin, Berardinelli-Seip congenital lipodystrophy 2, Berardinelli Seip Congenital Lipodystrophy 2 |
| SREBF2 | ENSP00000354476 | SREBP-2, sterol regulatory element-binding protein 2, sterol regulatory element-binding protein-2, SREBP2, sterol regulatory element binding protein 2, SREBF2, sterol regulatory element binding protein-2, SREBP 2, sterol-regulatory element binding protein 2, sterol regulatory element binding transcription factor 2, Sterol-regulatory-element-binding protein 2, sterol-regulatory element-binding protein-2, sterol-regulatory element-binding protein 2, SREBF-2, sterol regulatory element-binding transcription factor 2 |

| | | |
|---|---|---|
| PLA2G4A | ENSP00000356436 | cytosolic phospholipase A2, PLA2g4a, cPLA2, cPLA2-alpha, cPLA2alpha, cytosolic phospholipase-A2, Phospholipase A2, Group IVA, cPLA2 alpha, PLA2G4, Phospholipase A2 Group IVA, cytosolic phospholipaseA2 |
| S100A16 | ENSP00000357693 | S100A16, S100 calcium binding protein A16, S100 calcium-binding protein A16 |
| S100A2 | ENSP00000357697 | S100A2, protein S100A2, S100 calcium binding protein A2, S100 A2, CaN19, S100-A2, S-100A2 |
| HRNR | ENSP00000357791 | hornerin, S100A16, HRNR |
| LET7C5P | hsa-let-7c-5p | let-7c, hsa-let-7c, let7c, let- 7c, hsa-let-7c-5p, let-7c-5p, let7c-5p, let-7-c |
| ELP1 | ENSP00000363779 | IKAP, IKBKAP, ELP-1, Elp1p, p150, Elongator complex protein 1, IkappaB kinase complex-associated protein, IkappaB kinase complex associated protein, Elp1, Dys, hELP1, inhibitor of kappa light polypeptide gene enhancer in B cells, kinase complex-associated protein, inhibitor of kappa light polypeptide gene enhancer in B-cells, kinase complex-associated protein, HEL-P1 |
| SLC22A6 | ENSP00000367102 | rOAT1, hOAT1, PAH transporter, OAT1, organic anion transporter 1, SLC22A6, organic anion transporter-1, organic anion transporter1 |
| PCDH17 | ENSP00000367151 | PCDH17, Protocadherin 17, PCH68, Protocadherin-17, PCDH-17, Protocadherin17, Nonclustered Protocadherins |
| CA9 | ENSP00000367608 | carbonic anhydrase IX, CA IX, CA9, CAIX, EC 4.2.1.1, carbonic anhydrase 9, renal cell carcinoma-associated antigen G250, carbonic anhydrase-9, carbonic dehydratase, CA-IX, CA-9, hCA9, carbonic anhydrase-IX |
| GLRX | ENSP00000369314 | thioltransferase, Grx, glutaredoxin, GLRX, Grx1, glutaredoxin (thioltransferase, Glutaredoxin 1, thiol transferase, glutaredoxin-1, GRX-1, hGrx1, glutaredoxin1 |
| HRH1 | ENSP00000380247 | H1R, Histamine receptor H1, HRH1, histamine H1 receptor, histamine H1-receptor, HH1R, HRH-1 |
| GSTP1 | ENSP00000381607 | GstP, GSTP1, GST-P, GST3, GST 3, GST-3, GSTP1-1, glutathione S-transferase P, 18 GS, GST P, GST P1, hGSTP1, glutathione-S-transferase p, GST P1-1, GST-P1, glutathione-S-transferase pi1, glutathione s-transferase Pi1, GSTP-1, glutathione S-transferase Pi 1, glutathione S-transferase Pi-1, Glutathione-S-transferase pi 1, GSTP1 p, 20 GS, glutathione-S-transferase-P, glutathione S-transferase-P, 5 GSS, glutathione S transferase P |

| | | |
|---|---|---|
| MGAT5 | ENSP00000386377 | N-acetylglucosaminyltransferase V, GnT-V, Mgat5, GnT-VA, EC 2.4.1.155, GlcNAcT V, N-Acetylglucosaminyltransferase-V, N-acetylgluco-saminyltransferase V, N-acetylglucosaminyl transferase V, N-acetyl glucosaminyltransferase V, Mgat-5, Mannosyl (alpha-1, 6-)-glycoprotein beta-1, 6-N-acetyl-glucosaminyltransferase, GlcNAc-TV |
| CD8A | ENSP00000386559 | CD8, MAL, p32, Leu-2, Leu2, CD8A, hp32, 1 CD8, CD 8, m AL, CD-8, P3 2, Leu 2, 1CD8 |
| IGF2 | ENSP00000391826 | IGF-II, IGF2, insulin-like growth factor 2, Insulin-like growth factor II, IGF-2, insulin-like growth factor-2, IGF II, insulin like growth factor-II, insulin like growth factor II, insulin-like growth factor-II, somatomedin-A, Somatomedin A, insulin-like growth-factor 2, IGFII, 1gf2, IGF 2, insulin-like-growth factor II, insulin-like-growth-factor-II, insulinlike growth factor II, insulin-like growth-factor-II, Insulin like growth factor 2, preptin, insulin-like growth factor -II, Insulinlike Growth Factor 2, insulin like growth factor-2, insulinlike growth factor-II |
| BZW2 | ENSP00000397249 | BZW2, Basic leucine zipper and W2 domains 2 |
| TRIM48 | ENSP00000402414 | Tripartite Motif-Containing 48, TRIM48 |
| MUC15 | ENSP00000416753 | MUC15, PAS3, PAS 3, PAS-3, MUCIN 15 |
| DUSP7 | ENSP00000417183 | MKPX, DUSP7, MKP-X, Pyst2, Dual-specificity phosphatase 7 |
| CEBPA | ENSP00000427514 | C/EBP alpha, CEBPA, C/EBPalpha, CCAAT/enhancer binding proteins, CCAAT/enhancer-binding protein alpha, CCAAT/enhancer binding protein alpha, C/EBP-alpha, CCAAT/enhancer-binding proteins, CCAAT/enhancer binding protein-alpha, CCAAT/enhancer-binding protein-alpha, CCAAT/ enhancer binding protein alpha, CEBP, CCAAT/enhancer binding protein (C/EBP), alpha, CCAAT/enhancer binding protein A, CCAAT/enhancer-binding-protein-alpha, C-EBP |
| HSF1 | ENSP00000431512 | HSF1, heat shock transcription factor 1, HSF-1, heat-shock transcription factor 1, heat shock transcription factor-1, HSTF1, HSF 1, heat-shock transcription factor-1, HSTF-1, heat shock transcription factor1, hHSF1, heat shock factor protein 1, H-stf1 |
| MTRNR2L1 | ENSP00000439228 | HN-1, HN1 |

| | | |
|---|---|---|
| SLC16A1 | ENSP00000441065 | MCT1, MCT-1, SLC16A1, monocarboxylate transporter 1, monocarboxylate transporter-1, solute carrier family 16, member 1, m-CT, monocarboxylate transporter1, mCt, solute carrier family 16 member 1, MCT 1 |
| CITED2 | ENSP00000444198 | P35srj, CITED2, Mrg1, cited-2, Cbp/p300-interacting transactivator, with Glu/Asp-rich carboxy-terminal domain, 2, Cbp/p300-interacting transactivator with Glu/Asp-rich carboxy-terminal domain 2, Cbp/p300-interacting transactivator with Glu/Asp-rich carboxy-terminal domain-2, Cited 2 |
| NDRG2 | ENSP00000451712 | NDRG2, hNDRG2, NDRG family member 2 |
| CIB1 | ENSP00000479860 | calmyrin, cib, CIB1, Kip1, KIP, CIB-1, KIP 1 |
| GPIHBP1 | ENSP00000480053 | HBP1, GPI-HBP1, GPIHBP1, glycosylphosphatidylinositol-anchored high-density lipoprotein-binding protein 1, glycosylphosphatidylinositol-anchored high density lipoprotein-binding protein 1, high-density lipoprotein-binding protein 1, glycosylphosphatidylinositol-anchored high-density lipoprotein binding protein 1, GPIHBP-1, glycosyl-phosphatidylinositol anchored high-density lipoprotein-binding protein 1, HBP-1, glycosylphosphatidylinositol anchored high density lipoprotein binding protein 1, glycosylphosphatidylinositol anchored high-density lipoprotein-binding protein 1, high density lipoprotein binding protein 1, high-density lipoprotein binding protein 1, GPI-anchored high-density lipoprotein-binding protein 1 |
| GATD3B | ENSP00000480313 | Hes1, HES 1, Hes-1, Es-1, GT335, Es1, C21orf33 |
| CD24 | ENSP00000483985 | CD24, CD24a, signal transducer CD24, CD24 antigen, CD24 molecule, CD24- human, CD 24 antigen |
| MYOC | ENSP00000037502 | myocilin, MYOC, GLC1A, Trabecular meshwork Inducible Gluco-corticoid Response protein, tigr, trabecular meshwork-induced glucocorticoid response protein, Trabecular meshwork Inducible Glucocorticoid Response Protein, trabecular meshwork-inducible glucocorticoid response protein, trabecular meshwork induced glucocorticoid response protein |

| | | |
|---|---|---|
| TGFB1 | ENSP00000221930 | DPD1, transforming growth factor beta1, transforming growth factor-beta1, Tgfb1, transforming growth factor beta-1, Transforming growth factor-beta 1, transforming growth factor beta 1, transforming-growth-factor-beta 1, transforming growth factorbeta1, Tgfb-1, C-E-D, CE-D, transforming growth-factor-beta1, transforming growth factor-beta-1, transforming growth factor-beta 1, transforming growth-factor-beta 1, TGF-b1, TGF-B 1, Transforming-growth factor beta1, transforming growth factor-beta1, transforming growth factor, beta-1, Transforming growth factor-B1, CeD, transforming growth factor B1, transforming growth factor, beta 1, Transforming-growth-factor beta 1 |
| CSF3 | ENSP00000225474 | G-CSF, granulocyte colony-stimulating factor, GCSF, granulocyte-colony-stimulating factor, filgrastim, granulocyte colony stimulating factor, granulocyte-colony stimulating factor, CSF3, granulocyte colonystimulating factor, lenograstim, granulocyte colony-stimulating factor, Granulocyte colony-stimulating-factor, CSF-3, colony-stimulating factor 3, granulocyte- colony-stimulating factor, granulocyte- colony stimulating factor, granulocyte-colony-stimulating-factor |
| NAGLU | ENSP00000225927 | alpha-N-acetylglucosaminidase, NAGLU, nAG, EC 3.2.1.50, Sanfilippo disease IIIB |
| GMNN | ENSP00000230056 | Gem, Geminin, GMNN, geminin, DNA replication inhibitor |
| HES1 | ENSP00000232424 | Hhl, Hes1, Hes-1, HRY, Hes 1, transcription factor HES-1, hairy and enhancer of Split1, hairy and enhancer of split-1, Hairy and Enhancer of Split 1, HES1 human, hes family bHLH transcription factor 1, transcription factor Hes1 |
| PROC | ENSP00000234071 | Apc, protein C, protein-C, Anticoagulant protein C, ProC, Vitamin K-dependent protein C, anti-coagulant protein C, Pro-C, 2 PCT |
| RPN2 | ENSP00000237530 | Rpn2, RPN II, ribophorin II, Rpn-2, RPNII, hRpn2, SWP1, Ribophorin-II |
| MAPK8IP3 | ENSP00000250894 | JSAP1, MAPK8IP3, JIP3, c-Jun-amino-terminal kinase-interacting protein 3, SYD |
| CMPK2 | ENSP00000256722 | UMP/CMP kinase, UMP-CMPK2, CMPK2, TYKi, nucleoside diphosphate kinase, EC 2.7.4.14, nucleoside-diphosphate kinase, NDK, UMP/CMP-kinase |

| | | |
|---|---|---|
| UBE2S | ENSP00000264552 | ubiquitin-protein ligase, E2 ubiquitin-conjugating enzymes, ubiquitin carrier proteins, ubiquitin protein ligase, EPF5, ubiquitin carrier protein, E2-EPF, ubiquitin-carrier protein, ubiquitin-conjugating enzyme E2S, UBE2S, ubiquitin-conjugating enzyme E2 S |
| PXN | ENSP00000267257 | paxillin, 3 GM1, paxil-lin, PXN |
| RAB5A | ENSP00000273047 | Rab5, Rab5A, rab-5, Ras-related protein Rab-5A |
| ST14 | ENSP00000278742 | matriptase, epithin, St14, MT-SP1, HAI, ARCI11, SNC19, St 14, membrane-type serine protease 1, suppression of tumorigenicity-14, PRSS14, membrane type serine protease 1, TADG-15, Membrane-type serine protease-1, suppression of tumorigenicity 14, serine protease 14, membrane type-serine protease 1 |
| STC1 | ENSP00000290271 | STC1, Stanniocalcin-1, Stanniocalcin 1, stc, STC-1, hSTC-1, STC 1, Stanniocalcin1, hSTC1 |
| MCAT | ENSP00000290429 | MCT1, MCAT, MCT-1, M-CAT, m-CT, malonyl-CoA-acyl carrier protein transacylase, mCt |
| GATD3A | ENSP00000291577 | Hes1, HES-1, Es-1, KNP-I, ES1, GT335, protein KNP-I, HES 1, ES1 protein homolog, ES1 protein homolog,  mitochondrial, ES-1 human, C21orf33 |
| CLDN1 | ENSP00000295522 | CLDN1, claudin-1, SEMP1, claudin 1, CLD1, senescence-associated epithelial membrane protein 1, CLDN-1, claudin1, CLDN 1, Cld-1 |
| IGFBP7 | ENSP00000295666 | TAF, mac25, IGFBP7, mac25 protein, IGFBP-7, prostacyclin-stimulating factor, IGFBP-rP1, insulin-like growth factor binding protein-7, insulin-like growth factor binding protein 7, mac 25, prostacyclin stimulating factor, Insulin-like growth factor binding-protein-7, IGFBPrP1, T-A-f, Insulin-like growth factor-binding protein 7, Insulin-like growth factor-binding protein-7, P-SF, Insulin like growth factor binding protein 7, insulin- like growth factor-binding protein 7, Insulinlike growth factor-binding protein 7, insulin-like growth factor binding protein7, IGFBP 7, RAMSVPS, PGI2-stimulating factor |
| KCNK3 | ENSP00000306275 | KCNK3, TASK-1, OAT1, hOAT1, TASK1, K2P3.1, Potassium Channel Subfamily K Member 3, potassium two-pore-domain channel subfamily K member 3, potassium two-pore domain channel subfamily K member 3 |

| | | |
|---|---|---|
| ADAM17 | ENSP00000309968 | ADAM17, TNF-alpha convertase, TNF-alpha-converting enzyme, TNF-alpha converting enzyme, TNFalpha convertase, TNFalpha-converting enzyme, TNF alpha converting enzyme, ADAM 17, ADAM-17, snake venom-like protease, cSVP, TNF-alpha- converting enzyme, tACE, hADAM17, CD156b, ADAM metallopeptidase domain 17, disintegrin and metalloproteinase domain-containing protein 17, TNF alpha-converting enzyme |
| HNRNPA1 | ENSP00000341826 | hnRNP A1, hnRNPA1, heterogeneous nuclear ribonucleoprotein A1, UP1, HNRPA1, HNRP A1, hnRNP-A1, hnRNPA 1, single-strand RNA binding protein, helix-destabilizing protein |
| ATG7 | ENSP00000346437 | Gsa7, Atg7, autophagy-related 7, Gsa7p, ubiquitin-activating enzyme E1-like protein, ubiquitin-activating enzyme-E1-like protein, Atg-7, autophagy-related protein 7, Atg 7, autophagy related 7 |
| HTT | ENSP00000347184 | huntingtin, htt, IT15, HD human, IT-15, Huntington disease protein |
| IQGAP3 | ENSP00000354451 | IQGAP3, IQ Motif Containing GTPase-Activating Protein 3, IQ motif-containing GTPase activating protein 3 |
| PSEN2 | ENSP00000355747 | pS2, PSEN2, presenilin 2, E5-1, STM2, presenilin-2, HPS2, pS-2, HPS-2, PS -2, AD4, Ps 2, PSEN-2, AD5, presenilin2, HAd5, PSEN2 p, Presenilin- 2, HAD-4, e51 |
| KISS1 | ENSP00000356162 | KiSS1, KiSS-1, kisspeptin, KiSS-1 metastasis suppressor, KISS1 metastasis suppressor, kisspeptin-1, metastasis suppressor KiSS-1, metastasis suppressor KISS1, KiSS-1 metastasis-suppressor, Kisspeptin 1, KISS1 metastasis-suppressor |
| LMNA | ENSP00000357283 | lamin A/C, LMNA, LGMD1B, lamins, lamin-A/C, Pro-1, LMN1, laminA/C, iDC, limb girdle muscular dystrophy 1b, prelamin-A/C, prelamin A/C, Limb-Girdle muscular Dystrophy 1B, fPL |
| YY1AP1 | ENSP00000357323 | YAP, YY1AP1, HCCA2, HCCA1, GRN g, GrnG, YY1-associated protein 1, YY1 associated protein 1 |
| DMBT1 | ENSP00000357905 | gp-340, DMBT1, gp340, glycoprotein-340, Deleted in Malignant Brain Tumors 1, hensin, Muclin, deleted in malignant brain tumors-1, DMBT-1, Salivary agglutinin, deleted in malignant brain tumors 1 protein, glycoprotein 340, salivary scavenger and agglutinin |
| MCTS1 | ENSP00000360365 | MCT1, MCT-1, multiple copies in T-cell lymphoma-1, MCTS1 |

| | | |
|---|---|---|
| LGI1 | ENSP00000360472 | Epitempin, LGI1, leucine-rich glioma-inactivated 1, leucine-rich, glioma-inactivated 1, leucine-rich,  glioma inactivated 1, Leucine-rich Glioma Inactivated-1, LGI-1, leucine-rich glioma inactivated 1, Leucine-rich glioma-inactivated1, leucine-rich glioma inactivated protein 1, leucine-rich glioma-inactivated protein 1, LGI 1 |
| GHRH | ENSP00000362716 | GHRH, growth-hormone-releasing hormone, growth hormone-releasing hormone, growth hormone-releasing factor, GHRF, growth hormone releasing hormone, growth hormone releasing factor, grf, somatocrinin, GH-RH, growth-hormone releasing hormone, Growth Hormone - Releasing Hormone, growth-hormone-releasing factor |
| SPDEF | ENSP00000363149 | SPDEF, PDEF, SAM pointed domain-containing ETS transcription factor, prostate epithelium-specific ets transcription factor, prostate-derived Ets factor, pSE, prostate derived Ets factor, Sam-pointed domain containing Ets transcription factor, SAM pointed domain containing ETS transcription factor, SAM-pointed domain-containing ETS transcription factor |
| VRK1 | ENSP00000216639 | PCH-1, PCH1, VRK1, VRK-1, vaccinia-related kinase 1, PCH1a |
| CYP27B1 | ENSP00000228606 | CYP27B1, 1alpha-(OH)ase, 25-hydroxyvitamin D3-1alpha-hydroxylase, hCyP1, cytochrome p450 27B1, CYP1, P450c1, 25-hydroxyvitamin D3 1alpha-hydroxylase, v DD 1, 1alpha(OH)ase, 25-hydroxyvitamin d(3)-1 alpha-hydroxylase, 25-hydroxyvitamin D(3)-1alpha-hydroxylase, VDD1, 25-hydroxyvitamin D3-1 alpha-hydroxylase, Vdr, 1-alpha(OH)ase, 25-OHD 1 alpha-hydroxylase, 25-hydroxyvitamin D3 1 alpha-hydroxylase, 25-OHD-1 alpha-hydroxylase, CYP1alpha, 25-hydroxy-vitamin D(3)-1alpha-hydroxylase, CYP 27B1, 25-hydroxyvitamin D(3) 1alpha-hydroxylase, 25-hydroxyvitamin D(3) 1-alpha-hydroxylase, 25-hydroxyvitamin D31alpha-hydroxylase, 25-hydroxyvitamin D(3)1alpha-hydroxylase, cytochrome P450c1A, 25-hydroxyvitamin-D(3)-1-alpha-hydroxylase, 25-hydroxyvitamin D(3 )1alpha hydroxylase, 25-OHD-1alpha-hydroxylase, 25-hydroxyvitamin D(3)-1-alpha hydroxylase, 25-hydroxyvitamin-D3 1alpha-hydroxylase, cytochrome P450,  family 27,  subfamily B,  polypeptide 1, CYP27B, CYP27B1 p, 25-hydroxyvitamin D3 1-alpha-hydroxylase, cytochrome P450 family 27 subfamily B member 1, 1 alpha(OH)ase |

| | | |
|---|---|---|
| LNPEP | ENSP00000231368 | P-LAP, CaP, oxytocinase, IRAP, placental leucine aminopeptidase, aminopeptidases, insulin-responsive aminopeptidase, PLAP, OTase, insulin-regulated membrane aminopeptidase, insulin-responsive amino peptidase, Cystinyl aminopeptidase, cystinyl-aminopeptidase, amino peptidases, LNPEP, Insulin Responsive Amino Peptidase, leucyl/cystinyl aminopeptidase, amino-peptidases |
| CTSD | ENSP00000236671 | cathepsin D, cathepsin S, cathepsins, CTSD, EC 3.4.23.5, cathepsin-D, cathepsinD, 1LYB, cathepsin-S |
| EREG | ENSP00000244869 | epiregulin, EREG |
| BMP4 | ENSP00000245451 | bone morphogenetic protein-4, BMP-4, bone morphogenetic proteins, Bmp4, Bone morphogenetic protein 4, hBmp4, Zyme, bmp2b, Bmp-2b1, BMP-2B, OFC11, bone morphogenetic proteins, Bone-morphogenetic proteins, BMP 4, Bmp, BMP4-p, Bone morphogenetic protein4 |
| RFPL3 | ENSP00000249007 | RFPL3 |
| RRM2B | ENSP00000251810 | p53R2, RRM2B, EC 1.17.4.1, ribonucleoside-diphosphate reductase subunit M2B |
| APC | ENSP00000257430 | Apc, DP2, adenomatous polyposis coli protein, Adenomatous polyposis coli, adenomatosis polyposis coli, DP3, DP2.5, M 74088, Adenomatous Polyposis Coli-protein, Ade-nomatous polyposis coli, DP-2, adenomatous-polyposis coli, Dp-3, adenomatous polyposis coli, HDP2, Adenomatous-Polyposis-Coli, APC, WNT signaling pathway regulator |
| KDR | ENSP00000263923 | KDR, Flk1, VEGFR2, Flk-1, VEGFR-2, vascular endothelial growth factor receptor 2, kinase insert domain receptor, Fetal liver kinase 1, VEGF-R2, VEGF R2, vascular endothelial growth factor receptor-2, fetal liver kinase-1, vascular endothelial growth factor-receptor 2, hVEGFR2, 2 Met, VEGFR 2, kinase-insert domain receptor, K-D-R, Vascular endothelial growth factor receptor-2, FLK 1, 4 ASD, VEGF R 2, kinase insert-domain receptor, vascular-endothelial growth factor receptor 2, CD309, 4AG8, 4 as E |
| PRDX5 | ENSP00000265462 | Plp, AOEB166, PRDX5, Peroxiredoxin 5, antioxidant enzyme B166, peroxiredoxin-5, acr1, Peroxiredoxin V, PRXV, Prdx 6, Prdx6, peroxiredoxin 5, Acr-1, peroxisomal antioxidant enzyme, P-LP, hPRDX5, Prx V, Peroxiredoxin5 |

| | | |
|---|---|---|
| TIMP3 | ENSP00000266085 | TIMP-3, metalloproteinase inhibitor 3, Timp3, tissue inhibitor of metalloproteinases-3, tissue inhibitor of metalloproteinase-3, Tissue Inhibitor of Metalloproteinase 3, hTIMP-3, tissue inhibitor of metalloproteinases 3, tissue inhibitor of metalloproteinases- 3, TIMP metallopeptidase inhibitor 3, TIMP 3, hTIMP3 |
| RB1 | ENSP00000267163 | RB1, pRB, pp110, Rb-1, p105-RB, retinoblastoma 1, p105Rb, Rb 1, retinoblastoma-1, OS-RC, p-Rb, PR-B, PR_b, retinoblastoma-associated protein, OSRC, retinoblastoma1, RB transcriptional corepressor 1, p105 Rb |
| HAPLN1 | ENSP00000274341 | CrT1, Hcrt-1, CRTL1, CRTL-1, CRT-1, proteoglycan link protein, cartilage link protein, HAPLN1, hyaluronan and proteoglycan link protein 1, Hcrt1 |
| IL9 | ENSP00000274520 | Interleukin-9, IL-9, IL9, Interleukin 9, T-cell growth factor P40, p40 cytokine, HP40 |
| HOXA10 | ENSP00000283921 | Hoxa-10, HOXA10, HOX1, HOX-1, Hox 1, Hox 1H, Hoxa 10, homeobox A10, Homeo box A10, HomeoboxA10 |
| WNT7A | ENSP00000285018 | Wnt7a, WNT family, Wnt-7a, Wnt-family, Wnt 7a, wingless-type MMTV integration site family,  member 7A, protein Wnt7a |
| CBR1 | ENSP00000290349 | Crn, Cbr, carbonyl reductase 1, CBR1, CBR 1, carbonyl reductase1 |
| NOTCH4 | ENSP00000364163 | Notch4, int-3, Int3, Notch 4, Notch-4 |
| CUL4A | ENSP00000364589 | CUL-4A, CUL4A, cullin 4A, cullin-4A, Cullin4A, hCUL4A |
| POFUT1 | ENSP00000364902 | HDDD2, pofut1, Protein O-fucosyltransferase 1, Protein O-fucosyltransferase-1 |
| TP73 | ENSP00000367545 | p73, TP73, p53-related protein, 2 MPS, tumor protein p73, P73P |
| ASAH1 | ENSP00000371152 | acid ceramidase, EC 3.5.1.23, acid-ceramidase, ASAH1, N-acylsphingosine amidohydrolase 1, N-acylsphingosine amidohydrolase, acylsphingosine deacylase, pHP, A-CDase, aCDase, ASAH 1 |

| | | |
|---|---|---|
| G6PD | ENSP00000377192 | G6PD, glucose-6-phosphate dehydrogenase, G-6-PD, glucose 6-phosphate dehydrogenase, G-6PD, glucose-6-phosphate-dehydrogenase, EC 1.1.1.49, glucose 6 phosphate dehydrogenase, glucose- 6-phosphate dehydrogenase, glucose-6- phosphate dehydrogenase, glucose-6 phosphate dehydrogenase, hG6PD, glucose-6-phosphate 1-dehydrogenase, G6 PD, G 6-PD, Glucose-6 -Phosphate dehydrogenase, glucose -6 phosphate dehydrogenase, 2BH9, Glucose-6-Phosphate Dehy-drogenase, G6-PD, glucose 6-phosphatedehydrogenase, G6PD p, G-6-P-D |
| GPT | ENSP00000378408 | AAT-1, alanine-aminotransferase, Gpt-1, Gpt, GPT1, glutamic-pyruvate transaminase, alanine aminotransferase, glutamic-pyruvic transaminase, ALT1, glutamic-pyruvic transaminase-1, AAT1, GPT 1, glutamic pyruvic transaminase, ALT-1, alanine amino transferase, Alanine amino-transferase, glutamic pyruvate transaminase |
| ARHGAP24 | ENSP00000378611 | p73, Arhgap24, RC-GAP72, FilGAP, FLJ33877, p73RhoGAP, AK091196, DKFZp564- B1162, Rho GTPase-activating protein 24, P73P, RHO GTPase activating protein 24 |
| PTK2B | ENSP00000380638 | Pyk2, proline-rich tyrosine kinase 2, 3 GM1, FAK2, related adhesion focal tyrosine kinase, PYK-2, RAFTK, Cell adhesion kinase beta, proline-rich tyrosine kinase-2, PTK2B, pTK, CadTK, proline-rich tyrosine kinase2, focal adhesion kinase 2, protein tyrosine kinase 2 beta, Protein tyrosine kinase 2beta, proline rich tyrosine kinase 2, p-TK, 3-GM3, Pyk 2 |
| KDM1A | ENSP00000383042 | LSD1, BHC110, lysine-specific demethylase 1, KDM1, KDM1A, KIAA0601, AOF2, lysine-specific demethylase-1, lysine demethylase 1a, lysine-specific demethylase1, LSD-1, Lysine specific demethylase 1, lysine specific demethylase-1, lysine (K)-specific demethylase 1A, Lysine specific demethylase1, lysine-specific histone demethylase 1A, lysine-specific histone demethylase-1A |
| ESRRA | ENSP00000384851 | ERRalpha, ERR alpha, ESRL1, Estrogen-related receptor alpha, NR3B1, ESRRA, ERR-alpha, estrogen receptor-like 1, ERR1, Estrogen-related receptor-alpha, 3-D24, Estrogen related receptor alpha, Estrogen-related-receptor alpha |

| | | |
|---|---|---|
| GLI2 | ENSP00000390436 | Gli2, Thp, pHS2, Thp2, GLI-2, PHS-2, GLI-Kruppel family member GLI2, GLI family zinc finger 2, Th-p, Gli 2, GLI family zinc finger protein 2, glioma-associated oncogene family zinc finger 2 |
| HERPUD1 | ENSP00000409555 | Mif1, Herpud1, KIAA0025, HERP, MIF-1, HERPUD 1, homocysteine-inducible, endoplasmic reticulum stress-inducible, ubiquitin-like domain member 1 |
| STMN1 | ENSP00000410452 | stathmin, PP17, Lap18, Op18, leukemia-associated phosphoprotein p18, metablastin, stathmin 1, stathmin-1, oncoprotein 18, STMN1, LAg, phosphoprotein p19, oncoprotein18, stathmin1, LAP 18, oncoprotein-18, STMN-1, Pr22 |
| CNBP | ENSP00000410769 | ZNF9, Hdm2, CNBP, zinc finger protein 9, Cellular Nucleic Acid Binding Protein, cellular nucleic acid-binding protein, CCHC-type zinc finger, nucleic acid binding protein, HDM-2, CCHC-type zinc-finger nucleic acid-binding protein, Dm2 |
| HFE | ENSP00000417404 | HFE, Hh, HLA-H, hereditary hemochromatosis protein, HFE Human |
| FHIT | ENSP00000417480 | Fhit, FRA3B, fragile histidine triad, fragile histidine triad protein, fragile histidine triad gene, EC 3.6.1.29, FRA 3B, FHT, 1-FIT |
| GRIA1 | ENSP00000428994 | GluR1, HBGR1, GluA1, GLUH1, GRIA1, Glur-A, GluRA, glutamate receptor 1, glutamate receptor, ionotropic, AMPA 1, glutamate ionotropic receptor AMPA type subunit 1, glutamate receptor-1 |
| APPL2 | ENSP00000446917 | FLJ10659, APPL2, DCC-interacting protein 13-beta, adaptor protein, phosphotyrosine interacting with PH domain and leucine zipper 2 |
| NR1H4 | ENSP00000447149 | 5q14, FXR, 5q13, 5q12, bile acid receptor, Nr1h4, farnesoid X receptor, farnesoid-X-receptor, 5q11, RIP14, 5q15, 3-DCT, 5-q14, farnesoid X-activated receptor, farnesoid X activated receptor, farnesoid-X receptor, 3D-CT, 3D CT, nuclear receptor subfamily 1 group H member 4, HRR1, 3DCT, farnesoid X-receptor, far-nesoid X receptor, 3-D CT |
| SHANK2 | ENSP00000469689 | CortBP1, SHANK2, ProSAP1, proline-rich synapse-associated protein-1, SHANK, cortactin-binding protein-1, cortactin-binding protein 1, cortactin binding protein 1, pro-SAP-1, proline-rich synapse-associated protein 1, SH3 and multiple ankyrin repeat domains protein 2, SH3 and multiple ankyrin repeat domains 2 |

| | | |
|---|---|---|
| DYRK1B | ENSP00000469863 | mirk, DYRK1B, minibrain-related kinase, Mirk protein kinase, minibrain related kinase, dual specificity tyrosine-phosphorylation-regulated kinase 1B, miR-K, dual-specificity tyrosine phosphorylation-regulated kinase 1B, Dual-Specificity-Tyrosine-Phosphorylation-Regulated Kinase 1B, dual specificity tyrosine phosphorylation-regulated kinase 1B, dual-specificity tyrosine-(Y)-phosphorylation regulated kinase 1B |
| UHRF1 | ENSP00000479617 | ICBP90, Np95, UHRF1, ubiquitin-like with PHD and RING finger domains 1, inverted CCAAT box-binding protein of 90 kDa, Inverted CCAAT box Binding Protein of 90 kDa, 3 ASK, ubiquitin-like with PHD and ring-finger domains 1, Transcription factor ICBP90, ubiquitin-like PHD and ring finger domain-containing protein 1, ubiquitin like with PHD and ring finger domains 1, Nuclear protein 95, ubiquitin-like with PHD and RING finger domains-1 |
| RPS6KA5 | ENSP00000479667 | MSK1, MSK -1, MSK-1, RPS6KA5 |
| ACACA | ENSP00000483300 | acetyl-CoA carboxylase 1, ACACA, ACC1, ACC-alpha, acetyl-CoA carboxylase-alpha, acetyl-CoA carboxylase alpha, acc, EC 6.4.1.2, ACC-1, ACCalpha, acetyl-CoA-carboxylase-alpha, acetyl coenzyme A carboxylase alpha, acetyl-coenzyme A carboxylase alpha, ACCA, acetyl-Co A carboxylase-1, Ac-c, acetyl co-A carboxylase alpha, acetyl-CoA carboxylase-1, acetyl CoA carboxylase 1, acetyl-CoA carboxylase1 |
| SERPINB6 | ENSP00000484343 | CaP, PI-6, cytoplasmic antiproteinase, SERPINB6, pI6, placental thrombin inhibitor, SPI3, serpin B6 |
| MIR1265P | hsa-miR-126-5p | miR-126*, MiR-126-5p, hsa-mir-126*, hsa-miR-126-5p, miR126-5p |
| MIR1323P | hsa-miR-132-3p | miR132, miR-132, hsa-miR-132, hsa-miR-132-3p, miR-132-3p, miR132-3p |
| MIR151A3P | hsa-miR-151a-3p | miR-151, hsa-miR-151, miR-151-3p, MIR151, miR-151a-3p |
| MIR27A3P | hsa-miR-27a-3p | miR-27a, Hsa-mir-27a, miR-27a-3p, miR- 27a, miR27a, hsa-miR27a, miR27a-3p, hsa-miR-27a-3p, miR 27a |

| | | |
|---|---|---|
| CEBPB | ENSP00000305422 | C/EBPbeta, NF-IL6, CCAAT/enhancer-binding protein beta, CEBPB, CCAAT/enhancer binding proteins, C/EBP-beta, C/EBP beta, transcription factor 5, TCF5, NF-IL-6, CCAAT/enhancer binding protein beta, CCAAT/enhancer-binding proteins, CCAAT/enhancer-binding protein-beta, Interleukin-6-dependent DNA-binding protein, liver-enriched transcriptional activator protein, Crp2, CCAAT/enhancer binding protein-beta, NFIL6, nuclear factor NF-IL6, liver activator protein, liver enriched transcriptional activator protein, liver enriched inhibitory protein, liver-enriched inhibitory protein, IL-6DBP, NF-IL 6, CCAAT/enhancer-binding-protein-beta, CCAAT/enhancer binding protein B, CCAAT/enhancer-binding protein B, NFIL-6 |
| TERT | ENSP00000309572 | TERT, telomerase catalytic subunit, telomerase reverse transcriptase, TCS-1, TP2, hEST2, EST2, Telomerase reverse-transcriptase, 5 meq, hTRT, telomerase catalytic sub-unit, 5 MEN, TCS1, TERT human, Telomerase-reverse-transcriptase, Telomerase-reverse transcriptase |
| SOCS3 | ENSP00000330341 | SOCS3, SOCS-3, suppressors of cytokine signaling, suppressor of cytokine signaling-3, SOCS, suppressor of cytokine signaling 3, CIS3, SOCS 3, suppressor of cytokine signaling -3, suppressor of cytokine signaling3, suppressors of cytokine-signaling |
| GAS6 | ENSP00000331831 | Gas6, growth arrest specific 6, Axl stimulatory factor, Gas-6, growth arrest-specific 6, growth arrest-specific protein 6, Growth arrest specific protein 6, Gas 6, growth arrest specific-6, growth-arrest specific 6, Growth-arrest-specific protein 6, growth-arrest specific protein 6, AXL receptor tyrosine kinase ligand, hGAS6, growth-arrest-specific 6 |
| BSG | ENSP00000333769 | CD147, Bsg, basigin, tumor cell-derived collagenase stimulatory factor, EMMPRIN, extracellular matrix metalloproteinase inducer, Ok blood group antigen, CD 147, M6p, TCSF, extracellular matrix metallo-proteinase inducer, collagenase stimulatory factor, CD-147, Tumor cell-derived collagenase-stimulatory factor |
| GHRL | ENSP00000335074 | GHS, Ghrelin, obestatin, growth hormone secretagogue, GHRL, growth hormone-releasing peptide, growth hormone releasing peptide, GHRP, appetite-regulating hormone, MTLRP, growth-hormone secretagogue, Ghrelin and Obestatin Prepropeptide |

| | | |
|---|---|---|
| ANO1 | ENSP00000347454 | FLJ10261, Tmem16a, ANO1, ORAOV2, dog-1, transmembrane protein 16A, anoctamin 1, DOG1, Ano 1, TAOS2, Anoctamin-1, Anoctamin1, Anoctamins |
| NTRK3 | ENSP00000354207 | TrkC, NTRK3, gp145trkC, neurotrophic tyrosine kinase receptor type 3, TRK-C, Trk C, NTRK-3, neurotrophic tyrosine kinase, receptor, type 3, neurotrophic receptor tyrosine kinase 3, trk-C tyrosine kinase |
| HACD1 | ENSP00000355308 | Ptpla, CaP, HACD1, 3-hydroxyacyl-CoA dehydratase 1, protein-tyrosine phosphatase-like member-a, cementum attachment protein |
| DACT2 | ENSP00000355760 | DAPPER2, Dpr2, DACT2, DACT-2 |
| HEY2 | ENSP00000357348 | grl, Hey2, Hrt2, CHF1, HERP1, HERP, Hairy-related transcription factor 2, hesr2, HEY 2, Hey-2, Hairy/enhancer-of-split related with YRPW motif protein 2 |
| IRAK1 | ENSP00000358997 | IRAK, IRAK-1, IRAK1, interleukin-1 receptor associated kinase-1, pelle, interleukin-1 receptor-associated kinase-1, interleukin-1 receptor-associated kinase 1, Interleukin 1 receptor-associated kinase 1, interleukin-1 receptor associated kinase 1, interleukin-1-receptor-associated kinase 1, IRAK 1, interleukin 1 receptor associated kinase 1 |
| SORBS1 | ENSP00000360293 | CaP, SORBS1, sorbin and SH3-domain-containing-1, sorbin and SH3 domain containing 1, ponsin, 2DL3, c-Cbl-associated protein, SH3P12, R85FL, Sorbin and SH3 domain-containing protein 1 |
| ENTPD8 | ENSP00000360561 | E-NTPDase, NTPDase8, ecto-nucleoside triphosphate diphosphohydrolase 8, ENTPD8, EC 3.6.1.5 |
| CAP1 | ENSP00000361883 | CaP, CAP1, CAP-1, adenylate cyclase-associated protein 1, Adenylyl cyclase-associated protein-1, Adenylyl cyclase-associated protein 1, C AP1, Cap1p |
| GDF5 | ENSP00000363492 | CDMP1, cartilage-derived morphogenetic protein 1, GDF5, CDMP-1, growth differentiation factor 5, Bmp14, hCDMP-1, Growth/differentiation factor 5, growth/differentiation factor-5, cartilage-derived morphogenetic protein-1, Gdf-5, cartilage derived morphogenetic protein-1, Growth differentiation factor-5, GDF 5, OS 5, SYNS2, BMP-14, bone morphogenetic protein-14, growth differentiation factor5, SYM1B |
| PTGER1 | ENSP00000292513 | EP1, Ptger1, Hep-1, EP-1, prostaglandin E receptor 1, Hep1, Hep 1, prostanoid EP1 receptor |
| CXCL3 | ENSP00000296026 | GRO3, chemokine ligand 3, CXCL3, GRO-3, HMIP-2B |

| | | |
|---|---|---|
| IGFBP6 | ENSP00000301464 | insulin-like growth factor binding protein 6, IGFBP-6, insulin-like growth factor binding protein-6, insulin-like growth factor-binding protein-6, IGFBP6, IGF-BP6, insulin-like growth factor-binding protein 6, IGF BP-6, Insulin-like Growth Factor Binding Protein6 |
| AQP1 | ENSP00000311165 | aquaporin-1, AQP1, aquaporin 1, AQP-CHIP, CHIP-28, CHIP28, aquaporin CHIP, aquaporin-CHIP, AQP-1, hAQP1, aquaporin1 |
| BOK | ENSP00000314132 | BCL2 family, bcl-2 family, Bcl-2-family, Bcl-2-related ovarian killer, BCL2-family, Bcl-2-related ovarian killer protein, BOK, Bcl 2-family, Bcl-2 related ovarian killer, Bcl2-Related Ovarian Killer |
| VIPR1 | ENSP00000327246 | Vipr1, RDC-1, RDC1, HVR1, VPAC1, vasoactive intestinal peptide receptor family, HVR 1, HVR-1, VPAC1-R, VPAC1R, VPAC1 R, VirG, VPCAP1-R, VPAC-1, vasoactive intestinal peptide receptor 1, 3 HCV, 3-DTX, VIP-R1, 5 DEG, VAPC1, Vasoactive intestinal polypeptide receptor 1, VIPR, vasoactive intestinal peptide receptor-1, VIP-R |
| F3 | ENSP00000334145 | tissue factor, thromboplastin, 1 boy, coagulation factor III, tissue-factor, 3 Th2, TF-A, CD142, 1 TFH, TFa, 2 - CEH, HF 3, HF3 |
| ENOX2 | ENSP00000337146 | tumor-associated NADH oxidase, ENOX2, ecto-NOX disulfide-thiol exchanger 2 |
| SPRED2 | ENSP00000348753 | Spred-2, Spred2 |
| COPS5 | ENSP00000350512 | JAB1, Mov-34, CSN5, CSN-5, Jun activation-domain binding protein 1, Mov 34, Sgn5, Jun activation domain-binding protein 1, JAB-1, Mov34, Jun activation domain binding protein 1, Jun-activation-domain-binding protein 1, COP9 signalosome subunit 5, 4D10, COPS5, CSN 5, Jun activation domain binding protein-1, jun activation domain-binding protein-1, COP9 Signalosome Complex Subunit 5 |
| TFEB | ENSP00000351742 | TFEB, transcription factor EB, Tcfeb, Alpha-TFEB, AlphaTFEB |
| PIK3CG | ENSP00000352121 | PI3Kgamma, PIK3CG, PI3K gamma, p110gamma, p110 gamma, 3 pre, 3 CSF, 3 APC, PIK3, PI3-Kgamma, PI3CG, PI3K-p, PI3K p, PI3K-gamma |
| EMP2 | ENSP00000352540 | EMP2, Epithelial membrane protein-2, EMP-2, epithelial membrane protein 2, EMP 2 |
| ETS2 | ENSP00000353344 | Ets2, ETS-2, ets 2, v-ets erythroblastosis virus E26 oncogene homolog 2, V-ets erythroblastosis virus E26 oncogene homolog2, V-Ets avian erythroblastosis virus E26 oncogene homolog 2, Protein C-ets-2 |

| | | |
|---|---|---|
| MT-CO1 | ENSP00000354499 | COX I, COX-1, COI, COX1, EC 1.9.3.1, CO I, CO-1, CO1, cytochrome c oxidase subunit I, MTCO1, COX 1, hCox-1, cytochrome c oxidase I, COXI, cytochrome C oxidase-subunit 1, cytochrome c oxidase subunit 1, cytochrome c oxidase subunit-I, MT-CO1, COX-I, Mitochondrially Encoded Cytochrome C Oxidase I, COX- 1, Cytochrome c oxidase subunit-1, cytochrome c-oxidase subunit I |
| PTGS1 | ENSP00000354612 | COX-1, PGHS-1, Pes1, COX1, prostaglandin-endoperoxide synthase 1, PTGS1, cyclooxygenase-1, COX-3, PCOX-1, prostaglandin H2 synthase-1, PGG/HS, prostaglandin-endoperoxide-synthase-1, hCox-1, COX 1, cyclo-oxygenase-1, cyclooxygenase 1, PHS-1, PGHS1, EC 1.14.99.1, pcox1, prostaglandin endoperoxide synthase 1, COX3, prostaglandin G/H synthase 1, prostaglandin-endoperoxide synthase-1, COX- 1, PHS1 |
| CCN6 | ENSP00000357655 | WISP3, CCN6, Wnt1 inducible signaling pathway protein 3, WISP-3, LIBC, Wnt1 inducible signaling pathway protein-3, Wnt-1-inducible signaling pathway protein 3, p-PD, Wnt1-inducible signaling pathway protein 3, WNT1-inducible-signaling pathway protein 3, WNT1-inducible-signaling pathway protein3, WNT1-inducible signaling pathway protein-3 |
| MLLT11 | ENSP00000357917 | AF-1q, AF1q, ALL1-fused gene from chromosome 1q, MLLT11 |
| EIF3A | ENSP00000358140 | p185, p180, p167, eIF3a, eIF3-p170, KIAA0139, EIF3S10, p 180, eIF3 p170, Eukaryotic translation initiation factor 3 subunit A, eukaryotic translation initiation factor 3,  subunit A |
| HMGCS2 | ENSP00000358414 | 3-hydroxy-3-methylglutaryl coenzyme A synthase, HMGCS2, 3-hydroxy-3-methylglutaryl-coenzyme A synthase, hHMGCS2, 3-hydroxy-3-methylglutaryl-CoA synthase 2, 3-hydroxy-3-methylglutaryl CoA synthase-2 |
| BRS3 | ENSP00000359682 | BRS-3, bombesin receptor subtype-3, BB3, Bombesin receptor subtype 3, bombesin-like receptor-3, Bombesin-like receptor 3, Brs3 |
| NIBAN2 | ENSP00000362409 | MEG3, FAM129B, MEG 3, MEG-3, Family with sequence similarity 129,  member B |
| TRAF1 | ENSP00000362994 | TRAF1, TNF receptor-associated factors, EBI6, TNF-receptor associated factors, TRAF-1, TNF receptor-associated factor 1, TNF receptor-associated factor-1, TRAF, TNF Receptor Associated Factors, TNF receptor associated factor 1, TNF-receptor-associated factors |

| | | |
|---|---|---|
| ILRUN | ENSP00000363135 | C6orf106, Chromosome 6 open reading frame 106 |
| AGER | ENSP00000364210 | receptor for advanced glycation end products, receptor for advanced glycation end-products, receptor for advanced glycation endproducts, receptor for advanced glycosylation end products, receptor for advanced glycosylation endproducts, AGE-R, AGER, Receptor for advanced-glycation end products, advanced glycosylation end product-specific receptor, receptor for advanced glycation end-products, receptor for advanced glycation end-products, receptor for advanced-glycation-end-products, receptor for advanced glycation-end products, Receptor-for-Advanced-Glycation-End-products, advanced glycosylation end-product specific receptor |
| ARHGEF7 | ENSP00000364893 | p50, p85, PAK3, beta-Pix, Cool-1, betaPix, ARHGEF7, SH3 domain-containing proline-rich protein, p85SPR, P 5 0, 1 by 1, p85Cool-1, HP 50, Rho guanine nucleotide exchange factor 7 |
| HNRNPK | ENSP00000365439 | heterogeneous nuclear ribonucleoprotein K, hnRNP K, hnRNP-K, HNRNPK, CSBP, HNRPK |
| EDNRB | ENSP00000366416 | Ednrb, ET-B, ETB-R, ETRB, endothelin receptor type B, ETB, ETBR, ET B, ET-RB, ETB1, ETR-B, ET-BR, ET BR, Endothelin Receptor Type-B |
| MAOB | ENSP00000367309 | monoamine oxidase B, MAOB, MAO B, MAO-B, EC 1.4.3.4, monoamine oxidase type B, monoamine oxidase-B, monoamine-oxidase B, monoamine oxidase -B, monoamine oxidase type-B |
| SLC23A2 | ENSP00000368637 | SVCT2, Slc23a1, hSVCT2, YSPL2, sodium-dependent vitamin C transporter 2, SLC23A2, SVCT-2, solute carrier family 23 member 2 |
| POSTN | ENSP00000369071 | periostin, OSF-2, Osf2, osteoblast-specific factor-2, Osteoblast-specific factor 2, osteoblast specific factor 2, POSTN |
| FGF9 | ENSP00000371790 | Fgf9, Glia-activating factor, FGF-9, fibroblast growth factor 9, fibroblast growth factor-9, FGF 9 |
| SEMA5A | ENSP00000371936 | semaphorins, Semaphorin 5A, Sema5A, semF, semaphorin F, Semaf, semaphorin5A, Sema 5A, Semaphorin-5A |
| SERPINB5 | ENSP00000372221 | maspin, SERPINB5, PI5, serpin B5, serpin family B member 5 |

| | | |
|---|---|---|
| AKT2 | ENSP00000375892 | AKT2, protein kinase AKT2, PKBbeta, PKB beta, Akt 2, protein kinase Bbeta, Akt-2, PKB-beta, V-Akt Murine Thymoma Viral Oncogene Homolog 2, AKT serine/threonine kinase 2, Protein Kinase B Beta, protein kinase B-beta, 2 X 39 |
| CIAPIN1 | ENSP00000377914 | Anamorsin, CIAPIN1, cytokine-induced apoptosis inhibitor 1, cytokine induced apoptosis inhibitor 1 |
| DAAM1 | ENSP00000378557 | DAAM1, Dishevelled-associated activator of morphogenesis 1, dishevelled associated activator of morphogenesis 1, Daam-1, disheveled associated activator of morphogenesis 1 |
| FAF1 | ENSP00000379457 | hFAF1, Faf1p, FAF1, Fas associated factor 1, Fas-associated factor 1, Fas-associated factor-1, FAF-1 |
| JAK3 | ENSP00000391676 | Jak3, JAK 3, Janus kinase 3, L-JAK, leukocyte Janus kinase, Jak-3, janus kinase-3, 3LXK |
| PTPN13 | ENSP00000394794 | PTP-BL, PTP-Bas, PTPN13, PTPL1, hPTP1E, FAP 1, FAP-1, FAP1, protein-tyrosine phosphatase PTPL1, PTP1E, protein tyrosine phosphatase,  non-receptor type 13, protein tyrosine phosphatase PTPL1, tyrosine-protein phosphatase non-receptor type 13, PTPLE, PTPBAS, 3 PDZ, PTPN 13 |
| RHOA | ENSP00000400175 | RhoA, Rho family GTPases, rho cDNA clone 12, Rho-family GTPases, Ras homolog gene family,  member A, rho A, AR-H12, Rho-A, ras homolog family member A, Ras-Homolog-Family-Member-A |
| UBE2L3 | ENSP00000400906 | UbcH7, E2F-1, E2F1, UBE2L3, L-UBC, E2-F1, ubiquitin-conjugating enzyme E2L 3, Ubiquitin-conjugating enzyme E2L3, Ubiquitin-conjugating enzyme E2 L3, ubiquitin conjugating enzyme E2L3, Ubiquitin conjugating enzyme E2 L3 |
| MYD88 | ENSP00000401399 | MyD88, TIR domain-containing, myeloid differentiation primary response gene, TIR-domain-containing, hMyD88, TIR domain containing, myeloid differentiation primary response 88, MyD-88, Myd88p, Myd 88, myeloid differentiation primary response protein MyD88, TIRdomain-containing, MYD88 p |
| POLD1 | ENSP00000406046 | cdc2, POLD, POLD1, POLD1 p, EC 2.7.7.7, cdc2 homolog, cdc-2, cdc 2, poly-merase, DNA polymerase delta catalytic subunit, poly merase |

| | | |
|---|---|---|
| FBP1 | ENSP00000408025 | FBP1, Fbp, fructose-1, 6-bisphosphatase 1, fructose-1, 6-bisphosphatases, EC 3.1.3.11, fructose-1,  6-bisphosphatase 1, FBP-1, Fructose-1, 6-bisphosphatase-1, liver FBPase, fructose 1, 6-bisphosphatase 1, fructose-bisphosphatase 1, fructose bisphosphatase 1 |
| ADGRL3 | ENSP00000422533 | Lphn3, Latrophilin-3, LEC3, cl3, ADGRL3, CIRL-3, CL-3, latrophilin 3, latrophilin3, Adhesion G protein-coupled receptor L3 |
| ANGPT1 | ENSP00000428340 | Ang1, AGP-1, angiopoietin-1, Ang-1, Agpt, ANGPT1, Angiopoietin 1, Ang 1, Angiopoietin1, hAng1, angpt-1, ANGPT 1 |
| CNTN1 | ENSP00000447006 | contactin-1, cntn1, contactin 1, glycoprotein Gp135, Gp135, CNTN-1, contactin1, HF 3, HF3 |
| APAF1 | ENSP00000448165 | Apaf-1, Ced-4, Apaf1, apoptotic protease-activating factor-1, apoptotic protease activating factor-1, CED4, apoptotic protease activating factor 1, apoptotic protease-activating factor 1, Apaf 1, apoptotic protease activating factor, apoptotic peptidase activating factor 1, apoptotic-protease-activating factor-1, Apaf, apoptotic peptidase activating factor-1 |
| SLC16A2 | ENSP00000465734 | MCT8, Monocarboxylate transporter 8, SLC16A2, monocarboxylate transporter-8, DXS128E, Xpct, DXS128, X-linked PEST-containing transporter, SLC 16A2, solute carrier family 16, MCT 8, solute carrier family 16 member 2, hMCT8 |
| RPS19 | ENSP00000470972 | RPS19, ribosomal protein S19, RPS-19, RPS 19, 40S ribosomal protein S19, RP S19 |
| CCL5 | ENSP00000474412 | RANTES, CCL5, beta-chemokine RANTES, Scya5, CCL-5, chemokine ligand 5, chemokine (C-C motif) ligand 5, C-C motif chemokine 5, CC motif chemokine ligand 5, C-C motif chemokine ligand 5, chemokine ligand5, HCCL5, small inducible cytokine A5, CCL 5 |
| H3-2 | ENSP00000476960 | histone H3, histone-H3, histoneH3 |
| PRLR | ENSP00000482954 | PRLR, prolactin receptor, hPRL, PRL-R, prolactin-receptor, h-PRL |
| GRN | ENSP00000053867 | Progranulin, granulin, PCDGF, PGRN, granulin precursor, GP88, granulins, CCDS11483.1, Grn, proepithelin, pEPI, gep, Pep-I |
| HEATR6 | ENSP00000184956 | ABC1, amplified in breast cancer 1, hABC1, AbC-1, hABC-1, amplified in breast cancer-1, FLJ22087, Amplified-in-breast cancer 1, HEATR6 |

| | | |
|---|---|---|
| XBP1 | ENSP00000216037 | TREB5, XBP-1, XBP1, X-box binding protein 1, X-box-binding protein-1, X-box-binding protein 1, X box-binding protein 1, hXBP-1, X-box binding protein-1, hXBP1, X box binding protein 1, x box-binding protein-1, X box binding protein-1, X-box-binding-protein-1, X-box binding protein1, X-box- binding protein-1 |
| IL1RL1 | ENSP00000233954 | HT1, ST2, hst-2, ST2L, Hst2, TIR domain containing, IL33R, ST2V, Fit-1, HT-1, FIT1, TIR-domain-containing, IL-33R, IL1RL1, der4, ST-2, HT 1, interleukin-1 receptor-like 1, IL-1RL-1, TIR domain-containing, IL-1RL1, interleukin-1 receptor like 1, interleukin 1 receptor-like 1, Interleukin-1-receptor-like-1, TIRdomain-containing, T1P, interleukin 1 receptor like 1 |
| TWIST1 | ENSP00000242261 | twist-1, Twist1, H-twist, TWIST 1, TWIST homolog 1, class A basic helix-loop-helix protein 38, bHLHa38, Twist-related protein 1, twist family bHLH transcription factor 1, CRS1, twist basic helix-loop-helix transcription factor 1, hTWIST1, CRS-1 |
| GNRH2 | ENSP00000245983 | GnRH-II, GnRH II, GnRH2, gonadotropin-releasing hormone 2, LHRH-II, GnRHII, progonadoliberin-2 |
| PLVAP | ENSP00000252590 | Plvap, plasmalemma vesicle-associated protein, Plasmalemma Vesicle Associated Protein, fenestrated endothelial-linked structure protein, gp68 |
| TTC9 | ENSP00000256367 | TTC9, tetratricopeptide repeat domain 9 |
| MARK4 | ENSP00000262891 | MARKL1, MAP/microtubule affinity-regulating kinase-like 1, MARK4, MAP/microtubule affinity-regulating kinase 4, MARK4L, MARK4S, Microtubule affinity-regulating kinase 4, microtubule-affinity regulating kinase 4, Microtubule Affinity Regulating Kinase 4, Microtubule Affinity-Regulating Kinase4 |
| PLD2 | ENSP00000263088 | hPLD2, PLD2, phospholipase D2, phospholipaseD2, Phospholipase D-2 |
| APLP2 | ENSP00000263574 | APLP2, CDEBP, APPL2, APLP-2, APP-H |

| | | |
|---|---|---|
| EPCAM | ENSP00000263735 | Ep-CAM, TACSTD1, EPCAM, MK1, KS1/4, Trop-1, epithelial cell-surface antigen, CD326, GA733-2, epithelial cell adhesion molecule, epithelial cell surface antigen, adenocarcinoma-associated antigen, epithelial glycoprotein, TROP1, M4S1, tumor-associated calcium signal transducer 1, EGP314, EGP-2, MOC31, EGP40, MK-1, EsA, EGP2, HEA-125, EGP-314, MOC-31, epithelial-cell adhesion-molecule, epithelial cell-adhesion molecule, EpCAM- human, epithelial-cell adhesion molecule, CD 326, 323/A3 |
| NRP1 | ENSP00000265371 | NP-1, NRP1, NP1, neuropilin-1, neuropilin1, HNP-1, BDCA-4, neuropilin 1, HNP1, Nrp-1, VEGF165R, Nrp, HNP 1, CD304, BDCA4, NRP- 1 |
| MIR1555P | hsa-miR-155-5p | miR-155, hsa-miR-155, Mir155, miR-155-5p, miR -155, miR- 155, Hsa-miR-155-5p, MiR155-5p |
| MIR203A | hsa-miR-203a | miR-203, hsa-miR-203, MIR203, miR-203a, hsa-miR-203a, miR203a |
| MIR2213P | hsa-miR-221-3p | mir-221, miR 221, mir221, hsa-miR-221, miR-221-3p, miR- 221, miR221-3p, hsa-miR-221-3p |
| MIR5083P | hsa-miR-508-3p | miR-508-3p, miR-508, miR508-3p |
| MIR5743P | hsa-miR-574-3p | MiR-574-3p, hsa-miR-574-3p, miR-574 |
| MIR613 | hsa-miR-613 | miR-613 |
| MEG3 | MEG3 | Gtl2, MEG3, Meg3P, MEG 3, MEG-3, MEG3_2 |
| LET7B5P | hsa-let-7b-5p | let-7b, let7b, hsa-let-7b, Let-7b-5p, hsa-let-7b-5p, hsa-let7b-5p, let 7b, let7b-5p, let- 7b, let7-b |
| MIR107 | hsa-miR-107 | miR-107, hsa-miR-107, miR107 |
| MIR1423P | hsa-miR-142-3p | miR-142-3p, hsa-miR-142-3p, miR142-3p, miR-1423p |
| PINK1 | ENSP00000364204 | PARK6, PINK1, PTEN-induced putative kinase 1, PINK-1, PTEN induced putative kinase 1, hPINK1, PTEN-induced putative kinase protein 1, BRPK, PINK 1, PARK 6, PTEN-induced putative kinase-1, PTEN-induced putative kinase1, PINK1 p, PTEN-induced putative kinase -1 |
| MLLT3 | ENSP00000369695 | AF9, AF-9, MLLT3, myeloid/lymphoid or mixed-lineage leukemia; translocated to, 3, MLLT3, super elongation complex subunit |
| SDC1 | ENSP00000370542 | Synd1, syndecan-1, syndecans, syndecan, CD138, Syndecan 1, Sdc1, SDC-1, CD 138, sDC, syndecan1, hSDC1, Synd-1, hCD138 |
| TH | ENSP00000370571 | tyrosine hydroxylase, DYT14, tyrosine-hydroxylase, tyrosine 3-monooxygenase, EC 1.14.16.2, tyrosine 3-hydroxylase, DYT 1-4, DYT5b, tyrosine 3-mono-oxygenase, Tyrosine-3-monooxygenase |

| | | |
|---|---|---|
| IL6ST | ENSP00000370698 | gp130, IL6ST, interleukin-6 signal transducer, gp 130, interleukin 6 signal transducer, cd130, gp-130, IL-6ST, IL-6RB, Interleukin-6-signal transducer |
| IL33 | ENSP00000370842 | IL-33, interleukin-33, NF-HEV, IL-1F11, IL33, interleukin 33, hIL-33, IL 33, Interleukin33, Interleukin- 33, Interleukin -33, IL- 33 |
| SOD3 | ENSP00000371554 | EC 1.15.1.1, EC-SOD, SOD3, EC SOD, sod-3, ecSOD, Superoxide dismutase 3, superoxide dismutase-3, Superoxide Dismutase 3, Extracellular |
| GPX2 | ENSP00000374265 | glutathione peroxidase 2, Selenoprotein S, GSHPx-GI, Gpx2, GPX-GI, GI-GPx, gastrointestinal glutathione peroxidase, gastrointestinal-glutathione peroxidase, selenoprotein-S, glutathione peroxidase-2, hgpx2, GPXGI |
| CCL8 | ENSP00000378118 | MCP-2, MCP2, hC14, CCL8, monocyte chemotactic protein-2, SCYA8, monocyte chemoattractant protein-2, chemokine ligand 8, chemokine ligand-8, monocyte chemotactic protein 2, CCL-8, MCP 2, C-C motif chemokine ligand 8, chemokine (C-C motif) ligand 8 |
| RUNX3 | ENSP00000382800 | Runx3, Cbfa3, AML2, AML-2, Runt-related transcription factor 3, RUNX-3, runt-related transcription factor-3, Runx 3, runt related transcription factor 3 |
| CDC42 | ENSP00000383118 | Cdc42, Rho-family GTPases, Rho family GTPases, G25K, CDC42Hs, 3 VHL, cell division cycle 42, TKs, cell-division cycle 42, CDC42-Hs, Cdc 42, Cdc-42, 1 an 0, cell division control protein 42 homolog, 2 NGR |
| NRG1 | ENSP00000384620 | Neuregulin-1, NRG1, NRG-1, HRG-1, neuregulin1, HRG, GGF2, neuregulin 1, GGF, SMDF, hrgA, MSTP131, 1 HAE, HRG1, 1-HRE, hGL |
| ACAN | ENSP00000387356 | aggrecan, AGC1, CSPGCP, ACAN, aggrecan proteoglycan, aggrecan core protein, aggrecan-1, aggrecan core-protein |
| OPRM1 | ENSP00000394624 | mu-opioid receptor, MOR, mu opioid receptor, mu opiate receptor, OPRM1, Oprm, mu-type opioid receptor, mu-opioid-receptor, hMOR-1, Mop, mu-opiate receptor, opioid receptor mu 1, opioid receptor mu1, hOPRM1, opioid receptor mu-1, OPRM 1, opioid receptor,  mu 1, opioid-receptor mu 1 |
| ALDH3A1 | ENSP00000411821 | aldehyde dehydrogenases, ALDH3, ALDH3A1, M74542, aldehyde dehydrogenase 3 family member A1, aldehyde dehydrogenase-3 |
| ZNF260 | ENSP00000429803 | PEX1, Zfp260, Pex1p, PEX 1, PEX1-p |

| | | |
|---|---|---|
| ITGAM | ENSP00000441691 | CD11b, Mac-1, integrin alpha subunits, Mac1, ITGAM, integrin alpha-subunits, integrin alpha M, integrin-alpha-M, integrin alpha-M, integrin alphaM, hCD11b, complement component 3 receptor 3 subunit, integrin subunit alpha M |
| PTHLH | ENSP00000441765 | Pthlh, PTHrP, Plp, Parathyroid hormone-like hormone, parathyroid hormone-related protein, PThr, parathyroid hormone related protein, osteostatin, parathyroid-hormone-related protein, P-thr, PTH-R, PTH-rP, ParaThyroid Hormone Like Hormone, parathyroid hormonelike protein, parathyroid hormone-like protein, parathyroid-hormone like hormone, P-LP, Parathyroid hormone related-protein, PHrP, parathyroid-hormone-like hormone, Parathyroid hormone--related protein |
| PFKFB3 | ENSP00000443319 | PFKFB3, PFK-2, iPFK-2, PFKFB-3, 6-phosphofructo-2-kinase/fructose-2, 6-bisphosphatase-3, PFK2, 6-phosphofructo-2-kinase/fructose-2, 6-bisphosphatase 3, 6-phosphofructo-2-kinase/fructose-2,  6-bisphosphatase 3, iPFK2, 6-phosphofructo-2-kinase/fructose 2, 6-bisphosphatase-3, 6-Phosphofructo 2-kinase/fructose 2,  6-bisphosphatase 3 |
| USP10 | ENSP00000457411 | USP10, ubiquitin-specific peptidase 10, ubiquitin specific protease 10, ubiquitin-specific protease 10 |
| SIRT5 | ENSP00000476228 | SIRT5, sirtuins, sirtuin, sirtuin 5, Sirtuin5 |
| EWSAT1 | EWSAT1 | EWSAT1, LINC00277 |

| | | |
|---|---|---|
| CSF2 | ENSP00000296871 | GM-CSF, granulocyte-macrophage colony-stimulating factor, Csf2, GMCSF, granulocyte-macrophage colony stimulating factor, granulocyte macrophage-colony-stimulating factor, granulocyte macrophage colony-stimulating factor, granulocyte-macrophage-colony stimulating factor, granulocyte-macrophage-colony-stimulating factor, granulocyte macrophage colony stimulating factor, granulocyte macrophage-colony stimulating factor, granulocyte-macrophage- colony-stimulating factor, colony-stimulating factor-2, G-CSF, granulocyte macrophage colony-stimulating factor, sargramostim, colony-stimulating factor 2, colony stimulating-factor, colony stimulating factor 2, colony-stimulating-factor, colonystimulating factor, colony stimulating factor 2 (granulocyte-macrophage, granulocyte-macrophage colony-stimulating-factor, Csf-2, granulocyte macrophage colonystimulating factor, GM - CSF, colony- stimulating factor, Csf, granulocyte-macrophage--colony-stimulating factor, Colony stimulating factor-2 |
| DEFB1 | ENSP00000297439 | beta-defensin-1, HBD-1, beta-defensin 1, BD1, beta defensin-1, Hbd1, Defb1, BD-1, beta defensin 1, defensin beta-1, defensin beta 1, DEFB 1 |
| TK1 | ENSP00000301634 | TK2, TK1, thymidine kinase 1, TK-1, EC 2.7.1.21, Thymidine kinase-1, 2 or V, EC2.7.1.21, thymidine Kinase1, TK 1, TK 2 |
| SOST | ENSP00000301691 | SOST, sclerostin, cdd |
| ABCA3 | ENSP00000301732 | ABCA3, ABC-C, ABC3, ABCC, hABCA3, LBM180, ATP-binding-cassette subfamily A member 3, ATP binding cassette subfamily A member 3, A-B-C-C, ATP binding cassette, subfamily a, member 3, ATP-binding cassette subfamily A member 3, ATP-binding cassette subfamily A, ATP-binding cassette transporter 3, ATP binding cassette subfamily A, ATP-binding cassette sub-family A member 3 |
| DNAJB7 | ENSP00000307197 | HSC-3, HSC3, DNAJB7 |
| MLKL | ENSP00000308351 | mixed lineage kinase domain-like protein, MLKL, Mixed lineage kinase domain-like, mixed lineage kinase domain-like pseudokinase, mixed lineage kinase domain like, mixed-lineage kinase domain-like protein, mixed lineage kinase domain like protein, mixed-lineage kinase domain-like, mixed lineage kinase domain like pseudokinase, mixed-lineage kinase domain-like pseudokinase |

| | | |
|---|---|---|
| CCL19 | ENSP00000308815 | CCL19, ELC, SCYA19, EBI1-ligand chemokine, CC chemokine ligand 19, CK beta-11, CK beta 11, macrophage inflammatory protein-3beta, Epstein-Barr virus-induced molecule 1 ligand chemokine, macrophage inflammatory protein-3 beta, EBI1 ligand chemokine, CCL-19, C-C chemokine ligand 19, CC motif chemokine ligand 19, Chemokine (C-C motif) ligand 19, CC-chemokine ligand 19, C-C motif chemokine ligand 19, C-C motif chemokine 19, CCL 19 |
| NLRP6 | ENSP00000309767 | hPan3, NLRP6, PYPAF5, PAN-3, PAN3, NLR family, pyrin domain containing 6 |
| FOSL1 | ENSP00000310170 | fra-1, fra, Fosl1, fos-related antigen-1, F-R-A, fra1, Fos-related antigen 1, FOS-like antigen 1, Fos related antigen-1, Fos related antigen 1, Fos transcription factor family, Fosl-1, FOS-like antigen-1, FOS like antigen 1, FOS like 1, AP-1 Transcription Factor Subunit |
| EDNRA | ENSP00000315011 | Ednra, ETA, endothelin receptor type A, ETA-R, ET-A, ET A, ET-RA, ETAR, Endothelin-1 receptor, ET AR, ETRA, endothelin 1 receptor, endothelin-1-receptor, ET-Ar |
| PKM | ENSP00000320171 | PKM, p58, PKM2, EC 2.7.1.40, p 58, cytosolic thyroid hormone binding protein, PK3, PK-3, p5-8, tumor M2-PK, PK-M, PK-M2, pyruvate kinase, muscle, PK2, P58P, pyruvate kinase PKM, PKM-2, pyruvate kinase muscle isozyme, Pyruvate Kinase M1/2, 3-M E3 |
| P4HB | ENSP00000327801 | p55, protein disulfide isomerase, PDI, P4H-beta, PDIA1, P4HB, protein disulfide-isomerase, ERBA2L, protein-disulfide isomerase, cellular thyroid hormone-binding protein, EC 5.3.4.1, cellular thyroid hormone binding protein, proHB, pro-HB, p 55, prolyl 4-hydroxylase, beta polypeptide, HP55, G-IT, CLCRP1, prolyl 4-hydroxylase subunit beta, PD-I |
| SIRT7 | ENSP00000329466 | sirtuin, SIRT7, sirtuins, Sirtuin 7, Sirtuin7, Sirtuin-7 |
| ALDH1A3 | ENSP00000332256 | Aldehyde dehydrogenase 6, ALDH1A3, aldehyde dehydrogenases, ALDH6, Raldh3, Aldehyde dehydrogenase 1 family, member A3, retinaldehyde dehydrogenase 3, raldh-3, aldehyde dehydrogenase1family memberA3, aldehyde dehydrogenase 1 family member A3 |

| | | |
|---|---|---|
| PTGDR2 | ENSP00000332812 | GPR44, CRTH2, chemoattractant receptor-homologous molecule expressed on Th2 cells, DP2, DP-2, chemoattractant receptor-homologous molecule expressed on T helper type-2 cells, chemoattractant-receptor homologous molecule expressed on Th2 cells, CD294, hCRTh2, chemoattractant receptor homologous molecule expressed on T helper type 2 cells, Chemoattractant receptor-homologous molecule expressed on T helper type 2 cells, prostaglandin D2 receptor 2, HDP2, PTGDR2, Chemoattractant Receptorhomologous molecule expressed on Th2 cells |
| CDKN3 | ENSP00000335357 | cyclin-dependent kinase inhibitor, hKAP1, KAP1, CKI, Cdc14 phosphatases, KAP-1, CDKN3, Cdi1, cyclin dependent kinase inhibitor, CDKI, cyclindependent kinase inhibitor, cyclin-dependent kinase interactor 1, cyclin dependent-kinase inhibitor, CIP2, kinase-associated phosphatase, CDK-I, cyclin-dependent kinase inhibitor 3, kap, cyclin-dependent-kinase inhibitor, KAP 1, cyclin-dependent kinase inhibitor-3, cyclin dependent kinase inhibitor 3, cyclin dependent kinase-inhibitor, cyclin-dependent-kinase-Inhibitor |
| SIRT6 | ENSP00000337332 | sirtuins, SIRT6, sirtuin 6, Sirtuin, Sirtuin-6, SIRT-6, Sirtuin6, SIRT 6 |
| TRPC6 | ENSP00000340913 | TRPC6, hTRPC6, Trp6, transient receptor potential cation channel, subfamily C,  member 6, TRPC-6, TRPC6 p, transient receptor potential cation channel subfamily C member 6 |
| UGT2B15 | ENSP00000341045 | UGT2B15, HLUG4, UGT2B8, UDP-glucuronosyltransferase 2B15, UDP-glucuronosyltransferase 2 B15 |
| PTN | ENSP00000341170 | Pleiotrophin, PTN, harp, HB-GAM, Osf1, heparin-binding growth-associated molecule, HBNF, OSF-1, Heparin-binding growth associated molecule |
| TRPV2 | ENSP00000342222 | TRPV2, VRL-1, transient receptor potential cation channel subfamily V member 2, Transient receptor potential cation channel, subfamily V,  member 2 |
| CTSB | ENSP00000345672 | cathepsin B, cathepsin S, APPs, APP secretase, cathepsins, CTSB, cathepsin-B, cathepsinB, cathepsin-S, cathepsin B1, APP s, APP-secretase |

| | | |
|---|---|---|
| ARNT | ENSP00000351407 | ARNT, TANGO, aryl hydrocarbon receptor nuclear translocator, HIF-1beta, arylhydrocarbon receptor nuclear translocator, HIF-1 beta, HIF1beta, hypoxia-inducible factor 1 beta, hypoxia inducible factor 1beta, hypoxia-inducible factor 1beta, Hif1b, hypoxia-inducible factor-1 beta, HIF-1b, Aryl hydrocarbon receptor nuclear-translocator, Aryl hydrocarbon-receptor nuclear translocator, 5NJ8 |
| FUT4 | ENSP00000351602 | CD15, FUT4, ELAM-1 ligand fucosyl transferase, ELAM ligand fucosyltransferase, Fuc-TIV, fucosyltransferase IV, SSEA1, FucT-IV, fucosyltransferase 4, FucTIV, CD 15, FUT IV, SSEA-1, LeX, Fucosyltransferase-4, fucosyltransferase4, Fuc-T IV |
| CNTNAP2 | ENSP00000354778 | Caspr2, CNTNAP2, Contactin Associated Protein-Like 2, Contactin-associated protein-like 2, CNTNAP2-001, contactin-associated proteinlike 2 |
| S100A7 | ENSP00000357712 | S100A7, psoriasin, PSOR1, S100A7-c, hS100A7, S100 calcium-binding protein A7 |
| PPA1 | ENSP00000362329 | PP1, HPP1, PP-1, PPase, inorganic pyrophosphatase, PP 1, PPA1, HPP-1, HP-P1, Cytosolic inorganic pyrophosphatase, Pyrophosphatase-1, Pyrophosphatase 1 |
| NPY4R | ENSP00000363431 | PP1, HPP1, PP-1, hY4, Ppyr1, PP 1, HPP-1, HP-P1, neuropeptide Y receptor Y4, NPY4R |
| EIF6 | ENSP00000363574 | eIF6, p27(BBP, eIF3a, p27BBP, ITGB4BP, Cab, integrin beta 4 binding protein, Ca-B, eukaryotic translation initiation factor 6 |
| FKBP4 | ENSP00000001008 | FKBP52, p52, FKBP51, Hb I, FKBP59, 52-kDa FK506-binding protein, FKBP4, hFKBP52, 52-kDa FK506 binding protein, immunophilin FKBP52, PPIase, 51-kDa FK506-binding protein, hSP56, rotamase, immunophilin FKBP-52, FKBP-59, FKBP-52, P5-2, FKBP-51, FK506-binding protein 4, FKBP-4, FK506 binding protein 4, 51 kDa FK506-binding protein |
| SIRT4 | ENSP00000202967 | sirtuins, SIRT4, sirtuin, Sirtuin 4, sirtuin-4, Sirtuin4 |
| GCK | ENSP00000223366 | glucokinase, Gck, EC 2.7.1.2, GLK, hK4, hexokinase 4, 3 IDH, hexokinase D |
| NFKB1 | ENSP00000226574 | p50, p105, Nfkb1, Ebp1, KBF1, NFKBp50, P 5 0, nuclear factor of kappa light polypeptide gene enhancer in B-cells 1, NF-KB1, NF-kB p50, nuclear factor kappa-B subunit 1, NF-kB-p50, NFkB p50, nuclear factor of kappa light polypeptide gene enhancer in B cells 1, HP 50, nuclear factor kappa B subunit 1, NF-kBp50 |
| HRG | ENSP00000232003 | histidine-rich glycoprotein, HRGP, HRG |

| | | |
|---|---|---|
| NUDCD1 | ENSP00000239690 | CML66, OVA66, AF283301, NudCD1, NudC domain containing 1, NudC family |
| LRP1 | ENSP00000243077 | CD91, alpha 2-macroglobulin receptor, LRP1, Low-density lipoprotein receptor-related protein 1, LDL receptor-related protein-1, low density lipoprotein receptor-related protein-1, A2MR, apolipoprotein-E receptor, low density lipoprotein receptor-related protein 1, alpha-2-macroglobulin receptor, Lrp, apolipoprotein E receptor, LDL receptor-related protein 1, A2M-r, low-density-lipoprotein receptor-related protein 1, LRP-1, CD 91, low-density lipoprotein receptor related protein 1, low-density lipoprotein receptor-related protein-1, alpha2-macroglobulin receptor, low density lipoprotein receptor related protein-1, LDL receptor related protein-1, low-density lipoprotein receptor-related protein1, IGFBP-3R, Low density lipoprotein receptor related protein 1, low-density-lipoprotein receptor-related protein-1, BC072015, LRP1 - A, prolow-density lipoprotein receptor-related protein 1, LRP 1, aPR, low-density lipoprotein receptor related protein-1, alpha 2 macroglobulin receptor, alpha 2Macroglobulin receptor |
| KLF2 | ENSP00000248071 | LKLF, Kruppel-like factor 2, KLF2, lung Kruppel-like factor, Kruppel-like factors, Lung-Kruppel-like factor, Kruppel-like Factor2, hKLF2, KLF-2, Kruppel like factor 2, Krueppel-like factor 2 |
| PEX1 | ENSP00000248633 | PEX1, peroxins, Pex1p, peroxin 1, peroxisomal biogenesis factor 1, Peroxin1, PEX 1, peroxisome biogenesis factor 1, PEX1-p |
| LRPPRC | ENSP00000260665 | gp130, LRPPRC, gp 130, lrp130, leucine-rich pentatricopeptide repeat containing, 130-kDa leucine-rich protein, leucine-rich pentatricopeptide repeat-containing, lrp 130, gp-130, Leigh Syndrome,  French-Canadian Type, leucine-rich pentatricopeptide-repeat containing |
| CCNE1 | ENSP00000262643 | Cyclin E1, Cyclin Es, CCNE1, Cyclin ET, CCNE, CyclinE1, G1/S specific cyclin E1, cyclin-E1 |
| IL7 | ENSP00000263851 | IL-7, interleukin 7, interleukin-7, IL7, hIL-7, hIL7, IL- 7 |
| ZAP70 | ENSP00000264972 | ZAP-70, Zap70, 4a4b, Zeta-chain (TCR)-associated protein kinase 70 kDa, StD, ZAP 70, Srk, zeta-chain (TCR) associated protein kinase 70kDa, 4a-4c, St-D, s t d |

| | | |
|---|---|---|
| ASCL1 | ENSP00000266744 | Mash1, achaete-scute homolog-1, ASCL1, achaete-scute family bHLH transcription factor 1, hASH1, Mash-1, HASH-1, ash1, achaete-scute homolog 1, ASH-1, achaete-scute complex homolog-like 1, Achaete-scute complex-like 1, Hash 1, ASCL-1, achaete-scute complex homolog 1, Achaete-scute complex homolog-1 |
| NPC1 | ENSP00000269228 | NPC1, Niemann-Pick C1 protein, NP-C1, Niemann-Pick disease, type C1, Niemann Pick C1 protein, hNPC1, npc-1, NPC1 human, NP-C 1, Niemann Pick Disease, Type C1, NPC 1, NPC intracellular cholesterol transporter 1 |
| REN | ENSP00000272190 | renin, REN, EC 3.4.23.15, re- N |
| CRH | ENSP00000276571 | CRH, corticotropin-releasing hormone, CRF, Corticotropin-releasing factor, CRH-1, corticotropin releasing hormone, C Rh, CRH1, corticotropin releasing factor, corticotropin- releasing hormone, CRH 1, corticotropin releasing-factor, Corticotropin releasing-hormone, corticotropin-releasing-factor, corticotropin-releasing-hormone |
| TM7SF2 | ENSP00000279263 | Ang1, Ang-1, TM7SF2, C14 sterol reductase, Ang 1, transmembrane 7 superfamily member 2 |
| PDCD4 | ENSP00000280154 | PDCD4, programmed cell death 4, programmed cell death protein 4, Programmed cell death 4, PDCD-4, programmed cell death protein4, programmed cell death4, programmed cell death protein-4 |
| ADAMTS5 | ENSP00000284987 | ADAM-TS5, ADAMTS5, ADAMTS-5, ADAMTS11, aggrecanase-2, a-disintegrin and metalloproteinase with thrombospondin motifs-5, ADAMTS5-P, ADAMTS 5, ADAM metallopeptidase with thrombospondin type 1 motif 5, aggrecanase -2 |
| PART1 | PART1 | PART-1, PART1 |
| ERCC1 | ENSP00000013807 | ERCC1, RAD10, ERCC-1, UV20, excision repair cross complementation group 1, Excision repair cross-complementation group 1, excision repair cross-complementing rodent repair deficiency, complementation group 1, excision repair cross-complementation group-1, ERCC 1, Excision repair cross complementation group-1, Excision repair cross complementation group 1, 2 mut, Excision-Repair Cross-Complementation Group 1, AC138128, 2mut, Excision repair cross-complementation group1, excision repair cross- complementation group 1 |
| CLDN11 | ENSP00000064724 | Oligodendrocyte-specific protein, OSP, CLDN11, claudin 11, claudin-11, Claudin11, Cldn-11 |

| | | |
|---|---|---|
| TNFRSF1A | ENSP00000162749 | TNFR1, TNFR, TNFRI, TNFRSF1A, TNF-RI, TNF-R1, p60, p55, TNF-R, TBP-1, tumor necrosis factor receptor 1, CD120a, TBP1, MS-5, tumor necrosis factor receptor-1, TNFR-1, TNF-R55, TNFR-I, MS5, p55-R, tumor necrosis factor receptor type I, TNF receptor superfamily member 1A, TNFR60, TNFR 1, TNF-R-I, tumor-necrosis-factor-receptor 1, tumor necrosis factor-receptor 1, TNF R55, TNFRSF 1A, TNFR-SF1A, tumor necrosis factor receptor superfamily member 1A, p 55, TNFR I, TNF R I, TNF RI, tumor necrosis factor receptor superfamily,  member 1a, HP55, 1 ICH, 1 TNR, TNFR55, hTNF-R55, TNFR-55, TNF-R 1, TNF- R55 |
| BDKRB1 | ENSP00000216629 | Bdkrb1, BDKRB2, B1 bradykinin receptor, B1R, B1 R, bradykinin receptor B1, BKR1, BKB1R |
| CYP24A1 | ENSP00000216862 | CYP24, Cyp24a1, P450cc24, CYP-24, HCa-I, cytochrome P450, family 24,  subfamily A,  polypeptide 1, cytochrome p450 24A1, hCA I, cytochrome P450 family 24 subfamily A member 1, CP24 |
| CSNK2A1 | ENSP00000217244 | CKII, CSNK2A1, casein kinase II subunit alpha, CK-II, CK II, 3 at 3, 3 at 4, casein kinase 2,  alpha 1 polypeptide, 5 MMR, 3 h 30, Cka1, Casein kinase 2 a1, 5 HGV |
| ABCD1 | ENSP00000218104 | ALDP, adrenoleukodystrophy protein, ABCD1, ATP-binding cassette, subfamily D,  member 1, ATP-binding cassette,  sub-family D, member 1, amn, AM N, ABCD1 p, ATP binding cassette subfamily D member 1, ALD-P |
| CCL22 | ENSP00000219235 | ABCD1, CCL22, mDC, SCYA22, macrophage-derived chemokine, macrophage derived chemokine, macrophage-derived-chemokine, chemokine (C-C motif) ligand 22, C-C motif chemokine 22, C-C motif chemokine ligand 22, CCL-22, Ccl 22 |
| PLAT | ENSP00000220809 | tPA, t-PA, PLAT, tissue-type plasminogen activator, Tissue type plasminogen activator, tissue-type plasminogen-activator, tissue type-plasminogen activator, plasminogen activator,  tissue, plasminogen activator,  tissue type, reteplase, Alteplase |
| KLRK1 | ENSP00000240618 | NKG2D, KLR, NKG2-D, KLRK1, killer-cell lectin-like receptors, killer cell lectin-like receptors, NKG2D activating NK receptor, killer cell lectin like receptor K1, killer cell lectin-like receptor subfamily K, member 1 |
| IRF1 | ENSP00000245414 | IRF-1, interferon regulatory factor 1, IRF1, interferon-regulatory factor-1, interferon regulatory factor-1, mAR, interferon-regulatory factor 1, IRF 1, MA-R |

| | | |
|---|---|---|
| TRAF2 | ENSP00000247668 | TRAF2, TRAF-2, TNF receptor-associated factor 2, TR AF-2, TNF receptor-associated factors, TNF-receptor associated factors, TRAF 2, TNF receptor-associated factor-2, 1-CA4, TNF receptor-associated-factor 2, TNF-receptor-associated factor-2, TNF-receptor associated factor 2, TNF receptor associated factor 2, TRAF, TNF Receptor Associated Factors, TNF-receptor-associated factors |
| ALOX12 | ENSP00000251535 | 12-LO, arachidonate 12-lipoxygenase, EC 1.13.11.31, ALOX12, 12-LOX, 12LO, ALOX-12, ALOX 12 |
| KRT1 | ENSP00000252244 | hCK1, keratin 1, HK1, KRT1, HK 1, Krt-1, Ehk-1, Ehk, cytokeratin 1, HK-1, keratin-1, HKRT-1, K-Rta, cytokeratin-1, keratin1, K1p |
| NUMBL | ENSP00000252891 | Numblike, NUMBL, Nbl, CTG3a, NUMB-like, Numb homolog-like, N-bl |
| CYP27A1 | ENSP00000258415 | CP27, CYP27, vitamin D(3) 25-hydroxylase, sterol 27-hydroxylase, CYP27A1, CTx, CYP 27, M62401, CP 27, sterol 27 hydroxylase, sterol-27-hydroxylase, cytochrome P450, family 27, subfamily A, polypeptide 1 |
| FLT4 | ENSP00000261937 | VEGFR-3, FLT4, VEGFR3, vascular endothelial growth factor receptor 3, fms-like tyrosine kinase 4, fms-related tyrosine kinase-4, vascular endothelial growth factor receptor-3, FLT-4, fms-related tyrosine kinase 4, VEGF-R3, VEGF R3 |
| SLC1A1 | ENSP00000262352 | EAAC1, Slc1a1, Excitatory amino-acid carrier 1, EAAT3, excitatory amino acid carrier-1, hEAAT3, Solute carrier family 1, member 1, EAAC-1, neuronal and epithelial glutamate transporter, excitatory amino acid carrier 1, excitatory amino acid transporter-3, solute carrier family 1, member1, excitatory amino acid transporter 3 |

| | | |
|---|---|---|
| PIK3CA | ENSP00000263967 | p110alpha, PIK3CA, PI3Kalpha, p110 alpha, PI3-Kalpha, 5 ITD, p110-alpha, phosphoinositide-3-kinase, catalytic, a polypeptide, phosphoinositide-3-kinase, catalytic, alpha polypeptide, Phosphoinositide-3-kinase catalytic alpha polypeptide, phosphoinositide 3 kinase catalytic alpha polypeptide, PIK 3CA, phosphatidylinositol-4, 5-bisphosphate 3- kinase, catalytic subunit alpha, PI3K-p, PIK3CA p, phosphatidylinositol-4, 5-bisphosphate 3-kinase, catalytic subunit alpha, phosphatidylinositol-4, 5-bisphosphate 3-kinase catalytic subunit alpha, phosphatidylinositol-4, 5-bisphosphate 3-kinase, catalytic subunit alpha, phosphatidylinositol-4, 5-bisphosphate 3-kinase catalytic subunit alpha, PI3K alpha, phosphatidylinositol-4, 5-bisphosphate 3-kinase-catalytic subunit alpha, phosphatidylinositol 4, 5-bisphosphate 3-kinase catalytic subunit alpha, PI3K p, phosphatidylinositol -4, 5-bisphosphate 3-kinase catalytic subunit alpha, PI3K-alpha, phosphoinositide 3-kinase, catalytic, alpha polypeptide, M-CAP, PIK3 CA |
| IL11 | ENSP00000264563 | IL-11, IL11, interleukin-11, interleukin 11, IL 11, interleukin11, hIL-11 |
| VCAN | ENSP00000265077 | Versican, PG-M, Cspg2, large fibroblast proteoglycan, ERVR, VCAN, versican proteoglycan, glial hyaluronate-binding protein, WGN1, pgm, Chondroitin sulfate proteoglycan 2, chondroitin sulfate proteoglycan2, versican core protein |
| AMBP | ENSP00000265132 | HCP, lipocalins, protein HC, Ambp, bikunin, HI-30, alpha 1-microglobulin/bikunin precursor, inter-alpha-trypsin inhibitor light chain, complex-forming glycoprotein heterogeneous in charge, alpha1-microglobulin/bikunin precursor, A1M, trypstatin, HC P, EDC1 |
| GABARAPL1 | ENSP00000266458 | gec1, Apg8L, Atg8, gamma-aminobutyric acid receptor-associated protein-like 1, GABARAP-L1, GABARAPL1, GABA(A) receptor-associated protein like 1, ATG8b, GABARAPL-1, GABA type A receptor-associated protein like-1 |
| BCL2A1 | ENSP00000267953 | BCL2 family, BCL2A1, ACC1, Bfl-1, BCL-2 family, ACC2, GRs, Bcl-2-family, Bcl-2-related protein A1, ACC-1, BFL1, ACC-2, BCL2-family, BCL2-related protein A1, protein Bfl-1, Bcl-2A1, Bcl2-A1, HBPA1, Bcl 2-family, hBfl-1, BCL2 Related Protein A1 |

| | | |
|---|---|---|
| MAP1LC3B | ENSP00000268607 | MAP1LC3B, LC3B, microtubule-associated proteins 1A/1B light chain 3B, microtubule-associated protein 1 light chain 3beta, microtubule-associated protein 1 light chain-3B, microtubule-associated protein-1 light-chain-3B, microtubule-associated protein 1 light chain 3B, LC-3B, microtubule-associated protein 1 light chain 3 beta, LC3-B, microtubule associated protein 1 light chain 3 B, microtubule associated protein 1 light chain 3 beta, autophagy-related protein LC3B, microtubule-associated protein-1 light chain 3b, microtubule-associated protein-1 light chain-3B, microtubule-associated protein 1 light chain 3 beta, microtubule associated protein 1 light chain 3-B |
| WIF1 | ENSP00000286574 | Wnt-inhibitory factor-1, WIF-1, WIF1, Wnt inhibitory factor 1, Wnt inhibitory factor-1 |
| ADAMTS3 | ENSP00000286657 | ADAMTS4, ADAMTS-4, ADAMTS3, KIAA0366, ADAM-TS3, ADAM-TS4, ADAMTS-3, ADAMTS 3, ADAM metallopeptidase with thrombospondin type 1 motif 3 |
| CLDN12 | ENSP00000287916 | CLDN12, claudin-1-2, claudin-12 |
| SMAD6 | ENSP00000288840 | Smad6, MADH6, Smad family, Smad 6, Madh7, Smad-6, SMAD family member 6 |
| SYK | ENSP00000364907 | Syk, Spleen Tyrosine Kinase, p72syk, spleen-tyrosine kinase, spleentyrosine kinase, spleen associated tyrosine kinase, tyrosine-protein kinase SYK, spleen-associated tyrosine kinase |
| TNFRSF25 | ENSP00000367013 | wsl, TR3, WSL-1, DR3, TNFRSF25, Death receptor-3, hTR3, LARD, Apo3, Apo-3, death receptor 3, lymphocyte-associated receptor of death, DR-3, TNFRSF12, DR 3, Apo 3, D-R3, death-receptor 3, TR 3, hTR 3 |
| HAO1 | ENSP00000368066 | GOX, HAOX1, HAO1, hydroxyacid oxidase 1 |
| NPHS1 | ENSP00000368190 | nephrin, NPHS1, AF035835, NPHS 1, NPHS-1, cNF |
| SERPINB9 | ENSP00000370074 | PI9, PI-9, SERPINB9, Serpin B9 |
| TANK | ENSP00000376505 | TRAF2, I-TRAF, TRAF-2, TANK, Traf family member-associated NF-kappaB activator, TRAF interacting protein, TRAF-interacting protein, TR AF-2, TRAF 2, TRAF family member-associated NFkappaB activator, TRAF family member-associated NF-kappa-B activator |
| PTPA | ENSP00000377036 | PP2A, PTPA, phosphotyrosyl phosphatase activator, PP-2A, PPP2R4, protein phosphatase 2 phosphatase activator, PP 2A |
| KIF20A | ENSP00000378356 | RAB6KIFL, Rabkinesin6, MKlp2, Kif20A, rabkinesin-6, GG10_2, Kinesin family member 20A |

| | | |
|---|---|---|
| TYMP | ENSP00000379038 | thymidine phosphorylase, gliostatin, PD-ECGF, ECGF1, platelet-derived endothelial cell growth factor, TYMP, hPD-ECGF, endothelial cell growth factor 1, PDECGF, platelet-derived endothelial-cell growth factor, platelet derived endothelial cell growth factor, ECGF, endothelial cell growth factor-1, ECGF 1, endothelial-cell growth factor-1, ECGF-1, EC 2.4.2.4, Platelet-derived endothelial cell growth-factor, platelet derived-endothelial cell growth factor, thymidine-phosphorylase |
| DAPK1 | ENSP00000386135 | DAPK1, DAPK, death-associated protein kinase-1, death-associated protein kinase 1, DAPK 1, DAPK-1, DAP-K, death associated protein kinase-1, Death associated protein kinase 1, Death-associated protein kinase1, death-associated protein kinases |
| PTPN6 | ENSP00000391592 | SHP-1, HCP, Hcph, PTPN6, protein-tyrosine phosphatase SHP-1, SHP1, PTP1C, protein tyrosine phosphatase SHP-1, protein tyrosine phosphatase 1C, protein tyrosine phosphatase, nonreceptor type 6, hematopoietic cell protein-tyrosine phosphatase, SH-PTP1, protein-tyrosine phosphatase SHP1, protein tyrosine phosphatase, non-receptor type 6, SHP-1L, SHPTP1, HC P, PTP-1C, tyrosine-protein phosphatase non-receptor type 6, SHP -1 |
| TOP2B | ENSP00000396704 | DNA topoisomerase IIbeta, TOP2B, TOP2beta, topoisomerase (DNA) IIbeta 180 kDa, DNA topoisomerase II beta, TOP2 beta, 3QX3, DNA topoisomerase IIb |
| MAP2K4 | ENSP00000410402 | SEK1, mitogen-activated protein kinase kinase 4, MKK4, MAP2K4, JNKK1, JNKK, JNK-activating kinase 1, sek-1, mitogen activated protein kinase kinase 4, MEK4, SAPK/ERK kinase-1, SAPK/ERK kinase 1, SERK1, stress-activated protein kinase kinase 1, MEK-4, mitogen-activated protein kinase kinase-4, MAPKK4, mkk-4, SKK-1 |
| SLC35A2 | ENSP00000416002 | UGT, HUGT1, UGT2, Ugt1, SLC35A2, HUGT2, UDP-galactose transporter, UGT-1, UDP-galactose translocator, ugtL, UGT-2 |
| CXCL2 | ENSP00000427279 | CXCL2, MIP-2, GRO2, CXCL-2, chemokine ligand 2, MIP2, macrophage inflammatory protein 2 alpha, GRO-2, chemokine ligand-2, GR-Ob, chemokine (C-X-C motif) ligand 2, HMIP-2, C-X-C motif chemokine ligand 2, HMIP-2A, C-X-C motif chemokine ligand-2, CXC motif chemokine ligand 2 |
| CCNE2 | ENSP00000429089 | Cyclin E2, CCNE2, cyclinE2 |

| | | |
|---|---|---|
| GSDMD | ENSP00000433209 | DFNA5L, GSDMD, gasdermins, gasdermin-D, gasdermin D, GSDMDC1 |
| H2AX | ENSP00000434024 | H2AX, H2A.X, H2AFX, histone H2AX, histone H2A.X, H2A histone family member X, H2A histone family, member X |
| TLR5 | ENSP00000440643 | TLR5, Toll-like receptor 5, Sle1, TIL3, hSLE1, TLR-5, TLR 5, toll like receptors, Toll-like receptor-5, Tlr, toll- like receptors, Toll like receptor 5, Toll-Like-Receptors, toll-like receptor5 |
| HSD3B2 | ENSP00000445122 | HSD3B2, HSD3B, HSD3B2 p |
| VDR | ENSP00000447173 | vitamin D receptor, vitamin D-receptor, Vdr, 1, 25-dihydroxyvitamin D3 receptor, vitamin-D receptor, vitamin D3 receptor, vitamin-D-receptor, 1, 25-dihydroxyvitamin D-3 receptor, NR 1I1, NR1I1, vitamin D receptor, vitamin D (1, 25-dihydroxyvitamin D3) receptor, 2 Has |
| TRAF3 | ENSP00000454207 | TNF receptor-associated factors, TRAF3, LAP1, TNF receptor-associated factor 3, c-raf1, c-raf-1, CRAF1, CD40-binding protein, Lap-1, CAP-1, c-raf 1, CD40 binding protein, Cap1, LMP1-associated protein 1, TNF-receptor associated factors, TRAF-3, TNF-receptor-associated-factor 3, cRaf-1, TRAF, TNF Receptor Associated Factors, ZINC fingers, TNF-Receptor Associated Factor 3, TRAF 3, TNF receptor associated factor 3, TNF-receptor-associated factors, C AP1, Cap1p |
| HAMP | ENSP00000471894 | Hepcidin, HEPC, Hamp, hepcidin antimicrobial peptide, LEAP-1, pLTR, liver-expressed antimicrobial peptide 1, LEAP 1 |
| STIM1 | ENSP00000478059 | STIM1, stromal interaction molecule 1, STIM1 p, STRMK, Tam-1, D11S4896E, GOK, stromal interaction molecule1, Stromal-interaction molecule 1, STIM 1, STIM-1, stromal interaction molecule-1, TAM1 |
| VPS11 | ENSP00000481126 | VPS11, VPS 11, Vacuolar Protein Sorting 11, END1, END-1, pep5 |
| MIR1013P | hsa-miR-101-3p | miR-101, miR-101-2, MIR101, hsa-miR-101, miR-101-3p, miR- 101, hsa-miR-101-3p |
| MIR1256 | hsa-miR-1256 | miR-1256 |
| MIR200B3P | hsa-miR-200b-3p | miR-200b, hsa-miR-200b, MIR200B, miR-200b-3p, miR-200 b, hsa-miR-200b-3p, miR 200b, miR200b-3p, miR- 200b |
| MIR2233P | hsa-miR-223-3p | miR-223, miR-223-3p, miR- 223, miR223, hsa-mir-223, miR223-3p, hsa-miR-223-3p, miR- 223-3p, miR-223 3p |
| MIR29A3P | hsa-miR-29a-3p | miR-29a, miR-29a-3p, miR29a, hsa-miR-29a, hsa-miR-29a-3p |

| | | |
|---|---|---|
| MIR29B3P | hsa-miR-29b-3p | miR-29b, miR-29b-3p, miR29b, hsa-miR-29b, miR 29b, hsa-miR-29b-3p, hsa-miR29b-3p |
| MIR374B5P | hsa-miR-374b-5p | miR-374b, MiR-374b-5p, hsa-miR-374b-5p, miR374b |
| MIR4235P | hsa-miR-423-5p | MiR423-5p, hsa-miR-423-5P, miR-423-5p |
| MIR520B | hsa-miR-520b | miR-520b |
| BTG2 | ENSP00000290551 | BTG2, TIS21, HPC3, PC-3P, HPC-3, B-cell translocation gene 2, B cell translocation gene 2, B-cell translocation gene-2, Btg/Tob family, BTG-2 |
| CAPN2 | ENSP00000295006 | calpain 2, CAPN2, mCANP, Calpain-2, calpain2 |
| HPGDS | ENSP00000295256 | PGDS, H-PGDS, glutathione-S-transferase, GSTs, GST, EC 2.5.1.18, 5 AIS, hPGDS, glutathione S transferase, glutathione S -transferase, Glutathione s-transferase, hematopoietic prostaglandin D synthase, Glutathione- S-transferase, GST-S, glutathione S- transferase, glutathione-S transferase, GST s, g lutathione S-transferase, prostaglandin-H2 D-isomerase, GSTs1, Ptgds2 |
| PTX3 | ENSP00000295927 | Ptx3, Pentraxin 3, long pentraxin 3, TSG-14, Ptx-3, pentraxin-3, pentraxin3, long pentraxin-3, hPTX3, PTX 3, Pentraxin 3, long, long-pentraxin 3 |
| SYN1 | ENSP00000295987 | SynI, Synapsin I, synapsins, SYN1, synapsin 1, synapsin-I, Syn-1, synapsin-1, Syn I, Syn-1A, syn1a, Syn-I, hSYN1, Synapsin1, Syn 1 |
| ITGA2 | ENSP00000296585 | collagen receptor, GPIa, integrin subunit alpha 2, GP Ia, ITGA2, integrin alpha subunits, GPI-A, CD49B, platelet membrane glycoprotein Ia, integrin alpha-subunits, Integrin alpha 2, integrin subunit alpha2, HPA-5, integrin alpha2, integrin-alpha2, Integrin alpha-2, Integrin, alpha 2, collagen-receptor, HPA5, HPA- 5 |
| SCIN | ENSP00000297029 | scinderin, adseverin, SCIN |
| USP32 | ENSP00000300896 | USP32, USP10, NY-REN-60, NY-Ren60, Ubiquitin specific protease 32 |
| TACR1 | ENSP00000303522 | spr, NK1R, NK-1 receptor, NK-1R, NK1 receptor, TACR1, substance P receptor, NKIR, tachykinin receptor 1, substance P-receptor, SP-R, NK-1-R, NK-1 R, NK1-R, TAC1R, NK1-receptor, substance-P receptor, TAC-R1, NK-1-receptor |
| HTRA3 | ENSP00000303766 | HtrA3, high-temperature requirement factor A3, pregnancy-related serine protease, T Asp, T-Asp, serine protease HtrA3, TASP, High temperature requirement factor A3, PRSP |
| FAM53A | ENSP00000310057 | DNTNP, dorsal neural tube nuclear protein, FAM53A, Family with sequence similarity 53-member A |

| | | |
|---|---|---|
| LPA | ENSP00000321334 | lp(A, apo A, apolipoprotein(a, lipoprotein(a, apolipoprotein (a), LpA, Lp (a, lipoprotein (a, apolipoprotein (a, ApoA, apo-A, Lp A, LP-a |
| DNMT3B | ENSP00000328547 | DNMT3B, DNA methyltransferase 3B, ICF1, EC 2.1.1.37, hDNMT3b, DNA-methyltransferase-3B, DNA methyltransferase-3B, DNA (cytosine-5-)-methyltransferase 3 beta, DNMT-3B, I-CF, DNA-methyltransferase 3B, DNA methyltransferase 3 beta, DNMT 3B, ICF-1, DNA (cytosine-5)-methyltransferase 3B, 3 FLG |
| MC2R | ENSP00000333821 | ACTHR, MC2R, ACTH-R, Melanocortin 2 receptor, MC2-R, melanocortin-2 receptor, adrenocorticotropic hormone receptor, adrenocorticotropin receptor, melanocortin 2 receptor (adrenocorticotropic hormone, melanocortin- 2 receptor, melanocortin-2-receptor, melanocortin receptor 2, hMC2R, melanocortin receptor-2 |
| CCKBR | ENSP00000335544 | Cckbr, CCKB, cholecystokinin-2 receptor, CCK2R, CCK-BR, CCK-B, cholecystokinin B receptor, cholecystokinin B-receptor, cholecystokinin-B receptor, cholecystokinin 2-receptor, cholecystokinin 2 receptor, cholecystokinin2-receptor, cholecystokinin-B-receptor, CCK-2R, cholecystokinin2 receptor, cholecystokinin-2-receptor, cholecystokininB receptor, cholecystokininB-receptor |
| CHAT | ENSP00000337103 | choline acetyltransferase, ChAT, choline acetyl-transferase, EC 2.3.1.6, choline acetyl transferase, ChoAcTase, choline O-acetyltransferase, choline-acetyltransferase, cholineacetyl transferase |
| ADIPOR1 | ENSP00000341785 | AdipoR1, adiponectin receptors, adiponectin receptor 1, Adipo-R1, adiponectin receptor-1, Adipo R1, adiponectin receptor1, ADIPOR-1, adiponectin-receptors |
| NR4A2 | ENSP00000344479 | Nurr1, NR4A2, orphan nuclear receptor Nurr1, RNR1, TINUR, RNR-1, HZF-3, Nurr 1, Nurr-1, nuclear receptor subfamily 4, group A, member 2, nuclear receptor subfamily 4 group A member 2, NoT |
| FBLN5 | ENSP00000345008 | Fibulin-5, FBLN5, fibulins, fibulin 5, EVEC, FBLN-5 |
| E2F1 | ENSP00000345571 | E2F-1, E2F1, RBP3, E2F transcription factors, transcription factor E2F-1, transcription factor E2F1, RBAP-1, E2-F1, E2F transcription factor 1, E2F transcription factor-1, E2F1 p, E2F1-P, retinoblastoma-associated protein 1 |

| | | |
|---|---|---|
| C5AR1 | ENSP00000347197 | C5a, C5r1, C5AR, C5aR1, CD88, C5a-R, hC5a, complement component 5 receptor 1, Complement C5a Receptor 1, C5a anaphylatoxin chemotactic receptor 1, complement component 5a receptor 1 |
| ARG1 | ENSP00000349446 | Arginase, Arg-1, liver-type arginase, ARG1, EC 3.5.3.1, arginase-1, type I arginase, arginase 1, 3 The, type-I arginase, arginase1, Arg 1 |
| FZD6 | ENSP00000351605 | Fz6, frizzled 6, Hfz6, FZD6, Frizzled6, Frizzled homolog 6, frizzled class receptor 6 |
| CUX1 | ENSP00000353401 | CCAAT displacement protein, CDP/cut, p75, Cux1, p100, p110, CUTL1, cux, ret fusion, CCAAT-displacement protein, COY1, Cux/CDP, CDP1, HP75, CDP/Cux, casp, Cux-1, HP-75, C-Asp, CCAAT-Displacement-protein, Cut-like homeobox 1, RET fusion, RET-fusion, cutlike homeobox 1, Cut Like homeobox 1, Cut-like homeobox1, Cut-like homeobox-1 |
| VPS13A | ENSP00000353422 | chorein, ChAc, VPS13A, vacuolar protein sorting 13A, vacuolar protein sorting 13 homolog A, vacuolar protein sorting-associated protein 13A, chorea-acanthocytosis protein |
| EGLN1 | ENSP00000355601 | EGLN1, PHD2, Egl-9 family hypoxia inducible factor 1, hph2, Sm-20, c1orf12, PHD2 p, EGL nine homolog 1, SM20, PHD-2, HIF prolyl hydroxylase 2, HPH-2, prolyl hydroxylase domain containing protein 2, HIF prolyl hydroxylase-2, prolyl hydroxylase domain-containing protein-2, prolyl hydroxylase domain-containing protein 2, Hypoxia-inducible factor prolyl hydroxylase 2, EGLN 1, HIF-prolyl hydroxylase 2, HIF prolyl-hydroxylase-2 |
| FLVCR1 | ENSP00000355938 | FLVCR, FLVCR1, feline leukemia virus subgroup C cellular receptor 1, AXPC1, AxPC-1, FLVCR1 p |
| ADAMTS4 | ENSP00000356975 | ADAM-TS4, ADAMTS-2, ADAMTS-4, ADAMTS4, aggrecanase 1, ADAMTS2, aggrecanase-1, KIAA0688, ADAM-TS2, aggrecanase1, ADAMTS 2 |
| NES | ENSP00000357206 | nestin, Intermediate filaments type IV |
| BGLAP | ENSP00000357255 | osteocalcin, bone Gla protein, OCN, BGLAP, Bgp, gamma-carboxyglutamic acid-containing protein, Bone gamma-carboxyglutamate protein, Bone Gla-protein |
| S100A12 | ENSP00000357726 | MRP6, CAAF1, S100A12, calgranulin C, S100 A12, EN-RAGE, extracellular newly identified RAGE-binding protein, protein S100A12, HP6, ENRAGE, S100 calcium binding protein A12, protein S100-A12, S100 calcium-binding protein A12, calgranulin-C |

| | | |
|---|---|---|
| CTSS | ENSP00000357981 | cathepsins, cathepsin S, Ctss, 2 fra, 2-Fra, cathepsin-S |
| ATG5 | ENSP00000358072 | Apg5, Atg5, hAPG5, APG5L, autophagy-related 5, autophagy protein 5, hApg5p, Apg5p, apoptosis specific protein, Atg 5, Atg-5, autophagy related 5, AS-P |
| PITX3 | ENSP00000359019 | Ptx3, Pitx3, paired-like homeodomain transcription factor 3, Ptx-3, pituitary homeobox 3, paired-like homeodomain 3, Pitx-3, ASMd, paired like homeodomain 3 |
| GPC4 | ENSP00000359864 | GPC4, glypicans, glypican-4, K-glypican, glypican 4, GPC-4, Glypican4 |
| TET1 | ENSP00000362748 | TET1, leukemia-associated protein with a CXXC domain, ten-eleven translocation 1, CXXC6, Tet methyl cytosine dioxygenase 1, Tet methylcytosine dioxygenase 1, ten eleven translocation 1, methylcytosine dioxygenase TET1, Tet-1, TET 1, Ten-eleven-translocation 1, ten-eleven translocation-1 |
| MIR8080 | hsa-miR-8080 | miR-8080 |
| FOXP3 | ENSP00000365380 | Foxp3, scurfin, forkhead box P3, forkhead box protein P3, forkhead box-P3, Foxp-3, FOX-P3, FOX P3, hFoxp3, Forkhead boxP3, forkhead-box P3, Forkhead-Box-Protein P3, Forkhead-Box-P3, immune Dysregulation, Polyendocrinopathy, Enteropathy, X-linked |
| GATA1 | ENSP00000365858 | GATA-1, GATA1, NF-E1, Eryf1, Gf-1, hGATA1, Eryf 1, GF1, erythroid transcription factor, GATA binding protein 1, GATA binding protein-1, NFE1, globin transcription factor 1, Eryf-1, GATA-binding protein-1, HGF1, hEryf 1, HGF-1, GATA-binding protein 1, NFE-1, GATA-binding factor 1, GATA binding factor 1, G-F1, GATA 1 |
| PTER | ENSP00000367239 | p ter, rpr-1, PTER, Phosphotriesterase-related protein |
| TEK | ENSP00000369375 | Tie2, Tie-2, VMCM1, Tie 2, TEK receptor tyrosine kinase, Etk, angiopoietin-1 receptor, tyrosine-protein kinase receptor Tie2, Tie2-human, CD202b, tunica interna endothelial cell kinase, angiopoietin 1 receptor, TEK tyrosine kinase, endothelial, TIE2 p |
| TLR7 | ENSP00000370034 | hTLR7, TLR7, Toll-like receptor 7, TLR 7, TLR-7, Toll-like receptor-7, toll like receptors, Tlr, Toll-like receptor -7, toll- like receptors, toll like receptor 7, toll like receptor-7, Toll-like-receptor-7, Toll like receptor7, toll-like-receptor 7, Toll-Like-Receptors |

| | | |
|---|---|---|
| NFATC2 | ENSP00000379330 | NFAT1, NFATc2, NFATp, nuclear factors of activated T cells, nuclear factor of activated T cells 2, NF-ATp, NF-ATc2, NFAT C2, NFAT 1, NFAT-1, Nfat-C2, nuclear factor of activated T-cells, cytoplasmic, calcineurin-dependent 2, nuclear factor of activated T cells, cytoplasmic, calcineurin-dependent 2, Nuclear Factors of Activated T-cells, nuclear factor of activated T-cells, cytoplasmic 2, NF-AT1 |
| CCK | ENSP00000379472 | Cholecystokinin, CCK, C-CK, Preprocholecystokinin |
| JAM2 | ENSP00000383376 | Vascular endothelial junction-associated molecule, JAM2, JAM 2, JAM-B, VE-JAM, hJAM2, junctional adhesion molecule 2, junctional adhesion molecule B, junctional adhesion molecule-B, JAM-2 |
| ATCAY | ENSP00000390941 | CLAC, Caytaxin, BNIP-H, ATCAY, BNIP-2-homology |
| HK1 | ENSP00000402103 | hexokinase type I, HK1, HKI, Hexokinase-1, EC 2.7.1.1, hexokinase 1, hHK1, HK 1, HK-1, HK I, HKd, HK-I, HXK-1, hexokinase1, HXK1, HK1 p |
| WDR26 | ENSP00000408108 | WDR26, MIP-2, MIP2, hWDR26, HMIP-2 |
| SPATA2 | ENSP00000416799 | PD-1, PD1, hPD-1, SPATA2, PD 1, spermatogenesis associated 2, hPD1, Tamo, spermatogenesis-associated 2, PD- 1, spermatogenesis-associated protein 2 |
| GPBAR1 | ENSP00000430886 | TGR5, membrane-type receptor for bile acids, M-BAR, BG37, GPC-R, GPBAR1, G-protein-coupled bile acid receptor 1, G protein-coupled bile acid receptor 1, Gpbar-1, GP-BAR1, G-protein-coupled bile acid receptor, G protein-coupled bile acid receptor, G protein-coupled bile acid receptor-1, hTGR5, GPcR, G-protein coupled bile acid receptor 1 |
| HTR2A | ENSP00000437737 | 5-HT2A, HTR2A, HTR2, serotonin receptor 2A, Htr-2, 5Ht2a, 5-hydroxytryptamine receptor 2A, serotonin-receptor 2A, serotonin receptor-2A |
| KRT8 | ENSP00000449404 | Keratin 8, hK8, CK8, cytokeratin 8, keratin-8, KRT8, CK 8, cytokeratin-8, KRT 8, K8 p, CARD2, keratin8, CK-8, CARD 2 |
| NPC2 | ENSP00000451112 | hE1, NPC2, NP-C2, epididymal protein-1, HE-1, epididymal protein 1, Niemann-Pick Disease, type C2, hNPC2, Npc2p, Niemann-Pick disease type C 2 protein, NPC2 p, NPC-2 |

| | | |
|---|---|---|
| MC1R | ENSP00000451605 | Melanocortin-1 receptor, MC1R, melanocortin 1 receptor, melanocyte-stimulating hormone receptor, MSHR, MC1-R, melanocyte stimulating hormone receptor, melanocyte-stimulating--hormone receptor, melanocortin receptor 1, Melanocortin-1-receptor, MSH-R, Melanocortin receptor-1, Alpha-melanocyte-stimulating hormone receptor, MC-1R, melanocortin-receptor 1, SH-EP-2, melanocortin-1receptor, MC1R p, SHEP-2, hMC1R, MC1 R |
| MMP12 | ENSP00000458585 | MMP-12, MMP12, MME, macrophage metalloelastase, Macrophage elastase, matrix metalloproteinase 12, Matrix metalloproteinase-12, 4H84, hMMP-12, 2 MLS, matrixmetalloproteinase 12, matrix metallopeptidase 12, Matrix-metallo-proteinase-12, 2 mL s, 3F17, 3 RTS |
| RPL17-C18orf32 | ENSP00000463379 | PD-1, PD1, rpL17, L17P, PD 1, RPL23, PD- 1 |
| RBFOX3 | ENSP00000463653 | NeuN, Rbfox3, Neuronal Nuclei, Neu-N, RNA-binding protein,  fox-1 homolog 3, RNA-binding protein fox-1 homolog 3, RNA binding protein fox-1 homolog 3, FOX3p, RNA binding fox-1 homolog 3 |
| ANXA8 | ENSP00000478026 | annexin, annexins, ANX8, VAC-beta, Annexin A8, ANXA8 |
| GJA5 | ENSP00000484552 | Cx40, hCx40, connexin 40, connexin-40, connexin40, GJA5, gap junction protein alpha 5, GJA5-p, Cx 40 |
| DCN | ENSP00000052754 | decorin, DS-PG2, PG-II, PG-40, PGII, PG II, decorin proteoglycan, PG-S2, Dcn, PGS2, PGS-2, decorin-proteoglycan |
| MAP2K5 | ENSP00000178640 | MEK5, MAPK/ERK kinase 5, MAP2K5, mitogen-activated protein kinase kinase 5, mitogen activated protein kinase kinase 5, MKK5, MAPK/Erk kinase-5 |
| BRF2 | ENSP00000220659 | TFIIIB50, Brf2 |
| EPOR | ENSP00000222139 | erythropoietin receptor, EPOR, Epo-R, erythropoietin-receptor, EPO R |
| WNT16 | ENSP00000222462 | WNT-16, Wnt family, Wnt16, Wnt-family, WNT 16, hWNT16 |
| PON2 | ENSP00000222572 | PON2, paraoxonases, EC 3.1.1.2, Paraoxonase-2, paraoxonase 2, paraoxonase2, PON 2, PON-2 |
| EPX | ENSP00000225371 | Epo, eosinophil peroxidase, EPX, EC 1.11.1.7, eosinophil-peroxidase |
| MGP | ENSP00000228938 | MGP, matrix Gla protein, matrix-gla-protein, Mglap, Matrix Gla-protein, matrix-Gla protein |
| NR3C1 | ENSP00000231509 | glucocorticoid receptor, grl, NR3C1, 4 MDD, glucocorticoid-receptor, GCR, nuclear receptor subfamily 3,  group C,  member 1, GC-R, Nuclear Receptor Subfamily 3 Group C Member 1 |

| | | |
|---|---|---|
| TP53I3 | ENSP00000238721 | PIG3, TP53I3, PIG-3, tumor protein p53 inducible protein 3 |
| C3 | ENSP00000245907 | C3b, complement C3, C3a-anaphylatoxin, HC3, complement component C3b, complement component 3, c_3, C3A, HC 3, H-C3, C3a anaphylatoxin, complement component-3, complement-C3, 4 on T, 2 ICF, C3 p, C3-p, c-3p, AS-P |
| FFAR1 | ENSP00000246553 | GPR40, hGPR40, free fatty-acid receptor 1, FFAR1, G protein-coupled receptor 40, free fatty acid receptor 1, G-protein-coupled receptor 40, G-protein coupled receptor 40, FFA1R, free fatty acid receptors, free-fatty acid receptors, free fatty acid-receptor 1, free fatty acid receptor-1, G-protein coupled-receptor 40 |
| LIF | ENSP00000249075 | leukemia inhibitory factor, leukemia-inhibitory factor, differentiation inhibitory activity, differentiation-stimulating factor, Lif, differentiation-inducing factor, MLPLI, hepatocyte-stimulating factor-III, Hepatocyte-stimulating factor III, differentiation inducing factor, differentiation stimulating factor |
| EIF2S1 | ENSP00000256383 | eIF2alpha, eIF-2alpha, eIF-2 alpha, EIF2S1, eIF2A, eukaryotic translation initiation factor 2 subunit alpha, eukaryotic translation initiation factor 2 subunit 1, eIF2 alpha, eIF-2a, eIF 2 alpha, eIF2-alpha |
| DSG1 | ENSP00000257192 | Dsg1, desmoglein 1, desmosomal cadherins, Dsg, Desmoglein-1, Dsg 1, desmoglein1 |
| RIPK1 | ENSP00000259808 | RIP1, receptor-interacting protein 1, RIPK1, receptor-interacting serine/threonine-protein kinase 1, Rip-1, receptor interacting protein 1, rip, Receptor interacting protein kinase 1, receptor interacting protein-1, receptor-interacting protein kinase-1, receptor-interacting serine/threonine kinase 1, Receptor-interacting protein kinase 1, receptor-interacting serine-threonine kinase-1, Receptor-interacting protein-1, receptor (TNFRSF)-interacting serine-threonine kinase 1, Receptor-interacting Serine/Threonine Protein Kinase 1, receptor-interacting protein kinase1, receptor interacting protein kinase-1, receptor interacting serine/threonine kinase1, RIPK-1, receptor interacting serine/threonine kinase 1, Receptor-interacting serine threonine kinase 1 |

| | | |
|---|---|---|
| POU5F1 | ENSP00000259915 | Oct-4, Oct3, POU5F1, Oct4, OTF3, Otf-3, Oct-3, Octamer-binding transcription factor 3, Oct 3, POU domain, class 5, transcription factor 1, octamer binding transcription factor 3, Oct 4, POU class 5 homeobox 1, hOct4, Octamer-binding protein 4, octamer binding protein 4, hOCT3 |
| KHK | ENSP00000260599 | KHK, fructokinase, ketohexokinase, ketohexokinase (fructokinase, hepatic fructokinase |
| PSMD11 | ENSP00000261712 | PSMD11, p44.5, RPN-6, S9P, 26S proteasome non-ATPase regulatory subunit 11, proteasome 26S subunit, non ATPase 11 |
| EP300 | ENSP00000263253 | p300, EP300, Histone acetyltransferase p300, E1A-associated protein p300, E1A binding protein p300, histone acetyl transferase p300, KAT3B, histone-acetyltransferase p300, P300P, E1A-binding protein p300, RSTS2 |
| IGFBP1 | ENSP00000275525 | IGF-binding protein-1, IGFBP1, IGFBP-1, IBP-1, insulin-like growth factor-binding protein 1, IGF-BP25, IGF binding protein-1, IGF-binding protein 1, insulin-like growth factor binding protein 1, insulin-like growth factor binding protein-1, Insulin-like growth factor-binding protein-1, hIGFBP-1, IGF binding protein 1, insulinlike growth factor binding protein-1, insulin like growth factor binding protein 1, IGF BP-1, IGF-BP 1, hIGFBP1, insulinlike growth factor binding protein 1, binding protein 28, binding protein 25, PP12, IG-FBP-1, IGFBP 1 |
| ZFP36L2 | ENSP00000282388 | Zfp36l2, Brf2, ERF-2, Tis11d, Erf2p, zinc finger protein 36, C3H type-like 1, erf2 |
| FLT1 | ENSP00000282397 | VEGFR-1, Flt-1, Flt1, VEGFR1, fms-like tyrosine kinase 1, vascular endothelial growth factor receptor-1, Flt, fms-related tyrosine kinase 1, Fms-like tyrosine kinase-1, vascular endothelial growth factor receptor 1, VEGF-R1, Fms-related tyrosine kinase-1, Flt- 1, fms-like tyrosine kinase1, vascular-endothelial-growth factor receptor 1, VEGF R1, FLT 1, Fms related tyrosine kinase 1, VEGFR 1, Vascular permeability factor receptor |
| UCHL1 | ENSP00000284440 | Uchl1, Uch-L1, PGP9.5, Ubiquitin C-terminal hydrolase L1, ubiquitin C-terminal hydrolase-L1, PGP 9.5, Ubiquitin carboxyl-terminal esterase L1, UCHL-1, ubiquitin C-terminal hydrolases, PARK5, 2 ETL, ubiquitin carboxyl-terminal hydrolase isozyme L1, ubiquitin thiolesterase, PGP-9.5, UCH L1, Ubiquitin carboxyl- terminal esterase L1, Ubiquitin-C-terminal hydrolase L1, SPG79 |

| | | |
|---|---|---|
| SST | ENSP00000287641 | somatostatin, somatostatin-28, SST, somatostatin 28, somatostatin 14, preprosomatostatin, somatostatin-14, Smst, pre-prosomatostatin, prepro-somatostatin |
| MIR10A5P | hsa-miR-10a-5p | miR-10a, miR10a, hsa-miR-10a, miR-10a-5p, hsa-miR-10a-5p |
| MIR1275 | hsa-miR-1275 | miR-1275, hsa-miR-1275 |
| MIR135B5P | hsa-miR-135b-5p | mir135b, miR-135b, hsa-miR-135b, miR-135b-5p, MIR135B-5p, hsa-miR-135b-5p |
| MIR1885P | hsa-miR-188-5p | miR-188-5p, miR-188, hsa-miR-188-5p |
| MIR451A | hsa-mir-451a | miR-451, miR 451, hsa-miR-451, miR451, hsa-miR-451a, miR-451a, miR451a |
| LRP5 | ENSP00000294304 | LRP7, Hb M, LRP-5, LRP5, low-density lipoprotein receptor-related protein 5, low density lipoprotein receptor-related protein 5, HbM, LR3, LDL receptor-related protein 5, low-density lipoprotein-receptor-related protein 5, Hb-M, low-density-lipoprotein receptor-related protein 5, low-density lipoprotein receptor related protein 5, Low-density lipoprotein receptor-related protein-5, LDL-receptor-related protein 5, Lrp 7, low-density-lipoprotein-receptor-related protein 5, low density lipoprotein receptor related protein 5, LR 3, low density lipoprotein receptor-related protein-5 |
| RHO | ENSP00000296271 | Rho, rhodopsin, opsin 2, OPN2 |
| IL3 | ENSP00000296870 | interleukin-3, interleukin 3, IL-3, IL3, mast cell growth factor, hematopoietic growth factor, IL 3, MCGF, hIL-3, mast-cell growth-factor, multi-CSF, HGF, MGF, mast-cell growth factor, hematopoietic growth-factor, P-cell-stimulating factor |
| JAM3 | ENSP00000299106 | JAM2, JAM3, JAM-3, hJAM3, junctional adhesion molecule-C, JAM-C, junctional adhesion molecule 3, JAM 3, JAM 2, Junctional adhesion molecule C, JAM-2 |
| CLEC4E | ENSP00000299663 | Mincle, macrophage inducible C-type lectin, Clecsf9, Clec4e, macrophage-inducible C-type lectin, C-type lectin superfamily member 9 |
| HSP90B1 | ENSP00000299767 | gp96, GRP94, HtrA1, 94-kDa glucose-regulated protein, Grp 94, tumor rejection antigen 1, TRA-1, HSP90B1, TRA1, 94-kDa glucose regulated protein, Tra1p, 94 kDa glucose-regulated protein, Tra 1, heat shock protein 90 kDa beta (Grp94), member 1, HTRA-1, endoplasmin, grp-94 |
| PSCA | ENSP00000301258 | PSCA, prostate stem cell antigen, prostate stem-cell antigen, PSC-A |

| | | |
|---|---|---|
| KLK1 | ENSP00000301420 | kallikreins, tissue kallikrein, HK1, kallikrein 1, HK 1, KLK1, KLK6, hHK1, HK-1, hKLK1, kallikrein-1, KLK 1, kallikrein1, HK1 p |
| RPS9 | ENSP00000302896 | Rps9, ribosomal protein S9, S9P |
| GRK2 | ENSP00000312262 | GRK2, G protein-coupled receptor kinase 2, beta ARK1, GRK-2, G protein-coupled receptor kinase-2, G-protein-coupled receptor kinase 2, beta-adrenergic receptor kinase 1, betaARK1, G-protein-coupled receptor kinase-2, beta-ARK1, GRK 2, beta-adrenergic receptor kinase-1, G-protein coupled receptor kinase 2, G-protein coupled receptor-kinase 2, BARK1, Adrbk1, G protein coupled receptor kinase 2, G protein coupled receptor kinase-2, BARK-1, G-protein-coupled-receptor-kinase-2 |
| PRKDC | ENSP00000313420 | DNA-PKcs, XRCC7, p350, p460, PRKDC, DNA-dependent protein kinase catalytic subunit, HYRC1, HYRC, DNAPKcs, DNA PKcs, protein kinase, DNA-activated, catalytic polypeptide, DNA-PK-cs, DNA-PKcs, DNA dependent protein kinase catalytic subunit |
| CCNG2 | ENSP00000315743 | cyclin G2, CCNG2, cyclin-G2 |
| PCK1 | ENSP00000319814 | PCK1, phosphoenolpyruvate carboxykinase 1, Pck-1, phosphoenolpyruvate carboxykinase-1, PEPCK-C, EC 4.1.1.32, PEPCK1 |
| EML4 | ENSP00000320663 | C2orf2, EML4, ropp120, Ropp 120, restrictedly overexpressed proliferation-associated protein, echinoderm microtubule-associated protein-like 4, echinoderm microtubule-associated protein like 4, echinoderm microtubule-associated protein-like4, echinoderm microtubule-associated protein like-4, echinoderm microtubule associated protein like 4, echinoderm microtubule associated protein-like 4, echinoderm microtubule associated protein like-4, echinoderm microtubule-associated proteinlike 4, hEML4, Echinoderm microtubule associated proteinlike 4, echinoderm microtubule associated-protein like 4, echinodermmicro tubule associated protein-like 4 |
| IL17RA | ENSP00000320936 | interleukin 17 receptor, IL-17RA, IL-17R, hIL-17R, IL17RA, interleukin-17 receptor A, interleukin-17 receptor, CDw217, IL-17 RA, IL17R, IL17-RA, Interleukin 17 receptor A, 4-hsa |
| CDC25C | ENSP00000321656 | cdc25, Cdc25C, cdc-25, Cdc25-C, cell division cycle 25C, dual-specificity phosphatase Cdc25C, cdc-25c, M-phase inducer phosphatase 3 |

| | | |
|---|---|---|
| SOX2 | ENSP00000323588 | SOX2, transcription factor SOX2, Sox-2, SRY-Box 2, SRY box 2, SRY (sex-determining region Y)-box 2, Sox 2, hSox2, SRY (sex determining region Y)-box 2, SRY-box2, transcription factor SOX-2 |
| GSK3B | ENSP00000324806 | Glycogen synthase kinase-3beta, glycogen synthase kinase 3-beta, glycogen synthase kinase 3 beta, Glycogen synthase kinase-3 beta, glycogen synthase kinase 3beta, GSK3B, glycogen synthase kinase3-beta, GSK-3B, glycogen synthase kinase3beta, glycogen synthase kinase 3B, 3p up, 4 ACC, 4 PTC, glyco-gen synthase kinase 3 beta, glycogen synthase kinase-3b, 1J1B, 2 X 39 |
| STK11 | ENSP00000324856 | LKB1, STK11, liver kinase B1, serine/threonine kinase 11, hLKB1, LKB-1, stk-11, liver kinase B 1, serine/threo-nine kinase 11, STK 11, liver kinase B-1, STK11 p |
| KCNA4 | ENSP00000328511 | HK1, HuKII, HK 1, hHK1, Kv1.4, Kcna4, Kv 1.4, PCN2, hPCN2, HK-1, HK1 p, potassium voltage-gated channel subfamily A member 4 |
| TAC4 | ENSP00000334042 | PPT-C, HK1, TAC4, hHK1, Hemokinin, HK-1, preprotachykinin C, Tachykinin 4, HK 1, hHK-1, HK1 p |
| PDCD1 | ENSP00000335062 | hSLE1, PD-1, PDCD1, programmed cell death-1, programmed cell death 1, PD1, hPD-1, Programmed cell death protein 1, SLEB2, CD279, PD 1, hPDL, PDCD-1, programmed cell-death 1, programmed cell death protein-1, programmed-cell death-1, 5 GGs, programmed cell-death protein 1, hPD1, programmed cell death-protein 1, programmed-cell-death-protein-1, PD- 1, Programmed Cell Death -1, programmed cell-death protein-1 |
| ASCC1 | ENSP00000339404 | p50, ASCC1, SMABF2, P 5 0, CGI-18, HP 50 |
| ERBB4 | ENSP00000342235 | ErbB-4, erbB4, HER4, human epidermal growth factor receptor 4, HER-4, Her 4, human epidermal growth factor receptor-4, receptor tyrosine-protein kinase erbB-4, v-erb-b2 avian erythroblastic leukemia viral oncogene homolog 4, erb-B4, erb-b2 receptor tyrosine kinase 4, ErbB2 Receptor Tyrosine Kinase 4, receptor tyrosine-protein kinase ErbB4, ErbB 4 |
| HBM | ENSP00000349270 | Hb-M, HbM, Hb M, mu-globin, Hb K, hemoglobin subunits |
| LONP1 | ENSP00000353826 | Pim-1, PIM1, LONP1, LON, h-pim-1, PI M1, LonHS, mitochondrial ATP-dependent protease Lon, Pim 1, PIM- 1, Lon protease-like protein, LONP, LON peptidase 1,  mitochondrial |

| | | |
|---|---|---|
| TGM2 | ENSP00000355330 | tissue transglutaminase, EC 2.3.2.13, TG2, G alpha h, TG(C, Transglutaminase 2, TGM2, transglutaminase C, tissue trans-glutaminase, Tg-2, transglutaminase-2, tissue-transglutaminase, transglutaminase2, TGM-2, hTG2, TG-C, Tgc, tissue trans glutaminase, tg C |
| ULBP2 | ENSP00000356320 | RAET1H, ULBP2, ULBP-2, UL16 binding protein 2, UL16-binding protein 2 |
| TPM3 | ENSP00000357516 | trk, TPM3, TM5, tropomyosins, Nem1p, tropomyosin-3, TM30nm, gamma-tropomyosin, tropomyosin 3, TPM-3, hTM5, Tropomyosin3 |
| HOOK1 | ENSP00000360252 | HK1, Hook1, hHK1, HK 1, HK-1, hook 1, HK1 p, hook microtubule-tethering protein 1, Hook microtubule tethering protein 1 |
| DHCR24 | ENSP00000360316 | Seladin-1, DHCR24, 24-dehydrocholesterol reductase, DIMINUTO/DWARF1 homolog, 3beta-hydroxysterol Delta24-reductase, D-C-E, 3betahydroxysterol-delta24-reductase, 3-beta-hydroxysterol Delta-24-reductase, Dce, seladin 1, DHCR 24, 3beta-hydroxysterol-Delta24 reductase, 24-dehydrocholesterol reduc-tase, 3-betahydroxysterol delta-24-reductase, Seladin1, D-CE |
| MRPL41 | ENSP00000360498 | PIG3, MRPL41, PIG-3 |
| AGTR2 | ENSP00000360973 | AGTR2, angiotensin II type 2 receptor, AT2, AT-2, angiotensin II type-2 receptor, angiotensin II receptor type 2, angiotensin II type 2-receptor, Type 2 angiotensin II receptor, angiotensin II type2 receptor, angiotensin receptor 2, angiotensin-II-type-2-receptor, angiotensin-II type 2 receptor, angiotensin-II type-2 receptor |
| MKNK1 | ENSP00000361014 | MNK1, Mnk-1, MAP kinase interacting serine/threonine kinase 1, MKNK1, MAP kinase-interacting serine/threonine-protein kinase 1 |
| CDK9 | ENSP00000362361 | CDK9, cyclin-dependent kinase 9, Ctk1, C-2k, PITALRE, 4- or 5, Cyclin-dependent kinase-9, cyclin dependent kinase 9, Cyclin dependent kinase-9, cyclin-dependent-kinase 9, 4 or -5, CDK-9, CDK 9 |
| PIM1 | ENSP00000362608 | pim-1, PIM1, PiM, Pim-1 oncogene, 4 Alu, 5 toe, Pi M, PI M1, h-pim-1, 4 MBL, Pim 1, PIM1 oncogene, PIM- 1, 1XWS, 2BIK, Pim-1 proto-oncogene,  serine/threonine kinase, 4 as 0 |
| CXCR3 | ENSP00000362795 | CXCR3, GPR9, CXCR-3, chemokine (C-X-C motif) receptor 3, chemokine receptor-3, chemokine receptor 3, CD182, C-X-C motif chemokine receptor 3, C-X-C motif chemokine receptors, CXC motif chemokine receptor 3, CD183 |

| | | |
|---|---|---|
| MIR12965P | hsa-miR-1296-5p | miR-1296, miR 1296-5p, miR-1296-5p |
| MIR1413P | hsa-miR-141-3p | miR-141, mir141, Hsa-miR-141, hsa-miR-141-3p, hsa-miR141-3p, miR-141-3p, miR- 141, miR141-3p |
| MIR1469 | hsa-miR-1469 | miR-1469, hsa-miR-1469, MIR1469 |
| MIR2045P | hsa-miR-204-5p | miR-204, hsa-miR-204, miR-204-5p, miR204, hsa-miR-204-5p |
| MIR375 | hsa-miR-375 | miR-375, miR375, hsa-miR-375 |
| CCNY | ENSP00000363836 | cyclin X, HCFP1, CCNY, CFP1, cyclin Y, cyclinX |
| AIF1 | ENSP00000365227 | IRT1, Allograft inflammatory factor-1, ionized calcium binding adapter molecule 1, hG1, AIF-1, Iba1, IRT-1, interferon-gamma-responsive transcript, allograft inflammatory factor 1, AIF1, Iba-1, ionized calcium-binding adapter molecule-1, Hg-1, ionized calcium-binding adapter molecule 1, G1-P, Iba 1, ionized calcium binding adapter molecule-1, ionized calciumbinding adapter molecule 1, Allograft-inflammatory factor-1, I RT1 |
| SHB | ENSP00000366936 | Shb, SHB adaptor protein, s-HB |
| HSPA14 | ENSP00000367623 | HSP60, Hsp70L1, Hsp70-like protein 1, heat shock protein HSP60, HSP 60, HSP-60, heat shock 70 kDa proteins, HSPA14, heat-shock protein HSP-60 |
| PSIP1 | ENSP00000370109 | lens epithelium-derived growth factor, p52, LEDGF, p75, HP75, PSIP1, lens epithelium derived growth factor, P5-2, DFS70, HP-75, heparin-binding growth factor family, PC4 and SFRS1 Interacting Protein 1 |
| SLC7A1 | ENSP00000370128 | Cat-1, hCaT1, CaT1, ERR, hCAT-1, SLC7A1, ATRC1, Cat 1, c at 1, solute carrier family 7 member 1, ecotropic retroviral receptor |
| AK6 | ENSP00000370201 | TAF9, hTAFII32, hTAFII31, Cip, TAFII31, TAF2G, hCINAP, AK6, CINAP |
| AKR1C1 | ENSP00000370254 | aldo-keto reductases, aldoketoreductases, DDH1, HC-9, 20 alpha-hydroxysteroid dehydrogenase, h37, 20 alpha-HSD, HC9, 20alphaHSD, 20alpha-HSD, AKR1C1, 20alpha-hydroxysteroid dehydrogenase, EC 1.1.1.149, aldoketo reductases, DD-1, C9P, DDH-1, Dd1 |
| SDCBP2 | ENSP00000371233 | SITAC, ST2, hst-2, syntenin-2, HST2, similar to TACIP18, ST-2 |
| NADK2 | ENSP00000371362 | C5orf33, NADK2, DECRD, mitochondrial NAD kinase, MNADK |
| AQP4 | ENSP00000372654 | AQP4, aquaporin-4, aquaporin 4, MIWC, hMIWC2, mercurial-insensitive water channel, AQP-4, mercurial insensitive water channel, aquaporin4, hAQP4, AQP 4 |

| | | |
|---|---|---|
| HSPD1 | ENSP00000373620 | HSP60, GroEL, heat shock protein 60, HSPD1, Heat-shock protein 60, HSP-65, CPN60, Chaperonin 60, hsp65, HSP 60, HSP 65, heat shock 60 kDa protein 1, HSP-60, heat shock protein-60, heatshock protein 60, heat shock 60-kDa protein 1, hHSP60, heat shock 60 kDa protein 1 (chaperonin, heat shock 60kDa protein 1, chaperonin-60, heat shock protein family D member 1 |
| SUMO1 | ENSP00000376077 | sentrin, SUMO-1, Smt3, SUMO1, SENP2, small ubiquitin-related modifier-1, small ubiquitin-related modifier 1, PIC1, UBL1, DAP-1, ubiquitin-like 1, DAP1, Smt3p, HSMT3, small ubiquitin-like modifier 1, ubiquitin-homology domain protein PIC1, PIC-1, small ubiquitin-like modifier-1, hSUMO-1, OFC10, ubiquitin like 1, OFC -10, 2bF8, GMP-1 |
| BCL2L15 | ENSP00000376992 | BCL-2 family, BCL2 family, Bcl-2-family, BCL2-family, Bfk, C1orf178, BCL2L15, Bcl 2-family, AL137856 |
| BTC | ENSP00000379092 | betacellulin, Btc, beta-cellulin |
| AFP | ENSP00000379138 | alpha-fetoprotein, AFP, alpha feto-protein, Alphafetoprotein, alpha fetoprotein, alpha 1-fetoprotein, a-fetoprotein, alpha-feto protein, alpha1 fetoprotein, alpha1-fetoprotein, alpha-feto-protein, alpha-1-fetoprotein, alphafeto protein, FetA, Fet A, alpha feto protein, Fet-A, alpha - fetoprotein |
| RAPGEF4 | ENSP00000380271 | cAMP-GEFII, Epac, Epac2, RAPGEF4, exchange protein directly activated by cAMP 2, EPA-c, exchange protein directly activated by cAMP-2 |
| ITGB2 | ENSP00000380948 | CD18, LFA-1, integrin beta2, Mac-1, LF-A1, Mac1, integrin beta subunits, ITGB2, LFA1, integrin beta 2, integrin beta-2, integrinbeta2, CD 18, hCD18, 2J F1, integrin,  beta 2 (complement component 3 receptor 3 and 4 subunit, L A D, Integrin subunit beta 2, CD-18 |
| BCL2 | ENSP00000381185 | BCL2 family, bcl-2 family, Bcl-2-family, apoptosis regulator Bcl-2, BCL2-family, B-cell CLL/lymphoma 2, hbcl-2, Bcl-2 human, B cell CLL/lymphoma-2, B cell CLL/lymphoma 2, B-cell CLL/lymphoma-2, HBcL2, Bcl 2-family, BCL2,  apoptosis regulator, apoptosis regulator Bcl2 |
| GRB10 | ENSP00000381793 | GRB10, growth factor receptor-bound protein 10, Meg1, Irbp, Grb-IR, hGrb10, Grb10 adapter protein, Growth Factor Receptor Bound Protein 10 |
| MDK | ENSP00000385451 | midkine, MDK, MK1, MK-1, NEGF2, Amphiregulin-associated protein, ARAP |
| DGKB | ENSP00000385780 | diacylglycerol kinase beta, DGK, DGKbeta, DGKB, 90-kDa diacylglycerol kinase |

| | | |
|---|---|---|
| HSBP1 | ENSP00000392896 | HSBP1, heat shock factor binding protein 1, heat shock factor binding protein-1 |
| MAPK9 | ENSP00000394560 | Jnk2, JNK-2, Jun-kinase, JUN kinase, c-Jun N-terminal kinase 2, Mapk9, 3 NPC, JNK2alpha, MAPK-9, c-Jun N-terminal kinase-2, c-JUN-N-terminal kinase 2, mitogen-activated protein kinase 9, JNK 2 |
| MECP2 | ENSP00000395535 | MeCP2, methyl CpG binding protein 2, methyl-CpG-binding protein 2, MRX79, methyl CpG-binding protein 2, methyl-CpG binding protein 2, methyl-CpG-binding protein2, methyl-CpG binding protein-2, methyl-CpG binding-protein 2, MRX16, methyl-CpG-binding protein-2, methyl CpG-binding domain-containing, methyl-CpG binding protein2, hMeCP2, methylCpG-binding protein 2, Methyl CpG-Binding Protein-2, Methyl CpG binding protein-2, MeCp-2, MECP2 p, methyl-CpG-binding-protein 2, MethylCpG binding protein-2 |
| SMARCA4 | ENSP00000395654 | SNF2, SWI2, BRG1, SMARCA4, BRG-1, hSNF2, BAF190, protein BRG1, SWI/SNF-related,  matrix-associated,  actin-dependent regulator of chromatin,  subfamily a,  member 4, SWI/SNF related, matrix associated,  actin dependent regulator of chromatin, subfamily a,  member 4, RTPS2, transcription activator BRG1, BRM/SWI2-related gene 1 |
| RAPGEF3 | ENSP00000395708 | Epac, Epac1, cAMP-GEFI, exchange protein directly activated by cAMP 1, Epac-1, RapGEF-3, RAPGEF3, exchange factor directly activated by cAMP 1, exchange protein directly activated by cAMP1, exchange protein directly activated by cAMP-1, EPA-c |
| DIABLO | ENSP00000398495 | DIABLO, Smac, direct IAP binding protein with low pI, direct IAP-binding protein with low pI, second mitochondria-derived activator of caspase, second mitochondria derived activator of caspase, diablo IAP-binding mitochondrial protein |
| APTX | ENSP00000400806 | AOA1, Aprataxin, APTX, AOA-1 |
| PRAM1 | ENSP00000408342 | PML-RAR, PMLRAR, PRAM-1, PML- RAR |
| PTPRC | ENSP00000411355 | gp180, PTPRC, CD45, leukocyte common antigen, Ly-5, CD45R, B220, Ly5, LCA, leukocyte-common antigen, protein-tyrosine phosphatase,  receptor-type C, CD 45, hCD45, protein tyrosine phosphatase,  receptor type,  C, Protein tyrosine phosphatase, receptor type C, GP-18-0, gp-180 |
| EIF4E | ENSP00000425561 | eIF4E, eIF-4E, eIF4E-1, EIF4EL1, eukaryotic translation initiation factor 4E, c BP, Cbp, 4-aza, eIF4-E, eIF4E1 |

| | | |
|---|---|---|
| MLIP | ENSP00000426290 | Muscle-enriched A-type Lamin-interacting Protein, cip, MLIP, C6orf142, cardiac ISL1-interacting protein |
| PBK | ENSP00000428489 | PDZ-binding kinase, lymphokine-activated killer T-cell-originated protein kinase, TOPK, PBK, T-LAK cell-originated protein kinase, MAPKK-like protein kinase, PDZ binding kinase, T-LAK cell-originated protein kinase, T-Lak cell originated protein kinase, T-LAK-cell-originated protein kinase |
| LYN | ENSP00000428924 | Lyn, Src family tyrosine kinases, p56 LYN, Src-family tyrosine kinases, Lck/Yes-related novel protein tyrosine kinase |
| TRAF6 | ENSP00000433623 | TRAF6, TRAF-6, TNF receptor-associated factor 6, TNF receptor-associated factors, TNF-receptor associated factors, TNF-receptor-associated factor 6, TNF receptor-associated factor-6, TRAF, TNF receptor associated factor 6, TNF Receptor Associated Factors, TNF receptor associated factor-6, TRAF 6, TNF-receptor associated factor 6, TNF-receptor-associated factors |
| ARMS2 | ENSP00000436682 | ARMS2, age-related maculopathy susceptibility 2, Age-related maculopathy susceptibility protein 2, ARMS2 p, age-related maculopathy susceptibility2 |
| LATS1 | ENSP00000437550 | LATS1, hLATS1, Large tumor suppressor homolog 1, large tumor suppressor kinase 1, LATS 1 |
| NR4A1 | ENSP00000440864 | Nur77, hMR, HTR3, NGFI-B, TR3, Nur 77, NAK1, Nr4a1, nuclear receptor subfamily 4,  group A,  member 1, Nur-77, NAK-1, orphan nuclear receptor TR3, NGFIB, NGF-IB, HN10, testicular receptor 3, nuclear receptor subfamily 4 group A member 1, TR 3, hTR 3 |
| IL22 | ENSP00000442424 | IL-TIF, IL-21, IL-22, TIFA, IL21, interleukin-22, IL-10-related T cell-derived inducible factor, Interleukin 22, IL-2 2, IL22, IL- 21, IL 22, Interleukin -22, IL 21, IL2 1 |
| CEP290 | ENSP00000448012 | 3H11Ag, CEP290, NPHP6, nephrocystin-6, rd16, Poc3, centrosomal protein 290 kDa, centrosomal protein 290, centrosomal protein of 290 kDa, CEP-290 |
| SRD5A2 | ENSP00000477587 | SRD5A2, steroid 5 alpha-reductase 2, steroid 5alpha-reductase 2, 5 alpha-SR2, type II 5 alpha-reductase, steroid 5-alpha-reductase 2, Srd5A-2, SRD5A2 p, type II 5alpha-reductase, hSRD5A2, steroid-5-alpha-reductase,  alpha polypeptide 2, steroid 5-alpha reductase-2, 3-oxo-5-alpha-steroid 4-dehydrogenase 2 |
| PHB | ENSP00000479488 | prohibitin, PHB1, Phb1p, Ph- B, Phb, PHB-1 |
| ZMYM2 | ENSP00000479904 | MYM, ZNF198, ZMYM2, zinc finger,  MYM-type 2, M-YM |

| | | |
|---|---|---|
| SLC46A1 | ENSP00000480703 | HCP 1, HCP1, PCFT, SLC46A1, heme carrier protein 1, hHCP1, proton-coupled folate transporter, PCFT/HCP1, HCP-1, proton coupled folate transporter, heme-carrier protein 1, heme carrier protein-1 |
| BCL2L13 | ENSP00000480836 | BCL2 family, bcl-2 family, Bcl-2-family, BCL2-family, BCL2L13, Bcl-rambo, mIL-1, Bcl2-like 13, Bcl 2-family, Bcl-2-like protein 13 |
| BCL2L12 | ENSP00000482218 | BCL-2 family, Bcl2L12, BCL2 family, Bcl2-like-12, Bcl-2-family, BCL2-family, Bcl2-Like12, BCL2-like 12, Bcl 2-family, BPR, Bcl2-like protein 12, BCL2 like 12, Bcl2 Like-12, B-PR, Bcl2 like protein-12, Bcl2 like protein 12, Bcl2-like protein-12 |
| MUC1 | ENSP00000484824 | MUC1, MCKD1, MUC1/ZD, PUM, EMA, episialin, peanut-reactive urinary mucin, mucin 1, CA 15-3, polymorphic epithelial mucin, MUC-1, MUC1/SEC, Mucin1, CA15-3, mucin-1, ADMCKD1, CD227, PEMT, KL-6, H23Ag, MUC1/X, hMUC1, MUC 1, Krebs von den Lungen-6, cancer antigen 15-3, CA153, hMUC-1, MAM-6, mucin 1, cell surface associated, KL6, cancer antigen 153, Cancer Antigen15-3, Krebs von den Lungen 6, Tumor-Associated Mucin, CA-15-3 |
| MT-TP | MT-TP | tRNP, MTTP, MT-TP |
| PWAR1 | PWAR1 | PAR1, par-1, Par 1, hPar1, PWAR1 |
| RALBP1 | ENSP00000019317 | RLIP76, RalBP1, RIP1, Rip, Rip-1, RLIP1, 76-kDa Ral-interacting protein, RalA binding protein 1, RalA-binding protein 1, RalA binding protein-1 |
| PNPLA3 | ENSP00000216180 | Adiponutrin, PNPLA3, patatin-like phospholipase domain-containing protein 3, iPLA2epsilon, EC 3.1.1.3, ADPN, patatin-like phospholipase domain-containing 3, patatin-like  phospholipase domain-containing 3, patatin-like phospholipase domain containing 3, PNPLA3 p, Patatin-like phospholipase domain containing-3, PNPLA 3, Patatin-like phospholipase domain containing, patatin-like phospholipase domain-containing protein-3, PNPLA-3, patatin-like phospholipase domain-containing-3, acylglycerol O-acyltransferase, patatin-like phospholipase domain-containing protein3, patatin-like phospholipase domain containing-protein 3, patatin-like phospholipase domain containing protein 3, patatin like phospholipase domain-containing protein 3 |

| | | |
|---|---|---|
| SULT2A1 | ENSP00000222002 | hydroxysteroid sulfotransferase, ST2, hSTa, hST, HST2, DHEA-ST, DHEA ST, SULT2A1, hst-2, Dehydroepiandrosterone sulfotransferase, h-ST-a, ST-2, StD, sulfotransferase 2a1, St-D, Hs-T, s t d |
| C5 | ENSP00000223642 | C5a, C5D, HC5, complement C5, C5a anaphylatoxin, Hc-5, hC5a, 5 HCC, complement component 5, C-5B, C5 A, c-5p |
| RASD1 | ENSP00000225688 | DexRas1, ras-related protein, AGS1, activator of G protein signaling, RASD1, dexamethasone-induced Ras-related protein 1, Ras related protein, Ras-related dexamethasone-induced 1, AGS 1 |
| ZPR1 | ENSP00000227322 | Zpr1, zinc finger protein ZPR1, zinc-finger protein ZPR1, ZPR1 Zinc Finger, ZNF259, zinc finger protein 259, ZINC fingers |
| SART3 | ENSP00000228284 | hSART3, p110, Tip110, p100, SART3, DSAP1, p110(nrb, Tat-interacting protein of 110 kDa, Squamous cell carcinoma antigen recognized by T cells 3 |
| GAPDH | ENSP00000229239 | glyceraldehyde-3-phosphate dehydrogenase, glyceraldehyde 3-phosphate dehydrogenase, GAPD, GAPDH, glyceraldehyde 3 phosphate dehydrogenase, G3PD, glyceraldehyde-3-phosphate-dehydrogenase, glyceraldehyde-3 phosphate dehydrogenase, glyceraldehyde-3-phosphate dehy-drogenase, EC 1.2.1.12 |
| CHPT1 | ENSP00000229266 | hCPT1, cpt, CPT1, c Pt, CPT-1 |
| SPARC | ENSP00000231061 | cysteine-rich protein, osteonectin, CRP, BM-40, BM40, Secreted protein acidic and rich in cysteine, secreted protein, acidic, cysteine-rich (osteonectin, Sparc, secreted-protein-acidic-and-rich-in-cysteine, SPARC family, secreted-protein acidic and rich in cysteine, secreted protein acidic and cysteine rich |
| NCK2 | ENSP00000233154 | Grb4, Nckbeta, Nck2 |
| GHSR | ENSP00000241256 | GHS-R, growth hormone secretagogue receptor, GHSR, ghrelin receptor, GH-releasing peptide receptor, ghrelin-receptor, growth-hormone secretagogue receptor |
| BCL2L2 | ENSP00000250405 | BCL2 family, bcl-2 family, Bclw, bcl-w, Bcl-2-family, bcl -w, BCL2-family, BCL2L2, BCL2-like2, Bcl 2-family, BCL2-Like Protein 2, BCL2 like 2, Bcl-2-like-2 |
| ESS2 | ENSP00000252137 | TSSK 2, ES-2, DGSI, ES2, HES2, DiGeorge syndrome critical region 14, Dgcr14, Tsk2, DiGeorge syndrome critical region gene 14, BIS-1, BIS1 |
| PEMT | ENSP00000255389 | PEMT, PNMT, PEMT2, phosphatidylethanolamine N-methyltransferase, PEMPT |

| | | |
|---|---|---|
| CLPS | ENSP00000259938 | colipase, co-lipase, pancreatic colipase, pancreatic co-lipase, Clps |
| BCL2L10 | ENSP00000260442 | BCL2 family, Bcl-B, BCL-2 family, Bcl2-L-10, BCL2L10, Bcl-2-family, BCL2-family, BCLb, Bcl 2-family |
| LMNB1 | ENSP00000261366 | LMNB1, lamins, lamin B1, Lmnb, Lamin-B1, LAMINB1 |
| VWF | ENSP00000261405 | VWF, von Willebrand factor, F8vWF, 1-ATZ, vonWillebrand factor, von-Willebrand-factor, v-WF, von-Willebrand factor, von Willebrand-factor |
| MAP3K8 | ENSP00000263056 | Tpl2, Tpl-2, cot, Tumor progression locus-2, mitogen-activated protein kinase kinase kinase 8, MAP3K8, AURA2, cancer Osaka thyroid oncogene, Tumor progression locus 2 |
| SYP | ENSP00000263233 | synaptophysin, SYP, SY P, synapto-physin, Major Synaptic Vesicle Protein p38 |
| BIRC3 | ENSP00000263464 | hIAP-1, API2, aip-1, AIP1, c-IAP2, HIAP1, MIHC, cIAP-2, inhibitor of apoptosis protein-1, cIAP2, c-IAP-2, apoptosis inhibitor-2, apoptosis inhibitor 2, IAP-1, cellular inhibitor of apoptosis-2, cellular inhibitor of apoptosis 2, IAP1, h-IAP1, BIRC3, inhibitor of apoptosis protein 1, inhibitor-of-apoptosis protein 1, Aip1p, baculoviral iap repeat-containing 3, inhibitor of apoptosis protein1, BIRC 3 |
| DGUOK | ENSP00000264093 | deoxyguanosine kinase, dGK, DGUOK, EC 2.7.1.113, deoxy-guanosine kinase |
| CD80 | ENSP00000264246 | CD80, B7-1, HLA-B7, BB-1, BB1, B lymphocyte activation antigen B7, B7.1, hBB1, HB7, HLA B7, B-lymphocyte activation antigen B7, CD 80, CD80 antigen, CD80 molecule, HL-A-B7, Hb 7, HB-7, B-7.1, HLA-B 7 |
| CP | ENSP00000264613 | ceruloplasmin, CP2, CP-2, EC 1.16.3.1 |
| BNIP2 | ENSP00000267859 | BNIP-2, BCL-2 family, Nip2, BCL2 family, Bcl-2-family, Bcl-2/adenovirus E1B 19 kDa interacting protein 2, Bcl-2/adenovirus E1B 19kDa interacting protein 2, BNIP2, BCL2-family, BNIP 2, Bcl 2-family |
| SLC25A4 | ENSP00000281456 | HT1, ANT1, Adenine nucleotide translocator 1, Ant1p, Ant 1, SLC25A4, AAC1, HT-1, ANT-1, adenine nucleotide translocator-1, AAC-1, ADP/ATP translocase 1, AAC 1, HT 1, T1P |
| DGKE | ENSP00000284061 | dGK, DGKE, diacylglycerol kinase epsilon |
| THY1 | ENSP00000284240 | Thy-1, Thy1, Thy-1-antigen, Thy-1 antigen, CD90, Thy-1 cell surface antigen, Thy 1, Thy-1-human, CD 90, hCD90, Thy-1- human, THY1 membrane glycoprotein, Thy-1 membrane glycoprotein, CDw90, Thy 1 antigen |

| | | |
|---|---|---|
| ACAA2 | ENSP00000285093 | HT1, mitochondrial 3-oxoacyl-CoA thiolase, HT-1, beta-ketothiolase, acetyl CoA acyltransferase, HT 1, Acaa2, acetyl-CoA acyltransferase 2, T1P |
| HMGCR | ENSP00000287936 | 3-hydroxy-3-methylglutaryl coenzyme A reductase, HMGCR, EC 1.1.1.34, hydroxymethylglutaryl-CoA reductase, 3-hydroxy-3-methylglutaryl CoA reductase, 3-hydroxy-3-methylglutaryl-Coenzyme A reductase, 3-hydroxy-3-methyl-glutaryl coenzyme A reductase, 3-hydroxy-3-methyl glutaryl-CoA reductase, hydroxymethyl glutaryl-CoA reductase, 3-Hydroxy-3-methylglutaryl--coenzyme A reductase, 3-hydroxy-3-methylglutaryl-CoA reductase, 3-hydroxy-3-methyl-glutaryl-CoA reductase, 3-hydroxy-3-methyl-glutaryl coenzyme-A reductase, 3-hydroxy-3-methylglutaryl coenzyme-A reductase, 3 CD5, 3-hydroxy-3methylglutaryl CoA reductase, hydroxy-methyl-glutaryl CoA reductase, 3-hydroxy-3-methyl-glutaryl-coenzyme A reductase, 3-hydroxy-3-methylglutaryl-coenzyme-A-reductase, hydroxymethylglutaryl CoA reductase, 3-hydroxy-3methyl glutaryl Coenzyme A reductase, 3-hydroxy-3-methylglutaryl-co-enzyme A reductase, 3-hydroxy-3-methylglutaryl- coenzyme A reductase, 3-hydroxy-3-methylglutaryl-coenzyme-A reductase, 3-hydroxy-3-methylglutarylCoA reductase, hydroxy methylglutaryl-CoA reductase, hydroxymethyl glutaryl CoA reductase |
| REL | ENSP00000295025 | Rel, c-Rel, I-Rel, cRel, proto-oncogene c-Rel, c REL, protooncogene c-REL, v-rel avian reticuloendotheliosis viral oncogene homolog, REL proto-oncogene, NF-kB subunit |
| TGFA | ENSP00000295400 | transforming growth factor-alpha, TGFA, TFGA, Transforming growth factor alpha, transforming growth-factor alpha, transforming-growth-factor-alpha, 3 TGF, TGF-a, transforming growth factor-a, 2 TGF, pro-transforming growth factor alpha, 2-TGF |
| RICTOR | ENSP00000296782 | rictor, mTOR complex-2, rapamycin-insensitive companion of mTOR, rapamycininsensitive companion of mTOR, AVO3p, mTOR complex 2, mTor-complex-2, RPTOR-independent companion of MTOR complex 2, PI-a, mTOR complex2, PiA |
| TERF2IP | ENSP00000300086 | Rap1, Rap1p, Rap-1, TERF2IP, hRap1 |
| CDT1 | ENSP00000301019 | dup, Cdt1, hCdt1, Cdt-1, DNA replication factor Cdt1, chromatin licensing and DNA replication factor 1 |

| | | |
|---|---|---|
| FADD | ENSP00000301838 | protein FADD, FADD, MORT1, death effector domain containing, MORT-1, Fas-associated death domain protein, Fas-associated via death domain, Fas-associating protein with death domain, Fas (TNFRSF6)-associated via death domain, Fas associated via death domain |
| LGALS4 | ENSP00000302100 | GAL4, galectin 4, galectin-4, LGALS4, Gal4p, Gal-4, GAL 4 |
| AXIN2 | ENSP00000302625 | conductin, AXIN2, Axis inhibition protein 2, axin-2, Axin 2, Axis inhibition protein2 |
| UGT2B7 | ENSP00000304811 | UGT2B7, UGT2B9, UDP-glucuronosyltransferase 2B7, UDPGTh-2, UDP-glucuronosyltransferase-2B7, UDP glucuronosyltransferase 2B7 |
| RPTOR | ENSP00000307272 | raptor, MIP1, regulatory associated protein of mTOR,  complex 1, mTOR complex 1, MIP-1, regulatory associated protein of mTOR, Mip1p, p150 target of rapamycin (TOR)-scaffold protein, hMIP-1, regulatory-associated protein of mTOR, mTOR complex1, RPTOR, mTOR complex-1, regulatory associated protein of mTOR complex 1, KOG-1 |
| MCM7 | ENSP00000307288 | MCM family, MCM7, MCM2, Mcm2p, hCDC47, p85Mcm, CDC47, CDC 47, MCM-2, minichromosome maintenance complex component 7, DNA replication licensing factor MCM7, MCM-7 |
| GPD2 | ENSP00000308610 | mGPDH, GPDm, GDH2, mtGPD, GPD2, hGDH2, glycerol-3-phosphate dehydrogenase 2, GP-DM |
| BCL2L14 | ENSP00000309132 | BCL2 family, Bcl-G, Bcl-2-family, BCL-2 family, BCL2-family, BCLG, BCL2L14, Bcl 2-family, Bcl2-like 14 |
| RBBP6 | ENSP00000317872 | P2P-R, PACT, RBQ-1, RBBP6, Proliferation potential-related protein, RBQ1, retinoblastoma binding protein 6, PP-RP |
| PRKRA | ENSP00000318176 | RAX, PACT, PRKRA, DYT16, protein activator of the interferon-induced protein kinase, PKR associated protein X |
| CXCR2 | ENSP00000319635 | IL8RA, CXCR2, Il8rb, IL-8RB, IL8R2, IL-8R2, IL8RBP, chemokine receptor 2, IL-8RA, Cmkar2, CXCR-2, CXC-chemokine receptor type 2, chemokine receptor-2, C-X-C motif chemokine receptor 2, chemokine (C-X-C motif) receptor 2, CD182, C-X-C motif chemokine receptors, C-X-C-motif chemokine receptor 2, C-X-C chemokine receptor type 2, CXC motif chemokine receptor 2, chemokine receptor- 2 |
| RHBDF2 | ENSP00000322775 | iRhom2, RHBDF2, rhomboid family, FLJ22341, Rhomboid family member 2, inactive rhomboid protein 2, rhomboid 5 homolog 2, iRhom-2 |

| | | |
|---|---|---|
| SLC2A2 | ENSP00000323568 | GLUT2, Glut-2, SLC2A2, GLUT 2, solute carrier family 2 (facilitated glucose transporter), member 2, hGLUT2 |
| TRPM8 | ENSP00000323926 | TRPM8, Trp-p8, Transient receptor potential cation channel subfamily M member 8, transient receptor potential cation channel, subfamily M, member 8, hTRPM8 |
| TCHP | ENSP00000324404 | tumor suppressor protein, tumor-suppressor protein, MITOSTATIN, tumor-suppressor-protein, trichoplein keratin filament-binding protein, Tpms |
| SYT9 | ENSP00000324419 | synaptotagmins, Synaptotagmin IX, synaptotagmin-9, SYT9, Synaptotagmin 9 |
| KIR3DL2 | ENSP00000325525 | p140, killer cell immunoglobulin-like receptors, 3DL2, KIR, killer-cell immunoglobulin-like receptors, Cl-5, KIR3DL2, CD158k, CL5, killer cell immunoglobulinlike receptors, killer cell immunoglobulin like receptors, Killer Cell Immunoglobulin- Like Receptors, Killer Cell Immunoglobulin-like Receptor 3DL2, KIR-3DL2 |
| CCR8 | ENSP00000326432 | CCR8, TER1, CY6, CKR-L1, chemokine receptor-like 1, C-C motif chemokine receptors |
| OSBP2 | ENSP00000332576 | ORP4, OSBP2, OSBPL1, oxysterol-binding proteins, oxysterol binding proteins, Osbpl-1, oxysterol-binding protein-related protein 4 |
| TSLP | ENSP00000339804 | thymic stromal lymphopoietin, TSLP |
| MAOA | ENSP00000340684 | monoamine oxidase A, MAOA, MAO A, Mao-a, EC 1.4.3.4, monoamine oxidase type A, monoamine oxidase-A, monoamine-oxidase A, mono-amine oxidase-A, mono-amine-oxidase type A, monoamine-oxidase-A, monoamine oxidase type-A |
| MAP2K3 | ENSP00000345083 | MEK3, MKK3, MKK-3, MAP kinase-kinase 3, Map Kinase Kinase 3, MAP2K3, mitogen-activated protein kinase kinase 3, MKK 3, Mitogen-activated protein kinase kinase-3, mitogen-activated protein kinase-kinase 3 |
| CLDN7 | ENSP00000353475 | claudin-1, Claudin-7, CLDN-7, CLDN7, claudin 1, Claudin7, claudin 7, claudin1 |
| CNTF | ENSP00000355370 | ciliary neurotrophic factor, CNTF, hCNTF |
| PRKN | ENSP00000355865 | parkin, PARK2, E3 ubiquitin-protein ligase Parkin, PRKN, PARK 2, Park-2, AB009973, E3 ubiquitin protein ligase Parkin, parkin RBR E3 ubiquitin protein ligase, Parkin RBR E3 ubiquitin-protein ligase |

| | | |
|---|---|---|
| EPRS1 | ENSP00000355890 | PARs, GluProRS, EPRS, QARS, glutamyl-prolyl tRNA synthetase, bifunctional aminoacyl-tRNA synthetase, glutamyl-prolyl-tRNA synthetase, Glns, glutamyl prolyl tRNA synthetase, bifunctional glutamate/proline-tRNA ligase, Glutamyl-prolyl-tRNA-synthetase |
| ESRRG | ENSP00000355904 | ERR3, ERRgamma, Estrogen-related receptor gamma, ESRRG, estrogen receptor-related protein 3, NR3B3, ERRgamma2, ERRG, estrogen-related receptor-gamma, E srrg |
| HSD11B1 | ENSP00000355995 | 11 beta HSD1, 11beta-HSD 1, 11betaHSD1, 11beta-hydroxysteroid dehydrogenase 1, HSD11, HSD11L, 11 beta-HSD1, 11 beta-hydroxysteroid dehydrogenase-1, 11beta-HSD1, 11beta-HSD-1, 11beta -HSD1, 5 pg x, HSD11B1, 11beta-hydroxysteroid dehydrogenase-1, 11betaHSD-1, hydroxysteroid dehydrogenase 1, HSD11B, Hydroxysteroid dehydrogenase-1, hydroxysteroid-dehydrogenase 1, hydroxysteroid (11-beta) dehydrogenase 1, EC 1.1.1.146, Hydroxysteroid 11-Beta Dehydrogenase 1, hydroxysteroid 11-beta dehydrogenase-1, Hydroxysteroid dehydrogenase - 1 |
| CSRP1 | ENSP00000356275 | CRP1, Csrp, CRP, cysteine and glycine rich protein 1, CSRP1, CRP 1, C-RP, Cysteine-rich protein 1, cysteine- and glycine-rich protein 1, HCRP1, CR P |
| ATF6 | ENSP00000356919 | ATF6, Activating transcription factor 6, ATF-6, ATF6A, activating transcription factor 6A, activating transcription factor-6, activating transcription factor 6 alpha |
| BAG3 | ENSP00000358081 | BAG-3, Bcl-2-associated athanogene 3, Bag3, Bcl-2 associated athanogene-3, BAG3 p, BCL2-associated athanogene 3, Bcl-2-associated athanogene-3, Bcl-2-associated athanogene family, Bcl-2 associated athanogene 3, CAIR-1, Bcl-2 associated athanogene 3, BCL2-associated athanogene-3, BCL2 Associated Athanogene 3, BCL2- associated athanogene 3, Bcl-2 associated-athanogene-3, Bcl-2-associated athanogene3, BCL2-associated athanogene family |
| CNR1 | ENSP00000358513 | CB1R, Cannabinoid receptor 1, Cbr, CNR1, Cnr-1, cannabinoid receptor-1, CB1-P, cnr, CB1-R, CNR1 human, cannabinoid receptor1, hCB1, CB-1R, hCB1R |

| | | |
|---|---|---|
| NRAS | ENSP00000358548 | HRAS1, N-Ras, NRAS, neuroblastoma RAS viral oncogene homolog, Hras-1, H-ras-1, H-ras 1, H-ras1, neuroblastoma RAS viral (v-ras) oncogene homolog, 3-con, NRAS1, NRAS proto-oncogene, GTPase, GTPase NRas, neuro-blastoma RAS viral oncogene homolog |
| RAP1A | ENSP00000358723 | KREV1, Rap1A, Rap1, smg p21, Krev-1, c21KG, K rev-1, Rap-1, KREV1P, hRap1, Rap1p, K-rev-1, Ras-related protein Rap-1A, RAP1A, member of RAS oncogene family, rap 1A |
| FMR1 | ENSP00000359506 | FMR1, FMRP, Fragile X mental retardation-1, FRAXA, fragile X mental retardation 1, FMR-1, fragile X mental retardation protein 1, POF, POF1, fragile-X mental retardation 1, fragile-X mental-retardation 1, hFMR1, FMR 1, Fragile X mental-retardation 1, fragile X mental retardation1, fragile-X mental retardation-1, 2 FMR |
| PTPN1 | ENSP00000360683 | PTP-1B, PTP1B, protein tyrosine phosphatase 1B, PTPN1, protein-tyrosine phosphatase 1B, Protein tyrosine phosphatase-1B, protein-tyrosine-phosphatase 1B, hPTP1B, Protein tyrosine phosphatase 1-B, PTP 1B, protein-tyrosine phosphatase-1B, 2 CNI, protein tyrosine phosphatase 1 B, Tyrosine-protein phosphatase non-receptor type 1, protein tyrosine-phosphatase 1B, protein tyrosine phosphatase1B, Protein tyrosine phosphatase, non-receptor type 1 |
| PREX1 | ENSP00000361009 | P-Rex1, Prex1, phosphatidylinositol-3, 4, 5-trisphosphate-dependent Rac exchange factor 1, PIP3-dependent Rac exchange factor-1, Phosphatidylinositol-3, 4, 5-Trisphosphate Dependent Rac Exchange Factor 1, phosphatidylinositol 3, 4, 5-trisphosphate-dependent Rac exchanger 1 protein, phosphatidylinositol 3, 4, 5-trisphosphate-dependent Rac exchange factor 1 |
| NOX1 | ENSP00000362057 | mox1, gp91-2, NOX1, Mox-1, NOX-1, NADPH oxidase-1, Hmox1, NOH-1, NADPH oxidase homolog 1, mitogenic oxidase, HMOX-1, NADPH oxidase 1, NADPH-oxidase 1, Nox 1, NADPH oxidase1 |
| ENG | ENSP00000362299 | endoglin, HHT 1, HHT1, CD105, HHT-1, ORW1, CD-105, eng, CD 105 |
| BAK1 | ENSP00000363591 | BCL2 family, Bak-like, BCL-2 family, Bak1, Bcl-2-family, Bcl-2 homologous antagonist/killer, BCL2-family, Bak-1, BCL2-antagonist/killer 1, Bcl 2-family, BCL2 antagonist/killer 1 |
| RALA | ENSP00000005257 | Ral, RalA, Ras-related protein Ral-A, Ral A, V-ral simian leukemia viral oncogene homolog A, Ral-A |

| | | |
|---|---|---|
| CD74 | ENSP00000009530 | p33, CD74, Ia gamma, HLA-DG, HLA-DR-gamma, 3 PGD, HLA class II histocompatibility antigen gamma chain, CD 74, Ia-associated invariant chain |
| FAP | ENSP00000188790 | DPPIV, FAP alpha, seprase, fibroblast activation protein alpha, DPP-IV, serine integral membrane protease, SIMP, 170-kDa melanoma membrane-bound gelatinase, FAPalpha, DPP IV, fibroblast activation protein-alpha, FAP-alpha, FA-P, Fap |
| AQP2 | ENSP00000199280 | AQP2, aquaporin-2, Aquaporin 2, AQP-CD, AQP-2, WCH-CD, aquaporin2, aquaporin-CD, AQP2-p, AQP 2, AQP2 p |
| PPP1R15A | ENSP00000200453 | GADD34, Ppp1r15a, GADD 34, protein phosphatase 1, regulatory subunit 15A, Growth Arrest And DNA-Damage-Inducible 34 |
| CDC6 | ENSP00000209728 | Cdc6, Cdc6p, p62(cdc6, hCdc6, CDC6-related protein, cell division cycle 6, cell division cycle 6 homolog, CDC-6 |
| GADD45B | ENSP00000215631 | MyD118, Gadd45b, Gadd45 beta, growth arrest and DNA-damage-inducible-beta, gadd45beta, hMyD118, growth arrest and DNA-damage-inducible beta, Growth arrest and DNA-damage-inducible, beta, growth arrest and DNA damage inducible b |
| SEC14L3 | ENSP00000215812 | TAP2, SEC14L3, Sec14-like 3, TAP-2 |
| GZMB | ENSP00000216341 | Granzyme B, Grb, GzmB, cytotoxic serine protease B, hCTLA-1, c11, CTLA-1, HLp, CGL1, CGL-1, CTLA 1, CSPB, CTLA1, CCPI, granzyme-B, CCP I, granzymeB, cytotoxic serine protease-B, CSP-B, GR B, Gzm-B |
| CHGA | ENSP00000216492 | chromogranin A, granins, chga, pancreastatin, chromogranin-A, vasostatin, parastatin, chromograninA |
| GPR50 | ENSP00000218316 | GPR50, melatonin-related receptor, HH9, G protein-coupled receptor 50, G-protein-coupled receptor 50, Mel1c, G protein coupled receptor 50 |
| CSK | ENSP00000220003 | c-Src kinase, Csk, c-src tyrosine kinase, C-terminal Src kinase, 3 EAC, c-Src-kinase |
| NEFM | ENSP00000221166 | NF-M, Intermediate filaments type IV, neurofilament-medium, neurofilament medium, NEFM, NFM, neurofilament-3, neurofilament medium polypeptide |
| PIK3R2 | ENSP00000222254 | p85, p85beta, Pik3r2, p85 beta, phosphoinositide 3-kinase regulatory subunit 2, phosphoinositide-3-kinase, regulatory subunit 2, phosphoinositide-3-kinase regulatory subunit 2, Phosphatidylinositol 3-kinase regulatory subunit beta |

| | | |
|---|---|---|
| C1QBP | ENSP00000225698 | p32, hp32, p33, gC1qR, C1QBP, gC1q-R, C1q globular domain binding protein, 1 p32, SF2p32, HABP1, gC1qR protein, hyaluronan binding protein 1, P3 2, complement component 1, q subcomponent binding protein, hyaluronan-binding protein 1, complement component 1, q subcomponent-binding protein, HABP-1 |
| COL1A1 | ENSP00000225964 | COL1A1, LRG1, Col 1a1, collagen alpha 1(I) chain, Z74615, AF017178, collagen alpha1(I) chain, collagen, type I, alpha 1, alpha-1 type I collagen, collagen type I alpha-1 chain, COL-1A1, collagen type I alpha 1 chain, collagen, type I, alpha-1, LRG-1, COL1A1 p, COL1A1-014, alpha 1 type I collagen, alpha1 type I collagen |
| AFM | ENSP00000226355 | alf, alpha-albumin, afamin, alb-2, Al-F, a-LF, AlbA, Alb-A |
| GNRHR | ENSP00000226413 | GnRHR, GnRH-R, gonadotropin-releasing hormone receptor, GRHR, gonadotropin releasing hormone receptor, LHRH-R, GNRHR1, gonadotropin-releasing hormone receptor, LHRHR, Gn-RHR |
| LTBR | ENSP00000228918 | CD18, lymphotoxin-beta receptor, Lymphotoxin beta-receptor, LTbetaR, LT beta-R, LT-betaR, lymphotoxin beta receptor, D12S370, LTBR, LT-beta R, LTbeta-R, LT beta R, lymphotoxin-beta-receptor, CD 18, hCD18, TNFR3, CD-18 |
| NANOG | ENSP00000229307 | Nanog, Nanog homeobox, homeobox protein NANOG |
| GPLD1 | ENSP00000230036 | PI-G PLD, GPI-PLD, GPLD1, phosphatidylinositol-glycan-specific phospholipase D, glycosylphosphatidylinositol-specific phospholipase D, glycosylphosphatidylinositol specific phospholipase D, pld, glycosylphosphatidylinositol specific phospholipase D1 |
| MSH2 | ENSP00000233146 | MSH2, hMSH2, MutS homologs, msh-2, MSH 2, HN-PCC, MutS homolog 2, Msh2p, DNA mismatch repair protein MSH2, hMSH-2, LCFS2, hmsh 2, MSH2 p, Mut-S-homolog-2, mutS homolog2, MutS protein homolog 2 |
| PLEK | ENSP00000234313 | pleckstrin, p47, Plek, platelet 47-kDa protein |
| FGF23 | ENSP00000237837 | FGF23, phosphatonin, FGF-23, fibroblast growth factor 23, fibroblast growth factor-23, HPDR2, FGF-2 3, hFGF23, fibroblast-growth factor 23, HypF, hFGF-23, FGF23-P, fibroblast growth factor23, FGF 23, FGF- 23, fibroblast-growth-factor 23, fibroblast-growth factor-23, ad HR, FGF -23, FGF-23-P, fibroblast-growth-factor-23 |
| KALRN | ENSP00000240874 | TRAD, Kalirin, CHD5, KALRN, protein Duo, Huntingtin-associated protein-interacting protein, HAPIP |

| | | |
|---|---|---|
| PLCG1 | ENSP00000244007 | phospholipase C-gamma1, phospholipase Cgamma1, PLCG1, PLC-gamma 1, Phospholipase C gamma 1, PLC-gamma1, PLC1, PLC-148, PLCgamma1, phospholipase C-gamma 1, phospholipase C gamma-1, phospholipase Cgamma-1, PLC-gamma-1, plc-1, PLCgamma-1, phospholipase C gamma1, phospholipase C,  gamma 1, PLC-g1, PLC gamma-1, PLC gamma 1 |
| MRPS7 | ENSP00000245539 | MRPs, RPS7, MRPS7, mitochondrial ribosomal protein S7, MRP-S |
| F2RL3 | ENSP00000248076 | PAR4, par-4, coagulation factor II receptor-like 3, F2RL3, coagulation factor II (thrombin) receptor-like 3, Par 4 |
| MCHR1 | ENSP00000249016 | MCH-1R, MCH1R, MCH-R1, SLC-1, melanin-concentrating hormone receptor 1, MCH1-R, MCHR1, GPR24, SLC1, melanin concentrating hormone receptor 1, G protein-coupled receptor 24, Melanin concentrating hormone receptor-1 |
| NDUFA2 | ENSP00000252102 | CD14, NDUFA2, cI-B8, CD1 4, CD-14, CD 14, hCD14 |
| MLLT1 | ENSP00000252674 | ENL, LTG19, Mllt1 |
| NR0B2 | ENSP00000254227 | SHP-1, SHP, small heterodimer partner, NR0B2, SHP1, orphan nuclear receptor SHP, Nuclear receptor subfamily 0,  group B, member 2, nuclear receptor subfamily 0 group B member 2 |
| JAG1 | ENSP00000254958 | Jagged1, JAG1, Jagged-1, AGS1, Jagged 1, Ahd, HJ1, JAG-1, JAG 1, AwS, AGS 1 |
| NTS | ENSP00000256010 | Neurotensin/neuromedin N, neurotensin, NT/N, neuromedin N, neuromedin-N, NTS-1, NTS1, nt S1, hNTS1, Nts, NT-s, proneurotensin/neuromedin, Pro-neurotensin/neuromedin |
| SORT1 | ENSP00000256637 | Sortilin, NT-3, Sort1, gp95, NT3, sortilin-1, neurotensin receptor-3, 100-kDa NT receptor, nt -3, NTR3, sortilin 1, Sort-1, hSORT1, NTR-3, Sortilin1, NT 3, SORT 1 |
| SMPD2 | ENSP00000258052 | neutral sphingomyelinase, nSMase, N-SMase, smpd2, nSMase1, Isc1 |
| BCO1 | ENSP00000258168 | beta, beta-carotene-15, 15'-dioxygenase, BCDO, BCO1, beta-carotene 15, 15'-monooxygenase 1, BCMO1, beta, beta-carotene 15, 15'-dioxygenase, beta-carotene oxygenase 1, BCMO |
| ACSBG1 | ENSP00000258873 | very long-chain acyl-CoA synthetase, hsBG, lpd, very-long-chain acyl-CoA synthetase, BG1, Lipidosin, ACSBG1, BG-1, HBG1, very long chain acyl-CoA synthetase |
| BUD13 | ENSP00000260210 | BUD13, BUD13 homolog |

| | | |
|---|---|---|
| CYP2C9 | ENSP00000260682 | CYP2C9, cytochrome P-450 2C9, CYP2C, cholesterol 25-hydroxylase, CYP 2C, CYP2C10, cytochrome P450 2C9, cholesterol-25-hydroxylase, Cytochrome P4502C9, CYP 2C9, Cyp-2c, cytochrome P-450 PB-1, s-mephenytoin 4-hydroxylase, cytochrome P450-2C9, Cholesterol 25 hydroxylase, P450IIC9, cytochrome P450 family 2 subfamily C member 9, cytochrome P450, family 2, subfamily C, polypeptide 9 |
| GALC | ENSP00000261304 | GALC, galactosylceramidase, galactocerebrosidase, galactosylceramide beta-galactosidase, EC 3.2.1.46, galactocerebroside beta-galactosidase, galactosylceramide-beta-galactosidase, galactosyl ceramide beta-galactosidase, galacto-cerebrosidase, galactosyl-ceramidase |
| PEBP1 | ENSP00000261313 | RKIP, neuropolypeptide h3, PEBP, Raf kinase inhibitory protein, Raf kinase inhibitor protein, HCNP, hippocampal cholinergic neurostimulating peptide, HCNP-pp, raf kinase-inhibitory protein, Raf-kinase inhibitory protein, pBP, HCNPpp, PEBP1, phosphatidylethanolamine-binding protein 1, phosphatidylethanolamine-binding protein-1, Raf-kinase inhibitor protein, Raf-kinase-inhibitor protein, phosphatidylethanolamine binding protein 1 |
| CYBA | ENSP00000261623 | p22 phox, p22-phox, CYBA, p22phox, Cytochrome B-245, Alpha Polypeptide, cytochrome b-245 light chain, cytochrome b-245 alpha chain |
| ARG2 | ENSP00000261783 | Arg2, arginase, arginase-2, arginase II, type II arginase, Arg 2, Arginase 2, Arg-2, arginase2, arginase-II |
| CHD5 | ENSP00000262450 | NuRD complex, chromodomain helicase DNA-binding protein 5, CHD5, chromodomain helicase DNA binding protein 5, Chromodomain-helicase-DNA-binding protein 5, Chromodomain-helicase DNA binding protein 5 |
| CBFA2T2 | ENSP00000262653 | p85, MTGR1, CBFA2T2, MTG8-like protein, core-binding factor, runt domain, alpha-subunit 2 |
| PPP1R12C | ENSP00000263433 | p85, AAVS1, p84, PPP1R12C |
| TYRO3 | ENSP00000263798 | Tyro3, Tyro 3, TYRO3P, rse, rek, Tyro-3, TYRO3 human, Dtk, t-IF, tyrosine-protein kinase receptor Tyro3 |

| | | |
|---|---|---|
| DNMT3A | ENSP00000264709 | DNMT3A, hDNMT3a, Dnmt3a2, DNA methyltransferase 3a, EC 2.1.1.37, DNMT3A p, DNA-methyltransferase 3A, DNMT 3A, DNA methyltransferase-3a, DNA methyl transferase 3a, DNA methyltransferase 3 alpha, DNA-methyltransferase-3a, DNMT-3a, DNA (cytosine-5-)-methyltransferase 3A, DNA (cytosine-5-)-methyltransferase 3 alpha, DNA (cytosine-5)-methyltransferase 3A, DNA methyltransferase 3 A, DNA methyl-transferase 3A, DNA (cytosine-5)-methyltransferase 3 alpha, DNA methyltransferase-3-alpha, DNA methyl transferase 3 alpha, AC012074, T-B-RS |
| OPRK1 | ENSP00000265572 | Oprk1, hOPRK1, KOR, opioid receptor kappa-1, KOR-1 |
| CPT1A | ENSP00000265641 | cpt1, carnitine palmitoyltransferase 1A, CPT1A, hCPT1, EC 2.3.1.21, L-CPT1, CPT1 A, CPT 1A, CPT1-A, carnitine palmitoyltransferase 1a (liver, CPT-1a, CPT-1, carnitine-palmitoyltransferase 1A, carnitine palmitoyltransferase-1A, carnitine palmitoyl transferase 1a, carnitine palmitoyl-transferase 1a, Carnitine palmitoyl transferase-1A, carnitine palmitoyltransferase1A, carnitine palmitoyl transferase 1 A, carnitine palmitoyltransferase -1a |
| ANK1 | ENSP00000265709 | Ank, erythrocyte ankyrin, ankyrin 1, Ankyrin-1, ANK1, ankyrin R, Ank-1, ankyrinR, ankyrin-R, HSph2, Hank-1, HSPH1, Ank 1, ankyrin1, HANK1 |
| DIDO1 | ENSP00000266070 | DIO-1, Dido3, Dio1, hdio1, Death inducer-obliterator 1, death inducer-obliterator-1, Dido2, DATF1, DIDO1 |
| CDH11 | ENSP00000268603 | cadherin-11, CDH11, OB-cadherin, cadherin 11, osteoblast cadherin, osteoblast-cadherin, cad11, cad-11, OB cadherin, CDH-11 |
| FCGR2A | ENSP00000271450 | FcgammaRIIA, FCGR, FCGR2A, CDw32, CD32A, Fc gamma RIIA, Fc gamma receptor IIA, CD32, FCGR2, FCGR2C, Fcgamma receptor IIA, FCG2, IGF-R2, Fc gammaRIIa, Fc-gamma receptor IIa, Fc gamma-receptor IIA, Fcgamma RIIa, IGFR2, IGFR-2, low affinity immunoglobulin gamma Fc region receptor II-a, FCGR2A p, immunoglobulin G Fc receptor II, Fc fragment of IgG receptor IIa |

| | | |
|---|---|---|
| RASA1 | ENSP00000274376 | p120 Ras GAP, p120, p120GAP, p120-RASGAP, p120RasGAP, RASA1, rasGAP, Ras-GAP, Ras GAP, p120Ras-GTPase-activating protein, p120 rasGAP, RAS p21 protein activator 1, p120 ras GTPase-activating protein, p120 rasGTPase-activating protein, p120-GAP, p120ras-GAP, RAS p21 protein activator, p120 GAP, RASA-1, p120 Ras-GAP, Ras GTPase-activating protein 1, capillary malformation arterio-venous malformation |
| CDK16 | ENSP00000276052 | PCTAIRE 1, PCTK1, PCTAIRE-1, Pctaire, CDK16, PCTAIRE1, PCTAIRE protein kinase 1, cyclin-dependent kinase 16 |
| CDKN2B | ENSP00000276925 | p15, p15INK4b, cyclin-dependent kinase inhibitor 2B, MTS2, CDKN2B, INK4B, p14INK4B, CDK4I, p15 INK4b, cyclin-dependent kinase inhibitor 2B (p15, inhibits CDK4, Mts-2, Cyclin-Dependent Kinase Inhibitor-2B, cyclin dependent kinase inhibitor 2B, TP15, cyclin dependent kinase inhibitor-2B, cyclin-dependent kinase 4 inhibitor B, p 15, multiple tumor suppressor 2 |
| PIGF | ENSP00000281382 | Pig-f, Pigf, p-IGF |
| CAMK4 | ENSP00000282356 | Camk4, CaMKIV, Camk-4, CaM Kinase IV, CaMK, Calcium/calmodulin-dependent protein kinase IV, CaM-kinase IV, CaM-K, Calcium/calmodulin-dependent protein kinase type IV, CaMK IV |
| CA2 | ENSP00000285379 | carbonic anhydrase II, CAII, 1 Ca2, CA II, Car 2, Car-2, EC 4.2.1.1, 2 Ca2, CA-II, carbonic anhydrase-2, cA2, 3 Ca2, Car2, 5 dog, 4 Ca2, CA 2, 1 cal, CA-2, HCA2, 2 h 15, 5 FLs, 5 ULN, 3 RGE, Carbonic anhydrase 2, cac, 3 MHC, HCAR2, 5 AML, 5 FNH, 3R-16, 2 OSM, carbonic anhydrase-II, 2 HNC |
| NCF1 | ENSP00000289473 | p47-phox, NCF-1, p47phox, p47 phox, NCF1, neutrophil cytosolic factor 1, Ncf1--a, NCF-47k, neutrophil cytosolic factor-1 |
| EHMT2 | ENSP00000364678 | G9a, hBAT8, hG9a, BAT8, EHMT2, euchromatic histone-lysine N-methyltransferase 2, euchromatic histone-lysine-methyltransferase 2, euchromatic histone lysine N-methyltransferase 2, euchromatic histone lysine N-methyltransferase-2, EHMT-2, euchromatic histone lysine methyltransferase 2, KMT1C |
| BAG6 | ENSP00000365131 | HLA-B associated transcript 3, HLA-B-associated transcript 3, G3-P, BCL2-associated athanogene 6, HG3, BCL2-associated athanogene family |
| PCCA | ENSP00000365462 | PCCA, pcc A, EC 6.4.1.3, propionyl-CoA carboxylase alpha subunit |

| | | |
|---|---|---|
| CTTN | ENSP00000365745 | cortactin, EMS1, CTTN, EMS 1, Src substrate cortactin, EMS-1 |
| MTHFR | ENSP00000365777 | MTHFR, Methylenetetrahydrofolate reductase, methylene-tetrahydrofolate reductase, 5, 10-methylenetetrahydrofolate reductase, 5,  10-methylenetetrahydrofolate reductase, methylene tetrahydrofolate reductase, 5, 10-methylene tetrahydrofolate reductase, 5, 10 methylene tetrahydrofolate reductase, 5, 10 Methylenetetrahydrofolate reductase, methylenetetrahydrofolatereductase, methylenetetrahydrofolate-reductase, methylene- tetrahydrofolate reductase, EC 1.5.1.20, methylenetetra- hydrofolate reductase, methylene tetrahydrofolatereductase, methylene-tetrahydro-folate-reductase, methylenetetra-hydrofolate reductase, 5, 10-methylenetetrahydrofolatereductase, 5, 10-Methylene-tetrahydrofolate reductase, methylene-tetra-hydrofolate reductase, methylenetetrahydro-folate reductase, methylenetetrahydro folate reductase, methylene-tetrahydrofolate-reductase, 5, 10-methylenetetra- hydrofolate reductase, 5,  10-methylene tetrahydrofolate reductase, MTHF R, Methyl- enetetrahydrofolate reductase, methylenetetrahydro-folate-reductase, methylenetetra hydrofolate reductase, methylenetet rahydrofolate reductase, 5, 10 methylenete-trahydrofolate reductase, 5, 10- methylenetetrahydrofolate reductase, 5,  10-methylene-tetrahydrofolate reductase, methylenetetrahydrofolate reductase (NAD(P)H, methylene tetrahydrofolate reductase, methyl enetetrahydrofolate reductase, 5,  10- methylenetetrahydrofolate reductase, methylene tetra-hydrofolatereductase, methylenetetrahy-drofolate reductase, 5, 10 methylenetetrahydrofolate reductase |
| BMI1 | ENSP00000365851 | Bmi-1, Bmi1, PCGF4, polycomb complex protein Bmi1, B lymphoma Mo-MLV insertion region 1 homolog, B lymphoma Mo-MLV insertion region-1 homolog, Polycomb complex protein BMI-1, BMI1 proto-oncogene,  polycomb ring finger, BMI-1 proto-oncogene,  polycomb ring finger |
| COL2A1 | ENSP00000369889 | COL2A1, STL1, collagen type IIa1, collagen,  type II,  alpha 1, collagen type II alpha 1, collagen type II alpha-1, collagen type II alpha1, alpha-1 type II collagen, Collagen type II alpha 1 chain, alpha1 type II collagen |
| NKX3-1 | ENSP00000370253 | NKX3.1, Nkx3-1, Nkx-3.1, NKX3A, homeobox protein NKX3.1, Nkx3, NKX 3.1, NK3 homeobox 1 |
| CTNS | ENSP00000371294 | cystinosin, CTNS, cystinosin,  lysosomal cystine transporter |
| RAD51 | ENSP00000372088 | RAD51, RecA, hRad51, Rad51 recombinase, Rad-51, rec A, HsRAD51, MRMV2, RAD51 homolog, Rad 51, BRCC5, Rad 51 homolog, DNA repair protein RAD51 homolog 1 |

| | | |
|---|---|---|
| PAX3 | ENSP00000375921 | Pax3, HuP2, paired box gene 3, paired box 3, Pax-3, paired boxes, Pax 3, paired box-3 |
| PDK1 | ENSP00000376352 | PDK1, PDK-1, pyruvate dehydrogenase kinase 1, pyruvate dehydrogenase kinase-1, PDHK1, pyruvate dehydrogenase kinase1 |
| HMMR | ENSP00000377492 | RHAMM, Hmmr, receptor for hyaluronan-mediated motility, hyaluronan-mediated motility receptor, IHABP, CD168, hyaluronan mediated motility receptor (RHAMM, H MMR, CD 168, receptor for hyaluronan mediated motility, hyaluronan-mediated motility receptor (RHAMM, hyaluronan mediated motility receptor, intracellular hyaluronic acid binding protein |
| SPOCK1 | ENSP00000378401 | testican-1, SPOCK1, testican, TIC-1, testican 1, SPOCK, SPARC/osteonectin, cwcv and kazal-like domains proteoglycan 1, Sparc/osteonectin, cwcv and kazal-like domains proteoglycan, SPARC family, SPARC/osteonectin, cwcv and kazal like domains proteoglycan 1 |
| HLA-A | ENSP00000379873 | HLA- A, HLA-A, HLA A, HLA -A, HLA--A, HLAA, major histocompatibility complex, class I, A, HLA-A201 |
| RNASE1 | ENSP00000381057 | Rac1, ribonuclease A, RAC-1, Rib-1, RNase 1, Rib1, Rac 1, rNS1, RNase1, h_rac1, HP-RNase, ribonuclease 1, RNase-1 |
| TCF4 | ENSP00000381382 | TCF-4, ITF2, TCF4, SEF2, SEF-2, SEF2-1, SEF2-1B, hTCF-4, transcription factor 4, E2-2, ITF-2, PTHs, e22, transcription factor-4, immunoglobulin transcription factor-2, hTCF4, transcription-factor 4, Immunoglobulin transcription factor 2 |
| NPY | ENSP00000384364 | NPY, Neuropeptide Y, neuropeptide-Y, prepro-neuropeptide Y, preproneuropeptide Y, N PY, prepro neuropeptide Y, pre-pro-neuropeptide Y, NP-Y, pro-Neuropeptide Y |
| XPO1 | ENSP00000384863 | Crm1, CRM-1, XPO1, hCRM1, Emb, Exportin 1, Crm1p, exportin-1, exportin1, EXP1, EXP-1, XPO-1, chromosome region maintenance 1 homolog |
| SLC25A19 | ENSP00000385312 | Slc25a19, Mup-1, mitochondrial uncoupling protein1, MUP1, dnc, mitochondrial uncoupling protein-1, DN-c, dn c, mitochondrial uncoupling protein 1, solute carrier family 25 member 19, Mitochondrial Thiamine Pyrophosphate Carrier, THMD4 |
| CLDN5 | ENSP00000385477 | claudin-5, BEC-1, TMVCF, transmembrane protein deleted in VCFS, BEC1, CLDN5, CLDN-5, claudin 5, claudin5 |
| ZBTB10 | ENSP00000387462 | ZBTB10, zinc finger and BTB domain containing 10 |

| | | |
|---|---|---|
| NFATC1 | ENSP00000389377 | NF-ATc1, NFATc1, nuclear factor of activated T cells-1, nuclear factor of activated T-cells, cytoplasmic 1, Nfatc, NF-ATc, NFAT2, nuclear factors of activated T cells, nuclear factor of activated T cells 1, NFAT-2, nuclear factor of activated T cells, cytoplasmic, calcineurin-dependent 1, nuclear factor of activated T-cells 1, nuclear factor of activated T cells, cytoplasmic 1, nuclear factor of activated T-cells, cytoplasmic, calcineurin-dependent 1, Nuclear Factors of Activated T-cells, NFATc-1 |
| SGCE | ENSP00000398930 | Sgce, epsilon-sarcoglycan, epsilon-SG, DYT11, epsilon -sarcoglycan, epsilon sarcoglycan, Sarcoglycan epsilon, DYT-11, sarcoglycan-epsilon, DYT 11, CCDS5637.1, CCDS47642.1 |
| PTPRJ | ENSP00000400010 | Scc1, CD148, DEP-1, HPTP eta, PTPRJ, HPTP-eta, r-PTPeta, HPTPeta, protein tyrosine phosphatase receptor type J, SCC-1, Density-enhanced phosphatase-1, SCC 1, density-enhanced phosphatase 1, protein-tyrosine phosphatase receptor type J, density enhanced phosphatase-1, DEP1 |
| FOXA2 | ENSP00000400341 | Foxa2, Foxa-2, Forkhead box a2, Hepatocyte nuclear factor 3beta, HNF3B, HNF-3B, hepatocyte nuclear factor-3beta, Hepatocyte nuclear factor 3 beta, Forkhead box protein A2, hepatocyte nuclear factor 3-beta, hFOXA2, fork head box protein A2, forkhead-box A2 |
| BBC3 | ENSP00000404503 | p53 upregulated modulator of apoptosis, bbc3, p53 up-regulated modulator of apoptosis, p53-upregulated modulator of apoptosis, Bcl-2 binding component 3, Bcl2 binding component 3, p53-up-regulated modulator of apoptosis, p53-upregulated-modulator-of-apoptosis, p53-upregulated-modulator of apoptosis, p53 up regulated modulator of apoptosis, BCL2-binding component 3 |
| RASGRF1 | ENSP00000405963 | GRF1, cdc25, Rasgrf1, guanine nucleotide-releasing protein, GRF-1, cdc-25, guanine nucleotide releasing protein, GNRP, HCDC25L, H-GRF55, Ras-GRF1, Rasgrf-1, Ras protein-specific guanine nucleotide-releasing factor 1, Ras protein-specific guanine nucleotide releasing factor 1 |
| TOP2A | ENSP00000411532 | TOP2A, TOP2, DNA topoisomerase IIalpha, DNA topoisomerase 2alpha, DNA topoisomerase II alpha, DNA topoisomerase II-alpha, TOP2A p, TOP2-A, DNA topoisomerase 2-alpha, TOP-2, DNA topoisomerase IIa, TOP-2a |
| ST6GALNAC5 | ENSP00000417583 | ST6GalNAc V, ST6GALNAC5, GD1alpha synthase, SIAT7E, ST6GalNAcV, sialyltransferase 7e |

| | | |
|---|---|---|
| PARK7 | ENSP00000418770 | DJ-1, PARK7, DJ1, protein DJ-1, 2 or -3, hDJ-1, Hdj1, oncogene DJ-1, PARK-7, oncogene DJ1, protein DJ1, Parkinson protein 7, parkinsonism associated deglycase, PARK 7, parkinsonism-associated deglycase, Parkinson disease protein 7, DJ -1, Parkinson disease-associated genes |
| AADAT | ENSP00000423190 | AADAT, alpha-aminoadipate aminotransferase, KAT II, kynurenine aminotransferase II, KAT2, hKAT2, KATII, kynurenine-aminotransferase II, kynurenine amino transferase II |
| PIK3R1 | ENSP00000428056 | p85, p85alpha, p85 alpha, GRB-1, PIK3R1, 5 ITD, phosphoinositide-3-kinase,  regulatory subunit 1 (alpha, phosphoinositide-3-kinase regulatory subunit 1, GRb1, phosphoinositide-3 kinase regulatory subunit 1, phosphoinositide-3-kinase,  regulatory subunit 1, phosphoinositide-3-kinase regulatory subunit alpha, G-Rb1 |
| SP7 | ENSP00000443827 | Osx, Osterix, Sp7, HSP7, Sp transcription factors, Sp-transcription factors, zinc finger protein Osterix, Sp7 transcription factor, transcription factor sp7 |
| LIMS1 | ENSP00000446121 | PINCH, PINCH-1, PINCH1, senescent cell antigen, LIMS1, LIM and senescent cell antigen-like-containing domain protein 1, Senescent-cell antigen |
| DUX4 | ENSP00000458065 | DUX4, Double homeobox 4, DUX-4, double homeobox protein 4, DUX4L, DUX4p |
| GDF10 | ENSP00000464145 | BMP-3b, GDF-10, Bip, bone morphogenetic protein-3B, Growth/differentiation factor-10, Gdf10, BMP3B, Bone morphogenetic protein 3B, Growth differentiation factor-10, growth differentiation factor 10 |
| ACP5 | ENSP00000468767 | tartrate-resistant acid phosphatase, ACP5, Acp 5, TR-AP, human purple acid phosphatase, Type 5 acid phosphatase, TRACP 5b, TRACP-5b, TRACP5b, tartrate-resistant acid phosphatase type 5, tartrate resistant acid phosphatase, EC 3.1.3.2, hPAP, TRACP 5a, tartrate-resistant acid phosphatase type-5, acid phosphatase 5, tartrate resistant, TRACP5a |
| NTF4 | ENSP00000469455 | NT4, NT-4, NTF4, neurotrophin-4, NT4/5, neurotrophin-5, NT-4/5, NT-5, NT5, neurotrophin 4, NTF5, Ntf-5, NT-4 / 5, NT 4/5 |
| COMMD3-BMI1 | ENSP00000473391 | Bmi1, Bmi-1, PCGF4, polycomb complex protein Bmi1, Polycomb complex protein BMI-1, COMMD3-BMI1 |

| | | |
|---|---|---|
| DUOX2 | ENSP00000475084 | nicotinamide adenine dinucleotide phosphate oxidase, THOX2, DUOX2, LNOX2, thyroid oxidase 2, p138(Tox, nicotinamide adenine dinucleotide phosphate-oxidase, Dual oxidase 2, nicotin-amide adenine dinucleotide phosphate-oxidase, dual-oxidase 2, Dual Oxidase-2, Nicotinamide-Adenine Dinucleotide Phosphate Oxidase |
| NOS1 | ENSP00000477999 | nNOS, NOS1, nitric oxide synthase 1, Neuronal NOS, bNOS, constitutive NOS, NOS-1, Nos 1, NOS1-P, nitric oxide synthase-1, n-NOS, B-NOS |
| RPS10 | ENSP00000481646 | RPS10, Hs10, ribosomal protein S10, HS-10, S10-P |
| CCL4 | ENSP00000482259 | SCYA4, MIP-1beta, HC21, CCL4, LAG-1, Lag1, MIP1-beta, Scya2, MIP-1 beta, HLA-G1, macrophage inflammatory protein-1 beta, ACT-2, Mip-1b, AT744.1, macrophage inflammatory protein 1 beta, macrophage inflammatory protein-1beta, Lag1p, MIP1beta, macrophage inflammatory protein 1beta, macrophage-inflammatory protein-1beta, MIP 1beta, macrophage inflammatory protein-1-beta, small inducible cytokine A4, MIP1 beta, HLAG1, CCL-4, MIP1B, chemokine ligand 4, ACT2, chemokine (C-C motif) ligand 4, C-C motif chemokine ligand 4, HLAG-1 |
| NR2C2 | ENSP00000483059 | TAK1, HTR4, TR4, hTAK1, NR2C2, TAK-1, orphan nuclear receptor TR4, nuclear receptor subfamily 2, group C, member 2, testicular receptor 4, Testicular Receptor-4, TAK 1, nuclear receptor subfamily 2 group C member 2 |
| HOTAIR | HOTAIR | HOTAIR, HOTAIR 5, HOTAIR 3, HOTAIR3, HOTAIR5, HO-TAIR |
| MIR146B5P | hsa-miR-146b-5p | miR-146b, miR-146b5p, MiR-146b-5p, hsa-miR-146b, miR146b-5p, miR146b, hsa-miR-146b-5p, hsa-miR146b-5p |
| MIR7-3HG | MIR7-3HG | HuH7, HUH-7, HuH 7, uc002mbe.2, MIR7-3HG |
| PWAR4 | PWAR4 | PAR4, par-4, Par 4 |
| CYGB | ENSP00000293230 | cytoglobin, Hgb, Cygb, stellate cell activation-associated protein, STAP, HG-B |
| FGF19 | ENSP00000294312 | FGF19, 2----p23, FGF-19, fibroblast growth factor 19, Fibroblast growth factor-19, 2 p23, fibroblast growth factor19 |
| RAD21 | ENSP00000297338 | SCC1, hRad21, RAD21, Rad21p, kleisin, Scc1p, Mcd1p, hHR21, MCD1, rad 21, SCC-1, SCC 1, RAD21 homolog (S. pombe, RAD21 p, RAD21 cohesin complex component |

| | | |
|---|---|---|
| ALDH1A1 | ENSP00000297785 | HEL 12, ALDH1, aldehyde dehydrogenases, ALDH1A1, retinaldehyde dehydrogenase 1, Raldh1, AldC, aldehyde dehydrogenase 1 family member A1, aldehyde dehydrogenase 1 family-member A1, retinal dehydrogenase 1, ALD-C, ALDH-1, ALDH 1A1, retinal dehydrogenase-1, raldh-1, aldehyde dehydrogenase family 1 member A1, Aldh1a 1 |
| WRN | ENSP00000298139 | WRN, RECQL2, RecQ3, RECQL3, Werner syndrome ATP-dependent helicase, Werner syndrome RecQ like helicase, RECQ-like helicases |
| DEPP1 | ENSP00000298295 | decidual protein induced by progesterone, C10orf10, DEPP |
| TRIM68 | ENSP00000300747 | GC109, TRIM68, SS-56 |
| CYP2A6 | ENSP00000301141 | CYP2A6, CPA6, CYP2A, cytochrome P4502A6, coumarin 7-hydroxylase, Cytochrome P450 2A6, CYP2A3, P-450PB, CYP 2A, P450PB, CYP 2A6, cytochrome P450IIA3, cytochrome P450, family 2, subfamily A, polypeptide 6, cytochrome P450 family 2 subfamily A member 6 |
| PELP1 | ENSP00000301396 | PELP1, p160, Hmx3, MNAR, modulator of nongenomic activity of estrogen receptor, transcription factor Hmx3, Proline-, glutamic acid- and leucine-rich protein 1, proline-, glutamic acid- and leucine-rich-protein-1, proline, glutamate and leucine rich protein 1, PELP-1, Proline-, glutamic acid- and leucine-rich protein-1, modulator of non-genomic activity of estrogen receptor, proline, glutamic acid and leucine rich protein 1, proline-, glutamate- and leucine-rich protein 1 |
| CD14 | ENSP00000304236 | CD14, CD1 4, myeloid cell-specific leucine-rich glycoprotein, CD14 molecule, Monocyte differentiation antigen CD14, CD14 antigen, CD-14, CD 14, hCD14 |
| ING2 | ENSP00000307183 | ING1L, p32, p33ING2, ING2, hp32, inhibitor of growth family member 2, inhibitor of growth family, member 2, P3 2, inhibitor of growth protein 2 |
| KRT17 | ENSP00000308452 | PC-2, PC2, HPC2, CK 17, keratin 17, Krt17, CK17, cytokeratin 17, Pc 2, keratin-17, Ck-17, Hpc-2, cytokeratin 17, KRT 17, Keratin17 |
| TNKS | ENSP00000311579 | tankyrase, tankyrase 1, tankyrase-1, TNKS, poly(ADP-ribose) polymerases, TIN1, EC 2.4.2.30, poly (ADP-ribose) polymerases, TNKS-1, TNKS1, poly (ADP ribose) polymerases, tankyrase, TRF1-interacting ankyrin-related ADP-ribose polymerase, PARP5A, tankyrase1, ARTD-5 |

| | | |
|---|---|---|
| HNF4A | ENSP00000312987 | HNF4alpha, HNF-4alpha, HNF4, TCF, HNF-4, hepatocyte nuclear factor 4alpha, hepatocyte nuclear factor 4 alpha, NR2A1, Hnf4a, hHNF-4, HNF-4 alpha, hepatocyte nuclear factor-4 alpha, hepatocyte nuclear factor-4alpha, hHNF-4A, transcription factor HNF-4, TCF14, HNF4 alpha, HNF- 4 alpha, hepatocyte nuclear factor 4-alpha, hepatocyte nuclear factor4alpha, HNF 4 alpha, HNF-4A, HNF 4alpha, hepatocyte nuclear factor 4A, HNF4 A, hepatocyte nuclear factor-4a, HNF4-alpha, HNF4A p, Hepatocyte Nuclear Factor 4, Alpha, HNF4A Human, transcription factor 14, HNF4-A, transcription factor HNF4 |
| AURKB | ENSP00000313950 | Ipl1, Aim-1, Aurora B, Aurora-B, Aurora kinase B, AIM1, ARK2, STK12, AIK2, Ipl1p, Ark-2, STK1, STK-1, AURKB, Aurora kinase-B, AURKB-Sv2, AuroraB, S-TK1, AURK-B |
| DUOX1 | ENSP00000317997 | ThOX1, nicotinamide adenine dinucleotide phosphate oxidase, nicotin-amide adenine dinucleotide phosphate-oxidase, Duox1, DUOX, nicotinamide adenine dinucleotide phosphate-oxidase, LNOX1, dual oxidase 1, dual oxidase-1, Nicotinamide-Adenine Dinucleotide Phosphate Oxidase, dual oxidase1 |
| GEN1 | ENSP00000318977 | GEN1, Gen-1, Flap endonuclease GEN homolog 1 |
| CALR | ENSP00000320866 | calreticulin, cC1q-R, crt, Calr, CRTC, CR-t, cC1qR, CR T |
| PTH1R | ENSP00000321999 | PTHR, PTH1R, PTHR1, parathyroid hormone/parathyroid hormone-related peptide receptor, parathyroid hormone receptor-1, PTH/PTHrP type I receptor, parathyroid hormone 1 receptor, 1BL1, P-thr, PTH-1R, PTH-R, Parathyroid Hormone Receptor 1, parathyroid hormone-1 receptor, PTH-1-R, hPTHR1, parathyroid hormone/ parathyroid hormone-related peptide receptor |
| LMNB2 | ENSP00000327054 | lamins, LAMB2, LMNB2, lamin B2, Lamb-2, lamin B 2, HLA-MB2 |
| PAWR | ENSP00000328088 | Par-4, prostate apoptosis response-4, PAR4, prostate-apoptosis-response-4, PAWR, prostate apoptosis response-4 protein, prostate apoptosis response 4, PRKC, apoptosis, WT1, regulator, prostate apoptosis response 4 protein, Par 4 |
| WT1 | ENSP00000331327 | Wt1, AWT1, WT33, WT-1, Wilms tumor 1, EWS-WT1, EWS WT1, W T1, WT-33, Wilms tumor-1, EWS-WT 1, Wilms-Tumor 1, NPHS4, WT1 p, Wilms Tumor Protein, WT1 human, wt 1p |

| | | |
|---|---|---|
| MEF2C | ENSP00000340874 | MEF2C, MEF-2C, myocyte enhancer factor 2C, hMEF2C, myocyte enhancer factor-2C, myocyte-specific enhancer factor 2C, Myocyte enhancer factor 2 C, Myocyte-enhancer factor 2C |
| PTPN11 | ENSP00000340944 | Shp2, SHP-2, PTPN11, PTP2C, SH-PTP2, protein tyrosine phosphatase,  nonreceptor type 11, protein-tyrosine phosphatase, non-receptor-type 11, SH-PTP3, protein-tyrosine phosphatase-1D, BPTP3, Protein-tyrosine phosphatase,  nonreceptor type 11, protein-tyrosine phosphatase,  nonreceptor-type 11, PTP-1D, PTP-2C, SHPTP2, PTPN 11, protein tyrosine phosphatase,  non-receptor type 11, protein tyrosine phosphatase,  non-receptor-type 11, protein tyrosine phosphatase,  nonreceptor-type 11, CF C, PTPN11 p, tyrosine-protein phosphatase non-receptor type 11, 5 IBM, protein-tyrosine phosphatase,  non-receptor type 11, PTPN-11, hShp2, HNS1, cfc |
| TNNI3 | ENSP00000341838 | cardiac troponin I, TNNI3, cTnI, TNNC1, Cardiac troponin-I, 1OZS, c Tn-I, cTn-I, TNNI3-p, cTn I, TNNI3 p, Cardiac-Troponin-I |
| CYP1A2 | ENSP00000342007 | CYP1A2, cytochrome P450 1A2, cp12, P3-450, CYP 1A2, EC 1.14.14.1, cholesterol-25-hydroxylase, cytochrome P450 family 1, cholesterol 25-hydroxylase, cytochrome P4501A2, cytochrome P(3)450, CYPIA2, Cytochrome P-4501A2, Cholesterol 25 hydroxylase, cytochrome P450-1A2, cytochrome P-450 1A2, 2HI4, Cytochrome P450 Family 1 Subfamily A Member 2, Cytochrome P450 4, Cyp1a-2, CYP IA2 |
| CDH5 | ENSP00000344115 | vascular endothelial cadherin, VE-Cadherin, CDH5, vascular endothelial-cadherin, CD144, cadherin-5, vascular-endothelial-cadherin, cadherin 5, VEcadherin, VE cadherin, cadherin5 |
| PDPK1 | ENSP00000344220 | 3-phosphoinositide-dependent protein kinase 1, PDK1, PDK-1, PKB kinase, 3-phosphoinositide-dependent protein kinase-1, 3 ion, PDPK1, PDPK-1, 3-phosphoinositide dependent protein kinase-1, PDPK2P |

| | | |
|---|---|---|
| HAVCR1 | ENSP00000344844 | TIM-1, TIM, TIM1, HAVcr-1, Kidney injury molecule-1, hepatitis A virus cellular receptor 1, KIM-1, HAVCR1, hHAVcr-1, Ki-M1P, Kim 1, kidney injury molecule 1, HAVCR, hKIM-1, KIM1, T cell immunoglobulin and mucin domain-containing protein 1, Kidney-injury-molecule-1, T-cell immunoglobulin and mucin domain-containing protein 1, CD365, kidney injury molecule - 1, T-cell immunoglobulin mucin receptor 1, Kidney-injury-molecule 1, Ki-M1 |
| ANXA6 | ENSP00000346550 | p70, annexin VI, p68, ANX6, annexin A6, annexins, 67-kDa calelectrin, HP68, AnxA6, protein III, annexin-VI |
| HNRNPA2B1 | ENSP00000346694 | hnRNP A2, hnRNPA2B1, hnRNPA2, HNRPA2B1, hnRNP B1, heterogeneous nuclear ribonucleoprotein A2/B1, heterogeneous nuclear ribonucleoprotein-A2/B1, hnRNP-B1, hnRNP-A2, heterogeneous nuclear ribonucleoproteins A2/B1, hnRNPB1, hnRNP A2B1, hnRNP A2 B1 |
| RAC1 | ENSP00000348461 | Rac1, Rho family GTPases, Rac-1, Ras-related C3 botulinum toxin substrate 1, Rho-family GTPases, ras-related C3 botulinum toxin substrate-1, Rac 1, p21Rac1, Ras-related C3 botulinum toxin substrate 1, h_rac1, Ras-related C3 botulinum toxin substrate 1 (Rho family, small GTP-binding protein Rac1, Rac family small GTPase 1, Ras related C3 botulinum toxin substrate1, p21-rac1 |
| GIP | ENSP00000350005 | gastric inhibitory polypeptide, glucose-dependent insulinotropic polypeptide, incretin hormone, gastric-inhibitory polypeptide, Glucose dependent insulinotropic polypeptide |
| CX3CR1 | ENSP00000351059 | CCRL1, CX3CR1, fractalkine receptor, CMKBRL1, CX3C chemokine receptor 1, CMK-BRL1, chemokine receptor 1, frac-talkine receptor, chemokine (C-X3-C motif) receptor 1, chemokine receptor-1, CX3C-chemokine receptor 1, hCX3CR1, C-X3-C motif chemokine receptor 1 |
| FCGR2B | ENSP00000351497 | FCGR2B, FcgammaRIIb, FCGR2, FCG2, Fcgamma receptor IIB, CDw32, CD32, Fc gamma RIIb, IGF-R2, CD32B, Fc gamma receptor IIb, Fc-gamma receptor IIB, IGFR2, IGFR-2, Fc fragment of IgG receptor IIb, Fc-gamma receptor-IIb |
| DIO1 | ENSP00000354643 | DIO-1, selenoprotein S, dio1, hdio1, Type I iodothyronine deiodinase, type-I iodothyronine deiodinase, type I 5'-deiodinase, iodothyronine deiodinase 1, selenoprotein-S |
| MTR | ENSP00000355536 | MTR, methionine synthase, cblG, EC 2.1.1.13, 5-methyltetrahydrofolate-homocysteine methyltransferase, MTR-201, MTR-001 |

| | | |
|---|---|---|
| CFH | ENSP00000356399 | CFH, complement factor H, LRG-47, FHL1, beta 1H, FHL 1, FHL-1, 1 HCC, hUS, AMBP-1, HF-2, CFHL3, complement factor-H, 4 on T, beta1H, complement-factor H, Complement Factor- H, Hf2 |
| HBS1L | ENSP00000356811 | HBS1L, Hbs1, eRFS, Hbs1p, EF1A, EF-1A |
| PEA15 | ENSP00000357055 | PEA-15, PEA15, MAT1, ped/pea-15, HMAT1, Phosphoprotein Enriched in Astrocytes-15, astrocytic phosphoprotein PEA-15, phosphoprotein enriched in diabetes, death effector domain containing, PED/ PEA-15, phosphoprotein enriched in astrocytes 15, Mat 1, PED/PEA15, phosphoprotein enriched in astrocytes15 |
| ACKR1 | ENSP00000357103 | DARC, Duffy-blood-group, gpD, FY glycoprotein, Gp-Fy, gpFy, GP D, glycoprotein D, Duffy antigen/chemokine receptor, G lycoprotein D, atypical chemokine receptors, ACKR1, Atypical Chemokine Receptor 1, DARC/ACKR1, glycoprotein-D |
| FCGR1A | ENSP00000358165 | FcgammaRIa, Fc gamma RI, FCGR1A, FcgammaRI, FCGR1, CD64, Fcgamma RIA, Fc gammaRI, IGFR1, Fcg1, IGF-R1, IGFR-1, IGF-R-1, FcgammaR I, hCD64, high affinity immunoglobulin gamma Fc-receptor I, CD 64, FCRI, CD64A, CD64 - A |
| MAP3K7 | ENSP00000358335 | TAK1, Map3k7, hTAK1, TGF-beta-activated kinase 1, TGF-beta activated kinase 1, TGFbeta-activated kinase 1, TGF-beta-activated kinase-1, TAK-1, TAK 1, mitogen-activated protein kinase kinase kinase 7, MAP 3K7, MEKK7 |
| GSTA4 | ENSP00000360002 | GST A4-4, hGSTA4, Gsta4, glutathione S-transferase A4-4, Glutathione-S-transferase A4-4, GSTA4-4, glutathione S-transferase alpha 4, glutathione S-transferase A4, GST-A4, Glutathione-s-transferase A 4, glutathione S transferase-A4 |
| CPT2 | ENSP00000360541 | carnitine palmitoyltransferase II, CPT2, CPTase, CPT1, Carnitine-palmitoyltransferase 2, hCPT1, carnitine palmitoyl transferase II, carnitine palmitoyl transferase 2, carnitine palmitoyltransferase 2, EC 2.3.1.21, carnitine palmitoyltransferase-2, CPT 2, Carnitine palmitoyl-transferase II, CPT-2, Carnitine palmitoyltransferase-II, CPT-1, carnitine-palmitoyl-transferase II, carnitine-palmitoyl-transferase-II, carnitine palmitoyl transferase-II |
| ADA | ENSP00000361965 | adenosine deaminase, ADA1, ADA-1, adenosine aminohydrolase, EC 3.5.4.4, adenosinedeaminase, ADA 1, adenosine-deaminase, Ad-A, ADA human, ada, adenosine deaminase, ADA- human, EC3.5.4.4 |

| | | |
|---|---|---|
| TTPAL | ENSP00000361995 | C20ORF121, TTPAL |
| SGPL1 | ENSP00000362298 | SGPL1, Spl, S1PL, sphingosine-1-phosphate lyase 1, NPHS14, sphingosine-1-phosphate lyase-1 |
| CHD6 | ENSP00000362330 | CHD5, CHD6, chromodomain helicase DNA binding protein 6 |
| PGK1 | ENSP00000362413 | PGK1, Pgk-1, phosphoglycerate kinase 1, hPgk-1, Mig-10, phosphoglycerate kinase-1, MIG10, PGK 1, phosphoglyceratekinase-1 |
| ANGPTL2 | ENSP00000362524 | ARP2, HARP, Angptl2, ANGPTL-2, Angiopoietin-like, angiopoietin-related protein-2, angiopoietin-like 2, angiopoietin-related protein 2, angiopoietin-like protein 2, HARP2, Angiopoietin Like Protein 2, angiopoietin-like protein-2, Angiopoietin-Like-2, Angiopoietin like-2, Angiopoietin Like, Angptl 2 |
| TNFSF12 | ENSP00000293825 | TWEAK, tumor necrosis factor superfamily, Apo3L, Apo3 ligand, tumor-necrosis factor superfamily, TNFSF12, Apo3-L, TNF-related weak inducer of apoptosis, tumor necrosis factor super-family, tumor necrosis factor superfamily, member 12, tumor necrosis factor-superfamily, TNFSF-12, tumor necrosis factor (ligand) superfamily, member 12, TNF superfamily member 12, tumor necrosis factor super family |
| CXCR1 | ENSP00000295683 | IL8RA, CXCR1, CC CKR1, IL8R1, CC CKR-1, C-C CKR-1, IL-8R1, IL-8RA, CC-CKR1, CXCR-1, chemokine receptor 1, CKR-1, chemokine receptor-1, C-X-C motif chemokine receptors, CXC-chemokine receptor type1, Chemokine (C-X-C motif) receptor 1, C-X-C motif chemokine receptor 1 |
| GRIA2 | ENSP00000296526 | GluR-B, GluR2, GluA2, HBGR2, glutamate receptor 2, GluR B, GluRB, Gria2, glutamate ionotropic receptor AMPA type subunit 2, glutamate ionotropic receptor AMPA-type subunit 2 |
| SHH | ENSP00000297261 | Shh, Sonic Hedgehog, Tpt, Sonic hedgehog protein, Sonic Hedge Hog, Sonic Hedgehog homolog, Sonic-Hedgehog, HHG1, Shh human, TP-T |
| TSC1 | ENSP00000298552 | TSC1, hamartin, TSC, TSC complex, TSC-1, TSC 1, hTSC1, TS-C1, TS-C1, TSC1 p, lam |
| BIRC5 | ENSP00000301633 | EPR-1, Birc5, BIRC-5, EPR1, baculoviral IAP repeat-containing 5, Baculoviral IAP repeat containing 5 |

| | | |
|---|---|---|
| CYP7A1 | ENSP00000301645 | Cholesterol 7alpha-hydroxylase, CYP7A1, CYP7, cholesterol 7 alpha-hydroxylase, cholesterol 7alpha hydroxylase, cholesterol 7-alpha-hydroxylase, cytochrome P450 7A1, hCYP7A1, cholesterol-7-alpha-hydroxylase, Cholesterol 7-alpha hydroxylase, cytochrome P450, family 7, subfamily a, polypeptide 1, CYP 7A1 |
| CCDC8 | ENSP00000303158 | p90, CCDC8, coiled-coil domain containing 8, Coiled-coil domain-containing protein 8 |
| PEX6 | ENSP00000303511 | peroxisome assembly factor-2, Pex6, Pxaaa1p, PAF-2, peroxins, Pex6p, PXAAA1, peroxisomal biogenesis factor 6, HPAF2, PEX6 p |
| CDC25A | ENSP00000303706 | Cdc25A, cell division cycle 25A, dual-specificity phosphatase Cdc25A, CDC25 A, CDC 25A, Cell division cycle 25 A, CDC25-A, dual specificity phosphatase Cdc25A |
| SCG2 | ENSP00000304133 | secretogranin II, Secretoneurin, granins, chromogranin C, SCG2, SGII, EM66, SCG-2, secretogranin-2, secretogranin-II, SCG 2 |
| ADORA2B | ENSP00000304501 | adenosine receptors, adenosine A2b receptor, ADORA2B, ADORA2, adenosine receptor A2b, adenosine A2B-receptor |
| ISG20 | ENSP00000306565 | CD25, ISG20, HEM45, hCD25, interferon-stimulated gene 20 kDa protein, ISG-20, CD-25, interferon-stimulated exonuclease gene 20, CD 25 |
| NPTX1 | ENSP00000307549 | NP1, Np-1, Neuronal pentraxin 1, HNP-1, HNP1, NPTX1, HNP 1, neuronal pentraxin I, NPTX-1 |
| F2 | ENSP00000308541 | prothrombin, EC 3.4.21.5, coagulation factor II, 5 to -3, 1 ppb, HF-2, 4rKO, 1 THP, 1Thr, coagulation factor II (thrombin, pro-thrombin, 1 nt 1, 1-NT1, Hf2, 2 Fe-S, 1-NRP, coagulation factor II, thrombin, 2 cN0, 1 to m, 1-KTS |
| RHOD | ENSP00000308576 | Rho, Rho family GTPases, Rho-family GTPases, rhoHP1, RhoD, Rho-related protein HP1, ARHD |
| OLR1 | ENSP00000309124 | LOX-1, OLR1, lectin-like oxidized LDL receptor-1, oxidized low-density lipoprotein receptor 1, LOX1, oxidized low-density-lipoprotein receptor-1, oxidized low density lipoprotein receptor-1, lectin-like oxidized LDL receptor 1, oxidized low-density lipoprotein receptor-1, LOXIN, sLOX-1, oxidised low-density lipoprotein receptor 1, OLR-1, oxidized low-density lipoprotein-receptor 1, lectinlike oxidized LDL receptor-1, lectin-type oxidized LDL receptor 1, oxidized low density lipoprotein receptor 1, lectin-type oxidized LDL receptor-1, sLOX -1, LOX -1, Oxidized low-density lipoprotein (lectin-like) receptor 1, LOX_1 |

| | | |
|---|---|---|
| XCR1 | ENSP00000310405 | CCXCR1, GPR5, GPR-5, chemokine receptor 1, lymphotactin receptor, XCR1, XC chemokine receptor 1, G-protein coupled receptor 5, G protein-coupled receptor 5, G-protein-coupled receptor 5, G-protein coupled receptor5, chemokine receptor-1, X-C motif chemokine receptor 1, G Protein-coupled Receptor-5 |
| CTSF | ENSP00000310832 | cathepsin F, cathepsin S, cathepsins, CTSF, cathepsin-F, cathepsin-S |
| CTSW | ENSP00000311300 | cathepsin S, cathepsins, cathepsin W, lymphopain, cathepsin-S |
| IQCB1 | ENSP00000311505 | IQCB1, nephrocystin-5, NPHP5, p53 and DNA damage-regulated IQ motif protein, IQ calmodulin-binding motif-containing protein-1 |
| PLA2G1B | ENSP00000312286 | phospholipase A2, PlA2, phospholipase A-2, PLA2G1B, phospholipase-A2, phospholipaseA2, PLA2A, PPLA2 |
| PANK2 | ENSP00000313377 | PANK2, harp, hPANK2, pantothenate kinase 2, EC 2.7.1.33, pantothenate kinase-2, pantothenate-kinase 2, PANK-2 |
| GAK | ENSP00000314499 | cyclin-G associated kinase, GAK, Cyclin G-associated kinase, Cyclin-G-associated kinase, cyclin G associated kinase, DNAJC26 |
| TYMS | ENSP00000315644 | thymidylate synthase, TYMS, EC 2.1.1.45, thymidylate synthetase, 5 x 4 x, T MS |
| STAT2 | ENSP00000315768 | STAT2, ISGF3, ISGF-3, p113, Signal transducer and activator of transcription 2, P113P, Stat-2, signal transducer and activator of transcription-2, hSTAT2 |
| HSD11B2 | ENSP00000316786 | HSD11B2, 11beta-hydroxysteroid dehydrogenase type 2, 11 beta-hydroxysteroid dehydrogenase type II, HSD11K, 11 beta-hydroxysteroid dehydrogenase type 2, 11Beta-hydroxysteroid dehydrogenase type II, HSD2, HSD 11B2, 11betahydroxysteroid dehydrogenase type 2, HSD11 B2, 11beta-hydroxysteroid dehydrogenase type-2, hydroxysteroid dehydrogenase2, 11-beta hydroxysteroid dehydrogenase type 2, 11-beta-hydroxysteroid dehydrogenase type 2, hydroxysteroid dehydrogenase 2, HSD-2, hydroxysteroid (11-beta) dehydrogenase 2, AME-1, 11 beta-hydroxysteroid dehydrogenase type-2, hydroxysteroid dehydrogenase-2, HSD 2 |
| NEUROG1 | ENSP00000317580 | neurogenin1, ngn1, neuroD3, neurogenin-1, Neurogenin 1, ngn, NEUROG1 |
| TPO | ENSP00000318820 | thyroid peroxidase, T-P-X, Tpo, T PO, MS-A, thyroid-peroxidase, Tpx |

| | | |
|---|---|---|
| HCLS1 | ENSP00000320176 | p75, HP75, HCLS1, hematopoietic cell-specific Lyn substrate 1, HP-75, hematopoietic cell-specific Lyn substrate-1, hematopoietic cell specific Lyn substrate-1, hematopoietic-lineage-cell-specific protein, Hematopoietic lineage cell-specific protein |
| ETV4 | ENSP00000321835 | E1AF, PEA3, E1A-F, adenovirus E1A enhancer binding protein, E1A enhancer binding protein, ETV4, adenovirus E1A enhancer-binding protein, PEA-3, ets variant gene 4, ETV-4, ets variant 4, ETS translocation variant 4 |
| SIGLEC7 | ENSP00000323328 | siglec-7, p75, p75/AIRM1, adhesion inhibitory receptor molecule 1, Siglec7, HP75, adhesion inhibitory receptor molecule-1, HP-75, sialic acid-binding Ig-like lectin 7 |
| METAP2 | ENSP00000325312 | p67, aminopeptidases, MAP-2, MetAP2, MAP2, MAP 2, methionine aminopeptidase 2, Methionine aminopeptidase-2, MetAP-2, amino peptidases, amino-peptidases, MAP - 2 |
| CYP11B2 | ENSP00000325822 | CYP11B2, aldosterone synthase, steroid 18-hydroxylase, CYP11B, P-450C18, P-450aldo, cytochrome P-450aldo, CPN2, P450C18, aldosterone-synthase, Cpn-2, P450aldo, hCyp11B2, CYP11-B2, cytochrome P450 family 11 subfamily B member 2 |
| NOG | ENSP00000328181 | Nog, noggin, SYM1, SYNS1 |
| EPHB3 | ENSP00000332118 | EphB3, hek2, HEK 2, EK2, EK-2, EPH-B3, Ephrin type-B receptor 3 |
| PTP4A3 | ENSP00000332274 | PRL-3, PRLR, phosphatase of regenerating liver-3, PRL-R, PRL3, PTP4A3, Phosphatase of regenerating liver 3, protein-tyrosine phosphatase 4A3, Protein tyrosine phosphatase 4A3, protein-tyrosine phosphatase of regenerating liver 3, Phosphatase of Regenerating Liver3, Protein tyrosine phosphatase of regenerating liver 3, protein tyrosine phosphatase type IVA,  member 3, 2 MBC |
| SERPINA5 | ENSP00000333203 | protein C inhibitor, plasma serine protease inhibitor, plasminogen activator inhibitor-3, 1 PAI, pCI, Serpina 5, PAI-3, SERPINA5, Serpin A5, serpin-A5, ProteinC inhibitor, Plasminogen Activator Inhibitor 3, PC-I, 2 PAI |
| LAMP1 | ENSP00000333298 | LAMP1, CD107a, lamp-1, h-lamp-1, Lamp 1, lysosomal associated membrane protein-1, Lysosomal-associated membrane protein-1, lysosomal-associated membrane protein 1, CD 107a, lysosomal associated membrane protein 1, Lysosome associated membrane glycoprotein 1 |

| | | |
|---|---|---|
| TTF1 | ENSP00000333920 | TTF-1, TTF-I, TTF1, Transcription Termination Factor 1, transcription termination factor, RNA polymerase I, transcription termination factor-1 |
| HBB | ENSP00000333994 | Hb D, Hbb, 3D17, HBD, hemoglobin beta-chain, beta-Globin, hemoglobin beta chain, beta -globin, betaGlobin, hemoglobin subunit beta, hemoglobin subunits, Beta globin, 1-J40, 1 CBL, 1BB-B |
| ADORA2A | ENSP00000336630 | adenosine receptors, RDC8, adenosine A2a receptor, ADORA2, ADORA2A, adenosine receptor A2A, 1 mm H, adenosine A2A-receptor, adenosine-A2A receptor |
| EDN3 | ENSP00000337128 | ET-3, ET3, EDN3, endothelin-3, endothelin 3, Endothelin3, EDN-3, endothelin- 3, preproendothelin-3, PPET-3, EDN 3 |
| SLC22A8 | ENSP00000337335 | hOAT3, OAT3, SLC22A8, organic anion transporter 3, organic anion transporter-3, Oat 3 |
| FOXG1 | ENSP00000339004 | Foxg1, FOXG1B, Brain factor-1, HFK2, brain factor 1, Hb F1, BF-1, HFK3, FOXG1A, HBF-2, HFK1, FKH2, BF1, FOXG1C, forkhead box G1, HBF-1, fork-head box protein G1, HbF 2, forkhead box G1B, forkhead box protein G1, forkhead-box protein G1, FOXG1 c |
| RHD | ENSP00000339577 | RH30, RhD, Rh D, Rhced, RhXIII, Rh30A, Rh-D, HRH4, Rh4 |
| TRIM24 | ENSP00000340507 | TIF1, Tif1a, Tif-1alpha, TIF1alpha, TRIM24, transcriptional intermediary factor 1, TIF1 alpha, HTIF1, PTC6, htif 1, tripartite motif-containing 24, transcription intermediary factor 1alpha, TIF-1, transcription intermediary factor 1-alpha, tripartite motif containing 24, TF-1a, Tripartite motif-containing protein 24, transcriptional intermediary factor-1 |
| MIA3 | ENSP00000340900 | ARNT, TANGO, TANGO1, C219-reactive peptide, MIA3, MIA-3 |
| NEO1 | ENSP00000341198 | neogenin, NEO1, NGN, Neogenin-1, Neogenin1 |
| NANOS3 | ENSP00000341992 | NOS3, nanos3, NOS 3, NOS-3 |
| TNFSF13 | ENSP00000343505 | tumor-necrosis factor superfamily, tumor necrosis factor superfamily, Tnfsf13, tumor necrosis factor super-family, CD256, tumor necrosis factor-superfamily, APRoliferation inducing ligand, a-proliferation-inducing ligand, TNF superfamily member 13, tumor necrosis factor super family |
| CTSL | ENSP00000345344 | Cathepsin L, cathepsin S, 3 of 9, CatL, Cat L, cathepsins, 3 of 8, Mep, CTSL, major excreted protein, cathepsin L1, Cathepsin-L, cathepsin-S, cathepsin L-1, c-ATL |

| | | |
|---|---|---|
| TOR1A | ENSP00000345719 | DYT1, DQ2, TOR1A, torsin A, torsinA, TorA, torsin-A, DYT-1, DYT 1, torsin 1A, TOR1-A, torsin family 1,  member A, DQ 2, Torsin1A |
| ATP7A | ENSP00000345728 | HMC-1, ATP7A, MNK, MC1, ATP7 A, Mc 1, MC-1, HMC1, Copper-transporting ATPase 1, ATPase copper transporting alpha, ATPase copper-transporting alpha |
| ANXA2 | ENSP00000346032 | p36, annexin II, Annexin A2, lipocortin II, PAP-IV, annexins, LIP2, Annexin 2, Anx2, calpactin I heavy chain, annexin-II, Pap IV, Annexin-2, annexin2, AnxA2, p36p, annexin-A2, AnnexinA2, p3-6, lipocortin-II |
| UGT1A9 | ENSP00000346768 | HUGT1, UGT1A9, Gnt-1, UGT1, UDPGT, Hlug P4, UGT-1, UDP glycosyltransferase 1 family,  polypeptide A9, HlugP4, GNT1, UDP-glucuronosyltransferase 1A9 |
| NKX2-1 | ENSP00000346879 | thyroid transcription factor-1, TTF-1, NKX2.1, TTF1, NKX2-1, NK-2, NMTC1, TITF1, thyroid transcription factor 1, TEBP, Thyroid nuclear factor 1, T/ebp, NK2, TITF-1, Nk2 homeobox 1, Nkx 2.1, thyroid-specific enhancer-binding protein, Nkx-2.1, HT-ITF1, thyroid-specific-enhancer-binding protein, NK2 homeobox-1, NKX-2-1, thyroid transcription factor -1 |
| PDZD3 | ENSP00000347742 | intestinal and kidney-enriched PDZ protein, IKEPP, PDZK2, PDZ domain-containing protein 2, PDZ domain-containing protein 3, PDZD3, PDZ domain containing 3 |
| ZNF44 | ENSP00000348419 | ZNF, ZNF44, Kox7, ZNF-44 |
| AMPH | ENSP00000348602 | amphiphysin, 4 atm, AMPH1, AMPH-1 |
| ITGAL | ENSP00000349252 | LFA-1, CD11a, LF-A1, integrin alpha-subunits, ITGAL, integrin alpha subunits, LFA1, integrin alpha L, Itgal-003, CD11-a, integrin alpha-L, integrin subunit alpha L, integrin alphaL |
| SERTAD1 | ENSP00000350633 | TRIP-Br1, SEI-1, SERTAD1, SERTA domain containing 1, SEI1, SERTA domain-containing protein 1 |
| CTSE | ENSP00000350911 | cathepsin S, CTSE, cathepsin E, cathepsins, CatE, cathepsin-S, cathepsin-E |
| PTPN22 | ENSP00000352833 | Lyp, PTPN22, Lyp2, pep, Lyp1, tyrosine-protein phosphatase non-receptor type 22, lymphoid phosphatase, PTPN 22, LyP-1, protein tyrosine phosphatase,  non-receptor type 22, protein tyrosine phosphatase,  non-receptor type 22 (lymphoid, PTPN22.6, PTPN2 2, protein tyrosine phosphatase,  nonreceptor type 22, PTPN-22 |

| | | |
|---|---|---|
| MYLK | ENSP00000353452 | MYLK, MLCK, MLCK 1, KRP, myosin light chain kinase, kinase-related protein, smooth muscle myosin light chain kinase, kinase related protein, telokin, mylk1, SM MLCK, smMLCK, myosin light-chain kinase, SM-MLCK, Smooth muscle myosin light-chain kinase, MLCK210, MLCK1 |
| TOP1 | ENSP00000354522 | topoisomerase I, TOP1, DNA topoisomerase I, topoisomerase-I, Top-1, DNA-topoisomerase I, DNA topoisomerase-1, topoisomerase I, DNA topoisomerase 1, Top 1, Topoisomerase (DNA) I, DNA-Topoisomerase 1 |
| CXCL9 | ENSP00000354901 | Mig, CXCL9, CMK, HuMig, SCYB9, chemokine ligand 9, monokine induced by gamma-interferon, monokine induced by interferon-gamma, monokine induced by interferon gamma, monokine induced by gamma interferon, CXCL-9, MI-G, CXC L9, M IG, chemokine (C-X-C motif) ligand 9, C-X-C motif chemokine 9, chemokine (C-X-C motif) ligand-9, C-X-C motif chemokine ligand 9 |
| POU3F3 | ENSP00000355001 | Brn-1, POU3F3, Otf-8, Brn1, Brain-1, Brain1 |
| IL20 | ENSP00000356065 | IL-20, IL20, Interleukin 20, interleukin-20 |
| SYNE1 | ENSP00000356224 | Syne-1, cpg2, SYNE1, nesprin-1, Nesprin 1, synaptic nuclear envelope protein 1, spectrin repeat-containing nuclear envelope protein 1, Enaptin, spectrin repeat containing, nuclear envelope 1, CpG_2, CpG-2, synaptic nuclear envelope protein-1 |
| CRTC2 | ENSP00000357622 | CRTC2, CREB regulated transcription coactivator 2, CREB-regulated transcription co-activator 2, CREB-regulated transcription coactivator 2 |
| FLG | ENSP00000357789 | filaggrin, FLG |
| S100A10 | ENSP00000357801 | p11, Hp 1-1, CLP11, S100A10, Hp1-1, p 11, p10, calpactin I light chain, Cal1l, P1-1, S-100A10, protein S100-A10, S100-A10, HP10, P10 Protein, GP11, protein S100A10 |
| TCF7L2 | ENSP00000358404 | Tcf4, Tcf-4, TCF7L2, hTcf-4, transcription factor 7-like 2, transcription factor-7-like 2, FJ010174, hTCF4, transcription factor 7-like2, transcription factor 7 like-2, transcription factor 7-like 2 (T-cell specific, HMG-box, transcription factor 7 like 2, transcription factor 7-like 2 (T cell specific, HMG-box, HMG box transcription factor 4, T cell-specific transcription factor 4, transcription factor 7-like 2, transcription factor 7-like-2, TCF/LEF transcription factor family, transcription factor-7 like 2, Transcription Factor-7 like-2, transcription factor-7-like-2 |

| | | |
|---|---|---|
| PHGDH | ENSP00000358417 | 3-PGDH, NLS, PHGDH, PGDH, phosphoglycerate dehydrogenase, NLS-1, NLS1, d-3-phosphoglycerate dehydrogenase, Malate dehydrogenase |
| DUSP5 | ENSP00000358596 | Dusp-5, hVH-3, DUSP, VH3, VH 3, DUSP5, dual specificity phosphatase 5, dual specificity phosphatase-5, dual-specificity phosphatase 5, DUSP 5, dual-specificity protein phosphatase-5, Dual-Specificity Protein Phosphatase 5 |
| NFKB2 | ENSP00000358983 | p100, p52, NFKB2, lyt-10, p105, Nuclear factor kappa-B subunit 2, Lyt10, p49/p100, oncogene lyt-10, P5-2, H2TF-1, NF-kB2, NFKB-2 |
| L1CAM | ENSP00000359077 | L1CAM, L1 CAM, neural cell adhesion molecule L1, Hs10, CAM-L1, L1 cell adhesion molecule, MIC5, CAM L1, CamL1, L1-CAM, Spg1p, neural cell-adhesion molecule L1, L1-cell adhesion molecule, CD 171, CD171, HS-10, hL1CAM, S10-P, NCAM-L1, L1 cell-adhesion molecule |
| BTRC | ENSP00000359206 | 1p22, beta-TrCP, beta-Trcp1, betaTrcp1, BTRC, betaTRCP, 1----p22, beta TrCP, E3RSIkappaB, pIkappaBalpha-E3 receptor subunit, FBW1a, Fbxw1, beta TrCP1, 1 p22, FWD1, beta-transducin repeat containing E3 ubiquitin protein ligase, F-box/WD repeat-containing protein 1A, B-TrCP, beta-transducin repeat-containing E3 ubiquitin protein ligase |
| CMPK1 | ENSP00000360939 | UMP-CMP kinase, UMP/CMP kinase, nucleoside diphosphate kinase, CMK, UMP-CMPK, UMPK, cytidylate kinase, nucleoside-diphosphate kinase, UCK, UMP/CMP-kinase, CMPK1, Ndk, CMPK, Cytidine monophosphate kinase, cytidine/uridine monophosphate kinase 1 |
| RALGDS | ENSP00000361120 | Ral guanine nucleotide dissociation stimulator, RalGDS, RalGEF, Ral-GDS, Ral GEF, RGF, Ral guanine nucleotide exchange factor, Ral GDS |
| PTCH2 | ENSP00000361266 | patched2, PTCH2, ptc2, patched 2, Patched-2, PTC-2 |
| CTSA | ENSP00000361562 | PPCA, cathepsin S, cathepsins, cathepsin A, deamidase, PPGB, protective protein for beta-galactosidase, lysosomal protective protein, carboxypeptidase-L, protective protein cathepsin A, cathepsin-S, C-TSA, carboxypeptidase L |
| STK4 | ENSP00000361892 | Mst1, STK4, Krs-2, MST-1, serine/threonine kinase-4, mammalian sterile 20-like 1, serine/threonine-protein kinase 4, mamma-lian STE20-like protein kinase 1, serine/threonine kinase 4, Mammalian ste20-like protein kinase 1 |

| | | |
|---|---|---|
| STXBP1 | ENSP00000362399 | Munc18-1, UNC-18, p67, syntaxin binding protein 1, STXBP1, n-Sec1, Munc-18-1, Unc18, nSec1, rbSec1, syntaxin-binding protein 1, syntaxin- binding protein 1 |
| GSN | ENSP00000362924 | gelsolin, Actin-depolymerizing factor, brevin, GSN, actin depolymerizing factor, AGel, 2F-H1, Gel-solin, Ad-F |
| TNFSF15 | ENSP00000363157 | TL1A, TNFSF15, TL1, tumor necrosis factor superfamily, tumor-necrosis factor superfamily, tumor necrosis factor superfamily, member 15, vascular endothelial cell growth inhibitor, VEGI, tumor necrosis factor super-family, Tumor necrosis factor (ligand) superfamily, member 15, tumor necrosis factor-superfamily, TNF superfamily member 15, tumor necrosis factor ligand superfamily member 15, TNF super-family member 15, tumor necrosis factor super family |
| TRIM63 | ENSP00000363390 | striated muscle RING zinc finger protein, MuRF1, MURF-1, MURF-2, MuRF2, muscle specific ring finger protein 1, Muscle-specific RING finger protein 1, TRIM 63, muscle-specific RING-finger protein 1, muscle-specific RING-finger protein-1, TRIM63, E3 ubiquitin-protein ligase TRIM-63 |
| TXN | ENSP00000363641 | thioredoxin, TRX, Trx1, Htrx1, Txn, TRX-1, ATL-derived factor, Trx 1, hTrx-1, TRDX, 1 TRs |
| EDA | ENSP00000363680 | EDA-A1, ED-A, EDA-A2, tumor necrosis factor superfamily, ectodysplasin A, HectD1, Ectodysplasin-A, eda, ED1-A1, EDA1, ED1-A2, EDA2, tumor-necrosis factor superfamily, hED, Eda A1, tumor necrosis factor super-family, tumor necrosis factor-superfamily, EDA-1, E-D-A, tumor necrosis factor super family |
| MAP1LC3A | ENSP00000363970 | Lc3, MAP1LC3A, LC 3, LC-3, autophagy-related protein LC3 A, LC3A, microtubule-associated protein 1 light chain 3A, MAP1-LC3A, autophagy-related protein LC3A, microtubule-associated proteins 1A/1B light chain 3A, LC 3A, microtubule-associated protein 1 light chain 3 alpha |
| XPA | ENSP00000364270 | XPA, XPAC, xeroderma pigmentosum group A complementing protein, Xeroderma Pigmentosum Complementation Groups |
| PADI1 | ENSP00000364620 | PDI, PADI1, peptidylarginine deiminases, pad1, peptidylarginine deiminase type I, EC 3.5.3.15, pad-1, Pad1p, PADI 1, peptidyl arginine deiminases, Peptidylargininedeiminase 1, PDI1, PD-I |

| | | |
|---|---|---|
| SDHB | ENSP00000364649 | SDHB, sdh, Sdh1p, Sdh2p, sdh B, SDH1, SDH2, pGL4, PGL-4, SDH-IP, PGL 4, succinate dehydrogenase complex, subunit B, iron sulfur (Ip, SD- HB, succinate dehydrogenase complex iron sulfur subunit B, succinate dehydrogenase complex iron-sulfur subunit B, SDH-B |
| TNFRSF1B | ENSP00000365435 | tumor necrosis factor receptor 2, TNFRSF1B, p75, TNFR2, TNF-R2, T nfr2, TNF-RII, TNFR-II, Tnfr-2, TNF-R75, TBPII, CD120b, TNF receptor superfamily member 1B, HP75, TNFR II, TNFRII, TNFR80, p75 TNFR, TNF-RSF1B, tumor necrosis factor-receptor 2, p75TNFR, tumor necrosis factor receptor type II, TNF-R-II, p75 TNF-R, tumor necrosis factor receptor-2, TNF R75, HP-75, TNF RII, TNF R2, TNFR1BP, tumor necrosis factor receptor superfamily, member 1B, TNFR1B, tumor necrosis factor receptor superfamily member 1B, tumor necrosis factor-receptor superfamily member 1B, TNFR75, TNFR-75 |
| KLK6 | ENSP00000366047 | KLK7, neurosin, kallikreins, protease m, KLK6, Zyme, kallikrein-6, kallikrein 6, hK6, Bssp, KLK 7, kallikrein-related peptidase 6, Kallikrein-related peptidase-6, kallikrein related peptidase 6 |
| MOG | ENSP00000366095 | myelin oligodendrocyte glycoprotein, MOG, myelin-oligodendrocyte-glycoprotein, myelin-oligodendrocyte glycoprotein |
| THBD | ENSP00000366307 | Thrombomodulin, Thbd, BDCA3, BDCA-3, CD141, 5 to -3 |
| CTNNBIP1 | ENSP00000366474 | inhibitor of beta-catenin and Tcf-4, beta-catenin-interacting protein ICAT, inhibitor of beta-catenin and TCF4, CTNNBIP1, catenin beta interacting protein 1, catenin, beta interacting protein 1 |
| TBC1D4 | ENSP00000366863 | KIAA0603, AS160, Akt substrate of 160 kDa, TBC1D4, TBC1 domain family member 4, TBC1 domain family, member 4 |
| PRKCZ | ENSP00000367830 | PKCzeta, PKC zeta, PKC-zeta, Prkcz, protein kinase C-zeta, protein kinase Czeta, protein kinase C zeta, PKCz |
| XDH | ENSP00000368727 | XDH, XOR, xanthine dehydrogenase/oxidase, XAN1 |
| IL2RA | ENSP00000369293 | p55, CD25, IL2RA, IL-2R, IL2R, IL-2RA, Tac antigen, IL-2-R, Tac-antigen, IL-2 R, p 55, hCD25, IL 2R, Interleukin-2 receptor subunit alpha, IL 2-R, HP55, IL2-RA, CD-25, CD 25 |
| TMSB4X | ENSP00000370010 | thymosin beta4, thymosin-beta 4, thymosin beta 4, Ptmb4, thymosin beta-4, thymosin-beta4, TMSB4X, TMSB4, thymosin-beta-4, thymosin beta 4, X-linked |
| PDE4A | ENSP00000370078 | PDE4A, PDE4, PDE-46, DPDE2, pde46, PDE 4, Phosphodiesterase-4A, PDE4 A, PDE-4, phosphodiesterase 4A, PDE-4A |

| | | |
|---|---|---|
| AKR1C2 | ENSP00000370129 | AKR1C2, dihydrodiol dehydrogenase/bile acid-binding protein, DD/BABP, aldo-keto reductases, MCDR2, aldo-keto reductase family 1, member C2, HAKRd, 3 alpha-HSD3, aldoketoreductases, DDH2, Dd2, 3alpha-HSD3, aldoketo reductases, dihydrodiol dehydrogenase 2 |
| TP53BP1 | ENSP00000371475 | 53BP1, TP53BP1, p202, P53BP1, TP53-binding protein 1, tumor protein p53 binding protein 1, 53-BP1, 53BP-1 |
| VLDLR | ENSP00000371532 | VLDLR, very low-density lipoprotein receptor, CAMRQ1, very low density lipoprotein receptor, VLDL-R, very-low-density lipoprotein receptor, very-low-density-lipoprotein receptor, CARMQ1, VLDLR-CH, very-low-density lipoprotein-receptor, very-low density lipoprotein receptor |
| FECH | ENSP00000372326 | EC 4.99.1.1, FECH, ferrochelatase, heme synthase, protoheme ferrolyase |
| STAG1 | ENSP00000372689 | SA-1, stromal antigen 1, SA1, STAG1, HSA 1, Stromal Antigen-1 |
| GPX3 | ENSP00000373477 | glutathione peroxidase 3, GSHPx-P, selenoprotein S, plasma glutathione peroxidase, EC 1.11.1.9, Gpx3, Extracellular glutathione peroxidase, glutathione peroxidase-3, GPx-3, selenoprotein-S |
| GARS1 | ENSP00000373918 | GlyRs, glycyl tRNA synthetase, Smad1, glycyl-tRNA synthetase, GARS, Smad-1, hsMAD1, SMAD 1, GA rs, 2 PMF, glycyl tRNA-synthetase |
| AFDN | ENSP00000375960 | AF6, AF-6, afadin, MLL-AF6, MLLT4, AFDN, l-afadin |
| RNF34 | ENSP00000376258 | RNF34, hRFI, CARP-1, Human Ring-Finger homologous to Inhibitor of apoptosis protein, CARP1, CARP- 1, ring finger protein 34 |
| PIAS3 | ENSP00000376765 | PIAS3, protein inhibitor of activated STAT3, protein inhibitor of activated Stat 3 |
| NME1-NME2 | ENSP00000376889 | NM23-LV, nucleoside diphosphate kinase B, nm23-H2, PuF, NME2, nucleoside diphosphate kinase, NDPK-B, nucleoside-diphosphate kinase, c-myc purine-binding transcription factor PuF, NDK, NDK-B, NDPK B, NM23b, nm-23H2, NDPKB, NM23-B, NME1-NME2, NME/NM23 nucleoside diphosphate kinase 2, nucleoside diphosphate kinase-B, NDKB, Nm23H2, nm23 H2, NME 2, nm 23-H2 |
| GAP43 | ENSP00000377372 | neuromodulin, GAP43, growth-associated protein-43, growth-associated protein 43, protein F1, GAP 43, growth associated protein 43, growth associated protein-43, p-p46, Gap-43 |

| | | |
|---|---|---|
| CFI | ENSP00000378130 | I factor, complement factor I, C3b inactivator, I-factor, C3b-INA, CF I, C3b-inactivator, Cfi, C3b/C4b inactivator |
| CCL26 | ENSP00000378365 | TSC1, eotaxin-3, CCL26, SCYA26, small inducible cytokine subfamily A,  member 26, Eotaxin3, TSC-1, TSC 1, CCL-26, TS-C1, TS- C1, eotaxin 3, chemokine (C-C motif) ligand 26, chemokine ligand 26, C-C motif chemokine ligand 26 |
| CLDN3 | ENSP00000378577 | claudin-3, C7orf1, claudin 3, CLDN3, RVP1, CPETR2, CLDN-3, CLDN 3, claudin3 |
| MPRIP | ENSP00000379156 | RIP3, p116Rip, M-RIP, Myosin phosphatase-Rho interacting protein, KIAA 0864, MPRIP, RIP 3, MRIP |
| ITGB1 | ENSP00000379350 | integrin beta1, CD29, integrin beta subunits, integrin beta 1, FNRB, cd 29, GPIIa, integrin-beta1, VLA beta, VLA-beta, integrin beta-1, ITGB1, integrinbeta1, ITGB1P, integrin-subunit beta-1, integrin subunit beta 1, integrin-beta 1 |
| THRB | ENSP00000379904 | thyroid hormone receptor beta, Thrb, GRTH, c-erbA beta, ERBA beta, ERBA2, c-erbA-beta, thyroid hormone receptor-beta, c-erbA2, thyroid hormone receptor-beta 1, thyroid hormone receptor B, oncogene ERBA2, thyroid hormone receptor beta1, thyroid hormone receptor beta-1, thyroid hormone receptor,  beta, thyroid hormone receptor beta 1, ErbA-beta, Thrb1, Thrb2, NR1A2, avian erythroblastic leukemia viral oncogene homolog 2, Generalized Resistance to Thyroid Hormone |
| KHSRP | ENSP00000381216 | KHSRP, KSRP, p75, HP75, KH-type splicing-regulatory protein, KH-type splicing regulatory protein, FUSE-binding protein 2, HP-75, Far-upstream element-binding protein 2, FBP2, far upstream element-binding protein 2, KH type splicing regulatory protein, FUBP2 |
| CST3 | ENSP00000381448 | cystatin C, CST 3, CST3, gamma-trace, gamma trace, cystatin-C, cystatinC |
| MRAP | ENSP00000382684 | FGD2, Falp, MRAP, melanocortin 2 receptor accessory protein, melanocortin-2 receptor accessory protein, melanocortin- 2 receptor accessory protein, M-RAP |
| BCL6 | ENSP00000384371 | LAZ3, BCL6, BCL-6, BCL5, LAZ-3, B-cell CLL/lymphoma 6, ZNF51, B cell CLL/lymphoma 6, Bcl6a, B-cell lymphoma 6 protein, ZBTB27 |
| RTL10 | ENSP00000384924 | Bop, BH3-only protein, BH3 only protein, BH-3 only protein |

| | | |
|---|---|---|
| NLRC4 | ENSP00000385090 | CARD12, Ipaf, Nlrc4, NLR family, CARD domain containing 4, NOD-like receptor C4, NLR family CARD domain-containing protein 4, I PAF, NLR family, CARD-domain-containing 4, ICE protease-activating factor, NLR family CARD domain containing 4, NLR family CARD domain-containing 4, NLR family CARD domain containing protein 4 |
| NOLC1 | ENSP00000385410 | Nopp140, hNopp140, p130, nucleolar phosphoprotein p130, NOLC1, nucleolar and coiled-body phosphoprotein 1, NS5ATP13, Nopp-140 |
| TNFSF18 | ENSP00000385470 | hGITRL, tumor-necrosis factor superfamily, AITRL, tumor necrosis factor superfamily, tumor necrosis factor super-family, GITRL, tumor necrosis factor-superfamily, GITR-L, TNFSF18, tumor necrosis factor super family |
| ARHGAP35 | ENSP00000385720 | GRF1, p190-A, p190 RhoGAP, p190RhoGAP, GRF-1, p190-RhoGAP, GRLF1, ArhGAP35, p190A RhoGAP, p190-A RhoGAP, Rho GTPase-activating protein 35, Glucocorticoid receptor DNA binding factor 1, p190A |
| ITGA6 | ENSP00000386896 | CD49f, ITGA6, integrin alpha-subunits, integrin alpha subunits, integrin alpha 6, Integrin alpha6, integrin-alpha-6, ITGA6p, integrin subunit alpha 6 |
| TBXAS1 | ENSP00000389414 | TBXAS1, TXAS, thromboxane A synthase, TXS, thromboxane A synthase 1, CYP5A1, thromboxane-A-synthase |
| CYLD | ENSP00000392025 | CYLD, deubiquitinating enzyme CYLD, cylindromatosis, cyld1, MFT, Mft1, cylindromatosis (turban tumor syndrome, CYLDI, MF T, MFT-1, CYLD lysine 63 deubiquitinase |
| TMEM182 | ENSP00000394178 | TMEM182 |
| PDZK1 | ENSP00000394485 | PDZK1, CAP70, CLAMP, PDZ domain containing 1, NHERF3, PDZ domain-containing 1 |
| MAPKAPK3 | ENSP00000396467 | MAPKAPK3, 3pK, MK3, chromosome 3p kinase, MAPKAP-K3, MK 3, mitogen-activated protein kinase-activated protein kinase 3, MAPKAPK 3, 3 she, 3 PK |
| ENDOU | ENSP00000397679 | p11, PP11, p 11, placental protein 11, EndoU, Hp 1-1, Hp1-1, P1-1 |
| PRF1 | ENSP00000398568 | perforin, PRF1, perforin 1, cytolysin, Pfp, perforin1, lymphocyte pore-forming protein, PFN1, P1P, FLH2, perforin-1, hPFN1, PFN-1 |
| EXOSC6 | ENSP00000398597 | p11, p 11, Hp1-1, Hp 1-1, P1-1, MTR-3, EXOSC6 |

| | | |
|---|---|---|
| NCOA2 | ENSP00000399968 | 2p15, 2-p15, GRIP-1, GRIP1, TIF2, 4-q13, 5 due, NCOA2, SRC-2, transcriptional intermediary factor 2, hTIF2, 5 TMS, SRC2, 2----p15, 4q 13, TIF-2, transcriptional intermediary factor-2, 5-E15, nuclear receptor co-activator 2, nuclear receptor coactivator 2, 4-PPs, 5 T MR, 5TMS, 5-e19 |
| WEE1 | ENSP00000402084 | Wee1, WEE1Hu, Wee1A, wee 1, Wee1 homolog, 2 in 6, Wee1-like protein kinase, WEE1 G2 checkpoint kinase |
| CPAMD8 | ENSP00000402505 | VIP, CPAMD8, C3 and PZP-like alpha-2-macroglobulin domain-containing protein 8, CPAMD8 p, K-CaP |
| CCNQ | ENSP00000402949 | StAR, FAM58A, cyclin M, STA R |
| LTA | ENSP00000403495 | lymphotoxin-alpha, TNFbeta, lymphotoxin alpha, TNF-beta, tumor-necrosis factor superfamily, tumor necrosis factor superfamily, TNF beta, TNFb, lymphotoxin A, Lta, tumor necrosis factor super-family, lymphotoxin-a, LT-A, tumor necrosis factor-superfamily, TNF-b, lymphotoxin- alpha, TNFSF1, 1 TNR, tumor necrosis factor super family |
| TRPV4 | ENSP00000406191 | VR-OAC, OTRPC4, TRPV4, vanilloid receptor-related osmotically activated channel, transient receptor potential cation channel, subfamily V,  member 4, Trp12, VRL-2, transient receptor potential cation channel subfamily V member 4, TRPV-4, TRPV4 p |
| LAMP2 | ENSP00000408411 | LAMP2, LAMP-2, lysosomal-associated membrane protein 2, h-lamp-2, lgp110, Lamp 2, lysosome-associated membrane glycoprotein 2, Lysosomal-Associated Membrane Protein-2, lysosomal associated membrane protein 2, lysosome associated membrane glycoprotein 2, LAMP2 p |
| IFNL3 | ENSP00000409000 | IL-28B, interleukin 28B, IFNL3, IL28B, IFNlambda3, interferon-lambda-3, IFN-lambda-3, IFN-lambda3, interleukin-28B, interferon lambda 3, interleukin28B, IFN lambda 3, interleukin 28-B, IL28-B, interferon lambda-3, IFN-lambda 4, IFN lambda 4, interferon-lambda 3, interferon-lambda3, IFN-lambda 3, IL 28B, Interleukin-28B, IFN-Lambda4, interleukin-28 B, IL 28-B, interferon,  lambda 3 |
| LTB | ENSP00000410481 | p33, TNFc, Lymphotoxin beta, lymphotoxin B, lymphotoxin-beta, tumor necrosis factor superfamily, tumor-necrosis factor superfamily, TNF-c, Ltb, tumor necrosis factor super-family, tumor necrosis factor-superfamily, TNFSF3, TNF c, tumor necrosis factor super family |
| RAB3GAP1 | ENSP00000411418 | p130, Rab3 GAP, RAB3GAP, RAB3GAP1, Rab3-GAP, Rab3 GTPase-activating protein catalytic subunit |

| | | |
|---|---|---|
| MST1 | ENSP00000414287 | MST1, Hgfl, DNF15S2, macrophage-stimulating 1, macrophage stimulating protein, hepatocyte growth factor-like protein, D3F15S2, macrophage-stimulating protein, MST-1, macrophage stimulating-1, DNF 15S2, macrophage stimulating 1, hepatocyte-growth-factor-like, hepatocyte growth factor-like |
| CTSO | ENSP00000414904 | cathepsin S, cathepsins, Cathepsin O, CTSO, cathepsin-S |
| SERPINA1 | ENSP00000416066 | alpha1 antitrypsin, alpha1-antitrypsin, alpha 1-antitrypsin, alpha 1 AT, alpha 1AT, Serpina1, A1AT, alpha-1-antitrypsin, alpha 1-AT, alpha1-AT, alpha 1 antitrypsin, alpha-1-protease inhibitor, alpha 1-protease inhibitor, HPI1, Aat, alpha-1-AT, alpha1-protease inhibitor, alpha1AT, alpha 1-anti-trypsin, alpha-1 antitrypsin, alpha 1-antiproteinase, alpha-1-anti-trypsin, alpha 1- antitrypsin, alpha1-anti-trypsin, alpha-1 protease inhibitor, alpha1 -antitrypsin, a1-AT, A1a, alpha1antitrypsin, alpha1-antiproteinase, serpin A1, Alpha-1 anti-trypsin, SERPINA 1, Alpha1 protease inhibitor, serpin peptidase inhibitor, clade A (alpha-1 antiproteinase, antitrypsin), member 1, alpha-1- antitrypsin, HPI-1, alpha1 antiproteinase, Alpha 1 anti-trypsin, SERPINA-1, alpha-1-antiproteinase, serpin family A member 1, alpha-1antitrypsin, a1-A |
| BAP1 | ENSP00000417132 | BAP1, BRCA1 associated protein-1, ubiquitin C-terminal hydrolases, ubiquitin carboxy-terminal hydrolase, BRCA1-associated protein 1, BRCA1-associated protein-1, UCH, BAP-1, BRCA-1 associated protein-1, BRCA-1 associated protein 1, BRCA1 associated protein 1, BRCA-1-associated protein 1, BRCA1 associated protein1, HBAP1, BRCA1 associated-protein 1 |
| MME | ENSP00000418525 | CD10, neprilysin, NEP, neutral endopeptidase 24.11, MME, Neutral endopeptidase, atriopeptidase, EC 3.4.24.11, enkephalinase, membrane metalloendopeptidase, neutral-endopeptidase, Common acute lymphocytic leukemia antigen, CD 10, membrane metallo-endopeptidase, CD-10, membrane-metalloendopeptidase |
| SMYD3 | ENSP00000419184 | SMYD3, SET and MYND domain containing protein 3, SET and MYND domain-containing protein 3, SET and MYND domain containing 3, 5 CCM, SET and MYND domain-containing 3, SET and MYND domain containing-3, 5EX0 |

| | | |
|---|---|---|
| GC | ENSP00000421725 | Gc-globulin, vitamin-D-binding protein, group-specific component, vitamin D-binding protein, hDBP, group specific component, GROUP-SPECIFIC COMPONENT, vitamin D binding protein, Dbp, Gc globulin, DBP/GC, VDBP, group-specific component (vitamin D-binding protein, GcMAF, vitamin D binding-protein, GC, vitamin D-binding protein, vitamin-D binding protein, Gc-MAF, Vitamin Dbinding protein, 1 lot, Gc, vitamin D binding protein |
| ESRRB | ENSP00000422488 | ERRbeta, ESRRB, ERR-beta, estrogen-related receptor beta, ERR2, ERR beta, ERRB2, NR3B2, estrogen-related receptor-beta, estrogen related receptor beta, ERRB |
| SMAD1 | ENSP00000426568 | Smad1, SMAD family, MADR1, Smad-1, bsp-1, MADH1, hsMAD1, mothers against decapentaplegic homolog 1, SMAD 1, SMAD family member 1 |
| FBXO32 | ENSP00000428205 | MAFbx, atrogin-1, FBXO32, atrogin 1, atrogin1, F-box protein 32, Fbx32, F-box only protein 32, F-box protein-32, muscle atrophy F-box protein |
| IKBKB | ENSP00000430684 | IkappaB kinase 2, IKKbeta, IKK2, IKK-beta, IKBKB, IKK beta, IKK-2, IKK-b, I kappa B kinase-2, IkappaB kinase-2, IkkB, Inhibitor of kappa light polypeptide gene enhancer in B-cells, kinase beta, inhibitor of nuclear factor kappa-B kinase subunit beta, inhibitor of nuclear factor kappa B kinase subunit beta |
| IL32 | ENSP00000432218 | IL-32, NK4, Interleukin-32, IL32, IL-32alpha, IL-32gamma, IL-32 beta, interleukin 32, IL-32 gamma |
| CFL1 | ENSP00000432660 | Cofilin, Cofilin-1, Cfl1, cofilin 1, 18 kDa phosphoprotein, cofilin1, CFL-1 |
| SLN | ENSP00000435380 | sarcolipin, S-Ln, Sln |
| ATP2A2 | ENSP00000440045 | ATP2A2, SERCA2, dAR, SERCA-2, sarcoplasmic/endoplasmic reticulum calcium ATPase 2, sarcoplasmic/endoplasmic reticulum calcium-ATPase 2, ATPase, Ca++ transporting, cardiac muscle, slow twitch 2, ATP2A2 p, ATPase sarcoplasmic/endoplasmic reticulum Ca2+ transporting 2, SERCA 2 |
| ARSL | ENSP00000441417 | ARSE, arylsulfatase E, ase, CDPX1 |
| CTSV | ENSP00000445052 | cathepsin V, cathepsins, cathepsin S, cathepsin L2, CTSL2, CTSV, cathepsin-S, cathepsin L-2 |
| VIM | ENSP00000446007 | VIM |

| | | |
|---|---|---|
| HNRNPC | ENSP00000451291 | hnRNP, HC1, hnRNPC, HC2, heterogeneous nuclear ribonucleoprotein C, hnRNP C, c2p, HC 2, heterogeneous nuclear ribonucleoproteins C1/C2, heterogeneous nuclear ribonucleoprotein C (C1/C2, HC-2, HC -2, SNRPC |
| XRCC3 | ENSP00000451974 | XRCC3, CMM6, RAD51-like, XRCC 3, X-ray repair complementing defective repair in Chinese hamster cells 3, X-ray repair cross-complementing protein 3, DNA repair protein XRCC3, X-ray repair cross complementing protein 3, X-ray repair cross complementing 3 |
| ZEB2 | ENSP00000454157 | SIP1, ZFHX1B, SMADIP1, ZEB2, Smad-interacting protein 1, Smad interacting protein-1, zinc finger homeobox 1B, Smad-interacting protein-1, ZEB-2, Smad interacting protein 1, hZeb2, Sip1p, Smad-interacting-protein-1, SIP-1, zinc finger E-box-binding homeobox2, Zinc finger E-box-binding homeobox 2, zinc finger homeo box 1 B, ZFHX1 B, SIP 1, zinc finger E-box binding homeobox 2, zinc-finger E-box binding homeobox 2, zinc finger E box-binding homeobox 2, ZFX1B, ZEB 2, AC009951, Zinc finger E-box-binding homeobox-2, Zinc Finger E-Box Binding Homeobox-2, zinc-finger E-box-binding homeobox 2 |
| MRC1 | ENSP00000455897 | macrophage mannose receptor, hMR, CD206, Mrc1, human mannose receptor, mannose receptor C type 1, MRC-1, mmr, CD 206, hCD206, macrophage-mannose receptor |
| MAP2K6 | ENSP00000468348 | MKK6, MAPKK6, SKK3, MEK6, MKK-6, mitogen-activated protein kinase kinase 6, MAP2K6, MEK-6 |
| GFAP | ENSP00000468500 | GFAP, intermediate filament protein, intermediate-filament protein |
| TNFSF14 | ENSP00000469049 | TR2, HTR2, HVEM-L, tumor-necrosis factor superfamily, TNFSF-14, Htr-2, tumor necrosis factor superfamily, Herpesvirus entry mediator ligand, TNFSF14, tumor necrosis factor super-family, TNFSF 14, tumor necrosis factor-superfamily, CD258, TNF superfamily member 14, tumor necrosis factor super family |
| SLC37A4 | ENSP00000476176 | GSD-1b, glucose 6-phosphate translocase, GSD 1b, G6PT, glucose-6-phosphate translocase, G6PT1, GSD-1c, GSD1b, SLC37A4, GSD 1c, glucose- 6-phosphate translocase, hG6PT |

| | | |
|---|---|---|
| CCL3 | ENSP00000477908 | SCYA3, MIP-1alpha, macrophage inflammatory protein-1alpha, MIP-1 alpha, MIP1 alpha, CCL3, G0S19-1, macrophage inflammatory protein 1 alpha, MIP-1a, LD78 alpha, macrophage inflammatory protein-1 alpha, LD78alpha, macrophage inflammatory protein 1alpha, MIP 1 alpha, MIP1alpha, macrophage-inflammatory protein-1 alpha, MIP1-alpha, MIP-1alpha, macrophage-inflammatory protein-1alpha, Macrophage inflammatory protein-1-alpha, Macrophage inflammatory protein 1-alpha, CCL-3, MIP1a, MIP 1-alpha, macrophage-inflammatory protein 1alpha, macrophage-inflammatory protein 1 alpha, hCCL3, chemokine (C-C motif) ligand 3, chemokine ligand 3, C-C motif chemokine ligand 3, macrophage inflammatory protein 1- alpha, CC L3 |
| NLRP1 | ENSP00000478516 | DEFCAP, NALP1, NLRP1, CARD7, SLEV1, 3-KAT, NLR family pyrin domain-containing 1, NLRP-1, NLR family, pyrin domain containing 1, NLR Family Pyrin Domain Containing 1, MSPc, 3 KAT |
| CCL14 | ENSP00000479097 | CC3, Hcc-1, Scyl2, HCC1, NCC-2, SY14, CCL14, chemokine ligand 14, CC-motif chemokine, HCC3, C C3, C-C-motif chemokine, C-C motif chemokine, HCC-3 |
| EPHX1 | ENSP00000480004 | mEH, microsomal epoxide hydrolase, EPHX1, EPHX, epoxide hydrolase 1, epoxide hydratase, HYL1, epoxide hydrolase-1 |
| FGF1 | ENSP00000480791 | aFGF, acidic fibroblast growth factor, fibroblast growth factor-1, FGF1, FGF-1, HBGF-1, ECGF, FGFA, Fibroblast growth factor 1, endothelial cell growth factor, ECGFB, heparin binding growth factor-1, heparin-binding growth factor 1, FGF 1, 2 HWA, fibroblast growth factor -1, a-FGF, FGF-alpha, fibroblast growth factor-1 (acidic |
| N | ENSP00000481127 | PRODH, POX, proline oxidase, proline dehydrogenase, PRODH2, PIG-6, proline dehydrogenase (oxidase) 1, PRODH1, proline dehydrogenase 1 |
| HNRNPDL | ENSP00000483254 | heterogeneous nuclear ribonucleoprotein D-like, hnRNP, HNRPDL, laAUF1, JKTBP, HNRNPDL, heterogeneous nuclear ribonucleoprotein D like |
| GHR | ENSP00000483403 | GHR, GHBP, growth hormone binding protein, growth hormone-binding protein, growth-hormone-binding protein, GH-BP, GH-R |

| | | |
|---|---|---|
| LEPROT | ENSP00000483521 | LEPR, OB-R gene-related protein, LEPROT, OB-RGRP, OB-R, leptin receptor overlapping transcript, leptin receptor gene-related protein, OBR, Lep-R, Endospanin1, Endospanin 1 |
| BTK | ENSP00000483570 | Btk, Bruton's tyrosine kinase, bpk, atk, bruton tyrosine kinase, Tec family tyrosine kinases, Bruton agammaglobulinemia tyrosine kinase, Xla, B-cell progenitor kinase, B cell progenitor kinase, 1B 55, IMD1, TEC-family tyrosine kinases, Bruton's tyrosine-kinase, tyrosine-protein kinase BTK |
| CCL4L2 | ENSP00000483609 | MIP-1beta, LAG1, HLA-G1, SCYA4L, MIP-1 beta, AT744.2, macrophage inflammatory protein-1beta, macrophage inflammatory protein 1 beta, Lag1p, LAG-1, SCYA4L2, SCYA4L1, MIP1-beta, macrophage inflammatory protein-1 beta, CCL4L1, MIP1beta, macrophage inflammatory protein 1beta, macrophage-inflammatory protein-1beta, macrophage inflammatory protein-1-beta, MIP1 beta, HLAG1, CCL4L, CCL4L2, HLAG-1 |
| CYP51A1 | ENSP00000003100 | CYP51, lanosterol 14 alpha-demethylase, CYP51A1, Sterol 14alpha-demethylase, P45014DM, lanosterol 14-alpha-demethylase, lanosterol 14alpha-demethylase, sterol 14 alpha-demethylase, lanosterol 14-alpha demethylase, lanosterol-14-alpha-demethylase |
| CDH17 | ENSP00000027335 | cadherin, HPT-1, CDH16, Liver-Intestine Cadherin, liver intestine-cadherin, CDH17, Cadherin-17, CDH-17, cadherin 17, Liver-intestine-cadherin, HPT1, cadherin17 |
| APOH | ENSP00000205948 | apoH, apolipoprotein H (beta2-glycoprotein I, apolipoprotein H, apolipoprotein H (beta 2-glycoprotein I, APO H, beta 2-glycoprotein I, beta2-glycoprotein I, beta-2-glycoprotein I, beta 2-glycoprotein-I, B2G 1, beta 2-glycoprotein 1, beta2 glycoprotein I, Beta2 glycoprotein 1, B2G1, beta2-glycoprotein-I, beta-2-glycoprotein 1, beta-2-glycoprotein-I, beta 2 glycoprotein I, Beta-2-glycoprotein-1, Beta-2 Glycoprotein I, beta-2 glycoprotein-1, beta2glycoprotein I, b2GP1, beta-2glycoprotein-I, anticardiolipin cofactor |
| VCL | ENSP00000211998 | vinculin, metavinculin, 4EHP, meta-vinculin, Vcl |
| MIF | ENSP00000215754 | MIF, Macrophage migration inhibitory factor, GIF, macrophage migration-inhibitory factor, 4 OSF, 4 GRN, phenylpyruvate tautomerase, MMIF, macrophage-migration inhibitory factor, macrophage-migration-inhibitory factor, 5 h vs |

| | | |
|---|---|---|
| PVALB | ENSP00000216200 | parvalbumin, PVALB |
| CTSG | ENSP00000216336 | cathepsins, cathepsin S, catG, cathepsin G, EC 3.4.21.20, CTSG, cathepsin-G, Cat G, CAT-G, cathepsin-S |
| CTSZ | ENSP00000217131 | Cathepsin S, cathepsins, Cathepsin Z, CTSZ, cathepsin X, cathepsin-Z, CTSX, cathepsin-S, cathepsin B2 |
| CTSH | ENSP00000220166 | cathepsin S, CTSH, cathepsin H, Cathepsins, cathepsin-S, procathepsin H |
| SFRP1 | ENSP00000220772 | secreted Frizzled-related proteins, sFRP1, sFRP-1, Secreted Frizzled-related protein 1, FRP1, Secreted frizzled related protein 1, FRP, secreted Frizzled-related protein-1, SARP2, secreted frizzled related proteins, FRP-1, SFRP, SFRP 1, secreted apoptosis-related protein 2, secreted frizzled related protein-1, Secreted-frizzled related protein 1, Secreted-frizzled related proteins, SFRP1 - p, Secreted-Frizzled-Related-Protein 1, secreted-frizzled-related protein 1 |
| RELB | ENSP00000221452 | RelB, I-Rel, transcription factor RelB, Rel B, Rel-B, RELB Proto-Oncogene, NF-KB Subunit |
| RNASEH2A | ENSP00000221486 | junB, AGS4, RNASEH2A, RNase H2A, Jun-B, RNase HI, jun b |
| RETN | ENSP00000221515 | resistin, RSTN, RETN, FIZZ3, ADSF |
| CYP3A5 | ENSP00000222982 | CYP3A5, hPCN3, cytochrome p-450 3A5, EC 1.14.14.1, cytochrome P450HLp2, cytochrome P4503A5, cytochrome P450 3A5, CYP 3A5, cytochrome P450 family 3, cytochrome P450, family 3, subfamily A, polypeptide 5 |
| TNFSF8 | ENSP00000223795 | CD153, CD30L, tumor necrosis factor superfamily, CD30 ligand, tumor-necrosis factor superfamily, CD30-L, TNFSF8, tumor necrosis factor super-family, TNF superfamily member 8, tumor necrosis factor-superfamily, tumor necrosis factor super family |
| CTSC | ENSP00000227266 | cathepsin C, CTSC, cathepsins, DPPI, cathepsin S, dipeptidyl-peptidase I, dipeptidyl peptidase I, hMS, DPP1, DPP I, cathepsin-C, cathepsin-S, PAL-S |
| GORASP2 | ENSP00000234160 | p59, GRASP55, Golgi reassembly stacking protein of 55 kDa, HP59, GORASP2, GRS2 |
| MFN2 | ENSP00000235329 | Mfn2, mitofusin 2, Mfn-2, CMT2A2, mitofusin-2, mitofusin2, MARF, MFN2 p, hMfn2, MFN 2 |
| MMP8 | ENSP00000236826 | MMP8, neutrophil collagenase, Matrix metalloproteinase 8, CLG1, MMP-8, Matrix metalloproteinase-8, 1 mM B, matrixmetalloproteinase-8, matrix metalloproteinase -8, 3 dpf, Matrix metallopeptidase-8, matrix metal-loproteinase 8, 3 DPE, MMP -8 |

| | | |
|---|---|---|
| DUSP4 | ENSP00000240100 | DUSP4, mitogen-activated protein kinase phosphatase-2, Dual specificity phosphatase 4, MKP2, MAP kinase phosphatase-2, MKP-2, Dusp 4, mitogen-activated protein kinase phosphatase 2, dual-specificity phosphatase 4, dual specificity protein phosphatase 4, Dual-specificity protein phosphatase 4, Dual specificity phosphatase-4, MAP kinase phosphatase 2 |
| SOX9 | ENSP00000245479 | Sox9, Sra-1, SR-A1, SRA1, CMD1, transcription factor Sox9, SRY (sex-determining region Y) box 9, CMPD1, Sox-9, transcription factor Sox-9, SRY (sex determining region Y)-box 9, SRY- (sex determining region Y-) box 9, SRY-box 9, SRY (sex-determining region Y)-box 9, h sox9, SRY-box-9, SRY box 9, SO X9 |
| TNFSF9 | ENSP00000245817 | 4-1BB ligand, 4-1BBL, tumor necrosis factor superfamily, tumor-necrosis factor superfamily, 4-1BB-L, 4-1 BB ligand, CD137L, TNFSF9, tumor necrosis factor super-family, tumor necrosis factor-superfamily, 41BBL, tumor necrosis factor super family |
| CD70 | ENSP00000245903 | CD70, tumor necrosis factor superfamily, CD70 molecule, CD27 ligand, tumor-necrosis factor superfamily, TNFSF7, tumor necrosis factor super-family, tumor necrosis factor-superfamily, CD27L, tumor necrosis factor super family |
| CANX | ENSP00000247461 | CN X, p90, Calnexin, cnx, CANX, IP90 |
| PYCARD | ENSP00000247470 | TMS-1, TMS1, apoptosis-associated speck-like protein containing a CARD, Asc, Pycard, AS-C, AS c, target of methylation-induced silencing 1, apoptosis-associated specklike protein containing a CARD, target of methylation induced silencing 1, PYD and CARD domain containing, apoptosis-associated speck-like protein-containing a CARD, target of methylation-induced silencing-1, T MS, apoptosis associated speck-like protein containing a CARD, PY-CARD, PYD and CARD domain containing protein |
| PMPCB | ENSP00000249269 | p52, MPP11, peptidase B, PMPCB, peptidase-B, MPP11p, P5-2 |

| | | |
|---|---|---|
| G6PC1 | ENSP00000253801 | GSD1a, G-6-Pase, GSD-1, G6PT, G6Pase, glucose-6-phosphatase, glucose-6 phosphatase, GSD 1a, glucose 6-phosphatase, glucose-6-phosphatase catalytic subunit, GSD-1a, GSD1, G6PC, G6PC1, EC 3.1.3.9, glucose-6-phosphatase-alpha, G6-Pase, hG6PT, glucose-6-phosphatase, catalytic, glucose-6-phosphatase, catalytic subunit, glucose-6- phosphatase, G6 Pase, glucose 6 phosphatase, hG6PC, hG6Pase, G-6Pase, glucose 6 phosphatase catalytic subunit |
| HNF1A | ENSP00000257555 | HNF1, Tcf1, HNF-1alpha, HNF1A, HNF-1 alpha, HNF1alpha, hepatocyte nuclear factor-1alpha, HNF-1, LFB-1, TCF-1, LFB1, hepatocyte nuclear factor 1alpha, hepatocyte nuclear factor-1 alpha, hepatocyte nuclear factor 1 alpha, transcription factor 1, HNF1 alpha, LF-B1, transcription factor-1, liver-specific transcription factor LFB1, HNF 1alpha, hHNF1, HNF-1a, hepatocyte nuclear factor-1-alpha, HNF-1-alpha, hepatocyte nuclear factor 1-alpha, HNF 1A, HNF1 homeobox A, Transcription Factor1, HNF1A p, HNF1-A |
| HUS1 | ENSP00000258774 | HUS1, Hus1p, hHus1, hus-1, HUS1 checkpoint clamp component |
| SRSF1 | ENSP00000258962 | SF2, SRSF1, HSF2, AS-F, splicing factor 2, Serine/arginine-rich splicing factor 1, SRp30a, splicing factor-2, SFRS1, alternate splicing factor, SF-2, HSF-2, serine and arginine-rich splicing factor 1, splicing factor2, Serine and Arginine Rich Splicing Factor 1 |
| GTF2H4 | ENSP00000259895 | TFIIH, p52, TFB2, 5 of 4, P5-2, GTF2H4, general transcription factor IIH, polypeptide 4, general transcription factor IIH subunit 4 |
| BARD1 | ENSP00000260947 | BARD1, BRCA1-associated ring domain protein 1, BRCA1-associated RING domain 1, BRCA1-associated RING-domain 1, BRCA1-associated RING domain-1, hBARD1, BRCA1 associated RING domain protein 1, BRCA1 Associated RING Domain 1 |
| GUCY2C | ENSP00000261170 | StAR, STa receptor, GC-C, GUCY2C, Intestinal guanylate cyclase, heat stable enterotoxin receptor, EC 4.6.1.2, guanylyl cyclase C, guanylate cyclase 2C, heat-stable enterotoxin receptor, guanylyl cyclase-C, guanylylcyclase C, GUC2C, STA R |
| CCND2 | ENSP00000261254 | cyclin D2, cyclin-D2, CCND2, cyclinD2, CCND 2, CCND-2, G1/S-specific cyclin D2, G1/S-specific cyclin-D2 |

| | | |
|---|---|---|
| SLC6A4 | ENSP00000261707 | 5-HTTLPR, 5-HTT, SLC6A4, 5HTT, htt, SERT, 5-hydroxytryptamine transporter, 5HTTLPR, 5-HT transporter, hSERT, 5-HT T, 5HTT-LPR, 5-HT-T, 5HT transporter, 5 HTT, 5-HTT LPR, 5-HTT-LPR, 5HTT LPR, 5HTTL-PR, 5- HTTLPR, solute carrier family 6 member 4, Ser-T, 5-hydroxy tryptamine transporter, solute carrier family 6, member 4, SLC 6A4, solute carrier family 6 (neurotransmitter transporter), member 4, solute carrier family-6 member-4, sodium-dependent serotonin transporter, 5-hydroxytryptamine-transporter |
| IL17B | ENSP00000261796 | IL-17B, IL-20, IL20, Interleukin 20, interleukin-17B, interleukin-20, IL17B, interleukin 17B |
| CYP46A1 | ENSP00000261835 | CYP46, CYP46A1, cholesterol 24-hydroxylase, 3 MDR, 3-mdr, cytochrome P450 46A1, cholesterol-24-hydroxylase |
| ATF1 | ENSP00000262053 | ATF-1, ATF1, ATF 1, FUS/ATF-1, activating transcription factor 1, TREB36, EWS-ATF-1, Activating transcription factor-1, EWS-ATF1, FUS/ATF1 |
| RBL2 | ENSP00000262133 | p130, Rbl2, PRB2, Rb2, retinoblastoma-like 2, retinoblastoma-like protein 2, pRb 2 |
| CD33 | ENSP00000262262 | CD33, siglec-3, p67, hCD33, CD33 molecule, CD33 antigen, hP67, CD 33, hP 67 |
| RPE65 | ENSP00000262340 | p63, RPE65, mRPE65, sRPE65, hRPE65, retinal pigment epithelium-specific 65-kDa protein, rd12, retinal pigment epithelium-specific protein 65kDa, Retinal pigment epithelium-specific protein 65 kDa, retinal pigment epithelium-specific 65 kDa protein, retinoid isomerohydrolase |
| FOXF1 | ENSP00000262426 | Foxf1, freac-1, FKHL5, Forkhead Box f1, Forkhead box protein F1, Forkhead Box-F1 |
| TEP1 | ENSP00000262715 | TP1, TEP1, TLP1, telomerase protein component 1, telomerase-associated protein 1, tlp-1, TP-1, hTEP1, TEP-1, telomerase-associated protein-1, telomerase associated protein 1, p240, TP 1, telomerase associated protein1, hTP1, telomerase associated protein-1, HTP-1, hTLP1 |
| ELL | ENSP00000262809 | ELL, eleven-nineteen lysine-rich leukemia gene, eleven nineteen lysine-rich leukemia gene, RNA polymerase II elongation factor ELL, eleven-nineteen lysine-rich leukemia protein, ell-1, elongation factor for RNA polymerase II |
| MAP2K2 | ENSP00000262948 | MAP2K2, MEK-2, MEK2, MEK2 p, MKK2, mitogen-activated protein kinase kinase 2, Mek 2, MAPK/ERK Kinase 2, MAPKK2 |

| | | |
|---|---|---|
| TCF3 | ENSP00000262965 | E2A, TCF-3, Tcf3, p75, VDIR, transcription factor 3, transcription factor-3, ITF-1, HP75, HP-75, transcription-factor3, immunoglobulin enhancer-binding factor E12/E47, trans-cription factor 3, E2A immunoglobulin enhancer-binding factor E12/E47, transcription factor E2-alpha |
| UCP1 | ENSP00000262999 | uncoupling protein-1, UCP1, uncoupling protein 1, UCP-1, Ucp, thermogenin, hUCP1, uncoupling-protein-1, UCP 1, uncoupling protein1, mitochondrial brown fat uncoupling protein 1 |
| TUBB4A | ENSP00000264071 | beta5, beta 5, DYT4, TUBB4A, TUBB5, TUBB4 |
| MTRR | ENSP00000264668 | MSR, MTRR, methionine synthase reductase, cblE, 5-methyltetrahydrofolate-homocysteine methyltransferase reductase, methionine-synthase reductase, 2 QTL |
| PPARGC1A | ENSP00000264867 | PPARGC1, PGC-1, PGC-1alpha, peroxisome proliferator-activated receptor-gamma coactivator-1 alpha, PPARgamma coactivator 1alpha, PPARGC1A, peroxisome proliferator-activated receptor-gamma coactivator-1alpha, peroxisome proliferator-activated receptor gamma coactivator-1alpha, PGC1, PGC-1 alpha, PPAR-gamma coactivator 1alpha, peroxisome proliferator-activated receptor gamma coactivator 1alpha, PPAR gamma coactivator 1alpha, Peroxisome proliferator-activated receptor-gamma coactivator 1alpha, 5-two, Pgc1alpha, peroxisome proliferator-activated receptor gamma,  coactivator 1 alpha, PPAR gamma coactivator-1 alpha, peroxisome-proliferator-activated receptor-gamma coactivator-1alpha, Pgc1a, PPAR gamma coactivator 1-alpha, PGC1-alpha, peroxisome proliferator-activated receptor gamma co-activator 1alpha, PGC 1, Peroxisome proliferator-activated receptor gamma coactivator 1 alpha, Peroxisome proliferator-activated receptor-gamma co-activator-1alpha, peroxisome proliferator activated receptor-gamma co-activator-1 alpha, peroxisome proliferator-activated receptor gamma coactivator-1 alpha, PGC-1A, PPAR-gamma coactivator-1alpha, Peroxisome proliferator activated receptor gamma coactivator 1alpha, Peroxisome proliferator activated receptor gamma coactivator 1 alpha, peroxisome proliferator-activated receptor-gamma-coactivator-1alpha, Peroxisome proliferator-activated receptor gamma co-activator-1 alpha, PGC -1alpha, peroxisome proliferator activated receptor gamma coactivator-1 alpha, PPARgamma coactivator-1alpha, peroxisome proliferator-activated receptor-gamma coactivator1alpha, peroxisome proliferator-activated receptor gamma-coactivator-1alpha, PPAR-gamma-coactivator 1alpha, PPARG co-activator-1alpha, PPARgammacoactivator-1alpha, peroxisome proliferator-activated receptor gamma,  coactivator 1alpha, 3-D24, Peroxisome proliferator-activated receptor gamma coactivator 1-alpha, peroxisome proliferator-activated receptor-gamma coactivator 1 |

| | | |
|---|---|---|
| | | alpha, peroxisome proliferator-activated receptor gamma-coactivator 1-alpha, peroxisome proliferator-activated receptor gamma co-activator-1-alpha, PGC-1-alpha, peroxisome proliferator-activated receptor gamma coactivator-1 alpha, peroxisome proliferator-activated receptor gamma coactivator-1 alpha, peroxisome proliferator-activated receptor gamma co-activator 1-alpha, peroxisome proliferator activated receptor gamma co-activator 1 alpha, peroxisome proliferator-activated receptor gamma coactivator-1-alpha, peroxisome proliferator-activated receptor gamma coactivator1-alpha, PPARg co-activator 1-alpha, peroxisome proliferator-activated receptor gamma co-activator 1 alpha, PPARgamma-coactivator-1alpha, PPAR-gamma co-activator 1-alpha, peroxisome proliferator activated receptor gamma coactivator-1alpha, peroxisome proliferator-activated receptor gamma-coactivator 1 alpha, PPAR gamma coactivator 1 alpha, PPAR-Gamma-Coactivator-1 Alpha, peroxisome proliferator activated receptor gamma co-activator 1-alpha, PGC1-a, peroxisome proliferator-activated receptor-gamma coactivator 1-alpha |
| MCM2 | ENSP00000265056 | MCM family, MCM2, nuclear protein BM28, CDCL1, CCNL1, BM28, Mcm2p, MCM-2, minichromosome maintenance complex component 2, DNA replication licensing factor MCM2 |
| GOLPH3 | ENSP00000265070 | Golgi protein, Golgi-associated protein, coat protein, GPP34, Vps74p, GOLPH3, Golgi phosphoprotein 3, Golgi phosphoprotein-3, coat-protein, GOPP1 |
| CALB1 | ENSP00000265431 | Calbindin, d28k, Calb1, CaLB, calbindin 1, Calbindin-1, calbindin D28, cal- bindin |
| GAL | ENSP00000265643 | Gal1, GALN, galanin, gal-1, galanin message associated peptide, galanin peptides, galanin-message-associated peptide, galanin message-associated peptide, gal 1 |
| CCAR1 | ENSP00000265872 | CARP-1, CCAR1, cell cycle and apoptosis regulatory protein-1, Cell cycle and apoptosis-regulatory protein-1, CARP1, cell division cycle and apoptosis regulator 1, CARP- 1, CCAR-1 |
| PRDM16 | ENSP00000270722 | MEL1, PRDM16, PR domain containing 16, PR domain containing protein 16, PR-domain-containing 16, Mel-1, hmel1, PRDM-16, PR domain-containing 16, PR/SET domain 16, PR/SET Domain Family, PR-domain-containing-16 |

| | | |
|---|---|---|
| CTSK | ENSP00000271651 | cathepsin K, cathepsins, cathepsin S, CTSK, cathepsin O, cathepsin-K, cathepsin X, 2 A-to, PknD, CTSO, CathepsinK, cathepsin-S, U20280, EC 3.4.22.38 |
| STAR | ENSP00000276449 | StAR, steroidogenic acute regulatory protein, StarD1, steroidogenic acute regulator, STA R |
| MTA2 | ENSP00000278823 | MTA2, NuRD complex, MTA1-L1, MTA-2, MTA1L1, metastasis associated 1 family, member 2, metastasis-associated 1 family, member 2, Metastasis Associated 1 Family Member 2 |
| FBXW7 | ENSP00000281708 | Cdc4, hCdc4, Fbw6, Fbxw7, FBW7, SEL-10, Cdc4p, hAgo, Fbxw6, hSel-10, F-box and WD-40 domain protein 7, SEL10, F-box and WD40 domain protein 7, F-box and WD repeat domain-containing 7, F-box- and WD repeat domain-containing 7, F-box/WD repeat-containing protein 7, F-box and WD repeat domain containing-7, F-box and WD repeat domain containing 7, F-box/WD-repeat-containing protein 7, FBXW7 p, F-box and WD repeat domain-containing7 |
| TNFSF4 | ENSP00000281834 | OX40L, OX40 ligand, TNFSF4, gp34, CD134L, OX-40 ligand, tumor-necrosis factor superfamily, HGP34, tumor necrosis factor superfamily, tumor necrosis factor super-family, tumor necrosis factor superfamily, member 4, tumor necrosis factor (ligand) superfamily, member 4, tumor necrosis factor ligand superfamily member 4, tumor necrosis factor-superfamily, OX40-ligand, CD252, OX-40L, OX40-L, tumor necrosis factor super family, TXGP1 |
| BMP6 | ENSP00000283147 | bone morphogenetic proteins, BMP6, Vgr-1, BMP-6, bone morphogenetic protein-6, bone morphogenetic protein 6, vgr, hBMP-6, bone-morphogenetic protein-6, bone morphogenetic proteins, Bmp, BMP 6 |
| UBASH3B | ENSP00000284273 | p70, Sts-1, TULA-2, Sts1, T-cell ubiquitin ligand-2, UBASH3B, ubiquitin-associated and SH3 domain-containing B, Ubiquitin Associated and SH3 Domain Containing B |
| WNT3A | ENSP00000284523 | WNT3A, Wnt family, Wnt-3a, Wnt-family, wingless-type mmtv integration site family, member 3a, Wnt family member 3A, Wnt 3a |
| LET7A5P | hsa-let-7a-5p | let-7a, hsa-let-7a, let7a, let-7a-5p, hsa-let-7a-5p |
| MIR1260B | hsa-miR-1260b | miR-1260b, hsa-miR-1260b, miR1260b |
| MIR200C3P | hsa-miR-200c-3p | miR-200c, hsa-miR-200c, mir200C, miR-200c-3p, miR 200c, hsa-miR-200c-3p |
| MIR4103P | hsa-miR-410-3p | miR-410, miR410, miR-410-3p, hsa-miR-410-3p |
| MIR630 | hsa-miR-630 | miR-630 |

| | | |
|---|---|---|
| MIR92A3P | hsa-miR-92a-3p | miR-92a, miR-92, hsa-mir-9-2, hsa-mir-92a, miR-92a-3p, miR92a, miR-9-2, hsa-miR-92a-3p, hsa-miR-92, hsamiR-92a-3p, MIR9-2 |
| ATXN8OS | ATXN8OS | SCA8, ATXN8OS, KLHL1AS, SCA-8 |
| GSTT2B | ENSP00000290765 | GSTT2, GSTT2B, glutathione S-transferase theta 2, GST-T2 |
| LRRC3 | ENSP00000291592 | leucine-rich repeat containing protein 3, leucine-rich repeat-containing protein 3 |
| SCGB3A1 | ENSP00000292641 | HIN-1, high in normal-1, high-in-normal 1, high-in-normal-1, SCGB3A1, HIN1, UGRP2 |
| BMP10 | ENSP00000295379 | BMP-10, bone morphogenetic proteins, Bmp10, bone morphogenetic protein-10, bone morphogenetic proteins, Bone Morphogenetic Protein 10, Bmp |
| MITF | ENSP00000295600 | MITF, microphthalmia-associated transcription factor, COMMAD, MITF-C, CMM8, microphthalmia associated transcription factor, melanogenesis associated transcription factor, MITF c |
| CXCL5 | ENSP00000296027 | SCYB5, ENA-78, CXCL5, chemokine ligand 5, epithelial-derived neutrophil activating protein-78, ENA78, CXCL-5, chemokine (C-X-C motif) ligand 5, chemokine(C-X-C motif) ligand 5, ENA 78, C-X-C motif chemokine ligand 5, chemokine ligand5, CXC motif chemokine ligand 5, C-X-C motif chemokine 5, neutrophil-activating peptide ENA-78 |
| PPBP | ENSP00000296028 | Tc2, connective tissue-activating peptide-III, TC-1, beta-thromboglobulin, BTG1, Tc1, CTAP III, CTAP-III, BTG-1, NAP-2, beta TG, connective tissue activating peptide-III, beta-TG, PPBP, TC-2, platelet basic protein, connective tissue-activating peptide III, LA-PF-4, Pro-platelet basic protein, neutrophil-activating peptide-2, TC 2, connective tissue activating peptide III, CXCL7, beta thromboglobulin, MDGF, Nap2, HTC 2, T-C-2, T-C-1, chemokine ligand 7, pBP, proplatelet basic protein, CTAPIII, Pro Platelet Basic Protein |
| ABCE1 | ENSP00000296577 | RLI, RNase L inhibitor, RNS4I, RNAseL inhibitor, Rli1, ABCE1, RNASEL-inhibitor, ATP-binding cassette, subfamily E, member 1, RNase L inhibitor, ATP-binding cassette sub-family E member 1, ATP-binding cassette subfamily E member 1, rnasel-1 |
| CARTPT | ENSP00000296777 | cocaine- and amphetamine-regulated transcript, Cartpt, cocaine and amphetamine-regulated transcript, cocaine- and amphetamine-regulated-transcript, cocaine and amphetamine regulated transcript, cocaine-and amphetamine regulated transcript |

| | | |
|---|---|---|
| SOX17 | ENSP00000297316 | Sox17, SOX17 p, SOX17-p, Sox-17, SRY-box 17, SRY-BOX17, SRY (sex determining region Y)-box 17, transcription factor Sox17 |
| CFL2 | ENSP00000298159 | Cofilin-2, CFL2, cofilin 2, cofilin2 |
| AKR1E2 | ENSP00000298375 | testis specific protein, htAKR, aldo-keto reductases, testis-specific protein, testis aldo-keto reductase, aldoketoreductases, aldoketo reductases, AKR1E2 |
| PRDX3 | ENSP00000298510 | AOP-1, PRDX3, Prx III, MER5, Aop1, peroxiredoxin III, peroxiredoxin 3, peroxiredoxin-3, antioxidant protein 1, antioxidant protein-1, Prx-III, PrxIII, SP-22 |
| NELL1 | ENSP00000298925 | Nell1, NRP1, Nrp-1, nel-like 1, Nell-1, nel-like1, Nel-Like Protein-1, Neural EGFL-Like 1, neural EGFL like 1, NEL-Like Protein 1 |
| HIF1AN | ENSP00000299163 | HIF1AN, FIH-1, factor inhibiting HIF-1, FIH1, hypoxia-inducible factor asparagine hydroxylase, factor-inhibiting HIF-1, HIF-1AN, hypoxia-inducible factor 1-alpha inhibitor, hypoxia inducible factor 1 alpha subunit inhibitor, Factor inhibiting HIF1, hypoxia inducible factor 1-alpha subunit inhibitor |
| OR2D2 | ENSP00000299459 | HB2, hHb2, HH-B2 |
| SLC9A5 | ENSP00000299798 | NHE5, SLC9A5 |
| TPX2 | ENSP00000300403 | C20orf2, TPX2, p100, DIL-2, HCA519, HCA90, DIL2, C20orf1, Fls353, Repp86, targeting protein for Xklp2, HD IL-2, TPX-2, HD-IL2, HDIL-2, HD-IL-2 |
| CDK2AP2 | ENSP00000301488 | p14, HP-14, p 14, HP14, CDK2AP2 |
| KCNAB3 | ENSP00000302719 | Kvbeta3, KCNA3B, aldo-keto reductases, aldoketoreductases, aldoketo reductases |
| GUSB | ENSP00000302728 | beta-glucuronidase, GUSB, beta G1, beta glucuronidase, EC 3.2.1.31, MPS7 |
| MTNR1A | ENSP00000302811 | mt1, MT-1, HMT-1, MTNR1A, hMT1, melatonin receptor 1 A, melatonin receptor 1A, melatonin receptor type 1A |
| PTGER4 | ENSP00000302846 | EP4, prostaglandin E receptor 4, EP4R, PTGER4, PTGER2, hEP4, prostaglandin E2 receptor EP4 subtype, E-P4, EP-4, Prostanoid EP4 receptor, prostaglandin E receptor-4 |
| SLC23A1 | ENSP00000302851 | SVCT1, Slc23a1, SLC23A2, solute carrier family 23 member 1, SVCT-1, Sodium-dependent vitamin C transporter-1 |
| IL17D | ENSP00000302924 | IL-22, Il22, IL-17D, IL-2 2, interleukin-17D, IL17D, interleukin 17D, IL 22 |

| | | |
|---|---|---|
| LRP8 | ENSP00000303634 | ApoER2, LRP8, apolipoprotein E receptor 2, Low-density lipoprotein receptor-related protein 8, low density lipoprotein receptor-related protein 8, LRP-8, Apolipoprotein E receptor-2, Low density lipoprotein receptor related protein 8, MCI-1 |
| COPS6 | ENSP00000304102 | COP9 signalosome subunit 6, CSN6, Vpr-interacting protein, hVIP, Vpr Interacting Protein, 4D10, COP9 subunit 6, COPS6 |
| TM4SF1 | ENSP00000304277 | TM4SF1, L6P, tumor-associated antigen L6, transmembrane-4 L six family member 1, tumor associated antigen L6, transmembrane 4 L6 family member 1, Transmembrane-4 L-Six family member-1, Transmembrane-4-L-six-family member-1, transmembrane 4 L six family member 1, transmembrane 4 super family member 1, transmembrane-4 L6 family member 1 |
| RAC3 | ENSP00000304283 | Rac3, Rho family GTPases, Rho-family GTPases, Rac3 human, RAC 3, RAC-3 |
| BMP1 | ENSP00000305714 | Bmp1, BMP-1, Bone morphogenetic protein 1, bone morphogenetic proteins, procollagen C-endopeptidase, procollagen C-proteinase, Tld, PCP-2, bone morphogenetic protein-1, procollagen C proteinase, mammalian Tolloid protein, Pcp2, pcp, Bmp 1, bone morphogenetic proteins, PC p, Bmp, procollagen - C - proteinase |
| PRKCE | ENSP00000306124 | protein kinase C-epsilon, PRKCE, Protein kinase Cepsilon, protein-kinase-C epsilon, protein kinase C epsilon, Pkce, 5LIH |
| GRM5 | ENSP00000306138 | mGluR5, metabotropic glutamate receptor 5, GRM5, mGlu5, glutamate receptor, metabotropic 5, metabotropic glutamate receptor-5, mGluR 5, 5 CGD |
| ITPR1 | ENSP00000306253 | IP3R1, SCA16, InsP3R1, IP3R, Itpr1, SCA15, inositol 1, 4, 5-trisphosphate receptor type 1, type 1 inositol 1, 4, 5-trisphosphate receptor, SCA-16, ITPR1 p, inositol-1, 4, 5-triphosphate receptors, AC V, IP3 R1, IP3-R1, inositol 1, 4, 5-trisphosphate receptor, type 1, SCA29, IP3-R, inositol 1, 4, 5-triphosphate receptors, SCA1-5 |
| ADRA1B | ENSP00000306662 | alpha 1B-AR, ADRA1B, alpha1b-AR, alpha 1B-adrenergic receptor, ADRA1, alpha1B adrenergic receptor, alpha1B AR, alpha1B-adrenergic receptor, alpha 1B-adrenoreceptor, Alpha-1B adrenergic receptor, alpha-1B-adrenergic receptor |

| | | |
|---|---|---|
| PCSK1 | ENSP00000308024 | HPC1, PC-1, HPC3, Pc 1, prohormone convertase 3, SPC3, Nec-1, neuroendocrine convertase-1, prohormone convertase 1, NEC1, proprotein convertase 1, PCSK1, SP-C3, HPC-1, Pc1, PC-3P, prohormone convertase-1, HPC-3, pro-hormone convertase-1, proprotein convertase subtilisin/kexin type 1, Proprotein convertase subtilisin/kexin-type 1, PCSK-1 |
| PLG | ENSP00000308938 | plasminogen, Plg |
| TWNK | ENSP00000309595 | SCA8, Twinkle, C10orf2, TWNK, PEO1, ATXN8, chromosome 10 open reading frame 2, SCA-8, pEO, PEO-1, MTDPS7 |
| MAP3K11 | ENSP00000309597 | mlk3, MLK-3, Mixed lineage kinase 3, Mixed-lineage kinase 3, Sprk, Mixed lineage kinase-3, MAP3K11, mitogen-activated protein kinase kinase kinase 11, Mixed-lineage kinase-3, mitogen-activated protein kinase kinase kinase-11, SPR K |
| RIN1 | ENSP00000310406 | Rin1, Ras and Rab interactor 1 |
| PSMC5 | ENSP00000310572 | p45, Hs8, PSMC5, TRIP1, TRIP-1, Sug1, SUG-1, HS-8, Trip 1 |
| MAL | ENSP00000310880 | MAL, MyD88 adapter-like, myelin and lymphocyte protein, m AL, T-lymphocyte maturation associated protein, T-lymphocyte maturation-associated protein |
| CD7 | ENSP00000312027 | CD7, CD7 molecule, gp40, Leu9, Tp40, CD7 antigen, CD-7, L eu9, Tp41, Leu-9 |
| HP1BP3 | ENSP00000312625 | HP1-BP74, HP1BP3, heterochromatin protein 1 binding protein 3, heterochromatin protein 1-binding protein 3 |
| CD46 | ENSP00000313875 | CD46, Membrane cofactor protein, membrane co-factor protein, Tlx, CD46 molecule, MIC10, Mcp, MC p, hCD46, 3 in B |
| SGSH | ENSP00000314606 | sulfamidase, sulphamidase, EC 3.10.1.1, Sgsh, N-sulfoglucosamine sulfohydrolase, N-sulphoglucosamine sulphohydrolase |
| IL20RA | ENSP00000314976 | IL-20R1, IL-20Ralpha, IL20RA, IL-20RA |
| MOB4 | ENSP00000315702 | MOB1, MOB 3, Mob3, STRIPAK complex, Mob-1, class II mMOB1, hMOB1, hMOB3, MOB4 |
| COX5A | ENSP00000317780 | Cox5a, cytochrome c oxidase subunit 5a, COX Va |
| CEP57 | ENSP00000317902 | Translokin, CEP57, PIG8, Pig 8 |
| BID | ENSP00000318822 | BH3 interacting domain death agonist, BH3-interacting-domain death agonist, BH3-interacting domain death agonist, BH3 interacting-domain death agonist |
| CAMK2G | ENSP00000319060 | CaMK, calcium/calmodulin-dependent protein kinase II gamma, CAMK2G p, CAMKG, CAMK2G, CaM-K, calcium/calmodulin-dependent protein kinase IIgamma |

| | | |
|---|---|---|
| SERPINF2 | ENSP00000321853 | alpha2-antiplasmin, alpha 2-plasmin inhibitor, alpha 2-PI, alpha 2PI, alpha 2-antiplasmin, alpha2PI, alpha 2 antiplasmin, alpha2-plasmin inhibitor, Alpha-2 antiplasmin, alpha2-PI, AP-I, alpha2 antiplasmin, aap, alpha-2-plasmin inhibitor, SERPINF2, alpha-2-antiplasmin, serpin peptidase inhibitor, clade F, member 2, A2AP, ApI |
| MFAP3 | ENSP00000322956 | microfibril-associated protein-3, MFAP3 |
| PLEC | ENSP00000323856 | PLEC1, plectin, pcN, HD1, PLEC, plakins, EBS1, HD-1, plectin 1, EBS-1, Plectin-1, PLEC 1 |
| PENK | ENSP00000324248 | preproenkephalin, pre-proenkephalin, proenkephalin, PENK, PENKA, Proenkephalin A, PENK A, pro-enkephalin |
| ULK1 | ENSP00000324560 | UNC-51, ATG1, Unc-51 like autophagy activating kinase 1, Unc51.1, Unc-51-like kinase 1, hATG1, unc-51-like autophagy activating kinase 1, atg1p, UNC51-like kinase 1, Unc-51-like-kinase1, Unc-51 like kinase 1, UNC-51-like kinase1, Unc-51-like Kinase-1, Unc51like kinase 1, Unc-51-like autophagy-activating kinase 1, unc-51 like autophagy-activating kinase 1, UNC51 |
| CD28 | ENSP00000324890 | CD28, CD28 molecule, Tp44, CD28 antigen, CD-28, CD 28, t-p44 |
| PRX | ENSP00000326018 | PRX, periaxin |
| KLK4 | ENSP00000326159 | prostase, PRSS17, kallikreins, kallikrein, ARM-1, KLK4, Kallikrein 4, enamel matrix serine proteinase 1, ARM1, arm 1p, kallikrein-4, KLK-4, EMSP1, serine protease 17, KLK-L1, Kallikrein-related peptidase 4, P-STS, Kallikrein-related peptidase-4, kallikrein related peptidase 4, Arm 1, Kallikrein-related-peptidase-4, kalli krein |
| MAF | ENSP00000327048 | c-Maf, Maf, cMAF, V-maf avian musculoaponeurotic fibrosarcoma oncogene homolog, proto-oncogene c-Maf, v-maf musculoaponeurotic fibrosarcoma oncogene homolog, transcription factor Maf, MAF BZIP Transcription Factor |
| BMP8A | ENSP00000327440 | bone morphogenetic proteins, bone morphogenetic proteins, BMP8A, Bmp |
| SLC5A2 | ENSP00000327943 | SGLT2, SLC5A2, sodium/glucose cotransporter 2, sodium/glucose co-transporter 2, SGLT 2, SGLT-2, SGLT- 2, hSGLT2, sodium/glucose cotransporter-2, Sodium/glucose co-transporter-2 |
| IL25 | ENSP00000328111 | IL-17E, IL-25, IL17E, IL25, interleukin-25, hIL-17E, Interleukin-17E, Interleukin 25, interleukin 17E |
| IL20RB | ENSP00000328133 | IL-20R2, IL20RB, IL-20RB, IL20R2, interleukin-20 receptor beta |

| | | |
|---|---|---|
| DGKA | ENSP00000328405 | DAGK, 80-kDa diacylglycerol kinase, DGKA, diacylglycerol kinase alpha, DGKalpha |
| CADM1 | ENSP00000329797 | IGSF4, TSLC1, SynCAM, IGSF4A, Necl2, SynCAM 1, cell adhesion molecule-1, Cadm1, BL2, Necl-2, synaptic cell adhesion molecule, cell adhesion molecule 1, Tumor suppressor in lung cancer-1, RA175, SgIGSF, SynCAM1, Nectin-like protein 2, tumor suppressor in lung cancer 1, BL-2, HBL-2, TSLC-1, HBL2, CADM-1, cell adhesion molecule1, cell-adhesion molecule 1, cell-adhesion molecule-1, nectin-like-2, CADM 1, synaptic cell-adhesion molecule |
| PSMG1 | ENSP00000329915 | DSCR2, PAC-1, PAC1, Down Syndrome Critical Region gene 2, PSMG1, c21-LRP, chromosome 21 leucine-rich protein, PAC 1 |
| PDGFB | ENSP00000330382 | PDGFB, PDGF-2, c-sis, PDGF-B, PDGF2, proto-oncogene c-sis, platelet-derived growth factor B-chain, PDGF B, oncogenes--is, platelet-derived growth factor-beta polypeptide, platelet-derived growth factor B chain, protooncogene c-sis, platelet-derived growth factor-B chain, csis, platelet-derived growth factor subunit B, platelet-derived growth factor beta polypeptide, platelet derived growth factor b-chain |
| CD86 | ENSP00000332049 | B7-2, B7.2, CD86, B-lymphocyte antigen B7-2, CD86 molecule, CD86 antigen, B72, CD 86, CD-86, 1 NCN |
| SLIT3 | ENSP00000332164 | Slit3, Slit-2, SLIT2, Slit1, slit homolog 3, SLIT 2 |
| H2AC20 | ENSP00000332194 | H2A, H-2a, H2-A, histone cluster 2 H2A family member C |
| RARB | ENSP00000332296 | hAP, RARB, retinoic acid receptor beta, Rar-b, retinoic acid receptor-beta, retinoic acid receptor B, RARbeta1, RAR-beta1, retinoic acid receptor,  beta, RA- RB, NR1B2, RAR beta 1 |
| CCR4 | ENSP00000332659 | CCR4, chemokine receptor 4, ChemR13, K5-5, CMKBR4, CC-CKR4, Ccr4p, chemokine receptor-4, C-C chemokine receptor type 4, chemokine (C-C motif) receptor 4, CD19 4, chemokine (C-C motif) receptor-4, C-C motif chemokine receptor 4, C-C motif chemokine receptors, CD19-4, CC chemokine receptor type 4 |
| KCND2 | ENSP00000333496 | Kv4.2, Kcnd2, voltage-gated potassium channel subunit Kv4.2, Potassium Voltage-gated Channel Subfamily D Member 2 |
| CYP2F1 | ENSP00000333534 | CYP2F, CYP2F1, EC 1.14.14.1, cytochrome P450 2F1 |

| | | |
|---|---|---|
| PRKD1 | ENSP00000333568 | PKD, PKCmu, Protein kinase D, Protein kinase D1, PRKCM, PRKD1, PKC mu, PKCm, serine/threonine-protein kinase D1, protein kinase D 1, Pkd1 p, protein kinase D-1 |
| ABCC5 | ENSP00000333926 | MRP5, pABC11, SMRP, MOAT-C, ABCC5, ATP binding cassette sub-family C, multi-drug resistance-associated protein 5, ATP-binding cassette sub-family C, ATP binding cassette subfamily C |
| CYP2R1 | ENSP00000334592 | CYP2R1, vitamin D 25-hydroxylase, vitamin D-25-hydroxylase, cytochrome P450 2R1, cytochrome P450, family 2, subfamily R, polypeptide 1, hCYP2R1 |
| LIMK1 | ENSP00000336740 | LIMK1, LIMK, LIMK-1, Lim domain kinase 1, LIM-domain kinase 1 |
| ATF4 | ENSP00000336790 | activating transcription factor 4, CREB-2, Atf4, ATF-4, CREB2, TAXREB67, activating transcription factor-4 |
| PNRC1 | ENSP00000336931 | B4-2, PRR2, B42, PNRC1 |
| DACT1 | ENSP00000337439 | DAPPER1, Dact1, HDPR1, Dapper homolog 1, HDPR-1, Dpr1, dishevelled binding antagonist of beta-catenin 1 |
| LCK | ENSP00000337825 | lck, p56lck, LSK, 2 of 4, lymphocyte-specific protein tyrosine kinase, Src family tyrosine kinases, T cell-specific protein tyrosine kinase, lymphocyte-specific protein-tyrosine kinase, 1q6-8, p56 LCK, YT16, Src-family tyrosine kinases, 3 MPM, lymphocyte specific protein tyrosine kinase, lymphocyte cell-specific protein-tyrosine kinase, LCK proto-oncogene, Src family tyrosine kinase, LC-k, T-cell-specific protein tyrosine kinase |
| SUV39H1 | ENSP00000337976 | SUV39H1, Suv39h, Suv39 h1, MG44, Suv-39h1, SUV39H 1, Suv39-h1, KMT1A, suppressor of variegation 3-9 homolog 1, 3 MTS, suppressor of variegation 3-9 homolog1 |
| HEY1 | ENSP00000338272 | HEY1, hesr1, Hey-1, hairy/enhancer-of-split related with YRPW motif-1, hairy/enhancer-of-split related with YRPW motif 1, HERP2, Hrt1, Hrt-1, Hairy/enhancer-of-split related with YRPW motif protein 1, hes-related family bHLH transcription factor with YRPW motif 1 |
| LGALS12 | ENSP00000339374 | GRIP-1, galectin-12, GRIP1, LGALS12 |
| DFFB | ENSP00000339524 | caspase-activated nuclease, caspase-activated DNase, DFFB, DFF40, caspase-activated deoxyribonuclease, C-AD, caspase activated DNAse, CaD, DFF-40, C AD |
| IDS | ENSP00000339801 | iduronate-2-sulfatase, EC 3.1.6.13, Iduronate 2-sulfatase, alpha-L-iduronate sulfate sulfatase, IdS, iduronate 2 sulfatase, iduronate-2 sulfatase, iduronate -2- sulfatase, I-D-S |

| | | |
|---|---|---|
| TPST2 | ENSP00000339813 | TPST-2, tyrosylprotein sulfotransferase-2, 3 AP-1, TPST2, Tyrosylprotein sulfotransferase 2 |
| TCF7 | ENSP00000340347 | TCF1, TCF-1, transcription factor 7, Tcf7, T-cell factor 7, transcription factor-7, TCF-7, T-cell-factor-7, T cell-specific transcription factor-1, T Cell Factor 7, TCF/LEF transcription factor family |
| HUWE1 | ENSP00000340648 | HUWE1, HECT,  UBA and WWE domain-containing 1, HUWE1 p, 2 mul, ARF-BP1, UreB1, upstream regulatory element binding protein 1, hUREB1, HectH9, HECT,  UBA and WWE domain-containing protein-1, HECT,  UBA and WWE domain-containing protein 1 |
| SFN | ENSP00000340989 | 14-3-3Sigma, Sfn, Stratifin, 14-3-3 sigma, 5 MYC, 14-3-3-sigma, 14-3-3 protein sigma, epithelial cell marker protein-1 |
| NANOS2 | ENSP00000341021 | NOS2, NANOS2, NOS-2, hNOS2, NOS 2 |
| CSN2 | ENSP00000341030 | beta-casein, CSN2, CASB, beta -casein, beta casein, casein beta |
| ACACB | ENSP00000341044 | ACC2, EC 6.4.1.2, acetyl-CoA carboxylase 2, acetyl-CoA carboxylase-beta, ACC-beta, ACACB, ACC-2, ACCbeta, acetyl-coenzyme A carboxylase beta, Acetyl-CoA carboxylase beta, acetyl-coenzymeA carboxylase beta, acetyl CoA carboxylase 2, acetyl-coA carboxylase2, Acetyl-CoA carboxylase-2 |
| CAV3 | ENSP00000341940 | caveolin-3, CAV3, Cav-3, VIP-21, M-caveolin, VIP21, caveolin 3, caveolin3, LGMD-1c, CAV3 p |
| PRDX6 | ENSP00000342026 | 24-kDa protein, 1 cys, 24 kDa protein, PRX, p29, EC 1.11.1.7, antioxidant protein 2, peroxiredoxin 6, 1-cys peroxiredoxin, Prdx 6, Prdx6, peroxiredoxin-6, Peroxiredoxin6 |
| SP100 | ENSP00000343023 | HSP100, SP100, LYSp100B, nuclear autoantigen SP100, Sp100 nuclear antigen, Sp-100 |
| GJA4 | ENSP00000343676 | Cx37, connexin37, Gja4, gap junction protein alpha-4, connexin 37, hCx37, gap junction protein,  alpha 4,  37 kD, CX 37, Connexin-37 |
| TP53INP1 | ENSP00000344215 | p53DINP1, TP53INP1, p53-dependent damage-inducible nuclear protein 1, stress-induced protein, stress induced protein, tumor protein p53 inducible nuclear protein 1, tumor protein p53-inducible nuclear protein 1 |
| GATA2 | ENSP00000345681 | GATA2, GATA-2, NF-E1b, GATA-binding protein 2, GATA binding protein 2, GATA2 p, GATA binding protein2, endothelial transcription factor GATA-2 |

| | | |
|---|---|---|
| NFYA | ENSP00000345702 | CBFb, NF-YA, HAP2, CBFA, CBF-A, CBF-B, nuclear transcription factor Y subunit alpha, NFYA, Hap 2 |
| TAP1 | ENSP00000346206 | APT1, hy3, PSF1, RING4, TAP1, D6S114E, hTAP1, TAP-1, TAP 1, ABCB2, ATP-binding cassette subfamily B, transporter 1, ATP-binding cassette, subfamily B, Hy-3, peptide transporter TAP1 |
| COPS8 | ENSP00000346340 | Csn8, COP9 signalosome subunit 8, 4D10, COPS8 |
| MRTFA | ENSP00000347847 | MAL, MKL1, MRTF-A, myocardin family, megakaryocytic acute leukemia, megakaryoblastic leukemia-1, myocardin-related transcription factor A, MKL, Megakaryoblastic leukemia 1, KIAA 1438, m AL, myocardin-related transcription factor-A, MKL-1, Myocardin Related Transcription Factor-A, m-KL, myocardin related transcription factor A, MRTFA |
| C1D | ENSP00000348107 | LRP1, C1D, SUN-CoR, Small Unique Nuclear receptor CoRepressor, HC1D, LRP-1, Rrp47, LRP 1 |
| PTGER3 | ENSP00000349003 | EP3, EP3-VI, HEP3, EP3-IV, EP3-I, EP3-II, PTGER3, hEP3-I, EP3-III, prostanoid EP3-receptor, prostaglandin E receptor 3, prostaglandin E receptor 3 (subtype EP3, prostaglandin-E receptor 3, Prostaglandin E2 receptor EP3 subtype |
| CILK1 | ENSP00000349458 | intestinal cell kinase, MRK, ICK, KIAA0936, MAK-related kinase, I-CK |
| KLRG1 | ENSP00000349477 | KLRG1, MafA, killer cell lectin-like receptor G1, MAFA-L, 2F1, MAFA-like receptor, killer-cell lectin-like receptors, mast cell function-associated antigen, KLR, killer cell lectin-like receptors, killer cell lectin-like receptor subfamily G member 1, KLRG-1, killer-cell lectin like receptor G1 |
| PRODH | ENSP00000349577 | PRODH2, PRODH, proline oxidase, proline dehydrogenase, PIG-6, proline dehydrogenase (oxidase) 1, PRODH1, proline dehydrogenase 1 |
| ADIPOR2 | ENSP00000349616 | adiponectin receptors, ADIPOR2, Adipo-R2, adiponectin receptor 2, adiponectin receptor-2, PAQR-2, adiponectin-receptors |
| ATP2B4 | ENSP00000350310 | PMCA4b, PMCA4, ATP2B2, hPMCA4, ATP2B4, hPMCA4b |
| NR3C2 | ENSP00000350815 | mineralocorticoid receptor, NR3C2, mcr, 5 HCV, mineralocorticoid-receptor, mLR, Nuclear Receptor Subfamily 3 Group C Member 2 |

| | | |
|---|---|---|
| CCL20 | ENSP00000351671 | macrophage inflammatory protein-3alpha, MIP-3alpha, CCL20, Exodus-1, SCYA20, liver and activation-regulated chemokine, macrophage inflammatory protein-3 alpha, larc, MIP3alpha, chemokine (C-C motif) ligand 20, macrophage inflammatory protein 3alpha, liver- and activation-regulated chemokine, Liver-and activation-regulated chemokine, MIP-3 alpha, macrophage-inflammatory protein 3alpha, macrophage inflammatory protein-3a, macrophage inflammatory protein 3 alpha, MIP3-alpha, Chemokine ligand 20, macrophage-inflammatory protein-3alpha, CCL-20, MIP3a, C-C motif chemokine ligand 20, hCCL20, CC-motif chemokine 20, MIP-3a |
| KCNQ2 | ENSP00000352035 | KCNQ2, EBN1, Kv7.2, K V 7.2, KV 7.2, potassium voltage-gated channel, KQT-like subfamily, member 2, EBN, EIEE7, Potassium channel, voltage-gated KQT-like subfamily Q, member 2, potassium voltage-gated channel subfamily Q member 2, KCNQ2 p |
| CD163 | ENSP00000352071 | CD163, CD 163, M1-30, hemoglobin scavenger receptor, CD163 molecule, CD163 antigen, CD-163, scavenger receptor cysteine-rich type 1 protein M130, Scavenger receptor cysteine rich type 1 protein M130 |
| ELAVL3 | ENSP00000352162 | Elav, HuC, ELAVL3, ple21, hu-C, HuC-L, Hu antigen C, Hu C |
| ANXA10 | ENSP00000352248 | ANXA10, annexins, annexin A10, ANX A10, ANXA 10, Annexin10 |
| ATP2A3 | ENSP00000353072 | SERCA3, ATP2A3, SERCA 3 |
| DHDDS | ENSP00000353104 | DHDDS, cPt, cis-isoprenyltransferase, c Pt |
| CACNA1A | ENSP00000353362 | SCA6, SCA 6, EA-2, CACNA1A, EA2, CaV2.1, he-A2, CACNL1A4, SCA-6, mhp, Cav 2.1, HPCA, calcium channel, voltage-dependent, P/Q type, alpha 1A subunit, calcium channel, voltage-dependent, P/Q type, alpha-1A subunit, CACNA1A p, Calcium Voltage-Gated Channel Subunit Alpha1 A |
| F8 | ENSP00000353393 | F8B, coagulation factor VIII, FVIII, F8C, Antihemophilic factor, f_8, F VIII, coagulation factor VIII, procoagulant component, HemA, F8 B, F8 p, HF8 |
| ATP2B2 | ENSP00000353414 | PMCA2, ATP2B2, PMCA-2, PMCA 2, plasma membrane calcium-transporting ATPase 2 |

| | | |
|---|---|---|
| MAP2 | ENSP00000353508 | MAP2, Microtubule-associated protein 2, MAP-2, MAP2c, Map2B, MAP 2, microtubule-associated protein-2, MAP-2c, microtubule associated protein 2, Microtubule associated protein-2, MAP2a, MAP - 2, microtubule-associated-protein-2 |
| CES1 | ENSP00000353720 | ce-1, ACAT, CES2, CES1A1, acyl-coenzyme A:cholesterol acyltransferase, egasyn, CES1, Ses1, EC3.1.1.1, hCE-1, PCE-1, CES-1, carboxylesterase 1, C-E1, CeH, acyl coenzyme A: cholesterol acyltransferase, Acyl coenzyme A:cholesterol acyltransferase, acylcoenzyme A:cholesterol acyltransferase, HCE1, CES-2, Acyl-coenzyme A: cholesterol acyltransferase, triacylglycerol hydrolase, A-C-A-T, EC 3.1.1.1, CE1, TGH, SES-1, SES 1, acyl-coenzyme A:cholesterol acyl-transferase, CES1A2, liver carboxylesterase 1, carboxyl esterase 1, acyl coenzyme A:cholesterol acyl transferase, 1Yah, hCES1, hCES2 |
| MT-CYB | ENSP00000354554 | cytochrome b, cytb, cyt b, MT-CYB, MTCYB, cytochrome-b, CYB, mitochondrially encoded cytochrome B, Cyt-b |
| OPA1 | ENSP00000354681 | OPA1, dynamin-like guanosine triphosphatase, MGM1, OPA1 p, Mgm1p, NTg, KIAA0567, N-Tg, OPA-1, optic atrophy protein 1, npG, MGM-1 |
| CALD1 | ENSP00000354826 | l-CaD, h-CaD, Cdm, Caldesmon, CaD, LCAD, C-AD, CALD1, hCAD, c DM, Caldesmon 1, C AD, c-DM |
| SGMS1 | ENSP00000354829 | Mob-1, Mob1, hMOB1, SMS1, sphingomyelin synthase 1, SGMS1, sms-1 |
| MT-ND4 | ENSP00000354961 | ND4, NADH-dehydrogenase 4, NADH dehydrogenase subunit 4, MTND4, MT-ND4, NADH dehydrogenase 4, ND 4 |
| ABCG1 | ENSP00000354995 | ABCG1, hABCG1, ABC8, ATP-binding cassette subfamily G, ATP-Binding Cassette, Sub-Family G (WHITE), Member 1, ATP-binding cassette subfamily G member 1, ATP-binding cassette sub-family G, ATP-binding cassette, subfamily G, member 1, ABCG-1 |
| CTNNBL1 | ENSP00000355050 | p14, CTNNBL1, P14L, nuclear-associated protein, HP-14, beta-catenin-like protein 1, p 14, HP14 |
| SELENON | ENSP00000355141 | SEPN1, selenoprotein N, SelN, SELENON, selenoprotein N, 1 |
| AKR7A3 | ENSP00000355377 | aldo-keto reductases, aldoketoreductases, AFAR2, AKR7A3, aldoketo reductases, aflatoxin aldehyde reductase, aflatoxin B1 aldehyde reductase member 3 |
| AGT | ENSP00000355627 | AGT, angiotensinogen, A-G-T, Ag t, Ag-T, 4-APH |
| CENPF | ENSP00000355922 | HCP 1, HCP1, CENP-F, CENPF, mitosin, centromere protein F, HCP-1, Centromere protein-F |

| | | |
|---|---|---|
| CD55 | ENSP00000356030 | DAF, decay-accelerating factor, CD55, decay accelerating factor, complement decay-accelerating factor, CD55 molecule, CHAPLE, complement decay accelerating factor |
| MAPKAPK2 | ENSP00000356070 | MAPKAPK-2, MAPKAPK2, MAPKAP-K2, mitogen activated protein kinase-activated protein kinase 2, MK2, Mitogen-activated protein kinase-activated protein kinase 2, mitogen-activated protein kinase-activated protein kinase-2, MAPKAPK 2, MAP kinase-activated protein kinase-2, MAPK-APK2, MAPKAP K2, MAP kinase-activated protein kinase 2, Mitogen-activated Protein Kinase activated Protein Kinase-2, mitogen activated protein kinase activated protein kinase 2, Mitogen-Activated Protein Kinase Activated Protein Kinase 2, MK-2 |
| ADORA1 | ENSP00000356205 | adenosine receptors, adenosine A1 receptor, ADORA1, RDC7, adenosine-A1 receptor, adenosine A1-receptor, ADORA 1 |
| UCHL5 | ENSP00000356425 | UCH37, ubiquitin C-terminal hydrolase UCH37, UCHL5, ubiquitin C-terminal hydrolases, ubiquitin carboxyl-terminal hydrolase L5, Ubiquitin C-terminal hydrolase-L5, Uch-L5, ubiquitin carboxyl-terminal hydrolase isozyme L5, ubiquitin C-terminal hydrolase L5 |
| RNF2 | ENSP00000356480 | RNF2, Ring1B, RING2, BAP1, RING finger protein 2, DinG, BAP-1, ring finger protein 1B, HBAP1, RING1-B |
| NCF2 | ENSP00000356505 | NCF-2, p67phox, p67-phox, NCF2, neutrophil cytosolic factor 2, p67 phox, neutrophil cytosol factor 2 |
| NPHS2 | ENSP00000356587 | podocin, NPHS2, NPHS2 p, NPHS2-p, NPHS2, podocin |
| SERPINC1 | ENSP00000356671 | antithrombin, AT3, AT-III, Antithrombin III, ATIII, AT III, antithrombin-III, anti-thrombin, At-3, anti-thrombin III, serpin C1, SERPINC1, 1ant, serpin family C member 1, anti-thrombin-III, anti thrombin-III, antithrombin- III |
| XCL2 | ENSP00000356793 | chemokine ligand 2, chemokine ligand-2, XCL2, hXCL2 |
| PMF1 | ENSP00000357256 | PMF-1, hPMF-1, PMF1, polyamine modulated factor-1, Polyamine-modulated factor 1, polyamine-modulated factor-1 |
| PMF1-BGLAP | ENSP00000357259 | PMF-1, hPMF-1, PMF1, polyamine modulated factor-1, Polyamine-modulated factor 1, polyamine-modulated factor-1 |
| LAMTOR2 | ENSP00000357288 | p14, endosomal adaptor protein p14, MAPBPIP, ROBLD3, HP-14, p 14, LAMTOR2, endosomal adaptor protein, HP14 |

| | | |
|---|---|---|
| GOPC | ENSP00000357484 | GOPC, CAL, Golgi-associated PDZ- and coiled-coil motif-containing protein, CFTR associated ligand, PIST, Fused in Glioblastoma, Ca-L, Golgi-associated PDZ and coiled-coil motif-containing protein, Golgi-associated PDZ- and coiled-coil motif containing protein, CFTR-associated ligand, Golgi-associated PDZ- and coiled-coil motif-containing |
| MKI67 | ENSP00000357643 | MKi67, MIB1, Mib-1, MIB- 1, MIB 1, MKI-67, marker of proliferation Ki67, proliferation marker protein Ki-67, marker of proliferation Ki-67, antigen identified by monoclonal antibody Ki-67 |
| S100A4 | ENSP00000357705 | fibroblast-specific protein-1, mts1, pEL-98, Capl, MTS-1, placental calcium binding protein, S100A4, protein S100A4, p9Ka, protein Mts1, fibroblast-specific protein 1, metastasin, S100 calcium-binding protein A4, fibroblast specific protein-1, pEL98, calvasculin, placental calcium-binding protein, 18a2, S100 calcium binding protein A4, H-MTS1, MTs 1, hS100A4, fibroblast specific protein 1, S-100A4 |
| S100A8 | ENSP00000357722 | CAGA, MRP 8, MRP-8, MIF, calgranulin A, MRP8, hp8, migration inhibitory factor-related protein 8, cystic fibrosis antigen, hMRP8, CFAg, cp10, S100 calcium-binding protein A8, protein S100A8, Cag A, cag-A, S100 calcium binding protein A8, calgranulin-A, CAG_A, h-S100A8, C-A-G-A, protein S100 A8, migration-inhibitory factor-related protein-8 |
| S100A9 | ENSP00000357727 | p14, MIF, MRP-14, MRP 14, calgranulin B, Mrp14, MAC387, MAC 387, Migration inhibitory factor-related protein 14, calgranulin-B, S100 calcium-binding protein A9, HP-14, S100 calcium binding protein A9, Mac-387, p 14, protein S100A9, HP14 |
| LORICRIN | ENSP00000357731 | loricrin, LOR |
| UROS | ENSP00000357787 | URO-S, UROS, uroporphyrinogen-III-synthase, UROIIIS, EC 4.2.1.75, uroporphyrinogen III synthase, uroporphyrinogen-III synthase, uroporphyrinogen III cosynthase |
| PSMD4 | ENSP00000357879 | Rpn10, AS-F, antisecretory factor-1, 2kd-E, PSMD4 |
| AMD1 | ENSP00000357880 | AMD1, AdoMetDC, S-adenosylmethionine decarboxylase 1, 3EpA, 3EpB, AdoMet-DC, adenosylmethionine decarboxylase 1, 3 EPA |
| ANXA9 | ENSP00000357943 | annexins, annexin A9, Annexin 31, ANX31, ANX3-1, ANXA9 |
| NR2E1 | ENSP00000357979 | TLX, NR2E1, Tll, Nuclear receptor TLX, T-LL, nuclear receptor subfamily 2, group E, member 1, Nuclear receptor subfamily 2 group E member 1 |

| | | |
|---|---|---|
| H2AC18 | ENSP00000358155 | H2A, H-2a, H2A.2, H2-A |
| FAM72B | ENSP00000358397 | p17 |
| UBL4A | ENSP00000358674 | G6PD, G-6PD, GdX, G-6-PD, Ubl4A, DXS254E, G6 PD, ubiquitin-like protein 4A, G6PD p |
| TMIGD3 | ENSP00000358730 | adenosine receptors, A3AR, ADORA3, adenosine A3 receptor, A3 AR, A3-AR, adenosine-A3 receptor, TMIGD3 |
| SLC16A4 | ENSP00000358794 | MCT4, SLC16A4, MCT5, Monocarboxylate transporter 4, MCT-4, monocarboxylate transporter-4 |
| ABCA4 | ENSP00000359245 | ABCA4, STGD1, rim ABC transporter, RMP, ABCA 4, ATP-binding cassette sub-family A member 4, ABCA4 p, ATP-binding cassette, sub-family A, member 4, ATP binding cassette subfamily A, ATP binding cassette subfamily A member 4, ATP-binding cassette subfamily A, Retinal-specific ATP-binding cassette transporter |
| SCD | ENSP00000359380 | SCD1, stearoyl-CoA desaturase, hSCD1, delta 9 desaturase, Scd, fatty acid desaturases, fatty acid desaturase, Delta(9) desaturase, Delta-9 desaturase, stearoyl CoA desaturase, SCD-1, delta9-desaturase, Delta9 desaturase, stearoyl-CoA-desaturase, stearoyl-CoA- desaturase, SCD- 1, Stearoyl Co A desaturase |
| CHUK | ENSP00000359424 | IKKalpha, IKK1, IKK-alpha, Ikk alpha, CHUK, IKKA, conserved helix-loop-helix ubiquitous kinase, IKK-1, IkappaB kinase-1, IkappaB kinase 1, inhibitor of nuclear factor kappa-B kinase subunit alpha |
| GBP1 | ENSP00000359504 | GBP1, hGBP1, guanylate-binding protein 1, GBP-1, guanylate binding protein-1, guanylate binding protein 1, guanylatebinding protein-1, guanylate-binding protein-1, guanine nucleotide-binding protein 1, hGBP-1, GTP-binding protein 1 |
| NTSR1 | ENSP00000359532 | NTR, NTSR1, neurotensin receptors, neurotensin receptor 1, NT-R, neurotensin receptor-1, neurotensin receptor type 1, NTSR-1, neurotensin receptor1 |
| BMP5 | ENSP00000359866 | Bmp5, bone morphogenetic proteins, BMP-5, Bone morphogenetic protein-5, bone morphogenetic protein 5, bone morphogenetic proteins, Bmp |
| SRSF11 | ENSP00000359988 | p54, SRSF11, SFRS11 |
| CYP2C8 | ENSP00000360317 | CYP2C8, Cytochrome P450 2C8, s-mephenytoin 4-hydroxylase, EC 1.14.14.1, CYP 2C8, cytochrome p450 family 2 subfamily C member 8 |

| | | |
|---|---|---|
| CYP2C19 | ENSP00000360372 | CYP2C, cytochrome P450 2C19, CYP2C19, P450C2C, Cyp-2c, CYP 2C, cytochrome P4502C19, CYP 2C19, cytochrome P450, family 2, subfamily C, polypeptide 19, cytochrome P450, family2, subfamily C, polypeptide19, CYP-2C19, cytochrome P450 family 2 subfamily C member 19 |
| KCNG1 | ENSP00000360626 | KH2, k13, Kv6.1, K1-3, Hkh2, HKh-2, hK13 |
| SLC25A5 | ENSP00000360671 | ht2, aac2, SLC25A5, 2F1, ANT2, HT-2, HT-3, HT 3, ht3, adenine nucleotide translocator-2, T_2, Aac2p, T3p, adenine-nucleotide translocator-2, ANT-2 |
| TXNDC12 | ENSP00000360688 | ERp18, hTLP19, Ag1, ERp19, Ag-1, AGR-1, HAG-1, AG -1, Ag 1, agr1, TXNDC12 |
| KCNB1 | ENSP00000360806 | Kv2.1, KCNB1, DRK1, Potassium voltage-gated channel subfamily B member 1, KV 2.1 |
| IFIT2 | ENSP00000360891 | p54, IFI54, IFIT2, ISG 54, IFI-54K, ISG54K, ISG54, GARG39, interferon-induced protein with tetratricopeptide repeats 2, ISG-54 |
| AKR1A1 | ENSP00000361140 | DD3, ALR, aldehyde reductase, AKR1A1, aldo-keto reductases, aldoketoreductases, DD 3, DD-3, aldoketo reductases |
| SFTPA2 | ENSP00000361400 | PSAP, HspA2, SP-A2, SFTPA2, SP-A II, collectins, PSP-A, SFTP1, Sftpa, surfactant protein A2, Sftp-1, PspA, PSA P, surfactant protein-A2, SPA2, hSP-A2, P-SAP, sPA, Spa2p |
| CDC20 | ENSP00000361540 | Cdc20, p55CDC, hCDC20, cell division cycle 20, CDC-20, 5K-Hu, cell division cycle 20 homolog, cell-division cycle 20 |
| MPL | ENSP00000361548 | TPOR, thrombopoietin receptor, c-mpl, Mpl, proto-oncogene c-mpl, cMpl, thrombopoietin-receptor, myeloproliferative leukemia virus oncogene, TPO-R, CD110, myeloproliferative leukemia protein |
| ESX1 | ENSP00000361669 | HESX1, Esx1, ESXR1, ESX1L, ESX-1, HESX-1 |
| BMP8B | ENSP00000361915 | bone morphogenetic proteins, hop2, OP-2, BMP8B, Osteogenic protein-2, Bmp8, BMP-8, bone morphogenetic proteins, BMP8A, OP2, Bmp, Bone Morphogenetic Protein-8B |
| ANXA7 | ENSP00000362010 | ANX7, annexin A7, synexin, annexin VII, Annexin 7, annexins, Anxa7, Annexin-A7, annexin7, AnnexinA7 |
| DNM1 | ENSP00000362014 | dynamin 1, Dynamin-1, DNM1, Dnm1p, 1 dyn, 2 dyn, dynamin1 |
| CDCA8 | ENSP00000362146 | Borealin, Dasra B, CDCA8, cell division cycle associated 8 |

| | | |
|---|---|---|
| ATRX | ENSP00000362441 | RAD54, MRX52, ATRX, hRad54, X-linked nuclear protein, ATR-X, XNP, transcriptional regulator ATRX, RAD54L, ATR2, alpha thalassemia/mental retardation syndrome X-linked, Rad 54, rad-54, alpha-thalassemia/mental retardation syndrome X-linked |
| RNF8 | ENSP00000362578 | RNF8, Ring finger protein 8 |
| MTCH1 | ENSP00000362730 | PSAP, presenilin-associated protein, PSA P, mitochondrial carrier 1, P-SAP, Mtch1 |
| PTAFR | ENSP00000362965 | PAFR, PAF-R, platelet-activating factor receptor, PTAFR, platelet-activating-factor receptor, platelet activating factor receptor, platelet-activating factor-receptor, platelet- activating factor receptor |
| FGR | ENSP00000363117 | fgr, c-fgr, SRC2, c-src2, SRC-2, Src family tyrosine kinases, fg R, Gardner-Rasheed feline sarcoma viral oncogene homolog, Src-family tyrosine kinases, Gardner-Rasheed feline sarcoma viral (v-fgr) oncogene homolog, proto-oncogene c-fgr, tyrosine-protein kinase Fgr, p55c-fgr |
| MED12 | ENSP00000363193 | OPA1, FGS1, MED12, OPA1 p, TRAP230, KIAA0192, mediator complex subunit 12, mediator of RNA polymerase II transcription subunit 12, OPA-1, HopA, MED12 p, MED 12 |
| POLE3 | ENSP00000363286 | p17, EC 2.7.7.7, YBL1, POLE3, CHRAC17, poly-merase, poly merase |
| DVL2 | ENSP00000005340 | DVL2, Dishevelled 2, Dishevelled-2, Dishevelled2, Dvl-2, Dvl 2, dishevelled segment polarity protein 2 |
| SKAP2 | ENSP00000005587 | SKAP55R, RA70, SKAP-HOM, Saps, SKAP2, SCAP2, PRAP, Src kinase-associated phosphoprotein2, Src kinase-associated phosphoprotein 2, src kinase associated phosphoprotein 2 |
| CX3CL1 | ENSP00000006053 | NTN, Abcd3, fractalkine, FKN, CX3CL1, neurotactin, SCYD1, chemokine ligand 1, CXC3, Fractal-kine, chemokine (C-X3-C motif) ligand 1, NT-N, chemokine-ligand-1, C-X3-C motif chemokine ligand 1 |
| OTC | ENSP00000039007 | ornithine transcarbamylase, EC 2.1.3.3, Otc, ornithinetranscarbamylase |
| GNA11 | ENSP00000078429 | guanine nucleotide binding protein, GNBP, guanine-nucleotide binding protein, guanine nucleotide-binding protein, Gna11, guanine-nucleotide-binding protein, FHH2, FHH-2, G-protein subunit alpha-11, G-protein subunit alpha11, G protein subunit alpha 11 |
| CAMKK1 | ENSP00000158166 | CAMKK1, calcium/calmodulin-dependent protein kinase kinase 1, calcium/calmodulin-dependent protein kinase kinase-1, calcium/calmodulin-dependent protein kinase kinase1 |

| | | |
|---|---|---|
| KIF22 | ENSP00000160827 | KID, KIF22, KNSL4, OBP-1, kinesin-like DNA binding protein, OBP-2, kinesin family member 22, kinesin-like DNA-binding protein, SEMDJL2, OBP, O-BP |
| CA12 | ENSP00000178638 | CA XII, CA12, carbonic anhydrase XII, Carbonic Anhydrase 12, CAXII, EC 4.2.1.1, CA1-2 |
| NDUFB4 | ENSP00000184266 | HB15, NDUFB4, B1-5p |
| TRIP6 | ENSP00000200457 | zyxin family, TRIP6, OIP1, thyroid receptor-interacting protein 6, thyroid receptor interacting protein-6, OIP-1, ZRP-1, zyxin-related protein 1, thyroid receptor interacting protein 6, thyroid hormone receptor interactor 6 |
| SERPIND1 | ENSP00000215727 | HC2, HCII, heparin cofactor II, HC II, HCF-2, HC 2, heparin co-factor II, HC-II, HEP-2 human, Hcf2, Hep2 human, LS-2, HC-2, SERPIND1, HC -2, serpin family D member 1, Serpin D1 |
| RBX1 | ENSP00000216225 | ROC1, Rbx1, RBX-1, Roc-1, Regulator of Cullins-1, 3 DPL, Ring Box Protein-1, ring-box 1, Ring-box1, Rbx 1, RING-box protein 1, RING box protein1, ring box-1, Ring box protein 1 |
| ACO2 | ENSP00000216254 | mitochondrial aconitase, ACO2, aconitase 2, ACONM, Aconitase2, ACO-2, aconitase-2 |
| EFS | ENSP00000216733 | Efs, Efs2, Efs1, hEfs1, Embryonal Fyn-associated substrate, hEfs2, CASS3, cas3 |
| PCK2 | ENSP00000216780 | PEPCK, PCK2, EC 4.1.1.32, phosphoenolpyruvate carboxykinase 2, hPCK2, PEPCK-M |
| LBP | ENSP00000217407 | lipopolysaccharide binding protein, Lipopolysaccharide-binding Protein, lbp, Lipopolysaccharide-binding-protein |
| STS | ENSP00000217961 | Asc, steroid sulfatase, EC 3.1.6.2, arylsulfatase C, AS-C, steroidsulfatase, steryl-sulfatase, steryl-sulfate sulfohydrolase, steroid-sulfatase, ARSC, AS c |
| F9 | ENSP00000218099 | factor IX, Plasma thromboplastin component, fIX, coagulation factor IX, Christmas factor, ptc, F IX, coagulation factor-IX, factor-IX, p-TC, factorix, FI x |
| CBLN1 | ENSP00000219197 | Cbln1, precerebellin, cerebellin-1 |
| CCL17 | ENSP00000219244 | ABCD2, CCL17, TARC, Thymus and activation-regulated chemokine, SCYA17, thymus- and activation-regulated chemokine, CC chemokine TARC, thymus and activation regulated chemokine, C-C chemokine TARC, chemokine ligand 17, chemokine (c-c motif) ligand 17, CCL-17, C-C motif chemokine ligand 17, C-C motif chemokine 17, Ccl 17 |
| PDIA2 | ENSP00000219406 | PDI, PDIp, EC 5.3.4.1, protein disulfide isomerase A2, PDIR, protein disulfide isomerase,  pancreatic, PDIA2, PD-I |
| MEFV | ENSP00000219596 | MEFV, pyrin, marenostrin, TRIM20, mef |

| | | |
|---|---|---|
| RIPK2 | ENSP00000220751 | RICK, RIP2, Cck, RIPK-2, CARDIAK, RIPK2, Receptor interacting protein-2, receptor-interacting protein 2, receptor-interacting serine-threonine kinase-2, receptor-interacting serine/threonine kinase 2, receptor interacting protein 2, receptor-interacting serine/threonine protein kinase-2, receptor interacting serine/threonine-protein kinase 2, receptor-interacting serine-threonine kinase 2, C-CK, receptor-interacting serine/threonine-protein kinase 2, RIP-2, receptor interacting serine/threonine kinase 2, receptorinteracting protein 2, Receptor interacting-serine/threonine-protein kinase 2, CARD3, 5AR2 |
| AMH | ENSP00000221496 | AMH, MIF, anti-Mullerian hormone, anti Mullerian hormone, Antimullerian hormone, Anti-Muellerian Hormone, anti-mullerian-hormone, anti-Mul-lerian hormone |
| CEACAM5 | ENSP00000221992 | carcinoembryonic antigen, CEACAM5, carcino-embryonic-antigen, carcino-embryonic antigen, CD66e, carcinoembryonic-antigen, CEA CAM5, carcinoembryonic antigen-related cell adhesion molecule 5, carcino embryonic antigen, carci noembryonic antigen, carcino-embryonic antigen |
| CDC37 | ENSP00000222005 | Cdc37, p50Cdc37, cell division cycle 37, cell division cycle 37 homolog |
| RABAC1 | ENSP00000222008 | PRA1, Yip3, prenylated Rab acceptor 1, PRAF1, P-Raf-1, p-Raf1, PR AF1, RABAC1 |
| TFPI2 | ENSP00000222543 | Ref-1, PP5, Ref 1, placental protein 5, ref1, tissue factor pathway inhibitor-2, TFPI-2, tissue factor pathway inhibitor 2, TFPI2 |
| CAV2 | ENSP00000222693 | Caveolin-2, caveolin 2, Cav-2, CAV2 |
| STX1A | ENSP00000222812 | HPC1, syntaxin 1A, syntaxins, STX1A, HPC-1, STx1, syntaxin-1A, Stx 1, Stx-1, Syntaxin1A, Syn-1A, HsTX1, syn1a, Stx-1A |
| PCOLCE | ENSP00000223061 | PCOLCE, type I procollagen COOH-terminal proteinase enhancer, procollagen C-endopeptidase enhancer 1, Procollagen C-Endopeptidase Enhancer, P-cpe, procollagen C-proteinase enhancer 1, PCPE-1, procollagen C-proteinase enhancer-1 |
| FIS1 | ENSP00000223136 | hFis1, Fis1, Fis1p, Fis-1, fission 1, fission-1, mitochondrial fission 1 protein, fission,  mitochondrial 1 |
| CYP26A1 | ENSP00000224356 | Cyp26a1, Cyp26, hP450RAI, P450RAI, P450RAI-1, Retinoic acid 4-hydroxylase, cp26, cytochrome P450 26A1, retinoic acid-4-hydroxylase, cytochrome P450 family 26, CYP 26A1 |

| | | |
|---|---|---|
| VTN | ENSP00000226218 | vitronectin, somatomedin-B, complement S-protein, S-protein, somatomedin B, s protein, serum spreading factor, serum-spreading factor, VTN |
| SPI1 | ENSP00000227163 | SPI1, transcription factor PU.1, PU.1, Spi-1, Sfpi1, Sfpi-1, hematopoietic transcription factor PU.1, spleen focus forming virus proviral integration oncogene, SPI - 1 |
| CD69 | ENSP00000228434 | CD69, CD69 antigen, he-A1, CLEC2C, CD69 molecule, AIm, 3 h up |
| TPI1 | ENSP00000229270 | triosephosphate isomerase, TIM, EC 5.3.1.1, triose phosphate isomerase, TPI 1, Tpi-1, triose-phosphate isomerase, TPI1, tpi, triosephosphate isomerase-1, triose phosphate isomerase 1, triosephosphate-isomerase 1, Triosephosphate isomerase 1, TP I |
| IL5 | ENSP00000231454 | Trf, interleukin 5, IL-5, interleukin-5, IL5, eosinophil differentiation factor, T-cell replacing factor, T-cell-replacing factor, EDF, IL 5, hIL5, T cell-replacing factor |
| MLH1 | ENSP00000231790 | MLH1, hMLH1, h MLH1, MutL homologs, mutL, DNA mismatch repair protein MLH1, hMLH1-p, HN-PCC, MLH-1, hMLH-1, MutL homolog 1, Mlh1p, mutL homolog 1, colon cancer, nonpolyposis type 2, Mut-L homolog 1, MutL-homolog 1, mut L homolog 1, DNA mismatch-repair protein Mlh1, MLH1 p, MutL homolog-1, MutL protein homolog 1, MutL homolog1 |
| SF3B6 | ENSP00000233468 | p14, SF3b complex, HP-14, SF3B14, p 14, SF3B6, HP14 |
| SLC9A2 | ENSP00000233969 | NHE-2, SLC9A2, NHE2 |
| AZU1 | ENSP00000233997 | heparin-binding protein, azurocidin, neutrophil azurocidin, CAP37, HBP, heparin binding protein, hb P, AZU1, azu, Azurocidin 1, AZU-1 |
| ID2 | ENSP00000234091 | Id2, Id-2, Id-2H, inhibitor of DNA binding 2, inhibitor of differentiation-2, Inhibitor of differentiation 2, Inhibitor of DNA-binding 2, ID2a, I-D2 |
| NT5C1A | ENSP00000235628 | CN-1, cN-I, HCN1, CN1, 5'-nucleotidases, HCN-1A, EC 3.1.3.5, Cytosolic 5'-nucleotidase 1A, NT5C1A, cN1A, HCN1A, 5'-nucleotidases, cN-1A, cytosolic 5'nucleotidase 1A |
| AKR7A2 | ENSP00000235835 | AFAR, aldo-keto reductases, aldoketoreductases, AKR7A2, AKR7, aldo-keto reductase 7, 2 bp-1, Afar1, Succinic semialdehyde reductase, 2 BP1, aldoketo reductases, aflatoxin aldehyde reductase |
| PKD2 | ENSP00000237596 | polycystin-2, Pc2, HPC2, TRPP2, polycystin 2, Pc 2, PC-2, polycystwin, R48321, PKD4, Hpc-2, PKD2 p, hPKD2, polycystin2 |

| | | |
|---|---|---|
| IAPP | ENSP00000240652 | amylin, IAPP, islet amyloid polypeptide, diabetes-associated peptide, islet-amyloid polypeptide, Iap, I-AP |
| TFG | ENSP00000240851 | TFG, TRK-T3, TRK-fused gene, HMSN P, TRK fused gene |
| AKR1D1 | ENSP00000242375 | SRD5B1, AKR1D1, aldoketoreductases, aldo-keto reductase family 1, member D1, delta(4)-3-oxosteroid 5beta-reductase, aldo-keto reductases, Delta(4)-3-oxosteroid-5beta-reductase, 3 CAs, aldoketo reductases, Aldo-keto reductase family 1 member D1 |
| ACADS | ENSP00000242592 | ACADs, butyryl-CoA dehydrogenase, acyl-CoA dehydrogenase family, butyryl CoA dehydrogenase, Acyl-CoA dehydrogenase, C-2 to C-3 short chain |
| MC3R | ENSP00000243911 | Mc3r, melanocortin receptor 3, Melanocortin-3 receptor, Melanocortin 3 receptor, MC3, MC3-R, melanocortin-3-receptor, hMC3-R, melanocortin receptor-3, HMC3, hMC3R |
| PI3 | ENSP00000243924 | elafin, PI-3, PI3, SKALP, Trappin-2, elastase-specific inhibitor, Skin-derived antileukoproteinase, peptidase inhibitor 3 |
| MT2A | ENSP00000245185 | HMT2, metallothionein II, MT2A, CES1, metallothionein-II, MT II, metallothionein 2A, MT-II, ces-1, MT-2A, MT 2A, metallothionein-2, metallothionein 2, Metallothionein-2A, MT-2 human, hCES1, hmt-2 |
| VASP | ENSP00000245932 | VASP, vasodilator-stimulated phosphoprotein, vasodilator stimulated phosphoprotein, Vasodilator-Stimulated-Phosphoprotein |
| RALY | ENSP00000246194 | RNA-binding protein, RALY, RNA binding protein, RBP, p542, RNA binding protein RALY, autoantigen p542, RNA binding-protein, RNA-binding-protein |
| UROD | ENSP00000246337 | URO-D, upd, uroporphyrinogen decarboxylase, UROD, P-CT, PC-T, Pct, EC 4.1.1.37 |
| KRT13 | ENSP00000246635 | KRT13, keratin 13, cytokeratin 13, k13, K1-3, CK13, hK13, keratin-13, CK-13, CK 13 |
| CSN1S1 | ENSP00000246891 | alpha s1-casein, CSN1S1, CSN1, Alpha-s1 Casein |
| RAC2 | ENSP00000249071 | small G protein, Rac2, Rho family GTPases, Rho-family GTPases, p21rac2, small G-protein, Ras-related C3 botulinum toxin substrate 2, Rac-2, RAC family small GTPase 2 |
| CD68 | ENSP00000250092 | CD68, gp 110, macrosialin, CD68 molecule, CD68 antigen, CD-68, gp110, CD 68, LAMP4 |

| | | |
|---|---|---|
| FOXA1 | ENSP00000250448 | Foxa1, hepatocyte nuclear factor 3alpha, hepatocyte nuclear factor 3 alpha, hepatocyte nuclear factor 3-alpha, HNF3A, hepatocyte nuclear factor-3alpha, Foxa-1, Forkhead box A1, Hepatocyte nuclear factor-3 alpha, transcription factor 3a, Forkhead box protein A1, Forkhead-box protein A1, Fork-head box protein A1, forkhead-box A1, HNF-3a, Fork head box protein A1, FOX-A1, tcf3a |
| UGT2A3 | ENSP00000251566 | UGT2A3 |
| KRT6B | ENSP00000252252 | PC-2, PC2, HPC2, K6b, PC4, KRT6B, CK6B, HPC4, Pc 2, keratin 6b, Pc 4, Hpc-2, HPC-4, PC-4 |
| BMP15 | ENSP00000252677 | GDF-9B, BMP15, BMP-15, bone morphogenetic proteins, bone morphogenetic protein-15, bone morphogenetic protein 15, GDF9B, bone morphogenetic proteins, Bmp |
| ABCB7 | ENSP00000253577 | Atm1p, ABC7, Abcb7, hABC7, ATP-binding cassette subfamily B member-7, ATP-binding cassette transporter 7, ATP-binding cassette subfamily B, AsAT |
| RARA | ENSP00000254066 | RARA, RAR, Rar-a, retinoic acid receptor-alpha, retinoic acid receptor alpha, retinoic acid receptor alpha, retinoic acid receptor, alpha, retinoic acid receptor-a, r-AR |
| PPP1R1B | ENSP00000254079 | DARPP-32, dopamine and cAMP-regulated phosphoprotein, PPP1R1B, DARPP32, Dopamine- and cAMP-regulated phosphoprotein, AY070271, dopamine and cAMP-regulated phospho-protein, protein phosphatase 1 regulatory subunit 1B, Dopamine-and-cAMP-regulated neuronal phosphoprotein, Dopamine and cAMP regulated phosphoprotein, dopamine- and cAMP-regulated phosphoprotein, dopamine and c-AMP regulated phosphoprotein, dopamine- and cAMP-regulated neuronal phosphoprotein |
| LGALS3 | ENSP00000254301 | Galectin-3, gal3, Galectin 3, laminin-binding protein, Gal-3, galactoside-binding protein, Lgals3, galig, Mac-2, CBP35, hMac-2, LGALS2, galactoside binding protein, IgE-binding protein, laminin binding protein, hgal-3, Mac2, Mac-2 antigen, gal-3P, galectin3, L31P, LGALS-3, GAL 3, galactose-specific lectin 3, carbohydrate binding protein 35, IgE binding protein |
| HSD17B7 | ENSP00000254521 | HSD17B7, 3-ketosteroid reductase, Hydroxysteroid (17beta) dehydrogenase 7, PRAP |
| ALDH3B2 | ENSP00000255084 | ALDH8, aldehyde dehydrogenases, ALDH3B2 |
| CCNA1 | ENSP00000255465 | cyclin A1, Ccna1, cyclinA1, Cyclin-A1, cyclin A 1 |

| | | |
|---|---|---|
| FABP4 | ENSP00000256104 | aP2, AP-2, adipocyte fatty acid-binding protein, adipocyte lipid-binding protein, fatty acid-binding protein-4, FABP 4, FABP4, ALBP, adipocyte fatty acid binding protein, adipocyte lipid binding protein, aFABP, HAP2, Adipocyte fatty-acid-binding protein, fatty acid-binding protein 4, AP 2, adipocyte-type fatty acid binding protein, A-FABP, adipocyte type fatty acid binding protein, fatty acid binding protein-4, Hap 2, fatty acid binding protein 4, adipocyte, fatty acid binding protein 4, adipocyte-fatty acid binding protein, adipocyte-type fatty acid-binding protein, Adipocyte-fatty acid-binding protein, Fatty-acid binding protein 4, FABP-4, fatty acid binding protein family, fatty acid-binding protein, adipocyte, Fatty Acid Binding Protein4, Adipocyte Fatty-Acid Binding Protein, adipocyte-fatty-acid-binding protein, Fatty-acid-binding protein 4, fatty-acid binding protein-4, fatty acid-binding protein family |
| RRAS2 | ENSP00000256196 | TC21, RRAS2, R-Ras2, related RAS viral oncogene homolog 2, Ras-related protein R-Ras2 |
| MBD2 | ENSP00000256429 | MBD2, methyl CpG binding domain protein-2, NuRD complex, methyl-CpG-binding protein MBD2, methyl-CpG binding protein MBD2, dMTase, Methyl-CpG binding domain protein 2, methyl CpG-binding domain-containing, Methyl-CpG-binding domain protein-2, Mbd-2, methyl-CpG-binding domain protein 2, methyl CpG-binding domain protein 2, Demethylase, methyl CpG binding domain protein 2 |
| CHL1 | ENSP00000256509 | NCAM, Chl1, neural cell adhesion molecule, N-CAM, cell adhesion molecule with homology to L1CAM, neural cell-adhesion molecule, neural-cell adhesion molecule, close homolog of L1, CHL-1, neural cell adhesion molecule L1-like protein |
| FST | ENSP00000256759 | follistatin, activin-binding protein, activin binding protein |
| SLCO1B1 | ENSP00000256958 | LST-1, LST1, OATP-C, SLC21A6, OATP2, SLCO1B1, OATP1B1, solute carrier organic anion transporter family member 1B1, OATP-2, OATP 1B1, solute carrier organic anion-transporter family, member 1B1, solute carrier organic anion transporter family, member 1B1, OATPC |
| KRT82 | ENSP00000257974 | HB2, hHb2, HH-B2 |
| GNS | ENSP00000258145 | glucosamine 6-sulfatase, G6S, glucosamine-6-sulfatase, N-acetylglucosamine-6-sulfatase, N-acetylglucosamine 6-sulfatase, EC 3.1.6.14 |

| | | |
|---|---|---|
| BRIP1 | ENSP00000259008 | BACH1, BRIP1, BRCA1-associated C-terminal helicase 1, BRCA1-interacting protein C-terminal helicase 1, BRCA1 interacting protein C-terminal helicase 1, Fanconi anemia group J protein, Bach-1, BRIP-1, BRCA1 Interacting Protein C- terminal helicase 1 |
| BLK | ENSP00000259089 | Src family tyrosine kinases, B-lymphoid tyrosine kinase, blk, Src-family tyrosine kinases, B lymphoid tyrosine kinase, B lymphocyte kinase, B-lymphocyte kinase |
| IL1RN | ENSP00000259206 | IL-1RA, IRAP, interleukin-1 receptor antagonist, IL-1RN, IL1RN, interleukin-1-receptor antagonist, IL1RAP, interleukin 1 receptor antagonist, icIL-1Ra, interleukin-1 receptor antagonist protein, interleukin-1--receptor antagonist, IL1Ra, IL-1 ra, IL-1-ra, IL 1ra, hIL-1Ra, intracellular interleukin-1 receptor antagonist, IL-1 inhibitor, IL- 1Ra, interleukin -1 receptor antagonist, IL1-RA, IL-1 RN, IL1-RN, interleukin-1 receptor-antagonist, Interleukin- 1 receptor antagonist, ic-IL-1ra, interleukin 1-receptor antagonist, inter-leukin-1 receptor antagonist, hIL1Ra, IL1 RN, IL-1RAP, IL-1Ra 3, interleukin-1receptor antagonist, interleukin 1-receptor-antagonist protein |
| CCN3 | ENSP00000259526 | NOVH, CCN3, Nephroblastoma overexpressed, nephroblastoma overexpressed gene, hccn3, nephroblastoma over-expressed gene, nephroblastoma over-expressed, Nephroblastoma-overexpressed, nephroblastoma-overexpressed gene, N ephroblastoma overexpressed |
| CCL27 | ENSP00000259631 | CTACK, ESkine, CCL27, ILC, cutaneous T cell-attracting chemokine, IL-11Ralpha-locus chemokine, cutaneous T-cell-attracting chemokine, CC chemokine ILC, cutaneous T cell- attracting chemokine, c-TAK, AlP, chemokine (C-C motif) ligand 27, A-LP, chemokine ligand 27 |
| KIF11 | ENSP00000260731 | Eg5, KIF11, Knsl1, 2 GM1, kinesin family member 11, kinesin-like 1, kinesin-like-1, Eg-5, Kinesin-like protein KIF11, KIF-11, hEg-5, 3ZCW |
| MSTN | ENSP00000260950 | Gdf8, Myostatin, MSTN, GDF-8, Growth differentiation factor-8, growth differentiation factor 8, growth/differentiation factor-8 |
| MIB1 | ENSP00000261537 | DIP-1, Mib1, Mib-1, mindbomb homolog 1, MIB- 1, MIB 1, Mindbomb E3 ubiquitin protein ligase 1, E3 ubiquitin-protein ligase mib1, Dip1 |
| THOC1 | ENSP00000261600 | Hpr1, hHpr1, Thoc1, Hpr1p, hpr-1, p84N5, p84, hTREX84, HP-R1, THO complex 1 |

| | | |
|---|---|---|
| USP14 | ENSP00000261601 | T-G-T, USP14, ubiquitin-specific protease 14, Ubiquitin-specific protease-14, tg T, ubiquitin-specific peptidase 14, ubiquitin specific peptidase 14, tgT |
| FAT2 | ENSP00000261800 | FAT2, hFat2, MEGF1, FAT-2, protocadherin fat 2 |
| SLC30A4 | ENSP00000261867 | SLC30A4, hZnT4, ZNT4, ZnT-4 |
| SEMA7A | ENSP00000261918 | CDw108, semaphorins, Sema7A, semaphorin K1, Semaphorin 7A, sema K1, H-Sema-L, Sema-K1, SEMAL, CD108, JMH blood group antigen, Semaphorin7A, semaphorin-7A, SEMA-7A |
| MSR1 | ENSP00000262101 | MSR1, macrophage scavenger receptor 1, SR-A, SCARA1, macrophage scavenger receptor types I and II, SR-AI, SR-AII, sRA, macrophage scavenger receptor-1, CD204, SRA-I, scavenger receptor class A member 1 |
| ANXA13 | ENSP00000262219 | intestine-specific annexin, annexins, ANXA13, ANX13, annexin XIII, ANXA1 3, Annexin A13 |
| YY1 | ENSP00000262238 | Yin Yang 1, YY1, UCRBP, Yin Yang-1, YY1 transcription factor, NF-E1, YY-1, NFE1, yin and yang 1, Yin-Yang 1, YY-1 transcription factor, Yin Yang1, Yin-Yang-1, Yinyang1, NFE-1 |
| AXIN1 | ENSP00000262320 | AXIN1, Axin, Axin 1, Axin-1, axis inhibition protein 1, axis inhibition protein1 |
| CREBBP | ENSP00000262367 | CREB-binding protein, CREB binding protein, CREBBP, Cbp, c BP, RSTS1 |
| ALG1 | ENSP00000262374 | Mt1, MAT1, ALG1, hALG1, MT-1, ALG-1, beta1, 4 mannosyltransferase, HMT-1, HMAT1, Hmat-1, GDP-Man:GlcNAc2-PP-dolichol mannosyltransferase, beta 1, 4-mannosyltransferase, mannosyltransferase-1, hMT1, Mat 1, chitobiosyldiphosphodolichol beta-mannosyltransferase |
| N4BP1 | ENSP00000262384 | N4BP1, NEDD4-binding protein 1 |
| GNAO1 | ENSP00000262494 | Galphao, GNAO1, G alpha o, Galpha-o, guanine nucleotide-binding protein G(o) subunit alpha |
| PCSK2 | ENSP00000262545 | PC2, HPC2, PC-2, NEC-2, NEC2, PCSK2, SPC2, prohormone convertase 2, neuroendocrine convertase-2, Pc 2, Spc2p, proprotein convertase subtilisin/kexin type 2, Hpc-2, proprotein convertase subtilisin/kexin-type 2, prohormone convertase-2, proprotein convertase 2 |
| MYDGF | ENSP00000262947 | SF20, IL-25, chromosome 19 open reading frame 10, IL25, c19orf10, interleukin-25, Interleukin 25, Myeloid-derived growth factor, MYDGF |
| GNA15 | ENSP00000262958 | guanine nucleotide binding protein, guanine nucleotide-binding protein, Gna15, guanine-nucleotide-binding protein, guanine-nucleotide binding protein, GNBP |

| | | |
|---|---|---|
| SLC17A6 | ENSP00000263160 | DNPI, VGLUT2, SLC17A6, hVGLUT2, VGLUT-2, vesicular glutamate transporter 2, vesicular glutamate transporter-2, vesicular glutamate transporter2 |
| LY9 | ENSP00000263285 | Ly-9, CD229, Ly9, SLAMF3 |
| PRKCG | ENSP00000263431 | SCA14, PKC gamma, PRKCG, protein kinase C gamma, PKCgamma, protein kinase Cgamma, SCA-14, PKCG, PKC-gamma, Protein kinase C-gamma, SCA 14, PKC-G, Protein kinase C gamma type |
| PNO1 | ENSP00000263657 | RNA-binding protein, RNA binding protein, PNO1, RBP, hPno1, RNA binding-protein, RNA-binding protein PNO1, RNA-binding-protein |
| SELP | ENSP00000263686 | P-selectin, granule membrane protein-140, CD62, selectin P, GMP-140, P selectin, CD62P, P-sel, SELP, granule membrane protein 140, granule-membrane protein 140, PADGEM, CD-62, CD 62, PSEL, selectin-P, selectin P (granule membrane protein 140 kDa, antigen CD62, CD62 P, 1 G1-S, CD62-P, GMP140 |
| GRB14 | ENSP00000263915 | Grb14, GRB-14, growth factor receptor-bound protein 14 |
| ARCN1 | ENSP00000264028 | ARCN1, archain, coatomer subunit delta, COPD human, SR-MMD |
| CBL | ENSP00000264033 | c-cbl, Cbl, Cbl proto-oncogene, Cbl-2, CBL2, FRA11B, 2 cbl, oncogene CBL2, CBL2 p, proto-oncogene c-Cbl, Casitas B-lineage lymphoma proto-oncogene, 4a4b, E3 ubiquitin-protein ligase Cbl, 4a-4c, C-bl |
| CUL3 | ENSP00000264414 | Cul3, cullin 3, CUL-3, Cullin3, Cullin-3, CUL 3 |
| NNT | ENSP00000264663 | nicotinamide nucleotide transhydrogenase, Nnt |
| KLKB1 | ENSP00000264690 | PPK, KLKB1, plasma kallikrein, plasma prekallikrein, kallikreins, Fletcher factor, PKK, Klk3, KLK 3, hKLK3, p-KK, kallikrein B1 |
| TP63 | ENSP00000264731 | P5-1, p63, TP63, tumor protein p63, p51, p73L, tumor protein 63, TP73L, chronic ulcerative stomatitis protein, CUSP, A-Is, transformation related protein 63, TP53L, Transformation-related protein 63, HP40, AI s |
| ANXA3 | ENSP00000264908 | lipocortin III, ANX3, annexin III, annexins, inositol 1, 2-cyclic phosphate 2-phosphohydrolase, placental anticoagulant protein III, ANXA3, annexin A3, 1 AII, Annexin 3 |
| SUB1 | ENSP00000265073 | p15, p14, PC4, HPC4, 2 Phe, Pc 4, SUB1, HP-14, HPC-4, p 14, SUB1 homolog, positive cofactor 4, HP14, p 15 |
| PAPSS1 | ENSP00000265174 | PAPSS1, PAPSS, EC 2.7.1.25, SK1, EC 2.7.7.4, Hsk1, SK-1, 3'-Phosphoadenosine 5'-phosphosulfate synthase 1 |

| | | |
|---|---|---|
| SRF | ENSP00000265354 | serum response factor, serum-response factor, Srf, MCM1, serum-response-factor |
| PON3 | ENSP00000265627 | PON3, paraoxonases, paraoxonase-3, Paraoxonase 3, hPON3, EC 3.1.1.2, serum paraoxonase/lactonase 3, PON-3 |
| CHKA | ENSP00000265689 | choline kinase, Chka, cki, c-Ki, Ethanolamine kinase, CHK, choline kinase alpha, 2cKO, CKA, Choline kinase-alpha, ch-K |
| CDK6 | ENSP00000265734 | CDK6, cell division protein kinase 6, Cyclin-dependent kinase-6, cyclin-dependent kinase 6, CDK 6, PLSTIRE, cyclin dependent kinase 6, cyclindependent kinase 6 |
| MAPKAP1 | ENSP00000265960 | MIP1, MIP-1, Mip1p, sin1, MAPKAP1, mTOR complex 2, hMIP-1, MAPK-AP-1, mTor-complex-2, mitogen-activated protein kinase associated protein 1, mTOR Complex-2, MAPK-AP1, Mitogen-activated protein kinase-associated protein 1 |
| IDE | ENSP00000265986 | Insulin-degrading enzyme, IDE, insulysin, EC 3.4.24.56, insulin degrading enzyme, Abeta-degrading protease |
| SLC5A4 | ENSP00000266086 | SGLT2, hSGLT3, SGLT3, SGLT 2, SLC5A4, SGLT-2, SGLT- 2, hSGLT2, SGLT-3, SAAT1 |
| CBLN3 | ENSP00000267406 | Cbln3, cerebellin-3 |
| SORD | ENSP00000267814 | sorbitol dehydrogenase, SORD, SORD1 |
| CYP11A1 | ENSP00000268053 | P450scc, CYP11A, CYP11A1, cytochrome P-450(SCC, P-450scc, cholesterol desmolase, P450 scc, CYPXIA1, P450-scc, cytochrome P450 11A1, cytochrome P450 family 11 subfamily A member 1 |
| ARHGDIA | ENSP00000269321 | Rho GDIalpha, Rho-GDI, arhgdia, RhoGDI, Rho GDP-dissociation inhibitor-alpha, Rho GDI, GDIA1, RhoGDIalpha, RhoGDI alpha, Rho GDP-dissociation inhibitor 1, Rho-GDI 1, Rho-GDP dissociation inhibitor (GDI) alpha, Rho GDI alpha, Rho-GDP dissociation inhibitor alpha, Rho GDP-dissociation inhibitor alpha, RhoGDI1, Rho GDP dissociation inhibitor alpha, Rho-GDIalpha, RhoGDIA, Rho GDP dissociation inhibitor 1, Rho-GDP dissociation inhibitor 1, Rho GDP-dissociation inhibitor-1 |
| CBX4 | ENSP00000269397 | PC2, HPC2, Pc 2, PC-2, Cbx4, Hpc-2, Chromobox homolog 4, chromobox 4 |
| TINAGL1 | ENSP00000271064 | TIN-ag-RP, Arg-1, Arg1, Lcn7, TINAGL1, Arg 1, Tubulointerstitial nephritis antigen-like 1 |
| PSMA5 | ENSP00000271308 | proteasome zeta chain, proteasome subunit alpha type 5, PSMA5 |
| CELSR2 | ENSP00000271332 | CELSR2, ADGRC2, cadherin EGF LAG seven-pass G-type receptor 2 |

| | | |
|---|---|---|
| SFRP2 | ENSP00000274063 | secreted Frizzled-related proteins, Sfrp2, sarp1, Secreted Frizzled-related protein 2, sFRP-2, secreted frizzled related proteins, secreted frizzled-related protein-2, secreted apoptosis-related protein 1, SFRP, FRP2, Secreted frizzled related protein 2, SDF-5, hSFRP2, FRP-2, hFRP-2 |
| PHIP | ENSP00000275034 | WDR11, pleckstrin homology domain-interacting protein, NDRP, DCAF14, pleckstrin homology domain interacting protein, PH-interacting protein, phIP |
| SLC2A12 | ENSP00000275230 | GLUT8, GLUT-12, GLUT12, SLC2A12, SLC2a-12 |
| GNA12 | ENSP00000275364 | GNA12, NNX3, RMP, guanine nucleotide-binding protein alpha-12, gep |
| NONO | ENSP00000276079 | p54, NONO, p54nrb, p54(nrb, non-POU domain containing octamer-binding, nmt55, 55 kDa nuclear protein, non-POU domain-containing octamer-binding protein, non-POU-domain-containing octamer-binding, non-POU domain-containing octamer binding protein, non-POU domain containing octamer binding, Non-POU-domain-containing octamer-binding protein, non-POU domain-containing octamer binding |
| DLC1 | ENSP00000276297 | Dlc-1, DLC1, HDL-C 1, ARHGAP7, deleted in liver cancer 1, p122 RhoGAP, hdlc1, p122RhoGAP, Hp protein, deleted in liver cancer-1, deleted in liver cancer1, DLC1 Rho GTPase-activating protein, rho GTPase-activating protein 7, DLC1 Rho GTPase activating protein, Deleted-in-liver cancer 1 |
| PLIN2 | ENSP00000276914 | perilipins, ADRP, Adipophilin, adipose differentiation-related protein, ADFP, adipose-differentiation-related protein, perilipin 2, PLIN2, perilipin-2, adipose-differentiation related protein, Perilipin2, adipose differentiation related protein, Plin 2, PLIN-2 |
| EI24 | ENSP00000278903 | ei24, PIG8, HepG4, Pig 8, etoposide-induced 2.4 mRNA, etoposide induced 2.4 |
| SLC7A11 | ENSP00000280612 | xCT, cystine/glutamate transporter, SLC7a11, X CT, solute carrier family 7 member 11, x-CT |
| NOCT | ENSP00000280614 | CCR4, N-Oct, Nocturnin, Ccrn4l, Ccr4p, Noct |
| NDUFC2 | ENSP00000281031 | NDUFC2, B14.5b, HLC-1 |
| BMP3 | ENSP00000282701 | bone morphogenetic proteins, Bone morphogenetic protein-3, BMP-3, BMP3, osteogenin, bone morphogenetic protein 3, bone morphogenetic proteins, Bmp, BMP 3 |
| CYP2C18 | ENSP00000285979 | EC 1.14.14.1, Cyp-2c, CYP2C18, CYP2C, CYP 2C, CYP2C-18 |

| | | |
|---|---|---|
| RBM45 | ENSP00000286070 | RB1, RB-1, Drb1, Rb 1, RBM45, Developmentally Regulated RNA-binding Protein 1, RNA-binding Motif Protein 45, RNA binding motif protein 45 |
| CASP10 | ENSP00000286186 | caspase-10, CASP10, Mch4, FLICE2, CASP-10, caspase 10, death effector domain containing, Casp 10 |
| FZD7 | ENSP00000286201 | FZD7, FzE3, Frizzled-7, Frizzled 7, Frizzled homolog 7, frizzled7, frizzled family receptor 7, FZD-7, frizzled class receptor 7 |
| NAT2 | ENSP00000286479 | aac2, NAT2, N-acetyltransferase 2, polymorphic arylamine N-acetyltransferase, EC 2.3.1.5, arylamine N-acetyltransferase 2, N-acetyltransferase type 2, N-acetyltransferase-2, N-acetyl transferase-2, N-acetyl transferase 2, HNAT2, NAT-2, N -acetyltransferase 2, NAT 2, N-acetyltransferase2, arylamine-N-acetyltransferase 2, N-acetyl transferase2, Arylamine N-acetyltransferase-2, N-acetyl transferase type 2, N-acetyltransferase-2, N-acetyl-transferase 2, N-acetyltransferase 2 (arylamine N-acetyltransferase, Aac2p |
| ADK | ENSP00000286621 | adenosine kinase, ADK, EC 2.7.1.20 |
| ZFYVE9 | ENSP00000287727 | SARA, novel serine protease, Nsp, SMAD anchor for receptor activation, novel serine-protease, N-SP, ZFYVE9 |
| FZD1 | ENSP00000287934 | frizzled-1, Wnt-receptor, FZD 1, FZD1, WNT receptor, frizzled 1, Frizzled1, FZD-1, frizzled class receptor 1 |
| CCNB2 | ENSP00000288207 | Cyclin B2, CCNB2, cyclin-B2, cyclinB2 |
| DUSP2 | ENSP00000288943 | PAC1, PAC-1, DUSP2, dual specificity phosphatase 2, dual-specificity phosphatase-2, dual-specificity phosphatase 2, DUSP-2, Dual-specificity-phosphatase-2, PAC 1 |
| N | ENSP00000290277 | A-RAF, A-raf-1, proto-oncogene A-raf-1, ARAF1, AR-AF-1, RAF family, hA-raf-1, AR AF-1, ARAF, AR-AF1, Pks2, AR AF1 |
| IGF2BP1 | ENSP00000290341 | CRD-BP, ZBP1, coding region determinant binding protein, Igf2bp1, Imp1, IMP-1, coding region determinant-binding protein, Zipcode binding protein 1, hIMP1, IGF II mRNA-binding protein 1, zipcode-binding protein 1, Insulin-like growth factor 2 mRNA-binding protein-1, insulin-like growth factor 2 mRNA-binding protein 1, insulin-like growth factor 2 mRNA binding protein 1, insulin-like growth factor-2 mRNA-binding protein 1, IGF-2BP-1, IGF2BP 1, insulin like growth factor 2 mRNA binding protein 1, ZBP-1, VICKZ1, insulin-like growth factor-2 mRNA-binding protein-1 |

| | | |
|---|---|---|
| BMPR2 | ENSP00000363708 | PPH1, BMPR2, BMPR-II, BMPRII, bone morphogenetic protein receptor type II, bone morphogenetic protein receptor type 2, BMP-RII, bone morphogenetic protein receptor type-2, BMPR-2, BMP-R2, BMPR II, Bone morphogenetic protein receptor, type II, bone morphogenetic protein receptor type-II, BMPR2 p, t-ALK |
| ECE1 | ENSP00000364028 | ECE-1, ECE1, endothelin-converting enzyme 1, endothelin converting enzyme-1, endothelin-converting enzyme-1, hECE-1, Ece1p, endothelin converting enzyme 1, Endothelin converting-enzyme-1 |
| TGFBR1 | ENSP00000364133 | TbetaRI, ALK-5, ALK5, TGFBR1, ESS1, TbetaR-I, TGF-beta type I receptor, activin receptor-like kinase 5, T beta R-I, TBRI, TGFbeta type I receptor, transforming growth factor beta receptor type I, TGF beta type I receptor, T beta RI, transforming growth factor-beta receptor type I, transforming growth factor beta receptor I, ESS-1, activin-receptor-like kinase-5, transforming growth factor beta-receptor 1, transforming growth factor beta receptor 1, transforming growth factor-beta receptor 1, TbetaR I, TGF-beta type-I receptor, activin receptor-like kinase-5, TGFBR-1, TGFR1, TGF-beta receptor type 1, transforming growth factor beta-receptor I, TGFB-R1, Activin receptor-like kinase5, TGF-beta receptor type-1, transforming growth factor-beta receptor I, Transforming Growth Factor B Receptor I |
| MAP3K20 | ENSP00000364361 | MLTKalpha, MRK, MLTKbeta, MLTK, mlt, MLK-like mitogen-activated protein triple kinase, MLK7, leucine zipper and sterile alpha motif-containing kinase, sterile alpha motif and leucine zipper containing kinase AZK, MAP3K20 |
| PAX7 | ENSP00000364524 | Pax7, Pax-7, HuP1, paired boxes, paired box 7, Pax 7, paired-box 7 |
| PADI4 | ENSP00000364597 | peptidylarginine deiminases, PADI4, PAD4, EC 3.5.3.15, peptidylarginine deiminase 4, peptidyl arginine deiminase 4, HL-60 PAD, pAD, peptidylarginine deiminase IV, PAD-4, PADI-4, Peptidyl arginine deiminase, type IV, Peptidyl arginine deiminases, peptidylarginine deiminase-4, Peptidyl arginine deiminase IV, Peptidyl arginine deiminase-4, 1WDA |
| PADI3 | ENSP00000364609 | PADI3, peptidylarginine deiminases, EC 3.5.3.15, peptidylarginine deiminase 3, Pad3, PAD-3, peptidyl arginine deiminases |

| | | |
|---|---|---|
| PADI2 | ENSP00000364635 | peptidylarginine deiminases, EC 3.5.3.15, pad-2, peptidyl arginine deiminase 2, PAD2, peptidylarginine deiminase 2, Peptidyl argininedeiminase 2, PADI2, Peptidyl arginine deiminases, hPAD2, peptidylarginine deiminase-2, Peptidyl Arginine Deiminase,  Type II |
| F7 | ENSP00000364731 | factor VII, coagulation factor VII, SPCA, proconvertin, factor-VII, EC 3.4.21.21, 3 Th2, coagulation factorVII, factorVII, SPC-A, Eptacog Alfa, 5 PAS |
| DLG2 | ENSP00000365272 | PSD-93, chapsyn-110, 2 by G, DLG2, DLG-2, PSD93, discs large MAGUK scaffold protein 2, Disks Large Homolog 2, discs large homolog 2 |
| APBB1IP | ENSP00000365411 | RARP1, RIAM, Rap1-GTP-interacting adaptor molecule, APBB1IP |
| FLOT1 | ENSP00000365569 | flotillin-1, flotillin 1, FLOT1, flotillin1, Flot-1 |
| PSAT1 | ENSP00000365773 | PSAT, PSAT1, phosphoserine aminotransferase, NLS2, NLS-2, Psa, phosphoserine aminotransferase 1, PSA T |
| ANXA1 | ENSP00000366109 | annexin I, AnxA1, lipocortin I, annexins, annexin-1, Lpc1, annexin A1, phospholipase A2-inhibitory protein, annexin 1, Anx-1, p35p, annexin-I, annexin-A1, AnnexinA1, ANX1, Anx-A1, ANX A1, ANX - A1 |
| CASZ1 | ENSP00000366221 | Casz1, survival-related gene, castor zinc finger 1 |
| DFFA | ENSP00000366237 | DFF45, DFFA, ICAD, inhibitor of CAD, DFF-45, h13 |
| TRIM27 | ENSP00000366404 | rfp, RET finger protein, TRIM27, ret-finger protein, tripartite motif containing 27, TRIM 27, tripartite motif-containing protein 27, tripartite motif-containing 27, R-FP |
| POU4F1 | ENSP00000366413 | Brn-3a, RDC-1, RDC1, Brn3a, POU4F1, Pou domain,  class 4, transcription factor 1, POU4F1 p |
| SLC2A5 | ENSP00000366641 | GLUT5, fructose transporter, SLC2A5, Glut-5, GLUT 5, solute carrier family 2 member 5 |
| ERRFI1 | ENSP00000366702 | mig-6, Mig6, gene 33, RALT, ERRFI1, ERBB receptor feedback inhibitor 1, ERRFI-1, mitogen-inducible gene 6 protein, Gene33, ERBB receptor feedback inhibitor-1 |
| POLR1E | ENSP00000367029 | PAF53, POLR1E, P-Raf-1, p-Raf1, PR AF1 |
| HES2 | ENSP00000367065 | HES2, AK091122 |
| KCNAB2 | ENSP00000367323 | Kvbeta2, Kv beta 2, KCNA2B, Kvbeta 2, aldo-keto reductases, Kcnab2, aldoketoreductases, Kv beta2, potassium voltage-gated channel,  shaker-related subfamily,  beta member 2, KCNK2, aldoketo reductases |

| | | |
|---|---|---|
| IL1RAPL1 | ENSP00000368278 | IL1RAPL1, IL1RAPL, MRX21, TIR-domain-containing, MRX10, IL1RAPL-1, TIR domain containing, Interleukin-1 Receptor Accessory Protein-Like 1, IL-1R8, TIR domain-containing, Interleukin-1-receptor accessory protein like 1, interleukin 1 receptor accessory protein-like 1, interleukin-1 receptor accessory protein like 1, MRX34, TIRdomain-containing, TIGIRR2, IL-1RAPL1, mental retardation, X-linked 21, IL1R8 |
| DDX53 | ENSP00000368667 | cancer-associated gene, DDX53, cagE, C T26, cancer-associated gene |
| NAA16 | ENSP00000368716 | NAT2, NAT-2 |
| NEDD9 | ENSP00000368759 | CasL, NEDD9, HEF1, p105, CAS2, Cas-L, enhancer of filamentation 1, Crk-associated substrate-related protein, CASS2, neural precursor cell expressed, developmentally down-regulated 9, Neural precursor cell-expressed, developmentally downregulated 9, neural precursor cell expressed, developmentally downregulated 9, neural precursor cell expressed developmentally down-regulated protein 9, neural precursor cell-expressed developmentally down-regulated protein 9, neural precursor cell expressed, developmentally-downregulated 9, neural precursor cell expressed developmentally downregulated protein 9, Neural precursor cell-expressed, developmentally-downregulated 9, neural precursor cell-expressed developmentally downregulated protein 9 |
| HGSNAT | ENSP00000368965 | TMEM76, HGSNAT, heparan-alpha-glucosaminide N-acetyltransferase, EC 2.3.1.78, transmembrane protein 76 |
| STEAP4 | ENSP00000369419 | six transmembrane protein of prostate 2, STAMP2, Steap4, six-transmembrane protein of prostate 2, six transmembrane epithelial antigen of prostate 4, Six-transmembrane epithelial antigen of prostate4, TIARP, Six-transmembrane epithelial antigen of prostate 4, STEAP-4, TNFAIP9, SchLAH, seven chromosome locus associated with HCC |
| IFNB1 | ENSP00000369581 | IFN-beta, fibroblast interferon, IFNB, IFNbeta, interferon-beta, Ifnb1, IF-B, interferon beta, Interferon-beta 1, Ifb, IFN beta, interferon beta 1, interferon beta-1, interferon-beta1, Interferon beta1, IFN-b, Interferonbeta |
| RPS6 | ENSP00000369757 | 40S ribosomal protein S6, rpS6, ribosomal protein S6, ribosomal-protein-S6 |

| | | |
|---|---|---|
| AKR1C4 | ENSP00000369814 | 3 alpha-HSD, chlordecone reductase, Cdr, aldo-keto reductases, DD4, 3alpha-HSD, c11, aldoketoreductases, HAKRa, AKR1C4, 3 alpha HSD, aldoketo reductases, aldo-keto reductase family 1 member C4 |
| AKR1C3 | ENSP00000369927 | DD3, aldo-keto reductases, HAKRb, prostaglandin F synthase, HAKR e, DDX, aldoketoreductases, DDH1, AKR1C3, DD 3, aldo-keto reductase family 1, member C3, 17-beta-hydroxysteroid dehydrogenase type 5, HSD17B5, aldoketo reductases, 17beta-hydroxysteroid dehydrogenase type 5, aldo-keto reductase family 1 member C3, DD-3, 1 S1P, aldo-keto reductase family1 member C3, DDH-1 |
| SLC22A18 | ENSP00000369948 | TSSC5, HET, IMPT1, ORCTL2, SLC22A18, BWR1A, SLC22A1L, efflux transporter-like protein, solute carrier family 22 member 18, solute carrier family 22, member 18, h-ET |
| DDX1 | ENSP00000370745 | DDX1, DEAD box polypeptide 1, DEAD box protein 1 |
| GNG7 | ENSP00000371594 | GNG7 |
| CHEK2 | ENSP00000372023 | Cds1, hCHK2, Chk2, hCds1, RAD53, CHEK2, HuCds1, checkpoint kinase 2, Rad 53, CHEK 2, checkpoint kinase2, CDS-1, Chk-2, CHEK2 p, checkpoint kinase-2 |
| RBMS3 | ENSP00000373277 | RNA-binding protein, RNA binding protein, RBMS3, RBP, RNA binding motif, single-stranded interacting protein 3, RNA binding-protein, RNA-binding-protein |
| KRT18 | ENSP00000373487 | CK18, CK 18, keratin 18, cytokeratin 18, KRT18, cytokeratin-18, CK-18, Cytokeratin18, K18 p, hK18, keratin-18, keratin18, k18, cytokeratin 1-8 |
| AKT1S1 | ENSP00000375711 | PRAS40, mTOR complex 1, mTOR complex1, Akt1s1, mTOR complex-1, PRAS 40, AKT1 substrate 1, P-RAS40 |
| ATG16L1 | ENSP00000375872 | Apg16L, ATG16L1, Atg16L, autophagy-related 16-like 1, autophagy related 16-like 1, ATG16L1 p, Autophagy-related 16 like-1, ATG16L-1, autophagy related 16 like 1, autophagy-related protein 16-1, autophagy related 16-like 1 (S. cerevisiae |
| URI1 | ENSP00000376097 | RMP, NNX3, RPB5-mediating protein, unconventional prefoldin RPB5 interactor, URI1, RNA polymerase II subunit 5-mediating protein |
| GPS1 | ENSP00000376167 | Sgn1, gps1, 4D10, CSN1, GPS 1, G Protein Pathway Suppressor 1 |

| | | |
|---|---|---|
| TIRAP | ENSP00000376445 | MAL, TIRAP, TIR-domain-containing adaptor protein, TIR-domain-containing, TIR domain-containing adaptor protein, MyD88 adapter-like, TIR domain-containing, TIR domain containing, Toll-interleukin-1 receptor domain-containing adaptor protein, Toll-interleukin 1 receptor domain containing adaptor protein, m AL, Toll-interleukin 1 receptor (TIR) domain containing adaptor protein, Toll-interleukin-1 receptor (TIR) domain-containing adaptor protein, Toll-interleukin 1 receptor domain-containing adaptor protein, TIRdomain-containing, MyD88 adapter-like protein, MyD88-adapter-like protein |
| PRIMA1 | ENSP00000376848 | PRiMA, membrane anchor of acetylcholinesterase, PRIMA1, prima-1, proline rich membrane anchor 1 |
| NRF1 | ENSP00000376924 | NRF-1, Nrf1, alpha-Pal, nuclear respiratory factor 1, ewg, Nuclear respiratory factor-1, alpha -Pal, NRF 1 |
| CNOT6 | ENSP00000377024 | CCR4, Ccr4a, cytoplasmic deadenylase, CNOT6, Ccr4p, CCR4-NOT transcription complex |
| NUMA1 | ENSP00000377298 | NuMA, nuclear mitotic apparatus protein, NUMA1, nuclear mitotic apparatus protein 1, nuclear-mitotic apparatus protein, NMP-22, NMP22, nuclear matrix protein 22 |
| PC | ENSP00000377532 | Pyruvate carboxylase, EC 6.4.1.1, pC201, PC-B, Pyruvate-Carboxylase |
| ANXA4 | ENSP00000377833 | ZAP36, ANX4, PP4-X, annexin IV, annexins, protein II, annexin 4, placental anticoagulant protein II, endonexin I, annexin A4, ANXA4, Anx A4, Annexin-4 |
| PSAP | ENSP00000378394 | prosaposin, SAP-1, sphingolipid activator protein-1, PSAP, SAP1, sphingolipid activator protein 1, PSA P, SAP 1, Sap1p, proactivator polypeptide, P-SAP |
| MATK | ENSP00000378485 | LSK, MATK, megakaryocyte-associated tyrosine kinase, CHK, hyl, csk-type protein-tyrosine kinase, tyrosine kinase MATK, Csk-homologous kinase, hematopoietic consensus tyrosine-lacking kinase, Csk homologous kinase, ch-K |
| GPR135 | ENSP00000378548 | GPR135, PAF-R, PAFR |
| SULT1A3 | ENSP00000378570 | SULT1A3, TL PST, placental estrogen sulfotransferase, STP1, thermolabile phenol sulfotransferase, SULT1A4, ST1A5, M-PST, s-TM, sTM, MP-ST, hAST, SULT 1A3, Sulfotransferase 1A3, STP-1 |
| SULT1A2 | ENSP00000378992 | P-PST, STP2, TS PST2, SULT1A2, STP-2 |
| CAST | ENSP00000379157 | calpastatin, c-AST, cAST, PLACK |

| | | |
|---|---|---|
| CD59 | ENSP00000379191 | CD59, HRF20, 1F5, MEM-43, protectin, MIC11, membrane inhibitor of reactive lysis, MIRL, 1F5 antigen, MEM43, EJ-30, HM in 2, hCD59, CD59 p, CD59 glycoprotein, CD 59, MAC-inhibitory protein, MAC-IP, 20-kDa homologous restriction factor |
| BMP7 | ENSP00000379204 | bone morphogenetic proteins, Bmp7, BMP-7, OP-1, bone morphogenetic protein 7, bone morphogenetic protein-7, Hop1, osteogenic protein-1, 1 Bmp, BMP 7, osteogenic protein 1, Bone morphogenetic protein -7, bone morphogenetic proteins, hBMP7, Bone-morphogenetic protein-7, Bmp, oP1, BMP7-p, hop-1, BMP-7 human |
| MOB1A | ENSP00000379364 | Mob1, Mats1, Mob-1, hMOB1, MOB1A |
| PRH2 | ENSP00000379682 | protein C, Dbs, PRH1, protein-C, proline-rich proteins, PRH2, parotid proline-rich protein, parotid acidic protein, PIFs |
| DKK3 | ENSP00000379762 | Dkk-3, RIG, hDkk-3, DKK3, Dickkopf-homolog 3, dickkopf homolog 3, Dickkopf-related protein 3, REIC, dickkopf WNT signaling pathway inhibitor 3, regulated in Glioma |
| ITGA4 | ENSP00000380227 | integrin alpha subunits, CD49d, integrin alpha-subunits, integrin subunit alpha 4, integrin alpha 4, integrin alpha4, IA-4, ITGA4, Integrin alpha-4, integrin, alpha 4 |
| MB | ENSP00000380489 | myoglobin, PVALB |
| MAP2K7 | ENSP00000381070 | MKK7, JNKK2, mitogen-activated protein kinase kinase 7, MAP2K7, MKK-7, MKK7 p, Mitogen-activated protein kinase kinase-7 |
| GRIP1 | ENSP00000381098 | GRIP-1, GRIP, GRIP1, glutamate receptor interacting protein 1, glutamate receptor-interacting protein 1, glutamate receptor-interacting-protein 1, LGALS12, Glutamate Receptor Interacting Protein1, glutamate-receptor-interacting protein 1 |
| CBS | ENSP00000381231 | cystathionine-beta-synthase, Cystathionine beta-synthase, Cbs, cystathionine beta synthase, cystathionine beta--synthase, hip4, cystathionine-beta -synthase, cystathionine betasynthase, cystathionine-beta synthase, cystathionine beta synthase, 1JBQ |
| CBSL | ENSP00000381234 | cystathionine beta synthase, cystathionine beta-synthase, Cbs, cystathionine beta--synthase, cystathionine-beta-synthase, hip4, EC 4.2.1.22, serine sulfhydrase, cystathionine-beta -synthase, cystathionine betasynthase, cystathionine-beta synthase, cystathionine beta synthase |

| | | |
|---|---|---|
| MX1 | ENSP00000381601 | MxA, Hmx1, MX1, IFI-78K, MX-1, MX-1 human, interferon-regulated resistance GTP-binding protein MxA, myxovirus resistance protein 1, MX dynamin-like GTPase 1, MX dynamin like GTPase 1 |
| YPEL3 | ENSP00000381821 | YPEL3, Yippee-like-3, Yippee-like 3 |
| CTNND1 | ENSP00000382004 | p120, p120(ctn, p120 catenin, p120-catenin, p120cas, p120ctn, CTNND1, CTNND, p120 CTN, catenin delta 1, p120catenin, p 120 catenin, Catenin delta-1, p120-catenin family, p120 catenin family, p120-ctn |
| DLG4 | ENSP00000382428 | PSD-95, DLG4, PSD95, SAP90, hPSD95, post-synaptic density protein 95, postsynaptic density protein-95, postsynaptic density protein 95, Discs large homolog 4, Synapse-associated protein 90, discs,  large homolog 4 (Drosophila, post-synaptic density protein-95, PSD 95 |
| BACH1 | ENSP00000382805 | BACH1, ha2303, AF026200, BTB and CNC homology 1,  basic leucine zipper transcription factor 1, BTB and CNC homolog 1, Bach-1, 2 IHC, BTB domain and CNC homolog 1 |
| PLA2G2A | ENSP00000383364 | PLA2G2A, PLA2s, Mom1, Mom-1, PLA2L, sPLA2, PlA2, Phospholipase A2 group IIA, group IIa phospholipase A2, phospholipase A2,  group IIA, sPLA-2, phospholipase-A2 group IIA |
| HPSE | ENSP00000384262 | Hpa1, hep, HPSE, Hpa, hHpr1, Hpa 1, hpr-1, HSE1, Hpr1, heparanase 1, HPA-1, heparanase-1, HPSE-1, HPSE1, HP-R1, HSE-1 |
| ZGLP1 | ENSP00000384434 | glp-1, ZGLP1, glp1, GATA like protein-1, GLP 1 |
| AKAP12 | ENSP00000384537 | gravin, SSeCKS, src-suppressed C-kinase substrate, Src-suppressed C kinase substrate, AKAP12, A-Kinase anchoring protein 12, A-kinase anchor protein 12, A-Kinase-Anchoring Protein 12, AKAP-12 |
| ABCB5 | ENSP00000384881 | ABCB5 P, ABCB 5alpha, ABCB5, ATP-binding cassette protein, ABCB 5beta, ATP binding cassette protein, ABCB 5, ATP-binding cassette, sub-family B,  member 5, ATP-binding cassette subfamily B member 5, ATP-binding cassette subfamily B, ATP-binding cassette sub-family B member 5, ATP binding cassette subfamily B member 5, ATP -binding cassette protein |
| SAA1 | ENSP00000384906 | SAA1, SAA2, SAA, serum amyloid-A-1, serum amyloid A1, serum amyloid A 1, SAA-1, hSAA1, s AA, serum amyloid A-1, serum amyloid A1 protein, SAA-2 |

| | | |
|---|---|---|
| FAH | ENSP00000385080 | FAH, fumarylacetoacetase, fumarylace-toacetate hydrolase, fumaryl acetoacetate hydrolase, fumarylacetoacetate hydrolase, FA-H, EC 3.7.1.2, fumarylacetoacetate-hydrolase, fumarylaceto-acetate hydrolase |
| NCOA1 | ENSP00000385216 | 5q14, 5q12, SRC-1, steroid receptor coactivator 1, 5q13, NCOA1, 5q11, NCoA-1, SRC1, 5q15, F-SRC-1, Steroid Receptor Coactivator-1, 5-q14, 3-DCT, steroid receptor co-activator-1, steroid receptor co-activator 1, 2 Hb H, nuclear receptor coactivator 1, 3D-CT, hNCOA1, 3D CT, 3DCT, 4 mgC, 4mgC, 3-D CT |
| SAR1B | ENSP00000385432 | SAR1B, SARA2, and--d, SAR1 homolog B, SARA--2 |
| MAGI1 | ENSP00000385450 | MAGI-1, BAP1, AIP3, WWP3, MAGI1, membrane-associated guanylate kinase, WW and PDZ domain containing 1, BAP-1, AIP-3, HBAP1 |
| GAL3ST1 | ENSP00000385735 | Gal3ST-1, cerebroside sulfotransferase, EC 2.8.2.11, galactose-3-O-sulfotransferase-1, GAL3ST1 |
| PLEKHB2 | ENSP00000385892 | evectin-2, evt-2, 3 via |
| RMDN1 | ENSP00000385927 | regulator of microtubule dynamics 1, RMD-1, RMD1, FAM82B, family with sequence similarity 82, member B |
| MBD5 | ENSP00000386049 | Cdc37, MBD5, Mrd1p, methyl-CpG-binding domain protein 5, methyl CpG-binding domain-containing, methyl-CpG binding domain protein 5 |
| SFTPB | ENSP00000386346 | surfactant protein B, SP-B, PSP-B, SFTPB, pulmonary surfactant-associated protein B, SFTP3, pulmonary surfactant-associated proteolipid SPL(Phe, surfactant protein-B, Sftp-3, pulmonary surfactant associated protein B, pSPB |
| PSMD14 | ENSP00000386541 | pad-1, POH1, pad1, Rpn11, PSMD14, Pad1p, 26S proteasome non-ATPase regulatory subunit 14 |
| CAND2 | ENSP00000387641 | TIP120B, TBP-interacting protein, CAND2 |
| RARG | ENSP00000388510 | RARG, retinoic acid receptor gamma, retinoic acid receptor-gamma, NR1B3, RARc |
| CASP4 | ENSP00000388566 | caspase-4, ICErelII, CASP4, ICErel-II, ICH-2, caspase 4, CASP-4, MIH1, caspase4 |
| CHEK1 | ENSP00000388648 | Chk1, checkpoint kinase 1, CHEK1, chk1 homolog, CHK1 checkpoint homolog, cell cycle checkpoint kinase, cell-cycle checkpoint kinase, hCHK1, CHK-1, CHEK-1, check-point kinase 1, checkpoint kinase-1, Chk 1, 4 FTO, 3 TKI, Checkpoint kinase1, check point kinase 1, serine/threonine-protein kinase CHK1 |
| EIF4B | ENSP00000388806 | eIF-4B, eIF4B, eukaryotic translation initiation factor 4B |

| | | |
|---|---|---|
| AKR1B15 | ENSP00000389289 | AKR1B15, aldo-keto reductases, aldoketoreductases, aldoketo reductases |
| PLEKHM1 | ENSP00000389913 | Plekhm1, HB2, AP162, Pleckstrin homology domain-containing family M member 1 |
| PEX5 | ENSP00000391601 | Pex5p, PEX5, PTS1R, PXR1, peroxins, Pxr1p, Peroxisomal targeting signal-1 receptor, peroxin 5 |
| BCAP31 | ENSP00000392330 | BAP31, BCAP31, DXS1357E, B-cell receptor-associated protein 31, Cdm, hp28, HP-28, c DM, BCAP 31, c-DM |
| CDC7 | ENSP00000393139 | huCdc7, Cdc7, Hsk1, HsCdc7, Cdc7p, CDC7L1, cell division cycle 7, cell division cycle 7 homolog, Cell division cycle-7, cell division cycle 7-related protein kinase |
| PLA2G10 | ENSP00000393847 | sPLA2, hGX sPLA2, sPLA2-X, sPLA-2, PLA2G10, GX-sPLA2 |
| LPAR3 | ENSP00000395389 | EDG7, HOFNH30, LPA3, Edg-7, GPC-R, lysophosphatidic acid receptor 3, lysophosphatidic acid receptor-3, LPAR3, GPcR |
| URGCP | ENSP00000396918 | URG4, Upregulated gene 4, Upregulator of cell proliferation, URGCP, up regulated gene 4, Up-regulated gene-4, up-regulator of cell proliferation, URG-4, Up-regulated gene 4, upregulated gene-4 |
| SFTPA1 | ENSP00000397082 | SP-A1, SPA-1, PSAP, collectins, PSP-A, SFTP1, Sftp-1, Sftpa, SFTPA1, SFTPA1B, PspA, surfactant protein A1, PSA P, hSP-A1, SPA1, HSPA1, surfactant protein-A1, P-SAP, sPA, SP A1 |
| E2F5 | ENSP00000398124 | E2F-5, E2F transcription factors, E2F5, E2F transcription factor 5 |
| ANXA11 | ENSP00000398610 | Annexin A11, ANXA11, annexin XI, ANX11, annexins |
| ERN1 | ENSP00000401445 | IRE1alpha, hIre1p, Ire1p, IRE1 alpha, inositol-requiring protein 1, Inositol requiring enzyme-1, Inositol-requiring enzyme 1, Inositol-requiring enzyme-1, IR-E1A, IRE1-alpha, inositol-requiring protein-1, inositol requiring enzyme 1, IRE1a, serine/threonine-protein kinase/endoribonuclease IRE1, 4U6R, IRE-1a |
| CLSTN2 | ENSP00000402460 | CLSTN2, CS-2, HCS-2, HCS2, calsyntenin-2, calsyntenin2 |
| SMG1 | ENSP00000402515 | hSMG-1, SMG-1, SMG1, Atx, phosphatidylinositol-3-kinase-related kinase, phosphatidylinositol 3-kinase-related kinase, phosphatidylinositol 3-kinase related kinase, Phosphatidylinositol-3-kinase related kinase |
| PMEL | ENSP00000402758 | p100, gp100, Pmel17, SILV, melanocyte protein Pmel 17, Pmel 17, ME20, ME20-M, D12S53E, Pmel, melanoma-associated ME20 antigen, gp-100, Pmel-17, hgp100, siL, ME 20, P1P, s-IL, Silver-like, premelanosome protein, melanocyte protein PMEL, Hgp 100, GP 100 |

| | | |
|---|---|---|
| GRB7 | ENSP00000403459 | Grb7, GRB-7, Grb7 adapter protein, hGrb7, growth factor receptor-bound protein 7, growth factor receptor bound protein 7, Growth factor receptor-bound protein-7, Growth factor receptor bound protein-7 |
| ILF3 | ENSP00000404121 | NF90, DRBP76, NFAR-1, MPP4, Ilf3, NFAR, NFAR-2, nuclear factor associated with dsRNA, interleukin enhancer binding factor 3, double-stranded RNA-binding protein 76, ILF-3, Interleukin enhancer-binding factor 3, translational control protein 80, TCP80, NF110, MMP4, DR-Bf |
| RPL36A | ENSP00000404375 | rpL36a, Mig6, mig-6, ribosomal protein L36a, RPL44, L36a, rp L44 |
| OTUD4 | ENSP00000409279 | OTUD4, HIN-1, HIN1, OTU deubiquitinase 4 |
| ARRB1 | ENSP00000409581 | beta-arrestin1, beta-arrestin 1, ARRB1, arrestin-2, arrestin 2, beta-arrestin-1, Arr1, arrestin-beta1, betaArrestin1, arrestin2, arr-1, arrestin beta 1 |
| SYNJ1 | ENSP00000409667 | synaptojanin 1, SYNJ1, phosphoinositide 5-phosphatase, phosphoinositide-5-phosphatase, synaptojanin-1, synaptojanin1, PARK20, SYNJ1 p |
| SCN5A | ENSP00000410257 | SCN5A, Nav 1.5, hHb1, HB-1, SSS1p, hHb2, PFHB1, Nav1.5, Hbbd, HB2, HB1, HH-B2, sodium channel, voltage gated, type V, alpha subunit, SCN5A-p, SCN5A p, sodium channel, voltage-gated, type V, alpha subunit, Sss1, sodium channel, voltage gated, type V alpha subunit, sodium voltage-gated channel alpha subunit 5, SCN5A_p, Cmpd2 |
| SFRP4 | ENSP00000410715 | frpHE, secreted frizzled related proteins, secreted Frizzled-related proteins, sFRP4, FrzB-2, Secreted frizzled-related protein 4, sFRP-4, secreted frizzled-related protein-4, FRP-4, SFRP, FRP4, Secreted frizzled related protein 4, PYL |
| RPP14 | ENSP00000412894 | p14, Rpp14, HP-14, p 14, HP14 |
| FBLIM1 | ENSP00000416387 | FBLP-1, migfilin, CAL, filamin-binding LIM protein-1, Ca-L, FBLIM1, filamin-binding LIM protein 1, FBLP1, filamin binding LIM protein1 |
| EHMT1 | ENSP00000417980 | GLP-1, EHMT1, glp1, Eu-HMTase1, euchromatic histone-lysine N-methyltransferase 1, 5 VSD, euchromatic histone methyltransferase 1, 3 FPD, Glp, euchromatic histone methyltransferase-1, KMT1D, Euchromatic histone lysine methyltransferase 1, GLP 1 |
| PARP14 | ENSP00000418194 | KIAA1268, PARP-14, poly(ADP-Ribose) polymerases, EC 2.4.2.30, BAL2, PARP14, poly (ADP-ribose) polymerases, 4-abl, poly (ADP ribose) polymerases, 3Q71, Poly(ADP-ribose) polymerase family member 14, ARTD8, Bal-2, 4 ABL |

| | | |
|---|---|---|
| FTO | ENSP00000418823 | alpha-ketoglutarate-dependent dioxygenase, FTO, fat mass and obesity-associated, fat mass and obesity associated, fat mass- and obesity-associated, fat mass-and obesity-associated, fat-mass and obesity-associated, fat-mass-and-obesity-associated, Fat mass and obesity-associated protein, fat mass and obesity- associated, fat mass- and obesity- associated, ALKBH9, alpha-ketoglutarate dependent dioxygenase |
| NDUFA6 | ENSP00000418842 | Hb1-4, cI-B14, NDUFA6, LYR-motif-containing |
| WWTR1 | ENSP00000419465 | TAZ, Wwtr1, transcriptional coactivator with PDZ-binding motif, transcriptional co-activator with PDZ-binding motif, WW domain containing transcription regulator 1, transcriptional coactivator with PDZ binding motif, WW domain-containing transcription regulator 1, Transcriptional co-activator with PDZ binding motif, WW domain-containing transcription regulator protein-1, WW domain-containing transcription regulator protein 1, WW-domain-containing transcription regulator protein 1, WW domain containing transcription regulator protein 1 |
| KLF6 | ENSP00000419923 | KLF6, Zf9, PAC1, PAC-1, Kruppel-like factor 6, KLF-6, Kruppel-like factors, Bcd-1, BCD1, core promoter element binding protein, COPEB, CPBP, Kruppel like factor 6, ST-12, PAC 1 |
| ERVW-1 | ENSP00000419945 | syncytin, envelope glycoprotein, Syncytin 1, syncytin-1, HERV-W env, Envelope protein, ERVWE1, HERV-Wenv, envelope-glycoprotein, HERV-W envelope protein, HERVWE1, HERV-WE1, HERV-W-Env, syncytin1, ERVW-1 |
| KCNAB1 | ENSP00000419952 | aldo-keto reductases, Kv beta 1, KCNA1B, Kcnab1, aldoketoreductases, hKv beta 3, Kvbeta1, Kv beta1, aldoketo reductases |
| ZNF398 | ENSP00000420418 | ZER6, P5-1, p7-1, p71, p51 |
| SLIT2 | ENSP00000422591 | SLIT2, slit-2, SLIT 2, slit homolog 2, slit homolog 2 (Drosophila, slit guidance ligand 2 |
| ATG12 | ENSP00000425107 | Apg12p, Apg12, ATG12, hApg12p, ATG -12, hApg12, autophagy-related protein 12, Atg 12, Atg-12 |
| RAD17 | ENSP00000426191 | hRad17, Rad17, rad24, Rad17Sp, HRAD 17, R24L, Rad 24 |
| RNF4 | ENSP00000426503 | RNF4, SNURF, RING finger protein 4, RES4-26, Slx5, small nuclear RING finger protein |
| COX6C | ENSP00000428895 | COX6C, cytochrome c oxidase subunit VIc |
| CYRIB | ENSP00000429150 | FAM49B, family with sequence similarity 49 member B, L1P, BM009, HL -1 |

| | | |
|---|---|---|
| HDAC2 | ENSP00000430432 | RPD3, HDAC2, NuRD complex, Histone deacetylase 2, HD2, HDAC-2, SIN3 histone deacetylase complex, Rpd3p, histone deacetylase-2, HDAC 2, histone deacetylase2, EMSY Complex |
| IDO1 | ENSP00000430950 | IDO, IDO1, indoleamine 2, 3-dioxygenase 1, IDO-1, indoleamine-pyrrole 2, 3-dioxygenase, indoleamine 2, 3-dioxygenase-1, indoleamine 2, 3 dioxygenase 1, Indoleamine 2, 3 dioxygenase-1, indoleamine-2, 3-dioxygenase 1, IDO 1, indoleamine 2, 3-dioxygenase1, indoleamine-pyrrole-2, 3-dioxygenase, indoleamine-2, 3-dioxygenase1, indoleamine-2, 3-dioxygenase-1, indoleamine 2, 3-dioxygenase 1, indoleamine 2, 3-dioxygenase-1, indoleamine 2, 3 -dioxygenase 1, indoleamine-2, 3 dioxygenase 1, hIDO1 |
| EID1 | ENSP00000431162 | C15orf3, E1A-like inhibitor of differentiation 1, EID-1, EID1, E1A-like inhibitor of differentiation-1, CRI1, CRI-1, CREBBP/EP300 inhibitory protein 1, EP300 interacting inhibitor of differentiation 1 |
| API5 | ENSP00000431391 | AAC-11, apoptosis inhibitor-5, API-5, apoptosis inhibitor 5, API5, Apoptosis inhibitor5 |
| CAPN1 | ENSP00000431984 | calpain 1, CANP, muCANP, calpain-1, CAPN1, calpain1 |
| SPTBN2 | ENSP00000432568 | SCA 5, SCA5, beta-III spectrin, SPTBN2, betaIII spectrin, GTRAP41, Beta III spectrin, KIAA0302, beta-III-spectrin, FJ811850, spectrin, beta, nonerythrocytic 2, SCAR14, 1WYQ, 1WJM, SPTBN2 p, SCA-5 |
| TEAD1 | ENSP00000435233 | Ref-1, TEF1, TEF-1, Tead1, ref1, Ref 1, TEF 1, TEA domain family member 1, transcription factor 13, TEA domain transcription factors, TEA domain transcription factor 1, hTEF-1 |
| ENO2 | ENSP00000437402 | neuron-specific enolase, NSE, ENO2, Enolase-2, neuron specific enolase, gamma-enolase, neuron-specific-enolase, enolase 2, neuronspecific enolase, neuron-specific enolase |
| FZD3 | ENSP00000437489 | FZD3, frizzled-3, Frizzled 3, Frizzled3, Frizzled homolog 3, FZ3 |
| NFE2 | ENSP00000439120 | p45, NF-E2, p45 NF-E2, p45-NF-E2, Nfe2, p45 NFE2, nuclear factor, erythroid 2, NFE2 p |
| PDYN | ENSP00000440185 | Prodynorphin, dynorphin, PDYN, preproenkephalin B, preprodynorphin, SCA23, leu-enkephalin, pre-proenkephalin B, Ad-CA, pre-proenkephalin-B, AdcA, Spinocerebellar Ataxia 23 |
| DDX11 | ENSP00000440402 | CHLR1, DDX11, CHL1, hCHLR1, Chl1p, CHL-1 |

| | | |
|---|---|---|
| CCR3 | ENSP00000441600 | CCR3, CC CKR3, eosinophil eotaxin receptor, CKR-3, CCR-3, CC-CKR3, CMKBR3, hCCR-3, chemokine receptor-3, chemokine receptor 3, chemokine (C-C motif) receptor 3, C-C chemokine receptor type 3, CC chemokine receptor type 3, C-C motif chemokine receptors |
| LPAR2 | ENSP00000443256 | EDG4, LPA2, Edg-4, lysophosphatidic acid receptor-2, LPA 2, Lysophosphatidic acid receptor 2, LPAR2 |
| FKBP5 | ENSP00000444810 | 51-kDa FK506-binding protein, FKBP5, FKBP51, p54, PPIase, rotamase, 5 DIV, FKBP-51, FK506-binding protein 5, FK 506 binding protein 5, FK506 binding protein 5, Hsp90-binding immunophilin, FKBP-5, FK-506 binding protein 5, peptidyl-prolyl cis-trans isomerase FKBP5, 51 kDa FK506-binding protein |
| LDHA | ENSP00000445175 | Ldh-1, LDH M, LDHA, lactate dehydrogenase A, LDH A, EC 1.1.1.27, lactate dehydrogenase-A, LDH-A, LDH-M, Ldh1, L-lactate dehydrogenase A chain |
| PCSK5 | ENSP00000446280 | PC5, PCSK5, PC6, SP-C6, PC6A, proprotein convertase subtilisin/ kexin type 5, PC-6a, PC-5, PC-6, PC 5, PC 6, proprotein convertase subtilisin/kexin type 5 |
| TPCN1 | ENSP00000448083 | TPC-1, TPC1, TPCN1, TPC1 human, TPC-1 human |
| SLC38A1 | ENSP00000449756 | hATA1, SAT1, NAT2, Sat-1, SNAT1, ATA1, hsat-1, SLC38A1, NAT-2, Sodium-coupled neutral amino acid transporter 1, solute carrier family 38,  member 1, System A amino acid transporter1, solute carrier family 38 member 1, SAT 1 |
| DNAL1 | ENSP00000452037 | LC1, DNAL1, HLC-1, LC-1 |
| LDLR | ENSP00000454071 | fH C, fHC, 3 BPs |
| ITGAX | ENSP00000454623 | CD11c, integrin alpha-subunits, Leu M5, integrin alpha subunits, integrin alphaX, LeuM5, ITGAX, Integrin alpha x, Leu-M5, CD-11c |
| MLST8 | ENSP00000456405 | GbetaL, mLST8, mTOR complex 1, mTOR complex 2, mammalian lethal with SEC13 protein 8, mTor-complex-2, mTOR complex-1, mTOR complex-2, POP3, LST8, MTOR complex1, WAT-1 |
| CTRL | ENSP00000458537 | chymotrypsin-like, CTRL-1, chymotrypsinlike, CTRL1, ctrl, chymotrypsin like |
| DPEP2 | ENSP00000458977 | Mbd2, Mbd-2, EC 3.4.13.19, Dpep2 |
| USP6 | ENSP00000460380 | Tre2, tre, HRP-1, Tre-2, tre-2 oncogene, USP6, TRE17, HRP1, ubiquitin-specific protease 6, ubiquitin-specific peptidase 6, ubiquitin specific peptidase 6 |

| | | |
|---|---|---|
| YES1 | ENSP00000462468 | c-Yes, YES1, Src family tyrosine kinases, proto-oncogene c-yes, tyrosine protein kinase yes, Src-family tyrosine kinases, Yes-1, YES proto-oncogene 1, Src family tyrosine kinase |
| OAZ2 | ENSP00000463013 | Az2, ornithine decarboxylase antizyme 2, OAZ2, A-Z2 |
| NCOA4 | ENSP00000463027 | ELE1, PTC3, PTC-3, NCOA4, ARA70, nuclear receptor coactivator 4, PTC 3, nuclear receptor co-activator 4, Ret-activating protein ELE1, nuclear receptor coactivator-4, ARA-70 |
| RBP3 | ENSP00000463151 | RBP3, IRBP, interstitial retinol-binding protein, interphotoreceptor retinoid-binding protein, Rbp-3, interstitial retinol binding protein, interphotoreceptor retinoid binding protein, interstitial retinol-binding protein 3, inter-photoreceptor retinoid binding protein, retinol binding protein 3 |
| NBPF10 | ENSP00000463957 | Ag1, Ag-1, HAG-1, AG -1, Ag 1, NBPF10 |
| SLC16A3 | ENSP00000463978 | MCT3, MCT4, solute-carrier family 16, member 3, SLC16A3, Monocarboxylate transporter 4, MCT-4, MCT-3, monocarboxylate transporter-4, Solute Carrier Family 16 Member 3 |
| ELANE | ENSP00000466090 | neutrophil elastase, ELA2, HLE, elastase 2, leukocyte elastase, Elane, human leukocyte elastase, Scn 1, medullasin, EC 3.4.21.37, bone marrow serine protease, hNE, elastase-2, PMN elastase, ELA-2, SCN1, PMN-elastase, ELA 2, Elastase-2, neutrophil, elastase, neutrophil-expressed, elastase, neutrophil expressed, PMNE |
| HSD17B1 | ENSP00000466799 | 17BetaHSD, 17 beta-HSD, EDH17B2, 20 alpha-hydroxysteroid dehydrogenase, 20 alpha-HSD, HSD17B1, EDH17B1, 17beta-HSD, 17Beta-hydroxysteroid dehydrogenase type 1, 17-beta-HSD, 17 beta HSD, 20 alpha HSD, 20alphaHSD, 20alpha-HSD, 17-beta-hydroxysteroid dehydrogenase type 1, 17beta-hydroxysteroid dehydrogenase type1, HSD17, 20alpha-hydroxysteroid dehydrogenase, 17 beta hydroxy steroid dehydrogenase type 1, hydroxysteroid dehydrogenase 1, Hydroxysteroid dehydrogenase-1, 17 beta-hydroxysteroid-dehydrogenase type 1, Hydroxysteroid (17-beta)dehydrogenase 1, hydroxysteroid-dehydrogenase 1, Hydroxysteroid dehydrogenase - 1, 17 beta-hydroxysteroid dehydrogenase type 1 |
| RAD23A | ENSP00000467024 | hHR23A, RAD23A, HR23A |

| | | |
|---|---|---|
| SAFB | ENSP00000467423 | SAF-B, SAFB1, hap, hET, scaffold attachment factor B, SAFB, Hsp27 ERE-TATA binding protein, scaffold attachment factor-B, Hsp27-ERE-TATA-binding protein, Scaffold attachment factor B1, h-ET |
| TAZ | ENSP00000469981 | Taz1, TAZ, G4.5, tafazzin, Taz1p, XAP2, CMD3A, Efe2 |
| FGF21 | ENSP00000471477 | FGF-21, Fibroblast growth factor 21, FGF21, Fibroblast growth factor-21, hFGF-21, fibroblast-growth factor 21, FGF 21, fibroblast growth factor21 |
| H2AC19 | ENSP00000475814 | H2A, H-2a, H2A.2, H2-A |
| FRK | ENSP00000476145 | Src family tyrosine kinases, RAK, Fyn-related kinase, FRK, Src-family tyrosine kinases, fyn-related Src family tyrosine kinase, fyn related kinase, Fynrelated kinase |
| ANAPC10 | ENSP00000478501 | Doc1, APC10, doc-1, 5K-Hu, anaphase promoting complex subunit 10 |
| P3H3 | ENSP00000478600 | protein B, GRCB, Leprel2, proteinB, protein-B |
| DDX6 | ENSP00000478754 | RCK, p54, DDX6, DEAD-box helicase 6 |
| FMN1 | ENSP00000479134 | Formin, formin-1, Fmn1, Fmn, Formin1, Formin 1, Fmn 1 |
| PRH1 | ENSP00000479168 | protein C, protein-C, Dbs, PRH1, proline-rich proteins, PRH2, parotid acidic protein, PIFs |
| ADORA2A | ENSP00000480012 | ADORA2, ADORA2A, adenosine A2a receptor, A2aR, adenosine receptor A2A, 1 mm H, adenosine A2A-receptor, A2A R, A2 A R, SPECC1L-ADORA2A, adenosine-A2A receptor |
| ANXA8L1 | ENSP00000480221 | VAC-beta, annexins |
| CYFIP1 | ENSP00000481038 | SR-A1, Sra-1, specifically Rac1-associated protein-1, CYFIP1, Sra1, KIAA0068, p140Sra-1, cytoplasmic FMR1 interacting protein 1, Cytoplasmic FMR1-interacting protein 1, cytoplasmic FMRP-interacting protein 1, Cytoplasmic FMRP interacting protein 1 |
| SPRY1 | ENSP00000481675 | Sprouty, Spry1, hSpry1, SPRY-1, Sprouty RTK signaling antagonist 1 |
| NEFL | ENSP00000482169 | NF-L, NEFL, neurofilament light, NFL, NF68, Intermediate filaments type IV, neurofilament-light, neurofilament light polypeptide, NF--L, neurofilament,  light polypeptide |
| DHRS11 | ENSP00000482704 | 17 beta-hydroxysteroid dehydrogenase, 17beta-hydroxysteroid dehydrogenase, 17-beta-hydroxysteroid-dehydrogenase, estradiol 17 beta-dehydrogenase, 17-beta-hydroxysteroid dehydrogenase, 17 beta hydroxysteroid dehydrogenase, 17betahydroxysteroid dehydrogenase, 17-beta hydroxysteroid dehydrogenase |
| PADI6 | ENSP00000483125 | PADI6, EC 3.5.3.15, peptidylarginine deiminases, PAD-6, peptidyl arginine deiminases, PAD6 |

| | | |
|---|---|---|
| ADCYAP1R1 | ENSP00000483721 | PAC1, PAC-1, ADCYAP1R1, PACAP receptor 1, vasoactive intestinal peptide receptor family, PAC1-R, PAC1 R, pituitary adenylate cyclase-activating polypeptide type 1 receptor, PAC1R, pituitary adenylate cyclase-activating polypeptide type-1 receptor, adenylate cyclase activating polypeptide 1 (pituitary) receptor type I, PAC 1, PAC1p |
| IKBKG | ENSP00000483825 | NEMO, IKKgamma, IKK gamma, HIP1, IKBKG, Hip-2, Hip-1, NF-kappaB essential modifier, NF-kappaB essential modulator, IP1, IP2, FIP-3, FIP3, IKK-gamma, NF-kappa B Essential Modulator, NFkappaB essential modulator, inhibitor of nuclear factor kappaB kinase subunit gamma, NF- kappa B essential modulator, IKKAP1, inhibitor of kappa light polypeptide gene enhancer in B-cells, kinase gamma, IP-1, inhibitor of kappa light polypeptide gene enhancer in B cells, kinase gamma, HIP2, IKKG, NF-kappa-B essential modulator, inhibitor of nuclear factor kappa-B kinase subunit gamma |
| PCGEM1 | PCGEM1 | PCGEM1, PCAT9 |
| PVT1 | PVT1 | PVT1, pvt-1, Pvt 1 |
| HCP5 | HCP5 | p51, P5-1, HCP5 |
| MIR1225P | hsa-miR-122-5p | miR-122, miR-122a, hsa-mir-122, miR122, Mir122a, hsa-miR-122-5p, miR-122-5p, miR-122--a, miR122-5p |
| MIR1243P | hsa-miR-124-3p | miR-124, miR124a, miR-124a, miR 124a, miR 124, Hsa-miR-124a, hsa-miR-124, hsa-mir-124-3p, miR124, miR-124-3p, miR - 124a, miR124-a, hsa-mir124, MIR124-3p, miR-124- 3p |
| MIR1455P | hsa-miR-145-5p | mir-145, miR145, hsa-miR-145, miR- 145, miR 145, miR-145-5p, hsa-miR-145-5p, miR145-5p |
| MIR181A5P | hsa-miR-181a-5p | miR-181a, hsa-mir-181a, miR-181-a, miR181a, miR 181a, Hsa-miR-181a-5p, MiR-181a-5p, miR-181a5p |
| MIR217 | hsa-miR-217 | miR-217, hsa-miR-217, MIR217 |
| MIR315P | hsa-miR-31-5p | miR-31, hsa-miR-31, hsa-mir -31, MIR31, miR-31-5p, hsa-miR-31-5p, miR31-5p, hsa-miR31-5p, hsa-miR31 -5p, miR- 31 |
| MIR3693P | hsa-miR-369-3p | miR369, miR-369, hsa-mir-369, hsa-miR369-3p, miR-369-3p, miR369-3p |
| MIR3795P | hsa-miR-379-5p | miR-379, hsa-miR-379, miR379, miR-379-5p |
| MIR5703 | hsa-miR-5703 | miR-5703 |

| TABLE S5: GENE/PROTEIN NAMES ASSOCIATED WITH INFLUENZA |
|---|
| ABCA1 |
| ABCA3 |
| ABCB1 |
| ABL1 |
| AC007283.5 |
| AC015849.2 |
| ACE |
| ACE2 |
| ACKR2 |
| ACKR3 |
| ACP2 |
| ACP3 |
| ACTA1 |
| ACTC1 |
| ACTG1 |
| ACTG2 |
| ADAM11 |
| ADAMTS7 |
| ADAR |
| ADCY1 |
| ADGRL2 |
| ADM |
| ADM2 |
| AGA |
| AGO1 |
| AGO2 |
| AGTR1 |
| AHSA1 |
| AHSG |
| AICDA |
| AIFM1 |
| AIM2 |
| AIMP2 |
| AIP |
| AKT1 |
| ALB |
| ALDH16A1 |
| ALDH18A1 |
| ALDH1A1 |
| ALDH1A2 |
| ALDH1A3 |

ALDH1B1
ALDH1L1
ALDH1L2
ALDH2
ALDH3A1
ALDH3A2
ALDH3B1
ALDH3B2
ALDH4A1
ALDH5A1
ALDH6A1
ALDH7A1
ALDH8A1
ALDH9A1
ALK
ALYREF
AMOT
AMOTL1
AMOTL2
AMPH
ANKRD36B
ANP32A
ANP32B
ANPEP
ANXA1
ANXA2
ANXA5
ANXA6
ANXA7
AP2M1
APAF1
APCS
APEX1
API5
APOE
APOL2
APP
ARCN1
AREG
ARF3
ARG1
ARG2
ARHGAP21

ARHGEF7
ARNTL
ASCC1
ASNS
ASZ1
ATF3
ATG14
ATG5
ATG7
ATM
ATOH1
ATP12A
ATP4A
ATP7A
AURKAIP1
AZU1
B3GAT1
B3GNT3
B4GALNT2
BACE1
BACE2
BACH2
BAMBI
BANF1
BBOX1
BCAM
BCAT2
BCL2L2
BCL2L2-PABPN1
BCL6
BCL6B
BDNF
BECN1
BGN
BIRC3
BLOC1S3
BLOC1S5
BPIFA1
BSG
BST2
BZRAP1-AS1
C1D
C1QBP

C3
C5
C5AR1
C6orf47
C8orf44-SGK3
CADM1
CALCOCO2
CALM1
CALM2
CALM3
CALR
CAMKMT
CAMP
CAP1
CASD1
CASP1
CASP3
CASP8
CASP9
CAV1
CBX8
CCL11
CCL17
CCL2
CCL20
CCL22
CCL25
CCL28
CCL3
CCL4
CCL4L2
CCL5
CCL7
CCNA1
CCND3
CCNT1
CCR1
CCR3
CCR5
CCR7
CCR9
CD14
CD163

CD19
CD1A
CD1B
CD1C
CD1D
CD1E
CD2
CD209
CD248
CD27
CD274
CD276
CD28
CD33
CD4
CD40
CD40LG
CD44
CD46
CD55
CD59
CD69
CD7
CD70
CD80
CD81
CD86
CD8A
CD9
CDC25B
CDC42
CDH1
CDK1
CDK11A
CDK13
CDK5R1
CDK9
CDKN1A
CEACAM1
CEACAM6
CEBPB
CENPV
CEP70

CES1
CFDP1
CFL1
CFLAR
CFP
CFTR
CGAS
CHD6
CHD7
CHKB
CHMP4A
CHMP4B
CHP1
CHRM1
CISH
CKAP4
CLDN1
CLDN7
CLEC5A
CLEC7A
CLEC9A
CLK1
CNBP
CNOT4
COASY
COL11A1
COL11A2
COL1A1
COL2A1
COL3A1
COLEC10
COLEC11
COLEC12
COQ7
CPSF4
CPT2
CREB1
CREBBP
CRK
CRMP1
CRP
CRPPA
CSF1

CSF2
CSF2RA
CSF2RB
CSF3
CSRP1
CTCF
CTD-2639E6.9
CTHRC1
CTSB
CTSE
CTTN
CXCL1
CXCL10
CXCL12
CXCL8
CXCL9
CXCR1
CXCR2
CXCR3
CXCR4
CXCR5
CXCR6
CYBB
CYCS
CYLD
DAND5
DAPK2
DCTN2
DCTN4
DCTN6
DDIT4
DDR1
DDX1
DDX21
DDX39B
DDX3X
DDX41
DDX56
DDX58
DDX6
DDX60
DEFA1
DEFA1B

DEFA5
DEFB1
DEFB103A
DEFB103B
DENR
DGCR8
DHFR
DHRS2
DHX30
DHX36
DHX58
DHX9
DIAPH1
DICER1
DIRAS1
DISC1
DKK3
DLAT
DLL1
DMBT1
DNAJB1
DNM1L
DNMT1
DNMT3A
DNMT3B
DOT1L
DPEP2
DPF2
DPP4
DR1
DROSHA
DTNBP1
DUOX2
DUSP4
DYNC1LI1
DYNC1LI2
DYNLL1-AS1
DYRK3
E2F1
EEF1G
EGF
EGFR
EGOT

EHMT1
EIF2AK2
EIF2AK3
EIF4A1
EIF4A2
EIF4E
EIF4G1
EIF4G2
ELANE
ELF1
ENAH
ENC1
ENO1
ENPEP
EPCAM
EPG5
EPHA6
EPRS1
EPS8
EPX
ERBB2
ERBB3
ERN1
ERVW-1
ESR1
EXOSC10
EZH2
F2R
F2RL3
F3
F5
FABP5
FADD
FANCB
FAS
FASLG
FBXW11
FCAR
FCGR1A
FCGR2A
FCN1
FCN2
FER

FEZ1
FFAR2
FGF13
FGF2
FGF5
FGF7
FGFR1
FGFR2
FGFR4
FKBP10
FKBP1A
FKBP1B
FKBP3
FKBP4
FKBP5
FKBP6
FKBP7
FKBP9
FMR1
FN1
FOLH1
FOS
FOSB
FOXO1
FOXP3
FPR2
FURIN
FUT2
FYN
GABPA
GAD2
GAL
GALNS
GALNT15
GALNT3
GALNT7
GAPDH
GAST
GATA2
GATA3
GATD3A
GATD3B
GBP1

GBP5
GEM
GEMIN4
GINS4
GLDC
GLI1
GLRX
GNAQ
GOLPH3
GORASP1
GPI
GPRC5A
GPS1
GPT
GRAP2
GRK2
GRM5
GRN
GRP
GRSF1
GTF2H1
GZMA
GZMB
H1-2
H3-2
HAAO
HAMP
HAP1
HAVCR2
HAX1
HCN2
HCRT
HCRTR2
HDAC1
HDAC11
HDAC2
HDAC4
HDAC6
HDAC8
HEATR6
HECW1
HERC5
HERPUD1

HES1
HEY2
HIF1A
HIF1AN
HK2
HLA-A
HLA-B
HLA-C
HLA-DPA1
HLA-DPB1
HLA-DQA1
HLA-DQA2
HLA-DRB1
HLA-G
HMGB1
HMGCR
HMOX1
HMOX2
HNRNPC
HNRNPK
HOOK2
HOXD13
HP
HPD
HRAS
HSP90AA1
HSPA14
HSPA1A
HSPA1B
HSPA4
HSPA8
HSPD1
HTR2A
ICAM1
ICOS
ID1
ID2
IDO1
IFI27
IFI35
IFIT1
IFIT5
IFITM1

IFITM10
IFITM2
IFITM3
IFITM5
IFNA1
IFNA13
IFNA17
IFNA2
IFNA21
IFNA4
IFNA5
IFNA6
IFNA7
IFNAR1
IFNAR2
IFNB1
IFNG
IFNGR1
IFNL1
IFNL2
IFNL3
IFNLR1
IGF1
IGF2BP1
IGFBP3
IKBKB
IKBKG
IL10
IL10RA
IL10RB
IL13
IL15
IL17C
IL17D
IL17F
IL17RA
IL17RC
IL18
IL18BP
IL18R1
IL18RAP
IL1A
IL1B

IL1RAPL1
IL1RL1
IL1RL2
IL1RN
IL2
IL22
IL22RA1
IL22RA2
IL24
IL27RA
IL2RA
IL3
IL32
IL33
IL37
IL3RA
IL4
IL5
IL5RA
IL6
IL6R
IL6ST
IL7
IL7R
ILF3
INS
IPO5
IRAK1
IRAK3
IRF1
IRF3
IRF4
IRF5
IRF7
IRF9
ISG20
ISYNA1
ITGA1
ITGAE
ITGAM
ITGAX
ITPKB
IVNS1ABP

JAG1
JAG2
JAK1
JAK3
JCHAIN
JUN
JUNB
JUND
KARS1
KAT2A
KAT2B
KCNA1
KCNA5
KCNH4
KCNH8
KDM1A
KDM6A
KEAP1
KEL
KHDRBS1
KIF13A
KIF2A
KIR2DL1
KIR2DL3
KIR2DL4
KIR3DL1
KIR3DL2
KIR3DL3
KLF2
KLF6
KLK1
KLK2
KLK3
KLK4
KLK5
KLRB1
KLRC1
KLRC2
KLRD1
KLRG1
KMO
KPNA1
KPNA2

KPNA3
KPNA4
KRAS
KRIT1
KRR1
KRT10
KRT14
KRT5
LAG3
LAMP1
LANCL2
LARGE1
LBR
LEP
LET7B5P
LET7C5P
LET7E5P
LGALS1
LGALS3
LGALS9
LILRA6
LILRB1
LINC01587
LLGL1
LPO
LRP1
LRPPRC
LRRC3
LTA
LTF
LY75
LY96
LYAR
LZTS1
MAF
MAN2A1
MAP2K1
MAP2K3
MAP2K4
MAP2K6
MAP3K1
MAP3K19
MAP3K5

MAP3K7
MAPK1
MAPK14
MAPK3
MAPK8
MAPKAP1
MARK2
MAVS
MBD2
MBL2
MBP
MBTPS1
MCRS1
MCU
MDK
MDM2
MECP2
MED12
MED15
MED25
MET
MFSD2A
MGAT1
MICB
MIF
MIR1
MIR107
MIR1243P
MIR1249
MIR1254
MIR12541
MIR125A5P
MIR1260A
MIR1275
MIR1290
MIR13073P
MIR1323P
MIR1365P
MIR1443P
MIR146A5P
MIR146B5P
MIR1493P
MIR1555P

MIR155HG
MIR15A5P
MIR181C5P
MIR1825P
MIR184
MIR1873P
MIR1925P
MIR193B3P
MIR1941
MIR1945P
MIR198
MIR200A3P
MIR200C3P
MIR203A
MIR2045P
MIR206
MIR20A5P
MIR213P
MIR2233P
MIR223P
MIR243P
MIR29A3P
MIR29B3P
MIR29C3P
MIR302A3P
MIR315P
MIR320A
MIR323A3P
MIR326
MIR3283P
MIR3405P
MIR34A5P
MIR34C5P
MIR4514
MIR451A
MIR4833P
MIR4865P
MIR4915P
MIR5053P
MIR5091
MIR548D3P
MIR5845P
MIR5903P

MIR6545P
MIR664A3P
MIR7445P
MIR75P
MLANA
MLKL
MME
MMP13
MMP2
MMP9
MOGAT1
MOV10
MPO
MPRIP
MR1
MRC1
MRS2
MSR1
MST1
MT-CO2
MTOR
MTTP
MUC1
MUC13
MUC15
MUC16
MUC5AC
MUC5B
MX1
MX2
MYD88
MYO5B
MYOM2
NANOS2
NAT2
NCAM1
NCL
NCR1
NCR2
NCR3
NDFIP1
NDUFA2
NECTIN2

NEDD4
NEDD8
NEK3
NEK7
NEK8
NEK9
NELFCD
NEU2
NFE2L2
NFIA
NFKB1
NFKBIB
NFKBIL1
NLRC4
NLRC5
NLRP12
NLRP3
NLRX1
NOD1
NOD2
NOLC1
NOS2
NPM1
NPR2
NPRL2
NPTX1
NPY
NQO2
NR0B2
NR1D1
NR1I2
NR2C2
NR3C1
NR4A1
NRG1
NRP1
NRSN1
NSG1
NT5E
NTN1
NUDT11
NUP62
NUP93

OAS1
OAS2
OCA2
OCLN
OPRM1
OSM
OSTC
OTUD4
P2RX7
P2RY11
P4HB
PA2G4
PABPC1
PABPN1
PADI1
PADI4
PAGR1
PAK1
PANK4
PARP1
PARP11
PAWR
PAX5
PAX6
PDCD1
PDCD1LG2
PDE4B
PDIA2
PDIA3
PDIA4
PDK2
PDPK1
PDPN
PECAM1
PF4
PFN1
PFN2
PGAM5
PGPEP1
PHB
PHGDH
PI4KB
PIK3C3

PIK3R2
PKLR
PKM
PKN3
PKP2
PLAAT4
PLG
PLK1
PLK4
PLSCR1
PNKD
PNO1
POLD1
POLDIP2
POLI
PPARA
PPARG
PPAT
PPBP
PPIA
PPIE
PPIL1
PPIL6
PRDM1
PRDX1
PRF1
PRH1
PRH2
PRKD2
PRKRA
PRNP
PROC
PRPF18
PRPF8
PRSS12
PRSS57
PRSS8
PRTN3
PSAT1
PSG1
PSMB8
PSMB8-AS1
PSMD1

PSMD2
PSMD9
PTBP1
PTEN
PTGES3
PTGS1
PTGS2
PTK2
PTPN1
PTPN11
PTPN22
PTPRC
PTPRN
PVT1
PWAR1
PWAR4
PYCARD
RAB11A
RAB11FIP3
RAB23
RAB27A
RAB40B
RAB5A
RAB5B
RAB7A
RAB7B
RAB8A
RAC1
RAC2
RADIL
RAE1
RAF1
RAG1
RAG2
RALBP1
RALY
RANBP3
RAP2B
RAPGEF3
RARA
RARB
RB1CC1
RBBP6

RBM14
RBM14-RBM4
RBMS3
RC3H1
RELA
REN
REPIN1
RHO
RHOA
RHOD
RICTOR
RIPK1
RIPK3
RLN2
RNASE1
RNASE3
RNASEL
RNF135
RNF19A
RNF31
RNF5
ROBO3
RP11-670E13.5
RPE65
RPL17-C18orf32
RPL28
RPL6
RPS15
RPS28
RPS6KA1
RPS6KA2
RPS6KA3
RPS6KA5
RPSA
RPTOR
RRAD
RRAS
RSAD2
RTN3
RUBCN
S100A11
S1PR1
SAFB

SAG
SAMHD1
SART1
SCAP
SCGB2A2
SCGB3A1
SCNN1G
SDC1
SDCBP2
SEC14L2
SELL
SERPINA1
SERPINA3
SERPINA3
SERPINE1
SERPINH1
SETD7
SETDB1
SETX
SFTPA1
SFTPA2
SFTPB
SFTPD
SGK3
SH2D1A
SH2D2A
SH3GLB1
SH3GLB2
SHARPIN
SIGIRR
SIRT1
SLAMF1
SLAMF6
SLC19A2
SLC20A1
SLC22A8
SLC31A1
SLU7
SMAD3
SMARCA2
SMPD1
SMU1
SNCA

SNRNP200
SNRNP35
SOCS1
SOCS3
SOD1
SON
SP1
SPATA2
SPHK1
SPHK2
SPINT1
SPOCK2
SPP1
SPRY1
SPTBN1
SQSTM1
SRC
SRSF1
SRSF2
SRSF3
SRSF7
ST14
ST3GAL1
ST3GAL3
ST6GAL1
ST8SIA2
STAG1
STAT1
STAT2
STAT3
STAT6
STK4
STS
SULT2A1
SUMO1
SYT1
TAC1
TAP1
TARS1
TARS2
TARS3
TBK1
TBX21

TCEAL1
TCIM
TCN1
TEP1
TERT
TFF2
THBD
TICAM1
TIMP1
TINAGL1
TIPARP
TIRAP
TJP1
TLR10
TLR2
TLR3
TLR4
TLR5
TLR7
TLR8
TLR9
TMBIM6
TMED10
TMED2
TMED7
TMPRSS11A
TMPRSS15
TMPRSS2
TNC
TNF
TNFRSF10A
TNFRSF10B
TNFRSF18
TNFRSF1A
TNFRSF25
TNFRSF4
TNFRSF8
TNFRSF9
TNFSF10
TNFSF12
TNFSF13B
TNFSF8
TNFSF9

TNNC1
TNNI3
TNPO1
TOLLIP
TOR1B
TP53
TP63
TPK1
TPMT
TPP2
TPPP
TPT1
TRAF1
TRAF3
TRAF5
TRAF6
TRAPPC6A
TRAT1
TREM1
TREML4
TRIB2
TRIM14
TRIM22
TRIM23
TRIM25
TRIM28
TRIM3
TRIM41
TRIM5
TRIM56
TRIM69
TSC2
TSLP
TSPOAP1
TTR
TUFM
TUT4
TUT7
TXN
TXNDC9
TYK2
UBA52
UBASH3B

UBD
UBE2L3
UBQLN2
UCA1
UCK2
UGT1A1
UGT1A10
UGT1A3
UGT1A4
UGT1A5
UGT1A6
UGT1A7
UGT1A8
UGT1A9
UNC93B1
UQCRFS1
USP11
USP18
UTRN
UVRAG
VASP
VCAM1
VCP
VDR
VLDLR
VNN1
VPS28
VPS4A
VTN
WFDC1
WNK1
WNT2
WNT3A
WT1
XBP1
XCL1
XCL2
XCR1
XPO1
YBX1
YME1L1
ZBP1
ZBTB25

ZC3H12A
ZHX2
ZMPSTE24
ZNF683
ZNRD2
ZYX

| TABLE S6: INTERSECTION INFLUENZA AND PHYTOCHEMICALS GENE/PROTEIN NAMES |
|---|
| AKT1 |
| MAPK3 |
| TP53 |
| PTGS2 |
| IL6 |
| NFE2L2 |
| MMP9 |
| EGFR |
| TNF |
| CDKN1A |
| JUN |
| ESR1 |
| MAPK1 |
| GABPA |
| MAPK14 |
| STAT3 |
| EGF |
| AHSA1 |
| CRK |
| GRAP2 |
| RNF19A |
| POLDIP2 |
| IGF1 |
| FOS |
| AIMP2 |
| INS |
| MMP2 |
| CASP3 |
| BDNF |
| JUND |
| NSG1 |
| TCEAL1 |
| ERBB2 |
| HIF1A |
| HRAS |
| LEP |
| JUNB |
| CXCL8 |
| FOSB |

| |
|---|
| HMOX1 |
| CFTR |
| MT-CO2 |
| SIRT1 |
| RAF1 |
| MTOR |
| FGF2 |
| KRAS |
| SRC |
| APOE |
| CCL2 |
| MAP2K1 |
| PTEN |
| IL1B |
| CDH1 |
| RELA |
| CD44 |
| ICAM1 |
| APP |
| PPARG |
| HSPA4 |
| TNFRSF10B |
| GORASP1 |
| WNK1 |
| SYT1 |
| CAV1 |
| ABCB1 |
| CREB1 |
| TLR4 |
| IL10 |
| CXCR4 |
| NOS2 |
| PARP1 |
| CD40LG |
| IL4 |
| CRP |
| PPARA |
| MPO |
| ZHX2 |
| SPP1 |
| MTTP |

| |
|---|
| AREG |
| HMGB1 |
| CYCS |
| CXCL12 |
| MAPK8 |
| DPP4 |
| NQO2 |
| BACE1 |
| ABL1 |
| SOD1 |
| IL18 |
| NLRP3 |
| YBX1 |
| SP1 |
| ISYNA1 |
| SERPINE1 |
| TIMP1 |
| FN1 |
| NR1I2 |
| ABCA1 |
| MMP13 |
| ZNRD2 |
| HP |
| DCTN6 |
| TMED7 |
| PSMD9 |
| IFI27 |
| RNASE3 |
| MET |
| ATM |
| CASP8 |
| FASLG |
| EIF2AK3 |
| GAST |
| SMAD3 |
| COL11A2 |
| NPM1 |
| HSP90AA1 |
| CD40 |
| RPS6KA3 |
| MDM2 |

| |
|---|
| TLR2 |
| KCNH4 |
| KCNH8 |
| CASP9 |
| ALB |
| ANXA5 |
| CHP1 |
| DNMT1 |
| TNFSF10 |
| CCR7 |
| VCAM1 |
| FGF13 |
| STAT1 |
| BECN1 |
| RPS6KA1 |
| SEC14L2 |
| GLI1 |
| IL13 |
| HSPA1A |
| ALK |
| MIR34A5P |
| PGPEP1 |
| SCGB2A2 |
| FOXO1 |
| CCL11 |
| ATF3 |
| PRNP |
| CASP1 |
| TTR |
| CD274 |
| DNM1L |
| ACE |
| EZH2 |
| OSM |
| IFNG |
| GRP |
| CD4 |
| SQSTM1 |
| FGFR2 |
| MUC5AC |
| CXCL10 |

| |
|---|
| PLK1 |
| PSG1 |
| CFLAR |
| F2R |
| DAND5 |
| JAK1 |
| EIF4G1 |
| KEAP1 |
| IL2 |
| HK2 |
| CCR5 |
| UGT1A1 |
| AIM2 |
| UGT1A8 |
| KCNA5 |
| ERBB3 |
| TJP1 |
| ID1 |
| CDK1 |
| CXCL1 |
| FGFR1 |
| AGTR1 |
| MUC5B |
| STAT6 |
| SPHK1 |
| KLK3 |
| MAP3K5 |
| UTRN |
| PSMD1 |
| KLK2 |
| TAC1 |
| MBTPS1 |
| HDAC1 |
| HOOK2 |
| KIF2A |
| PECAM1 |
| IL1A |
| AIP |
| MIR146A5P |
| TNFSF13B |
| IGFBP3 |

| |
|---|
| CD9 |
| BGN |
| SNCA |
| PTK2 |
| APEX1 |
| SETX |
| RIPK3 |
| TNFRSF10A |
| DAPK2 |
| IFNA1 |
| PAK1 |
| DENR |
| MARK2 |
| FPR2 |
| UGT1A6 |
| CRMP1 |
| OCLN |
| FAS |
| CYBB |
| IL24 |
| MAP3K1 |
| DCTN4 |
| TIPARP |
| RPS6KA2 |
| NUP62 |
| TSC2 |
| EIF2AK2 |
| IL17C |
| GTF2H1 |
| FGF7 |
| KHDRBS1 |
| MX2 |
| PKLR |
| FYN |
| LET7C5P |
| GLRX |
| CD8A |
| MUC15 |
| GATD3B |
| CSF3 |
| HES1 |

| |
|---|
| PROC |
| RAB5A |
| ST14 |
| GATD3A |
| CLDN1 |
| ATG7 |
| DMBT1 |
| GPT |
| KDM1A |
| HERPUD1 |
| CNBP |
| RPS6KA5 |
| MIR1323P |
| CEBPB |
| TERT |
| SOCS3 |
| BSG |
| HEY2 |
| IRAK1 |
| CAP1 |
| F3 |
| PTGS1 |
| TRAF1 |
| ARHGEF7 |
| HNRNPK |
| JAK3 |
| RHOA |
| UBE2L3 |
| MYD88 |
| POLD1 |
| APAF1 |
| CCL5 |
| H3-2 |
| GRN |
| HEATR6 |
| XBP1 |
| IL1RL1 |
| EPCAM |
| NRP1 |
| MIR1555P |
| MIR203A |

| |
|---|
| LET7B5P |
| MIR107 |
| SDC1 |
| IL6ST |
| IL33 |
| CDC42 |
| NRG1 |
| OPRM1 |
| ALDH3A1 |
| ITGAM |
| CSF2 |
| DEFB1 |
| ABCA3 |
| MLKL |
| PKM |
| P4HB |
| ALDH1A3 |
| CTSB |
| FKBP4 |
| NFKB1 |
| LRP1 |
| KLF2 |
| LRPPRC |
| IL7 |
| REN |
| TNFRSF1A |
| CCL22 |
| IRF1 |
| TNFRSF25 |
| MAP2K4 |
| TLR5 |
| VDR |
| TRAF3 |
| HAMP |
| MIR2233P |
| MIR29A3P |
| MIR29B3P |
| DNMT3B |
| E2F1 |
| C5AR1 |
| ARG1 |

| |
|---|
| ATG5 |
| FOXP3 |
| TLR7 |
| SPATA2 |
| HTR2A |
| RPL17-C18orf32 |
| EPX |
| NR3C1 |
| C3 |
| RIPK1 |
| MIR1275 |
| MIR451A |
| RHO |
| IL3 |
| KLK1 |
| GRK2 |
| IL17RA |
| PDCD1 |
| ASCC1 |
| CDK9 |
| CXCR3 |
| MIR2045P |
| HSPA14 |
| SDCBP2 |
| HSPD1 |
| SUMO1 |
| MDK |
| MECP2 |
| RAPGEF3 |
| PTPRC |
| EIF4E |
| TRAF6 |
| NR4A1 |
| IL22 |
| PHB |
| MUC1 |
| PWAR1 |
| RALBP1 |
| SULT2A1 |
| C5 |
| GAPDH |

| |
|---|
| BCL2L2 |
| BIRC3 |
| CD80 |
| HMGCR |
| RICTOR |
| FADD |
| RPTOR |
| RBBP6 |
| PRKRA |
| CXCR2 |
| KIR3DL2 |
| TSLP |
| MAP2K3 |
| CLDN7 |
| EPRS1 |
| CSRP1 |
| FMR1 |
| PTPN1 |
| GZMB |
| PIK3R2 |
| C1QBP |
| COL1A1 |
| F2RL3 |
| NDUFA2 |
| NR0B2 |
| JAG1 |
| ARG2 |
| DNMT3A |
| FCGR2A |
| CTTN |
| COL2A1 |
| HLA-A |
| RNASE1 |
| NPY |
| XPO1 |
| DUOX2 |
| CCL4 |
| NR2C2 |
| MIR146B5P |
| PWAR4 |
| ALDH1A1 |

| |
|---|
| CD14 |
| CALR |
| PAWR |
| WT1 |
| PTPN11 |
| TNNI3 |
| PDPK1 |
| ANXA6 |
| RAC1 |
| FCGR1A |
| MAP3K7 |
| CPT2 |
| CHD6 |
| TNFSF12 |
| CXCR1 |
| ISG20 |
| NPTX1 |
| RHOD |
| XCR1 |
| STAT2 |
| LAMP1 |
| SLC22A8 |
| ATP7A |
| ANXA2 |
| UGT1A9 |
| AMPH |
| CTSE |
| PTPN22 |
| CXCL9 |
| PHGDH |
| STK4 |
| TXN |
| PADI1 |
| THBD |
| IL2RA |
| VLDLR |
| STAG1 |
| MPRIP |
| BCL6 |
| NLRC4 |
| NOLC1 |

| |
|---|
| CYLD |
| PRF1 |
| LTA |
| IFNL3 |
| MST1 |
| SERPINA1 |
| MME |
| IKBKB |
| IL32 |
| CFL1 |
| HNRNPC |
| MRC1 |
| MAP2K6 |
| CCL3 |
| CCL4L2 |
| MIF |
| TNFSF8 |
| DUSP4 |
| TNFSF9 |
| CD70 |
| PYCARD |
| SRSF1 |
| CD33 |
| RPE65 |
| TEP1 |
| GOLPH3 |
| GAL |
| UBASH3B |
| WNT3A |
| MIR200C3P |
| LRRC3 |
| SCGB3A1 |
| PPBP |
| HIF1AN |
| IL17D |
| GRM5 |
| PLG |
| CD7 |
| CD46 |
| CD28 |
| KLK4 |

| |
|---|
| MAF |
| CADM1 |
| CD86 |
| RARB |
| NANOS2 |
| GATA2 |
| TAP1 |
| C1D |
| KLRG1 |
| CCL20 |
| CD163 |
| CES1 |
| CD55 |
| XCL2 |
| GBP1 |
| SFTPA2 |
| ANXA7 |
| MED12 |
| STS |
| CCL17 |
| PDIA2 |
| VTN |
| CD69 |
| IL5 |
| AZU1 |
| ID2 |
| VASP |
| RALY |
| RAC2 |
| RARA |
| LGALS3 |
| ALDH3B2 |
| CCNA1 |
| MBD2 |
| IL1RN |
| MSR1 |
| CREBBP |
| PNO1 |
| ARCN1 |
| TP63 |
| MAPKAP1 |

| |
|---|
| TINAGL1 |
| NAT2 |
| IGF2BP1 |
| PADI4 |
| PSAT1 |
| ANXA1 |
| IL1RAPL1 |
| IFNB1 |
| DDX1 |
| RBMS3 |
| GPS1 |
| TIRAP |
| CD59 |
| PRH2 |
| DKK3 |
| MX1 |
| SFTPB |
| SFTPA1 |
| ERN1 |
| ILF3 |
| OTUD4 |
| EHMT1 |
| KLF6 |
| ERVW-1 |
| HDAC2 |
| IDO1 |
| API5 |
| CCR3 |
| FKBP5 |
| ITGAX |
| DPEP2 |
| ELANE |
| SAFB |
| DDX6 |
| PRH1 |
| SPRY1 |
| IKBKG |
| PVT1 |
| MIR1243P |
| MIR315P |

| TABLE S7: Pathways enriched (KEGG) | | | | | | | | |
|---|---|---|---|---|---|---|---|---|
| **Term_ID** | **Function** | **P-value** | **.-log10 Pvalue** | **Term Size** | **Query size** | **Intersection Size** | **Enrichment Score %** | **Positive Hits** |
| KEGG: 05417 | Lipid and atherosclerosis | 2.45e-40 | 39,6 | 209 | 435 | 76 | 36,36 | AKT1, MAPK3, TP53, IL6, NFE2L2, MMP9, TNF, JUN, MAPK1, MAPK14, STAT3, FOS, CASP3, HRAS, CXCL8, KRAS, SRC, CCL2, IL1B, RELA, ICAM1, PPARG, TNFRSF10B, TLR4, CD40LG, CYCS, MAPK8, IL18, NLRP3, ABCA1, CASP8, FASLG, EIF2AK3, HSP90AA1, CD40, TLR2, CASP9, TNFSF10, VCAM1, HSPA1A, CASP1, CXCL1, MAP3K5, PTK2, TNFRSF10A, IFNA1, FAS, CYBB, IRAK1, RHOA, MYD88, APAF1, CCL5, XBP1, CDC42, NFKB1, TNFRSF1A, MAP2K4, TRAF3, HSPD1, TRAF6, MAP2K3, CD14, PDPK1, RAC1, MAP3K7, VLDLR, IKBKB, MAP2K6, CCL3, PYCARD, IFNB1, TIRAP, ERN1, IKBKG |

| | | | | | | | | |
|---|---|---|---|---|---|---|---|---|
| KEGG: 04668 | TNF signaling pathway | 8.02e-34 | 33,1 | 110 | 435 | 52 | 47,27 | AKT1, MAPK3, PTGS2, IL6, MMP9, TNF, JUN, MAPK1, MAPK14, FOS, CASP3, JUNB, CCL2, MAP2K1, IL1B, RELA, ICAM1, CREB1, MAPK8, CASP8, VCAM1, DNM1L, CXCL10, CFLAR, CXCL1, MAP3K5, RIPK3, FAS, RPS6KA5, CEBPB, SOCS3, TRAF1, CCL5, CSF2, MLKL, NFKB1, TNFRSF1A, IRF1, MAP2K4, TRAF3, RIPK1, BIRC3, FADD, MAP2K3, JAG1, MAP3K7, LTA, IKBKB, MAP2K6, CCL20, IFNB1, IKBKG |
| KEGG: 05200 | Pathways in cancer | 1.63e-31 | 30,8 | 517 | 435 | 106 | 20,5 | AKT1, MAPK3, TP53, PTGS2, IL6, NFE2L2, MMP9, EGFR, CDKN1A, JUN, ESR1, MAPK1, STAT3, EGF, CRK, IGF1, FOS, MMP2, CASP3, ERBB2, HIF1A, HRAS, CXCL8, HMOX1, RAF1, MTOR, FGF2, KRAS, MAP2K1, PTEN, CDH1, RELA, PPARG, CXCR4, NOS2, IL4, CYCS, CXCL12, MAPK8, ABL1, SP1, FN1, MET, CASP8, FASLG, SMAD3, HSP90AA1, MDM2, CASP9, STAT1, GLI1, IL13, ALK, FOXO1, IFNG, FGFR2, F2R, JAK1, |

| | | | | | | | | |
|---|---|---|---|---|---|---|---|---|
| | | | | | | | | KEAP1, IL2, FGFR1, AGTR1, STAT6, KLK3, HDAC1, PTK2, DAPK2, IFNA1, FAS, FGF7, HES1, RPS6KA5, TERT, HEY2, TRAF1, JAK3, RHOA, APAF1, IL6ST, CDC42, NFKB1, IL7, TRAF3, E2F1, IL3, TRAF6, RALBP1, BIRC3, FADD, F2RL3, JAG1, RAC1, STAT2, STK4, IL2RA, IKBKB, WNT3A, RARB, IL5, RAC2, RARA, CCNA1, CREBBP, HDAC2, IKBKG |
| KEGG: 04620 | Toll-like receptor signaling pathway | 1.71e-31 | 30,8 | 100 | 435 | 48 | 48 | AKT1, MAPK3, IL6, TNF, JUN, MAPK1, MAPK14, FOS, CXCL8, MAP2K1, IL1B, RELA, TLR4, SPP1, MAPK8, CASP8, CD40, TLR2, STAT1, CXCL10, IFNA1, IRAK1, MYD88, CCL5, NFKB1, MAP2K4, TLR5, TRAF3, TLR7, RIPK1, TRAF6, CD80, FADD, MAP2K3, CCL4, CD14, RAC1, MAP3K7, CXCL9, IKBKB, MAP2K6, CCL3, CCL4L2, CD86, IFNB1, TIRAP, IKBKG |

| | | | | | | | | |
|---|---|---|---|---|---|---|---|---|
| KEGG: 04657 | IL-17 signaling pathway | 3.66e-30 | 29,4 | 91 | 435 | 45 | 49,45 | MAPK3, PTGS2, IL6, MMP9, TNF, JUN, MAPK1, MAPK14, FOS, CASP3, JUND, CXCL8, FOSB, CCL2, IL1B, RELA, IL4, MAPK8, MMP13, CASP8, HSP90AA1, IL13, CCL11, IFNG, MUC5AC, CXCL10, CXCL1, MUC5B, IL17C, CSF3, CEBPB, CSF2, NFKB1, TRAF3, IL17RA, TRAF6, FADD, MAP3K7, IKBKB, SRSF1, IL17D, CCL20, CCL17, IL5, IKBKG |
| KEGG: 05161 | Hepatitis B | 2.72e-28 | 27,6 | 159 | 435 | 56 | 35,22 | AKT1, MAPK3, TP53, IL6, MMP9, TNF, CDKN1A, JUN, MAPK1, MAPK14, STAT3, FOS, CASP3, HRAS, CXCL8, RAF1, KRAS, SRC, MAP2K1, RELA, CREB1, TLR4, CYCS, MAPK8, CASP8, FASLG, SMAD3, TLR2, CASP9, STAT1, JAK1, STAT6, IFNA1, FAS, MAP3K1, IRAK1, JAK3, MYD88, APAF1, NFKB1, MAP2K4, TRAF3, E2F1, TRAF6, FADD, MAP2K3, MAP3K7, STAT2, IKBKB, MAP2K6, CCNA1, CREBBP, IFNB1, TIRAP, IKBKG |

| | | | | | | | | |
|---|---|---|---|---|---|---|---|---|
| KEGG: 04060 | Cytokine-cytokine receptor interaction | 8.00e-27 | 26,1 | 281 | 435 | 72 | 25,62 | IL6, TNF, LEP, CXCL8, CCL2, IL1B, TNFRSF10B, IL10, CXCR4, CD40LG, IL4, CXCL12, IL18, FASLG, CD40, TNFSF10, CCR7, IL13, CCL11, OSM, IFNG, CD4, CXCL10, IL2, CCR5, CXCL1, IL1A, TNFSF13B, TNFRSF10A, IFNA1, FAS, IL24, IL17C, CSF3, CCL5, IL1RL1, IL6ST, IL33, CSF2, IL7, TNFRSF1A, CCL22, TNFRSF25, IL3, IL17RA, CXCR3, IL22, CXCR2, TSLP, CCL4, TNFSF12, CXCR1, XCR1, CXCL9, IL2RA, LTA, IFNL3, IL32, CCL3, CCL4L2, TNFSF8, TNFSF9, CD70, PPBP, IL17D, CCL20, CCL17, IL5, IL1RN, IFNB1, CCR3 |
| KEGG: 05164 | Influenza A | 1.09e-25 | 25 | 163 | 435 | 54 | 33,13 | AKT1, MAPK3, IL6, TNF, MAPK1, CASP3, CXCL8, RAF1, CCL2, MAP2K1, IL1B, RELA, ICAM1, TNFRSF10B, TLR4, CYCS, IL18, NLRP3, CASP8, FASLG, CASP9, TNFSF10, STAT1, CASP1, IFNG, CXCL10, JAK1, IL1A, TNFRSF10A, IFNA1, FAS, EIF2AK2, MX2, SOCS3, MYD88, APAF1, CCL5, IL33, NFKB1, TNFRSF1A, |

| | | | | | | | | |
|---|---|---|---|---|---|---|---|---|
| | | | | | | | | TRAF3, TLR7, BCL2L2, FADD, XPO1, STAT2, IKBKB, PYCARD, PLG, CREBBP, IFNB1, MX1, IKBKG |
| KEGG: 05135 | Yersinia infection | 9.40e-25 | 24 | 132 | 435 | 48 | 36,36 | AKT1, MAPK3, IL6, TNF, JUN, MAPK1, MAPK14, CRK, FOS, CXCL8, SRC, CCL2, MAP2K1, IL1B, RELA, TLR4, IL10, MAPK8, IL18, NLRP3, FN1, RPS6KA3, RPS6KA1, CASP1, CD4, IL2, PTK2, RPS6KA2, CD8A, IRAK1, ARHGEF7, RHOA, MYD88, CDC42, NFKB1, MAP2K4, TRAF6, MAP2K3, FCGR2A, RAC1, MAP3K7, NLRC4, IKBKB, MAP2K6, PYCARD, RAC2, IFNB1, IKBKG |
| KEGG: 05163 | Human cytomegalovirus infection | 3.54e-24 | 23,5 | 215 | 435 | 60 | 27,91 | AKT1, MAPK3, TP53, PTGS2, IL6, EGFR, TNF, CDKN1A, MAPK1, MAPK14, STAT3, CRK, CASP3, HRAS, CXCL8, RAF1, MTOR, KRAS, SRC, CCL2, MAP2K1, IL1B, RELA, CREB1, CXCR4, CYCS, CXCL12, SP1, CASP8, FASLG, MDM2, CASP9, JAK1, CCR5, PTK2, IFNA1, |

| | | | | | | | | |
|---|---|---|---|---|---|---|---|---|
| | | | | | | | | FAS, TSC2, RHOA, CCL5, NFKB1, TNFRSF1A, E2F1, RIPK1, FADD, CXCR2, HLA-A, CCL4, CALR, RAC1, IKBKB, MAP2K6, CCL3, CCL4L2, TAP1, RAC2, IFNB1, CCR3, IKBKG |
| KEGG: 05167 | Kaposi sarcoma-associated herpesvirus infection | 1.20e-23 | 22,9 | 191 | 435 | 56 | 29,32 | AKT1, MAPK3, TP53, PTGS2, IL6, CDKN1A, JUN, MAPK1, MAPK14, STAT3, FOS, CASP3, HIF1A, HRAS, CXCL8, RAF1, MTOR, FGF2, KRAS, SRC, MAP2K1, RELA, ICAM1, CREB1, CYCS, MAPK8, CASP8, CASP9, STAT1, BECN1, JAK1, CCR5, CXCL1, IFNA1, FAS, EIF2AK2, IL6ST, CSF2, NFKB1, TNFRSF1A, MAP2K4, TRAF3, E2F1, C3, FADD, HLA-A, RAC1, STAT2, IKBKB, MAP2K6, CD86, CREBBP, IFNB1, CCR3, IKBKG |
| KEGG: 05130 | Pathogenic Escherichia coli infection | 5.17e-22 | 21,3 | 183 | 435 | 53 | 28,96 | MAPK3, IL6, TNF, JUN, MAPK1, MAPK14, FOS, CASP3, CXCL8, SRC, IL1B, RELA, TNFRSF10B, TLR4, CYCS, MAPK8, ABL1, IL18, NLRP3, CASP8, FASLG, CASP9, TNFSF10, CASP1, F2R, TJP1, TNFRSF10A, |

| | | | | | | | | |
|---|---|---|---|---|---|---|---|---|
| | | | | | | | | PAK1, OCLN, FAS, FYN, CLDN1, IRAK1, RHOA, MYD88, CDC42, NFKB1, TNFRSF1A, TLR5, RIPK1, TRAF6, GAPDH, FADD, CLDN7, FCGR2A, CTTN, PTPN11, RAC1, MAP3K7, IKBKB, PYCARD, TIRAP, IKBKG |
| KEGG: 05160 | Hepatitis C | 1.25e-21 | 20,9 | 152 | 435 | 48 | 31,58 | AKT1, MAPK3, TP53, EGFR, TNF, CDKN1A, MAPK1, STAT3, EGF, CASP3, HRAS, RAF1, KRAS, MAP2K1, RELA, PPARA, CYCS, CASP8, FASLG, EIF2AK3, CASP9, STAT1, IFNG, CXCL10, CFLAR, JAK1, IFNA1, OCLN, FAS, EIF2AK2, MX2, CLDN1, SOCS3, APAF1, NFKB1, TNFRSF1A, TRAF3, E2F1, RIPK1, TRAF6, FADD, CLDN7, STAT2, IKBKB, IFNB1, MX1, IKBKG |
| KEGG: 05142 | Chagas disease | 1.41e-21 | 20,9 | 98 | 435 | 39 | 39,8 | AKT1, MAPK3, IL6, TNF, JUN, MAPK1, MAPK14, FOS, CXCL8, CCL2, IL1B, RELA, TLR4, IL10, NOS2, MAPK8, SERPINE1, CASP8, FASLG, TLR2, ACE, IFNG, CFLAR, IL2, FAS, IRAK1, MYD88, CCL5, NFKB1, TNFRSF1A, MAP2K4, C3, TRAF6, FADD, CALR, IKBKB, |

| | | | | | | | | |
|---|---|---|---|---|---|---|---|---|
| | | | | | | | | CCL3, IFNB1, IKBKG |
| KEGG: 05162 | Measles | 2.68e-21 | 20,6 | 135 | 435 | 45 | 33,33 | AKT1, TP53, IL6, JUN, STAT3, FOS, CASP3, IL1B, RELA, TLR4, CYCS, MAPK8, CASP8, FASLG, EIF2AK3, TLR2, CASP9, STAT1, HSPA1A, JAK1, IL2, IL1A, IFNA1, FAS, EIF2AK2, MX2, IRAK1, JAK3, MYD88, APAF1, NFKB1, TRAF3, TLR7, TRAF6, FADD, MAP3K7, STAT2, IL2RA, IKBKB, CD46, CD28, IFNB1, MX1, IKBKG |
| KEGG: 05169 | Epstein-Barr virus infection | 4.87e-21 | 20,3 | 191 | 435 | 53 | 27,75 | AKT1, TP53, IL6, TNF, CDKN1A, JUN, MAPK14, STAT3, CASP3, RELA, CD44, ICAM1, CYCS, MAPK8, CASP8, CD40, MDM2, TLR2, CASP9, STAT1, CXCL10, JAK1, PSMD1, HDAC1, IFNA1, FAS, EIF2AK2, HES1, IRAK1, JAK3, MYD88, APAF1, NFKB1, MAP2K4, TRAF3, E2F1, RIPK1, TRAF6, FADD, MAP2K3, HLA-A, CALR, RAC1, MAP3K7, STAT2, IKBKB, MAP2K6, TAP1, CCNA1, IFNB1, HDAC2, IKBKG |

| | | | | | | | | |
|---|---|---|---|---|---|---|---|---|
| KEGG: 05132 | Salmonella infection | 4.97e-21 | 20,3 | 236 | 435 | 59 | 25 | AKT1, MAPK3, IL6, TNF, JUN, MAPK1, MAPK14, FOS, CASP3, HRAS, CXCL8, RAF1, MAP2K1, IL1B, RELA, TNFRSF10B, TLR4, CYCS, MAPK8, IL18, NLRP3, DCTN6, CASP8, HSP90AA1, TLR2, TNFSF10, CASP1, RIPK3, TNFRSF10A, PAK1, DCTN4, RAB5A, IRAK1, RHOA, MYD88, CDC42, MLKL, NFKB1, TNFRSF1A, MAP2K4, TLR5, RIPK1, PTPRC, TRAF6, GAPDH, BIRC3, FADD, MAP2K3, CD14, RAC1, MAP3K7, ANXA2, TXN, NLRC4, IKBKB, MAP2K6, PYCARD, TIRAP, IKBKG |
| KEGG: 05152 | Tuberculosis | 1.22e-20 | 19,9 | 173 | 435 | 50 | 28,9 | AKT1, MAPK3, IL6, TNF, MAPK1, MAPK14, CASP3, RAF1, SRC, IL1B, RELA, CREB1, TLR4, IL10, NOS2, CYCS, MAPK8, IL18, CASP8, TLR2, CASP9, STAT1, IFNG, JAK1, SPHK1, IL1A, IFNA1, RAB5A, CEBPB, IRAK1, RHOA, MYD88, APAF1, ITGAM, NFKB1, TNFRSF1A, VDR, C3, HSPD1, TRAF6, FADD, FCGR2A, CD14, FCGR1A, MRC1, |

| | | | | | | | | |
|---|---|---|---|---|---|---|---|---|
| | | | | | | | | CREBBP, IFNB1, TIRAP, ITGAX |
| KEGG: 05235 | PD-L1 expression and PD-1 checkpoint pathway in cancer | 2.53e-20 | 19,6 | 88 | 435 | 36 | 40,91 | AKT1, MAPK3, EGFR, JUN, MAPK1, MAPK14, STAT3, EGF, FOS, HIF1A, HRAS, RAF1, MTOR, KRAS, MAP2K1, PTEN, RELA, TLR4, TLR2, STAT1, ALK, CD274, IFNG, CD4, JAK1, MYD88, NFKB1, PDCD1, TRAF6, MAP2K3, PTPN11, IKBKB, MAP2K6, CD28, TIRAP, IKBKG |
| KEGG: 05133 | Pertussis | 1.55e-19 | 18,8 | 76 | 435 | 33 | 43,42 | MAPK3, IL6, TNF, JUN, MAPK1, MAPK14, FOS, CASP3, CXCL8, IL1B, RELA, TLR4, IL10, NOS2, MAPK8, NLRP3, CASP1, IL1A, IRAK1, RHOA, MYD88, ITGAM, NFKB1, IRF1, C3, TRAF6, C5, CD14, CFL1, PYCARD, SFTPA2, TIRAP, SFTPA1 |
| KEGG: 04061 | Viral protein interaction with cytokine and | 2.40e-19 | 18,6 | 93 | 435 | 36 | 38,71 | IL6, TNF, CXCL8, CCL2, TNFRSF10B, IL10, CXCR4, CXCL12, IL18, TNFSF10, CCR7, CCL11, CXCL10, IL2, CCR5, CXCL1, TNFRSF10A, |

| | | | | | | | | |
|---|---|---|---|---|---|---|---|---|
| | cytokine receptor | | | | | | | IL24, CCL5, IL6ST, TNFRSF1A, CCL22, CXCR3, CXCR2, CCL4, CXCR1, XCR1, CXCL9, IL2RA, LTA, CCL3, CCL4L2, PPBP, CCL20, CCL17, CCR3 |
| KEGG: 04210 | Apoptosis | 3.35e-19 | 18,5 | 130 | 435 | 42 | 32,31 | AKT1, MAPK3, TP53, TNF, JUN, MAPK1, FOS, CASP3, HRAS, RAF1, KRAS, MAP2K1, RELA, TNFRSF10B, PARP1, CYCS, MAPK8, ATM, CASP8, FASLG, EIF2AK3, CASP9, TNFSF10, CFLAR, MAP3K5, TNFRSF10A, FAS, TRAF1, APAF1, CTSB, NFKB1, TNFRSF1A, RIPK1, IL3, BIRC3, FADD, GZMB, PDPK1, PRF1, IKBKB, ERN1, IKBKG |
| KEGG: 05418 | Fluid shear stress and atherosclerosis | 1.01e-17 | 17 | 134 | 435 | 41 | 30,6 | AKT1, TP53, NFE2L2, MMP9, TNF, JUN, MAPK14, FOS, MMP2, HMOX1, SRC, CCL2, IL1B, RELA, ICAM1, CAV1, MAPK8, HSP90AA1, VCAM1, IFNG, SQSTM1, KEAP1, MAP3K5, PECAM1, IL1A, PTK2, RHOA, SDC1, NFKB1, KLF2, TNFRSF1A, MAP2K4, SUMO1, RAC1, MAP3K7, TXN, THBD, IKBKB, MAP2K6, RAC2, IKBKG |

| | | | | | | | | |
|---|---|---|---|---|---|---|---|---|
| KEGG: 05171 | Coronavirus disease - COVID-19 | 1.13e-17 | 16,9 | 207 | 435 | 51 | 24,64 | MAPK3, IL6, EGFR, TNF, JUN, MAPK1, MAPK14, STAT3, FOS, CXCL8, CCL2, IL1B, RELA, TLR4, MAPK8, NLRP3, TLR2, STAT1, CASP1, ACE, CXCL10, JAK1, IL2, AGTR1, IFNA1, CYBB, EIF2AK2, MX2, CSF3, IRAK1, MYD88, NRP1, IL6ST, CSF2, NFKB1, TNFRSF1A, TRAF3, C5AR1, TLR7, RPL17-C18orf32, C3, TRAF6, C5, FCGR2A, MAP3K7, STAT2, IKBKB, IFNB1, MX1, IKBKG |
| KEGG: 05170 | Human immunodeficiency virus 1 infection | 1.13e-17 | 16,9 | 207 | 435 | 51 | 24,64 | AKT1, MAPK3, TNF, JUN, MAPK1, MAPK14, CRK, FOS, CASP3, HRAS, RAF1, MTOR, KRAS, MAP2K1, RELA, TLR4, CXCR4, CYCS, MAPK8, ATM, CASP8, FASLG, TLR2, CASP9, CD4, CCR5, CDK1, PTK2, IFNA1, PAK1, FAS, IRAK1, MYD88, NFKB1, TNFRSF1A, RIPK1, TRAF6, FADD, MAP2K3, HLA-A, CALR, RAC1, MAP3K7, IKBKB, CFL1, MAP2K6, TAP1, RAC2, IFNB1, IKBKG |

| | | | | | | | | |
|---|---|---|---|---|---|---|---|---|
| KEGG: 04062 | Chemokine signaling pathway | 2.06e-17 | 16,7 | 179 | 435 | 47 | 26,26 | AKT1, MAPK3, MAPK1, STAT3, CRK, HRAS, CXCL8, RAF1, KRAS, SRC, CCL2, MAP2K1, RELA, CXCR4, CXCL12, CCR7, STAT1, CCL11, CXCL10, CCR5, CXCL1, PTK2, PAK1, JAK3, RHOA, CCL5, CDC42, NFKB1, CCL22, GRK2, CXCR3, CXCR2, CCL4, RAC1, CXCR1, XCR1, STAT2, CXCL9, IKBKB, CCL3, CCL4L2, PPBP, CCL20, CCL17, RAC2, CCR3, IKBKG |
| KEGG: 04010 | MAPK signaling pathway | 2.87e-17 | 16,5 | 286 | 435 | 60 | 20,98 | AKT1, MAPK3, TP53, EGFR, TNF, JUN, MAPK1, MAPK14, EGF, CRK, IGF1, FOS, INS, CASP3, BDNF, JUND, ERBB2, HRAS, RAF1, FGF2, KRAS, MAP2K1, IL1B, RELA, AREG, MAPK8, MET, FASLG, RPS6KA3, RPS6KA1, HSPA1A, FGFR2, ERBB3, FGFR1, MAP3K5, IL1A, PAK1, FAS, MAP3K1, RPS6KA2, FGF7, RPS6KA5, IRAK1, MYD88, CDC42, NFKB1, TNFRSF1A, MAP2K4, TRAF6, NR4A1, MAP2K3, CD14, RAC1, MAP3K7, STK4, IKBKB, MAP2K6, DUSP4, RAC2, IKBKG |

| | | | | | | | | |
|---|---|---|---|---|---|---|---|---|
| KEGG: 04621 | NOD-like receptor signaling pathway | 4.90e-17 | 16,3 | 175 | 435 | 46 | 26,29 | MAPK3, IL6, TNF, JUN, MAPK1, MAPK14, CXCL8, CCL2, IL1B, RELA, TLR4, MAPK8, IL18, NLRP3, CASP8, HSP90AA1, STAT1, CASP1, DNM1L, JAK1, AIM2, CXCL1, RIPK3, IFNA1, CYBB, RHOA, MYD88, CCL5, CTSB, NFKB1, TRAF3, ATG5, RIPK1, TRAF6, BIRC3, FADD, MAP3K7, STAT2, TXN, NLRC4, IKBKB, PYCARD, GBP1, IFNB1, IKBKG |
| KEGG: 04936 | Alcoholic liver disease | 8.23e-17 | 16,1 | 141 | 435 | 41 | 29,08 | AKT1, IL6, TNF, MAPK14, CASP3, CXCL8, SIRT1, IL1B, RELA, TLR4, PPARA, MAPK8, CASP8, FASLG, FOXO1, CXCL1, MAP3K5, IFNA1, FAS, IRAK1, MYD88, NFKB1, TNFRSF1A, MAP2K4, TRAF3, C5AR1, C3, RIPK1, IL17RA, TRAF6, C5, FADD, MAP2K3, CD14, MAP3K7, IKBKB, MAP2K6, IFNB1, TIRAP, IKBKG |
| KEGG: 04660 | T cell receptor signaling pathway | 8.78e-17 | 16,1 | 102 | 435 | 35 | 34,31 | AKT1, MAPK3, TNF, JUN, MAPK1, MAPK14, GRAP2, FOS, HRAS, RAF1, KRAS, MAP2K1, RELA, IL10, CD40LG, IL4, MAPK8, IFNG, CD4, IL2, PAK1, FYN, CD8A, |

| | | | | | | | | |
|---|---|---|---|---|---|---|---|---|
| | | | | | | | | RHOA, CDC42, CSF2, NFKB1, PDCD1, PTPRC, PDPK1, MAP3K7, IKBKB, CD28, IL5, IKBKG |
| KEGG: 04625 | C-type lectin receptor signaling pathway | 8.7 8e-17 | 16,1 | 10 2 | 435 | 35 | 34,31 | AKT1, MAPK3, PTGS2, IL6, TNF, JUN, MAPK1, MAPK14, HRAS, RAF1, KRAS, SRC, IL1B, RELA, IL10, MAPK8, NLRP3, CASP8, MDM2, STAT1, CASP1, IL2, PAK1, RHOA, NFKB1, CCL22, IRF1, PTPN11, STAT2, CYLD, IKBKB, PYCARD, IL17D, CCL17, IKBKG |
| KEGG: 05145 | Toxoplas mosis | 5.1 1e-16 | 15,3 | 10 7 | 435 | 35 | 32,71 | AKT1, MAPK3, TNF, MAPK1, MAPK14, STAT3, CASP3, RELA, TLR4, IL10, NOS2, CD40LG, CYCS, MAPK8, CASP8, CD40, TLR2, CASP9, STAT1, HSPA1A, IFNG, JAK1, CCR5, IRAK1, MYD88, NFKB1, TNFRSF1A, TRAF6, BIRC3, MAP2K3, PDPK1, MAP3K7, IKBKB, MAP2K6, IKBKG |
| KEGG: 04064 | NF-kappa B signaling pathway | 7.9 9e-16 | 15,1 | 10 2 | 435 | 34 | 33,33 | PTGS2, TNF, CXCL8, IL1B, RELA, ICAM1, TLR4, PARP1, CD40LG, CXCL12, ATM, CD40, VCAM1, CFLAR, CXCL1, TNFSF13B, IRAK1, TRAF1, MYD88, NFKB1, TNFRSF1A, TRAF3, |

| | | | | | | | | |
|---|---|---|---|---|---|---|---|---|
| | | | | | | | | RIPK1, TRAF6, BIRC3, CCL4, CD14, MAP3K7, CYLD, LTA, IKBKB, CCL4L2, TIRAP, IKBKG |
| KEGG: 04217 | Necroptosis | 1.68e-15 | 14,8 | 152 | 435 | 41 | 26,97 | TNF, STAT3, IL1B, TNFRSF10B, TLR4, PARP1, HMGB1, MAPK8, NLRP3, CASP8, FASLG, HSP90AA1, TNFSF10, STAT1, CASP1, DNM1L, IFNG, SQSTM1, CFLAR, JAK1, STAT6, IL1A, RIPK3, TNFRSF10A, IFNA1, FAS, CYBB, EIF2AK2, JAK3, IL33, MLKL, TNFRSF1A, SPATA2, RIPK1, BIRC3, FADD, STAT2, CYLD, PYCARD, IFNB1 |
| KEGG: 05134 | Legionellosis | 2.10e-15 | 14,7 | 54 | 435 | 25 | 46,3 | IL6, TNF, CASP3, CXCL8, IL1B, RELA, TLR4, CYCS, IL18, CASP8, TLR2, CASP9, HSPA1A, CASP1, CXCL1, MYD88, APAF1, ITGAM, NFKB1, TLR5, C3, HSPD1, CD14, NLRC4, PYCARD |
| KEGG: 04933 | AGE-RAGE signaling pathway in diabetic complications | 2.49e-15 | 14,6 | 99 | 435 | 33 | 33,33 | AKT1, MAPK3, IL6, TNF, JUN, MAPK1, MAPK14, STAT3, MMP2, CASP3, HRAS, CXCL8, KRAS, CCL2, IL1B, RELA, ICAM1, MAPK8, SERPINE1, FN1, SMAD3, VCAM1, STAT1, FOXO1, |

| | | | | | | | | |
|---|---|---|---|---|---|---|---|---|
| | | | | | | | | AGTR1, IL1A, CYBB, F3, CDC42, NFKB1, COL1A1, RAC1, THBD |
| KEGG: 05215 | Prostate cancer | 2.6 6e-15 | 14,6 | 93 | 435 | 32 | 34,41 | AKT1, MAPK3, TP53, MMP9, EGFR, CDKN1A, MAPK1, EGF, IGF1, INS, ERBB2, HRAS, RAF1, MTOR, KRAS, MAP2K1, PTEN, RELA, CREB1, HSP90AA1, MDM2, CASP9, FOXO1, FGFR2, FGFR1, KLK3, NFKB1, E2F1, PDPK1, IKBKB, CREBBP, IKBKG |
| KEGG: 05140 | Leishmani asis | 4.3 6e-15 | 14,4 | 71 | 435 | 28 | 39,44 | MAPK3, PTGS2, TNF, JUN, MAPK1, MAPK14, FOS, IL1B, RELA, TLR4, IL10, NOS2, IL4, TLR2, STAT1, IFNG, JAK1, IL1A, CYBB, IRAK1, MYD88, ITGAM, NFKB1, C3, TRAF6, FCGR2A, FCGR1A, MAP3K7 |
| KEGG: 05131 | Shigellosis | 8.5 5e-15 | 14,1 | 23 9 | 435 | 51 | 21,34 | AKT1, MAPK3, TP53, EGFR, TNF, JUN, MAPK1, MAPK14, CRK, CXCL8, MTOR, SRC, IL1B, RELA, CD44, TLR4, CYCS, MAPK8, IL18, NLRP3, ATM, MDM2, BECN1, FOXO1, CASP1, SQSTM1, HK2, PTK2, RPS6KA5, RHOA, MYD88, CCL5, CDC42, CSF2, NFKB1, TNFRSF1A, TLR5, ATG5, |

| | | | | | | | | |
|---|---|---|---|---|---|---|---|---|
| | | | | | | | | C3, RIPK1, TRAF6, RPTOR, CTTN, CD14, RAC1, MAP3K7, NLRC4, IKBKB, PYCARD, IFNB1, IKBKG |
| KEGG: 04380 | Osteoclast differentiation | 1.31e-14 | 13,9 | 124 | 435 | 36 | 29,03 | AKT1, MAPK3, TNF, JUN, MAPK1, MAPK14, FOS, JUND, JUNB, FOSB, MAP2K1, IL1B, RELA, PPARG, CREB1, MAPK8, STAT1, IFNG, SQSTM1, JAK1, IL1A, FYN, SOCS3, NFKB1, TNFRSF1A, TRAF6, FCGR2A, RAC1, FCGR1A, MAP3K7, STAT2, CYLD, IKBKB, MAP2K6, IFNB1, IKBKG |
| KEGG: 05165 | Human papilloma virus infection | 1.75e-14 | 13,8 | 315 | 435 | 59 | 18,73 | AKT1, MAPK3, TP53, PTGS2, EGFR, TNF, CDKN1A, MAPK1, EGF, CASP3, HRAS, RAF1, MTOR, KRAS, MAP2K1, PTEN, RELA, CREB1, SPP1, FN1, ATM, CASP8, FASLG, MDM2, STAT1, FOXO1, JAK1, HDAC1, PTK2, IFNA1, FAS, TSC2, EIF2AK2, MX2, HES1, TERT, HEY2, CDC42, PKM, NFKB1, TNFRSF1A, IRF1, TRAF3, E2F1, FADD, COL1A1, JAG1, COL2A1, HLA-A, STAT2, IKBKB, |

| | | | | | | | | |
|---|---|---|---|---|---|---|---|---|
| | | | | | | | | WNT3A, CCNA1, CREBBP, IFNB1, MX1, HDAC2, IKBKG |
| KEGG: 05205 | Proteoglycans in cancer | 7.90e-14 | 13,1 | 200 | 435 | 45 | 22,5 | AKT1, MAPK3, TP53, MMP9, EGFR, TNF, CDKN1A, ESR1, MAPK1, MAPK14, STAT3, IGF1, MMP2, CASP3, ERBB2, HIF1A, HRAS, RAF1, MTOR, FGF2, KRAS, SRC, MAP2K1, CD44, CAV1, TLR4, FN1, MET, FASLG, MDM2, TLR2, ERBB3, FGFR1, PTK2, PAK1, FAS, RHOA, SDC1, CDC42, COL1A1, CTTN, PTPN11, PDPK1, RAC1, WNT3A |
| KEGG: 05212 | Pancreatic cancer | 1.65e-13 | 12,8 | 74 | 435 | 27 | 36,49 | AKT1, MAPK3, TP53, EGFR, CDKN1A, MAPK1, STAT3, EGF, ERBB2, RAF1, MTOR, KRAS, MAP2K1, RELA, MAPK8, SMAD3, CASP9, STAT1, JAK1, CDC42, NFKB1, E2F1, RALBP1, RAC1, IKBKB, RAC2, IKBKG |

| | | | | | | | | |
|---|---|---|---|---|---|---|---|---|
| KEGG: 04151 | PI3K-Akt signaling pathway | 2.29e-13 | 12,6 | 342 | 435 | 60 | 17,54 | AKT1, MAPK3, TP53, IL6, EGFR, CDKN1A, MAPK1, EGF, IGF1, INS, BDNF, ERBB2, HRAS, RAF1, MTOR, FGF2, KRAS, MAP2K1, PTEN, RELA, CREB1, TLR4, IL4, SPP1, AREG, FN1, MET, FASLG, HSP90AA1, MDM2, TLR2, CASP9, OSM, FGFR2, F2R, JAK1, IL2, ERBB3, FGFR1, PTK2, IFNA1, TSC2, FGF7, CSF3, JAK3, NFKB1, IL7, IL3, EIF4E, NR4A1, RPTOR, COL1A1, COL2A1, PDPK1, RAC1, IL2RA, IKBKB, IFNB1, IKBKG |
| KEGG: 04630 | JAK-STAT signaling pathway | 3.58e-13 | 12,4 | 159 | 435 | 39 | 24,53 | AKT1, IL6, EGFR, CDKN1A, STAT3, EGF, HRAS, LEP, RAF1, MTOR, IL10, IL4, STAT1, IL13, OSM, IFNG, JAK1, IL2, STAT6, IFNA1, IL24, CSF3, SOCS3, JAK3, IL6ST, CSF2, IL7, IL3, IL22, TSLP, PTPN11, STAT2, IL2RA, IFNL3, IL17D, IL5, CREBBP, IFNB1 |
| KEGG: 05321 | Inflammatory bowel disease | 8.82e-13 | 12,1 | 61 | 435 | 24 | 39,34 | IL6, TNF, JUN, STAT3, IL1B, RELA, TLR4, IL10, IL4, IL18, SMAD3, TLR2, STAT1, IL13, IFNG, IL2, STAT6, IL1A, NFKB1, TLR5, FOXP3, IL22, |

| | | | | | | | | |
|---|---|---|---|---|---|---|---|---|
| | | | | | | | | MAF, IL5 |
| KEGG: 05219 | Bladder cancer | 1.04e-12 | 12 | 41 | 435 | 20 | 48,78 | MAPK3, TP53, MMP9, EGFR, CDKN1A, MAPK1, EGF, MMP2, ERBB2, HRAS, CXCL8, RAF1, KRAS, SRC, MAP2K1, CDH1, MDM2, DAPK2, RPS6KA5, E2F1 |
| KEGG: 04659 | Th17 cell differentiation | 6.15e-12 | 11,2 | 104 | 435 | 30 | 28,85 | MAPK3, IL6, JUN, MAPK1, MAPK14, STAT3, FOS, HIF1A, MTOR, IL1B, RELA, IL4, MAPK8, SMAD3, HSP90AA1, STAT1, IFNG, CD4, JAK1, IL2, STAT6, JAK3, IL6ST, NFKB1, FOXP3, IL22, IL2RA, IKBKB, RARA, IKBKG |
| KEGG: 04722 | Neurotrophin signaling pathway | 1.78e-11 | 10,8 | 115 | 435 | 31 | 26,96 | AKT1, MAPK3, TP53, JUN, MAPK1, MAPK14, CRK, BDNF, HRAS, RAF1, KRAS, MAP2K1, RELA, MAPK8, ABL1, FASLG, RPS6KA3, RPS6KA1, MAP3K5, MAP3K1, RPS6KA2, RPS6KA5, IRAK1, RHOA, CDC42, NFKB1, TRAF6, PTPN11, PDPK1, RAC1, IKBKB |
| KEGG: 04068 | FoxO signaling pathway | 6.94e-11 | 10,2 | 128 | 435 | 32 | 25 | AKT1, MAPK3, IL6, EGFR, CDKN1A, MAPK1, MAPK14, STAT3, EGF, IGF1, INS, HRAS, SIRT1, RAF1, |

| | | | | | | | | |
|---|---|---|---|---|---|---|---|---|
| | | | | | | | | KRAS, MAP2K1, PTEN, IL10, MAPK8, ATM, FASLG, SMAD3, MDM2, TNFSF10, FOXO1, PLK1, KLF2, PDPK1, STK4, BCL6, IKBKB, CREBBP |
| KEGG: 05144 | Malaria | 7.12e-11 | 10,1 | 49 | 435 | 20 | 40,82 | IL6, TNF, CXCL8, CCL2, IL1B, ICAM1, TLR4, IL10, CD40LG, IL18, MET, CD40, TLR2, VCAM1, IFNG, PECAM1, CSF3, MYD88, SDC1, LRP1 |
| KEGG: 04932 | Non-alcoholic fatty liver disease | 2.71e-10 | 9,6 | 142 | 435 | 33 | 23,24 | AKT1, IL6, TNF, JUN, MAPK14, FOS, INS, CASP3, LEP, CXCL8, MT-CO2, IL1B, RELA, PPARG, PPARA, CYCS, MAPK8, CASP8, FASLG, EIF2AK3, MAP3K5, IL1A, FAS, PKLR, SOCS3, XBP1, CDC42, NFKB1, TNFRSF1A, NDUFA2, RAC1, IKBKB, ERN1 |
| KEGG: 01522 | Endocrine resistance | 3.00e-10 | 9,5 | 90 | 435 | 26 | 28,89 | AKT1, MAPK3, TP53, MMP9, EGFR, CDKN1A, JUN, ESR1, MAPK1, MAPK14, IGF1, FOS, MMP2, ERBB2, HRAS, RAF1, MTOR, KRAS, SRC, MAP2K1, MAPK8, SP1, MDM2, PTK2, E2F1, JAG1 |

| | | | | | | | | |
|---|---|---|---|---|---|---|---|---|
| KEGG: 05166 | Human T-cell leukemia virus 1 infection | 3.01e-10 | 9,5 | 211 | 435 | 41 | 19,43 | AKT1, MAPK3, TP53, IL6, TNF, CDKN1A, JUN, MAPK1, FOS, HRAS, KRAS, MAP2K1, PTEN, RELA, ICAM1, CREB1, MAPK8, ATM, SMAD3, CD40, CD4, JAK1, IL2, MAP3K1, TERT, JAK3, NRP1, CSF2, NFKB1, TNFRSF1A, MAP2K4, E2F1, HLA-A, XPO1, CALR, IL2RA, LTA, IKBKB, CCNA1, CREBBP, IKBKG |
| KEGG: 04664 | Fc epsilon RI signaling pathway | 3.04e-10 | 9,5 | 64 | 435 | 22 | 34,38 | AKT1, MAPK3, TNF, MAPK1, MAPK14, HRAS, RAF1, KRAS, MAP2K1, IL4, MAPK8, IL13, FYN, CSF2, MAP2K4, IL3, MAP2K3, PDPK1, RAC1, MAP2K6, IL5, RAC2 |
| KEGG: 05202 | Transcriptional misregulation in cancer | 9.11e-10 | 9 | 182 | 435 | 37 | 20,33 | TP53, IL6, MMP9, CDKN1A, IGF1, CXCL8, RELA, PPARG, MPO, SP1, MET, ATM, CD40, MDM2, FOXO1, HDAC1, IGFBP3, PTK2, CEBPB, TRAF1, ITGAM, CSF2, NFKB1, IL3, CDK9, BIRC3, GZMB, CD14, WT1, FCGR1A, BCL6, MAF, CD86, RARA, CCNA1, HDAC2, ELANE |
| KEGG: 05330 | Allograft rejection | 1.07e-09 | 9 | 34 | 435 | 16 | 47,06 | TNF, IL10, CD40LG, IL4, FASLG, CD40, IFNG, IL2, FAS, CD80, GZMB, HLA- |

| | | | | | | | | |
|---|---|---|---|---|---|---|---|---|
| | | | | | | | | A, PRF1, CD28, CD86, IL5 |
| KEGG: 05120 | Epithelial cell signaling in Helicobacter pylori infection | 1.70e-09 | 8,8 | 69 | 435 | 22 | 31,88 | EGFR, JUN, MAPK14, CASP3, CXCL8, SRC, RELA, MAPK8, MET, TJP1, CXCL1, PAK1, CCL5, CDC42, NFKB1, MAP2K4, CXCR2, PTPN11, RAC1, CXCR1, IKBKB, IKBKG |
| KEGG: 01521 | EGFR tyrosine kinase inhibitor resistance | 1.95e-09 | 8,7 | 76 | 435 | 23 | 30,26 | AKT1, MAPK3, IL6, EGFR, MAPK1, STAT3, EGF, IGF1, ERBB2, HRAS, RAF1, MTOR, FGF2, KRAS, SRC, MAP2K1, PTEN, MET, FGFR2, JAK1, ERBB3, NRG1, EIF4E |
| KEGG: 05221 | Acute myeloid leukemia | 3.75e-09 | 8,4 | 65 | 435 | 21 | 32,31 | AKT1, MAPK3, MAPK1, STAT3, HRAS, RAF1, MTOR, KRAS, MAP2K1, RELA, MPO, ITGAM, CSF2, NFKB1, IL3, CD14, FCGR1A, IKBKB, RARA, CCNA1, IKBKG |
| KEGG: 04066 | HIF-1 signaling pathway | 4.73e-09 | 8,3 | 108 | 435 | 27 | 25 | AKT1, MAPK3, IL6, EGFR, CDKN1A, MAPK1, STAT3, EGF, IGF1, INS, ERBB2, HIF1A, HMOX1, MTOR, MAP2K1, RELA, TLR4, NOS2, SERPINE1, TIMP1, IFNG, HK2, CYBB, NFKB1, EIF4E, GAPDH, CREBBP |
| KEGG: 04623 | Cytosolic DNA- | 5.84e- | 8,2 | 60 | 435 | 20 | 33,33 | IL6, IL1B, RELA, IL18, CASP1, CXCL10, AIM2, |

| | | | | | | | | |
|---|---|---|---|---|---|---|---|---|
| | sensing pathway | 09 | | | | | | RIPK3, IFNA1, CCL5, IL33, NFKB1, RIPK1, CCL4, IKBKB, CCL4L2, PYCARD, IFNB1, IKBKG |
| KEGG: 04650 | Natural killer cell mediated cytotoxicit y | 6.2 8e-09 | 8,2 | 11 7 | 435 | 28 | 23,93 | MAPK3, TNF, MAPK1, CASP3, HRAS, RAF1, KRAS, MAP2K1, ICAM1, TNFRSF10B, FASLG, TNFSF10, IFNG, TNFRSF10A, IFNA1, PAK1, FAS, FYN, CSF2, KIR3DL2, GZMB, HLA-A, PTPN11, RAC1, PRF1, RAC2, IFNB1 |
| KEGG: 05203 | Viral carcinogen esis | 6.6 0e-09 | 8,2 | 19 4 | 435 | 37 | 19,07 | MAPK3, TP53, CDKN1A, JUN, MAPK1, STAT3, CASP3, HRAS, KRAS, SRC, RELA, CREB1, CASP8, MDM2, JAK1, CCR5, CDK1, HDAC1, EIF2AK2, GTF2H1, TRAF1, HNRNPK, JAK3, RHOA, IL6ST, CDC42, PKM, NFKB1, TRAF3, C3, HLA-A, RAC1, CCNA1, CREBBP, HDAC2, CCR3, IKBKG |
| KEGG: 04012 | ErbB signaling pathway | 8.3 5e-09 | 8,1 | 81 | 435 | 23 | 28,4 | AKT1, MAPK3, EGFR, CDKN1A, JUN, MAPK1, EGF, CRK, ERBB2, HRAS, RAF1, MTOR, KRAS, SRC, MAP2K1, AREG, MAPK8, ABL1, ERBB3, PTK2, PAK1, NRG1, MAP2K4 |
| KEGG: 05416 | Viral myocarditi | 8.8 0e- | 8,1 | 55 | 435 | 19 | 34,55 | CASP3, ICAM1, CAV1, CD40LG, CYCS, ABL1, |

| | | | | | | | | |
|---|---|---|---|---|---|---|---|---|
| | s | 09 | | | | | | CASP8, CD40, CASP9, EIF4G1, FYN, CD80, HLA-A, RAC1, PRF1, CD28, CD86, CD55, RAC2 |
| KEGG: 04218 | Cellular senescence | 9.6 3e-09 | 8 | 15 2 | 435 | 32 | 21,05 | AKT1, MAPK3, TP53, IL6, CDKN1A, MAPK1, MAPK14, HRAS, CXCL8, SIRT1, RAF1, MTOR, KRAS, MAP2K1, PTEN, RELA, SERPINE1, ATM, SMAD3, MDM2, FOXO1, SQSTM1, CDK1, IL1A, IGFBP3, TSC2, NFKB1, E2F1, MAP2K3, HLA-A, MAP2K6, CCNA1 |
| KEGG: 04622 | RIG-I-like receptor signaling pathway | 9.8 7e-09 | 8 | 68 | 435 | 21 | 30,88 | TNF, MAPK14, CXCL8, RELA, MAPK8, CASP8, CXCL10, IFNA1, MAP3K1, NFKB1, TRAF3, ATG5, RIPK1, TRAF6, FADD, MAP3K7, CYLD, IKBKB, IFNB1, IKBKG |
| KEGG: 05210 | Colorectal cancer | 1.4 4e-08 | 7,8 | 83 | 435 | 23 | 27,71 | AKT1, MAPK3, TP53, EGFR, CDKN1A, JUN, MAPK1, EGF, FOS, CASP3, HRAS, RAF1, MTOR, KRAS, MAP2K1, AREG, CYCS, MAPK8, SMAD3, CASP9, RHOA, RAC1, RAC2 |
| KEGG: 04940 | Type I diabetes mellitus | 2.2 2e-08 | 7,7 | 40 | 435 | 16 | 40 | TNF, INS, IL1B, FASLG, IFNG, IL2, IL1A, FAS, HSPD1, CD80, GZMB, HLA-A, PRF1, LTA, CD28, CD86 |

| | | | | | | | | |
|---|---|---|---|---|---|---|---|---|
| KEGG: 05223 | Non-small cell lung cancer | 2.45e-08 | 7,6 | 71 | 435 | 21 | 29,58 | AKT1, MAPK3, TP53, EGFR, CDKN1A, MAPK1, STAT3, EGF, ERBB2, HRAS, RAF1, KRAS, MAP2K1, MET, CASP9, ALK, JAK3, E2F1, PDPK1, STK4, RARB |
| KEGG: 04658 | Th1 and Th2 cell differentiation | 3.16e-08 | 7,5 | 86 | 435 | 23 | 26,74 | MAPK3, JUN, MAPK1, MAPK14, FOS, RELA, IL4, MAPK8, STAT1, IL13, IFNG, CD4, JAK1, IL2, STAT6, JAK3, NFKB1, JAG1, IL2RA, IKBKB, MAF, IL5, IKBKG |
| KEGG: 05323 | Rheumatoid arthritis | 3.16e-08 | 7,5 | 86 | 435 | 23 | 26,74 | IL6, TNF, JUN, FOS, CXCL8, CCL2, IL1B, ICAM1, TLR4, CXCL12, IL18, TLR2, IFNG, CXCL1, IL1A, TNFSF13B, CCL5, CSF2, CD80, CCL3, CD28, CD86, CCL20 |
| KEGG: 05206 | MicroRNAs in cancer | 4.02e-08 | 7,4 | 306 | 435 | 47 | 15,36 | MAPK3, TP53, PTGS2, MMP9, EGFR, CDKN1A, MAPK1, STAT3, CRK, CASP3, ERBB2, HRAS, HMOX1, SIRT1, RAF1, MTOR, KRAS, MAP2K1, PTEN, CD44, ABCB1, ABL1, MET, ATM, MDM2, DNMT1, EZH2, ERBB3, HDAC1, RPS6KA5, HNRNPK, RHOA, MIR203A, MIR107, NFKB1, |

| | | | | | | | | |
|---|---|---|---|---|---|---|---|---|
| | | | | | | | | DNMT3B, E2F1, MIR451A, BCL2L2, RPTOR, DNMT3A, IKBKB, WNT3A, CREBBP, TP63, IGF2BP1, HDAC2 |
| KEGG: 05220 | Chronic myeloid leukemia | 4.36e-08 | 7,4 | 73 | 435 | 21 | 28,77 | AKT1, MAPK3, TP53, CDKN1A, MAPK1, CRK, HRAS, RAF1, KRAS, MAP2K1, RELA, ABL1, SMAD3, MDM2, HDAC1, NFKB1, E2F1, PTPN11, IKBKB, HDAC2, IKBKG |
| KEGG: 05332 | Graft-versus-host disease | 6.62e-08 | 7,2 | 37 | 435 | 15 | 40,54 | IL6, TNF, IL1B, FASLG, IFNG, IL2, IL1A, FAS, CD80, KIR3DL2, GZMB, HLA-A, PRF1, CD28, CD86 |
| KEGG: 04640 | Hematopoietic cell lineage | 1.08e-07 | 7 | 91 | 435 | 23 | 25,27 | IL6, TNF, IL1B, CD44, IL4, CD4, IL1A, CD9, CD8A, CSF3, ITGAM, CSF2, IL7, IL3, CD14, FCGR1A, IL2RA, MME, CD33, CD7, CD55, IL5, CD59 |
| KEGG: 01524 | Platinum drug resistance | 1.32e-07 | 6,9 | 70 | 435 | 20 | 28,57 | AKT1, MAPK3, TP53, CDKN1A, MAPK1, CASP3, ERBB2, CYCS, ATM, CASP8, FASLG, MDM2, CASP9, MAP3K5, FAS, APAF1, BIRC3, FADD, PDPK1, ATP7A |
| KEGG: 05218 | Melanoma | 1.32e-07 | 6,9 | 70 | 435 | 20 | 28,57 | AKT1, MAPK3, TP53, EGFR, CDKN1A, MAPK1, EGF, IGF1, |

| | | | | | | | | |
|---|---|---|---|---|---|---|---|---|
| | | | | | | | | HRAS, RAF1, FGF2, KRAS, MAP2K1, PTEN, CDH1, MET, MDM2, FGFR1, FGF7, E2F1 |
| KEGG: 04613 | Neutrophil extracellular trap formation | 3.92e-07 | 6,4 | 184 | 435 | 33 | 17,93 | AKT1, MAPK3, MAPK1, MAPK14, RAF1, MTOR, SRC, MAP2K1, RELA, TLR4, MPO, HMGB1, TLR2, CASP1, HDAC1, FPR2, CYBB, ATG7, ITGAM, NFKB1, C5AR1, TLR7, C3, C5, FCGR2A, RAC1, FCGR1A, MAP3K7, AZU1, RAC2, PADI4, HDAC2, ELANE |
| KEGG: 04917 | Prolactin signaling pathway | 3.97e-07 | 6,4 | 67 | 435 | 19 | 28,36 | AKT1, MAPK3, ESR1, MAPK1, MAPK14, STAT3, FOS, INS, HRAS, RAF1, KRAS, SRC, MAP2K1, RELA, MAPK8, STAT1, SOCS3, NFKB1, IRF1 |
| KEGG: 04935 | Growth hormone synthesis, secretion and action | 4.03e-07 | 6,4 | 113 | 435 | 25 | 22,12 | AKT1, MAPK3, MAPK1, MAPK14, STAT3, CRK, IGF1, FOS, HRAS, JUNB, RAF1, MTOR, KRAS, MAP2K1, CREB1, MAPK8, STAT1, IGFBP3, PTK2, MAP3K1, SOCS3, MAP2K4, MAP2K3, MAP2K6, CREBBP |
| KEGG: 05222 | Small cell lung cancer | 4.97e-07 | 6,3 | 90 | 435 | 22 | 24,44 | AKT1, TP53, PTGS2, CDKN1A, CASP3, PTEN, RELA, NOS2, CYCS, FN1, CASP9, PTK2, TRAF1, APAF1, NFKB1, TRAF3, E2F1, TRAF6, |

| | | | | | | | | |
|---|---|---|---|---|---|---|---|---|
| | | | | | | | | BIRC3, IKBKB, RARB, IKBKG |
| KEGG: 05208 | Chemical carcinogenesis - reactive oxygen species | 5.72e-07 | 6,2 | 206 | 435 | 35 | 16,99 | AKT1, MAPK3, NFE2L2, EGFR, JUN, MAPK1, MAPK14, EGF, FOS, HIF1A, HRAS, HMOX1, MT-CO2, RAF1, KRAS, SRC, MAP2K1, PTEN, RELA, MAPK8, ABL1, SOD1, MET, KEAP1, MAP3K5, PTK2, NFKB1, MAP2K4, PTPN1, NDUFA2, PTPN11, PDPK1, RAC1, IKBKB, IKBKG |
| KEGG: 05320 | Autoimmune thyroid disease | 7.29e-07 | 6,1 | 49 | 435 | 16 | 32,65 | IL10, CD40LG, IL4, FASLG, CD40, IL2, IFNA1, FAS, CD80, GZMB, HLA-A, PRF1, CD28, CD86, IL5 |
| KEGG: 04140 | Autophagy - animal | 9.81e-07 | 6 | 135 | 435 | 27 | 20 | AKT1, MAPK3, MAPK1, INS, HIF1A, HRAS, RAF1, MTOR, KRAS, MAP2K1, PTEN, HMGB1, MAPK8, EIF2AK3, BECN1, SQSTM1, CFLAR, DAPK2, TSC2, ATG7, CTSB, ATG5, TRAF6, RPTOR, PDPK1, MAP3K7, ERN1 |

| | | | | | | | | |
|---|---|---|---|---|---|---|---|---|
| KEGG: 05010 | Alzheimer disease | 1.63e-06 | 5,8 | 353 | 435 | 48 | 13,6 | AKT1, MAPK3, PTGS2, IL6, TNF, MAPK1, INS, CASP3, HRAS, MT-CO2, RAF1, MTOR, KRAS, APOE, MAP2K1, IL1B, RELA, APP, NOS2, CYCS, MAPK8, BACE1, PSMD9, CASP8, EIF2AK3, CASP9, BECN1, MAP3K5, PSMD1, IL1A, SNCA, FAS, CYBB, EIF2AK2, APAF1, XBP1, NFKB1, LRP1, TNFRSF1A, GAPDH, FADD, NDUFA2, MME, IKBKB, WNT3A, GRM5, ERN1, IKBKG |
| KEGG: 05146 | Amoebiasis | 2.20e-06 | 5,7 | 97 | 435 | 22 | 22,68 | IL6, TNF, CASP3, CXCL8, IL1B, RELA, TLR4, IL10, NOS2, FN1, TLR2, IFNG, CXCL1, PTK2, RAB5A, ITGAM, CSF2, NFKB1, ARG1, COL1A1, ARG2, CD14 |
| KEGG: 05224 | Breast cancer | 3.56e-06 | 5,4 | 143 | 435 | 27 | 18,88 | AKT1, MAPK3, TP53, EGFR, CDKN1A, JUN, ESR1, MAPK1, EGF, IGF1, FOS, ERBB2, HRAS, RAF1, MTOR, FGF2, KRAS, MAP2K1, PTEN, SP1, FGFR1, FGF7, HES1, HEY2, E2F1, JAG1, WNT3A |

| | | | | | | | | |
|---|---|---|---|---|---|---|---|---|
| KEGG: 05207 | Chemical carcinogenesis - receptor activation | 4.32e-06 | 5,4 | 202 | 435 | 33 | 16,34 | AKT1, MAPK3, EGFR, JUN, ESR1, MAPK1, STAT3, EGF, FOS, HRAS, RAF1, MTOR, FGF2, KRAS, SRC, MAP2K1, RELA, CREB1, PPARA, HSP90AA1, RPS6KA3, RPS6KA1, UGT1A1, UGT1A8, AIP, UGT1A6, RPS6KA2, FGF7, NFKB1, VDR, E2F1, JAG1, BCL6 |
| KEGG: 05230 | Central carbon metabolism in cancer | 4.37e-06 | 5,4 | 69 | 435 | 18 | 26,09 | AKT1, MAPK3, TP53, EGFR, MAPK1, ERBB2, HIF1A, HRAS, RAF1, MTOR, KRAS, MAP2K1, PTEN, MET, FGFR2, HK2, FGFR1, PKM |
| KEGG: 04520 | Adherens junction | 4.37e-06 | 5,4 | 69 | 435 | 18 | 26,09 | MAPK3, EGFR, MAPK1, ERBB2, SRC, CDH1, MET, SMAD3, TJP1, FGFR1, FYN, RHOA, CDC42, PTPN1, RAC1, MAP3K7, RAC2, CREBBP |
| KEGG: 04931 | Insulin resistance | 7.03e-06 | 5,2 | 103 | 435 | 22 | 21,36 | AKT1, IL6, TNF, STAT3, INS, MTOR, PTEN, RELA, CREB1, PPARA, MAPK8, RPS6KA3, RPS6KA1, FOXO1, RPS6KA2, SOCS3, NFKB1, TNFRSF1A, PTPN1, PTPN11, PDPK1, IKBKB |
| KEGG: 04370 | VEGF signaling pathway | 8.22e-06 | 5,1 | 57 | 435 | 16 | 28,07 | AKT1, MAPK3, PTGS2, MAPK1, MAPK14, HRAS, RAF1, KRAS, SRC, MAP2K1, CASP9, |

| | | | | | | | | |
|---|---|---|---|---|---|---|---|---|
| | | | | | | | | SPHK1, PTK2, CDC42, RAC1, RAC2 |
| KEGG: 05213 | Endometrial cancer | 8.22e-06 | 5,1 | 57 | 435 | 16 | 28,07 | AKT1, MAPK3, TP53, EGFR, CDKN1A, MAPK1, EGF, ERBB2, HRAS, RAF1, KRAS, MAP2K1, PTEN, CDH1, CASP9, PDPK1 |
| KEGG: 04115 | p53 signaling pathway | 8.95e-06 | 5 | 72 | 435 | 18 | 25 | TP53, CDKN1A, IGF1, CASP3, PTEN, TNFRSF10B, CYCS, SERPINE1, ATM, CASP8, MDM2, CASP9, CDK1, IGFBP3, TNFRSF10A, FAS, TSC2, APAF1 |
| KEGG: 05211 | Renal cell carcinoma | 1.01e-05 | 5 | 65 | 435 | 17 | 26,15 | AKT1, MAPK3, CDKN1A, JUN, MAPK1, CRK, HIF1A, HRAS, RAF1, KRAS, MAP2K1, MET, PAK1, CDC42, PTPN11, RAC1, CREBBP |
| KEGG: 05310 | Asthma | 1.31e-05 | 4,9 | 27 | 435 | 11 | 40,74 | TNF, IL10, CD40LG, IL4, RNASE3, CD40, IL13, CCL11, EPX, IL3, IL5 |
| KEGG: 04510 | Focal adhesion | 1.50e-05 | 4,8 | 192 | 435 | 31 | 16,15 | AKT1, MAPK3, EGFR, JUN, MAPK1, EGF, CRK, IGF1, ERBB2, HRAS, RAF1, SRC, MAP2K1, PTEN, CAV1, SPP1, MAPK8, FN1, MET, PTK2, PAK1, FYN, RHOA, CDC42, BIRC3, COL1A1, COL2A1, PDPK1, RAC1, VASP, RAC2 |

| | | | | | | | | |
|---|---|---|---|---|---|---|---|---|
| KEGG: 04015 | Rap1 signaling pathway | 1.65e-05 | 4,8 | 203 | 435 | 32 | 15,76 | AKT1, MAPK3, EGFR, MAPK1, MAPK14, EGF, CRK, IGF1, INS, HRAS, RAF1, FGF2, KRAS, SRC, MAP2K1, CDH1, MET, FGFR2, F2R, ID1, FGFR1, FGF7, RHOA, CDC42, ITGAM, RAPGEF3, MAP2K3, F2RL3, RAC1, MAP2K6, VASP, RAC2 |
| KEGG: 05143 | African trypanosomiasis | 3.24e-05 | 4,5 | 35 | 435 | 12 | 34,29 | IL6, TNF, IL1B, ICAM1, IL10, IL18, FASLG, VCAM1, IFNG, FAS, MYD88, IDO1 |
| KEGG: 04071 | Sphingolipid signaling pathway | 4.66e-05 | 4,3 | 114 | 435 | 22 | 19,3 | AKT1, MAPK3, TP53, TNF, MAPK1, MAPK14, HRAS, RAF1, KRAS, MAP2K1, PTEN, RELA, MAPK8, SPHK1, MAP3K5, FYN, RHOA, NFKB1, TNFRSF1A, PDPK1, RAC1, RAC2 |
| KEGG: 04014 | Ras signaling pathway | 5.93e-05 | 4,2 | 225 | 435 | 33 | 14,67 | AKT1, MAPK3, EGFR, MAPK1, EGF, IGF1, INS, BDNF, HRAS, RAF1, FGF2, KRAS, MAP2K1, RELA, MAPK8, ABL1, MET, FASLG, FGFR2, FGFR1, PAK1, FGF7, RAB5A, RHOA, CDC42, NFKB1, RALBP1, PTPN11, RAC1, STK4, IKBKB, RAC2, IKBKG |
| KEGG: 04672 | Intestinal immune network for IgA | 7.34e-05 | 4,1 | 44 | 435 | 13 | 29,55 | IL6, IL10, CXCR4, CD40LG, IL4, CXCL12, CD40, IL2, TNFSF13B, CD80, CD28, CD86, IL5 |

| | | | | | | | | |
|---|---|---|---|---|---|---|---|---|
| | production | | | | | | | |
| KEGG: 04919 | Thyroid hormone signaling pathway | 8.67e-05 | 4,1 | 118 | 435 | 22 | 18,64 | AKT1, MAPK3, TP53, ESR1, MAPK1, HIF1A, HRAS, RAF1, MTOR, KRAS, SRC, MAP2K1, MDM2, CASP9, STAT1, FOXO1, HDAC1, TSC2, PDPK1, MED12, CREBBP, HDAC2 |
| KEGG: 04670 | Leukocyte transendothelial migration | 1.03e-04 | 4 | 110 | 435 | 21 | 19,09 | MMP9, MAPK14, MMP2, ICAM1, CXCR4, CXCL12, VCAM1, PECAM1, PTK2, OCLN, CYBB, CLDN1, RHOA, CDC42, ITGAM, RAPGEF3, CLDN7, PTPN11, RAC1, VASP, RAC2 |
| KEGG: 05022 | Pathways of neurodegeneration - multiple diseases | 1.17e-04 | 3,9 | 442 | 435 | 51 | 11,54 | MAPK3, PTGS2, IL6, TNF, MAPK1, MAPK14, CASP3, BDNF, HRAS, MT-CO2, RAF1, MTOR, KRAS, MAP2K1, IL1B, RELA, APP, NOS2, CYCS, MAPK8, SOD1, DCTN6, PSMD9, CASP8, FASLG, EIF2AK3, CASP9, BECN1, PRNP, SQSTM1, MAP3K5, PSMD1, IL1A, SNCA, FAS, CYBB, DCTN4, RAB5A, UBE2L3, APAF1, XBP1, NFKB1, TNFRSF1A, FADD, MAP2K3, NDUFA2, |

| | | | | | | | | |
|---|---|---|---|---|---|---|---|---|
| | | | | | | | | RAC1, MAP2K6, WNT3A, GRM5, ERN1 |
| KEGG: 05231 | Choline metabolism in cancer | 1.39e-04 | 3,9 | 94 | 435 | 19 | 20,21 | AKT1, MAPK3, EGFR, JUN, MAPK1, EGF, FOS, HIF1A, HRAS, RAF1, MTOR, KRAS, MAP2K1, MAPK8, SP1, TSC2, PDPK1, RAC1, RAC2 |
| KEGG: 04211 | Longevity regulating pathway | 1.56e-04 | 3,8 | 86 | 435 | 18 | 20,93 | AKT1, TP53, IGF1, INS, HRAS, SIRT1, MTOR, KRAS, RELA, PPARG, CREB1, FOXO1, TSC2, NFKB1, ATG5, EIF4E, RPTOR, EHMT1 |
| KEGG: 04150 | mTOR signaling pathway | 1.57e-04 | 3,8 | 151 | 435 | 25 | 16,56 | AKT1, MAPK3, TNF, MAPK1, IGF1, INS, HRAS, RAF1, MTOR, KRAS, MAP2K1, PTEN, RPS6KA3, RPS6KA1, RPS6KA2, TSC2, RHOA, TNFRSF1A, EIF4E, RICTOR, RPTOR, PDPK1, IKBKB, WNT3A, MAPKAP1 |
| KEGG: 04926 | Relaxin signaling pathway | 1.81e-04 | 3,7 | 123 | 435 | 22 | 17,89 | AKT1, MAPK3, MMP9, EGFR, JUN, MAPK1, MAPK14, FOS, MMP2, HRAS, RAF1, KRAS, SRC, MAP2K1, RELA, CREB1, NOS2, MAPK8, MMP13, NFKB1, MAP2K4, COL1A1 |

| | | | | | | | | |
|---|---|---|---|---|---|---|---|---|
| KEGG: 05226 | Gastric cancer | 2.00e-04 | 3,7 | 143 | 435 | 24 | 16,78 | AKT1, MAPK3, TP53, EGFR, CDKN1A, MAPK1, EGF, ERBB2, HRAS, RAF1, MTOR, FGF2, KRAS, MAP2K1, CDH1, ABCB1, MET, SMAD3, FGFR2, FGF7, TERT, E2F1, WNT3A, RARB |
| KEGG: 05214 | Glioma | 2.15e-04 | 3,7 | 71 | 435 | 16 | 22,54 | AKT1, MAPK3, TP53, EGFR, CDKN1A, MAPK1, EGF, IGF1, HRAS, RAF1, MTOR, KRAS, MAP2K1, PTEN, MDM2, E2F1 |
| KEGG: 04915 | Estrogen signaling pathway | 2.40e-04 | 3,6 | 125 | 435 | 22 | 17,6 | AKT1, MAPK3, MMP9, EGFR, JUN, ESR1, MAPK1, FOS, MMP2, HRAS, RAF1, KRAS, SRC, MAP2K1, CREB1, SP1, HSP90AA1, HSPA1A, OPRM1, FKBP4, RARA, FKBP5 |
| KEGG: 04213 | Longevity regulating pathway - multiple species | 4.00e-04 | 3,4 | 58 | 435 | 14 | 24,14 | AKT1, IGF1, INS, HRAS, SIRT1, MTOR, KRAS, SOD1, HSPA1A, FOXO1, HDAC1, ATG5, RPTOR, HDAC2 |
| KEGG: 04072 | Phospholipase D signaling pathway | 4.22e-04 | 3,4 | 139 | 435 | 23 | 16,55 | AKT1, MAPK3, EGFR, MAPK1, EGF, INS, HRAS, CXCL8, RAF1, MTOR, KRAS, MAP2K1, F2R, AGTR1, SPHK1, TSC2, FYN, RHOA, RAPGEF3, CXCR2, PTPN11, CXCR1, GRM5 |

| | | | | | | | | |
|---|---|---|---|---|---|---|---|---|
| KEGG: 04920 | Adipocytokine signaling pathway | 7.27e-04 | 3,1 | 69 | 435 | 15 | 21,74 | AKT1, TNF, STAT3, LEP, MTOR, RELA, PPARA, MAPK8, SOCS3, NFKB1, TNFRSF1A, NPY, PTPN11, IKBKB, IKBKG |
| KEGG: 04810 | Regulation of actin cytoskeleton | 7.52e-04 | 3,1 | 207 | 435 | 29 | 14,01 | MAPK3, EGFR, MAPK1, EGF, CRK, INS, HRAS, RAF1, FGF2, KRAS, SRC, MAP2K1, CXCR4, CXCL12, FN1, FGFR2, F2R, FGFR1, PTK2, PAK1, FGF7, ARHGEF7, RHOA, CDC42, ITGAM, RAC1, CFL1, RAC2, ITGAX |
| KEGG: 04215 | Apoptosis - multiple species | 8.47e-04 | 3,1 | 32 | 435 | 10 | 31,25 | CASP3, CYCS, MAPK8, CASP8, CASP9, BECN1, APAF1, TNFRSF1A, BIRC3, FADD |
| KEGG: 04912 | GnRH signaling pathway | 1.79e-03 | 2,7 | 92 | 435 | 17 | 18,48 | MAPK3, EGFR, JUN, MAPK1, MAPK14, MMP2, HRAS, RAF1, KRAS, SRC, MAP2K1, MAPK8, MAP3K1, CDC42, MAP2K4, MAP2K3, MAP2K6 |
| KEGG: 04610 | Complement and coagulation cascades | 1.83e-03 | 2,7 | 83 | 435 | 16 | 19,28 | SERPINE1, F2R, PROC, F3, ITGAM, C5AR1, C3, C5, F2RL3, THBD, SERPINA1, PLG, CD46, CD55, CD59, ITGAX |
| KEGG: 04910 | Insulin signaling pathway | 1.85e-03 | 2,7 | 131 | 435 | 21 | 16,03 | AKT1, MAPK3, MAPK1, CRK, INS, HRAS, RAF1, MTOR, KRAS, MAP2K1, MAPK8, FOXO1, HK2, TSC2, PKLR, SOCS3, EIF4E, RPTOR, PTPN1, |

| | | | | | | | | |
|---|---|---|---|---|---|---|---|---|
| | | | | | | | | PDPK1, IKBKB |
| KEGG: 04666 | Fc gamma R-mediated phagocytosis | 2.41e-03 | 2,6 | 94 | 435 | 17 | 18,09 | AKT1, MAPK3, MAPK1, CRK, RAF1, MAP2K1, SPHK1, PAK1, CDC42, PTPRC, FCGR2A, RAC1, FCGR1A, AMPH, CFL1, VASP, RAC2 |
| KEGG: 04662 | B cell receptor signaling pathway | 2.51e-03 | 2,6 | 76 | 435 | 15 | 19,74 | AKT1, MAPK3, JUN, MAPK1, FOS, HRAS, RAF1, KRAS, MAP2K1, RELA, NFKB1, RAC1, IKBKB, RAC2, IKBKG |
| KEGG: 05415 | Diabetic cardiomyopathy | 2.60e-03 | 2,6 | 187 | 435 | 26 | 13,9 | AKT1, MMP9, MAPK14, INS, MMP2, MT-CO2, MTOR, PTEN, RELA, PARP1, PPARA, MAPK8, SP1, SMAD3, ACE, AGTR1, CYBB, NFKB1, REN, GAPDH, COL1A1, NDUFA2, TNNI3, RAC1, CPT2, RAC2 |
| KEGG: 04914 | Progesterone-mediated oocyte maturation | 3.68e-03 | 2,4 | 97 | 435 | 17 | 17,53 | AKT1, MAPK3, MAPK1, MAPK14, IGF1, INS, RAF1, KRAS, MAP2K1, MAPK8, HSP90AA1, RPS6KA3, RPS6KA1, PLK1, CDK1, RPS6KA2, CCNA1 |
| KEGG: 04930 | Type II diabetes mellitus | 3.97e-03 | 2,4 | 45 | 435 | 11 | 24,44 | MAPK3, TNF, MAPK1, INS, MTOR, MAPK8, HK2, PKLR, SOCS3, PKM, IKBKB |
| KEGG: 04137 | Mitophagy - animal | 4.03e-03 | 2,4 | 70 | 435 | 14 | 20 | TP53, JUN, HIF1A, HRAS, KRAS, SRC, RELA, MAPK8, SP1, |

| | | | | | | | | |
|---|---|---|---|---|---|---|---|---|
| | | | | | | | | EIF2AK3, BECN1, SQSTM1, E2F1, ATG5 |
| KEGG: 04514 | Cell adhesion molecules | 7.12e-03 | 2,1 | 143 | 435 | 21 | 14,69 | CDH1, ICAM1, CD40LG, CD40, VCAM1, CD274, CD4, PECAM1, OCLN, CD8A, CLDN1, SDC1, ITGAM, PDCD1, PTPRC, CD80, CLDN7, HLA-A, CD28, CADM1, CD86 |
| KEGG: 05168 | Herpes simplex virus 1 infection | 9.76e-03 | 2 | 477 | 435 | 48 | 10,06 | AKT1, TP53, IL6, TNF, CASP3, MTOR, SRC, CCL2, IL1B, RELA, CYCS, CASP8, FASLG, EIF2AK3, TLR2, CASP9, STAT1, IFNG, JAK1, IFNA1, FAS, TSC2, EIF2AK2, SOCS3, IRAK1, MYD88, APAF1, CCL5, NFKB1, TNFRSF1A, TRAF3, C3, TRAF6, C5, BIRC3, FADD, HLA-A, CALR, PTPN11, MAP3K7, STAT2, LTA, IKBKB, SRSF1, TAP1, IFNB1, IKBKG |
| KEGG: 04550 | Signaling pathways regulating pluripotency of stem cells | 1.88e-02 | 1,7 | 131 | 435 | 19 | 14,5 | AKT1, MAPK3, MAPK1, MAPK14, STAT3, IGF1, HRAS, RAF1, FGF2, KRAS, MAP2K1, SMAD3, FGFR2, JAK1, ID1, FGFR1, JAK3, IL6ST, WNT3A |
| KEGG: 05216 | Thyroid cancer | 2.15e-02 | 1,7 | 37 | 435 | 9 | 24,32 | MAPK3, TP53, CDKN1A, MAPK1, HRAS, KRAS, MAP2K1, CDH1, PPARG |

| | | | | | | | | |
|---|---|---|---|---|---|---|---|---|
| KEGG: 04720 | Long-term potentiatio n | 3.2 2e-02 | 1,5 | 65 | 435 | 12 | 18,46 | MAPK3, MAPK1, HRAS, RAF1, KRAS, MAP2K1, RPS6KA3, RPS6KA1, RPS6KA2, RAPGEF3, GRM5, CREBBP |
| KEGG: 05020 | Prion disease | 3.6 5e-02 | 1,4 | 24 3 | 435 | 28 | 11,52 | MAPK3, IL6, TNF, MAPK1, MAPK14, CASP3, MT-CO2, IL1B, CAV1, CREB1, CYCS, MAPK8, SOD1, PSMD9, EIF2AK3, CASP9, HSPA1A, PRNP, PSMD1, IL1A, CYBB, FYN, APAF1, CCL5, C5, NDUFA2, RAC1, RAC2 |
| KEGG: 04612 | Antigen processing and presentatio n | 4.3 2e-02 | 1,4 | 67 | 435 | 12 | 17,91 | TNF, CREB1, HSP90AA1, HSPA1A, IFNG, CD4, CD8A, CTSB, KIR3DL2, HLA-A, CALR, TAP1 |
| KEGG: 04360 | Axon guidance | 4.4 5e-02 | 1,4 | 17 4 | 435 | 22 | 12,64 | MAPK3, MAPK1, HRAS, RAF1, KRAS, SRC, CXCR4, CXCL12, ABL1, MET, PTK2, PAK1, FYN, RHOA, NRP1, CDC42, PTPN11, PDPK1, RAC1, RHOD, CFL1, RAC2 |

| **TABLE S8: Pathways enriched (Reactome)** | | | | | | | | |
|---|---|---|---|---|---|---|---|---|
| **Term_ID** | **Function** | **P-value** | **-log10 Pvalue** | **Term Size** | **Query size** | **Intersection Size** | **Enrichment Score %** | **Positive Hits** |
| REAC:R-HSA-1280215 | Cytokine Signaling in Immune system | 8.85e-61 | 60,1 | 705 | 491 | 150 | 21,28 | AKT1, MAPK3, TP53, PTGS2, IL6, MMP9, TNF, CDKN1A, JUN, MAPK1, MAPK14, STAT3, CRK, GRAP2, FOS, MMP2, CASP3, HIF1A, HRAS, JUNB, CXCL8, HMOX1, FGF2, KRAS, CCL2, MAP2K1, IL1B, RELA, CD44, ICAM1, APP, CREB1, IL10, NOS2, CD40LG, IL4, HMGB1, MAPK8, SOD1, IL18, TIMP1, FN1, PSMD9, IFI27, FASLG, SMAD3, HSP90AA1, RPS6KA3, VCAM1, STAT1, RPS6KA1, IL13, FOXO1, CCL11, CASP1, OSM, IFNG, CD4, SQSTM1, CXCL10, JAK1, EIF4G1, IL2, CCR5, CXCL1, STAT6, PSMD1, IL1A, AIP, TNFSF13B, IFNA1, IL24, RPS6KA2, NUP62, EIF2AK2, IL17C, MX2, FYN, CSF3, RPS6KA5, SOCS3, IRAK1, JAK3, MYD88, CCL5, IL1RL1, SDC1, IL6ST, IL33, CDC42, OPRM1, ITGAM, |

| | | | | | | | | |
|---|---|---|---|---|---|---|---|---|
| | | | | | | | | CSF2, P4HB, NFKB1, IL7, TNFRSF1A, CCL22, IRF1, TNFRSF25, MAP2K4, TRAF3, IL3, IL17RA, SUMO1, EIF4E, TRAF6, IL22, MUC1, BIRC3, CD80, TSLP, MAP2K3, PTPN1, PIK3R2, HLA-A, CCL4, PTPN11, FCGR1A, MAP3K7, TNFSF12, ISG20, STAT2, ANXA2, IL2RA, BCL6, LTA, IFNL3, IKBKB, IL32, CFL1, MAP2K6, CCL3, MIF, TNFSF8, DUSP4, TNFSF9, CD70, CD86, CCL20, GBP1, IL5, IL1RN, ANXA1, IL1RAPL1, IFNB1, MX1, ITGAX, IKBKG |
| REAC:R-HSA-449147 | Signaling by Interleukins | 1.46e-56 | 55,8 | 460 | 491 | 119 | 25,87 | AKT1, MAPK3, TP53, PTGS2, IL6, MMP9, TNF, CDKN1A, JUN, MAPK1, MAPK14, STAT3, CRK, FOS, MMP2, CASP3, HIF1A, JUNB, CXCL8, HMOX1, FGF2, CCL2, MAP2K1, IL1B, RELA, ICAM1, APP, CREB1, IL10, NOS2, IL4, HMGB1, MAPK8, SOD1, IL18, TIMP1, FN1, PSMD9, FASLG, SMAD3, HSP90AA1, RPS6KA3, VCAM1, STAT1, RPS6KA1, IL13, FOXO1, CCL11, CASP1, OSM, IFNG, CD4, SQSTM1, |

| | | | | | | | | |
|---|---|---|---|---|---|---|---|---|
| | | | | | | | | CXCL10, JAK1, IL2, CCR5, CXCL1, STAT6, PSMD1, IL1A, AIP, IL24, RPS6KA2, IL17C, FYN, CSF3, RPS6KA5, SOCS3, IRAK1, JAK3, MYD88, CCL5, IL1RL1, SDC1, IL6ST, IL33, CDC42, OPRM1, ITGAM, CSF2, P4HB, NFKB1, IL7, TNFRSF1A, CCL22, MAP2K4, IL3, IL17RA, TRAF6, IL22, MUC1, CD80, TSLP, MAP2K3, PIK3R2, CCL4, PTPN11, MAP3K7, STAT2, ANXA2, IL2RA, BCL6, IFNL3, IKBKB, IL32, CFL1, MAP2K6, CCL3, MIF, DUSP4, CD86, CCL20, IL5, IL1RN, ANXA1, IL1RAPL1, ITGAX, IKBKG |

| | | | | | | | | |
|---|---|---|---|---|---|---|---|---|
| REAC:R-HSA-168256 | Immune System | 3.17e-56 | 55,5 | 2041 | 491 | 248 | 12,15 | AKT1, MAPK3, TP53, PTGS2, IL6, MMP9, TNF, CDKN1A, JUN, MAPK1, MAPK14, STAT3, CRK, GRAP2, RNF19A, FOS, MMP2, CASP3, HIF1A, HRAS, JUNB, CXCL8, HMOX1, RAF1, MTOR, FGF2, KRAS, CCL2, MAP2K1, PTEN, IL1B, CDH1, RELA, CD44, ICAM1, APP, CREB1, TLR4, IL10, NOS2, CD40LG, IL4, CRP, MPO, HMGB1, MAPK8, ABL1, SOD1, IL18, NLRP3, TIMP1, FN1, HP, DCTN6, PSMD9, IFI27, RNASE3, CASP8, FASLG, SMAD3, HSP90AA1, RPS6KA3, TLR2, CASP9, VCAM1, STAT1, RPS6KA1, IL13, HSPA1A, FOXO1, CCL11, CASP1, TTR, CD274, OSM, IFNG, CD4, SQSTM1, MUC5AC, CXCL10, JAK1, EIF4G1, KEAP1, IL2, CCR5, AIM2, CXCL1, MUC5B, STAT6, PSMD1, KIF2A, PECAM1, IL1A, AIP, TNFSF13B, PTK2, RIPK3, IFNA1, PAK1, FPR2, CYBB, IL24, MAP3K1, DCTN4, RPS6KA2, NUP62, EIF2AK2, IL17C, MX2, FYN, CD8A, MUC15, CSF3, ATG7, |

| | | | | | | | | |
|---|---|---|---|---|---|---|---|---|
| | | | | | | | | RPS6KA5, SOCS3, IRAK1, CAP1, JAK3, RHOA, UBE2L3, MYD88, APAF1, CCL5, GRN, IL1RL1, SDC1, IL6ST, IL33, CDC42, OPRM1, ITGAM, CSF2, DEFB1, PKM, P4HB, CTSB, NFKB1, IL7, TNFRSF1A, CCL22, IRF1, TNFRSF25, MAP2K4, TLR5, TRAF3, C5AR1, ARG1, ATG5, TLR7, EPX, C3, RIPK1, IL3, IL17RA, PDCD1, SUMO1, RAPGEF3, PTPRC, EIF4E, TRAF6, IL22, MUC1, C5, BIRC3, CD80, RICTOR, FADD, RBBP6, CXCR2, KIR3DL2, TSLP, MAP2K3, PTPN1, PIK3R2, COL1A1, FCGR2A, COL2A1, HLA-A, CCL4, CD14, CALR, PTPN11, PDPK1, RAC1, FCGR1A, MAP3K7, TNFSF12, CXCR1, ISG20, STAT2, LAMP1, ATP7A, ANXA2, CTSE, PTPN22, TXN, IL2RA, BCL6, NLRC4, CYLD, LTA, IFNL3, SERPINA1, MME, IKBKB, IL32, CFL1, MRC1, MAP2K6, CCL3, MIF, TNFSF8, DUSP4, TNFSF9, CD70, PYCARD, CD33, PPBP, CD46, CD28, CD86, |

| | | | | | | | | |
|---|---|---|---|---|---|---|---|---|
| | | | | | | | | TAP1, KLRG1, CCL20, CD55, GBP1, SFTPA2, VTN, IL5, AZU1, VASP, RAC2, LGALS3, IL1RN, CREBBP, MAPKAP1, ANXA1, IL1RAPL1, IFNB1, TIRAP, CD59, MX1, SFTPA1, ITGAX, ELANE, IKBKG |
| REAC:R-HSA-6785807 | Interleukin-4 and Interleukin-13 signaling | 5.40e-28 | 27,3 | 110 | 491 | 44 | 40 | AKT1, TP53, PTGS2, IL6, MMP9, TNF, CDKN1A, STAT3, FOS, MMP2, HIF1A, JUNB, CXCL8, HMOX1, FGF2, CCL2, IL1B, ICAM1, IL10, NOS2, IL4, IL18, TIMP1, FN1, FASLG, HSP90AA1, VCAM1, STAT1, IL13, FOXO1, CCL11, OSM, JAK1, STAT6, IL1A, SOCS3, JAK3, OPRM1, ITGAM, CCL22, MUC1, BCL6, ANXA1, ITGAX |

| | | | | | | | | |
|---|---|---|---|---|---|---|---|---|
| REAC:R-HSA-162582 | Signal Transduction | 2.78e-26 | 25,6 | 2523 | 491 | 225 | 8,92 | AKT1, MAPK3, TP53, IL6, MMP9, EGFR, TNF, CDKN1A, JUN, ESR1, MAPK1, MAPK14, STAT3, EGF, CRK, GRAP2, IGF1, FOS, INS, MMP2, CASP3, BDNF, JUND, ERBB2, HIF1A, HRAS, LEP, JUNB, CXCL8, FOSB, CFTR, RAF1, MTOR, FGF2, KRAS, SRC, APOE, CCL2, MAP2K1, PTEN, CDH1, RELA, APP, PPARG, TNFRSF10B, CAV1, CREB1, CXCR4, PARP1, SPP1, AREG, CXCL12, MAPK8, ABL1, YBX1, SP1, SERPINE1, FN1, ABCA1, PSMD9, MET, CASP8, FASLG, GAST, SMAD3, COL11A2, HSP90AA1, RPS6KA3, MDM2, CASP9, TNFSF10, CCR7, STAT1, RPS6KA1, GLI1, ALK, FOXO1, CCL11, CD274, EZH2, GRP, SQSTM1, CXCL10, PLK1, CFLAR, F2R, JAK1, EIF4G1, IL2, CCR5, TJP1, ID1, CDK1, CXCL1, AGTR1, STAT6, SPHK1, KLK3, PSMD1, KLK2, TAC1, HDAC1, KIF2A, PTK2, TNFRSF10A, PAK1, FPR2, FAS, CYBB, RPS6KA2, TSC2, FGF7, |

| | | | | | | | | |
|---|---|---|---|---|---|---|---|---|
| | | | | | | | | KHDRBS1, FYN, HES1, KDM1A, RPS6KA5, TERT, SOCS3, HEY2, IRAK1, F3, TRAF1, ARHGEF7, JAK3, RHOA, MYD88, CCL5, IL1RL1, NRP1, IL33, CDC42, NRG1, OPRM1, CSF2, P4HB, ALDH1A3, FKBP4, NFKB1, TNFRSF1A, CCL22, E2F1, C5AR1, HTR2A, NR3C1, C3, RIPK1, RHO, IL3, GRK2, CDK9, CXCR3, MDK, RAPGEF3, EIF4E, TRAF6, NR4A1, PHB, RALBP1, C5, BIRC3, CD80, RICTOR, FADD, RPTOR, RBBP6, CXCR2, PTPN1, GZMB, PIK3R2, C1QBP, COL1A1, F2RL3, JAG1, CTTN, COL2A1, NPY, XPO1, CCL4, ALDH1A1, PTPN11, PDPK1, RAC1, MAP3K7, CXCR1, RHOD, XCR1, CXCL9, STK4, IL2RA, STAG1, CYLD, IKBKB, IL32, CFL1, HNRNPC, CCL3, CCL4L2, DUSP4, GAL, WNT3A, PPBP, GRM5, PLG, CD28, CD86, RARB, CCL20, CD55, XCL2, CCL17, IL5, ID2, RAC2, RARA, CREBBP, MAPKAP1, IGF2BP1, ANXA1, GPS1, HDAC2, |

| | | | | | | | | |
|---|---|---|---|---|---|---|---|---|
| | | | | | | | | CCR3, FKBP5, SPRY1, IKBKG |
| REAC:R-HSA-6783783 | Interleukin-10 signaling | 1.29e-22 | 21,9 | 45 | 491 | 27 | 60 | PTGS2, IL6, TNF, STAT3, CXCL8, CCL2, IL1B, ICAM1, IL10, IL18, TIMP1, CXCL10, JAK1, CCR5, CXCL1, IL1A, CSF3, CCL5, CSF2, TNFRSF1A, CCL22, CD80, CCL4, CCL3, CD86, CCL20, IL1RN |

| | | | | | | | | |
|---|---|---|---|---|---|---|---|---|
| REAC:R-HSA-168249 | Innate Immune System | 2.50e-22 | 21,6 | 1094 | 491 | 128 | 11,7 | MAPK3, MMP9, JUN, MAPK1, MAPK14, CRK, GRAP2, FOS, HRAS, HMOX1, RAF1, MAP2K1, IL1B, RELA, CD44, APP, CREB1, TLR4, NOS2, CRP, MPO, HMGB1, MAPK8, ABL1, NLRP3, HP, PSMD9, RNASE3, CASP8, HSP90AA1, RPS6KA3, TLR2, CASP9, RPS6KA1, HSPA1A, CASP1, TTR, CD4, MUC5AC, AIM2, CXCL1, MUC5B, STAT6, PSMD1, PECAM1, PTK2, RIPK3, IFNA1, PAK1, FPR2, CYBB, MAP3K1, RPS6KA2, FYN, MUC15, ATG7, RPS6KA5, IRAK1, CAP1, RHOA, MYD88, APAF1, GRN, CDC42, ITGAM, DEFB1, PKM, CTSB, NFKB1, MAP2K4, TLR5, TRAF3, C5AR1, ARG1, ATG5, TLR7, EPX, C3, RIPK1, PTPRC, TRAF6, MUC1, C5, BIRC3, FADD, CXCR2, MAP2K3, PIK3R2, FCGR2A, CD14, PTPN11, PDPK1, RAC1, FCGR1A, MAP3K7, CXCR1, LAMP1, ATP7A, ANXA2, TXN, NLRC4, CYLD, SERPINA1, MME, IKBKB, CFL1, MAP2K6, MIF, DUSP4, PYCARD, |

| | | | | | | | | |
|---|---|---|---|---|---|---|---|---|
| | | | | | | | | CD33, PPBP, CD46, CD55, SFTPA2, VTN, AZU1, RAC2, LGALS3, CREBBP, IFNB1, TIRAP, CD59, SFTPA1, ITGAX, ELANE, IKBKG |
| REAC:R-HSA-168898 | Toll-like Receptor Cascades | 5.71e-21 | 20,2 | 154 | 491 | 44 | 28,57 | MAPK3, JUN, MAPK1, MAPK14, FOS, MAP2K1, RELA, APP, CREB1, TLR4, HMGB1, MAPK8, CASP8, RPS6KA3, TLR2, RPS6KA1, RIPK3, MAP3K1, RPS6KA2, RPS6KA5, IRAK1, MYD88, ITGAM, CTSB, NFKB1, MAP2K4, TLR5, TRAF3, TLR7, RIPK1, TRAF6, BIRC3, FADD, MAP2K3, CD14, PTPN11, MAP3K7, IKBKB, MAP2K6, DUSP4, SFTPA2, TIRAP, SFTPA1, IKBKG |
| REAC:R-HSA-166016 | Toll Like Receptor 4 (TLR4) Cascade | 5.72e-21 | 20,2 | 132 | 491 | 41 | 31,06 | MAPK3, JUN, MAPK1, MAPK14, FOS, MAP2K1, RELA, APP, CREB1, TLR4, HMGB1, MAPK8, CASP8, RPS6KA3, TLR2, RPS6KA1, RIPK3, |

| | | | | | | | | |
|---|---|---|---|---|---|---|---|---|
| | | | | | | | | MAP3K1, RPS6KA2, RPS6KA5, IRAK1, MYD88, ITGAM, NFKB1, MAP2K4, TRAF3, RIPK1, TRAF6, BIRC3, FADD, MAP2K3, CD14, PTPN11, MAP3K7, IKBKB, MAP2K6, DUSP4, SFTPA2, TIRAP, SFTPA1, IKBKG |
| REAC:R-HSA-166166 | MyD88-independent TLR4 cascade | 3.36e-19 | 18,5 | 96 | 491 | 34 | 35,42 | MAPK3, JUN, MAPK1, MAPK14, FOS, MAP2K1, RELA, APP, CREB1, TLR4, HMGB1, MAPK8, CASP8, RPS6KA3, RPS6KA1, RIPK3, RPS6KA2, RPS6KA5, IRAK1, NFKB1, MAP2K4, TRAF3, RIPK1, TRAF6, BIRC3, FADD, MAP2K3, CD14, PTPN11, MAP3K7, IKBKB, MAP2K6, DUSP4, IKBKG |
| REAC:R-HSA-937061 | TRIF(TICAM1)-mediated TLR4 signaling | 3.36e-19 | 18,5 | 96 | 491 | 34 | 35,42 | MAPK3, JUN, MAPK1, MAPK14, FOS, MAP2K1, RELA, APP, CREB1, TLR4, HMGB1, MAPK8, CASP8, RPS6KA3, RPS6KA1, RIPK3, RPS6KA2, RPS6KA5, IRAK1, NFKB1, MAP2K4, TRAF3, RIPK1, TRAF6, BIRC3, FADD, MAP2K3, CD14, PTPN11, MAP3K7, IKBKB, MAP2K6, DUSP4, IKBKG |

| | | | | | | | | |
|---|---|---|---|---|---|---|---|---|
| REAC:R-HSA-181438 | Toll Like Receptor 2 (TLR2) Cascade | 4.99e-17 | 16,3 | 103 | 491 | 33 | 32,04 | MAPK3, JUN, MAPK1, MAPK14, FOS, MAP2K1, RELA, APP, CREB1, TLR4, HMGB1, MAPK8, RPS6KA3, TLR2, RPS6KA1, MAP3K1, RPS6KA2, RPS6KA5, IRAK1, MYD88, NFKB1, MAP2K4, TRAF6, MAP2K3, CD14, MAP3K7, IKBKB, MAP2K6, DUSP4, SFTPA2, TIRAP, SFTPA1, IKBKG |
| REAC:R-HSA-168179 | Toll Like Receptor TLR1:TLR2 Cascade | 4.99e-17 | 16,3 | 103 | 491 | 33 | 32,04 | MAPK3, JUN, MAPK1, MAPK14, FOS, MAP2K1, RELA, APP, CREB1, TLR4, HMGB1, MAPK8, RPS6KA3, TLR2, RPS6KA1, MAP3K1, RPS6KA2, RPS6KA5, IRAK1, MYD88, NFKB1, MAP2K4, TRAF6, MAP2K3, CD14, MAP3K7, IKBKB, MAP2K6, DUSP4, SFTPA2, TIRAP, SFTPA1, IKBKG |
| REAC:R-HSA-2219528 | PI3K/AKT Signaling in Cancer | 1.24e-16 | 15,9 | 92 | 491 | 31 | 33,7 | AKT1, EGFR, CDKN1A, ESR1, EGF, ERBB2, MTOR, FGF2, SRC, PTEN, CREB1, AREG, MET, MDM2, CASP9, FOXO1, TSC2, FGF7, FYN, NRG1, NR4A1, CD80, RICTOR, PIK3R2, PTPN11, PDPK1, RAC1, CD28, CD86, RAC2, |

| | | | | | | | | |
|---|---|---|---|---|---|---|---|---|
| | | | | | | | | MAPKAP1 |
| REAC:R-HSA-168164 | Toll Like Receptor 3 (TLR3) Cascade | 1.24e-16 | 15,9 | 92 | 491 | 31 | 33,7 | MAPK3, JUN, MAPK1, MAPK14, FOS, MAP2K1, RELA, APP, CREB1, HMGB1, MAPK8, CASP8, RPS6KA3, RPS6KA1, RIPK3, RPS6KA2, RPS6KA5, IRAK1, NFKB1, MAP2K4, TRAF3, RIPK1, TRAF6, BIRC3, FADD, MAP2K3, MAP3K7, IKBKB, MAP2K6, DUSP4, IKBKG |
| REAC:R-HSA-9006934 | Signaling by Receptor Tyrosine Kinases | 1.33e-16 | 15,9 | 502 | 491 | 73 | 14,54 | AKT1, MAPK3, MMP9, EGFR, ESR1, MAPK1, MAPK14, STAT3, EGF, CRK, GRAP2, IGF1, FOS, INS, BDNF, JUND, ERBB2, HIF1A, HRAS, JUNB, FOSB, MTOR, FGF2, SRC, APOE, MAP2K1, CAV1, CREB1, SPP1, AREG, CXCL12, FN1, MET, COL11A2, HSP90AA1, RPS6KA3, STAT1, RPS6KA1, ALK, CD274, ID1, STAT6, SPHK1, HDAC1, PTK2, PAK1, CYBB, RPS6KA2, FGF7, FYN, RPS6KA5, F3, ARHGEF7, JAK3, RHOA, NRP1, CDC42, NRG1, MDK, RICTOR, PTPN1, PIK3R2, COL1A1, COL2A1, |

| | | | | | | | | |
|---|---|---|---|---|---|---|---|---|
| | | | | | | | | PTPN11, PDPK1, RAC1, DUSP4, PLG, ID2, MAPKAP1, HDAC2, SPRY1 |
| REAC:R-HSA-975138 | TRAF6 mediated induction of NFkB and MAP kinases upon TLR7/8 or 9 activation | 4.74e-16 | 15,3 | 89 | 491 | 30 | 33,71 | MAPK3, JUN, MAPK1, MAPK14, FOS, MAP2K1, RELA, APP, CREB1, TLR4, HMGB1, MAPK8, RPS6KA3, RPS6KA1, MAP3K1, RPS6KA2, RPS6KA5, IRAK1, MYD88, NFKB1, MAP2K4, TLR7, TRAF6, MAP2K3, CD14, MAP3K7, IKBKB, MAP2K6, DUSP4, IKBKG |
| REAC:R-HSA-975155 | MyD88 dependent cascade initiated on endosome | 6.81e-16 | 15,2 | 90 | 491 | 30 | 33,33 | MAPK3, JUN, MAPK1, MAPK14, FOS, MAP2K1, RELA, APP, CREB1, TLR4, HMGB1, MAPK8, RPS6KA3, RPS6KA1, MAP3K1, RPS6KA2, RPS6KA5, IRAK1, MYD88, NFKB1, MAP2K4, TLR7, TRAF6, MAP2K3, CD14, MAP3K7, IKBKB, MAP2K6, DUSP4, IKBKG |

| | | | | | | | | |
|---|---|---|---|---|---|---|---|---|
| REAC:R-HSA-1643685 | Disease | 7.53e-16 | 15,1 | 1694 | 491 | 153 | 9,03 | AKT1, MAPK3, TP53, IL6, EGFR, CDKN1A, JUN, ESR1, MAPK1, MAPK14, STAT3, EGF, CRK, ERBB2, HRAS, JUNB, HMOX1, CFTR, RAF1, MTOR, FGF2, KRAS, SRC, MAP2K1, PTEN, IL1B, CDH1, RELA, APP, SYT1, CREB1, TLR4, IL10, CXCR4, NOS2, PARP1, AREG, HMGB1, MAPK8, ABL1, IL18, NLRP3, FN1, ABCA1, PSMD9, MET, ATM, CASP8, FASLG, EIF2AK3, SMAD3, NPM1, HSP90AA1, MDM2, TLR2, CASP9, DNMT1, STAT1, BECN1, HSPA1A, ALK, FOXO1, CASP1, TTR, EZH2, CD4, SQSTM1, FGFR2, MUC5AC, JAK1, CCR5, UGT1A1, FGFR1, MUC5B, PSMD1, HDAC1, IL1A, CD9, BGN, PTK2, NUP62, TSC2, EIF2AK2, GTF2H1, FGF7, FYN, MUC15, HES1, RAB5A, ATG7, KDM1A, CEBPB, BSG, HEY2, HNRNPK, JAK3, MYD88, SDC1, CDC42, NRG1, ABCA3, FKBP4, NFKB1, MAP2K4, TLR5, TRAF3, DNMT3B, E2F1, TLR7, NR3C1, C3, RIPK1, |

| | | | | | | | | |
|---|---|---|---|---|---|---|---|---|
| | | | | | | | | PDCD1, CDK9, SUMO1, NR4A1, PHB, MUC1, CD80, RICTOR, FADD, PIK3R2, C1QBP, JAG1, DNMT3A, FCGR2A, XPO1, CD14, CALR, PTPN11, PDPK1, RAC1, FCGR1A, STAT2, TXN, MPRIP, IKBKB, MRC1, PYCARD, WNT3A, CD28, CD86, CD163, SFTPA2, RAC2, CREBBP, MAPKAP1, TIRAP, SFTPB, SFTPA1, HDAC2, DPEP2, IKBKG |
| REAC:R-HSA-168181 | Toll Like Receptor 7/8 (TLR7/8) Cascade | 9.74e-16 | 15 | 91 | 491 | 30 | 32,97 | MAPK3, JUN, MAPK1, MAPK14, FOS, MAP2K1, RELA, APP, CREB1, TLR4, HMGB1, MAPK8, RPS6KA3, RPS6KA1, MAP3K1, RPS6KA2, RPS6KA5, IRAK1, MYD88, NFKB1, MAP2K4, TLR7, TRAF6, MAP2K3, CD14, MAP3K7, IKBKB, MAP2K6, DUSP4, IKBKG |
| REAC:R-HSA-1257604 | PIP3 activates AKT signaling | 1.06e-15 | 15 | 252 | 491 | 49 | 19,44 | AKT1, MAPK3, TP53, EGFR, CDKN1A, JUN, ESR1, MAPK1, EGF, INS, ERBB2, MTOR, FGF2, SRC, PTEN, PPARG, CREB1, AREG, PSMD9, MET, MDM2, CASP9, FOXO1, EZH2, PSMD1, |

| | | | | | | | | |
|---|---|---|---|---|---|---|---|---|
| | | | | | | | | HDAC1, TSC2, FGF7, FYN, KDM1A, IRAK1, MYD88, IL1RL1, IL33, NRG1, TRAF6, NR4A1, CD80, RICTOR, RPTOR, PIK3R2, PTPN11, PDPK1, RAC1, CD28, CD86, RAC2, MAPKAP1, HDAC2 |
| REAC:R-HSA-5218859 | Regulated Necrosis | 1.32e-15 | 14,9 | 55 | 491 | 24 | 43,64 | TP53, CASP3, IL1B, TNFRSF10B, HMGB1, CYCS, IL18, CASP8, FASLG, HSP90AA1, TNFSF10, CASP1, IL1A, RIPK3, TNFRSF10A, FAS, MLKL, IRF1, RIPK1, BIRC3, FADD, GZMB, TP63, ELANE |
| REAC:R-HSA-168188 | Toll Like Receptor TLR6:TLR2 Cascade | 1.97e-15 | 14,7 | 100 | 491 | 31 | 31 | MAPK3, JUN, MAPK1, MAPK14, FOS, MAP2K1, RELA, APP, CREB1, TLR4, HMGB1, MAPK8, RPS6KA3, TLR2, RPS6KA1, MAP3K1, RPS6KA2, RPS6KA5, IRAK1, MYD88, NFKB1, MAP2K4, TRAF6, MAP2K3, CD14, MAP3K7, IKBKB, MAP2K6, DUSP4, TIRAP, IKBKG |
| REAC:R-HSA-166058 | MyD88:MAL(TIRAP) cascade initiated on plasma membrane | 1.97e-15 | 14,7 | 100 | 491 | 31 | 31 | MAPK3, JUN, MAPK1, MAPK14, FOS, MAP2K1, RELA, APP, CREB1, TLR4, HMGB1, MAPK8, RPS6KA3, TLR2, RPS6KA1, MAP3K1, |

| | | | | | | | | |
|---|---|---|---|---|---|---|---|---|
| | | | | | | | | RPS6KA2, RPS6KA5, IRAK1, MYD88, NFKB1, MAP2K4, TRAF6, MAP2K3, CD14, MAP3K7, IKBKB, MAP2K6, DUSP4, TIRAP, IKBKG |
| REAC:R-HSA-168138 | Toll Like Receptor 9 (TLR9) Cascade | 2.76e-15 | 14,6 | 94 | 491 | 30 | 31,91 | MAPK3, JUN, MAPK1, MAPK14, FOS, MAP2K1, RELA, APP, CREB1, TLR4, HMGB1, MAPK8, RPS6KA3, RPS6KA1, MAP3K1, RPS6KA2, RPS6KA5, IRAK1, MYD88, NFKB1, MAP2K4, TLR7, TRAF6, MAP2K3, CD14, MAP3K7, IKBKB, MAP2K6, DUSP4, IKBKG |
| REAC:R-HSA-5357801 | Programmed Cell Death | 7.61e-15 | 14,1 | 205 | 491 | 43 | 20,98 | AKT1, MAPK3, TP53, MAPK1, STAT3, CASP3, IL1B, CDH1, TNFRSF10B, TLR4, HMGB1, CYCS, MAPK8, IL18, PSMD9, CASP8, FASLG, HSP90AA1, CASP9, TNFSF10, CASP1, DNM1L, TJP1, PSMD1, IL1A, PTK2, RIPK3, TNFRSF10A, DAPK2, OCLN, FAS, APAF1, MLKL, IRF1, E2F1, RIPK1, BIRC3, FADD, GZMB, C1QBP, CD14, TP63, ELANE |

| | | | | | | | | |
|---|---|---|---|---|---|---|---|---|
| REAC:R-HSA-168142 | Toll Like Receptor 10 (TLR10) Cascade | 9.85e-15 | 14 | 84 | 491 | 28 | 33,33 | MAPK3, JUN, MAPK1, MAPK14, FOS, MAP2K1, RELA, APP, CREB1, HMGB1, MAPK8, RPS6KA3, RPS6KA1, MAP3K1, RPS6KA2, RPS6KA5, IRAK1, MYD88, NFKB1, MAP2K4, TLR5, TRAF6, MAP2K3, MAP3K7, IKBKB, MAP2K6, DUSP4, IKBKG |
| REAC:R-HSA-168176 | Toll Like Receptor 5 (TLR5) Cascade | 9.85e-15 | 14 | 84 | 491 | 28 | 33,33 | MAPK3, JUN, MAPK1, MAPK14, FOS, MAP2K1, RELA, APP, CREB1, HMGB1, MAPK8, RPS6KA3, RPS6KA1, MAP3K1, RPS6KA2, RPS6KA5, IRAK1, MYD88, NFKB1, MAP2K4, TLR5, TRAF6, MAP2K3, MAP3K7, IKBKB, MAP2K6, DUSP4, IKBKG |
| REAC:R-HSA-975871 | MyD88 cascade initiated on plasma membrane | 9.85e-15 | 14 | 84 | 491 | 28 | 33,33 | MAPK3, JUN, MAPK1, MAPK14, FOS, MAP2K1, RELA, APP, CREB1, HMGB1, MAPK8, RPS6KA3, RPS6KA1, MAP3K1, RPS6KA2, RPS6KA5, IRAK1, MYD88, NFKB1, MAP2K4, TLR5, TRAF6, MAP2K3, MAP3K7, IKBKB, MAP2K6, DUSP4, IKBKG |

| | | | | | | | | |
|---|---|---|---|---|---|---|---|---|
| REAC:R-HSA-5663202 | Diseases of signal transduction by growth factor receptors and second messengers | 3.05e-14 | 13,5 | 417 | 491 | 62 | 14,87 | AKT1, MAPK3, EGFR, CDKN1A, ESR1, MAPK1, STAT3, EGF, ERBB2, HRAS, JUNB, RAF1, MTOR, FGF2, KRAS, SRC, MAP2K1, PTEN, CREB1, AREG, FN1, PSMD9, MET, EIF2AK3, SMAD3, NPM1, HSP90AA1, MDM2, CASP9, STAT1, ALK, FOXO1, SQSTM1, FGFR2, FGFR1, PSMD1, HDAC1, TSC2, FGF7, FYN, HES1, ATG7, CEBPB, HEY2, NRG1, NR4A1, PHB, CD80, RICTOR, PIK3R2, JAG1, PTPN11, PDPK1, RAC1, MPRIP, WNT3A, CD28, CD86, RAC2, CREBBP, MAPKAP1, HDAC2 |
| REAC:R-HSA-380108 | Chemokine receptors bind chemokines | 5.67e-14 | 13,2 | 57 | 491 | 23 | 40,35 | CXCL8, CCL2, CXCR4, CXCL12, CCR7, CCL11, CXCL10, CCR5, CXCL1, CCL5, CCL22, CXCR3, CXCR2, CCL4, CXCR1, XCR1, CXCL9, CCL3, PPBP, CCL20, XCL2, CCL17, CCR3 |
| REAC:R-HSA-9006925 | Intracellular signaling by second messengers | 1.20e-13 | 12,9 | 292 | 491 | 50 | 17,12 | AKT1, MAPK3, TP53, EGFR, CDKN1A, JUN, ESR1, MAPK1, EGF, INS, ERBB2, MTOR, FGF2, SRC, PTEN, PPARG, CREB1, AREG, PSMD9, MET, MDM2, CASP9, FOXO1, EZH2, PSMD1, |

| | | | | | | | | |
|---|---|---|---|---|---|---|---|---|
| | | | | | | | | HDAC1, TSC2, FGF7, FYN, KDM1A, IRAK1, MYD88, IL1RL1, IL33, NRG1, GRK2, TRAF6, NR4A1, CD80, RICTOR, RPTOR, PIK3R2, PTPN11, PDPK1, RAC1, CD28, CD86, RAC2, MAPKAP1, HDAC2 |
| REAC:R-HSA-199418 | Negative regulation of the PI3K/AKT network | 1.22e-12 | 11,9 | 99 | 491 | 28 | 28,28 | AKT1, MAPK3, EGFR, ESR1, MAPK1, EGF, INS, ERBB2, FGF2, SRC, PTEN, AREG, MET, FGF7, FYN, IRAK1, MYD88, IL1RL1, IL33, NRG1, TRAF6, CD80, PIK3R2, PTPN11, RAC1, CD28, CD86, RAC2 |
| REAC:R-HSA-6811558 | PI5P, PP2A and IER3 Regulate PI3K/AKT Signaling | 1.41e-12 | 11,9 | 92 | 491 | 27 | 29,35 | AKT1, MAPK3, EGFR, ESR1, MAPK1, EGF, INS, ERBB2, FGF2, SRC, AREG, MET, FGF7, FYN, IRAK1, MYD88, IL1RL1, IL33, NRG1, TRAF6, CD80, PIK3R2, PTPN11, RAC1, CD28, CD86, RAC2 |
| REAC:R-HSA-448424 | Interleukin-17 signaling | 1.42e-12 | 11,8 | 71 | 491 | 24 | 33,8 | MAPK3, JUN, MAPK1, MAPK14, FOS, MAP2K1, CREB1, MAPK8, RPS6KA3, RPS6KA1, RPS6KA2, IL17C, RPS6KA5, IRAK1, NFKB1, MAP2K4, IL17RA, TRAF6, MAP2K3, MAP3K7, IKBKB, MAP2K6, |

| | | | | | | | | |
|---|---|---|---|---|---|---|---|---|
| | | | | | | | | DUSP4, IKBKG |
| REAC:R-HSA-168643 | Nucleotide-binding domain, leucine rich repeat containing receptor (NLR) signaling pathways | 7.22e-12 | 11,1 | 56 | 491 | 21 | 37,5 | MAPK14, HMOX1, RELA, APP, NLRP3, CASP8, CASP9, CASP1, AIM2, IRAK1, NFKB1, TRAF6, BIRC3, MAP3K7, TXN, NLRC4, CYLD, IKBKB, MAP2K6, PYCARD, IKBKG |
| REAC:R-HSA-450294 | MAP kinase activation | 9.57e-12 | 11 | 63 | 491 | 22 | 34,92 | MAPK3, JUN, MAPK1, MAPK14, FOS, MAP2K1, CREB1, MAPK8, RPS6KA3, RPS6KA1, RPS6KA2, RPS6KA5, IRAK1, NFKB1, MAP2K4, TRAF6, MAP2K3, MAP3K7, IKBKB, MAP2K6, DUSP4, IKBKG |
| REAC:R-HSA-375276 | Peptide ligand-binding receptors | 4.26e-11 | 10,4 | 195 | 491 | 37 | 18,97 | CXCL8, CCL2, APP, CXCR4, CXCL12, CCR7, CCL11, GRP, CXCL10, F2R, CCR5, CXCL1, AGTR1, TAC1, FPR2, CCL5, OPRM1, CCL22, C5AR1, C3, CXCR3, C5, CXCR2, F2RL3, NPY, CCL4, CXCR1, XCR1, CXCL9, CCL3, GAL, PPBP, CCL20, XCL2, CCL17, ANXA1, CCR3 |

| | | | | | | | | |
|---|---|---|---|---|---|---|---|---|
| REAC:R-HSA-446652 | Interleukin-1 family signaling | 3.16e-10 | 9,5 | 139 | 491 | 30 | 21,58 | STAT3, MAP2K1, IL1B, RELA, APP, IL4, HMGB1, MAPK8, IL18, PSMD9, SMAD3, IL13, CASP1, SQSTM1, PSMD1, IL1A, IRAK1, MYD88, IL1RL1, IL33, NFKB1, MAP2K4, TRAF6, PTPN11, MAP3K7, IKBKB, MAP2K6, IL1RN, IL1RAPL1, IKBKG |
| REAC:R-HSA-73887 | Death Receptor Signalling | 3.85e-10 | 9,4 | 140 | 491 | 30 | 21,43 | TNF, CASP3, RELA, TNFRSF10B, MAPK8, CASP8, FASLG, TNFSF10, SQSTM1, CFLAR, HDAC1, TNFRSF10A, FAS, IRAK1, TRAF1, ARHGEF7, RHOA, MYD88, NFKB1, TNFRSF1A, RIPK1, TRAF6, BIRC3, FADD, RAC1, MAP3K7, CYLD, IKBKB, HDAC2, IKBKG |
| REAC:R-HSA-109581 | Apoptosis | 1.39e-09 | 8,9 | 176 | 491 | 33 | 18,75 | AKT1, MAPK3, TP53, MAPK1, STAT3, CASP3, CDH1, TNFRSF10B, TLR4, HMGB1, CYCS, MAPK8, PSMD9, CASP8, FASLG, CASP9, TNFSF10, DNM1L, TJP1, PSMD1, PTK2, TNFRSF10A, DAPK2, OCLN, FAS, APAF1, E2F1, RIPK1, FADD, GZMB, C1QBP, CD14, TP63 |

| | | | | | | | | |
|---|---|---|---|---|---|---|---|---|
| REAC:R-HSA-5357769 | Caspase activation via extrinsic apoptotic signalling pathway | 5.21e-09 | 8,3 | 25 | 491 | 13 | 52 | CASP3, TNFRSF10B, TLR4, CASP8, FASLG, CASP9, TNFSF10, TNFRSF10A, DAPK2, FAS, RIPK1, FADD, CD14 |
| REAC:R-HSA-389356 | CD28 co-stimulation | 9.54e-09 | 8 | 31 | 491 | 14 | 45,16 | AKT1, GRAP2, MTOR, PAK1, FYN, CDC42, CD80, RICTOR, PIK3R2, PDPK1, RAC1, CD28, CD86, MAPKAP1 |
| REAC:R-HSA-212436 | Generic Transcription Pathway | 9.59e-09 | 8 | 1221 | 491 | 106 | 8,68 | AKT1, MAPK3, TP53, IL6, EGFR, CDKN1A, JUN, ESR1, MAPK1, MAPK14, FOS, INS, BDNF, ERBB2, JUNB, MT-CO2, SIRT1, MTOR, KRAS, SRC, APOE, PTEN, RELA, PPARG, TNFRSF10B, CAV1, CREB1, PARP1, PPARA, SPP1, CYCS, ABL1, SP1, SERPINE1, MMP13, PSMD9, MET, ATM, FASLG, SMAD3, NPM1, MDM2, STAT1, FOXO1, CASP1, EZH2, IFNG, GRP, IL2, TJP1, CDK1, PSMD1, HDAC1, IGFBP3, TNFRSF10A, OCLN, FAS, TSC2, GTF2H1, HES1, CEBPB, SOCS3, HEY2, IRAK1, APAF1, OPRM1, CSF2, NFKB1, LRPPRC, VDR, E2F1, FOXP3, NR3C1, IL3, CDK9, HSPD1, SUMO1, |

| | | | | | | | | |
|---|---|---|---|---|---|---|---|---|
| | | | | | | | | NR4A1, RICTOR, RPTOR, PTPN1, COL1A1, NR0B2, JAG1, NPY, NR2C2, PTPN11, PDPK1, TXN, IL2RA, BCL6, NLRC4, MAP2K6, MAF, RARB, GATA2, MED12, RARA, LGALS3, CCNA1, CREBBP, TP63, MAPKAP1, EHMT1, HDAC2, FKBP5 |
| REAC:R-HSA-9009391 | Extra-nuclear estrogen signaling | 2.45e-08 | 7,6 | 73 | 491 | 20 | 27,4 | AKT1, MAPK3, MMP9, EGFR, ESR1, MAPK1, EGF, FOS, MMP2, HRAS, SRC, CAV1, CREB1, AREG, HSP90AA1, SPHK1, PTK2, PIK3R2, XPO1, PDPK1 |
| REAC:R-HSA-140534 | Caspase activation via Death Receptors in the presence of ligand | 4.56e-08 | 7,3 | 15 | 491 | 10 | 66,67 | TNFRSF10B, TLR4, CASP8, FASLG, TNFSF10, TNFRSF10A, FAS, RIPK1, FADD, CD14 |
| REAC:R-HSA-166520 | Signaling by NTRKs | 8.26e-08 | 7,1 | 131 | 491 | 26 | 19,85 | MAPK3, MAPK1, MAPK14, STAT3, CRK, FOS, BDNF, JUND, HRAS, JUNB, FOSB, SRC, MAP2K1, CREB1, RPS6KA3, RPS6KA1, ID1, RPS6KA2, RPS6KA5, F3, RHOA, PIK3R2, PTPN11, RAC1, DUSP4, ID2 |

| | | | | | | | | |
|---|---|---|---|---|---|---|---|---|
| REAC:R-HSA-2559583 | Cellular Senescence | 1.15e-07 | 6,9 | 195 | 491 | 32 | 16,41 | MAPK3, TP53, IL6, CDKN1A, JUN, MAPK1, MAPK14, STAT3, FOS, CXCL8, RELA, MAPK8, SP1, ATM, RPS6KA3, MDM2, RPS6KA1, EZH2, ID1, MAP3K5, IL1A, RPS6KA2, CEBPB, NFKB1, MAP2K4, E2F1, MAP2K3, TXN, MAP2K6, CCNA1, IFNB1, EHMT1 |
| REAC:R-HSA-8953897 | Cellular responses to stimuli | 1.70e-07 | 6,8 | 765 | 491 | 74 | 9,67 | MAPK3, TP53, IL6, NFE2L2, CDKN1A, JUN, MAPK1, MAPK14, STAT3, FOS, HIF1A, CXCL8, HMOX1, MT-CO2, SIRT1, MTOR, CCL2, RELA, HSPA4, CREB1, TLR4, PPARA, CYCS, MAPK8, SOD1, NLRP3, SP1, DCTN6, PSMD9, ATM, EIF2AK3, HSP90AA1, RPS6KA3, MDM2, ALB, RPS6KA1, HSPA1A, ATF3, EZH2, KEAP1, ID1, MAP3K5, PSMD1, MBTPS1, IL1A, CYBB, DCTN4, RPS6KA2, NUP62, HERPUD1, CEBPB, P4HB, FKBP4, NFKB1, LRPPRC, MAP2K4, E2F1, NR3C1, HSPA14, RPTOR, MAP2K3, CSRP1, XPO1, CALR, ATP7A, TXN, MAP2K6, HIF1AN, CCNA1, CREBBP, IFNB1, ERN1, EHMT1, FKBP5 |

| | | | | | | | | |
|---|---|---|---|---|---|---|---|---|
| REAC:R-HSA-2262752 | Cellular responses to stress | 1.83e-07 | 6,7 | 751 | 491 | 73 | 9,72 | MAPK3, TP53, IL6, NFE2L2, CDKN1A, JUN, MAPK1, MAPK14, STAT3, FOS, HIF1A, CXCL8, HMOX1, MT-CO2, SIRT1, MTOR, CCL2, RELA, HSPA4, CREB1, TLR4, PPARA, CYCS, MAPK8, SOD1, NLRP3, SP1, DCTN6, PSMD9, ATM, EIF2AK3, HSP90AA1, RPS6KA3, MDM2, ALB, RPS6KA1, HSPA1A, ATF3, EZH2, KEAP1, ID1, MAP3K5, PSMD1, MBTPS1, IL1A, CYBB, DCTN4, RPS6KA2, NUP62, HERPUD1, CEBPB, P4HB, FKBP4, NFKB1, LRPPRC, MAP2K4, E2F1, NR3C1, HSPA14, RPTOR, MAP2K3, XPO1, CALR, ATP7A, TXN, MAP2K6, HIF1AN, CCNA1, CREBBP, IFNB1, ERN1, EHMT1, FKBP5 |
| REAC:R-HSA-2219530 | Constitutive Signaling by Aberrant PI3K in Cancer | 1.90e-07 | 6,7 | 65 | 491 | 18 | 27,69 | EGFR, ESR1, EGF, ERBB2, FGF2, SRC, AREG, MET, FGF7, FYN, NRG1, CD80, PIK3R2, PTPN11, RAC1, CD28, CD86, RAC2 |
| REAC:R-HSA-5674 | Constitutive Signaling by AKT1 E17K in Cancer | 2.00e-07 | 6,7 | 26 | 491 | 12 | 46,15 | AKT1, CDKN1A, MTOR, CREB1, MDM2, CASP9, FOXO1, TSC2, NR4A1, RICTOR, PDPK1, MAPKAP1 |

| | | | | | | | | |
|---|---|---|---|---|---|---|---|---|
| 400 | | | | | | | | |
| REAC:R-HSA-9660826 | Purinergic signaling in leishmaniasis infection | 2.00e-07 | 6,7 | 26 | 491 | 12 | 46,15 | HMOX1, IL1B, RELA, APP, IL18, NLRP3, CASP1, IL1A, NFKB1, C3, TXN, PYCARD |
| REAC:R-HSA-9664424 | Cell recruitment (pro-inflammatory response) | 2.00e-07 | 6,7 | 26 | 491 | 12 | 46,15 | HMOX1, IL1B, RELA, APP, IL18, NLRP3, CASP1, IL1A, NFKB1, C3, TXN, PYCARD |
| REAC:R-HSA-76002 | Platelet activation, signaling and aggregation | 2.36e-07 | 6,6 | 258 | 491 | 37 | 14,34 | AKT1, MAPK3, MAPK1, MAPK14, EGF, CRK, IGF1, RAF1, SRC, APP, SOD1, SERPINE1, TIMP1, FN1, ALB, ANXA5, F2R, PECAM1, CD9, PTK2, FYN, CAP1, RHOA, CDC42, RAPGEF3, PTPN1, PIK3R2, COL1A1, F2RL3, PTPN11, PDPK1, RAC1, SERPINA1, CFL1, PPBP, PLG, RAC2 |
| REAC:R-HSA-388841 | Costimulation by the CD28 family | 3.29e-07 | 6,5 | 67 | 491 | 18 | 26,87 | AKT1, GRAP2, MTOR, CD274, CD4, PAK1, FYN, CDC42, PDCD1, CD80, RICTOR, PIK3R2, PTPN11, PDPK1, RAC1, CD28, CD86, MAPKAP1 |

| | | | | | | | | |
|---|---|---|---|---|---|---|---|---|
| REAC:R-HSA-5620971 | Pyroptosis | 3.46e-07 | 6,5 | 27 | 491 | 12 | 44,44 | TP53, CASP3, IL1B, HMGB1, CYCS, IL18, CASP1, IL1A, IRF1, GZMB, TP63, ELANE |
| REAC:R-HSA-109582 | Hemostasis | 3.72e-07 | 6,4 | 672 | 491 | 67 | 9,97 | AKT1, MAPK3, TP53, MAPK1, MAPK14, EGF, CRK, IGF1, HRAS, RAF1, SRC, CD44, APP, TNFRSF10B, CAV1, NOS2, ABL1, SOD1, SERPINE1, TIMP1, FN1, ALB, ANXA5, PSG1, F2R, HDAC1, KIF2A, PECAM1, CD9, PTK2, TNFRSF10A, IFNA1, FYN, PROC, RAB5A, KDM1A, BSG, CAP1, F3, RHOA, EPCAM, SDC1, CDC42, ITGAM, IRF1, RAPGEF3, PTPN1, PIK3R2, C1QBP, COL1A1, F2RL3, PTPN11, PDPK1, RAC1, ANXA2, THBD, SERPINA1, CFL1, MIF, PPBP, PLG, GATA2, RAC2, IFNB1, HDAC2, ITGAX |
| REAC:R-HSA-5218900 | CASP8 activity is inhibited | 3.86e-07 | 6,4 | 10 | 491 | 8 | 80 | TNFRSF10B, CASP8, FASLG, TNFSF10, TNFRSF10A, FAS, RIPK1, FADD |

| | | | | | | | | |
|---|---|---|---|---|---|---|---|---|
| REAC:R-HSA-69416 | Dimerization of procaspase-8 | 3.86e-07 | 6,4 | 10 | 491 | 8 | 80 | TNFRSF10B, CASP8, FASLG, TNFSF10, TNFRSF10A, FAS, RIPK1, FADD |
| REAC:R-HSA-3371378 | Regulation by c-FLIP | 3.86e-07 | 6,4 | 10 | 491 | 8 | 80 | TNFRSF10B, CASP8, FASLG, TNFSF10, TNFRSF10A, FAS, RIPK1, FADD |
| REAC:R-HSA-5675482 | Regulation of necroptotic cell death | 5.80e-07 | 6,2 | 28 | 491 | 12 | 42,86 | TNFRSF10B, CASP8, FASLG, HSP90AA1, TNFSF10, RIPK3, TNFRSF10A, FAS, MLKL, RIPK1, BIRC3, FADD |
| REAC:R-HSA-5213460 | RIPK1-mediated regulated necrosis | 5.80e-07 | 6,2 | 28 | 491 | 12 | 42,86 | TNFRSF10B, CASP8, FASLG, HSP90AA1, TNFSF10, RIPK3, TNFRSF10A, FAS, MLKL, RIPK1, BIRC3, FADD |
| REAC:R-HSA-5683057 | MAPK family signaling cascades | 9.23e-07 | 6 | 308 | 491 | 40 | 12,99 | MAPK3, IL6, EGFR, JUN, MAPK1, EGF, ERBB2, HRAS, RAF1, FGF2, KRAS, SRC, MAP2K1, AREG, FN1, PSMD9, MET, FOXO1, JAK1, IL2, CDK1, PSMD1, PTK2, PAK1, FGF7, FYN, JAK3, CDC42, NRG1, CSF2, IL3, PHB, PIK3R2, XPO1, PTPN11, RAC1, IL2RA, DUSP4, IL5, IGF2BP1 |

| | | | | | | | | |
|---|---|---|---|---|---|---|---|---|
| REAC:R-HSA-73857 | RNA Polymerase II Transcription | 1.18e-06 | 5,9 | 1342 | 491 | 107 | 7,97 | AKT1, MAPK3, TP53, IL6, EGFR, CDKN1A, JUN, ESR1, MAPK1, MAPK14, FOS, INS, BDNF, ERBB2, JUNB, MT-CO2, SIRT1, MTOR, KRAS, SRC, APOE, PTEN, RELA, PPARG, TNFRSF10B, CAV1, CREB1, PARP1, PPARA, SPP1, CYCS, ABL1, SP1, SERPINE1, MMP13, PSMD9, MET, ATM, FASLG, SMAD3, NPM1, MDM2, STAT1, FOXO1, CASP1, EZH2, IFNG, GRP, IL2, TJP1, CDK1, PSMD1, HDAC1, IGFBP3, TNFRSF10A, OCLN, FAS, TSC2, GTF2H1, HES1, CEBPB, SOCS3, HEY2, IRAK1, APAF1, OPRM1, CSF2, NFKB1, LRPPRC, VDR, E2F1, FOXP3, NR3C1, IL3, CDK9, HSPD1, SUMO1, NR4A1, RICTOR, RPTOR, PTPN1, COL1A1, NR0B2, JAG1, NPY, NR2C2, PTPN11, PDPK1, TXN, IL2RA, BCL6, NLRC4, MAP2K6, SRSF1, MAF, RARB, GATA2, MED12, RARA, LGALS3, CCNA1, CREBBP, TP63, MAPKAP1, EHMT1, HDAC2, FKBP5 |

| | | | | | | | | |
|---|---|---|---|---|---|---|---|---|
| REAC:R-HSA-168928 | DDX58/IFIH1-mediated induction of interferon-alpha/beta | 1.67e-06 | 5,8 | 82 | 491 | 19 | 23,17 | RELA, APP, HMGB1, CASP8, HSP90AA1, IFNA1, MAP3K1, NFKB1, TRAF3, ATG5, RIPK1, TRAF6, FADD, CYLD, IKBKB, CREBBP, IFNB1, IKBKG |
| REAC:R-HSA-8939211 | ESR-mediated signaling | 1.83e-06 | 5,7 | 217 | 491 | 32 | 14,75 | AKT1, MAPK3, MMP9, EGFR, JUN, ESR1, MAPK1, EGF, FOS, MMP2, JUND, HRAS, FOSB, SRC, CAV1, CREB1, AREG, CXCL12, SP1, HSP90AA1, SPHK1, HDAC1, PTK2, KDM1A, FKBP4, CDK9, PIK3R2, XPO1, PDPK1, STAG1, CREBBP, FKBP5 |
| REAC:R-HSA-9006931 | Signaling by Nuclear Receptors | 2.05e-06 | 5,7 | 291 | 491 | 38 | 13,06 | AKT1, MAPK3, MMP9, EGFR, JUN, ESR1, MAPK1, EGF, FOS, MMP2, JUND, HRAS, FOSB, SRC, APOE, CAV1, CREB1, AREG, CXCL12, SP1, ABCA1, HSP90AA1, SPHK1, HDAC1, PTK2, KDM1A, ALDH1A3, FKBP4, CDK9, PIK3R2, XPO1, ALDH1A1, PDPK1, STAG1, RARB, RARA, CREBBP, FKBP5 |

| | | | | | | | | |
|---|---|---|---|---|---|---|---|---|
| REAC:R-HSA-74160 | Gene expression (Transcription) | 2.13e-06 | 5,7 | 1478 | 491 | 114 | 7,71 | AKT1, MAPK3, TP53, IL6, EGFR, CDKN1A, JUN, ESR1, MAPK1, MAPK14, FOS, INS, BDNF, ERBB2, JUNB, MT-CO2, SIRT1, MTOR, KRAS, SRC, APOE, PTEN, RELA, PPARG, TNFRSF10B, CAV1, CREB1, PARP1, PPARA, SPP1, CYCS, ABL1, SP1, SERPINE1, MMP13, PSMD9, MET, ATM, FASLG, SMAD3, NPM1, HSP90AA1, MDM2, DNMT1, STAT1, FOXO1, CASP1, EZH2, IFNG, GRP, IL2, TJP1, CDK1, PSMD1, HDAC1, IGFBP3, TNFRSF10A, OCLN, FAS, NUP62, TSC2, GTF2H1, HES1, CEBPB, SOCS3, HEY2, IRAK1, APAF1, OPRM1, CSF2, NFKB1, LRPPRC, VDR, DNMT3B, E2F1, FOXP3, NR3C1, IL3, CDK9, HSPD1, SUMO1, NR4A1, RICTOR, RPTOR, PRKRA, PTPN1, COL1A1, NR0B2, JAG1, DNMT3A, NPY, NR2C2, PTPN11, PDPK1, TXN, IL2RA, BCL6, NLRC4, MAP2K6, SRSF1, MAF, RARB, GATA2, MED12, RARA, LGALS3, CCNA1, MBD2, CREBBP, TP63, |

| | | | | | | | | |
|---|---|---|---|---|---|---|---|---|
| | | | | | | | | MAPKAP1, EHMT1, HDAC2, FKBP5 |
| REAC:R-HSA-9006927 | Signaling by Non-Receptor Tyrosine Kinases | 2.27e-06 | 5,6 | 51 | 491 | 15 | 29,41 | AKT1, EGFR, STAT3, EGF, CRK, ERBB2, HIF1A, HRAS, KHDRBS1, SOCS3, RHOA, NRG1, NR3C1, PTPN1, RAC1 |
| REAC:R-HSA-8848021 | Signaling by PTK6 | 2.27e-06 | 5,6 | 51 | 491 | 15 | 29,41 | AKT1, EGFR, STAT3, EGF, CRK, ERBB2, HIF1A, HRAS, KHDRBS1, SOCS3, RHOA, NRG1, NR3C1, PTPN1, RAC1 |
| REAC:R-HSA-5602358 | Diseases associated with the TLR signaling cascade | 2.37e-06 | 5,6 | 31 | 491 | 12 | 38,71 | RELA, TLR4, HMGB1, TLR2, MYD88, NFKB1, TLR5, TRAF3, CD14, IKBKB, TIRAP, IKBKG |
| REAC:R-HSA-5260271 | Diseases of Immune System | 2.37e-06 | 5,6 | 31 | 491 | 12 | 38,71 | RELA, TLR4, HMGB1, TLR2, MYD88, NFKB1, TLR5, TRAF3, CD14, IKBKB, TIRAP, IKBKG |

| | | | | | | | | |
|---|---|---|---|---|---|---|---|---|
| REAC:R-HSA-450282 | MAPK targets/ Nuclear events mediated by MAP kinases | 2.37e-06 | 5,6 | 31 | 491 | 12 | 38,71 | MAPK3, JUN, MAPK1, MAPK14, FOS, CREB1, MAPK8, RPS6KA3, RPS6KA1, RPS6KA2, RPS6KA5, DUSP4 |
| REAC:R-HSA-187037 | Signaling by NTRK1 (TRKA) | 2.66e-06 | 5,6 | 112 | 491 | 22 | 19,64 | MAPK3, MAPK1, MAPK14, STAT3, CRK, FOS, JUND, HRAS, JUNB, FOSB, MAP2K1, CREB1, RPS6KA3, RPS6KA1, ID1, RPS6KA2, RPS6KA5, F3, RHOA, PIK3R2, DUSP4, ID2 |
| REAC:R-HSA-881907 | Gastrin-CREB signalling pathway via PKC and MAPK | 3.58e-06 | 5,4 | 16 | 491 | 9 | 56,25 | MAPK3, EGFR, MAPK1, HRAS, CREB1, GAST, RPS6KA3, RPS6KA1, RPS6KA2 |
| REAC:R-HSA-622312 | Inflammasomes | 4.18e-06 | 5,4 | 21 | 491 | 10 | 47,62 | HMOX1, RELA, APP, NLRP3, CASP1, AIM2, NFKB1, TXN, NLRC4, PYCARD |
| REAC:R-HSA-198725 | Nuclear Events (kinase and transcription factor activation) | 4.36e-06 | 5,4 | 61 | 491 | 16 | 26,23 | MAPK3, MAPK1, MAPK14, FOS, JUND, JUNB, FOSB, CREB1, RPS6KA3, RPS6KA1, ID1, RPS6KA2, RPS6KA5, F3, DUSP4, ID2 |
| REAC:R-HSA | Intrinsic Pathway for Apoptosis | 5.44e-06 | 5,3 | 54 | 491 | 15 | 27,78 | AKT1, MAPK3, TP53, MAPK1, STAT3, CASP3, CYCS, MAPK8, CASP8, |

| | | | | | | | | |
|---|---|---|---|---|---|---|---|---|
| - 1096 06 | | | | | | | | CASP9, APAF1, E2F1, GZMB, C1QBP, TP63 |
| REAC:R-HSA-389357 | CD28 dependent PI3K/Akt signaling | 7.35e-06 | 5,1 | 22 | 491 | 10 | 45,45 | AKT1, MTOR, FYN, CD80, RICTOR, PIK3R2, PDPK1, CD28, CD86, MAPKAP1 |
| REAC:R-HSA-6798695 | Neutrophil degranulation | 1.25e-05 | 4,9 | 476 | 491 | 50 | 10,5 | MMP9, MAPK1, MAPK14, CD44, MPO, HMGB1, HP, RNASE3, HSP90AA1, TLR2, HSPA1A, TTR, CXCL1, PSMD1, PECAM1, FPR2, CYBB, ATG7, CAP1, RHOA, APAF1, GRN, ITGAM, PKM, CTSB, NFKB1, C5AR1, ARG1, EPX, C3, PTPRC, CXCR2, FCGR2A, CD14, RAC1, CXCR1, LAMP1, ANXA2, SERPINA1, MME, MIF, PYCARD, CD33, PPBP, CD55, AZU1, LGALS3, CD59, ITGAX, ELANE |
| REAC:R-HSA-168638 | NOD1/2 Signaling Pathway | 1.71e-05 | 4,8 | 36 | 491 | 12 | 33,33 | MAPK14, CASP8, CASP9, CASP1, IRAK1, TRAF6, BIRC3, MAP3K7, CYLD, IKBKB, MAP2K6, IKBKG |
| REAC:R-HSA-3730 | Class A/1 (Rhodopsin-like receptors) | 1.79e-05 | 4,7 | 328 | 491 | 39 | 11,89 | CXCL8, CCL2, APP, CXCR4, CXCL12, CCR7, CCL11, GRP, CXCL10, F2R, CCR5, CXCL1, AGTR1, TAC1, FPR2, |

| | | | | | | | | |
|---|---|---|---|---|---|---|---|---|
| 76 | | | | | | | | CCL5, OPRM1, CCL22, C5AR1, HTR2A, C3, RHO, CXCR3, C5, CXCR2, F2RL3, NPY, CCL4, CXCR1, XCR1, CXCL9, CCL3, GAL, PPBP, CCL20, XCL2, CCL17, ANXA1, CCR3 |
| REAC:R-HSA-4420097 | VEGFA-VEGFR2 Pathway | 1.84e-05 | 4,7 | 94 | 491 | 19 | 20,21 | AKT1, MAPK14, CRK, HRAS, MTOR, CAV1, HSP90AA1, SPHK1, PTK2, PAK1, CYBB, FYN, RHOA, CDC42, RICTOR, PIK3R2, PDPK1, RAC1, MAPKAP1 |
| REAC:R-HSA-194138 | Signaling by VEGF | 1.94e-05 | 4,7 | 104 | 491 | 20 | 19,23 | AKT1, MAPK14, CRK, HRAS, MTOR, CAV1, HSP90AA1, SPHK1, PTK2, PAK1, CYBB, FYN, RHOA, NRP1, CDC42, RICTOR, PIK3R2, PDPK1, RAC1, MAPKAP1 |
| REAC:R-HSA-9634638 | Estrogen-dependent nuclear events downstream of ESR-membrane signaling | 2.05e-05 | 4,7 | 24 | 491 | 10 | 41,67 | AKT1, MAPK3, EGFR, MAPK1, EGF, FOS, CREB1, AREG, PTK2, XPO1 |
| REAC:R-HSA-4442 | RSK activation | 3.09e-05 | 4,5 | 7 | 491 | 6 | 85,71 | MAPK3, MAPK1, RPS6KA3, RPS6KA1, RPS6KA2, PDPK1 |

| | | | | | | | | |
|---|---|---|---|---|---|---|---|---|
| 57 | | | | | | | | |
| REAC:R-HSA-1059683 | Interleukin-6 signaling | 5.81e-05 | 4,2 | 11 | 491 | 7 | 63,64 | IL6, STAT3, STAT1, JAK1, SOCS3, IL6ST, PTPN11 |
| REAC:R-HSA-389359 | CD28 dependent Vav1 pathway | 5.81e-05 | 4,2 | 11 | 491 | 7 | 63,64 | PAK1, FYN, CDC42, CD80, RAC1, CD28, CD86 |
| REAC:R-HSA-9020702 | Interleukin-1 signaling | 6.17e-05 | 4,2 | 101 | 491 | 19 | 18,81 | MAP2K1, IL1B, RELA, APP, HMGB1, PSMD9, SQSTM1, PSMD1, IL1A, IRAK1, MYD88, NFKB1, MAP2K4, TRAF6, MAP3K7, IKBKB, MAP2K6, IL1RN, IKBKG |
| REAC:R-HSA-844456 | The NLRP3 inflammasome | 8.65e-05 | 4,1 | 16 | 491 | 8 | 50 | HMOX1, RELA, APP, NLRP3, CASP1, NFKB1, TXN, PYCARD |
| REAC:R-HSA-913531 | Interferon Signaling | 9.55e-05 | 4 | 193 | 491 | 27 | 13,99 | MAPK3, CD44, ICAM1, IFI27, VCAM1, STAT1, IFNG, JAK1, EIF4G1, IFNA1, NUP62, EIF2AK2, MX2, SOCS3, IRF1, SUMO1, EIF4E, PTPN1, HLA-A, PTPN11, FCGR1A, ISG20, STAT2, GBP1, IFNB1, MX1 |

| | | | | | | | | |
|---|---|---|---|---|---|---|---|---|
| REAC:R-HSA-5684996 | MAPK1/MAPK3 signaling | 1.01e-04 | 4 | 269 | 491 | 33 | 12,27 | MAPK3, IL6, EGFR, MAPK1, EGF, ERBB2, HRAS, RAF1, FGF2, KRAS, SRC, MAP2K1, AREG, FN1, PSMD9, MET, JAK1, IL2, CDK1, PSMD1, PTK2, FGF7, FYN, JAK3, NRG1, CSF2, IL3, PHB, PIK3R2, PTPN11, IL2RA, DUSP4, IL5 |
| REAC:R-HSA-9614085 | FOXO-mediated transcription | 1.02e-04 | 4 | 66 | 491 | 15 | 22,73 | AKT1, CDKN1A, INS, SIRT1, CAV1, FASLG, SMAD3, FOXO1, HDAC1, NR3C1, NPY, TXN, BCL6, CREBBP, HDAC2 |
| REAC:R-HSA-5621481 | C-type lectin receptors (CLRs) | 1.04e-04 | 4 | 136 | 491 | 22 | 16,18 | HRAS, RAF1, IL1B, RELA, PSMD9, CASP8, MUC5AC, MUC5B, PSMD1, PAK1, FYN, MUC15, RPS6KA5, NFKB1, TRAF6, MUC1, PDPK1, MAP3K7, IKBKB, PYCARD, CREBBP, IKBKG |
| REAC:R-HSA-75158 | TRAIL signaling | 1.19e-04 | 3,9 | 8 | 491 | 6 | 75 | TNFRSF10B, CASP8, TNFSF10, CFLAR, TNFRSF10A, FADD |
| REAC:R-HSA-8940 | RUNX2 regulates osteoblast differentiation | 1.92e-04 | 3,7 | 23 | 491 | 9 | 39,13 | MAPK3, MAPK1, SRC, ABL1, GRP, HES1, HEY2, COL1A1, MAF |

| | | | | | | | | |
|---|---|---|---|---|---|---|---|---|
| 973 | | | | | | | | |
| REAC:R-HSA-5633008 | TP53 Regulates Transcription of Cell Death Genes | 2.06e-04 | 3,7 | 44 | 491 | 12 | 27,27 | TP53, TNFRSF10B, ATM, CASP1, IGFBP3, TNFRSF10A, FAS, APAF1, BCL6, NLRC4, CREBBP, TP63 |
| REAC:R-HSA-372790 | Signaling by GPCR | 2.24e-04 | 3,6 | 693 | 491 | 61 | 8,8 | AKT1, MAPK3, EGFR, MAPK1, HRAS, CXCL8, SRC, CCL2, APP, CREB1, CXCR4, CXCL12, GAST, RPS6KA3, CCR7, RPS6KA1, CCL11, GRP, CXCL10, F2R, CCR5, CXCL1, AGTR1, TAC1, PAK1, FPR2, RPS6KA2, ARHGEF7, RHOA, CCL5, CDC42, OPRM1, CCL22, C5AR1, HTR2A, C3, RHO, GRK2, CXCR3, C5, CXCR2, PIK3R2, F2RL3, NPY, CCL4, PDPK1, CXCR1, XCR1, CXCL9, CCL3, CCL4L2, GAL, WNT3A, PPBP, GRM5, CCL20, CD55, XCL2, CCL17, ANXA1, CCR3 |
| REAC:R-HSA-3700989 | Transcriptional Regulation by TP53 | 2.48e-04 | 3,6 | 362 | 491 | 39 | 10,77 | AKT1, TP53, CDKN1A, JUN, MAPK14, FOS, MT-CO2, MTOR, PTEN, TNFRSF10B, CYCS, ATM, NPM1, MDM2, CASP1, CDK1, HDAC1, IGFBP3, TNFRSF10A, FAS, TSC2, GTF2H1, |

| | | | | | | | | |
|---|---|---|---|---|---|---|---|---|
| | | | | | | | | APAF1, LRPPRC, E2F1, CDK9, RICTOR, RPTOR, PDPK1, TXN, BCL6, NLRC4, MAP2K6, CCNA1, CREBBP, TP63, MAPKAP1, EHMT1, HDAC2 |
| REAC:R-HSA-9725371 | Nuclear events stimulated by ALK signaling in cancer | 2.71e-04 | 3,6 | 18 | 491 | 8 | 44,44 | MAPK3, MAPK1, STAT3, JUNB, FN1, NPM1, ALK, CEBPB |
| REAC:R-HSA-2562578 | TRIF-mediated programmed cell death | 3.43e-04 | 3,5 | 9 | 491 | 6 | 66,67 | TLR4, CASP8, RIPK3, RIPK1, FADD, CD14 |
| REAC:R-HSA-448706 | Interleukin-1 processing | 3.43e-04 | 3,5 | 9 | 491 | 6 | 66,67 | IL1B, RELA, IL18, CASP1, IL1A, NFKB1 |
| REAC:R-HSA-110056 | MAPK3 (ERK1) activation | 3.43e-04 | 3,5 | 9 | 491 | 6 | 66,67 | MAPK3, IL6, MAP2K1, JAK1, CDK1, PTPN11 |
| REAC:R-HSA-3108232 | SUMO E3 ligases SUMOylate target proteins | 3.91e-04 | 3,4 | 170 | 491 | 24 | 14,12 | TP53, ESR1, RELA, PPARG, PARP1, PPARA, NPM1, MDM2, DNMT1, HDAC1, NUP62, HNRNPK, VDR, DNMT3B, NR3C1, |

| | | | | | | | | |
|---|---|---|---|---|---|---|---|---|
| | | | | | | | | SUMO1, DNMT3A, STAG1, HNRNPC, RARA, CREBBP, HDAC2, SAFB, IKBKG |
| REAC:R-HSA-8854691 | Interleukin-20 family signaling | 4.42e-04 | 3,4 | 25 | 491 | 9 | 36 | STAT3, STAT1, JAK1, IL24, JAK3, IL22, PTPN11, STAT2, IFNL3 |
| REAC:R-HSA-201556 | Signaling by ALK | 4.42e-04 | 3,4 | 25 | 491 | 9 | 36 | STAT3, HIF1A, ALK, CD274, HDAC1, JAK3, MDK, PIK3R2, HDAC2 |
| REAC:R-HSA-1280218 | Adaptive Immune System | 4.67e-04 | 3,3 | 806 | 491 | 67 | 8,31 | AKT1, GRAP2, RNF19A, HRAS, RAF1, MTOR, PTEN, CDH1, RELA, ICAM1, TLR4, CD40LG, HMGB1, DCTN6, PSMD9, TLR2, VCAM1, CD274, CD4, KEAP1, PSMD1, KIF2A, PAK1, CYBB, DCTN4, FYN, CD8A, ATG7, SOCS3, UBE2L3, MYD88, CDC42, CTSB, NFKB1, C3, PDCD1, RAPGEF3, PTPRC, TRAF6, CD80, RICTOR, RBBP6, KIR3DL2, PIK3R2, COL1A1, COL2A1, HLA-A, CD14, CALR, PTPN11, PDPK1, RAC1, FCGR1A, MAP3K7, CTSE, PTPN22, IKBKB, MRC1, CD33, |

| | | | | | | | | |
|---|---|---|---|---|---|---|---|---|
| | | | | | | | | CD28, CD86, TAP1, KLRG1, VASP, MAPKAP1, TIRAP, IKBKG |
| REAC:R-HSA-5660668 | CLEC7A/inflammasome pathway | 5.87e-04 | 3,2 | 6 | 491 | 5 | 83,33 | IL1B, RELA, CASP8, NFKB1, PYCARD |
| REAC:R-HSA-447115 | Interleukin-12 family signaling | 6.40e-04 | 3,2 | 57 | 491 | 13 | 22,81 | STAT3, IL10, SOD1, STAT1, IFNG, JAK1, AIP, IL6ST, CDC42, P4HB, ANXA2, CFL1, MIF |
| REAC:R-HSA-5357905 | Regulation of TNFR1 signaling | 6.68e-04 | 3,2 | 33 | 491 | 10 | 30,3 | TNF, CASP8, TRAF1, TNFRSF1A, RIPK1, BIRC3, FADD, CYLD, IKBKB, IKBKG |
| REAC:R-HSA-5663205 | Infectious disease | 7.08e-04 | 3,2 | 916 | 491 | 73 | 7,97 | MAPK3, IL6, EGFR, JUN, MAPK1, MAPK14, CRK, HMOX1, SRC, MAP2K1, IL1B, CDH1, RELA, APP, SYT1, CREB1, IL10, CXCR4, NOS2, PARP1, MAPK8, ABL1, IL18, NLRP3, PSMD9, MET, NPM1, HSP90AA1, TLR2, BECN1, HSPA1A, CASP1, EZH2, CD4, JAK1, CCR5, PSMD1, |

| | | | | | | | | |
|---|---|---|---|---|---|---|---|---|
| | | | | | | | | HDAC1, IL1A, CD9, PTK2, NUP62, EIF2AK2, GTF2H1, FYN, RAB5A, KDM1A, HNRNPK, JAK3, CDC42, FKBP4, NFKB1, MAP2K4, TLR7, NR3C1, C3, RIPK1, PDCD1, CDK9, SUMO1, FCGR2A, XPO1, CALR, RAC1, FCGR1A, STAT2, TXN, MRC1, PYCARD, CD28, CD163, HDAC2, DPEP2 |
| REAC:R-HSA-5621575 | CD209 (DC-SIGN) signaling | 7.20e-04 | 3,1 | 20 | 491 | 8 | 40 | HRAS, RAF1, RELA, PAK1, FYN, RPS6KA5, NFKB1, CREBBP |
| REAC:R-HSA-2990846 | SUMOylation | 7.39e-04 | 3,1 | 176 | 491 | 24 | 13,64 | TP53, ESR1, RELA, PPARG, PARP1, PPARA, NPM1, MDM2, DNMT1, HDAC1, NUP62, HNRNPK, VDR, DNMT3B, NR3C1, SUMO1, DNMT3A, STAG1, HNRNPC, RARA, CREBBP, HDAC2, SAFB, IKBKG |
| REAC:R-HSA-8878166 | Transcriptional regulation by RUNX2 | 7.60e-04 | 3,1 | 118 | 491 | 19 | 16,1 | AKT1, MAPK3, CDKN1A, ESR1, MAPK1, SRC, ABL1, MMP13, PSMD9, STAT1, GRP, CDK1, PSMD1, HES1, HEY2, NR3C1, COL1A1, MAF, LGALS3 |

| | | | | | | | | |
|---|---|---|---|---|---|---|---|---|
| REAC:R-HSA-450341 | Activation of the AP-1 family of transcription factors | 8.23e-04 | 3,1 | 10 | 491 | 6 | 60 | MAPK3, JUN, MAPK1, MAPK14, FOS, MAPK8 |
| REAC:R-HSA-909733 | Interferon alpha/beta signaling | 9.34e-04 | 3 | 68 | 491 | 14 | 20,59 | IFI27, JAK1, IFNA1, MX2, SOCS3, IRF1, PTPN1, HLA-A, PTPN11, ISG20, STAT2, IFNB1, MX1 |
| REAC:R-HSA-5218920 | VEGFR2 mediated vascular permeability | 9.35e-04 | 3 | 27 | 491 | 9 | 33,33 | AKT1, MTOR, CAV1, HSP90AA1, PAK1, RICTOR, PDPK1, RAC1, MAPKAP1 |
| REAC:R-HSA-75893 | TNF signaling | 9.85e-04 | 3 | 42 | 491 | 11 | 26,19 | TNF, CASP8, TRAF1, TNFRSF1A, RIPK1, BIRC3, FADD, MAP3K7, CYLD, IKBKB, IKBKG |
| REAC:R-HSA-388396 | GPCR downstream signalling | 1.51e-03 | 2,8 | 620 | 491 | 54 | 8,71 | AKT1, MAPK3, EGFR, MAPK1, HRAS, CXCL8, SRC, APP, CREB1, CXCR4, CXCL12, GAST, RPS6KA3, CCR7, RPS6KA1, GRP, CXCL10, F2R, CCR5, CXCL1, AGTR1, TAC1, PAK1, FPR2, RPS6KA2, ARHGEF7, RHOA, CCL5, CDC42, OPRM1, C5AR1, HTR2A, C3, RHO, GRK2, CXCR3, C5, CXCR2, |

| | | | | | | | | |
|---|---|---|---|---|---|---|---|---|
| | | | | | | | | PIK3R2, F2RL3, NPY, CCL4, PDPK1, CXCR1, XCR1, CXCL9, CCL4L2, GAL, PPBP, GRM5, CCL20, XCL2, ANXA1, CCR3 |
| REAC:R-HSA-6804757 | Regulation of TP53 Degradation | 1.62e-03 | 2,8 | 36 | 491 | 10 | 27,78 | AKT1, TP53, MTOR, ATM, MDM2, CDK1, RICTOR, PDPK1, CCNA1, MAPKAP1 |
| REAC:R-HSA-381426 | Regulation of Insulin-like Growth Factor (IGF) transport and uptake by Insulin-like Growth Factor Binding Proteins (IGFBPs) | 1.63e-03 | 2,8 | 124 | 491 | 19 | 15,32 | IL6, IGF1, MMP2, APOE, APP, SPP1, TIMP1, FN1, ALB, KLK3, KLK2, MBTPS1, IGFBP3, PROC, P4HB, C3, KLK1, SERPINA1, PLG |
| REAC:R-HSA-193639 | p75NTR signals via NF-kB | 1.65e-03 | 2,8 | 16 | 491 | 7 | 43,75 | RELA, SQSTM1, IRAK1, MYD88, NFKB1, TRAF6, IKBKB |
| REAC:R-HSA-9022699 | MECP2 regulates neuronal receptors and channels | 1.65e-03 | 2,8 | 16 | 491 | 7 | 43,75 | CREB1, MET, HDAC1, OPRM1, PTPN1, HDAC2, FKBP5 |

| | | | | | | | | |
|---|---|---|---|---|---|---|---|---|
| REAC:R-HSA-937041 | IKK complex recruitment mediated by RIP1 | 1.68e-03 | 2,8 | 22 | 491 | 8 | 36,36 | TLR4, RIPK3, RIPK1, TRAF6, BIRC3, CD14, IKBKB, IKBKG |
| REAC:R-HSA-198753 | ERK/MAPK targets | 1.68e-03 | 2,8 | 22 | 491 | 8 | 36,36 | MAPK3, MAPK1, MAPK14, RPS6KA3, RPS6KA1, RPS6KA2, RPS6KA5, DUSP4 |
| REAC:R-HSA-112409 | RAF-independent MAPK1/3 activation | 1.68e-03 | 2,8 | 22 | 491 | 8 | 36,36 | MAPK3, IL6, MAPK1, MAP2K1, JAK1, CDK1, PTPN11, DUSP4 |
| REAC:R-HSA-5673001 | RAF/MAP kinase cascade | 1.84e-03 | 2,7 | 264 | 491 | 30 | 11,36 | MAPK3, EGFR, MAPK1, EGF, ERBB2, HRAS, RAF1, FGF2, KRAS, SRC, MAP2K1, AREG, FN1, PSMD9, MET, JAK1, IL2, PSMD1, PTK2, FGF7, FYN, JAK3, NRG1, CSF2, IL3, PHB, PIK3R2, IL2RA, DUSP4, IL5 |
| REAC:R-HSA-199920 | CREB phosphorylation | 1.98e-03 | 2,7 | 7 | 491 | 5 | 71,43 | CREB1, RPS6KA3, RPS6KA1, RPS6KA2, RPS6KA5 |
| REAC:R-HSA- | Regulation of TP53 Expression and | 2.13e-03 | 2,7 | 37 | 491 | 10 | 27,03 | AKT1, TP53, MTOR, ATM, MDM2, CDK1, RICTOR, PDPK1, CCNA1, MAPKAP1 |

| | | | | | | | | |
|---|---|---|---|---|---|---|---|---|
| 6806003 | Degradation | | | | | | | |
| REAC:R-HSA-9686347 | Microbial modulation of RIPK1-mediated regulated necrosis | 2.25e-03 | 2,6 | 4 | 491 | 4 | 100 | CASP8, RIPK3, MLKL, RIPK1 |
| REAC:R-HSA-8941326 | RUNX2 regulates bone development | 2.52e-03 | 2,6 | 30 | 491 | 9 | 30 | MAPK3, MAPK1, SRC, ABL1, GRP, HES1, HEY2, COL1A1, MAF |
| REAC:R-HSA-5357956 | TNFR1-induced NFkappaB signaling pathway | 2.52e-03 | 2,6 | 30 | 491 | 9 | 30 | TNF, TRAF1, TNFRSF1A, RIPK1, BIRC3, MAP3K7, CYLD, IKBKB, IKBKG |
| REAC:R-HSA-1810476 | RIP-mediated NFkB activation via ZBP1 | 2.69e-03 | 2,6 | 17 | 491 | 7 | 41,18 | RELA, RIPK3, MYD88, NFKB1, RIPK1, IKBKB, IKBKG |
| REAC:R-HSA-512988 | Interleukin-3, Interleukin-5 and GM-CSF signaling | 3.26e-03 | 2,5 | 47 | 491 | 11 | 23,4 | CRK, JAK1, IL2, FYN, JAK3, CSF2, IL3, PIK3R2, PTPN11, IL2RA, IL5 |
| REAC:R-HSA- | TP53 Regulates Transcription of Death | 3.35e-03 | 2,5 | 12 | 491 | 6 | 50 | TP53, TNFRSF10B, IGFBP3, TNFRSF10A, FAS, TP63 |

| | | | | | | | | |
|---|---|---|---|---|---|---|---|---|
| 6803 211 | Receptors and Ligands | | | | | | | |
| REAC:R-HSA-877312 | Regulation of IFNG signaling | 3.35e-03 | 2,5 | 12 | 491 | 6 | 50 | IFNG, JAK1, SOCS3, SUMO1, PTPN1, PTPN11 |
| REAC:R-HSA-6803207 | TP53 Regulates Transcription of Caspase Activators and Caspases | 3.35e-03 | 2,5 | 12 | 491 | 6 | 50 | TP53, ATM, CASP1, APAF1, NLRC4, TP63 |
| REAC:R-HSA-6783589 | Interleukin-6 family signaling | 3.57e-03 | 2,4 | 24 | 491 | 8 | 33,33 | IL6, STAT3, STAT1, OSM, JAK1, SOCS3, IL6ST, PTPN11 |
| REAC:R-HSA-912694 | Regulation of IFNA/IFNB signaling | 3.57e-03 | 2,4 | 24 | 491 | 8 | 33,33 | JAK1, IFNA1, SOCS3, PTPN1, PTPN11, STAT2, IFNB1 |
| REAC:R-HSA-445989 | TAK1-dependent IKK and NF-kappa-B activation | 4.56e-03 | 2,3 | 32 | 491 | 9 | 28,12 | RELA, APP, HMGB1, IRAK1, NFKB1, TRAF6, MAP3K7, IKBKB, IKBKG |
| REAC:R-HSA- | TRAF6 mediated NF-kB activation | 5.05e-03 | 2,3 | 25 | 491 | 8 | 32 | RELA, APP, HMGB1, MAP3K1, NFKB1, TRAF6, IKBKB, IKBKG |

| | | | | | | | | |
|---|---|---|---|---|---|---|---|---|
| 933542 | | | | | | | | |
| REAC:R-HSA-5357786 | TNFR1-induced proapoptotic signaling | 5.98e-03 | 2,2 | 13 | 491 | 6 | 46,15 | TNF, CASP8, TNFRSF1A, RIPK1, FADD, CYLD |
| REAC:R-HSA-209560 | NF-kB is activated and signals survival | 5.98e-03 | 2,2 | 13 | 491 | 6 | 46,15 | RELA, SQSTM1, IRAK1, NFKB1, TRAF6, IKBKB |
| REAC:R-HSA-111461 | Cytochrome c-mediated apoptotic response | 5.98e-03 | 2,2 | 13 | 491 | 6 | 46,15 | MAPK3, MAPK1, CASP3, CYCS, CASP9, APAF1 |
| REAC:R-HSA-2559582 | Senescence-Associated Secretory Phenotype (SASP) | 6.13e-03 | 2,2 | 112 | 491 | 17 | 15,18 | MAPK3, IL6, CDKN1A, JUN, MAPK1, STAT3, FOS, CXCL8, RELA, RPS6KA3, RPS6KA1, IL1A, RPS6KA2, CEBPB, NFKB1, CCNA1, EHMT1 |
| REAC:R-HSA-500792 | GPCR ligand binding | 6.29e-03 | 2,2 | 459 | 491 | 42 | 9,15 | CXCL8, CCL2, APP, CXCR4, CXCL12, CCR7, CCL11, GRP, CXCL10, F2R, CCR5, CXCL1, AGTR1, TAC1, FPR2, CCL5, OPRM1, CCL22, C5AR1, HTR2A, C3, RHO, CXCR3, C5, CXCR2, F2RL3, NPY, CCL4, CXCR1, XCR1, CXCL9, CCL3, GAL, |

| | | | | | | | | |
|---|---|---|---|---|---|---|---|---|
| | | | | | | | | WNT3A, PPBP, GRM5, CCL20, CD55, XCL2, CCL17, ANXA1, CCR3 |
| REAC:R-HSA-111471 | Apoptotic factor-mediated response | 6.45e-03 | 2,2 | 19 | 491 | 7 | 36,84 | MAPK3, MAPK1, CASP3, CYCS, CASP9, APAF1, C1QBP |
| REAC:R-HSA-354192 | Integrin signaling | 7.00e-03 | 2,2 | 26 | 491 | 8 | 30,77 | AKT1, CRK, SRC, FN1, PTK2, RAPGEF3, PTPN1, PDPK1 |
| REAC:R-HSA-912526 | Interleukin receptor SHC signaling | 7.00e-03 | 2,2 | 26 | 491 | 8 | 30,77 | JAK1, IL2, JAK3, CSF2, IL3, PIK3R2, IL2RA, IL5 |
| REAC:R-HSA-442742 | CREB1 phosphorylation through NMDA receptor-mediated activation of RAS signaling | 7.00e-03 | 2,2 | 26 | 491 | 8 | 30,77 | MAPK3, MAPK1, HRAS, CREB1, RPS6KA3, RPS6KA1, RPS6KA2, PDPK1 |
| REAC:R-HSA-186763 | Downstream signal transduction | 7.00e-03 | 2,2 | 26 | 491 | 8 | 30,77 | STAT3, CRK, HRAS, SRC, STAT1, STAT6, PIK3R2, PTPN11 |

| | | | | | | | | |
|---|---|---|---|---|---|---|---|---|
| REAC:R-HSA-445144 | Signal transduction by L1 | 9.53e-03 | 2 | 20 | 491 | 7 | 35 | MAPK3, EGFR, MAPK1, MAP2K1, PAK1, NRP1, RAC1 |
| REAC:R-HSA-114604 | GPVI-mediated activation cascade | 1.02e-02 | 2 | 35 | 491 | 9 | 25,71 | FYN, RHOA, CDC42, PIK3R2, COL1A1, PTPN11, PDPK1, RAC1, RAC2 |
| REAC:R-HSA-75157 | FasL/ CD95L signaling | 1.08e-02 | 2 | 5 | 491 | 4 | 80 | CASP8, FASLG, FAS, FADD |
| REAC:R-HSA-451927 | Interleukin-2 family signaling | 1.14e-02 | 1,9 | 44 | 491 | 10 | 22,73 | STAT3, STAT1, JAK1, IL2, JAK3, CSF2, IL3, PIK3R2, IL2RA, IL5 |
| REAC:R-HSA-1236975 | Antigen processing-Cross presentation | 1.19e-02 | 1,9 | 106 | 491 | 16 | 15,09 | TLR4, HMGB1, PSMD9, TLR2, PSMD1, CYBB, MYD88, HLA-A, CD14, CALR, FCGR1A, IKBKB, MRC1, TAP1, TIRAP, IKBKG |
| REAC:R-HSA-186797 | Signaling by PDGF | 1.34e-02 | 1,9 | 54 | 491 | 11 | 20,37 | STAT3, CRK, HRAS, SRC, SPP1, STAT1, STAT6, PIK3R2, COL2A1, PTPN11, PLG |

| | | | | | | | | |
|---|---|---|---|---|---|---|---|---|
| REAC:R-HSA-1606322 | ZBP1(DAI) mediated induction of type I IFNs | 1.37e-02 | 1,9 | 21 | 491 | 7 | 33,33 | RELA, RIPK3, MYD88, NFKB1, RIPK1, IKBKB, IKBKG |
| REAC:R-HSA-2454202 | Fc epsilon receptor (FCERI) signaling | 1.43e-02 | 1,8 | 182 | 491 | 22 | 12,09 | MAPK3, JUN, MAPK1, GRAP2, FOS, HRAS, RELA, MAPK8, PSMD9, PSMD1, PAK1, MAP3K1, FYN, NFKB1, MAP2K4, TRAF6, PIK3R2, PDPK1, RAC1, MAP3K7, IKBKB, IKBKG |
| REAC:R-HSA-202403 | TCR signaling | 1.55e-02 | 1,8 | 120 | 491 | 17 | 14,17 | GRAP2, PTEN, RELA, PSMD9, CD4, PSMD1, PAK1, NFKB1, PTPRC, TRAF6, PIK3R2, PDPK1, MAP3K7, PTPN22, IKBKB, VASP, IKBKG |
| REAC:R-HSA-5669034 | TNFs bind their physiological receptors | 1.70e-02 | 1,8 | 29 | 491 | 8 | 27,59 | FASLG, TNFSF13B, TNFRSF1A, TNFRSF25, LTA, TNFSF8, TNFSF9, CD70 |
| REAC:R-HSA-1227986 | Signaling by ERBB2 | 1.72e-02 | 1,8 | 46 | 491 | 10 | 21,74 | AKT1, EGFR, EGF, ERBB2, HRAS, SRC, HSP90AA1, FYN, RHOA, NRG1 |
| REAC:R-HSA-6806 | Signaling by MET | 1.81e-02 | 1,7 | 76 | 491 | 13 | 17,11 | STAT3, CRK, HRAS, SRC, FN1, MET, COL11A2, PTK2, PTPN1, COL1A1, COL2A1, PTPN11, RAC1 |

| | | | | | | | | |
|---|---|---|---|---|---|---|---|---|
| 834 | | | | | | | | |
| REAC:R-HSA-1474244 | Extracellular matrix organization | 2.00e-02 | 1,7 | 298 | 491 | 30 | 10,07 | MMP9, MMP2, CASP3, FGF2, CDH1, CD44, ICAM1, APP, SPP1, SERPINE1, TIMP1, FN1, MMP13, COL11A2, VCAM1, TTR, KLK2, PECAM1, BGN, BSG, SDC1, ITGAM, P4HB, CTSB, COL1A1, COL2A1, PLG, VTN, ITGAX, ELANE |
| REAC:R-HSA-418594 | G alpha (i) signalling events | 2.04e-02 | 1,7 | 313 | 491 | 31 | 9,9 | MAPK1, CXCL8, SRC, APP, CREB1, CXCR4, CXCL12, CCR7, CXCL10, CCR5, CXCL1, FPR2, CCL5, OPRM1, C5AR1, C3, RHO, GRK2, CXCR3, C5, CXCR2, NPY, CCL4, CXCR1, CXCL9, CCL4L2, GAL, PPBP, CCL20, ANXA1, CCR3 |
| REAC:R-HSA-8864260 | Transcriptional regulation by the AP-2 (TFAP2) family of transcription factors | 2.08e-02 | 1,7 | 38 | 491 | 9 | 23,68 | EGFR, CDKN1A, ESR1, ERBB2, APOE, NPM1, HSPD1, SUMO1, CREBBP |
| REAC:R-HSA-76009 | Platelet Aggregation (Plug Formation) | 2.08e-02 | 1,7 | 38 | 491 | 9 | 23,68 | AKT1, CRK, SRC, FN1, PTK2, RAPGEF3, PTPN1, COL1A1, PDPK1 |

| | | | | | | | | |
|---|---|---|---|---|---|---|---|---|
| REAC:R-HSA-9020591 | Interleukin-12 signaling | 2.10e-02 | 1,7 | 47 | 491 | 10 | 21,28 | IL10, SOD1, IFNG, JAK1, AIP, CDC42, P4HB, ANXA2, CFL1, MIF |
| REAC:R-HSA-9701898 | STAT3 nuclear events downstream of ALK signaling | 2.12e-02 | 1,7 | 10 | 491 | 5 | 50 | STAT3, HIF1A, CD274, HDAC1, HDAC2 |
| REAC:R-HSA-5668541 | TNFR2 non-canonical NF-kB pathway | 2.30e-02 | 1,6 | 100 | 491 | 15 | 15 | TNF, CD40LG, PSMD9, FASLG, PSMD1, TNFSF13B, TNFRSF1A, TNFRSF25, TRAF3, BIRC3, TNFSF12, LTA, TNFSF8, TNFSF9, CD70 |
| REAC:R-HSA-5676594 | TNF receptor superfamily (TNFSF) members mediating non-canonical NF-kB pathway | 2.48e-02 | 1,6 | 16 | 491 | 6 | 37,5 | CD40LG, TNFSF13B, TRAF3, BIRC3, TNFSF12, LTA |
| REAC:R-HSA-399954 | Sema3A PAK dependent Axon repulsion | 2.48e-02 | 1,6 | 16 | 491 | 6 | 37,5 | HSP90AA1, PAK1, FYN, NRP1, RAC1, CFL1 |
| REAC:R-HSA- | Signaling by SCF-KIT | 2.59e-02 | 1,6 | 39 | 491 | 9 | 23,08 | MMP9, STAT3, GRAP2, HRAS, STAT1, FYN, PIK3R2, PTPN11, RAC1 |

| | | | | | | | | |
|---|---|---|---|---|---|---|---|---|
| 1433557 | | | | | | | | |
| REAC:R-HSA-5689896 | Ovarian tumor domain proteases | 2.59e-02 | 1,6 | 39 | 491 | 9 | 23,08 | TP53, ESR1, PTEN, CDK1, RHOA, TRAF3, RIPK1, TRAF6, IKBKG |
| REAC:R-HSA-450302 | activated TAK1 mediates p38 MAPK activation | 2.67e-02 | 1,6 | 23 | 491 | 7 | 30,43 | MAPK14, IRAK1, TRAF6, MAP2K3, MAP3K7, MAP2K6, IKBKG |
| REAC:R-HSA-9658195 | Leishmania infection | 3.08e-02 | 1,5 | 305 | 491 | 30 | 9,84 | MAPK3, IL6, JUN, MAPK1, MAPK14, CRK, HMOX1, IL1B, RELA, APP, CREB1, IL10, MAPK8, ABL1, IL18, NLRP3, CASP1, IL1A, PTK2, FYN, CDC42, NFKB1, C3, FCGR2A, RAC1, FCGR1A, TXN, PYCARD, CD163, DPEP2 |
| REAC:R-HSA-5674499 | Negative feedback regulation of MAPK pathway | 3.13e-02 | 1,5 | 6 | 491 | 4 | 66,67 | MAPK3, MAPK1, RAF1, MAP2K1 |
| REAC:R-HSA-111459 | Activation of caspases through apoptosome-mediated cleavage | 3.13e-02 | 1,5 | 6 | 491 | 4 | 66,67 | CASP3, CYCS, CASP9, APAF1 |

| | | | | | | | | |
|---|---|---|---|---|---|---|---|---|
| REAC:R-HSA-9013957 | TLR3-mediated TICAM1-dependent programmed cell death | 3.13e-02 | 1,5 | 6 | 491 | 4 | 66,67 | CASP8, RIPK3, RIPK1, FADD |
| REAC:R-HSA-8986944 | Transcriptional Regulation by MECP2 | 3.19e-02 | 1,5 | 59 | 491 | 11 | 18,64 | BDNF, PTEN, PPARG, CREB1, MET, HDAC1, IRAK1, OPRM1, PTPN1, HDAC2, FKBP5 |
| REAC:R-HSA-8943724 | Regulation of PTEN gene transcription | 3.19e-02 | 1,5 | 59 | 491 | 11 | 18,64 | MAPK3, TP53, JUN, MAPK1, MTOR, PPARG, EZH2, HDAC1, KDM1A, RPTOR, HDAC2 |
| REAC:R-HSA-8875878 | MET promotes cell motility | 3.21e-02 | 1,5 | 40 | 491 | 9 | 22,5 | CRK, SRC, FN1, MET, COL11A2, PTK2, COL1A1, COL2A1, RAC1 |
| REAC:R-HSA-4090294 | SUMOylation of intracellular receptors | 3.63e-02 | 1,4 | 24 | 491 | 7 | 29,17 | ESR1, PPARG, PPARA, VDR, NR3C1, SUMO1, RARA |
| REAC:R-HSA-5602498 | MyD88 deficiency (TLR2/4) | 3.69e-02 | 1,4 | 17 | 491 | 6 | 35,29 | TLR4, HMGB1, TLR2, MYD88, CD14, TIRAP |

| | | | | | | | | |
|---|---|---|---|---|---|---|---|---|
| REAC:R-HSA-9627069 | Regulation of the apoptosome activity | 3.73e-02 | 1,4 | 11 | 491 | 5 | 45,45 | MAPK3, MAPK1, CYCS, CASP9, APAF1 |
| REAC:R-HSA-111458 | Formation of apoptosome | 3.73e-02 | 1,4 | 11 | 491 | 5 | 45,45 | MAPK3, MAPK1, CYCS, CASP9, APAF1 |
| REAC:R-HSA-383280 | Nuclear Receptor transcription pathway | 3.95e-02 | 1,4 | 41 | 491 | 9 | 21,95 | ESR1, PPARG, PPARA, VDR, NR4A1, NR0B2, NR2C2, RARB, RARA |
| REAC:R-HSA-844615 | The AIM2 inflammasome | 4.96e-02 | 1,3 | 3 | 491 | 3 | 100 | CASP1, AIM2, PYCARD |

| TABLE S9: Transcription factors predicted to be regulated by P.veris metabolites and affected by influenza infection | | | | | | | | |
|---|---|---|---|---|---|---|---|---|
| **Term_ID** | **Function** | **P-value** | **.-log10 Pvalue** | **Term Size** | **Query size** | **Intersection Size** | **Enrichment Score %** | **Positive Hits** |
| TF:M03563_1 | Factor: RelA-p65; motif: GGGANTTTCCNN; match class: 1 | 1.07e-04 | 4 | 895 | 555 | 55 | 6,15 | MMP9, EGF, SIRT1, ICAM1, TNFRSF10B, SYT1, CREB1, DPP4, CASP8, CD40, KCNH4, VCAM1, BECN1, FOXO1, FGFR2, CXCL10, CFLAR, EIF4G1, AIM2, CXCL1, IFNA1, EIF2AK2, IL17C, RAB5A, PTGS1, TRAF1, MYD88, MLKL, FKBP4, NFKB1, TRAF3, TLR7, KLK1, BIRC3, CXCR2, CSRP1, XCR1, CTSE, IL2RA, STAG1, MPRIP, CYLD, IKBKB, IL32, MIF, CD70, WNT3A, CADM1, TAP1, C1D, CD69, IGF2BP1, IFNB1, DPEP2 |

| | | | | | | | | |
|---|---|---|---|---|---|---|---|---|
| TF:M11665 | Factor: IRF-2; motif: NGAAASYGAAAS | 1.26e-03 | 2,9 | 2424 | 555 | 108 | 4,46 | MMP9, MAPK1, HIF1A, CFTR, MAP2K1, TNFRSF10B, ABCB1, TLR4, CD40LG, IL4, HMGB1, FN1, ABCA1, ZNRD2, IFI27, CASP8, FASLG, TLR2, KCNH8, TNFSF10, FGF13, STAT1, SCGB2A2, FOXO1, PRNP, CASP1, CD274, MUC5AC, CXCL10, CFLAR, EIF4G1, AIM2, KLK3, MAP3K5, HDAC1, AIP, TNFSF13B, FPR2, OCLN, FAS, CYBB, NUP62, EIF2AK2, KHDRBS1, GLRX, KDM1A, RPS6KA5, F3, MYD88, APAF1, H3-2, EPCAM, IL6ST, ITGAM, IL7, C5AR1, NR3C1, RIPK1, IL3, IL17RA, MDK, MECP2, IL22, RPTOR, RBBP6, KIR3DL2, MAP2K3, CLDN7, FMR1, C1QBP, HLA-A, RNASE1, XPO1, WT1, FCGR1A, XCR1, AMPH, TXN, THBD, NLRC4, CYLD, IFNL3, HNRNPC, DUSP4, TNFSF9, PYCARD, PPBP, GRM5, PLG, CD28, TAP1, KLRG1, CCL20, CD163, XCL2, GBP1, CD69, IL5, ID2, PNO1, PSAT1, ANXA1, IL1RAPL1, RBMS3, MX1, OTUD4, IDO1, CCR3 |
| TF:M08887 | Factor: IRF; motif: NNGAAANTGAAANN | 2.43e-02 | 1,6 | 1280 | 555 | 63 | 4,92 | TNFRSF10B, ABCB1, TLR4, IL4, FN1, IFI27, FASLG, TLR2, KCNH8, TNFSF10, STAT1, SCGB2A2, PRNP, CASP1, CD274, CXCL10, CFLAR, JAK1, EIF4G1, AIM2, AIP, TNFSF13B, FPR2, FAS, CYBB, EIF2AK2, GLRX, KDM1A, F3, MYD88, IL6ST, ITGAM, MLKL, IL7, IL3, MDK, MECP2, RBBP6, C1QBP, HLA-A, RNASE1, WT1, FCGR1A, XCR1, TXN, CYLD, IFNL3, HNRNPC, DUSP4, PYCARD, PPBP, PLG, C1D, CCL20, XCL2, |

| | | | | | | | | |
|---|---|---|---|---|---|---|---|---|
| | | | | | | | | GBP1, CD69, IL5, IL1RAPL1, MX1, ERVW-1, IDO1, CCR3 |
